# Suppression of hnRNP A1 binding to HK1 RNA leads to glycolytic dysfunction in Alzheimer’s disease models

**DOI:** 10.3389/fnagi.2023.1218267

**Published:** 2023-08-31

**Authors:** Xin-Hao Ji, Ting-Ting Liu, Ai-Hong Wei, Hui-Ping Lei, Yue Chen, Ling-Nan Wu, Ju Liu, Ying Zhang, Fei Yan, Mei-Xiang Chen, Hai Jin, Jing-Shan Shi, Shao-Yu Zhou, Feng Jin

**Affiliations:** ^1^Key Laboratory of Basic Pharmacology of Ministry of Education and Joint International Research Laboratory of Ethnomedicine of Ministry of Education, Zunyi Medical University, Zunyi, Guizhou, China; ^2^Nuclear Medicine and Molecular Imaging Key Laboratory of Sichuan Province, Department of Nuclear Medicine, Affiliated Hospital of Southwest Medical University, Luzhou, Sichuan, China; ^3^Department of Gastroenterology, Digestive Disease Hospital, Affiliated Hospital of Zunyi Medical University, Zunyi, Guizhou, China

**Keywords:** Alzheimer’s disease, neuron, glucose metabolism, hnRNP A1, HK1, APP, β-amyloid, p-p38 MAPK

## Abstract

**Objective:**

To investigate the mechanism of RNA-binding protein hnRNP A1 in mouse hippocampal neurons (HT22) on glycolysis.

**Methods:**

RIP and CLIP-qPCR were performed by HT22 *in vitro* to observe the mechanism of hnRNP A1 regulating the expression of key proteins in glycolysis. The RNA binding domain of hnRNP A1 protein in HT22 was inhibited by VPC-80051, and the effect of hnRNP A1 on glycolysis of HT22 was observed. Lentivirus overexpression of hnRNP A1 was used to observe the effect of overexpression of hnRNP A1 on glycolysis of Aβ_25–35_-injured HT22. The expression of hnRNP A1 in brain tissues of wild-type mice and triple-transgenic (APP/PS1/Tau) AD mice at different ages was studied by Western blot assay.

**Results:**

The results of RIP experiment showed that hnRNP A1 and HK1 mRNA were significantly bound. The results of CLIP-qPCR showed that hnRNP A1 directly bound to the 2605-2821 region of HK1 mRNA. hnRNP A1 inhibitor can down-regulate the expression of HK1 mRNA and HK1 protein in HT22 cells. Overexpression of hnRNP A1 can significantly reduce the toxic effect of Aβ_25–35_ on neurons via the hnRNP A1/HK1/ pyruvate pathway. In addition, inhibition of hnRNP A1 binding to amyloid precursor protein (APP) RNA was found to increase Aβ expression, while Aβ_25–35_ also down-regulated hnRNP A1 expression by enhancing phosphorylation of p38 MAPK in HT22. They interact to form bidirectional regulation, further down-regulating the expression of hnRNP A1, and ultimately aggravating glycolytic dysfunction. Protein immunoblotting showed that hnRNP A1 decreased with age in mouse brain tissue, and the decrease was greater in AD mice, suggesting that the decrease of hnRNP A1 may be a predisposed factor in the pathogenesis of AD.

## Highlights

- Brain glycolytic dysfunction is involved in the development of Alzheimer’s disease (AD).
- hnRNP A1 is significantly reduced in the AD models.
- hnRNP A1 binds directly to HK1 mRNA to regulate glycolytic function.
- A bidirectional regulation between hnRNP A1 and Aβ exacerbates glycolytic dysfunction.

## Introduction

Alzheimer’s disease (AD) is the most common neurodegenerative disease (Abeysinghe et al., 2020). The behavioral features of AD are memory loss and cognitive decline (Lei et al., 2021). The pathology is characterized by amyloid plaques and neurofibrillary tangles (NFTs) composed mainly of β-amyloid (Aβ) peptides and hyperphosphorylated tau, respectively (Ju and Tam, 2022).

In recent years, there has been increasing evidence that abnormal glucose metabolism (Butterfield and Halliwell, 2019) in the brain are early markers of AD (Wang et al., 2022). Glycolysis is a common stage of glucose catabolism, which is critical for the formation of neuronal networks (Segarra-Mondejar et al., 2018), and the ability to learn (Shannon et al., 2016) and remember (Pan et al., 2022). In patients with AD, metabolic intermediates of glycolysis are significantly reduced and the reduction of glycolytic intermediates is positively correlated with Aβ_1–42_ and Aβ_1–42_/Aβ_1–40_ levels (Bergau et al., 2019). Three key rate-limiting enzymes control glycolytic flux: hexokinase (HK), 6-phosphofructokinase-1 (PFK) and pyruvate kinase (PK) (Zhang et al., 2021). HK is the first rate-limiting enzyme of the glycolytic pathway, which is required for glucose phosphorylation (Wilson, 2003). From the current findings, it has been established that impaired glucose metabolism is affected by downregulation of HK protein expression and activity through the WNT signaling pathway in APP/PS1 transgenic AD mice (Cisternas et al., 2019). It is noteworthy that HK has four isozymes and HK1 is expressed mainly in neurons (Leng et al., 2022). However, molecular mechanisms leading to the downregulation of HK, especially the aberrant transcription of HK1 in AD neurons, have not been described.

Heterogeneous nuclear ribonucleoprotein A1 (hnRNP A1) is an important RNA binding protein (RBP), which is highly expressed in neurons. The main function of hnRNP A1 is to regulate various RNA metabolic processes, including transcription, alternative splicing of pre-mRNA, translation, miRNA processing and mRNA stability (Clarke et al., 2021). Significant variation in the hnRNP A1 gene has been established between AD patients and controls in the study of AD risk genes (Xiao et al., 2021). Knockdown of hnRNP A1 is known to cause cellular senescence, and dysfunction of RBP is the basis of neurodegeneration in neurological diseases (Shimada et al., 2009). hnRNP A1 is an important component of the spliceosome that contributes to structural and selective mRNA splicing. hnRNP A1 primarily acts as a negative cis splicing element that binds to exon splice silencing elements or intron splice silencing elements in pre-mRNAs, resulting in preventing exon recognition or promoting exon exclusion in mRNAs (Han et al., 2005). Recent studies have shown that anti-hnRNP A1 antibodies can lead to increased hnRNP A1 mislocalization and nuclear depletion in neurons, ultimately leading to neuronal death (Libner et al., 2022). Yet, it is currently uncertain how hnRNP A1 is engaged in the progressive loss in neuronal function and the development of neurodegenerative diseases with age (Sikora et al., 2021). Another study reported that hnRNP A1 could regulate glucose metabolism in tumor cells by regulating pyruvate kinase type M2 (PKM2), a key enzyme of aerobic glycolysis in tumor cells (Luan et al., 2015). Therefore, we hypothesized that hnRNP A1 may be involved in the pathogenesis of AD through transcriptional regulation of HK1, a key glycolytic enzyme highly expressed in neurons, ultimately leading to glycolytic dysfunction.

To dissect the contribution of hnRNP A1 to the regulation of HK1 in neurons leading to glycolytic dysfunction. In this study, 3 × Tg-AD mice and Aβ_25–35_-induced HT22 cells was used to observe neuronal glycolytic dysfunction, the expression of HK1 and hnRNP A1. We also observed the effect of hnRNP A1 overexpression on HK1 and glycolytic function. Applying CHIP-qPCR and RNA Immunoprecipitation (RIP), we further investigated the regulatory role of hnRNP A1 on HK1.

## Materials and methods

### Mice

Model mice (Stock Number: 034830-JAX, RRID:MMRRC_034830-JAX) with APP, PS1 and Tau mutations were purchased from the Jackson Laboratory (Bar Harbor, ME, USA), and were housed and bred in the SPF class animal house of the Key Laboratory of Basic Pharmacology, Ministry of Education, Zunyi Medical University. During the breeding process, the offspring mice were genotyped according to the method provided by Jackson Laboratory, and the APP, PS1 and Tau genes containing mutations were identified. Non-transgenic B6129SF2/J wild-type (WT) mice (Stock Number: 101045, RRID:IMSR_JAX:101045) were obtained from the Jackson Laboratory (Bar Harbor, ME, USA). Wild-type mice and model mice were bred in the same environment and at the same time period. All mice were fed and watered freely in a 12-h day/night cycle with relatively constant room temperature (18–25°C) and humidity (40–70%). This experiment complied with the guidelines related to experimental animal welfare and ethics.

### Morris water maze (MWM)

The Morris water maze apparatus (TopScan Version 3.00, CleverSys Inc) consisted of a white circular water basin with a diameter of 120 cm and a height of 50 cm, a hidden platform and an automatic recording and analysis system. The water basin was divided into four quadrants: 1, 2, 3 and 4, and the hidden platform was always fixed in the third quadrant. During the experiment, the mice were placed into the water facing the basin wall, and the first 1 to 4 days were used for positioning navigation test, and the time to find the hidden platform within 60 s was recorded. If the mice did not find the hidden platform within the specified time, the latency period of escape was 60 s and the mice were guided to the platform for 15 s. On day 5, the platform was removed. The trajectory of the mice within 60 s and their residence time in the target quadrant were recorded.

### Rotating bar experiment

ZH-300 fatigue rotating rod instrument was purchased from Anhui Zhenghua Biologic Apparatus Facilities Co., Ltd. The rotating bar experiment was used to test the motor function of mice. The mice were first acclimatized. The experiment was started at 10 rpm for each mouse, and the rpm was increased by 5 rpm every 60 s. The upper limit of the test was 300 s. If the movement time was greater than 300 s, it was recorded as 300 s. The mice were trained for 3 days, once a day, and the average of the 3 times was taken for statistical purposes.

### Open field experiments

The open field (TopScan Version 3.00, CleverSys Inc) was composed of four experimental boxes of the same size, divided into the middle area and the peripheral area. Mice were placed from a fixed position and the camera simultaneously tracked the movement trajectory of four mice in each experimental box for 5 min. To avoid any effect on the subsequent experiments, 75% alcohol was used to wipe the residues of feces and urine after each mouse was tested.

### Nesting experiments

Nesting experiments are commonly used to assess daily activities in cognitively impaired mice, and increasing age and damage to the hippocampus and other parts of the body can lead to a gradual decrease in nesting behavior. Therefore, we referred to the literature (Xu et al., 2003) to observe the nesting behavior of mice. Clean cages and bedding were changed 1 h before the experiment, wood chips covered the bottom of the cages with a thickness of approximately 0.5 cm, and a piece of 5 cm × 5 cm compressed cotton was placed in each cage, which was observed and recorded after 12 h. The nesting scoring criteria were as follows: 1: no visible changes in nesting material; 2: partial shredding of nesting material; 3: most of the nesting material was shredded but disorganized without a definite direction; 4: most of the nesting material was shredded but the nest built was flat; and 5: the nest was completely built.

### Brain tissue preparation

After behavioral testing of the learning memory ability and autonomous exploration ability of mice, three mice were randomly selected from each group, anesthetized with sodium pentobarbital (Sigma-Aldrich; Merk KGaA; Darmstadt, Germany) intraperitoneally, and fixed on a homemade surgical plate. The heart was exposed by opening the chest, and a perfusion needle was inserted and fixed from the apical position. PBS buffer (0.1 M) was slowly injected until the liver and lungs turned white in color, followed by 4% paraformaldehyde solution. The brain was fixed by perfusion, and the head was severed. The dissected brains were quickly and carefully fixed in 4% paraformaldehyde solution for 48 h (4°C). After complete fixation, brain tissues were cut into appropriate sizes and then neatly placed in the embedding boxes, washed to remove the fixative and dehydrated transparently, and routinely paraffin-embedded. The rest of the mice were executed by the same method, and the brains were quickly removed on the ice box and stored in the −80°C refrigerator for backup.

### Preparation of Aβ_25–35_ solution

Aβ_25–35_ was synthesized by Sangon Biotechnology (P26152). Aβ_25–35_ powder was added to Phosphate Buffer Solution (P1020, Solarbio) to make the final concentration of 1 mM. The solution was placed in a 37°C incubator for 7 days and then stored at −20°C.

### Cell lines and reagents

HT22 cells were cultured in DMEM medium (C11995500BT, GIBCO) containing 10% FBS (C04001500, VIVACELL) and 1% penicillin-streptomycin (P1400, Solarbio) at 37°C and 5% CO_2_, and passaged every 2–3 d. The lentiviruses of hnRNP A1 and HK1 were provided by HanBio. Sodium pyruvate (P8380, Solarbio) and medium (PM150271, Procell) were used when sodium pyruvate was used as the sole carbon source for the cells. The following inhibitors were used in the cell experiments: 2-Deoxy-D-glucose (HY-13966, MCE), UK-5099 (HY-15475, MCE), VPC-80051 (Sangon), p38 MAPK-IN-1 (HY-12839, MCE). Dosage of drugs: 2-DG (1 mM); VPC-80051 (50 μM); UK5099 (30 μM); p38 MAPK-IN-1 (40 μM); Aβ_25–35_ (20 μM).

### Primary mouse hippocampal neuron extraction

Brain tissues of model and wild-type mice within 24 to 48 h after birth were collected and placed in precooled DMEM/F-12 medium. The tissue was then transferred to a 15 mL glass centrifuge tube and the hippocampus was mashed with a disposable plastic tip dropper. F-12 containing 0.125% trypsin (C25200056, GIBCO) was then added, and the tissue homogenate was placed in an incubator at 37°C with a volume fraction of 5% CO_2_ saturated humidity for 15 min. An appropriate amount of DMEM/F-12 medium was added to terminate the digestion, then filtered through a mesh sieve into a 15 mL centrifuge tube and centrifuged for 8 min (1000 rpm/min). The supernatant from the centrifuge tube was discarded, and the resultant cell pellet was resuspended with neuron cell seeding medium and mix evenly. The cells were counted with an automatic counter. According to the needs of the experiment, the cells were seeded into 6-well culture plates coated with poly-D-lysine at a density of 5 **×** 10^5^/ml, and cultured in an incubator at 37°C with a volume fraction of 5% CO_2_ saturated humidity. After culturing for 4 h, the seeding solution was replaced with cell maintenance solution, and cytarabine solution (A607079, SANGON BIOTECH) was added 48 h after neuron inoculation to inhibit the proliferation of glial cells. After that, the cell maintenance solution was changed every 2–3 d. After 7 days of culture, Aβ_25–35_ was added to make a cellular AD model. Planting solution used in this study: 10% fetal bovine serum (C04001500, VIVACELL), 90% DMEM/F-12 (C11330500BT, GIBCO); maintenance solution: 97% Neurobasal-A (10888022, GIBCO), 2% B27 (17504044, GIBCO), 1% Glutamine (A2916801, GIBCO).

### Hematoxylin-eosin and Nissl staining

Paraffin was cut into 5.0 μm thick sections and baked at 60°C for 30 min. Slices were dewaxed with xylene and gradient ethanol. HE staining: hematoxylin solution 10 min → distilled water wash 3 s → hydrochloric acid ethanol fractionation 2 s → tap water rinse 10 min → eosin staining 2 min → tap water rinse 10 min. Finally, the sections were routinely dehydrated, cleared and sealed. Nissl staining: 1% toluidine blue staining: After dewaxing, toluidine blue solution was added dropwise to the sections for 30 min (60°C) and rinsed in running water for 5 min. Finally, the sections were routinely dehydrated, transparent and sealed. The sections were observed using a light microscope (BX43; Olympus).

### Tissue immunofluorescence staining

Paraffin embedded tissues were cut at a thickness of 5 μm. Oven baking the pieces at 60°C for 35 min. They were deparaffinized by xylene and dehydrated in graded series of pure alcohol and graded alcohol until distilled water. 1% H_2_O_2_ pipette was applied to the tissue sections and placed in a damp box sheltered from light for 15 min. After being washed with PBS, the citric acid buffer was placed in microwave oven at high temperature for antigen repair. Then, tissue were blocked with goat serum (ZSGB-BIO) for 50 min (37°C). Tissue sections were combined with Aβ primary antibody (1:400, ab201060, Abcam) and incubated overnight at 4°C. Sections were then incubated with fluorescent secondary antibody (1:400; SA00013-2, Proteintech) for 50 min at 37°C in a light-proof environment. The sections were then stained with DAPI (C0065, Solarbio) for 6 min at 37°C. Slides were mounted with anti-fluorescence quencher (S2100, Solarbio). Finally, they were observed under a fluorescent microscope and photographed (OLYMPUS, BX53).

### Cell immunofluorescence staining

HT22 was spread on 24-well plates at 20,000/well. After completion of modeling, 24-well plates were washed with PBS and cells were fixed with 4% fixative (P1110, Solarbio) for 20 min and 0.5% Triton X-100 (T8200, Solarbio) for 2 h at room temperature. Cells were sealed with goat serum (ZLI-9022, ZSBG-BIO) for 50 min. Cells were incubated overnight at 4°C with primary antibody hnRNP A1 (1:100; #8443S, Cell Signaling Technology). The sections were then stained with DAPI (C0065, Solarbio) for 6 min. Slides were mounted with anti-fluorescence quencher (S2100, Solarbio). Finally, they were observed and photographed under a 60-fold oil-based fluorescence microscope (OLYMPUS, BX53).

### Western blotting

Brief steps were as follows: extraction of total protein from cells (R0010, Solarbio) → protein quantification using BCA kit (GK5012, GENERAY) → protein denaturation at 95°C for 10 min → preparation of SDS-PAGE gels at 10% → loading volume (20 μg) → electrophoresis → electrotransfer (7 min, 25 V, 2.5 A) → blocking of non-specific antigens for 2 h (5% skim milk) → reaction with primary antibody (overnight at 4°C) → reaction with secondary antibody (1 h at room temperature) → imaging (Bio-Rad). The band intensities were quantified with ImageJ software. The following primary antibodies were used: p38 MAPK (1:1500; #8690, Cell Signaling Technology), p-p38 MAPK (1:1500; #4511, Cell Signaling Technology), hnRNP A1 (1:1000; #8443S, Cell Signaling Technology), HK1 (1:2000; 19662-1-AP, Proteintech), Aβ (1:1000; ab201060, Abcam), APP (1:1000; 25524-1-AP, Proteintech), β-Tubulin (1:5000; 10068-1-AP, Proteintech), IgG (1:5000; SA00001-2, Proteintech).

### Mitochondrial membrane potential

Mitochondrial membrane potential was assayed according to the manufacturer’s (M8650, Solarbio) instructions. First, the medium of the 6-well plate was removed and washed with PBS. Then, 1 ml cell culture medium and 1 mL JC-1 (1 ×) staining working solution were added to each well. The cells were incubated at 37°C for 30 min. After washing with JC-1 buffer, 1 mL medium was added to each well and observed under an inverted fluorescent microscope (OLYMPUS, IX73P2F).

### RT-qPCR

Brief description of the procedure: RNAiso Plus Kit (9108, TaKaRa) to extract RNA from HT22 cells → PrimeScript RT kit (RR037A, TaKaRa) and thermal cycler (C1000 Touch, BIO-RAD) for reverse transcription of RNA → amplification of cDNA on a real-time PCR detector (CFX Connect, BIO-RAD) to amplify cDNA. The thermal cycling procedure was as follows: 95°C (3 min) → 95°C (10 s) → 60°C (45 s) → 40 cycles repeated. Reactions consisted of 7.5 μL TB Green Premix Ex Taq II (RR820A, TaKaRa), 4 μL nucleic acid-free water, 0.5 μL primer, and 3 μL cDNA. Data were analyzed using the comparative cycle threshold (Ct) method (ΔΔCt). The synthesized primers (Sangon Biotech) are listed in Table 1.

**TABLE 1:** List of qPCR primers.

Primers	Forward (5′-3′)	Reverse (5′-3′)
HK1	GGATGGGAACTC TCCCCTG	GCATACGTGCTGG ACCGATA
β-actin	GGCATAGAGGTCTTTA CGGATGTC	TATTGGACGAG CGGTTCC
PKM1	CACCGTCTGC TGTTTGAAGA	TCAAAGCTGCTG CTAAACACTT
PKM2	AGGCTGCCATCT ACCACTTG	CACTGCAGCACT TGAAGGAG

### Measurement of ATP levels

The ATP content was detected using a Luminescent ATP Detection Assay Kit (ab113849, abcam). HT22 cells were digested with trypsin and spread into 96-well plates in the same number of cells in a 50 μL system. Detergent solution (solution A) was added and incubated for 5 min, then substrate solution (solution B) was added and incubated for 5 min. The 96-well plates were placed in a light-proof environment for 9 min and finally the luminescence was measured with a multifunctional microplate reader (Varioskan LUX, ThermoFisher). The excitation wavelength is 488 and the emission wavelength is 525.

### Measurement of pyruvate content

Pyruvate content was measured according to the manufacturer’s instructions (BC2200, Solarbio). HT22 cells were collected into a centrifuge tube, and the supernatant was discarded after centrifugation (200 g, 5 min). The extract was added in appropriate proportions and crushed by ultrasound (Scientz, Scientz-IID). Hippocampal tissue was added to the extract according to the ratio of about 0.1 g tissue to 1mL extract and homogenized in an ice bath. The solution was allowed to stand for 30 min, centrifuged at 8000 g for 10 min at room temperature, and the supernatant was taken to be measured. Reagent II was added after standard solution or sample was mixed with reagent I in a 96-well plate, and absorbance A was determined at a wavelength of 520 nm using a microplate reader (MuitisKan, ThermoFisher). Finally, the pyruvate content was calculated after measuring the protein concentration of the samples using the BCA method.

### Enzyme activity assay of key glycolytic enzymes

The enzymatic activities of HK, PFK and PK were assayed according to the instructions of the commercial kits (BC0745, Solarbio; BC0535, Solarbio; and BC0545, Solarbio). Cells or tissues were collected into centrifuge tubes and extracts were added in appropriate proportions. Cells were crushed with ultrasound (Scientz, Scientz-IID) and tissues were homogenized in ice bath. The extracts were then centrifuged at 8000 g for 10 min at 4°C. The supernatant was taken for testing. The reagents were added sequentially to the 96-well plate according to the instructions. After mixing well, the absorbance difference values were measured at different times on a microplate reader (MuitisKan, ThermoFisher) according to the instructions. The wavelength of the microplate reader was set to 340 nm.

### Cell viability assay

The thiazolyl blue powder (IM0280, Solarbio) was dissolved in PBS (P1020, Solarbio) and stored in the dark at 4°C. HT22 cells were plated at 2000 cells/well in a 96-well plate in a volume of 100 μL. Following drug intervention for 24 h, HT22 cells were given 20 μL of thiazolyl blue solution. The 96-well plate was placed in a 37°C incubator and incubated in the dark for 4 h. After aspirating the medium, 100 μL of DMSO was added to dissolve the purple precipitate at the bottom. Finally, absorbance was determined at a wavelength of 490 nm using a microplate reader (MuitisKan, ThermoFisher).

### CLIP-qPCR

The experiment was performed by IEMed Guangzhou Biomedical Technology Co, Ltd. Crosslinking-immunprecipitatio assays were performed by using the CLIP Kit (IEMed, K319) along with the hnRNP A1 antibody (#8443S, Cell Signaling Technology). HT22 cells were irradiated with 254 nm UV lamp at a dose of 0.15 J/cm^2^ for cross-linking for 1 min. PBS was added into the culture plate. Cells were scraped off with a cell scraper and transferred to a centrifuge tube, centrifuged at 1000rpm for 5 min at 4°C, and supernatant was removed. 2 mL of cell lysis buffer and 20 μL of protease inhibitor were added to cell precipitation. IP and IgG groups were incubated on a vertical mixer with shaking at room temperature for 1 h. The beads were collected on a magnetic rack. 500 uL of CLIP wash buffer was added and washed with shaking at room temperature for 3 min, and the beads were collected on a magnetic rack. Input group, IP group and IgG group were added with 50 μL CLIP washing buffer and 1 μL RNase T1 respectively. Each group was incubated at 37°C for 1 h. Beads of IP group and IgG group were collected on the magnetic rack, and then mixed upside down with 200 μL CLIP washing buffer. Beads were collected on the magnetic rack and repeated twice. The Input group, IP group and IgG group were added with 50 μL PK buffer and 10 μL protease K for digestion at 55°C for 1 h. 120 μl nucleic acid BF C and 10 μL magnetic beads were added to each tube, incubated at room temperature for 20 min, and the magnetic beads were collected on the magnetic rack. Each group was added with 60 μL nucleic acid BF C, which was collected on the magnetic rack immediately and repeated twice. After drying at room temperature for about 10 min, 20 μL RNase-free water was added and the supernatant RNA was collected on the magnetic rack after 3 min of elution at room temperature. Finally, qPCR was performed to detect the enrichment. The synthesized primers are listed in Table 2.

**TABLE 2:** List of qPCR primers.

Primer number	Starting position	Primer sequence
**Primer design for APP mRNA (ID: NM_001198823.1** **Length: 3357)**		
P7107	104	TCTTCCACTCGCACACGG
P7108	410	AGCCAACCAGCCAGTGAC
P7109	482	CGTGATTCCTTACCGTTGCC
P7110	590	TCTTCACTGGCACACCGTC
P7111	622	GCAGCGAGAAGAGCACTAAC
P7113	783	GGAGCGGACACAGACTACG
P7114	837	GAAGAGGAGGAAGTGGCTGAT
P7115	873	GCTGATGATGATGAGGATGTGG
P7116	902	GGACGAGGTGGAGGAGGA.
P7117	973	CCACCACAACCACCACTGA
P7118	1006	TGGTCCGAGAGGTGTGCT
P7119	1122	GGCGGCAACAGGAACAAC
P7120	1169	GTGTGGCAGCGTGTCAAC
P7121	1343	GGAAGCCAAGCACCGAGA
P7122	1388	GGAAGAGGCAGAGCGTCAA
P7123	1475	GGAACAGGAAGCAGCCAATG
P7124	2033	TGTGCCAGCCAATACCGAA
**Primer design for HK1 mRNA (ID: NM_010438.3** **Length: 4100)**		
P7068	2585	TTCCTCTTCCGGGGACAGAT
P7069	990	TTAACACGGAATGGGGAGCC
P7070	2661	AGAGTGACCGATTAGCGCTG
P7071	1691	CGGCAGATTGAGGAAACCCT
P7072	3681	GTGATGGTGGTGGTGGTAGG
P7073	2081	CTGGGCTTCACCTTCTCGTT
P7074	3397	GACTGTCTTCTGGGCCCTTC
P7075	2267	GGCACCATGATGACTTGTGC
P7076	3235	CGCGTGCTGTTGATGATCTG
P7077	3950	AAGCTCCCCAGGTATGCCTA
P7078	802	GAAGCGAGGGGACTATGACG
P7079	2822	ATCCGAGAGAACAGAGGCCT
P7080	505	GCAGGTGAACCACGAGAAGA
P7081	439	CGGCTCAGAAAAGGGGGATT
P7082	4042	TCCAACATGTGAGCCCATCC
P7084	48	CCCCCTCTTCTCAAGGCTTG
P7085	3674	TTGTCTCGTGATGGTGGTGG
P7086	1377	CGTCTCACGATGACTGCGTA

### RNA immunoprecipitation (RIP)

RNA immunoprecipitation assays were performed by using the RIP Kit (IEMed, K303) along with the hnRNP A1 antibody (1:50; #8443S, Cell Signaling Technology). Two 10 μL of Protein A/G beads (labeled as IP and IgG, respectively) were added to 100 μL of AB binding buffer and mixed by flicking, then the beads were collected on a magnetic stand and the supernatant was removed; IP group: 100 μL of AB binding buffer and 2 μg of hnRNP A1 antibody were added; IgG group: 100 μL of AB binding buffer and 2 μL of IgG antibody were added and mixed by flicking, then the beads were collected on a magnetic stand and the supernatant was removed. For the IgG group, 100 μL of AB binding buffer and 2 μL of IgG antibody were added and mixed with flicking. The mixture was incubated at room temperature for 30 min, then the beads were collected on a magnetic rack and the supernatant was removed; for the IP and IgG groups, 100 μL of AB binding buffer were added and mixed upside down, then the beads were collected on a magnetic rack and 80 μL of supernatant were removed; collect 10^7^ HT22 cells were washed twice with 1 × PBS, and the last time the residual PBS was aspirated; 700 μL of 2 × RIP buffer, 20 μL of Protease inhibitors, 100 μL of RNase inhibitor were added to the cell precipitate, mixed with sufficient blowing, and then lysed at room temperature for 20 min; 700 μL of Binding enhancer was added. Binding enhancer, mix upside down, centrifuge at 12000g for 10 min at 4°C, transfer supernatant to new RNA-free centrifuge tube; 5 μL of Clean beads were added to supernatant, incubate on vertical mixer at room temperature with shaking for 20 min; 800 μL of supernatant were transfered to IP sample tube on magnetic stand; 400 μL of supernatant were transfered to IgG sample tube. 800 μL of supernatant were transfered to the IP sample tube and 400 μL of supernatant to the IgG sample tube; 100 μL of supernatant were transfered (labeled as Input group and stored at −80°C for use); The IP and IgG groups were combined by incubation on a vertical mixer with shaking at room temperature for 60 min; The beads were collected on the magnetic stand and removed the supernatant; 500 μL of RIP washing buffer were added to each of the IP and IgG groups and washed with shaking at room temperature for 5 min; then the beads were collected on the magnetic stand and removed the supernatant. 50 μL of Polysome elution buffer were added to the IP and IgG groups, mixed with a light flick, and then eluted at 75°C for 10 min; 100 μL of Nucleic acid BF C were added to the IP and IgG groups and 200 μL of Nucleic acid BF C to the Input group. 10 μL Magnetic beads were added, shaked and combined for 10 min at room temperature; The beads were collected on the magnetic rack to remove the supernatant; 100 μL Nucleic acid BF C were added to each group, flicked and mixed, then removed the supernatant directly on the magnetic rack; after drying at room temperature (about 10 min), 20 μL RNase-free water were added to each group elute at room temperature for 3 min, and collected the supernatant on the magnetic rack RNA. HK1 mRNA levels in the immunoprecipitates were measured by qPCR. Finally, the binding situation was judged by the enrichment efficiency.

### GSH content test

No less than 5 million cells were collected according to glutathione determination instructions (BC1175, Solarbio). The cells were washed with PBS and then resuspended with 1 mL reagent A. The cells were ultrasonically broken (Scientz, Scientz-iID) and centrifuged. Then, supernatant measurements were collected. The wavelength was adjusted to 412 nm with an enzyme marker (MuitisKan, ThermoFisher). Finally, the GSH content was calculated according to the number of cells.

### ROS content test

A concentration of 1 ml DCFH-DA (10 μM) was added to a six-well plate with the medium removed according to the instructions of the Reactive Oxygen Species Test Kit (CA1410, Solarbio). The cells were cultured in an incubator for 30 min. HT22 cells were digested with trypsin and spread into 96-well plates in the same number of cells in a 50 μL system. Finally, the luminescence was measured with a multifunctional microplate reader (Varioskan LUX, ThermoFisher). The excitation wavelength is 488 and the emission wavelength is 525.

### ^18^F-FDG MicroPET

The experiment was completed under the experimental platform provided by the Sichuan Key Laboratory of Nuclear Medicine and Molecular Imaging. Mice were injected intraperitoneally with sodium barbiturate, and after complete anesthesia, approximately 14.8–16.5 mBq of ^18^F-FDG (prepared by the Sichuan Key Laboratory of Nuclear Medicine and Molecular Imaging) was injected via the tail vein. The mice were fixed in prone position on the scanner bed. The long axis of the head was parallel to the long axis of the scanner and within the field of view of the scanner. 20 min of ^18^F-FDG uptake in the mice was followed by 10 min of PET image acquisition and 10 min of CT image acquisition. The data were then processed using SIEMENS Inveon Research Workplace V4.2 software. ROI selection: The 3D ROI technique was used to manually select the 3D ROI of the whole brain, hippocampal and cortical regions in the cross-sectional, coronal and sagittal planes with reference to the mouse brain *ex vivo* localization spectrograms, and the average ROI per gram of tissue within the ROI was calculated of uptake rate.

### Statistical analysis

Statistical analysis was performed using GraphPad software Prism 8.0. All data were expressed as mean ± standard error (mean ± SEM). Student’s *t*-test and one-way analysis of variance (ANOVA) were used to determine the levels of statistical differences between two and multiple groups, respectively. Statistically significant differences were set at *p < 0.05, **p < 0.01, *** *p* < 0.001.

## Results

### Glycolytic dysfunction in AD models

**FIGURE 1:**
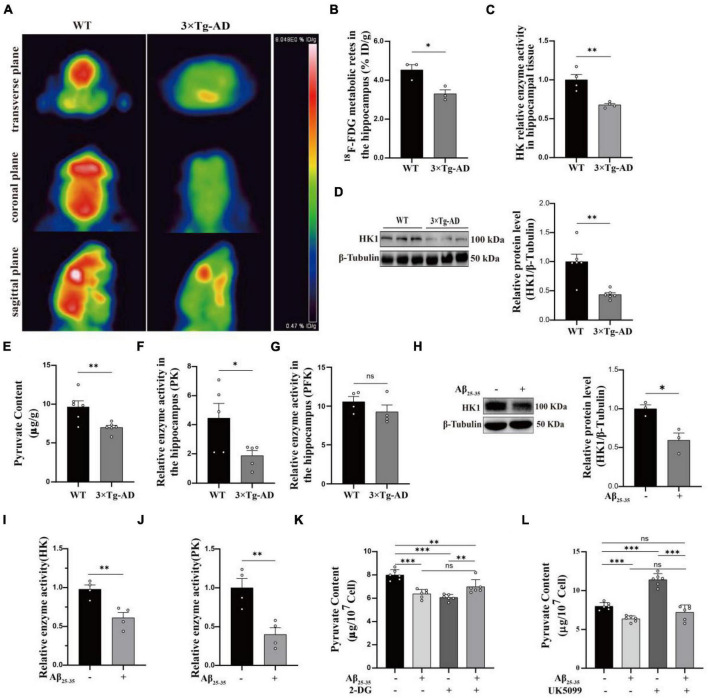
Glycolytic dysfunction in AD models. **(A,B)** ^18^F-FDG MicroPET analysis: glucose utilization in the hippocampus of 3 × Tg-AD mice (*n* = 3 mice per group). **(C)** Kit to analyze altered HK enzyme activity in the hippocampus of 3 × Tg-AD mice (*n* = 6 mice per group). **(D)** Western blotting analysis: protein expression of HK1 in the hippocampus of 9-month-old WT and 3 × Tg-AD mice and quantitative statistics (*n* = 6 mice per group). **(E)** Pyruvate content in hippocampal tissue of 9-month-old WT and 3 × Tg-AD mice (*n* = 6 mice per group). **(F,G)** PFK and PK enzyme activity in the hippocampus of 3 × Tg-AD mice (*n* = 6 mice per group). **(H)** Western blotting analysis: role of Aβ_25–35_ on HK1 protein expression of HT22 cells and quantitative statistics (*n* = 3 per group). **(I,J)** HK and PK enzyme activity in HT22 cells (*n* = 4 per group). **(K)** The effect of Aβ_25–35_ on pyruvate entering mitochondria as observed by using 2-DG in HT22 cells (*n* = 3 per group). **(L)** The effect of Aβ_25–35_ on pyruvate production via glycolysis as observed by using UK5099 in HT22 cells (*n* = 3 per group). Statistical analysis was performed using GraphPad software Prism 8.0. All data were expressed as mean ± standard error (mean ± SEM). Student’s *t*-test and one-way analysis of variance (ANOVA) were used to determine the levels of statistical differences between two and multiple groups, respectively. Statistically significant differences were set at *p < 0.05, **p < 0.01, ***p < 0.001.

Numerous studies have pointed that AD is inextricably linked to impaired glycolysis (Lee et al., 2016). The 3 × Tg-AD mice have been used successfully to model Alzheimer’s disease for years (Chen et al., 2022). As illustrated in the schematic diagram (Supplementary Figure 1A), the selected time points represented corresponding age stages for conducting animal experiments. Our previous studies have shown Aβ deposition in the brains of 9-month-old AD mice, so we selected 9-month-old mice for follow-up experiments (Yan et al., 2023). ^18^F-FDG MicroPET showed reduced glucose utilization in the hippocampal region of 3 × Tg-AD mice compared to WT group mice in 9-month-old (Figures 1A, B). This phenomenon is mainly related to both glucose uptake and reduced HK activity (Figure 1C). We found that reduced HK activity was associated with reduced HK1 expression in the hippocampal tissue of 9-month-old 3 × Tg-AD mice (Figure 1D). It is suggested that reduced HK1 expression is involved in AD pathogenesis. HK is the first rate-limiting enzyme of the glycolytic pathway and controls the entire glycolytic flux. Of these, brain tissue is predominantly dominated by HK1, so we proceeded to assess whether hippocampal tissue glycolytic function is impaired in the AD model. We assessed the content of pyruvate, the end product of glycolysis, in the hippocampal tissue of 9-month-old mice. The pyruvate biochemical kit showed a significant decrease in pyruvate levels in hippocampal tissue of 3 × Tg-AD mice compared to the WT group (Figure 1E). This was associated with reduced activity of HK (Figure 1C) and PK (Figure 1F) enzymes among the three key enzymes of glycolysis in hippocampal tissue of 9-month-old 3 × Tg-AD mice. No change in PFK enzyme activity was found (Figure 1G). The HT22 cell line is an immortalized mouse hippocampal neuron cell line with properties similar to those of mature hippocampal neurons *in vivo*, such as neurite growth, expression of functional cholinergic markers and receptors, which allows HT22 cells to be utilized to characterize the molecular events underlying neurodegeneration and neuronal injuries. We used HT22 to administer exogenous Aβ_25–35_ to simulate the AD model to explore the effect of Aβ on neurons. Similarly, we observed a significant reduction in HK1 protein expression (Figure 1H), HK enzyme activity (Figure 1I), PK enzyme activity (Figure 1J) and its end product pyruvate (Figure 1K) in Aβ_25–35_-induced HT22 cells, which is in line with the findings of animal tests. To further verify that the decrease in pyruvate during glucose metabolism was a result of decreased glycolytic production rather than increased oxidative phosphorylation into the mitochondria, we designed to use a glucose analog (2-DG) to inhibit glycolysis and a mitochondrial pyruvate carrier inhibitor (UK5099) to inhibit mitochondrial uptake of pyruvate. When 2-DG was intervened, the 2-DG + Aβ_25–35_ group exhibited increased pyruvate levels compared to the 2-DG group (Figure 1K). Aβ_25–35_ may have inhibited oxidative phosphorylation of pyruvate into mitochondria, resulting in increased pyruvate abundance. At the time of the UK5099 intervention, pyruvate was reduced by approximately 15% in the Aβ_25–35_ group compared with the control group (Figure 1L), while the Aβ_25–35_ + UK5099 group showed a greater reduction of 35% compared with the UK5099 group (Figure 1L). Our study supported the function of Aβ_25–35_ injury on neuronal glycolysis, whose dysfunction is involved in AD.

### Expression of hnRNP A1 decreases in AD models

**FIGURE 2:**
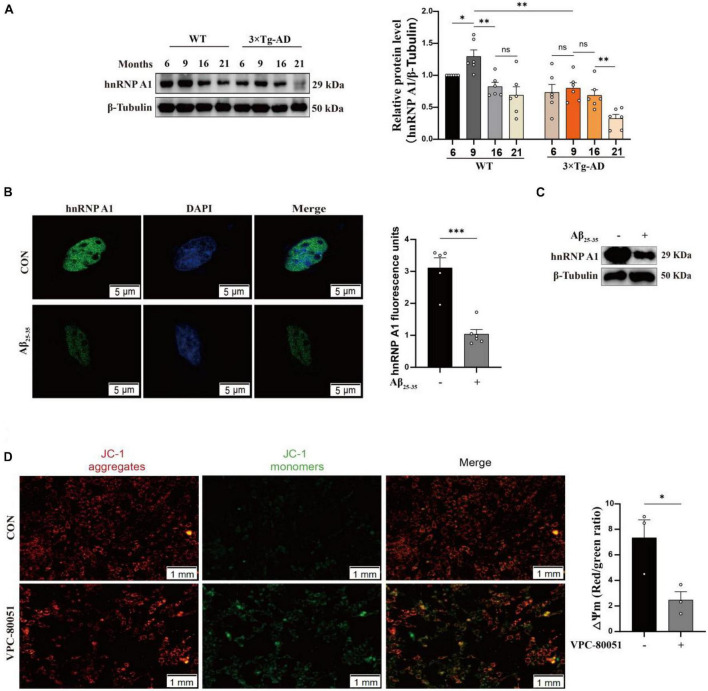
Expression of hnRNP A1 decreases in AD models. **(A)** Changes in hnRNP A1 in brain tissue of WT and 3 × Tg-AD mice by Western blotting (*n* = 6 mice per group). **(B)** Laser confocal observation: fluorescence intensity and cellular localization of hnRNP A1 in Aβ_25–35_-induced HT22 cells (*n* > 4 per group). **(C)** Western blotting analysis: effect of Aβ_25–35_ on hnRNP A1 protein expression in mouse primary hippocampal neurons. **(D)** Observe the changes in mitochondrial membrane potential after using VPC-80051 inhibitor in HT22 cells: red fluorescence represents higher membrane potential levels and green fluorescence represents lower membrane potential levels (*n* = 3 per group). Statistical analysis was performed using GraphPad software Prism 8.0. All data were expressed as mean ± standard error (mean ± SEM). Student’s *t*-test and one-way analysis of variance (ANOVA) were used to determine the levels of statistical differences between two and multiple groups, respectively. Statistically significant differences were set at *p < 0.05, **p < 0.01, ***p < 0.001.

Using MWM to test learning and memory abilities is dependable (Tucker et al., 2018). It showed that 9-month-old 3 × Tg-AD mice exhibited prolonged escape latency on the fourth day (Supplementary Figure 1B) and impaired spatial perception (Supplementary Figure 1C), which is in agreement with earlier study (Jiang et al., 2015). Subsequently, we confirmed this with different behavioral tests. Turning bar experiment showed that 9-month-old 3 × Tg-AD mice with poor coordination could fall off the batting in a short period of time (Supplementary Figure 1D). The open-field experiment suggested that autonomous and exploratory behaviors were diminished in 9-month-old 3 × Tg-AD mice (Supplementary Figure 1E). Nesting experiments showed reduced daily living skills in 9-month-old 3 × Tg-AD mice (Supplementary Figure 1F). These lines of evidence suggest that 9-month-old 3 × Tg-AD mice already exhibit cognitive impairments that affect motor behavior. The hippocampus is a site of memory storage and spatial orientation (Zhang et al., 2022). Next, we observed structural changes and neuronal survival in DG and CA3 regions of hippocampal tissue by tissue sectioning. HE staining showed that the structure of neurons was obviously intact and densely arranged in WT mice, whereas neurons appeared with obvious pyknosis and loose arrangement in 3 × Tg-AD mice (Supplementary Figure 2A). Nissl staining suggested that neuron morphological structure destruction and the number of surviving neurons were reduced in 3 × Tg-AD mice (Supplementary Figures 2B–D). Anti-hnRNP A1 antibodies are known to cause mislocalization and increased nuclear depletion of hnRNP A1 in neurons and induce neuronal death (Libner et al., 2022). One study also reported that hnRNP A1 regulates glycolytic function by selective cutting of PK RNA (David et al., 2010). We hypothesized that hnRNP A1 may be an important molecule that links AD neuronal death and glycolytic dysfunction. Therefore, we assessed the hnRNP A1 expression in brain tissue from 6-, 9-, 16-, and 21-month-old AD and WT mice to investigate whether there is an association between AD and hnRNP A1. Western blotting revealed that hnRNP A1 in mice brain tissue gradually decreased from 9 months of age. Meanwhile, hnRNP A1 expression in the brain tissue of 3 × Tg-AD mice was consistently lower than that of WT mice at the same month of age (Figure 2A). This result supported that 9-month-old 3 × Tg-AD mice exhibited a variety of behavioral abnormalities and reduced hnRNP A1 expression compared to control mice. Furthermore, we hypothesized that Aβ_25–35_ induced neuronal death by regulating the down-regulation of hnRNP A1 As expected, immunofluorescence showed that Aβ_25–35_ resulted in a downregulation of the mean fluorescence intensity of hnRNP A1 (Figure 2B). Western blotting revealed that Aβ_25–35_ induced a downregulation of hnRNP A1 expression in primary mice hippocampal neurons (Figure 2C). We used VPC-80051 (Carabet et al., 2019) to inhibit the RNA binding domain (RBD) of hnRNP A1 and detected changes in the mitochondrial membrane potential. JC1 emits red or green fluorescence to represent high or low mitochondrial membrane potential, respectively. At the same time, according to the effect of VPC-80051 on the viability of HT22 cells, we decided to use 50 μM for the experiment (Supplementary Figure 3A). We found that mitochondrial membrane potential in VPC-80051 group showed a significant decrease in HT22 cells (Figure 2D), which is an early sign of apoptosis (Jin et al., 2019). These results suggested that neuronal death is closely associated with the reduced hnRNP A1 expression in AD models. The reduced hnRNP A1 expression is involved in AD pathogenesis, and hnRNP A1 may be a predisposing factor for age-related neurodegeneration.

### hnRNP A1 directly binds to HK1 mRNA

**FIGURE 3:**
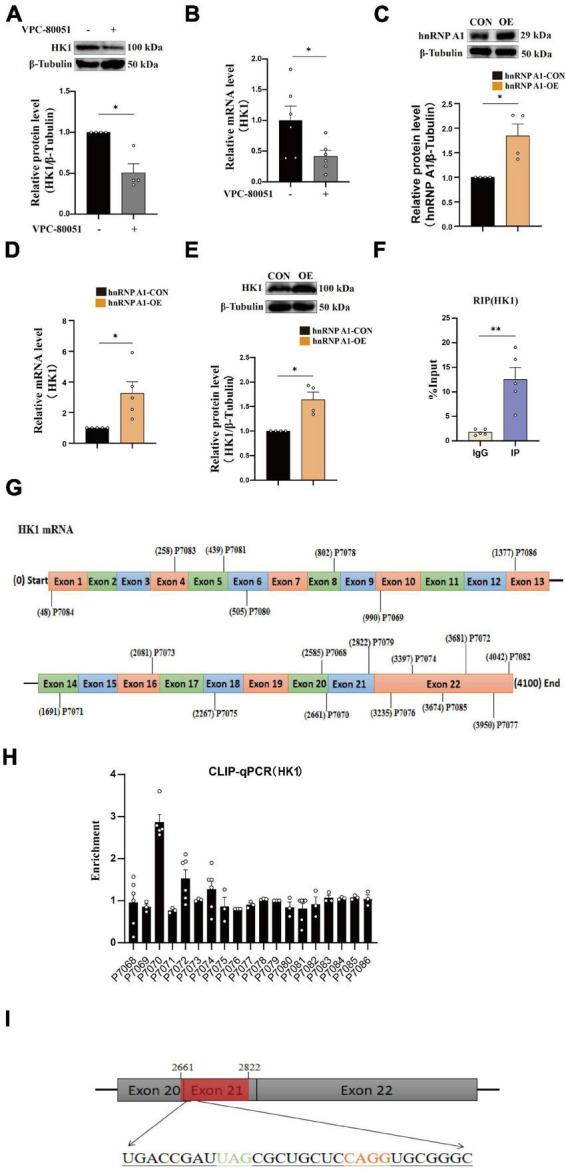
hnRNP A1 regulates HK1 RNA. **(A)** Effects on HK1 expression after VPC-80051 in HT22 as observed by Western blotting (*n* = 4 per group). **(B)** RT-qPCR analysis: the effect VPC-80051 on the HK1 mRNA expression of HT22 (*n* = 4 per group). **(C)** Observation of overexpression of hnRNP A1 protein in HT22 (*n* = 4 per group). **(D)** RT-qPCR analysis: the effect of hnRNP A1 overexpression on the HK1 mRNA expression of HT22 (*n* = 4 per group). **(E)** Effect on HK1 expression after overexpression of hnRNP A1 as observed by Western blotting (*n* = 4 per group). **(F)** Enrichment of HK1 mRNA by qPCR after pulling down RNA using hnRNP A1 antibody (*n* = 8 per group). **(G)** Schematic diagram of primer design for HK1 mRNA base sequence. **(H)** CLIP-qPCR analysis: binding region of HK1 mRNA bound to hnRNP A1 was inferred by enrichment efficiency (*n* = 3 per group). **(I)** Schematic diagram: Possible binding regions of HK1 mRNA and hnRNP A1 inferred from the enrichment efficiency of CLIP-qPCR. Statistical analysis was performed using GraphPad software Prism 8.0. All data were expressed as mean ± standard error (mean ± SEM). Student’s *t*-test and one-way analysis of variance (ANOVA) were used to determine the levels of statistical differences between two and multiple groups, respectively. Statistically significant differences were set at *p < 0.05, **p < 0.01.

It has been reported that hnRNP A1 regulates glycolytic function by selective cutting of PK RNA (David et al., 2010). We also confirmed the cooperative relationship between hnRNP A1 and PKM through RIP experiments (Supplementary Figure 3B). We speculated that the decreased HK1 might be related to the lower regulation of hnRNP A1. To test this conjecture, we used HT22 cells as cell model *in vitro* to elucidate the mechanism of hnRNP A1 in AD. Then, we cultured HT22 cells with VPC-80051 to inhibit the RNA binding domain (RBD) of hnRNP A1 for 24 h and found a significant down-regulation of HK1 protein (Figure 3A) and HK1 mRNA expression (Figure 3B). Next, we constructed a stable overexpression cell line of hnRNP A1 (Figure 3C) in cultured HT22 cells. We found that the mRNA (Figure 3D) and protein expression (Figure 3E) levels of HK1 were significantly increased after hnRNP A1 overexpression by western blotting and RT-qPCR. To further verify the existence of their partnership, we used hnRNP A1 antibody to pull down all RNAs bound to hnRNP A1. By RT-qPCR, we found a significant enrichment of HK1 compared to the IgG group (Figure 3F). Finally, to find the base sequence of HK1 mRNA binding to hnRNP A1, we designed 19 primers targeting HK1 mRNA sequences (Figure 3G). The CLIP-qPCR revealed a clear enrichment at primer number P7070, which was analyzed to act on the 2605-2821 region of the HK1 mRNA (Figure 3H). The binding site may be related to the best sequence 5′-UAG-3′ (Jain et al., 2017) in the sequence as determined by analysis of the affinity distribution, or to the sequence 5′-UAG-3′ (Bartys et al., 2022) that can be used as an efficient binding platform for hnRNP A1 by binding screening assays, Kd assays and UV melting experiments (Figure 3I). In conclusion, the above results supported that hnRNP A1 binds directly to HK1 mRNA to regulate the protein expression of HK1 and provided a possible base sequence for binding to HK1 mRNA.

### Enhancing of the hnRNP A1 ameliorated glycolytic impairment and neuronal death in Aβ_25–35_-induced HT22 cells

**FIGURE 4:**
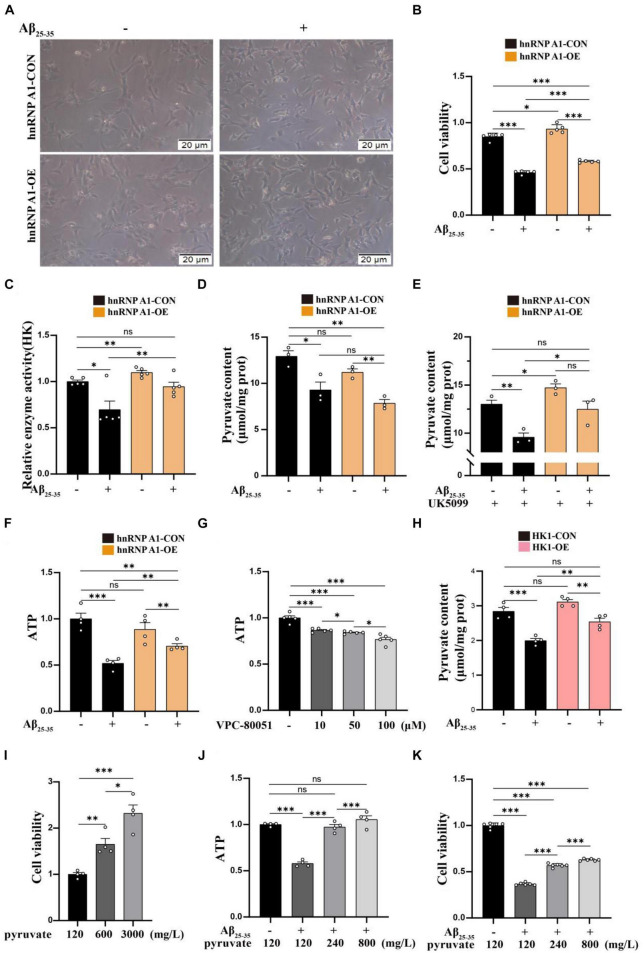
Enhancing of the hnRNP A1 ameliorated Aβ_25–35_-induced glycolytic impairment and neuronal death in HT22 cells. **(A)** Protective effect of hnRNP A1 overexpression on Aβ_25–35_-induced HT22 as observed by passaged microscopy. **(B)** Observation of the effect of hnRNP A1 overexpression on the viability of Aβ_25–35_-induced HT22 cells (*n* = 5 per group). **(C)** Observation of the effect of hnRNP A1 overexpression on HK enzyme activity of Aβ_25–35_-induced HT22 cells (*n* = 5 per group). **(D,E)** Observation of the effect of hnRNP A1 overexpression on Pyruvate content of Aβ_25–35_-induced HT22 cells (*n* = 3 per group). **(F)** Observation of the effect of hnRNP A1 overexpression on ATP of Aβ_25–35_-induced HT22 cells (*n* = 4 per group). **(G)** Observation of the effect of different concentrations of VPC-80051 on ATP in HT22 cells (*n* = 5 per group). **(H)** Observation of the effect of HK1 overexpression on Pyruvate content of Aβ_25–35_-induced HT22 cells (*n* = 4 per group). **(I)** Observation of the effect of different concentrations of pyruvate on the viability of HT22 cells (*n* = 4 per group). **(J)** Effect of increasing pyruvate concentration on Aβ_25–35_-induced ATP deficiency in HT22 (*n* = 5 per group). **(K)** Effect of increasing pyruvate concentration on the cell viability of Aβ_25–35_-induced HT22 (*n* = 4 per group). Statistical analysis was performed using GraphPad software Prism 8.0. All data were expressed as mean ± standard error (mean ± SEM). Student’s *t*-test and one-way analysis of variance (ANOVA) were used to determine the levels of statistical differences between two and multiple groups, respectively. Statistically significant differences were set at *p < 0.05, **p < 0.01, ***p < 0.001.

To investigate the effect of hnRNP A1 on the glycolytic function of hippocampal neurons in mice. First, we found that the hnRNP A1 overexpression could ameliorate Aβ_25–35_-induced cell axonal damage (Figure 4A) and cell death (Figure 4B) in HT22 cells. Mechanistically, hnRNP A1 overexpression caused elevation of pyruvate (Figure 4E) and lactate (Supplementary Figure 3C) from glycolysis by improving protein expression and enzymatic activity of HK1 (Figure 4C) in Aβ_25–35_-induced HT22 cells. Here, a point to note was that the pyruvate content of hnRNP A1 overexpression did not change when not given the UK5099 intervention compared to the normal group, which may be related to increased mitochondrial uptake of pyruvate by hnRNP A1 overexpression (Figure 4D). We then demonstrated that hnRNP A1 overexpression ameliorated energy impairment in Aβ_25–35_-induced HT22 cells by assaying the ATP content (Figure 4F). Reverse validation by VPC-80051 showed that inhibition of hnRNP A1 by RBD can cause a decrease in neuronal ATP (Figure 4G) and death (Supplementary Figure 3A). Secondly, to further confirm that hnRNP A1 overexpression is mitigating HT22 damage by improving HK1 expression. We also constructed a stable overexpression cell line of HK1 in cultured HT22 cells. As expected, HK1 overexpression also improved the Aβ_25–35_-induced reduction of pyruvate (Figure 4H) in HT22. Finally, to show that overexpression of hnRNP A1 ultimately ameliorated neuronal death by promoting pyruvate production. We used sodium pyruvate as the sole carbon source for neurons in culture, and the results showed that increasing the amount of sodium pyruvate under normal conditions significantly increased neuronal survival (Figure 4I). Similarly, increased levels of sodium pyruvate improved Aβ_25–35_-induced impairment of energy metabolism (Figure 4J) and neuronal death (Figure 4K) in HT22. In conclusion, Aβ in neurons caused glycolytic damage by downregulating hnRNP A1 expression. Impaired energy metabolism and neuronal death could be reversed by enhancing hnRNP A1. At the same time, we found that the protection of hnRNP A1 on neurons was multifaceted, which may be related to the improvement of glycolysis function (Supplementary Figures 3D–G). We described this in more detail and expanded discussion on it in the later discussion section.

### A bidirectional regulatory mechanism between hnRNP A1 and Aβ

**FIGURE 5:**
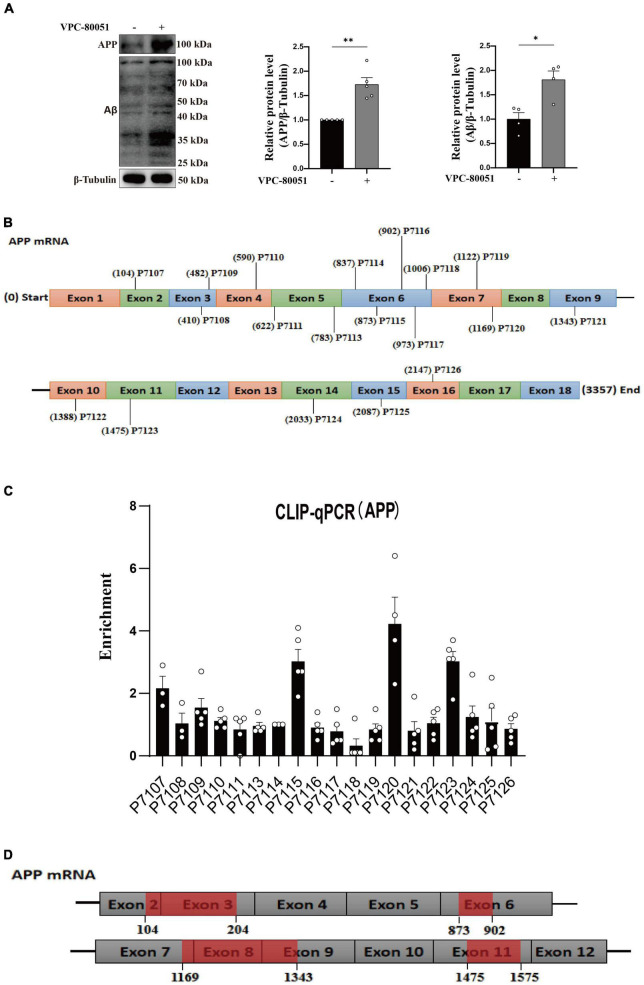
hnRNP A1 regulates Aβ through APP RNA. **(A)** Western blotting analysis: effect of VPC-80051 on APP and Aβ expression of HT22 (*n* = 4 per group). **(B)** Schematic diagram of primer design for APP mRNA base sequence. **(C)** CLIP-qPCR analysis: Binding region of APP mRNA bound to hnRNP A1 was inferred by enrichment efficiency (*n* = 3 per group). **(D)** Schematic diagram: possible binding regions of APP mRNA and hnRNP A1 inferred from the enrichment efficiency of CLIP-qPCR. Statistical analysis was performed using GraphPad software Prism 8.0. All data were expressed as mean ± standard error (mean ± SEM). Student’s *t*-test and one-way analysis of variance (ANOVA) were used to determine the levels of statistical differences between two and multiple groups, respectively. Statistically significant differences were set at *p < 0.05, **p < 0.01.

**FIGURE 6:**
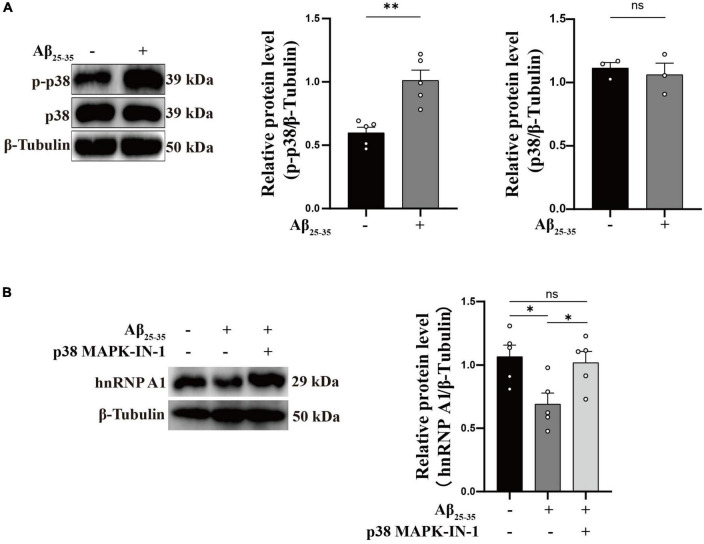
Aβ regulates hnRNP A1 through p-p38 MAPK. **(A)** Effect of Aβ_25–35_ on p38 MAPK and p-p38 MAPK expression in HT22 cells as observed by Western blotting (*n* ≥ 3 per group). **(B)** Western blotting: effect of Aβ_25–35_ and p38 MAPK-IN-1 on hnRNP A1 protein expression in HT22. Statistical analysis was performed using GraphPad software Prism 8.0. All data were expressed as mean ± standard error (mean ± SEM). Student’s *t*-test and one-way analysis of variance (ANOVA) were used to determine the levels of statistical differences between two and multiple groups, respectively. Statistically significant differences were set at *p < 0.05, **p < 0.01.

As is well known, damage to neurons exposed to Aβ is considered to be a major pathological feature of AD (Yan et al., 2023). It has been reported that hnRNP A1 is involved in alternative splicing of amyloid precursor protein (APP) RNA in neuronal cells differentiated from malignant pluripotent embryonal carcinoma, and increasing hnRNP A1 reduces the formation of Aβ (Donev et al., 2007). At the same time, immunofluorescence revealed that Aβ was widely distributed in DG and CA3 regions of 9-month-old 3 × Tg-AD mice, whereas no significant deposition was detected in WT mice at the same month of age (Supplementary Figures 2E–H), which is consistent with previously reported clinical observations (Colom-Cadena et al., 2020). To verify whether the mechanism as described above exists between Aβ deposition and hnRNP A1 in mice hippocampal neurons, follow-up experiments were performed using HT22 cells. As expected, the expression of APP, Aβ oligomers were significantly elevated after inhibition of hnRNP A1 using VPC-80051 (Figure 5A). Next, we further designed different primers for CLIP-qPCR experiments based on APP mRNA (Figure 5B). The results showed the presence of four main binding regions with significant enrichment, namely base sequences 104-204, 873-902, 1169-1343, and 1475-1575 (Figures 5C, D). Exon 7 have the similarity as previously reported, but the binding sites of exon 2, exon 6 and exon 11 were not reported. After searching all transcripts known to APP, no transcripts were found that did not include exons 2, 6 and 11. These results suggest that hnRNP A1 binds directly to APP mRNA to regulate Aβ production. Furthermore, recent studies have reported that Aβ-induced phosphorylation of p38 MAPK increases ROS, leading to neuronal cell death (Lee and Kim, 2017). In fibroblasts hnRNP A1 expression levels are regulated in a p38 MAPK-dependent manner, probably through its phosphorylation (Shimada et al., 2009). Therefore, we hypothesized that Aβ_25–35_ induced downregulation of hnRNP A1 probably through activation of p-p38 MAPK. As expected, Western blotting results showed that Aβ_25–35_ significantly increased the expression of p-p38 MAPK (Figure 6A), but not the expression of p38 MAPK (Figure 6A). To test this conjecture, the p38 MAPK activity inhibitor p38 MAPK-IN-1was used, and the results showed that p38 MAPK-IN-1 significantly inhibited Aβ_25–35_-induced downregulation of hnRNP A1 (Figure 6B). Our data suggested that Aβ_25–35_-induced downregulation of hnRNP A1 may be achieved through activation of p38 MAPK phosphorylation. Taken together, the evidence suggested a bidirectional regulatory mechanism between hnRNP A1 and Aβ, which may further induce hnRNP A1 downregulation and ultimately exacerbate neuronal glycolytic dysfunction.

## Discussion

hnRNP A1 is abundantly expressed in nuclear proteins and belongs to the hnRNP subfamily. As an RNA binding protein, it plays an important role in mRNA stabilization, miRNA processing, mRNA translation, variable splicing, and transcriptional regulation. Abnormal RNA metabolism may have serious consequences for a variety of diseases due to abnormal regulation of RNA-binding proteins (Feng et al., 2022). In fibroblasts (Shimada et al., 2009) and hepatocellular carcinoma (Zhao et al., 2022), downregulation of hnRNP A1 accelerates cellular senescence, and hnRNP A1 expression gradually decreases to undetectable levels during fibroblast senescence (Wang et al., 2016). Aging is the most common risk factor in AD (Li et al., 2021). In the present study, we found that the expression of hnRNP A1 in the brain tissue of 3 × Tg-AD mice was highest at 9 months of age and decreased gradually thereafter, and its expression in 3 × Tg-AD mice was significantly lower than that in WT mice at the same month of age, which suggest that the decreased hnRNP A1 is involved in AD pathogenesis, and hnRNP A1 may be a susceptibility factor for AD. It is worth mentioning that the relationship between hnRNP A1 expression and age in this study may be related to the growth and development of the mice themselves, but more possible mechanisms need to be further investigated in depth. It is well known that brain glycolytic dysfunction is involved in the pathogenesis of AD, however, the relationship between glycolytic imbalance and AD pathogenesis has not been fully elucidated. The severity of tau pathology and amyloid correlates with reduced glycolytic flux in patients with preclinical and clinical AD (Li et al., 2021). Our study went one step further and we discovered direct binding of hnRNP A1 to the HK1 mRNA by RIP experiments. Consistently, we obtained the same conclusion by inhibiting and overexpressing hnRNP A1, and found that hnRNP A1 may function by binding to the HK1 mRNA sequence at positions 2661-2821 by CLIP-qPCR technology. Our present study suggests that reduced expression of hnRNP A1 is involved in the pathogenesis of AD by transcriptional regulation of HK, which links glycolytic dysfunction to dysregulated hnRNP A1 expression in AD.

Recent evidence reveals that extraneuronal Aβ is mainly derived from the pathology of “PANTHOS” generated by defective autophagy in neurons themselves (Lee et al., 2022). Therefore, after we showed that hnRNP A1 regulates HK1, we sought to link Aβ deposition in neurons to glycolysis via hnRNP A1. We have reached the similar conclusion as our predecessors in hippocampal neurons that hnRNP A1 affects Aβ through the selective shearing of APP mRNA (Donev et al., 2007). However, we also found binding sites in exons 2, 6 and 11 of APP mRNA, and the specific mechanism of action needs to be further investigated. Compared with WT mice of the same age, the hnRNP A1 expression in 3 × Tg-AD mice brain decreased significantly, which may be related to APP transgenic or Aβ sedimentation. Our study showed that Aβ_25–35_ induces a concomitant downregulation of hnRNP A1 expression in primary mice hippocampal neurons (Supplementary Figure 2B) and HT22 cells that may be associated with activation of p38 MAPK phosphorylation. This suggests a bidirectional regulation between hnRNP A1 and Aβ, promoting further loss of hnRNP A1 and further exacerbating glycolytic dysfunction. It is well established that pyruvate, an end product of glycolysis, is decreased in the brain of AD clinical patients and AD mice. Pyruvate treatment significantly reduced cognitive impairment, brain energy reserve (Koivisto et al., 2016) and neuronal death (Wang et al., 2015) in a mice model of AD. We found that decreased hnRNP A1 aggravated glycolytic dysfunction and neuronal death in hippocampal neurons of AD mice. In addition, our study showed that impairment of energy metabolism can be ameliorated by enhancing hnRNP A1 expression, thereby alleviating hippocampal neuronal cell death, which provides a new concept for potential AD treatment strategies.

Notably, hnRNP A1 also selectively shears into PKM1 and PKM2 by binding to the pyruvate kinase precursor mRNA in glycolysis (David et al., 2010). We also confirmed the binding of hnRNP A1 to PKM mRNA by RIP assay in HT22 cells (Supplementary Figure 3B). Meanwhile, the ratio of PKM2 to PKM1 determines the switch between neuronal aerobic glycolysis and oxidative phosphorylation (Zheng et al., 2016). We found that hnRNP A1 could increase pyruvate by regulating HK1 and also increase lactate (Supplementary Figure 2C). This could be related to an increase in overall glycolytic flux, or more toward aerobic glycolysis. This may explain the phenomenon that hnRNP A1 confers a metabolic advantage to tumors, not only by shearing PKM, but also by increasing HK1 expression to promote overall glycolytic flux. However, there is no conflict with our present study. Undoubtedly, activation of hnRNP A1 can ameliorate impaired energy metabolism and neuronal death. At the same time, we found that hnRNP A1 overexpression may act through the glycolytic pathway to improve ROS. Oxidative stress is the bridge between the various mechanisms of AD. Oxidative stress plays a critical role in AD and may even be considered as a key central factor in AD pathogenesis (Bai et al., 2022). hnRNP A1 overexpression group reversed the Aβ_25–35_-induced HT22 glycolytic flux, leading to an increase in the glycolytic derivative glutathione (GSH) (Supplementary Figure 3D). GSH acts as an essential free radical scavenger, which may be responsible for reversing elevated ROS (Supplementary Figure 3E). To further confirm this link, the glycolysis inhibitor 2-DG reduced the elevation of GSH in the hnRNP A1 overexpression group (Supplementary Figure 3F), while elevated ROS in the overexpression group (Supplementary Figure 3G). These results suggested that the hnRNP A1 overexpression group ameliorates Aβ_25–35_-induced ROS in HT22, possibly by increasing GSH. Although these results were correlational studies, they were sufficient to suggest that the protection of hnRNP A1 on neurons is multifaceted and has important research value.

This study has limitations. First, CLIP-qPCR is designed based on mRNA primers and does not involve intron detection. Second, this study focused on exploring the molecular mechanisms of AD and did not address the individual differences between male and female in AD. Third, although it is uncertain whether exogenous Aβ can accurately reflect the pathophysiology of AD, it seems to be optimal for molecular interaction studies.

In conclusion, our study suggested that Aβ is a causative factor, perhaps not the originator, in promoting the development of AD disease course. We provided a more direct regulatory mechanism for the relationship between impaired glycolysis and AD.

This study also lays a foundation for the further study of hnRNP A1 as a potential target for the prevention and treatment of AD.

## Data availability statement

The original contributions presented in this study are included in the article/Supplementary material, further inquiries can be directed to the corresponding authors.

## Ethics statement

The animal study was approved by the Laboratory Animal Welfare and Ethics Committee (Zunyi Medical University). The study was conducted in accordance with the local legislation and institutional requirements.

## Author contributions

X-HJ: experiment implementation, data analysis, and manuscript draft. A-HW and T-TL: experimental assistance and data analysis. YC, YZ, HJ, and FJ: 18F-FDG MicroPET. H-PL, L-NW, JL, YZ, FY, and M-XC: experimental assistance. HJ and J-SS: research design and supervision. S-YZ and FJ: research design, manuscript revision, supervision, and funding application. IEMed Guangzhou Biomedical Technology Co., Ltd: CLIP-qPCR technical support. All authors contributed to the article and approved the submitted version.

## Supplementary material

Supplementary Figure 1 Behavioral abnormalities in 9-month-old 3 × Tg-AD mice. **(A)** Schematic diagram of the different age stages of animals and experimental procedures. **(B,C)** Spatial cognitive ability and escape latency in 9-month-old WT and 3 × Tg-AD mice (*n* > 6 mice per group). **(D)** Evaluation of motor function in mice: statistical graphs of persistence time in turning the stick (*n* = 6 mice per group). **(E)** Evaluating the spatial exploration ability of mice: a statistical chart of the distance mice move (*n* = 6 mice per group). **(F)** Nesting ability in 9-month-old WT and 3 × Tg-AD mice (*n* = 4 mice per group).

Supplementary Figure 2 Immunohistochemistry of hippocampal tissues from AD mice. **(A)** Histological changes in the hippocampus of 9-month-old WT and 3 × Tg-AD mice were analyzed by HE staining. **(B–D)** Analysis of neuronal status in the hippocampus of 9-month-old WT and 3 × Tg-AD mice by Nissler staining (*n* = 3 mice per group). **(E–H)** Analysis and quantification of Aβ deposition in hippocampal tissue of 9-month-old WT and 3 × Tg-AD mice by immunofluorescence (*n* = 4 mice per group).

Supplementary Figure 3 Prospects: other regulatory roles of hnRNP A1. **(A)** Observation of the effect of different concentrations of VPC-80051 on viability in HT22 cells (*n* = 6 per group). **(B)** Enrichment of PKM1 and PKM2 mRNA by qPCR after pulling down RNA using hnRNP A1 antibody (*n* = 6 per group). **(C)** Observation of the effect of hnRNP A1 overexpression on lactate content of Aβ_25–35_-induced HT22 cells (*n* = 3 per group). **(D)** Observation of the effect of hnRNP A1 overexpression on GSH of Aβ_25–35_-induced HT22 cells (*n* = 3 per group). **(E)** Observation of the effect of hnRNP A1 overexpression on ROS of Aβ_25–35_-induced HT22 cells (*n* = 3 per group). **(F)** To observe the effect of hnRNP A1 overexpression on GSH after the use of glycolysis inhibitor 2-DG (*n* = 3 per group). **(G)** To observe the effect of hnRNP A1 overexpression on ROS after the use of glycolysis inhibitor 2-DG (*n* = 3 per group).
