# Supplementary figures and images for: Suppression of hnRNP A1 binding to HK1 RNA leads to glycolytic dysfunction in Alzheimer’s disease models (part 1 of 4)
Source: Front Aging Neurosci. 2023 Aug 31;15:1218267. doi: 10.3389/fnagi.2023.1218267 (PMC10516183; doi:10.3389/fnagi.2023.1218267)

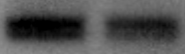

Supplement: Supplementary Figure 1 — Behavioral abnormalities in 9-month-old 3 × Tg-AD mice. (A) Schematic diagram of the different age stages of animals and experimental procedures. (B,C) Spatial cognitive ability and escape latency in 9-month-old WT and 3 × Tg-AD mice (n > 6 mice per group). (D) Evaluation of motor function in mice: statistical graphs of persistence time in turning the stick (n = 6 mice per group). (E) Evaluating the spatial exploration ability of mice: a statistical chart of the distance mice move (n = 6 mice per group). (F) Nesting ability in 9-month-old WT and 3 × Tg-AD mice (n = 4 mice per group). [file Data_Sheet_2.zip › FIG1/HT22 HK/2022-11-03 jxh 1'1HK.png]

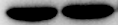

Supplement: Supplementary Figure 1 — Behavioral abnormalities in 9-month-old 3 × Tg-AD mice. (A) Schematic diagram of the different age stages of animals and experimental procedures. (B,C) Spatial cognitive ability and escape latency in 9-month-old WT and 3 × Tg-AD mice (n > 6 mice per group). (D) Evaluation of motor function in mice: statistical graphs of persistence time in turning the stick (n = 6 mice per group). (E) Evaluating the spatial exploration ability of mice: a statistical chart of the distance mice move (n = 6 mice per group). (F) Nesting ability in 9-month-old WT and 3 × Tg-AD mice (n = 4 mice per group). [file Data_Sheet_2.zip › FIG1/HT22 HK/2022-11-03 jxh 1'1tub.png]

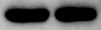

Supplement: Supplementary Figure 1 — Behavioral abnormalities in 9-month-old 3 × Tg-AD mice. (A) Schematic diagram of the different age stages of animals and experimental procedures. (B,C) Spatial cognitive ability and escape latency in 9-month-old WT and 3 × Tg-AD mice (n > 6 mice per group). (D) Evaluation of motor function in mice: statistical graphs of persistence time in turning the stick (n = 6 mice per group). (E) Evaluating the spatial exploration ability of mice: a statistical chart of the distance mice move (n = 6 mice per group). (F) Nesting ability in 9-month-old WT and 3 × Tg-AD mice (n = 4 mice per group). [file Data_Sheet_2.zip › FIG1/HT22 HK/2022-11-03 jxh 2'1tub.png]

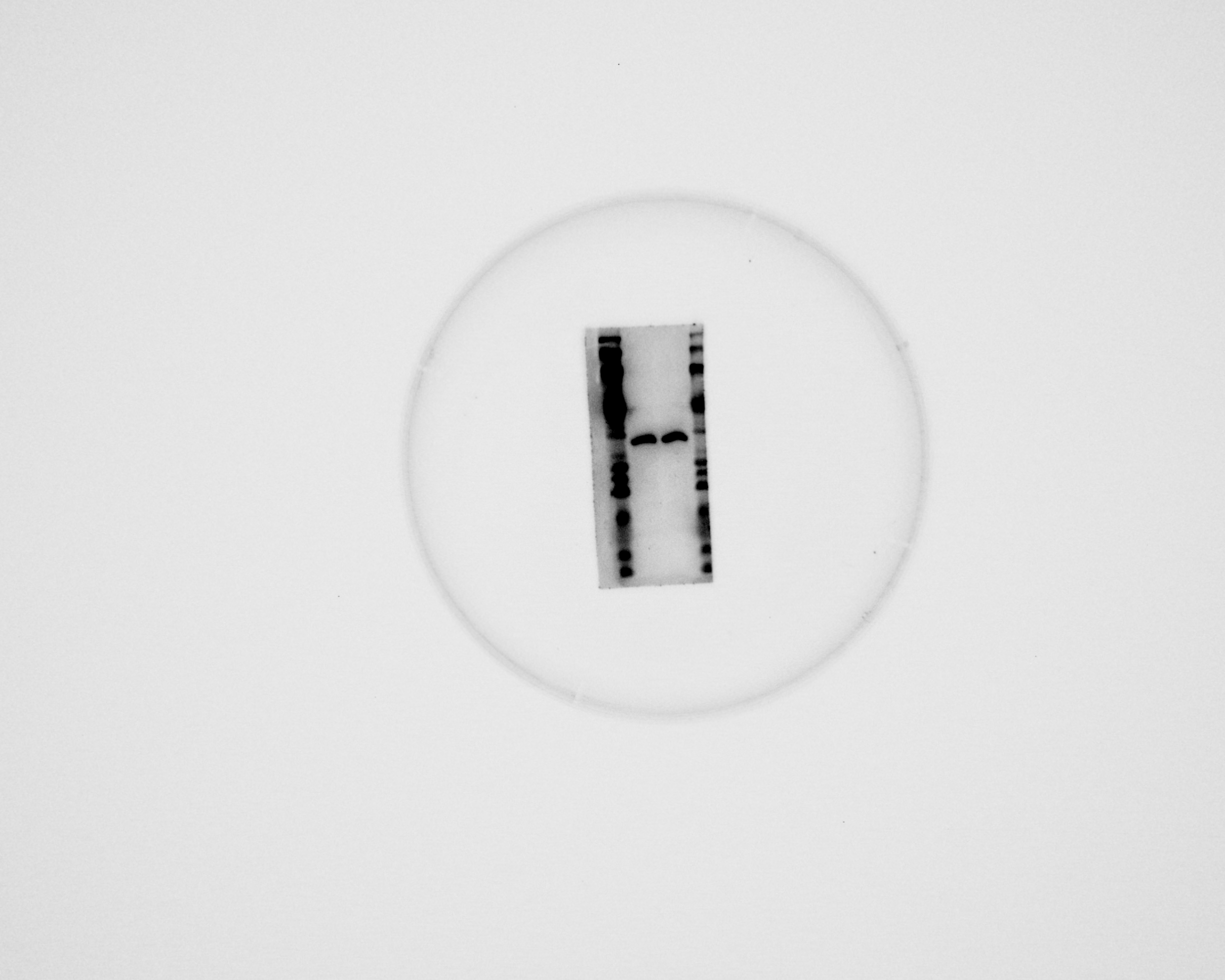

Supplement: Supplementary Figure 1 — Behavioral abnormalities in 9-month-old 3 × Tg-AD mice. (A) Schematic diagram of the different age stages of animals and experimental procedures. (B,C) Spatial cognitive ability and escape latency in 9-month-old WT and 3 × Tg-AD mice (n > 6 mice per group). (D) Evaluation of motor function in mice: statistical graphs of persistence time in turning the stick (n = 6 mice per group). (E) Evaluating the spatial exploration ability of mice: a statistical chart of the distance mice move (n = 6 mice per group). (F) Nesting ability in 9-month-old WT and 3 × Tg-AD mice (n = 4 mice per group). [file Data_Sheet_2.zip › FIG1/HT22 HK/original data/2022-11-03 4'1tub.tif]

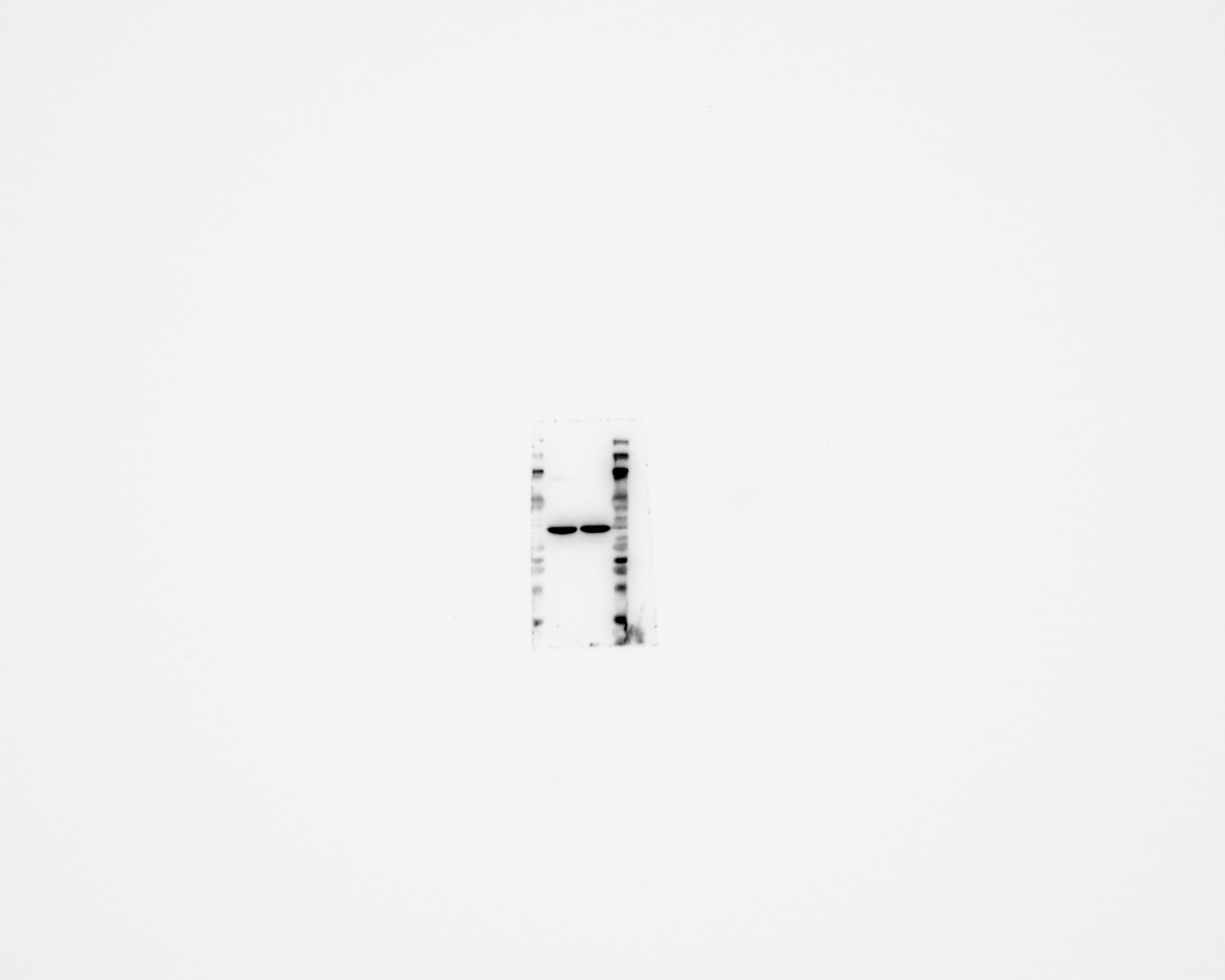

Supplement: Supplementary Figure 1 — Behavioral abnormalities in 9-month-old 3 × Tg-AD mice. (A) Schematic diagram of the different age stages of animals and experimental procedures. (B,C) Spatial cognitive ability and escape latency in 9-month-old WT and 3 × Tg-AD mice (n > 6 mice per group). (D) Evaluation of motor function in mice: statistical graphs of persistence time in turning the stick (n = 6 mice per group). (E) Evaluating the spatial exploration ability of mice: a statistical chart of the distance mice move (n = 6 mice per group). (F) Nesting ability in 9-month-old WT and 3 × Tg-AD mice (n = 4 mice per group). [file Data_Sheet_2.zip › FIG1/HT22 HK/original data/2022-11-03 1'1tub.tif]

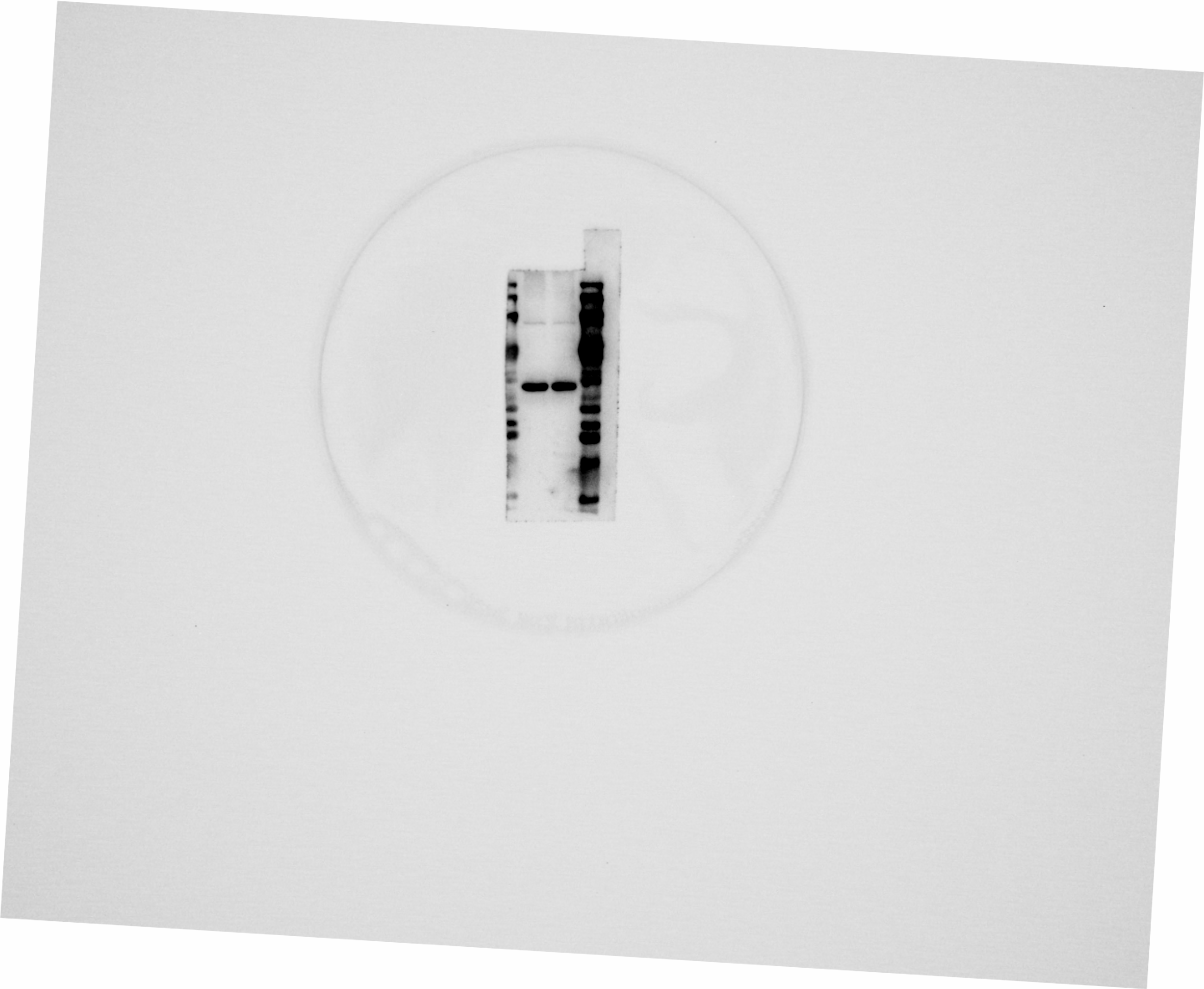

Supplement: Supplementary Figure 1 — Behavioral abnormalities in 9-month-old 3 × Tg-AD mice. (A) Schematic diagram of the different age stages of animals and experimental procedures. (B,C) Spatial cognitive ability and escape latency in 9-month-old WT and 3 × Tg-AD mice (n > 6 mice per group). (D) Evaluation of motor function in mice: statistical graphs of persistence time in turning the stick (n = 6 mice per group). (E) Evaluating the spatial exploration ability of mice: a statistical chart of the distance mice move (n = 6 mice per group). (F) Nesting ability in 9-month-old WT and 3 × Tg-AD mice (n = 4 mice per group). [file Data_Sheet_2.zip › FIG1/HT22 HK/original data/2022-11-03 2'1tub.tif]

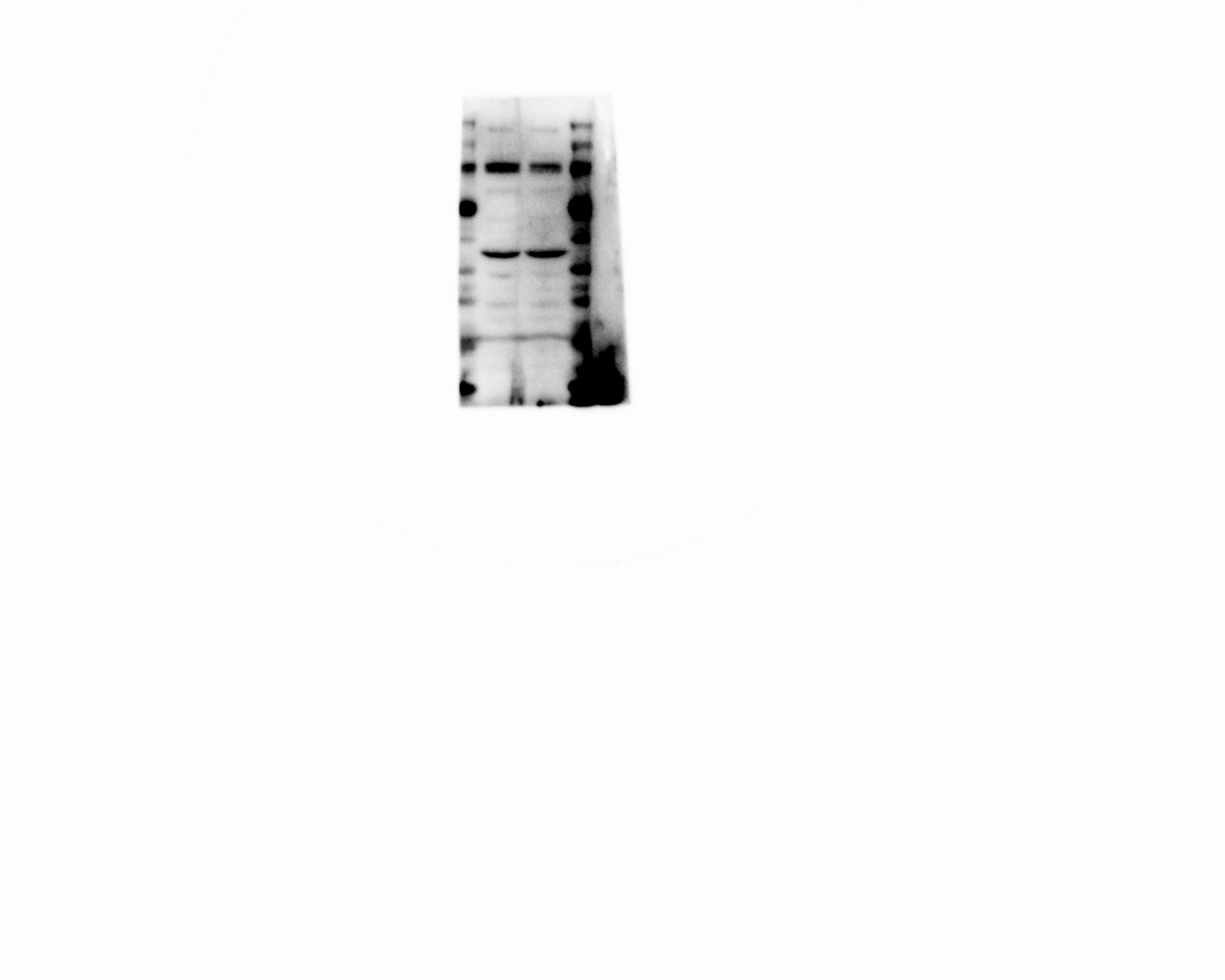

Supplement: Supplementary Figure 1 — Behavioral abnormalities in 9-month-old 3 × Tg-AD mice. (A) Schematic diagram of the different age stages of animals and experimental procedures. (B,C) Spatial cognitive ability and escape latency in 9-month-old WT and 3 × Tg-AD mice (n > 6 mice per group). (D) Evaluation of motor function in mice: statistical graphs of persistence time in turning the stick (n = 6 mice per group). (E) Evaluating the spatial exploration ability of mice: a statistical chart of the distance mice move (n = 6 mice per group). (F) Nesting ability in 9-month-old WT and 3 × Tg-AD mice (n = 4 mice per group). [file Data_Sheet_2.zip › FIG1/HT22 HK/original data/wb 2022-11-01 1'1 hk.tif]

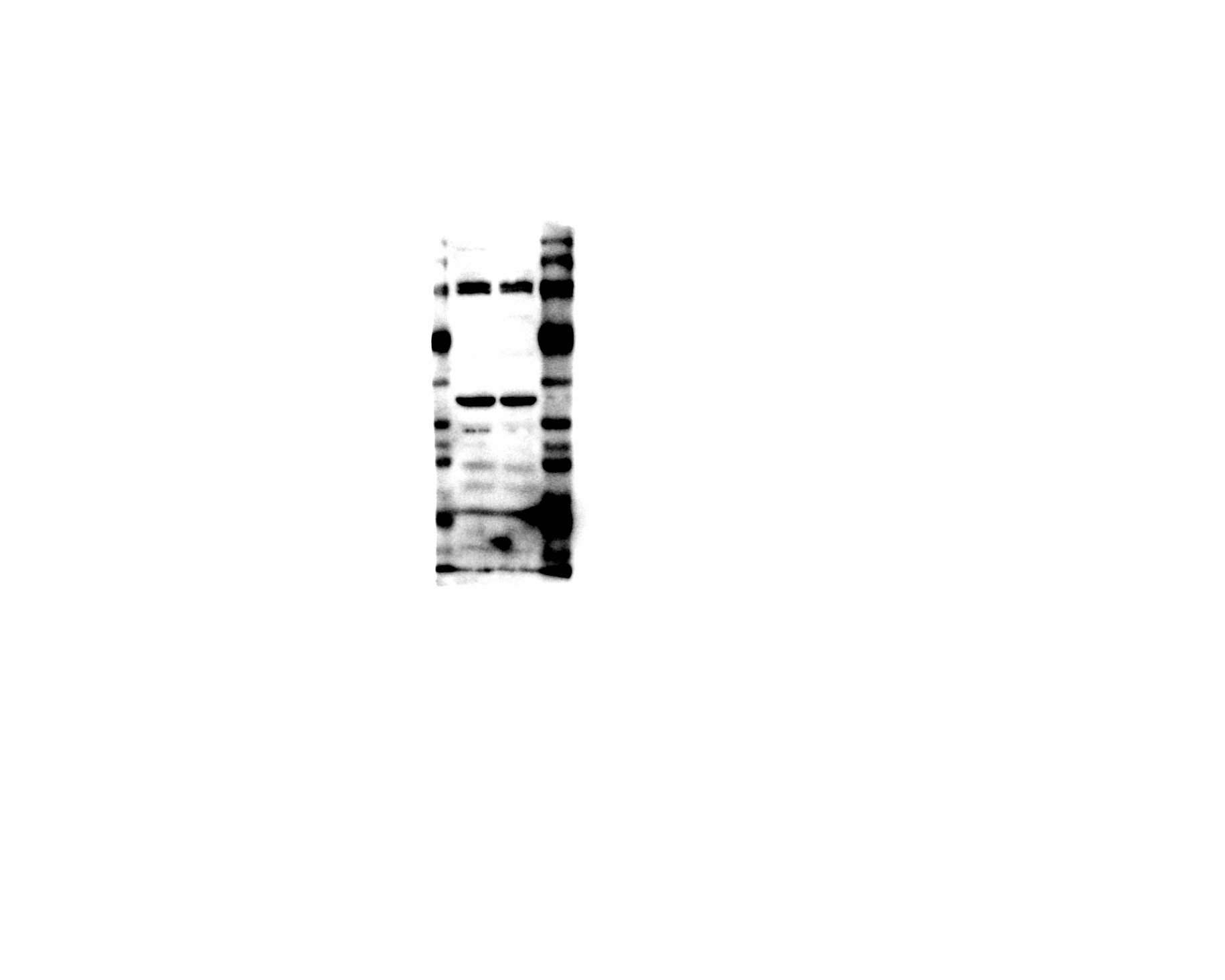

Supplement: Supplementary Figure 1 — Behavioral abnormalities in 9-month-old 3 × Tg-AD mice. (A) Schematic diagram of the different age stages of animals and experimental procedures. (B,C) Spatial cognitive ability and escape latency in 9-month-old WT and 3 × Tg-AD mice (n > 6 mice per group). (D) Evaluation of motor function in mice: statistical graphs of persistence time in turning the stick (n = 6 mice per group). (E) Evaluating the spatial exploration ability of mice: a statistical chart of the distance mice move (n = 6 mice per group). (F) Nesting ability in 9-month-old WT and 3 × Tg-AD mice (n = 4 mice per group). [file Data_Sheet_2.zip › FIG1/HT22 HK/original data/wb 2022-11-01 2'1 hk.tif]

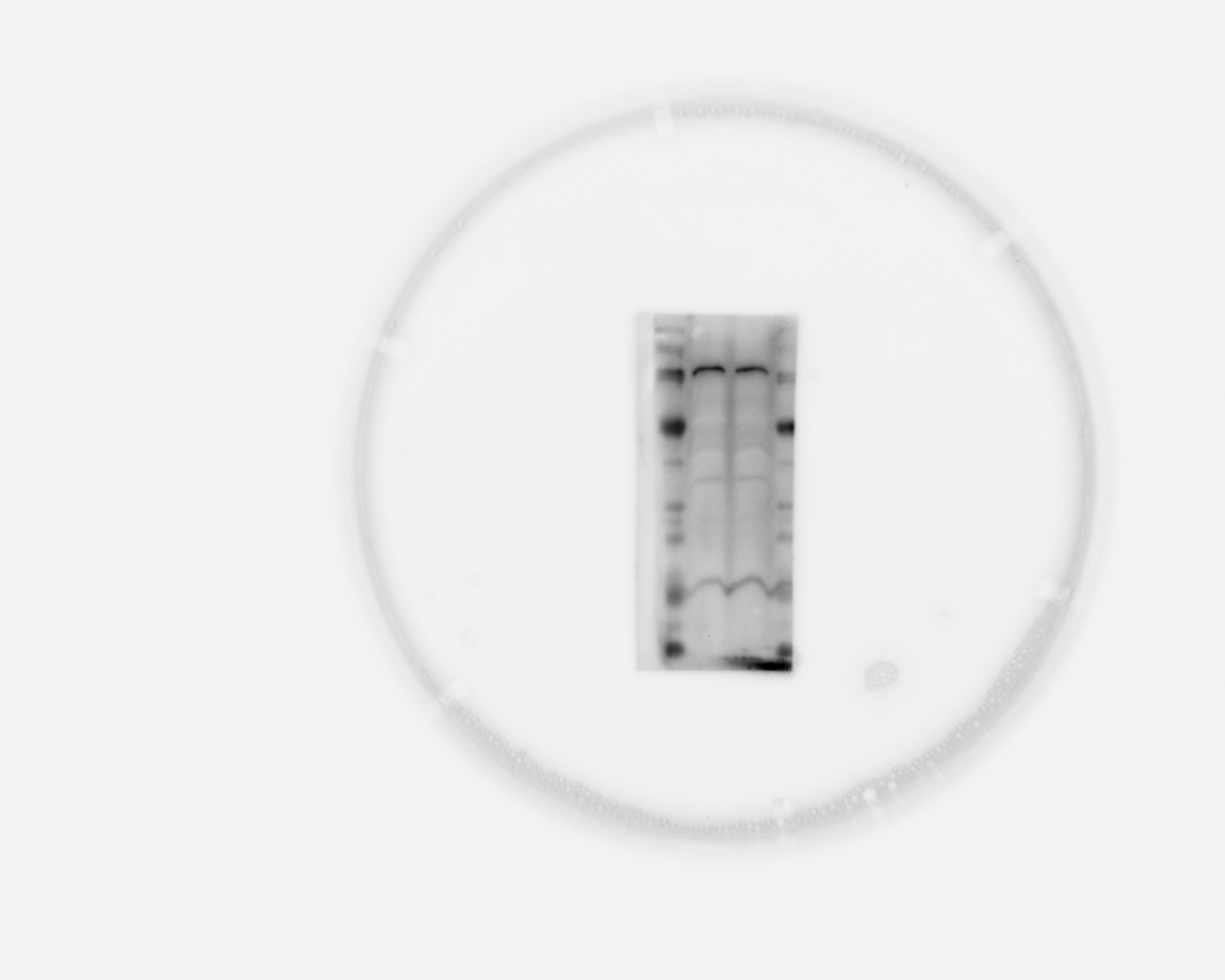

Supplement: Supplementary Figure 1 — Behavioral abnormalities in 9-month-old 3 × Tg-AD mice. (A) Schematic diagram of the different age stages of animals and experimental procedures. (B,C) Spatial cognitive ability and escape latency in 9-month-old WT and 3 × Tg-AD mice (n > 6 mice per group). (D) Evaluation of motor function in mice: statistical graphs of persistence time in turning the stick (n = 6 mice per group). (E) Evaluating the spatial exploration ability of mice: a statistical chart of the distance mice move (n = 6 mice per group). (F) Nesting ability in 9-month-old WT and 3 × Tg-AD mice (n = 4 mice per group). [file Data_Sheet_2.zip › FIG1/HT22 HK/original data/wb 2022-11-01 4'1hk.tif]

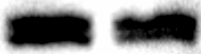

Supplement: Supplementary Figure 1 — Behavioral abnormalities in 9-month-old 3 × Tg-AD mice. (A) Schematic diagram of the different age stages of animals and experimental procedures. (B,C) Spatial cognitive ability and escape latency in 9-month-old WT and 3 × Tg-AD mice (n > 6 mice per group). (D) Evaluation of motor function in mice: statistical graphs of persistence time in turning the stick (n = 6 mice per group). (E) Evaluating the spatial exploration ability of mice: a statistical chart of the distance mice move (n = 6 mice per group). (F) Nesting ability in 9-month-old WT and 3 × Tg-AD mice (n = 4 mice per group). [file Data_Sheet_2.zip › FIG1/HT22 HK/wb 2022-11-01 jxh 2'1 hk.png]

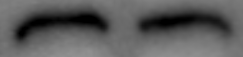

Supplement: Supplementary Figure 1 — Behavioral abnormalities in 9-month-old 3 × Tg-AD mice. (A) Schematic diagram of the different age stages of animals and experimental procedures. (B,C) Spatial cognitive ability and escape latency in 9-month-old WT and 3 × Tg-AD mice (n > 6 mice per group). (D) Evaluation of motor function in mice: statistical graphs of persistence time in turning the stick (n = 6 mice per group). (E) Evaluating the spatial exploration ability of mice: a statistical chart of the distance mice move (n = 6 mice per group). (F) Nesting ability in 9-month-old WT and 3 × Tg-AD mice (n = 4 mice per group). [file Data_Sheet_2.zip › FIG1/HT22 HK/wb 2022-11-01 jxh 4'1HK.png]

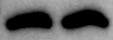

Supplement: Supplementary Figure 1 — Behavioral abnormalities in 9-month-old 3 × Tg-AD mice. (A) Schematic diagram of the different age stages of animals and experimental procedures. (B,C) Spatial cognitive ability and escape latency in 9-month-old WT and 3 × Tg-AD mice (n > 6 mice per group). (D) Evaluation of motor function in mice: statistical graphs of persistence time in turning the stick (n = 6 mice per group). (E) Evaluating the spatial exploration ability of mice: a statistical chart of the distance mice move (n = 6 mice per group). (F) Nesting ability in 9-month-old WT and 3 × Tg-AD mice (n = 4 mice per group). [file Data_Sheet_2.zip › FIG1/HT22 HK/wb 2022-11-01 jxh 4'1TUB.png]

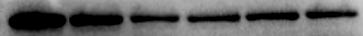

Supplement: Supplementary Figure 1 — Behavioral abnormalities in 9-month-old 3 × Tg-AD mice. (A) Schematic diagram of the different age stages of animals and experimental procedures. (B,C) Spatial cognitive ability and escape latency in 9-month-old WT and 3 × Tg-AD mice (n > 6 mice per group). (D) Evaluation of motor function in mice: statistical graphs of persistence time in turning the stick (n = 6 mice per group). (E) Evaluating the spatial exploration ability of mice: a statistical chart of the distance mice move (n = 6 mice per group). (F) Nesting ability in 9-month-old WT and 3 × Tg-AD mice (n = 4 mice per group). [file Data_Sheet_2.zip › FIG1/in vivo(HK pro)/2022-09-29 jxh 7hk.png]

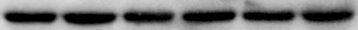

Supplement: Supplementary Figure 1 — Behavioral abnormalities in 9-month-old 3 × Tg-AD mice. (A) Schematic diagram of the different age stages of animals and experimental procedures. (B,C) Spatial cognitive ability and escape latency in 9-month-old WT and 3 × Tg-AD mice (n > 6 mice per group). (D) Evaluation of motor function in mice: statistical graphs of persistence time in turning the stick (n = 6 mice per group). (E) Evaluating the spatial exploration ability of mice: a statistical chart of the distance mice move (n = 6 mice per group). (F) Nesting ability in 9-month-old WT and 3 × Tg-AD mice (n = 4 mice per group). [file Data_Sheet_2.zip › FIG1/in vivo(HK pro)/2022-10-01 jxh 7tub.png]

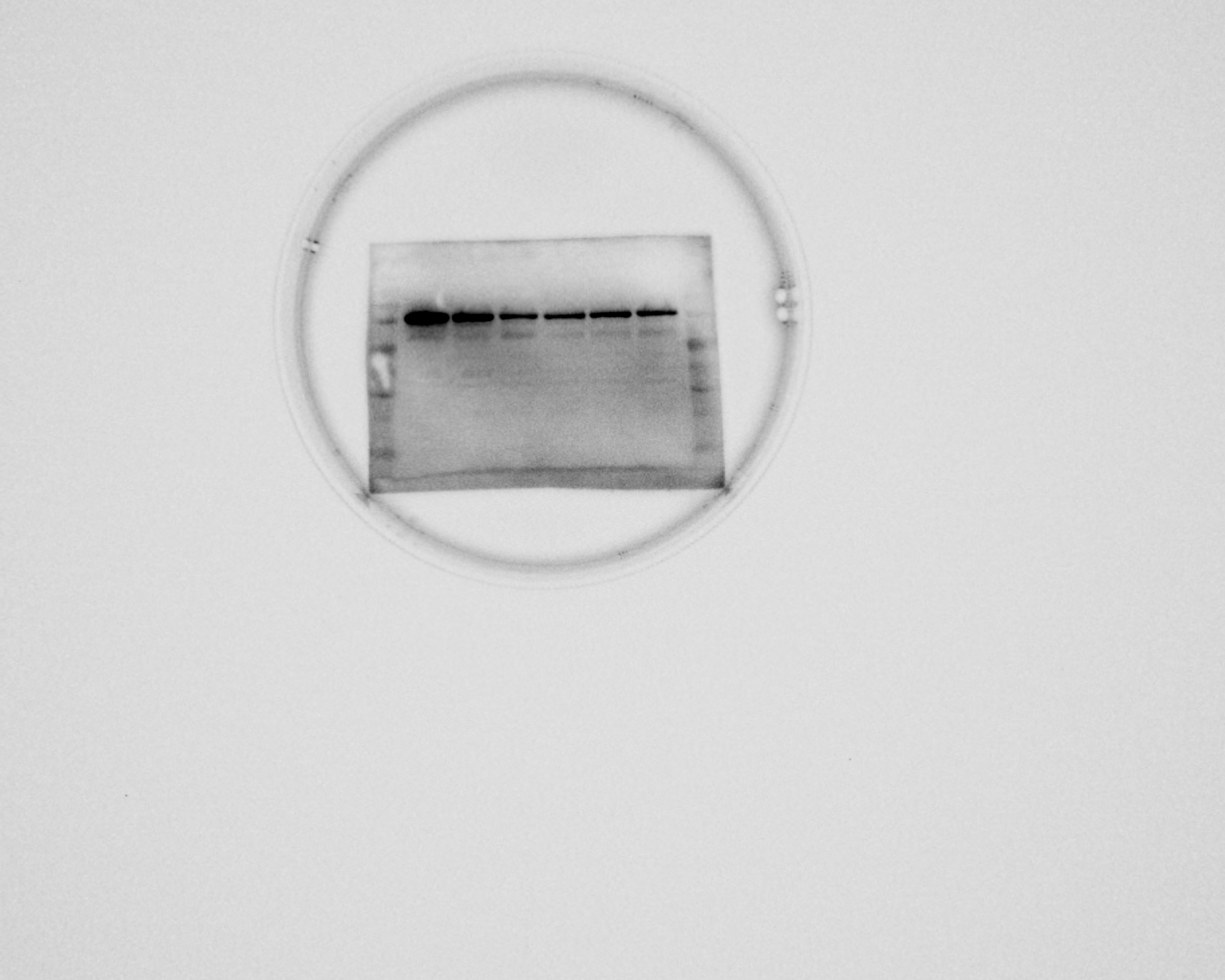

Supplement: Supplementary Figure 1 — Behavioral abnormalities in 9-month-old 3 × Tg-AD mice. (A) Schematic diagram of the different age stages of animals and experimental procedures. (B,C) Spatial cognitive ability and escape latency in 9-month-old WT and 3 × Tg-AD mice (n > 6 mice per group). (D) Evaluation of motor function in mice: statistical graphs of persistence time in turning the stick (n = 6 mice per group). (E) Evaluating the spatial exploration ability of mice: a statistical chart of the distance mice move (n = 6 mice per group). (F) Nesting ability in 9-month-old WT and 3 × Tg-AD mice (n = 4 mice per group). [file Data_Sheet_2.zip › FIG1/in vivo(HK pro)/original data/2022-09-29 7hk.tif]

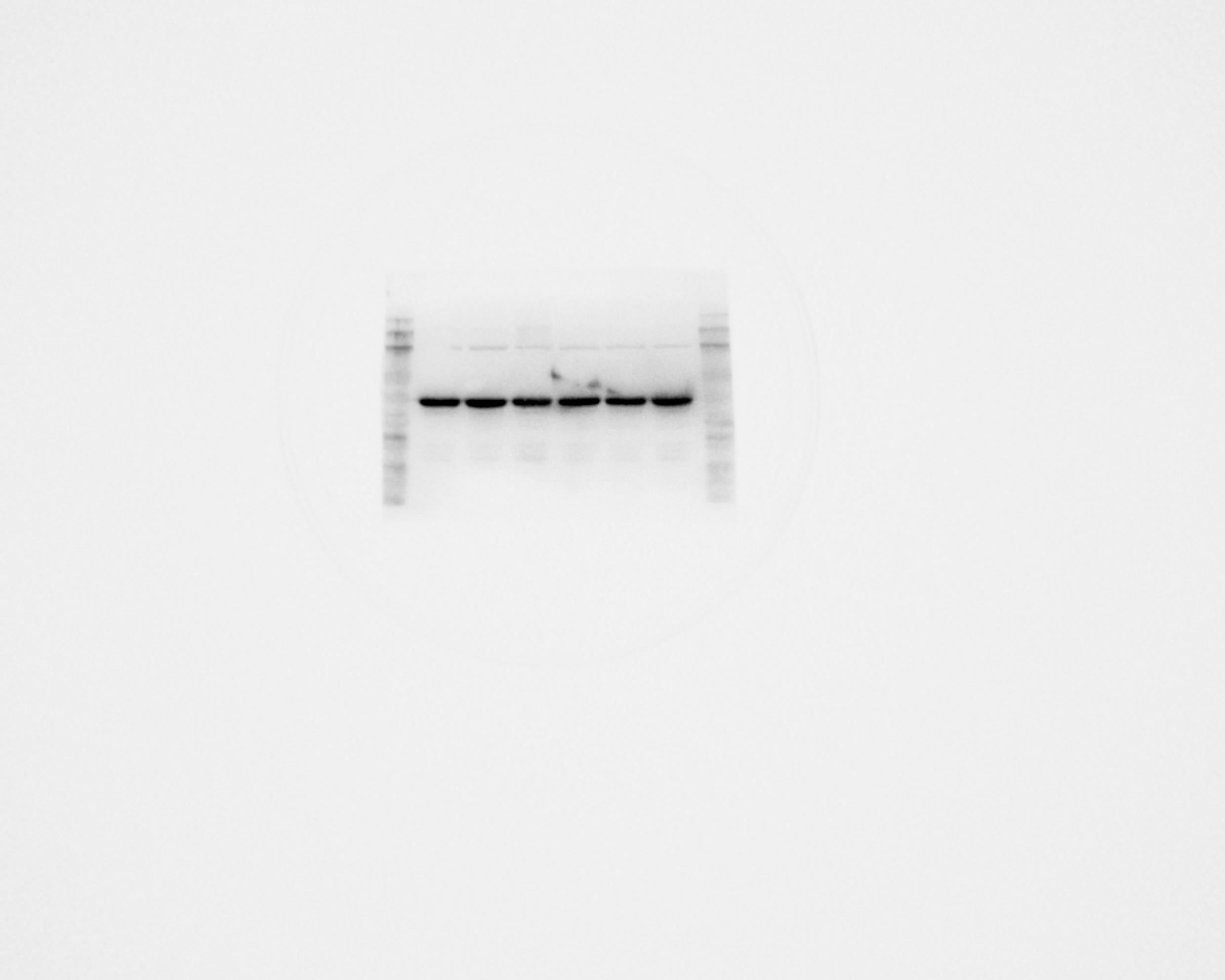

Supplement: Supplementary Figure 1 — Behavioral abnormalities in 9-month-old 3 × Tg-AD mice. (A) Schematic diagram of the different age stages of animals and experimental procedures. (B,C) Spatial cognitive ability and escape latency in 9-month-old WT and 3 × Tg-AD mice (n > 6 mice per group). (D) Evaluation of motor function in mice: statistical graphs of persistence time in turning the stick (n = 6 mice per group). (E) Evaluating the spatial exploration ability of mice: a statistical chart of the distance mice move (n = 6 mice per group). (F) Nesting ability in 9-month-old WT and 3 × Tg-AD mice (n = 4 mice per group). [file Data_Sheet_2.zip › FIG1/in vivo(HK pro)/original data/2022-10-01 7tub.tif]

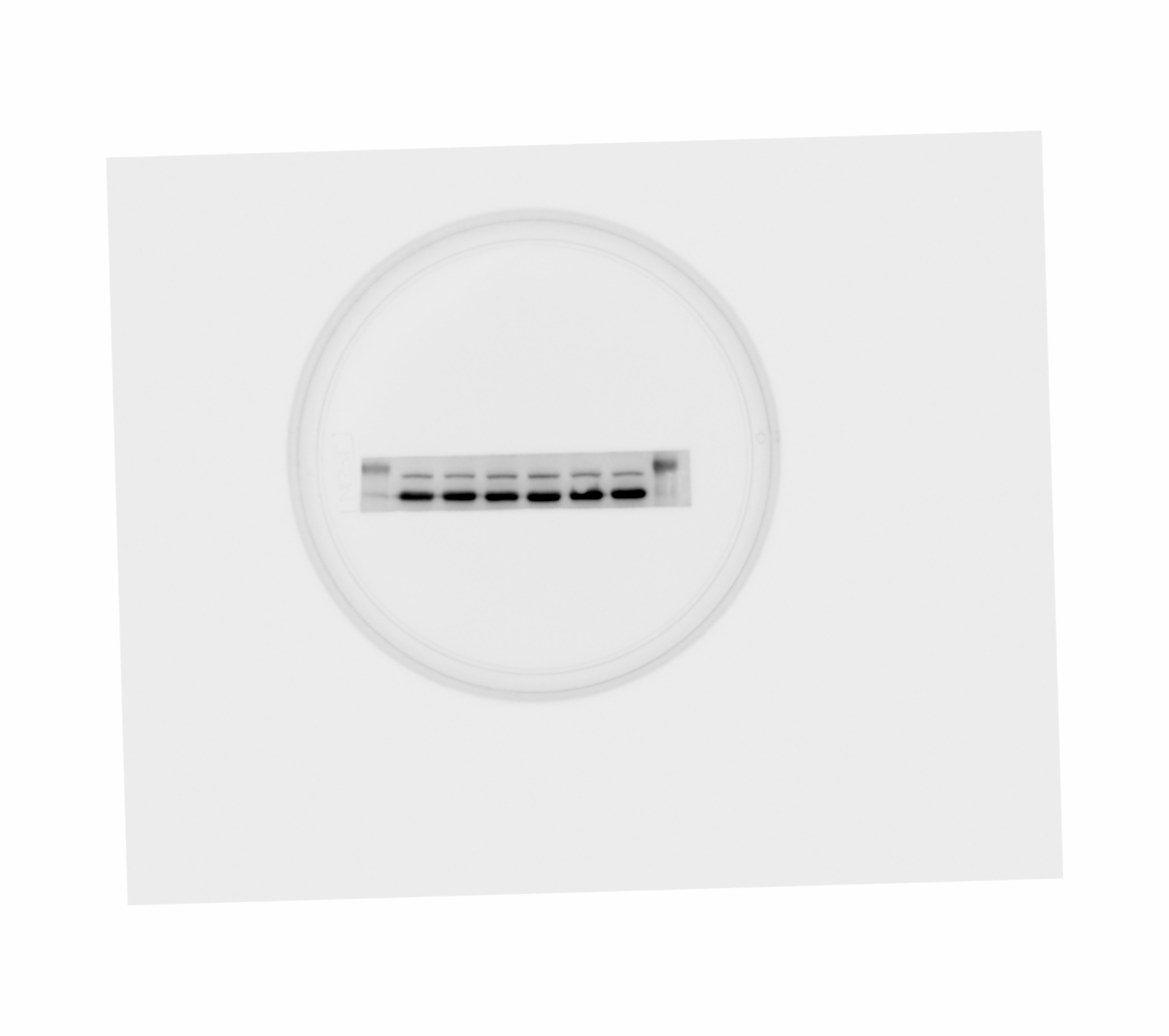

Supplement: Supplementary Figure 1 — Behavioral abnormalities in 9-month-old 3 × Tg-AD mice. (A) Schematic diagram of the different age stages of animals and experimental procedures. (B,C) Spatial cognitive ability and escape latency in 9-month-old WT and 3 × Tg-AD mice (n > 6 mice per group). (D) Evaluation of motor function in mice: statistical graphs of persistence time in turning the stick (n = 6 mice per group). (E) Evaluating the spatial exploration ability of mice: a statistical chart of the distance mice move (n = 6 mice per group). (F) Nesting ability in 9-month-old WT and 3 × Tg-AD mice (n = 4 mice per group). [file Data_Sheet_2.zip › FIG1/in vivo(HK pro)/original data/wb 2022-09-11 7tub.tif]

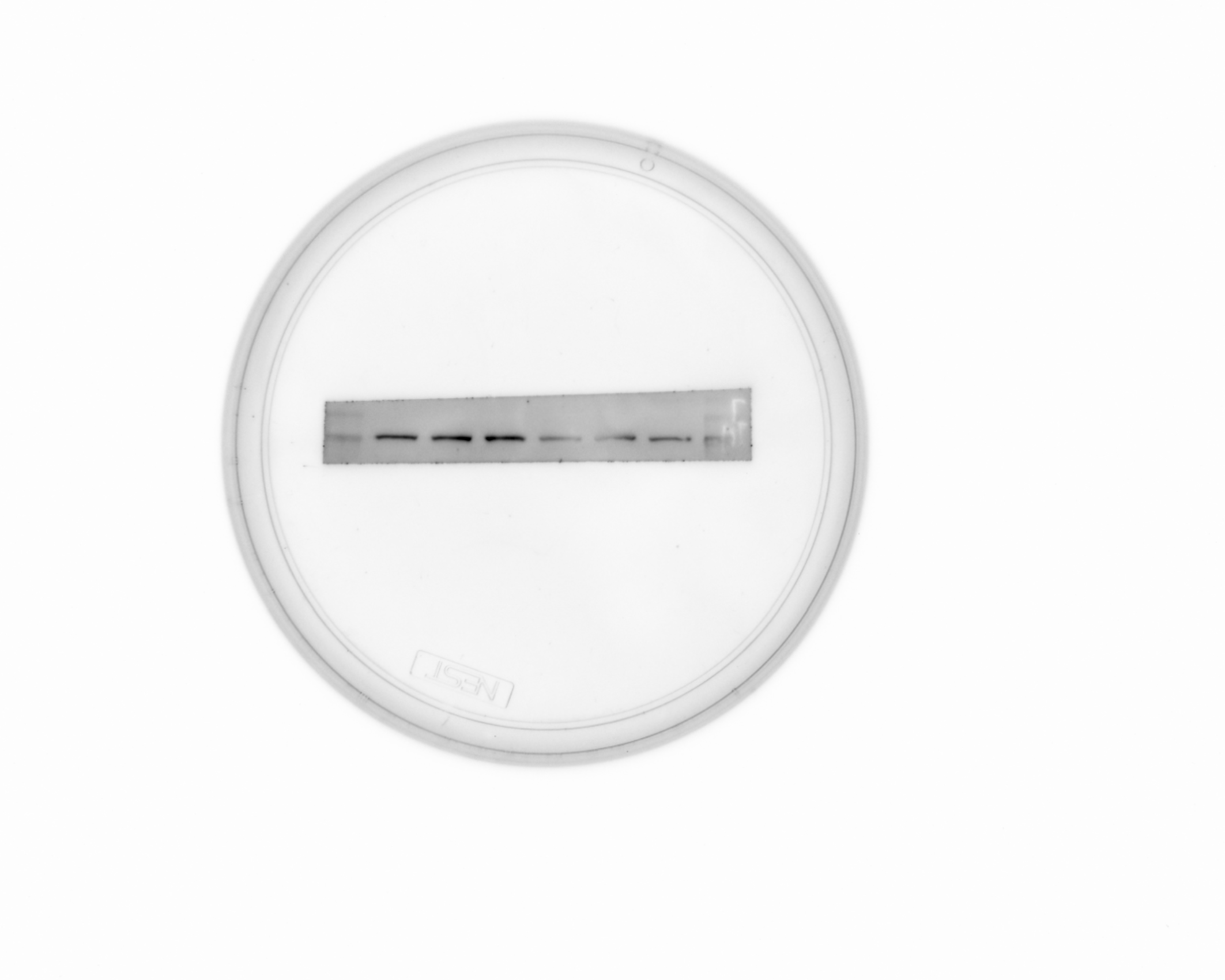

Supplement: Supplementary Figure 1 — Behavioral abnormalities in 9-month-old 3 × Tg-AD mice. (A) Schematic diagram of the different age stages of animals and experimental procedures. (B,C) Spatial cognitive ability and escape latency in 9-month-old WT and 3 × Tg-AD mice (n > 6 mice per group). (D) Evaluation of motor function in mice: statistical graphs of persistence time in turning the stick (n = 6 mice per group). (E) Evaluating the spatial exploration ability of mice: a statistical chart of the distance mice move (n = 6 mice per group). (F) Nesting ability in 9-month-old WT and 3 × Tg-AD mice (n = 4 mice per group). [file Data_Sheet_2.zip › FIG1/in vivo(HK pro)/original data/wb 2022-09-11 7hk.tif]

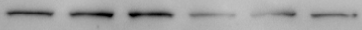

Supplement: Supplementary Figure 1 — Behavioral abnormalities in 9-month-old 3 × Tg-AD mice. (A) Schematic diagram of the different age stages of animals and experimental procedures. (B,C) Spatial cognitive ability and escape latency in 9-month-old WT and 3 × Tg-AD mice (n > 6 mice per group). (D) Evaluation of motor function in mice: statistical graphs of persistence time in turning the stick (n = 6 mice per group). (E) Evaluating the spatial exploration ability of mice: a statistical chart of the distance mice move (n = 6 mice per group). (F) Nesting ability in 9-month-old WT and 3 × Tg-AD mice (n = 4 mice per group). [file Data_Sheet_2.zip › FIG1/in vivo(HK pro)/wb 2022-09-11 jxh 7hk.png]

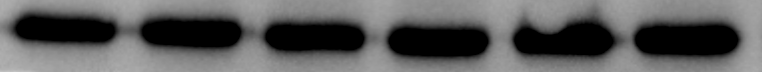

Supplement: Supplementary Figure 1 — Behavioral abnormalities in 9-month-old 3 × Tg-AD mice. (A) Schematic diagram of the different age stages of animals and experimental procedures. (B,C) Spatial cognitive ability and escape latency in 9-month-old WT and 3 × Tg-AD mice (n > 6 mice per group). (D) Evaluation of motor function in mice: statistical graphs of persistence time in turning the stick (n = 6 mice per group). (E) Evaluating the spatial exploration ability of mice: a statistical chart of the distance mice move (n = 6 mice per group). (F) Nesting ability in 9-month-old WT and 3 × Tg-AD mice (n = 4 mice per group). [file Data_Sheet_2.zip › FIG1/in vivo(HK pro)/wb 2022-09-11 jxh 7tub.png]

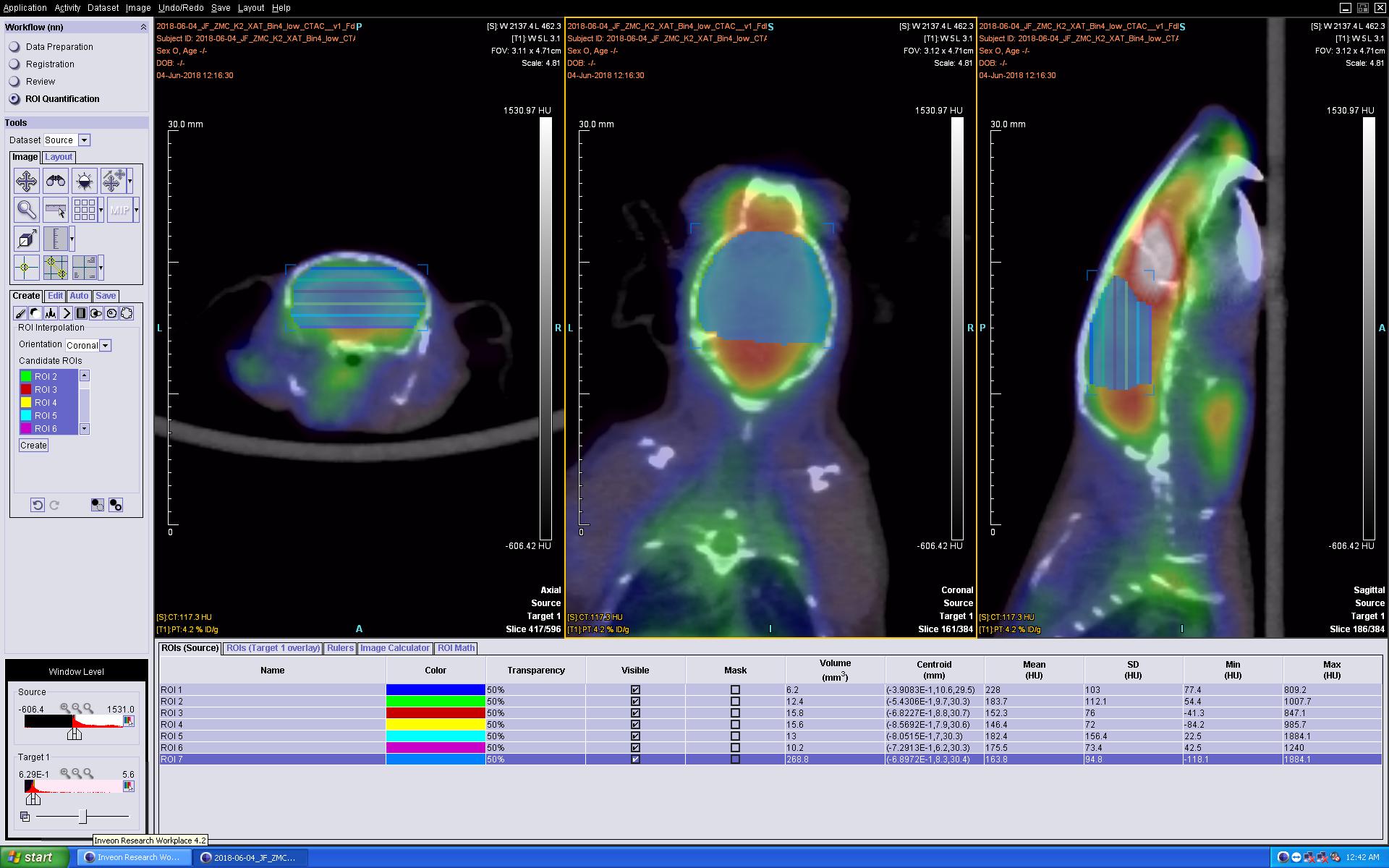

Supplement: Supplementary Figure 2 — Immunohistochemistry of hippocampal tissues from AD mice. (A) Histological changes in the hippocampus of 9-month-old WT and 3 × Tg-AD mice were analyzed by HE staining. (B–D) Analysis of neuronal status in the hippocampus of 9-month-old WT and 3 × Tg-AD mice by Nissler staining (n = 3 mice per group). (E–H) Analysis and quantification of Aβ deposition in hippocampal tissue of 9-month-old WT and 3 × Tg-AD mice by immunofluorescence (n = 4 mice per group). [file Data_Sheet_1.zip › FIG 2 pET-CT/PET-CT/K2/10.JPG]

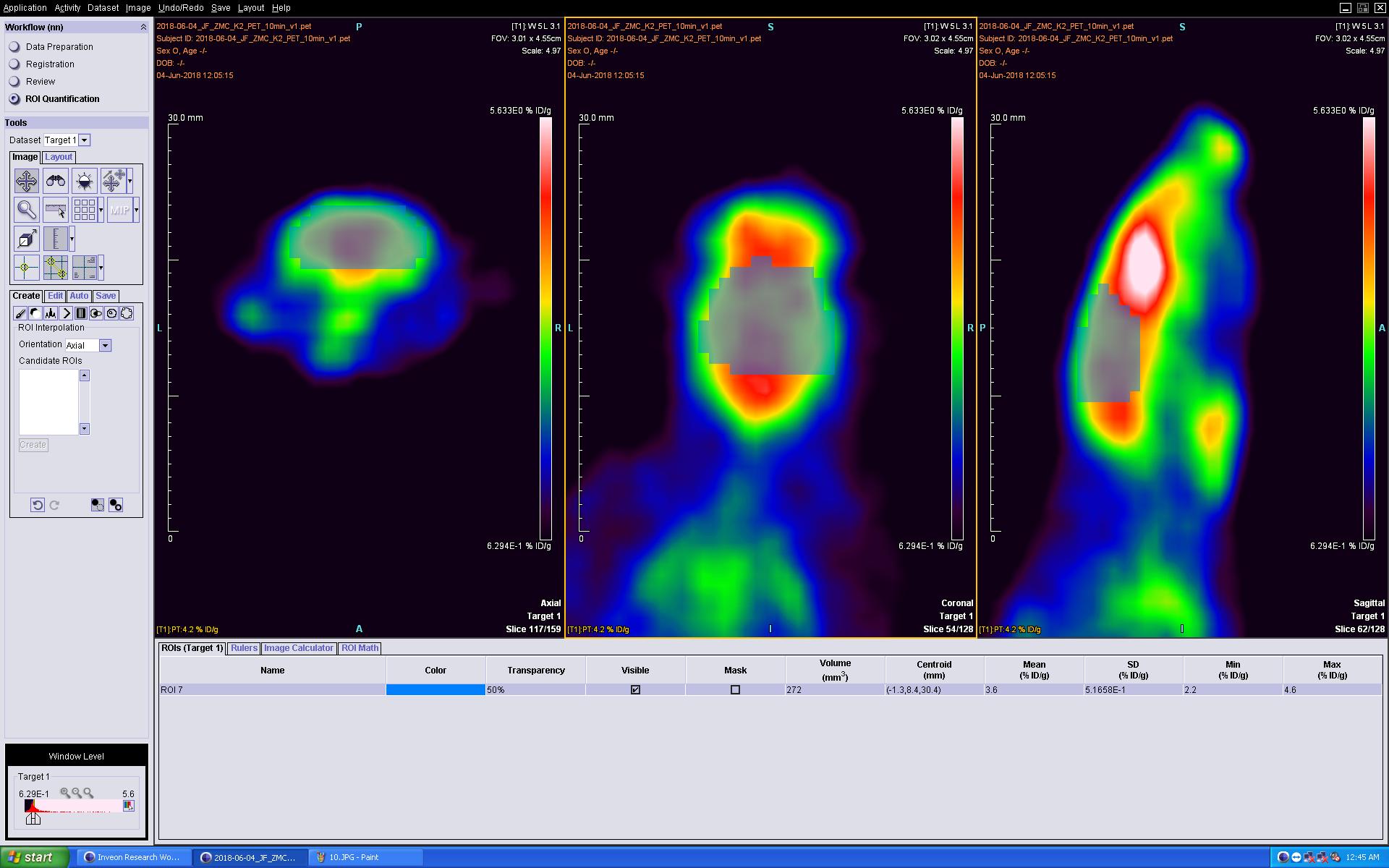

Supplement: Supplementary Figure 2 — Immunohistochemistry of hippocampal tissues from AD mice. (A) Histological changes in the hippocampus of 9-month-old WT and 3 × Tg-AD mice were analyzed by HE staining. (B–D) Analysis of neuronal status in the hippocampus of 9-month-old WT and 3 × Tg-AD mice by Nissler staining (n = 3 mice per group). (E–H) Analysis and quantification of Aβ deposition in hippocampal tissue of 9-month-old WT and 3 × Tg-AD mice by immunofluorescence (n = 4 mice per group). [file Data_Sheet_1.zip › FIG 2 pET-CT/PET-CT/K2/11.JPG]

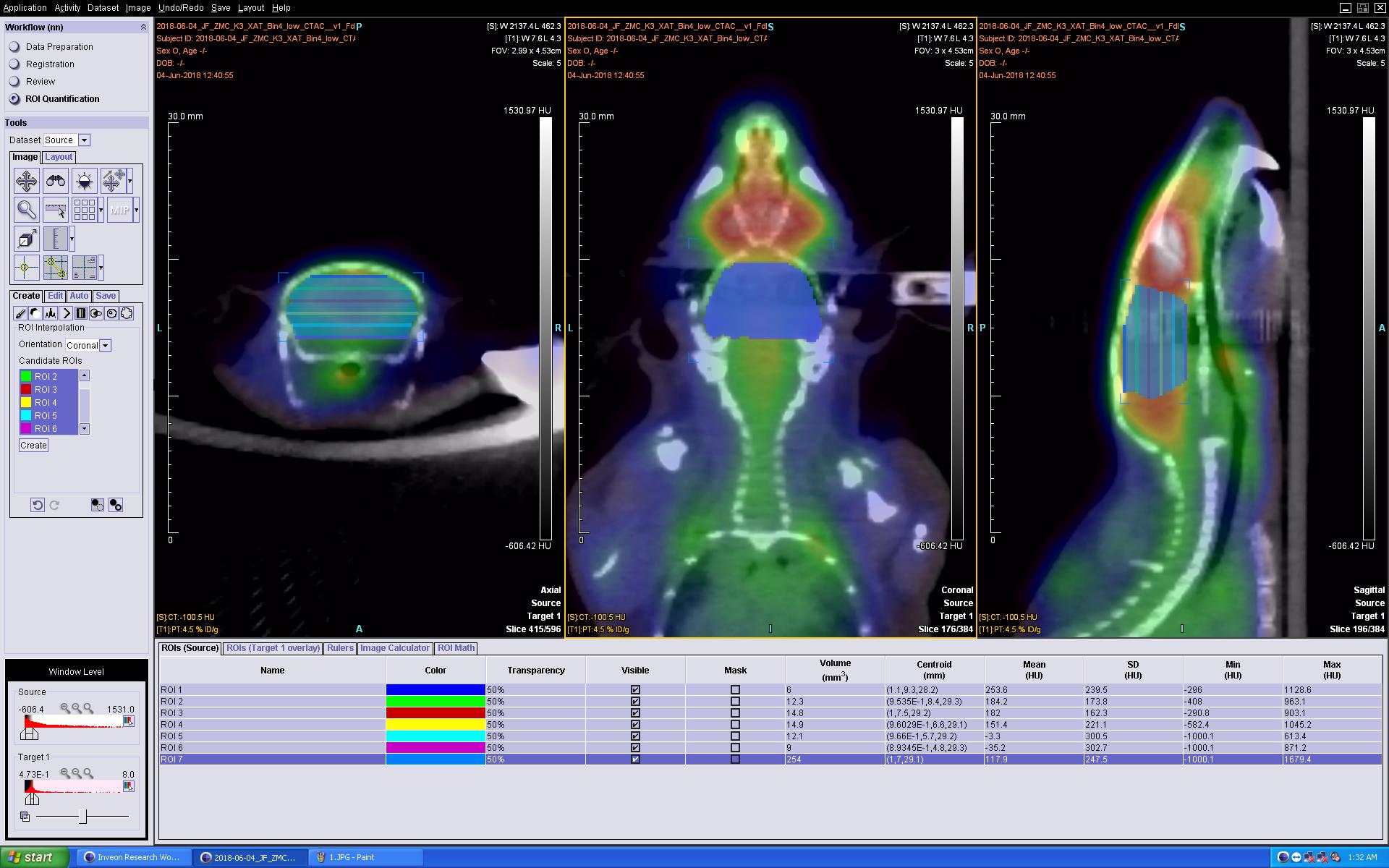

Supplement: Supplementary Figure 2 — Immunohistochemistry of hippocampal tissues from AD mice. (A) Histological changes in the hippocampus of 9-month-old WT and 3 × Tg-AD mice were analyzed by HE staining. (B–D) Analysis of neuronal status in the hippocampus of 9-month-old WT and 3 × Tg-AD mice by Nissler staining (n = 3 mice per group). (E–H) Analysis and quantification of Aβ deposition in hippocampal tissue of 9-month-old WT and 3 × Tg-AD mice by immunofluorescence (n = 4 mice per group). [file Data_Sheet_1.zip › FIG 2 pET-CT/PET-CT/K3/1.JPG]

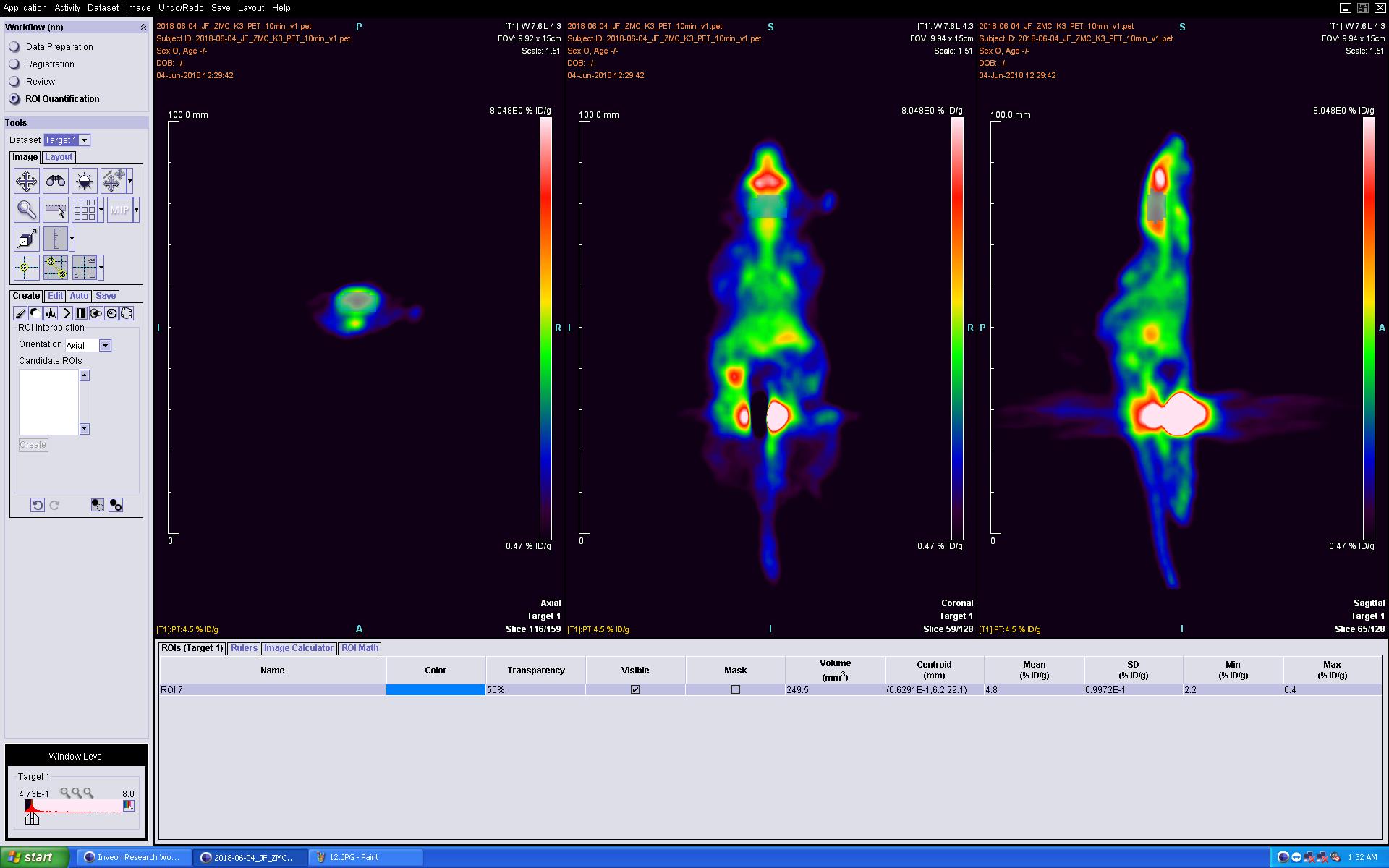

Supplement: Supplementary Figure 2 — Immunohistochemistry of hippocampal tissues from AD mice. (A) Histological changes in the hippocampus of 9-month-old WT and 3 × Tg-AD mice were analyzed by HE staining. (B–D) Analysis of neuronal status in the hippocampus of 9-month-old WT and 3 × Tg-AD mice by Nissler staining (n = 3 mice per group). (E–H) Analysis and quantification of Aβ deposition in hippocampal tissue of 9-month-old WT and 3 × Tg-AD mice by immunofluorescence (n = 4 mice per group). [file Data_Sheet_1.zip › FIG 2 pET-CT/PET-CT/K3/13.JPG]

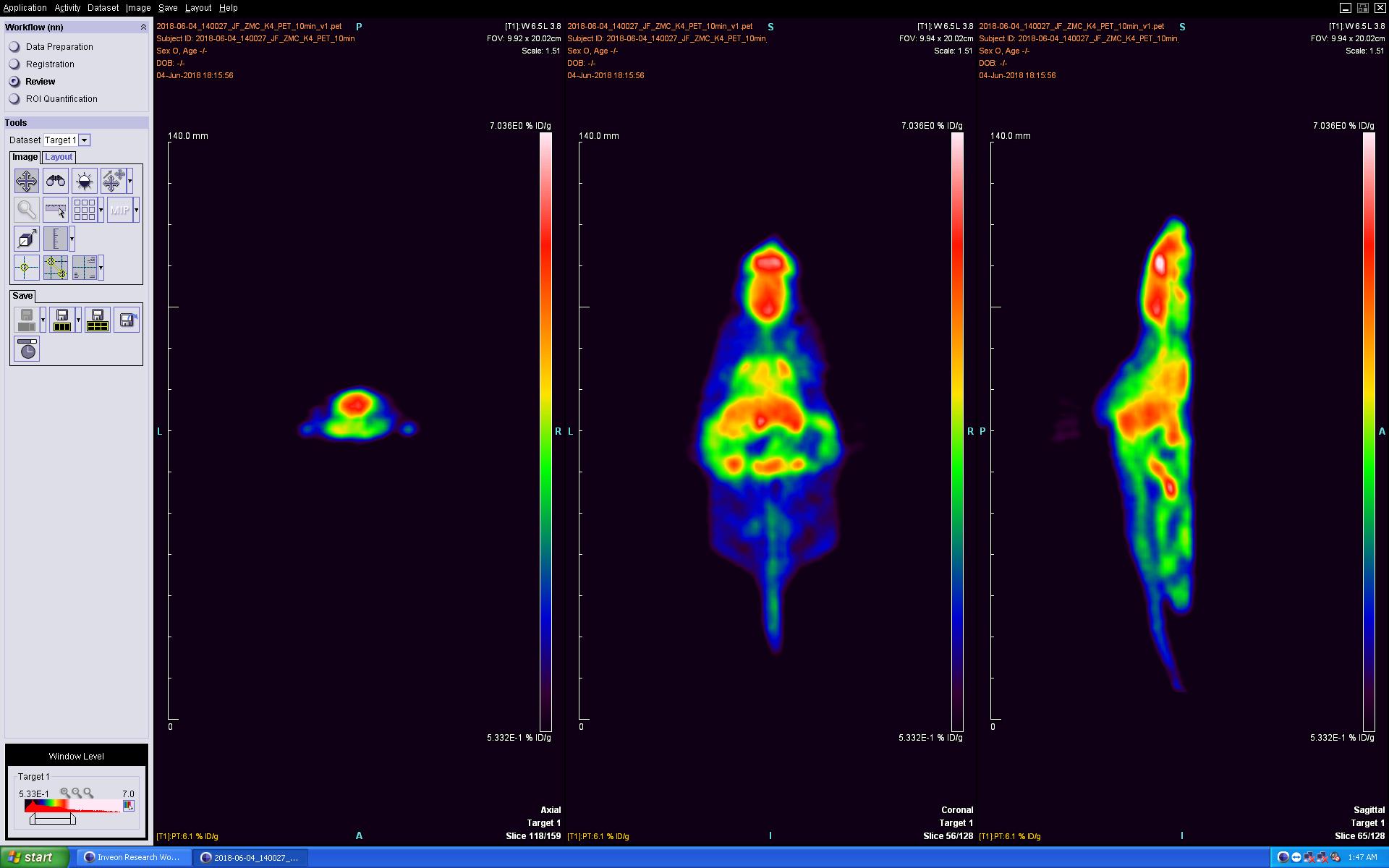

Supplement: Supplementary Figure 2 — Immunohistochemistry of hippocampal tissues from AD mice. (A) Histological changes in the hippocampus of 9-month-old WT and 3 × Tg-AD mice were analyzed by HE staining. (B–D) Analysis of neuronal status in the hippocampus of 9-month-old WT and 3 × Tg-AD mice by Nissler staining (n = 3 mice per group). (E–H) Analysis and quantification of Aβ deposition in hippocampal tissue of 9-month-old WT and 3 × Tg-AD mice by immunofluorescence (n = 4 mice per group). [file Data_Sheet_1.zip › FIG 2 pET-CT/PET-CT/K4/1.JPG]

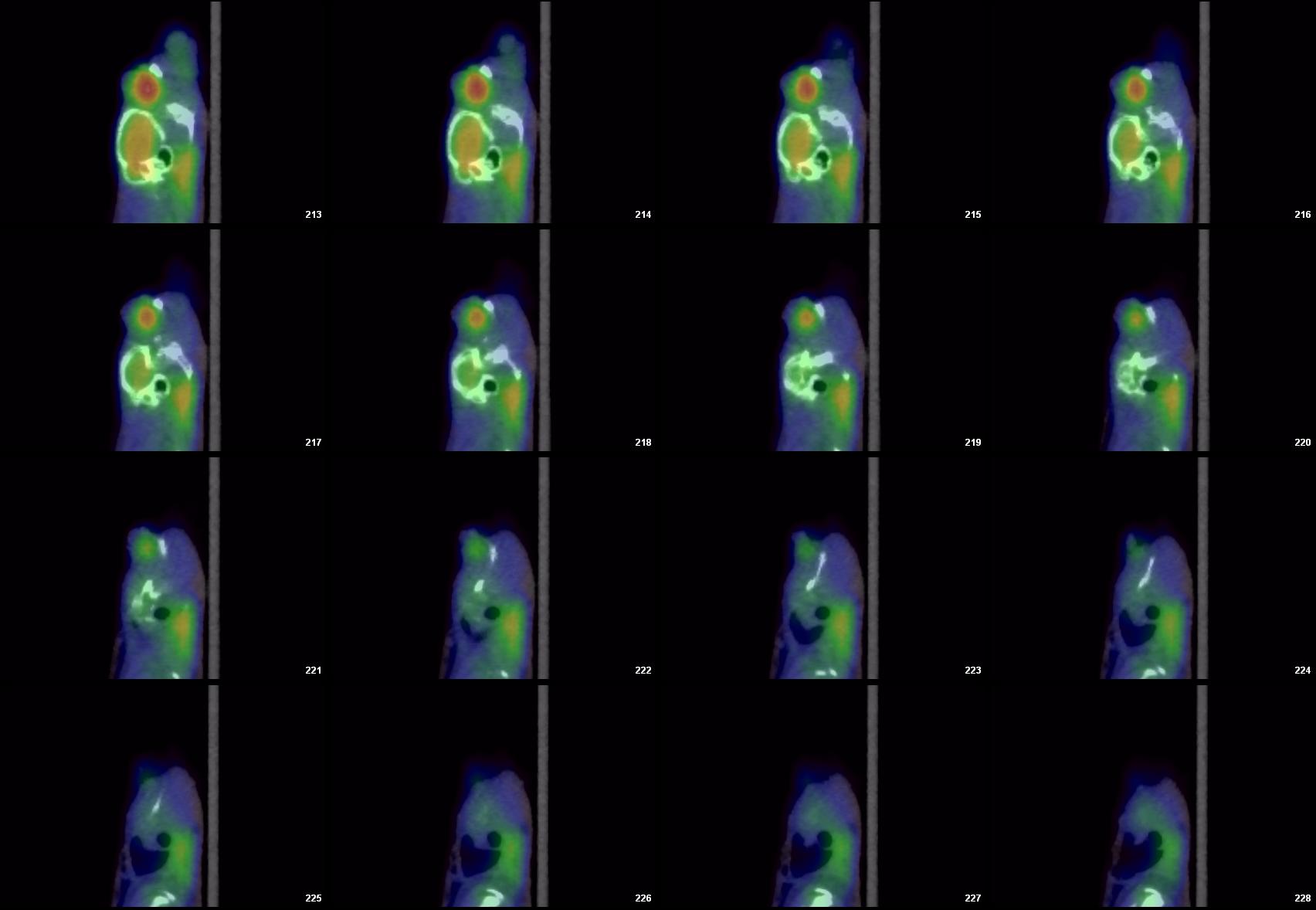

Supplement: Supplementary Figure 2 — Immunohistochemistry of hippocampal tissues from AD mice. (A) Histological changes in the hippocampus of 9-month-old WT and 3 × Tg-AD mice were analyzed by HE staining. (B–D) Analysis of neuronal status in the hippocampus of 9-month-old WT and 3 × Tg-AD mice by Nissler staining (n = 3 mice per group). (E–H) Analysis and quantification of Aβ deposition in hippocampal tissue of 9-month-old WT and 3 × Tg-AD mice by immunofluorescence (n = 4 mice per group). [file Data_Sheet_1.zip › FIG 2 pET-CT/PET-CT/K4/12.jpg]

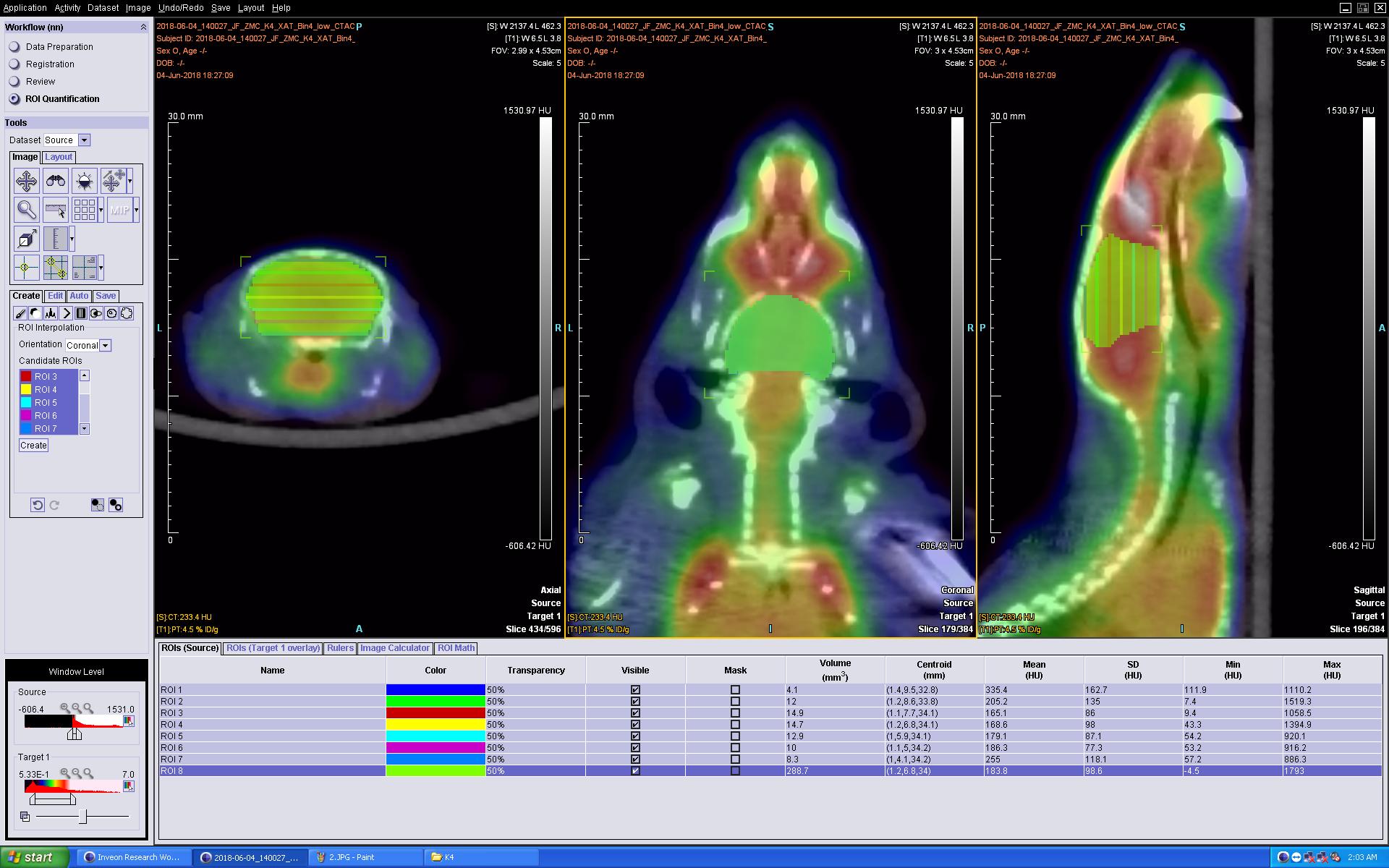

Supplement: Supplementary Figure 2 — Immunohistochemistry of hippocampal tissues from AD mice. (A) Histological changes in the hippocampus of 9-month-old WT and 3 × Tg-AD mice were analyzed by HE staining. (B–D) Analysis of neuronal status in the hippocampus of 9-month-old WT and 3 × Tg-AD mice by Nissler staining (n = 3 mice per group). (E–H) Analysis and quantification of Aβ deposition in hippocampal tissue of 9-month-old WT and 3 × Tg-AD mice by immunofluorescence (n = 4 mice per group). [file Data_Sheet_1.zip › FIG 2 pET-CT/PET-CT/K4/13.JPG]

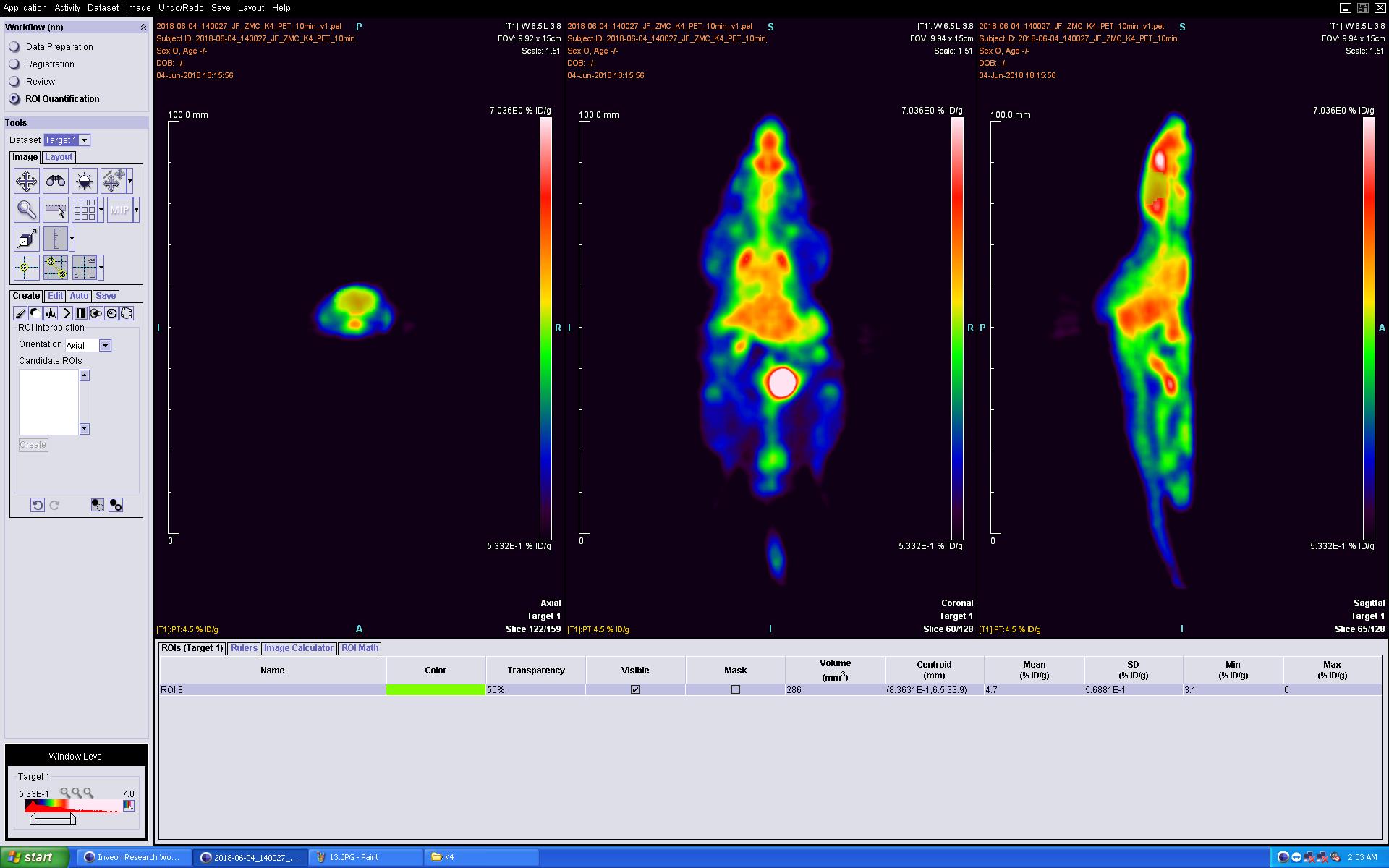

Supplement: Supplementary Figure 2 — Immunohistochemistry of hippocampal tissues from AD mice. (A) Histological changes in the hippocampus of 9-month-old WT and 3 × Tg-AD mice were analyzed by HE staining. (B–D) Analysis of neuronal status in the hippocampus of 9-month-old WT and 3 × Tg-AD mice by Nissler staining (n = 3 mice per group). (E–H) Analysis and quantification of Aβ deposition in hippocampal tissue of 9-month-old WT and 3 × Tg-AD mice by immunofluorescence (n = 4 mice per group). [file Data_Sheet_1.zip › FIG 2 pET-CT/PET-CT/K4/14.JPG]

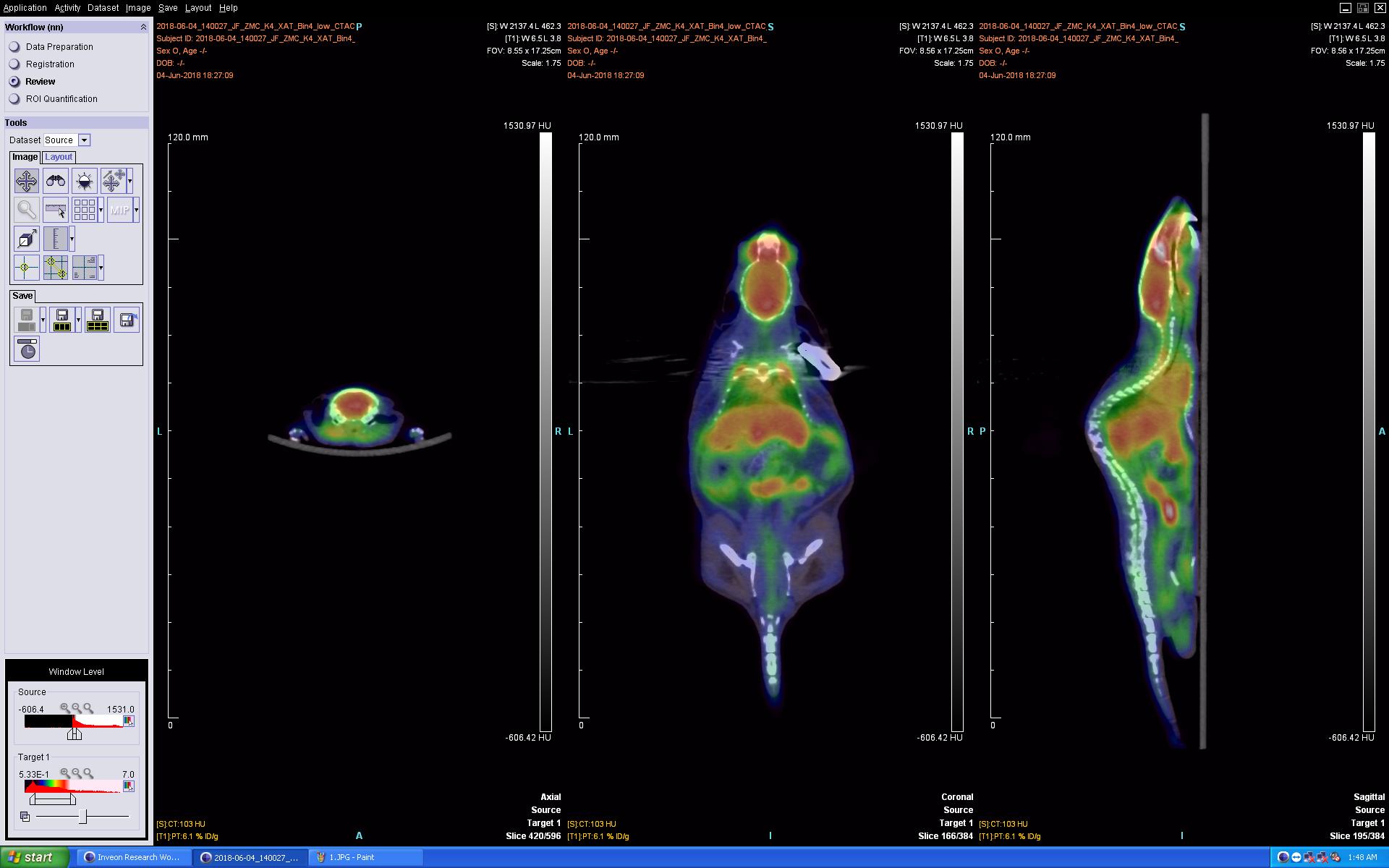

Supplement: Supplementary Figure 2 — Immunohistochemistry of hippocampal tissues from AD mice. (A) Histological changes in the hippocampus of 9-month-old WT and 3 × Tg-AD mice were analyzed by HE staining. (B–D) Analysis of neuronal status in the hippocampus of 9-month-old WT and 3 × Tg-AD mice by Nissler staining (n = 3 mice per group). (E–H) Analysis and quantification of Aβ deposition in hippocampal tissue of 9-month-old WT and 3 × Tg-AD mice by immunofluorescence (n = 4 mice per group). [file Data_Sheet_1.zip › FIG 2 pET-CT/PET-CT/K4/2.JPG]

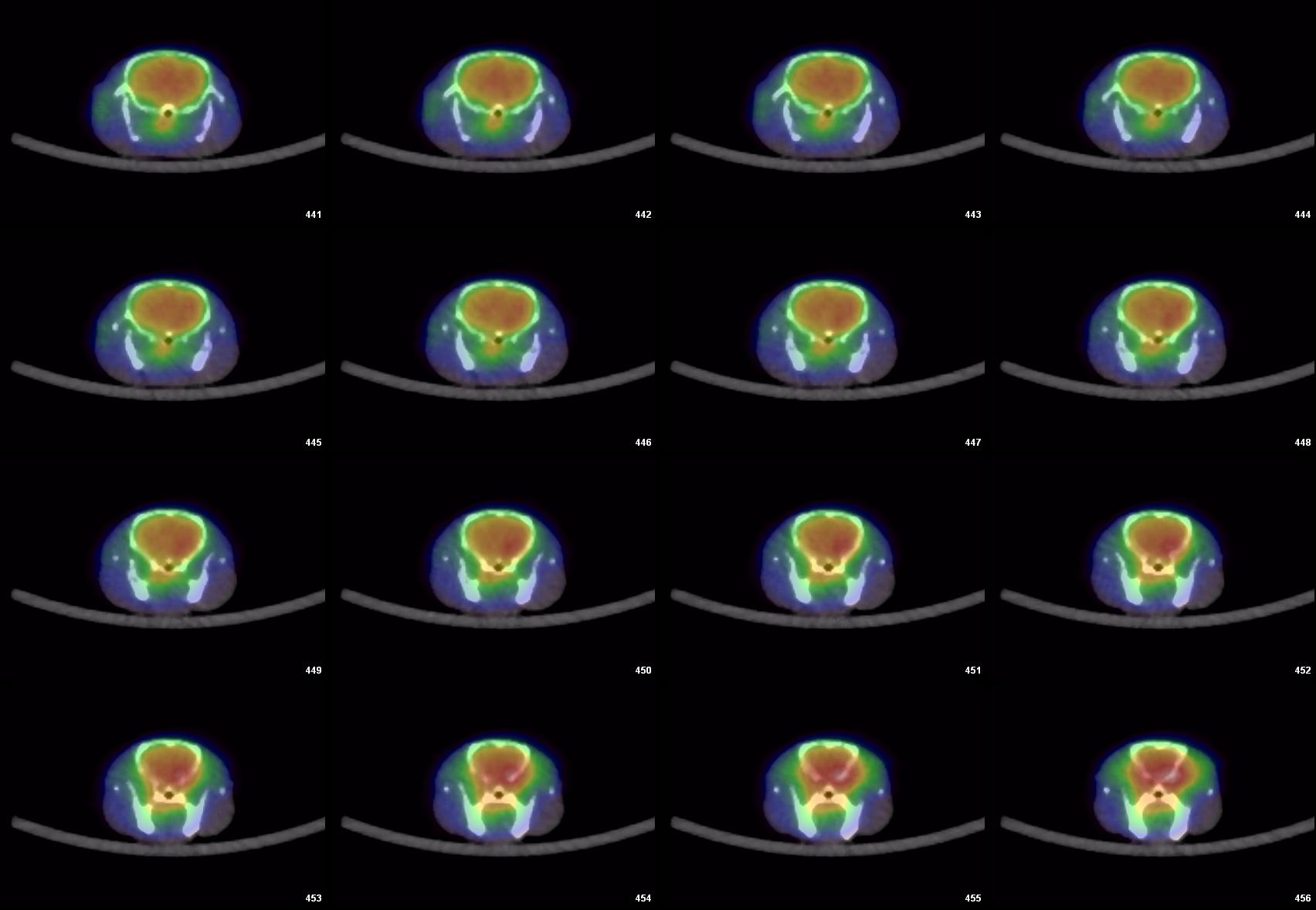

Supplement: Supplementary Figure 2 — Immunohistochemistry of hippocampal tissues from AD mice. (A) Histological changes in the hippocampus of 9-month-old WT and 3 × Tg-AD mice were analyzed by HE staining. (B–D) Analysis of neuronal status in the hippocampus of 9-month-old WT and 3 × Tg-AD mice by Nissler staining (n = 3 mice per group). (E–H) Analysis and quantification of Aβ deposition in hippocampal tissue of 9-month-old WT and 3 × Tg-AD mice by immunofluorescence (n = 4 mice per group). [file Data_Sheet_1.zip › FIG 2 pET-CT/PET-CT/K4/6.jpg]

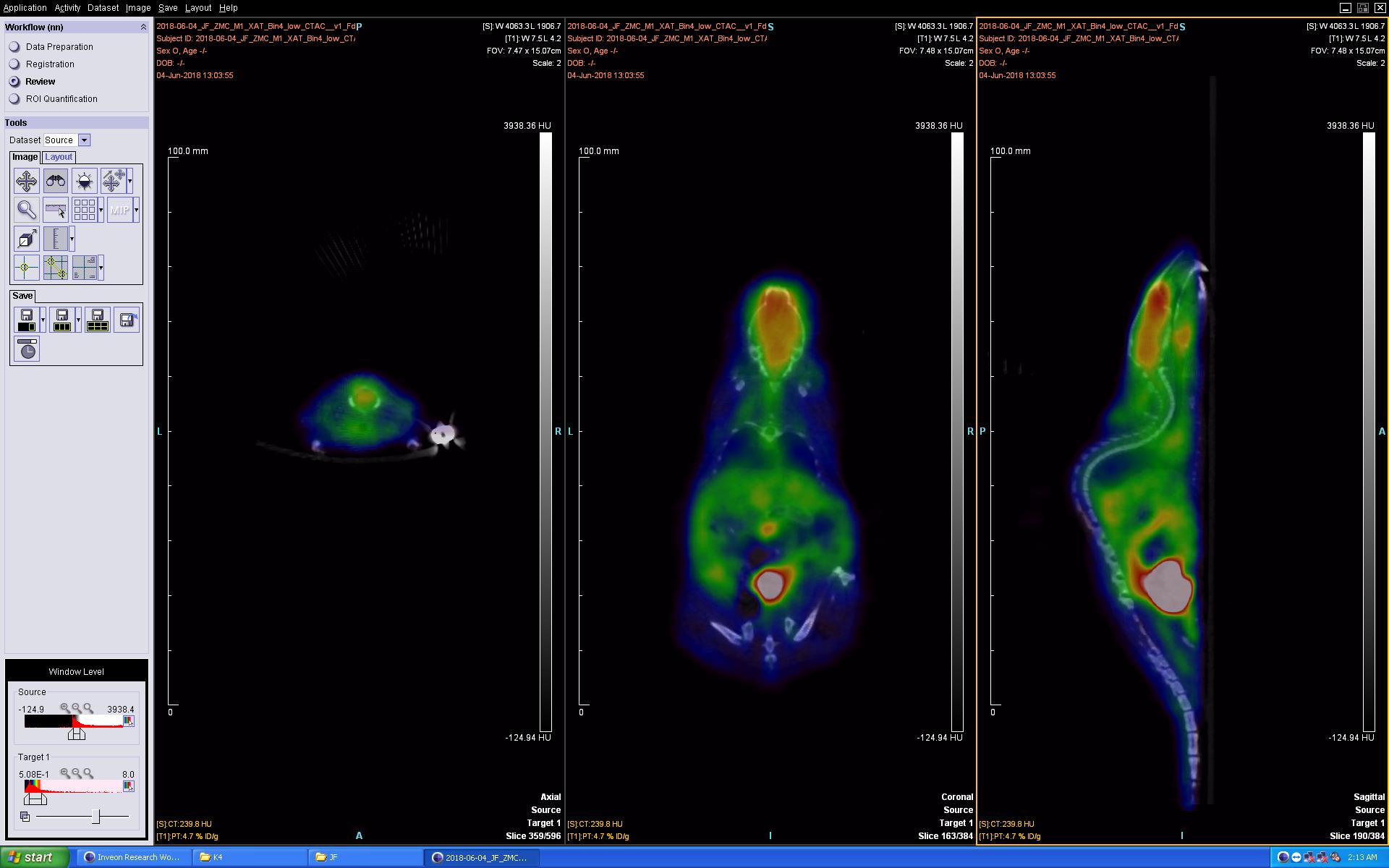

Supplement: Supplementary Figure 2 — Immunohistochemistry of hippocampal tissues from AD mice. (A) Histological changes in the hippocampus of 9-month-old WT and 3 × Tg-AD mice were analyzed by HE staining. (B–D) Analysis of neuronal status in the hippocampus of 9-month-old WT and 3 × Tg-AD mice by Nissler staining (n = 3 mice per group). (E–H) Analysis and quantification of Aβ deposition in hippocampal tissue of 9-month-old WT and 3 × Tg-AD mice by immunofluorescence (n = 4 mice per group). [file Data_Sheet_1.zip › FIG 2 pET-CT/PET-CT/M1/1.JPG]

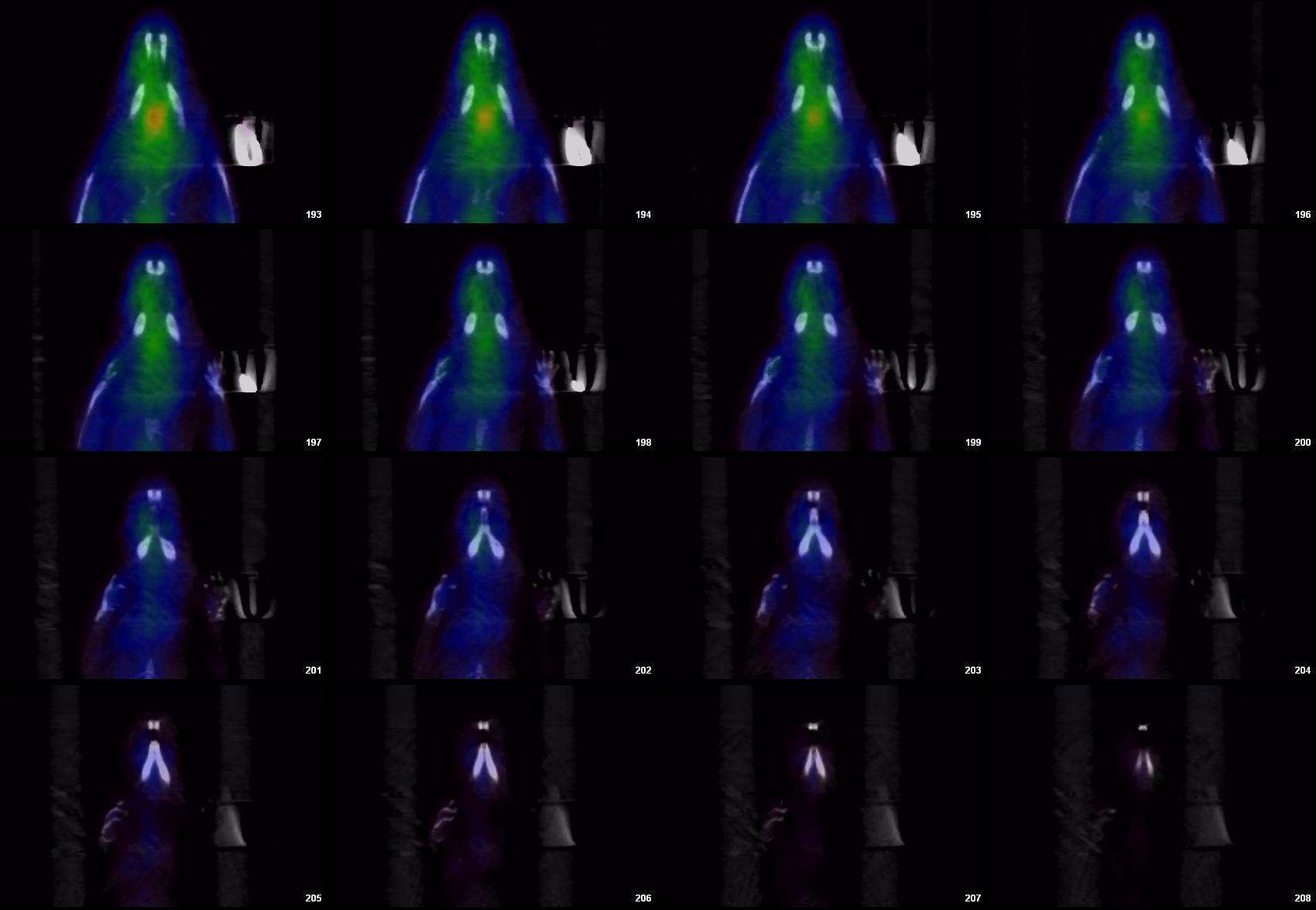

Supplement: Supplementary Figure 2 — Immunohistochemistry of hippocampal tissues from AD mice. (A) Histological changes in the hippocampus of 9-month-old WT and 3 × Tg-AD mice were analyzed by HE staining. (B–D) Analysis of neuronal status in the hippocampus of 9-month-old WT and 3 × Tg-AD mice by Nissler staining (n = 3 mice per group). (E–H) Analysis and quantification of Aβ deposition in hippocampal tissue of 9-month-old WT and 3 × Tg-AD mice by immunofluorescence (n = 4 mice per group). [file Data_Sheet_1.zip › FIG 2 pET-CT/PET-CT/M1/11.jpg]

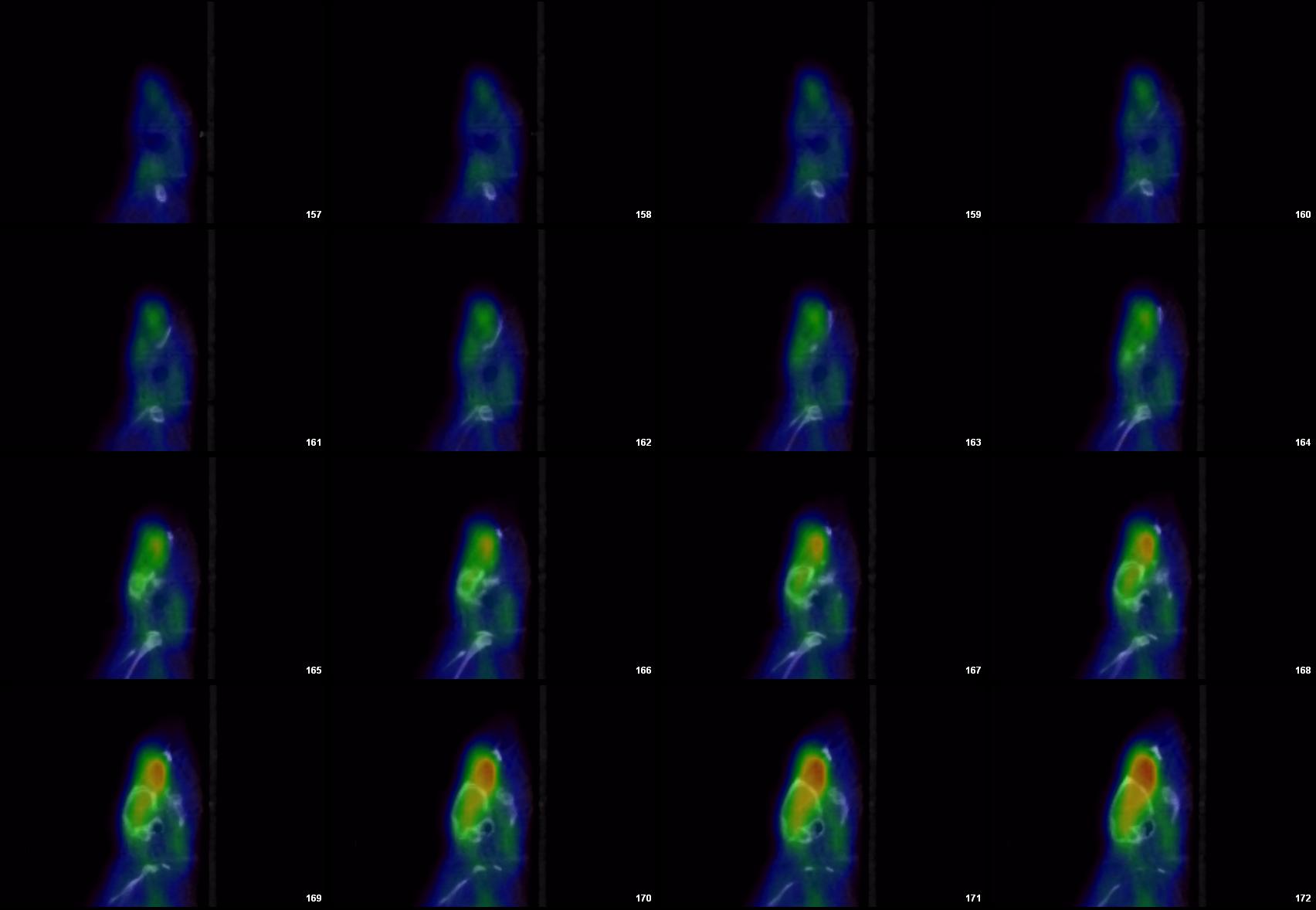

Supplement: Supplementary Figure 2 — Immunohistochemistry of hippocampal tissues from AD mice. (A) Histological changes in the hippocampus of 9-month-old WT and 3 × Tg-AD mice were analyzed by HE staining. (B–D) Analysis of neuronal status in the hippocampus of 9-month-old WT and 3 × Tg-AD mice by Nissler staining (n = 3 mice per group). (E–H) Analysis and quantification of Aβ deposition in hippocampal tissue of 9-month-old WT and 3 × Tg-AD mice by immunofluorescence (n = 4 mice per group). [file Data_Sheet_1.zip › FIG 2 pET-CT/PET-CT/M1/12.jpg]

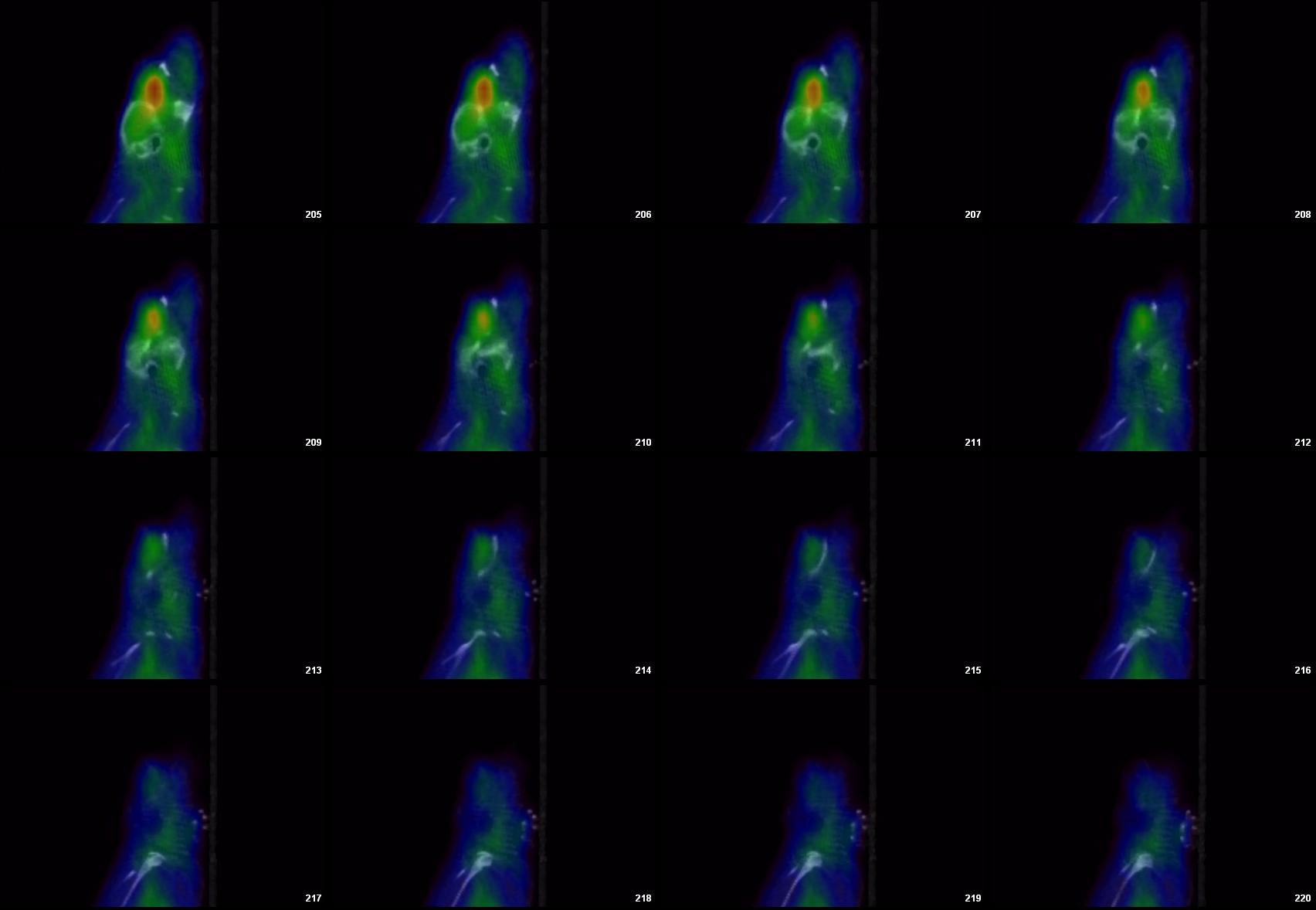

Supplement: Supplementary Figure 2 — Immunohistochemistry of hippocampal tissues from AD mice. (A) Histological changes in the hippocampus of 9-month-old WT and 3 × Tg-AD mice were analyzed by HE staining. (B–D) Analysis of neuronal status in the hippocampus of 9-month-old WT and 3 × Tg-AD mice by Nissler staining (n = 3 mice per group). (E–H) Analysis and quantification of Aβ deposition in hippocampal tissue of 9-month-old WT and 3 × Tg-AD mice by immunofluorescence (n = 4 mice per group). [file Data_Sheet_1.zip › FIG 2 pET-CT/PET-CT/M1/15.jpg]

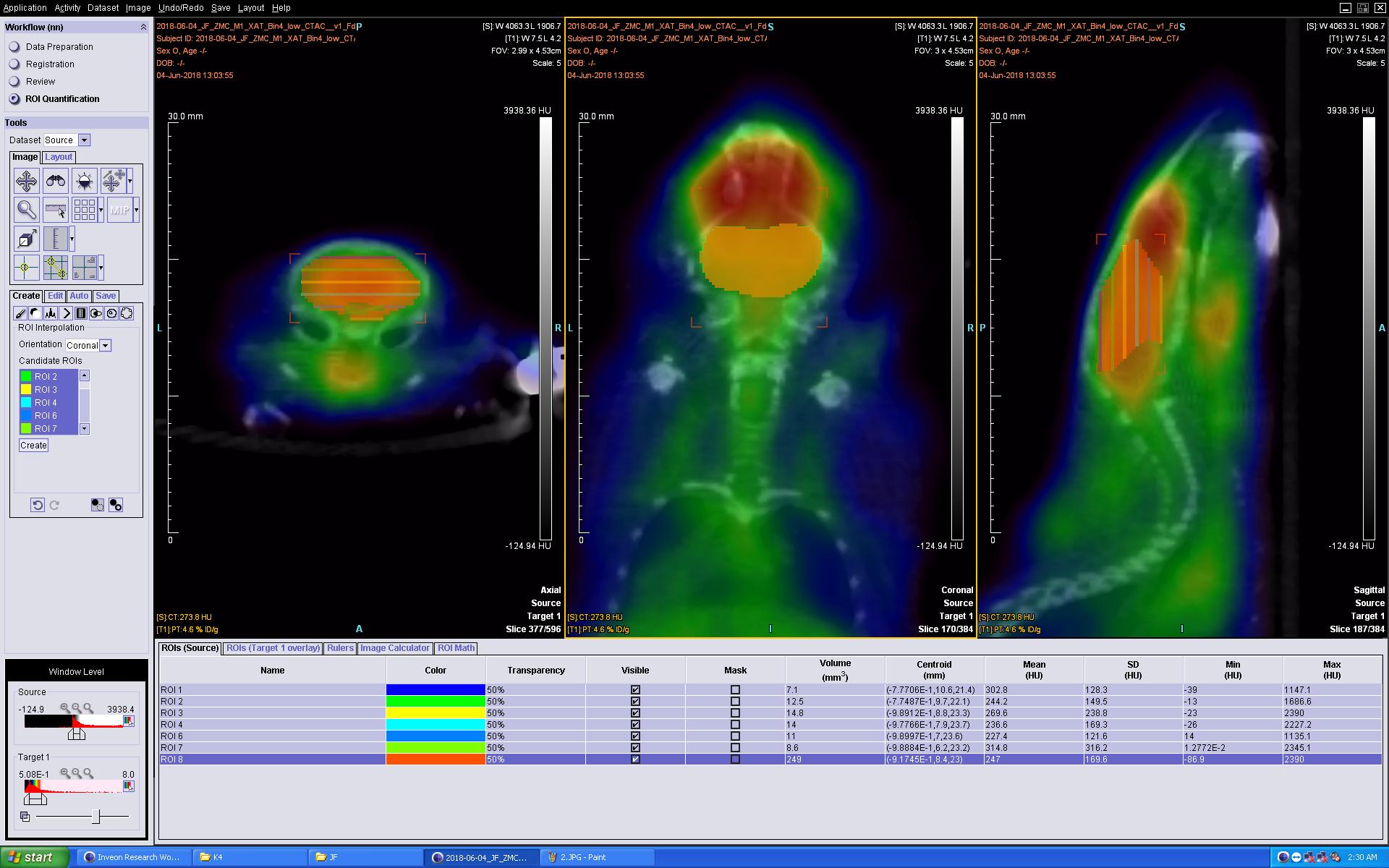

Supplement: Supplementary Figure 2 — Immunohistochemistry of hippocampal tissues from AD mice. (A) Histological changes in the hippocampus of 9-month-old WT and 3 × Tg-AD mice were analyzed by HE staining. (B–D) Analysis of neuronal status in the hippocampus of 9-month-old WT and 3 × Tg-AD mice by Nissler staining (n = 3 mice per group). (E–H) Analysis and quantification of Aβ deposition in hippocampal tissue of 9-month-old WT and 3 × Tg-AD mice by immunofluorescence (n = 4 mice per group). [file Data_Sheet_1.zip › FIG 2 pET-CT/PET-CT/M1/16.JPG]

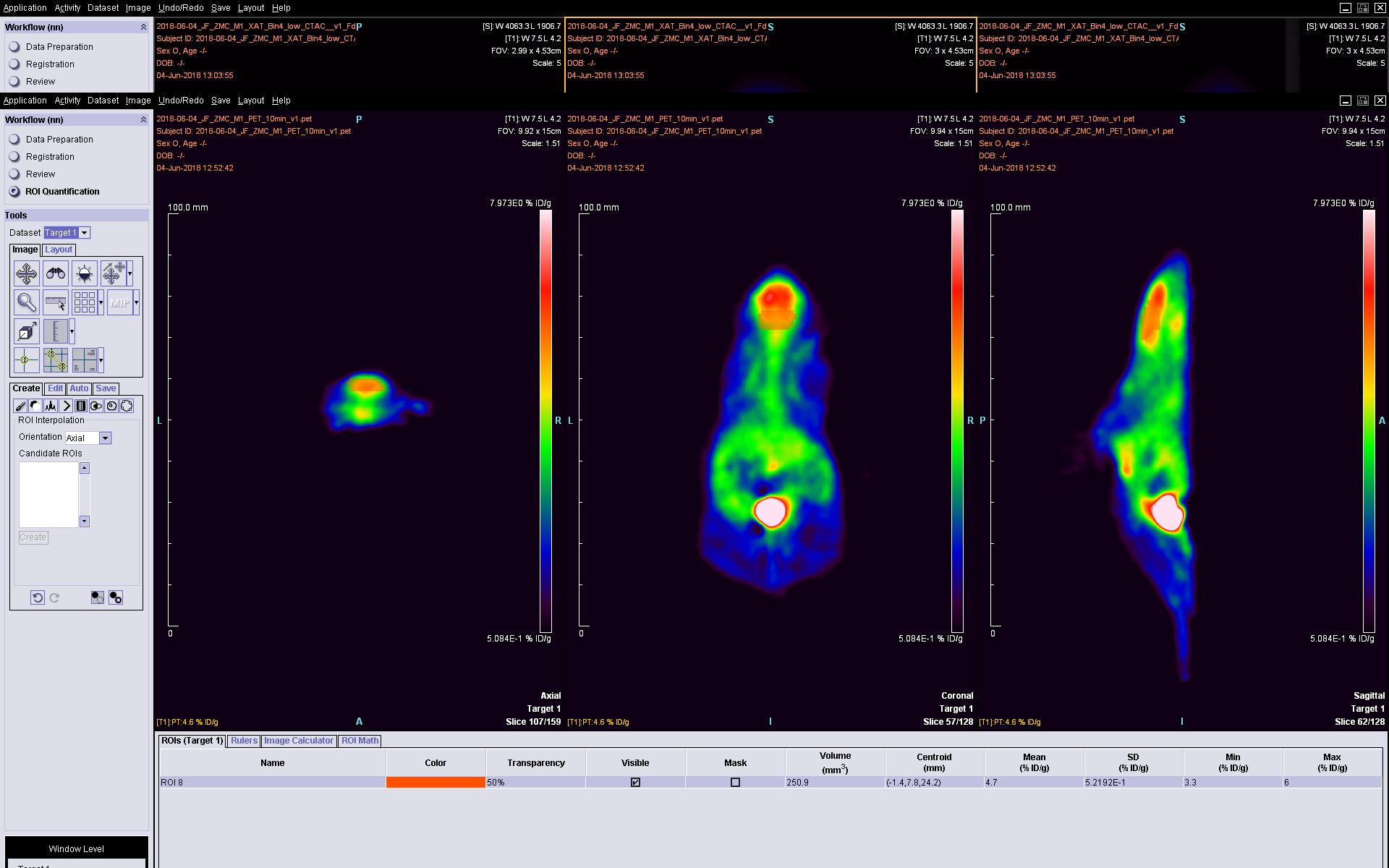

Supplement: Supplementary Figure 2 — Immunohistochemistry of hippocampal tissues from AD mice. (A) Histological changes in the hippocampus of 9-month-old WT and 3 × Tg-AD mice were analyzed by HE staining. (B–D) Analysis of neuronal status in the hippocampus of 9-month-old WT and 3 × Tg-AD mice by Nissler staining (n = 3 mice per group). (E–H) Analysis and quantification of Aβ deposition in hippocampal tissue of 9-month-old WT and 3 × Tg-AD mice by immunofluorescence (n = 4 mice per group). [file Data_Sheet_1.zip › FIG 2 pET-CT/PET-CT/M1/17.JPG]

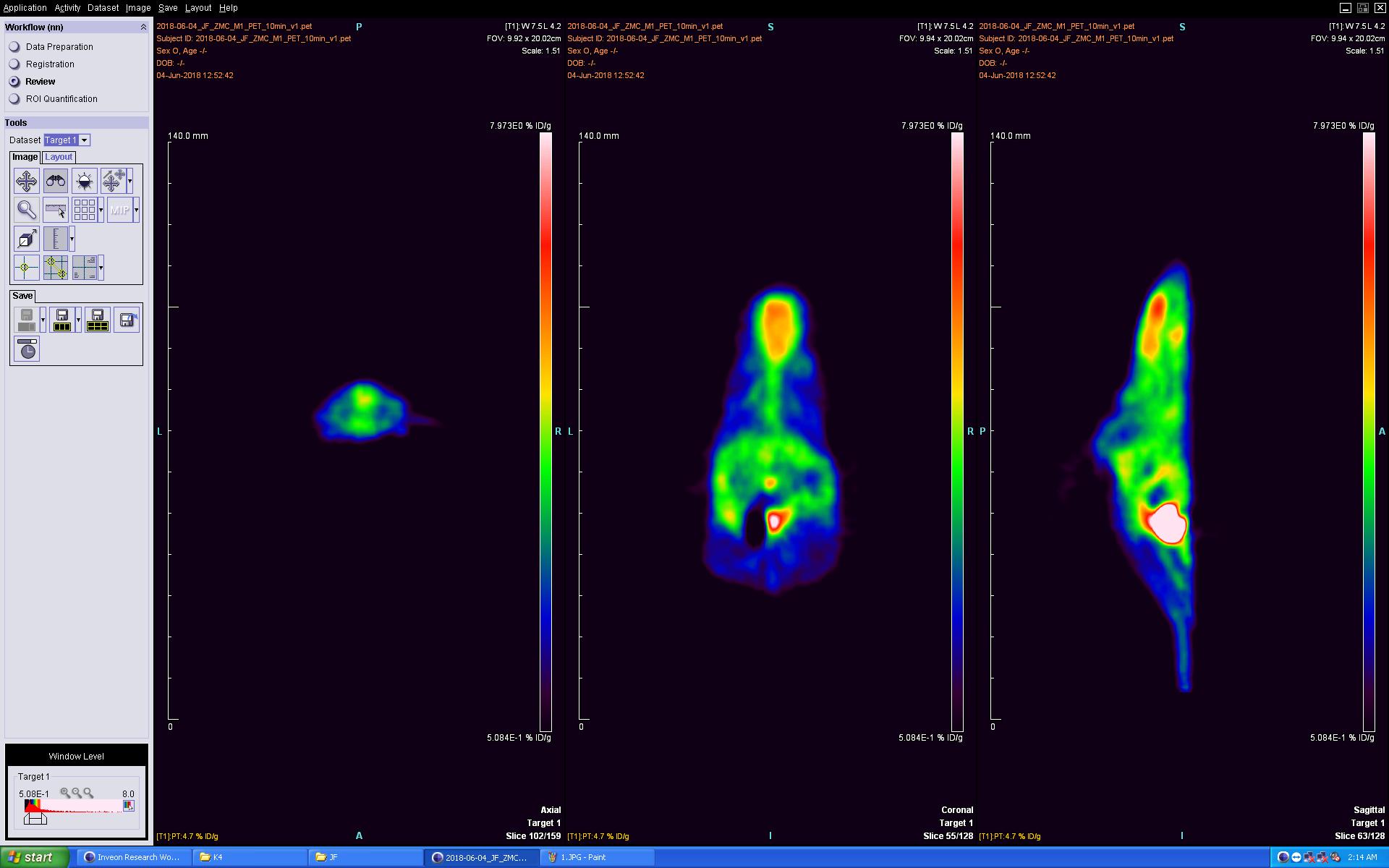

Supplement: Supplementary Figure 2 — Immunohistochemistry of hippocampal tissues from AD mice. (A) Histological changes in the hippocampus of 9-month-old WT and 3 × Tg-AD mice were analyzed by HE staining. (B–D) Analysis of neuronal status in the hippocampus of 9-month-old WT and 3 × Tg-AD mice by Nissler staining (n = 3 mice per group). (E–H) Analysis and quantification of Aβ deposition in hippocampal tissue of 9-month-old WT and 3 × Tg-AD mice by immunofluorescence (n = 4 mice per group). [file Data_Sheet_1.zip › FIG 2 pET-CT/PET-CT/M1/2.JPG]

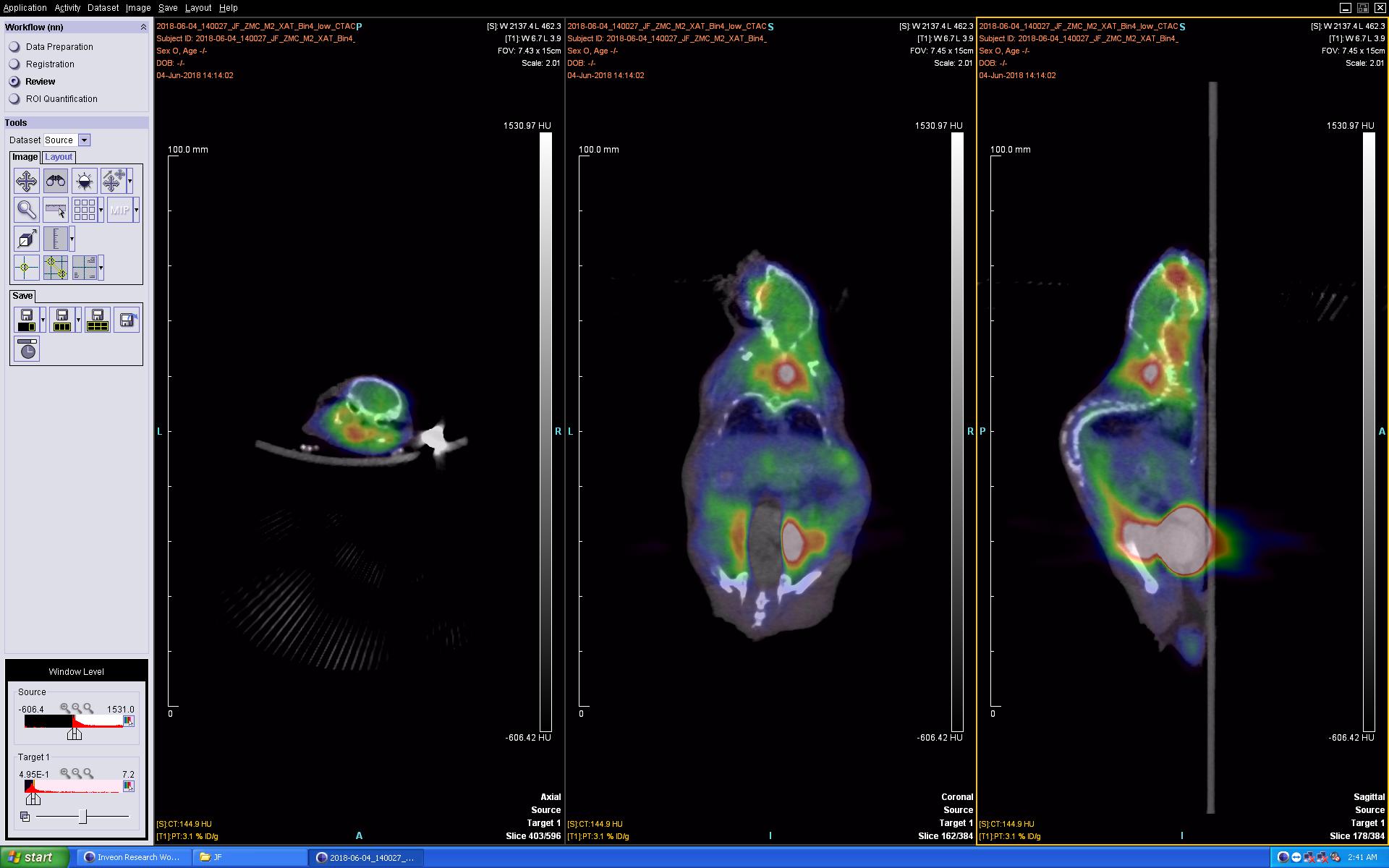

Supplement: Supplementary Figure 2 — Immunohistochemistry of hippocampal tissues from AD mice. (A) Histological changes in the hippocampus of 9-month-old WT and 3 × Tg-AD mice were analyzed by HE staining. (B–D) Analysis of neuronal status in the hippocampus of 9-month-old WT and 3 × Tg-AD mice by Nissler staining (n = 3 mice per group). (E–H) Analysis and quantification of Aβ deposition in hippocampal tissue of 9-month-old WT and 3 × Tg-AD mice by immunofluorescence (n = 4 mice per group). [file Data_Sheet_1.zip › FIG 2 pET-CT/PET-CT/M2/1.JPG]

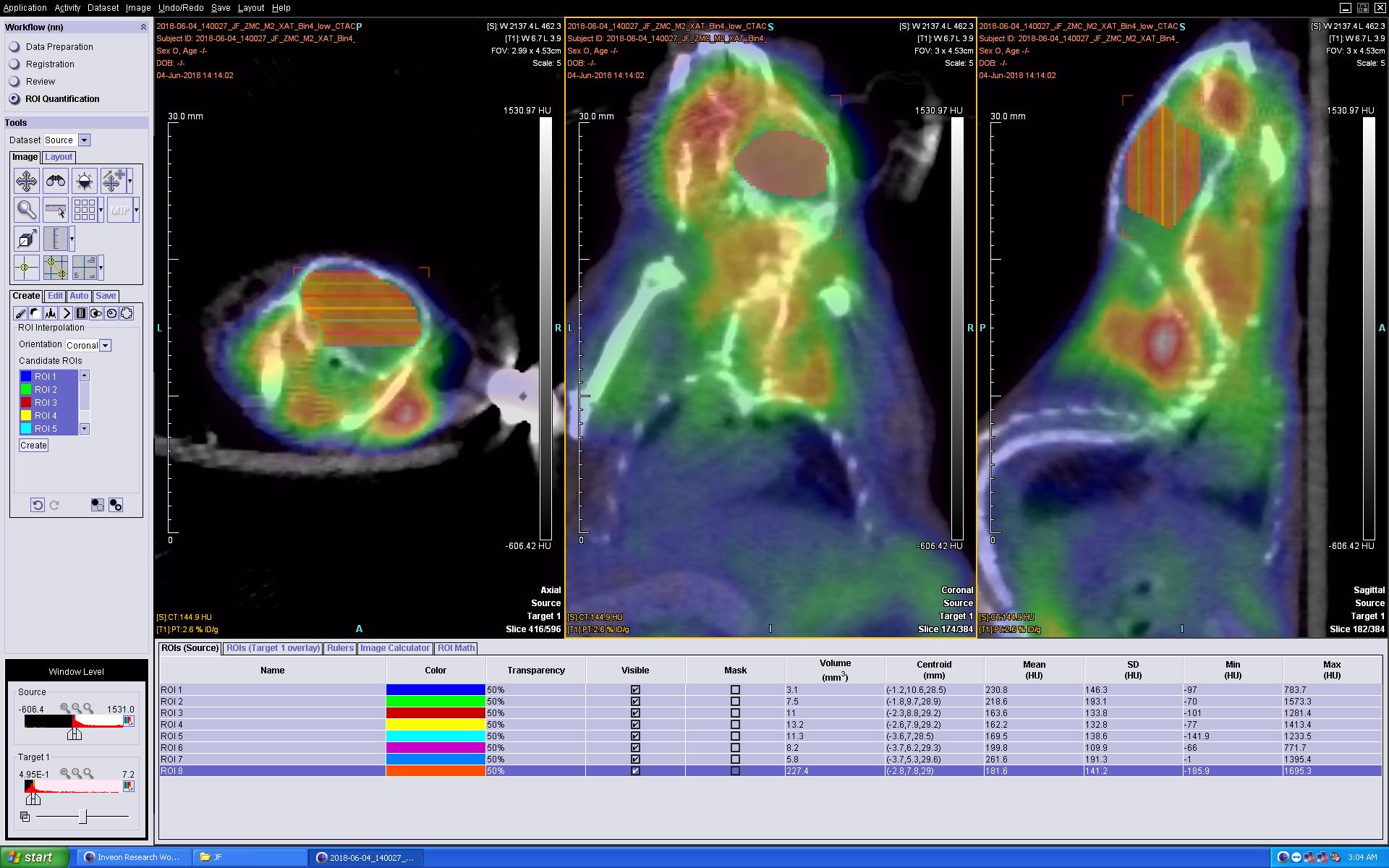

Supplement: Supplementary Figure 2 — Immunohistochemistry of hippocampal tissues from AD mice. (A) Histological changes in the hippocampus of 9-month-old WT and 3 × Tg-AD mice were analyzed by HE staining. (B–D) Analysis of neuronal status in the hippocampus of 9-month-old WT and 3 × Tg-AD mice by Nissler staining (n = 3 mice per group). (E–H) Analysis and quantification of Aβ deposition in hippocampal tissue of 9-month-old WT and 3 × Tg-AD mice by immunofluorescence (n = 4 mice per group). [file Data_Sheet_1.zip › FIG 2 pET-CT/PET-CT/M2/12.JPG]

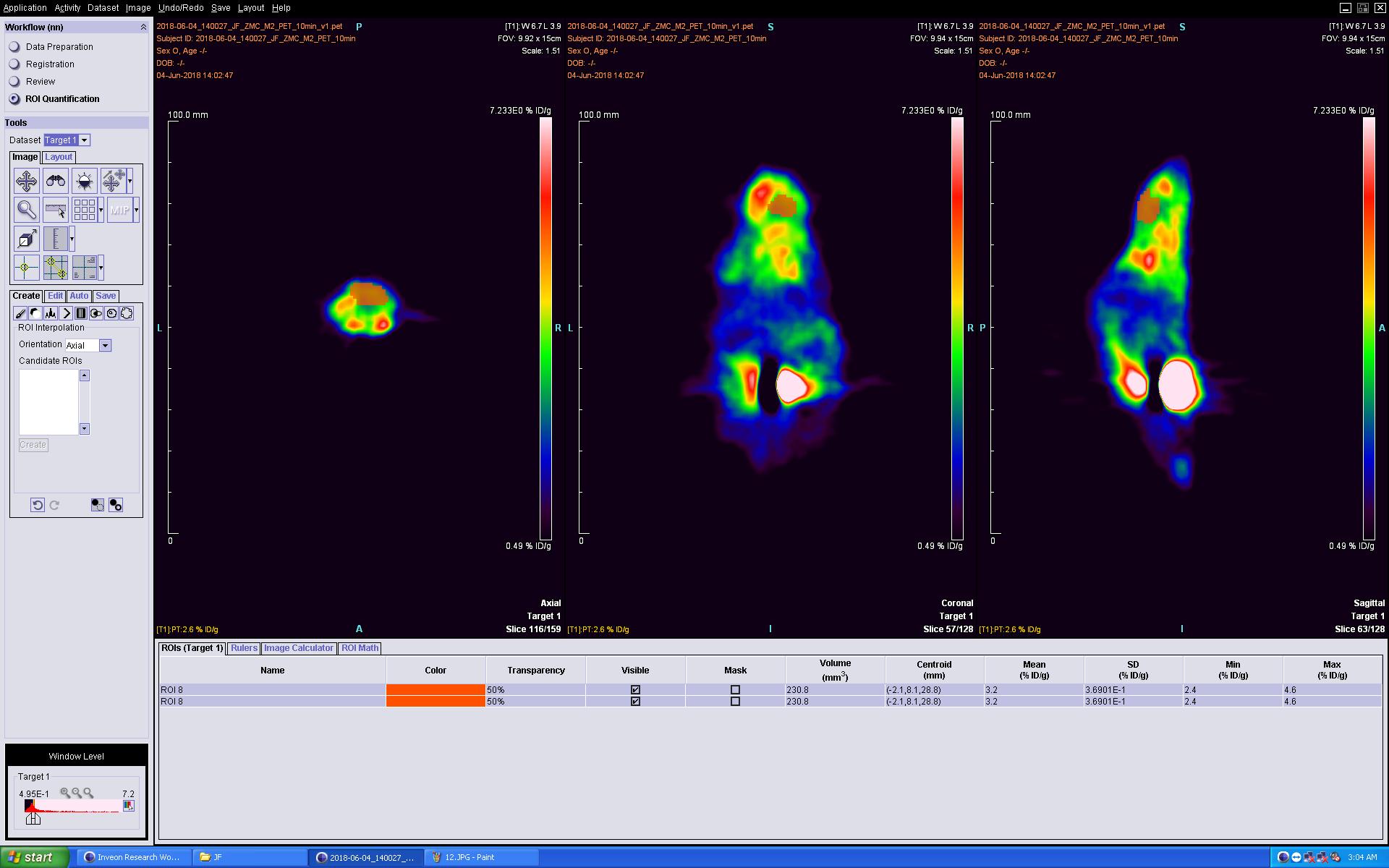

Supplement: Supplementary Figure 2 — Immunohistochemistry of hippocampal tissues from AD mice. (A) Histological changes in the hippocampus of 9-month-old WT and 3 × Tg-AD mice were analyzed by HE staining. (B–D) Analysis of neuronal status in the hippocampus of 9-month-old WT and 3 × Tg-AD mice by Nissler staining (n = 3 mice per group). (E–H) Analysis and quantification of Aβ deposition in hippocampal tissue of 9-month-old WT and 3 × Tg-AD mice by immunofluorescence (n = 4 mice per group). [file Data_Sheet_1.zip › FIG 2 pET-CT/PET-CT/M2/13.JPG]

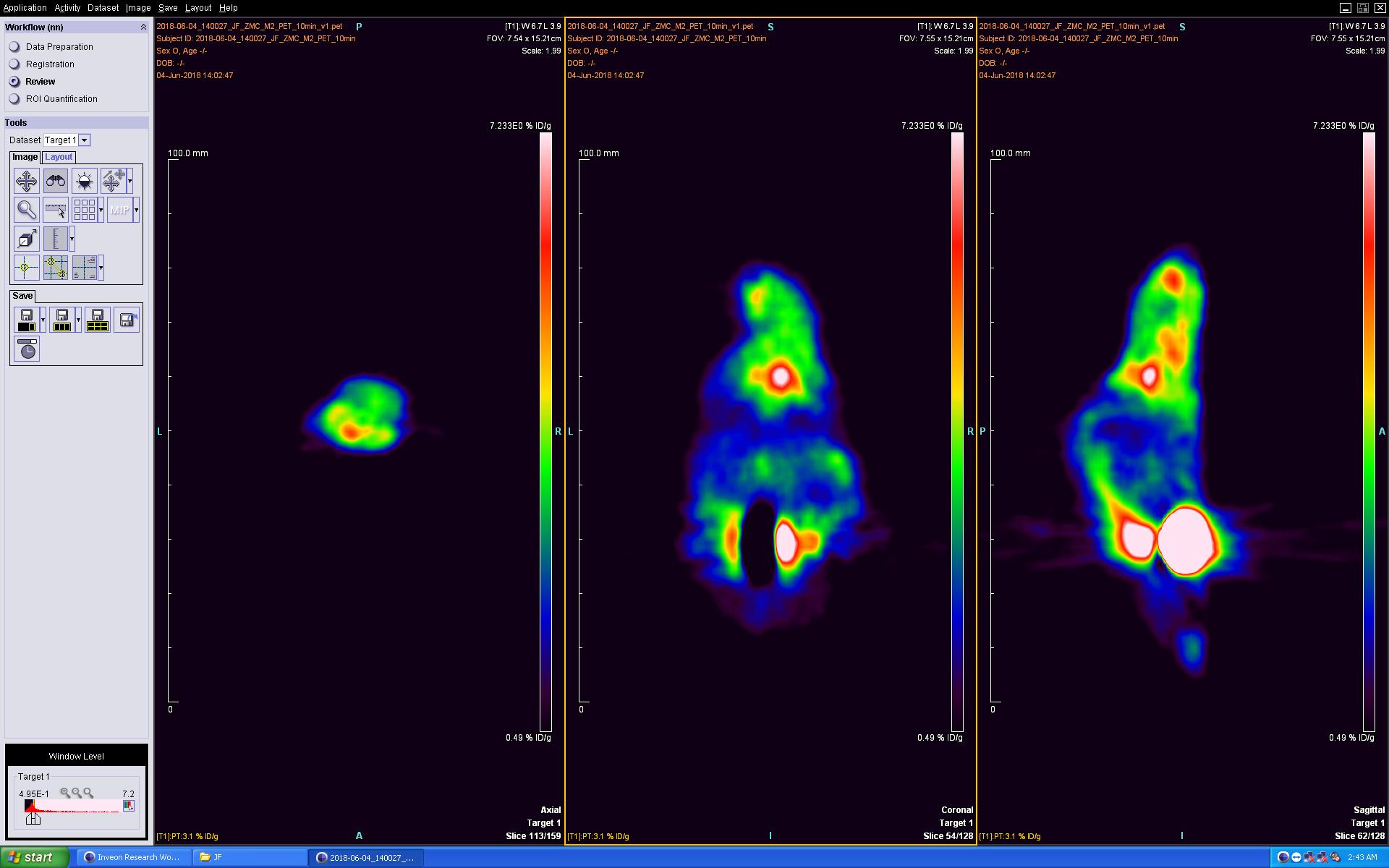

Supplement: Supplementary Figure 2 — Immunohistochemistry of hippocampal tissues from AD mice. (A) Histological changes in the hippocampus of 9-month-old WT and 3 × Tg-AD mice were analyzed by HE staining. (B–D) Analysis of neuronal status in the hippocampus of 9-month-old WT and 3 × Tg-AD mice by Nissler staining (n = 3 mice per group). (E–H) Analysis and quantification of Aβ deposition in hippocampal tissue of 9-month-old WT and 3 × Tg-AD mice by immunofluorescence (n = 4 mice per group). [file Data_Sheet_1.zip › FIG 2 pET-CT/PET-CT/M2/2.JPG]

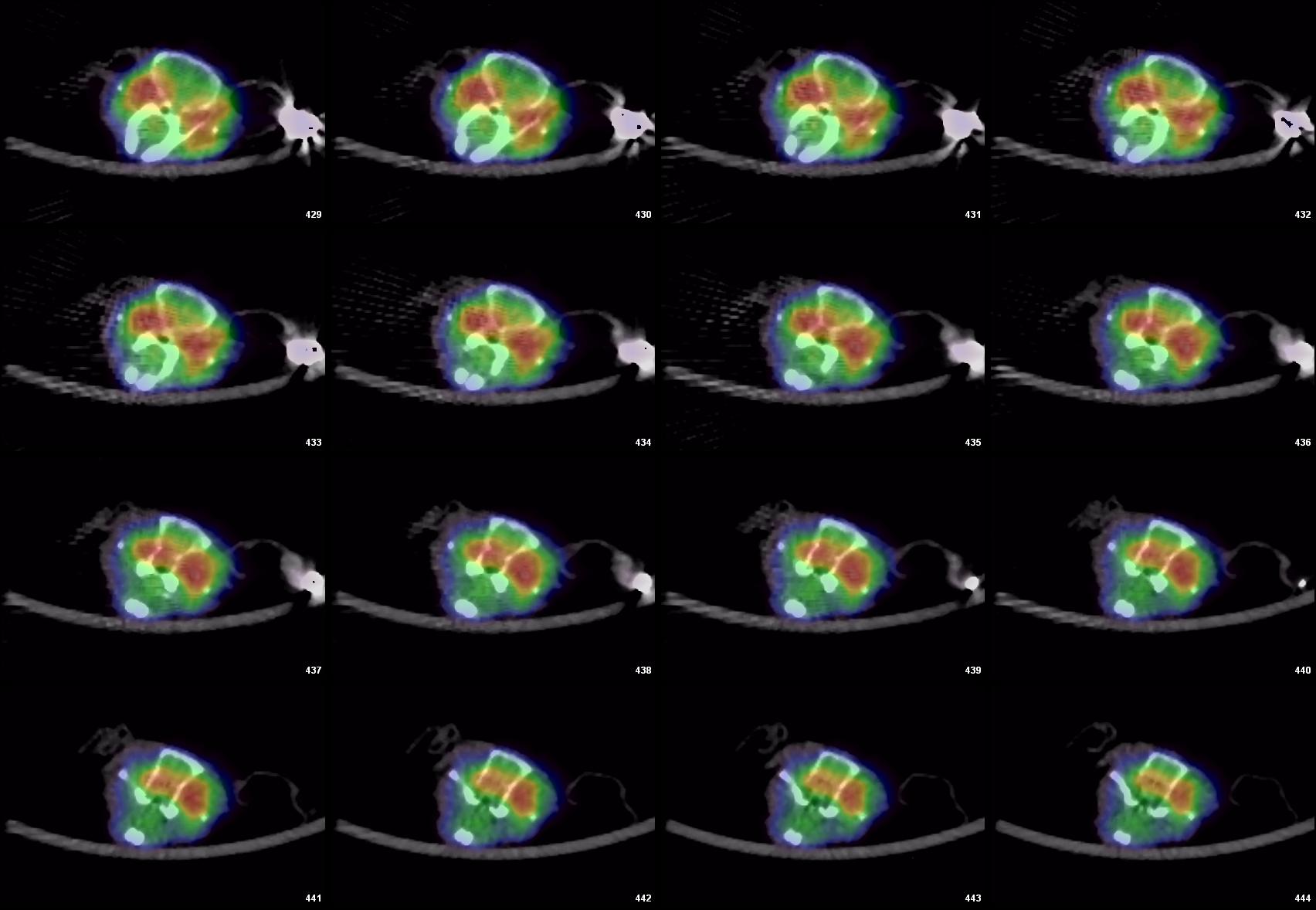

Supplement: Supplementary Figure 2 — Immunohistochemistry of hippocampal tissues from AD mice. (A) Histological changes in the hippocampus of 9-month-old WT and 3 × Tg-AD mice were analyzed by HE staining. (B–D) Analysis of neuronal status in the hippocampus of 9-month-old WT and 3 × Tg-AD mice by Nissler staining (n = 3 mice per group). (E–H) Analysis and quantification of Aβ deposition in hippocampal tissue of 9-month-old WT and 3 × Tg-AD mice by immunofluorescence (n = 4 mice per group). [file Data_Sheet_1.zip › FIG 2 pET-CT/PET-CT/M2/6.jpg]

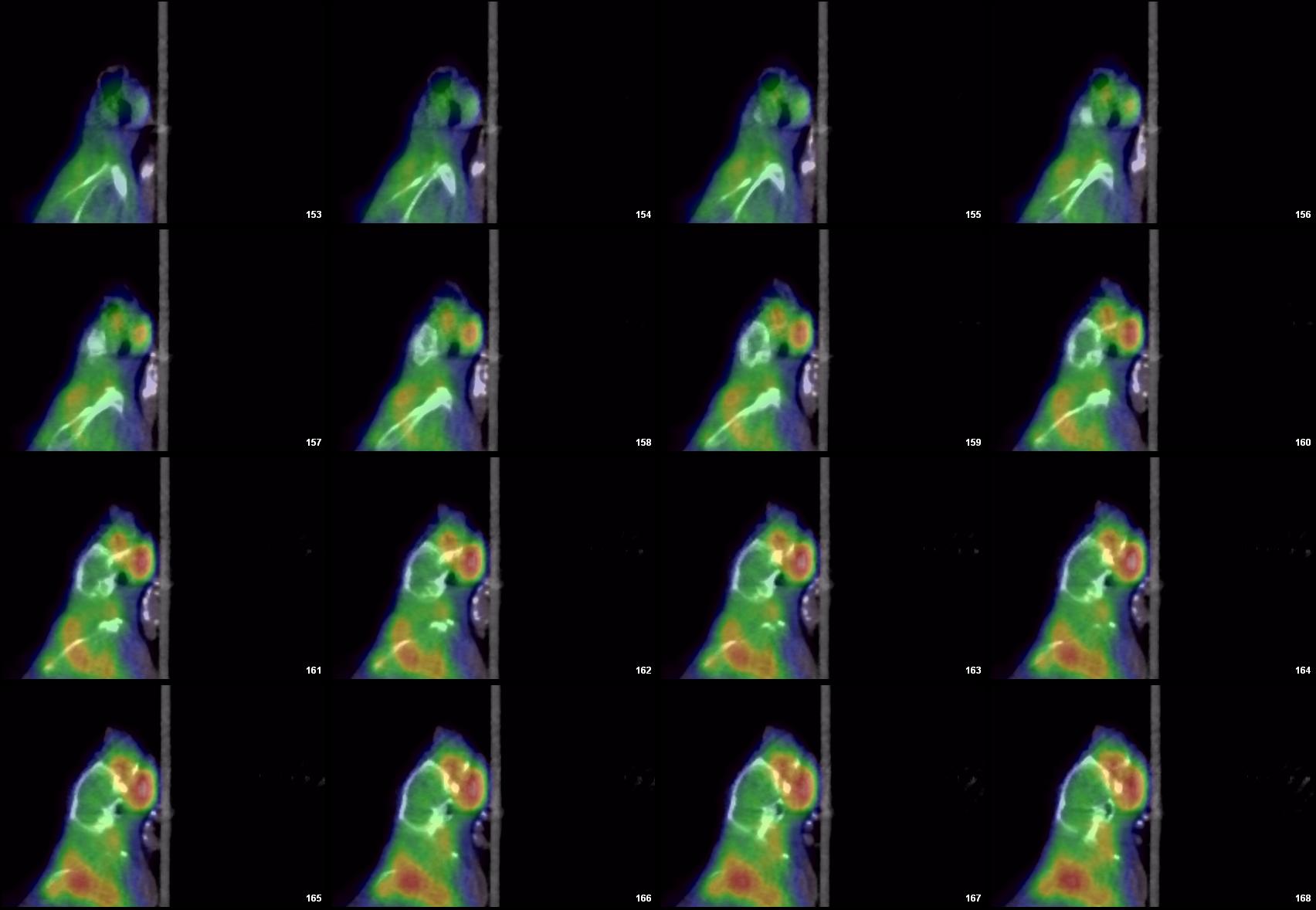

Supplement: Supplementary Figure 2 — Immunohistochemistry of hippocampal tissues from AD mice. (A) Histological changes in the hippocampus of 9-month-old WT and 3 × Tg-AD mice were analyzed by HE staining. (B–D) Analysis of neuronal status in the hippocampus of 9-month-old WT and 3 × Tg-AD mice by Nissler staining (n = 3 mice per group). (E–H) Analysis and quantification of Aβ deposition in hippocampal tissue of 9-month-old WT and 3 × Tg-AD mice by immunofluorescence (n = 4 mice per group). [file Data_Sheet_1.zip › FIG 2 pET-CT/PET-CT/M2/9.jpg]

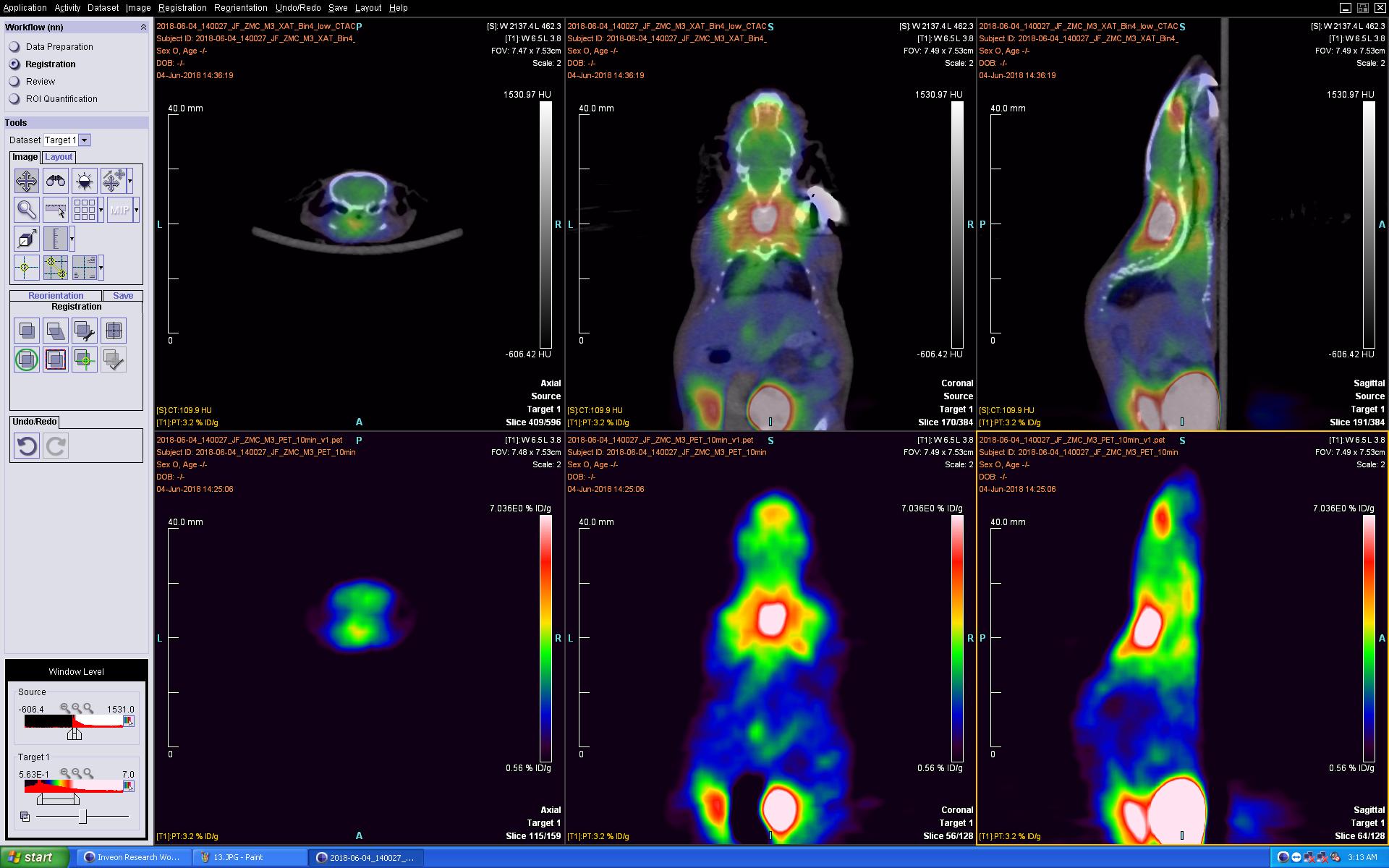

Supplement: Supplementary Figure 2 — Immunohistochemistry of hippocampal tissues from AD mice. (A) Histological changes in the hippocampus of 9-month-old WT and 3 × Tg-AD mice were analyzed by HE staining. (B–D) Analysis of neuronal status in the hippocampus of 9-month-old WT and 3 × Tg-AD mice by Nissler staining (n = 3 mice per group). (E–H) Analysis and quantification of Aβ deposition in hippocampal tissue of 9-month-old WT and 3 × Tg-AD mice by immunofluorescence (n = 4 mice per group). [file Data_Sheet_1.zip › FIG 2 pET-CT/PET-CT/M3/1.JPG]

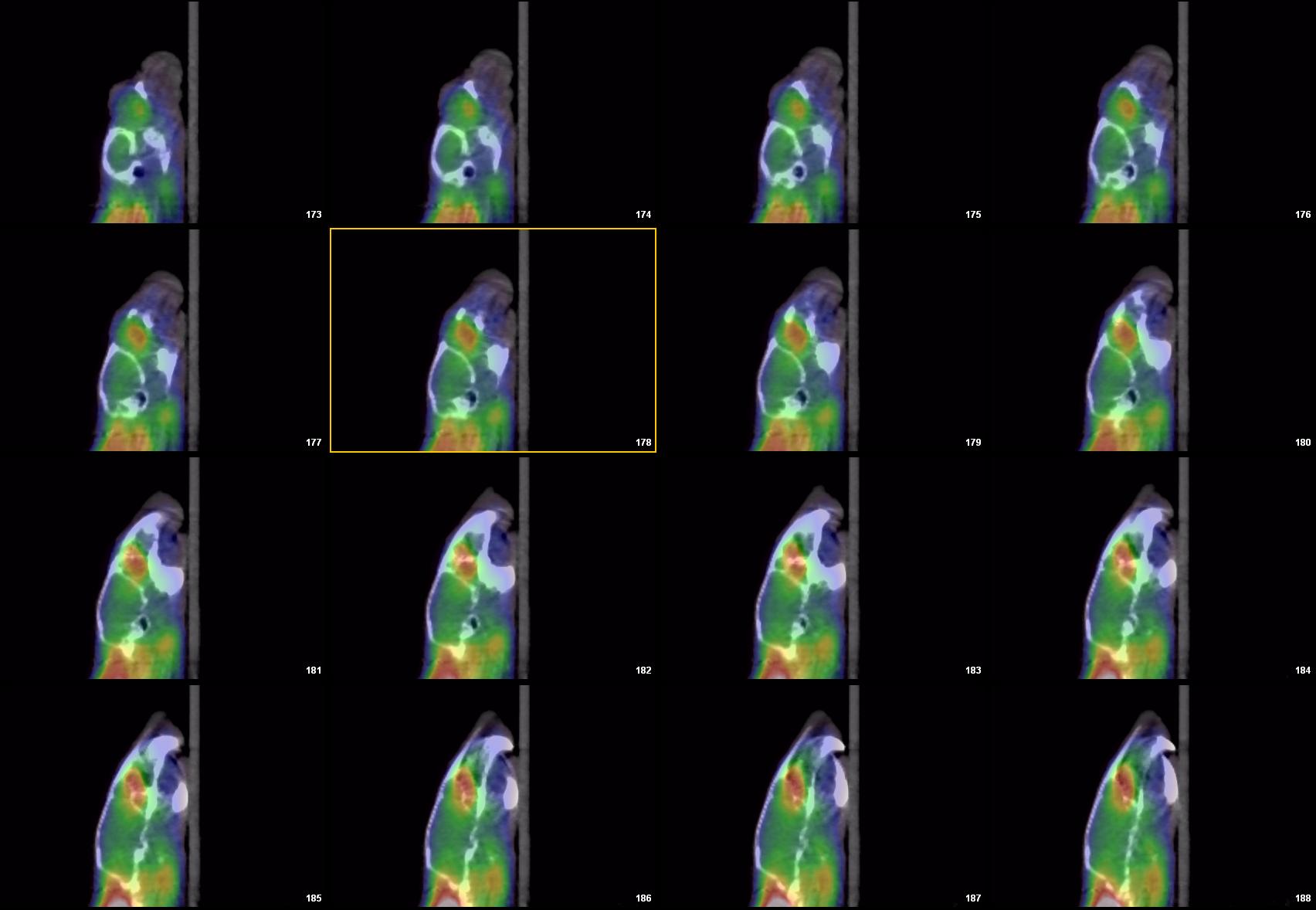

Supplement: Supplementary Figure 2 — Immunohistochemistry of hippocampal tissues from AD mice. (A) Histological changes in the hippocampus of 9-month-old WT and 3 × Tg-AD mice were analyzed by HE staining. (B–D) Analysis of neuronal status in the hippocampus of 9-month-old WT and 3 × Tg-AD mice by Nissler staining (n = 3 mice per group). (E–H) Analysis and quantification of Aβ deposition in hippocampal tissue of 9-month-old WT and 3 × Tg-AD mice by immunofluorescence (n = 4 mice per group). [file Data_Sheet_1.zip › FIG 2 pET-CT/PET-CT/M3/10.jpg]

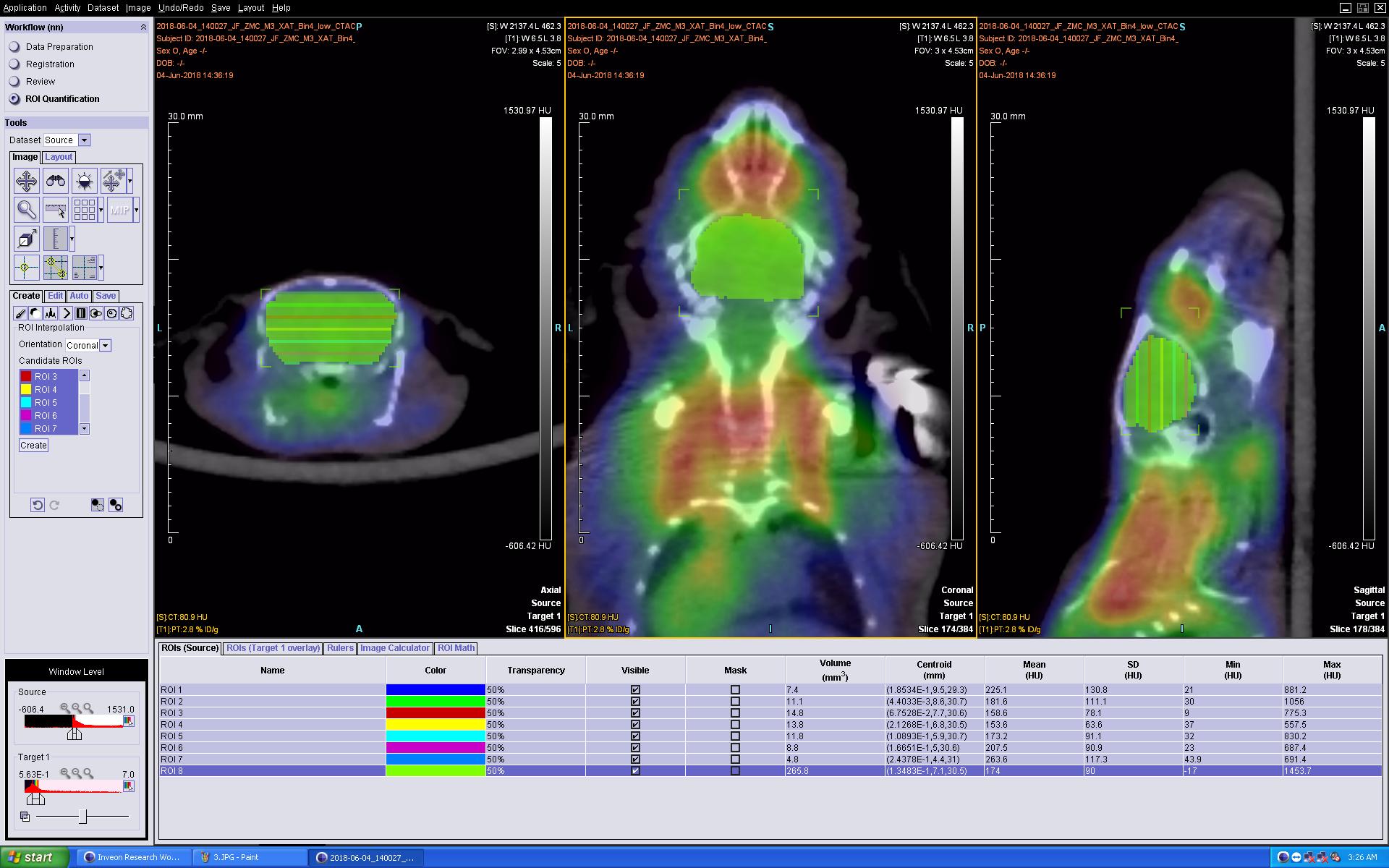

Supplement: Supplementary Figure 2 — Immunohistochemistry of hippocampal tissues from AD mice. (A) Histological changes in the hippocampus of 9-month-old WT and 3 × Tg-AD mice were analyzed by HE staining. (B–D) Analysis of neuronal status in the hippocampus of 9-month-old WT and 3 × Tg-AD mice by Nissler staining (n = 3 mice per group). (E–H) Analysis and quantification of Aβ deposition in hippocampal tissue of 9-month-old WT and 3 × Tg-AD mice by immunofluorescence (n = 4 mice per group). [file Data_Sheet_1.zip › FIG 2 pET-CT/PET-CT/M3/13.JPG]

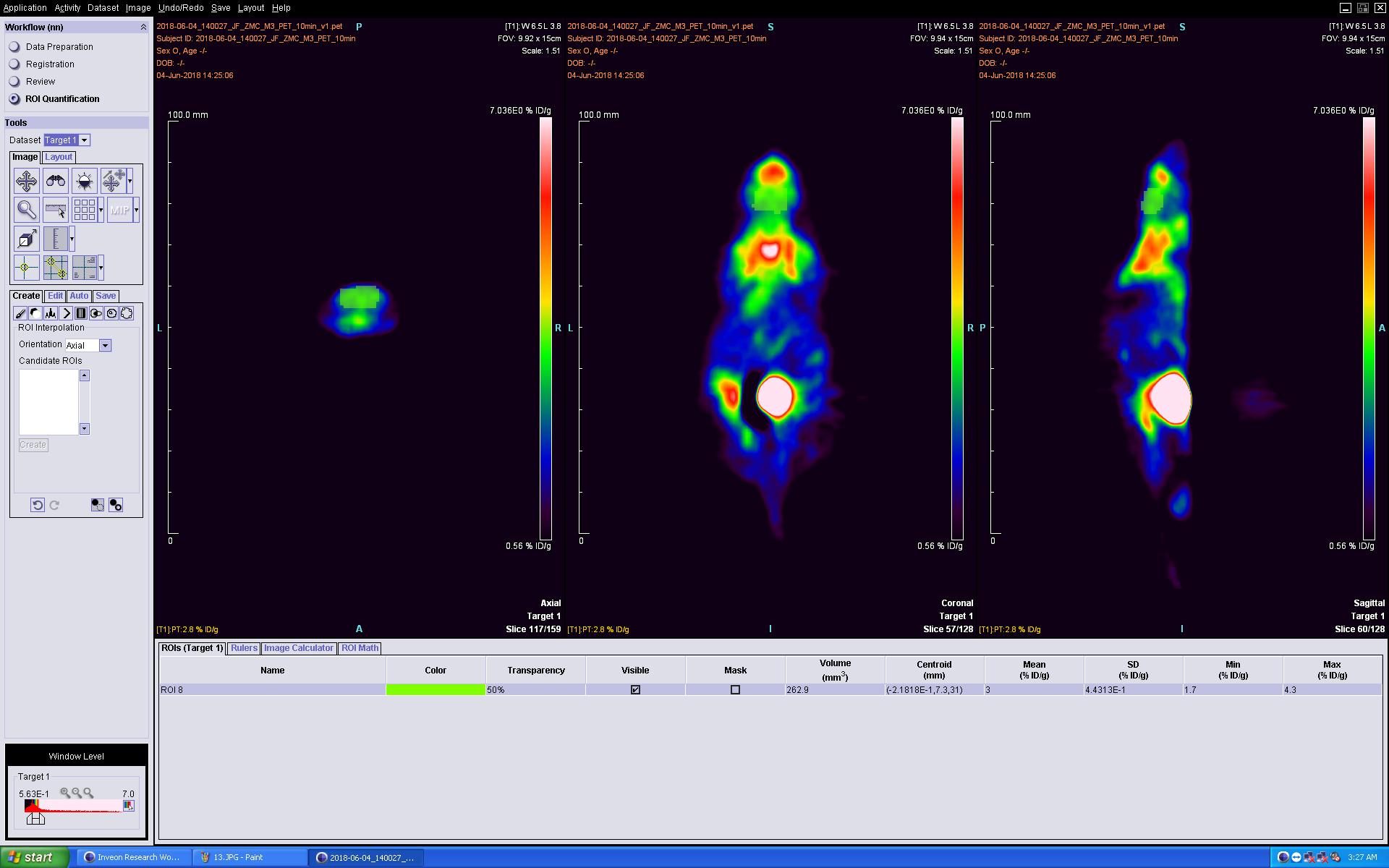

Supplement: Supplementary Figure 2 — Immunohistochemistry of hippocampal tissues from AD mice. (A) Histological changes in the hippocampus of 9-month-old WT and 3 × Tg-AD mice were analyzed by HE staining. (B–D) Analysis of neuronal status in the hippocampus of 9-month-old WT and 3 × Tg-AD mice by Nissler staining (n = 3 mice per group). (E–H) Analysis and quantification of Aβ deposition in hippocampal tissue of 9-month-old WT and 3 × Tg-AD mice by immunofluorescence (n = 4 mice per group). [file Data_Sheet_1.zip › FIG 2 pET-CT/PET-CT/M3/14.JPG]

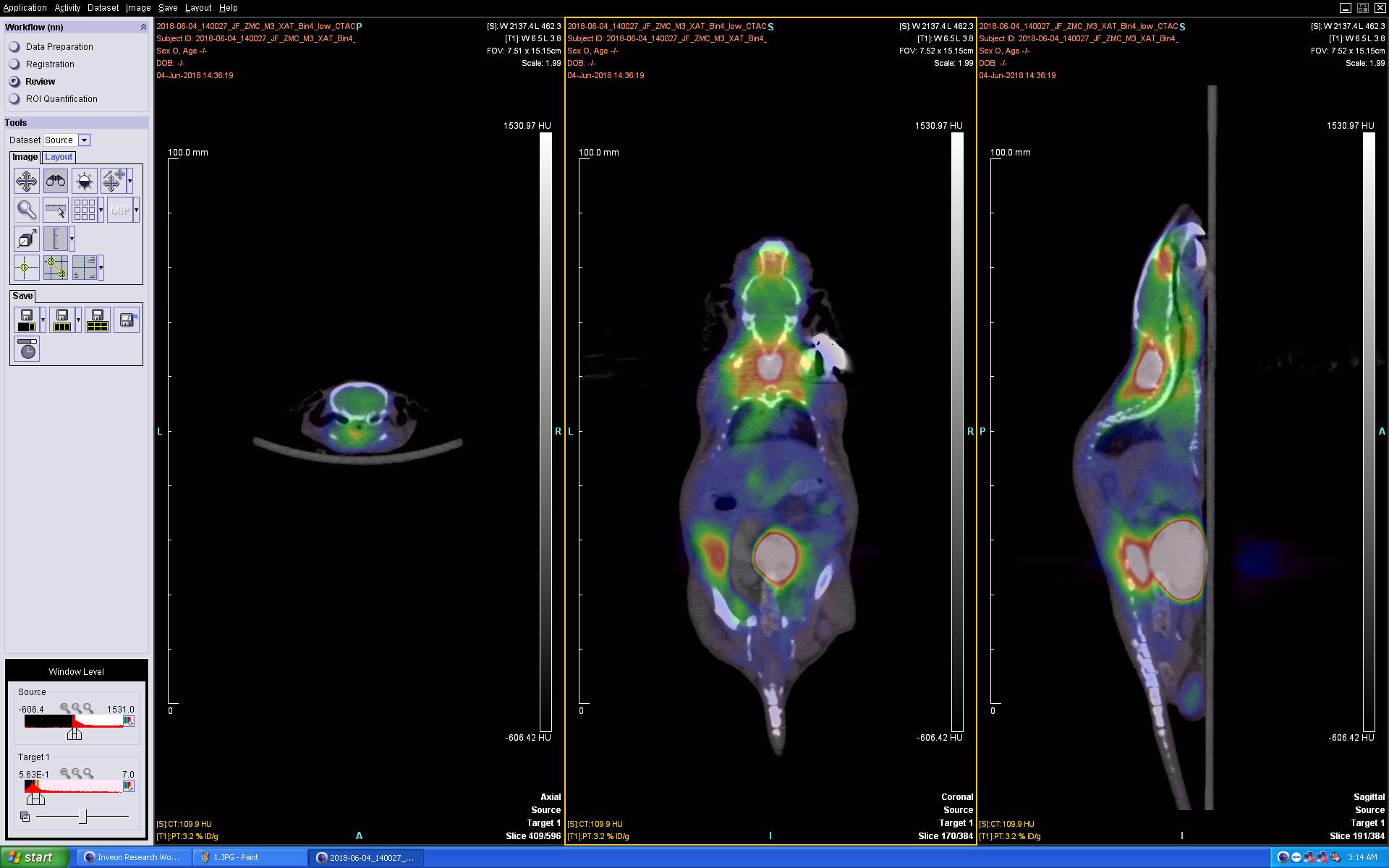

Supplement: Supplementary Figure 2 — Immunohistochemistry of hippocampal tissues from AD mice. (A) Histological changes in the hippocampus of 9-month-old WT and 3 × Tg-AD mice were analyzed by HE staining. (B–D) Analysis of neuronal status in the hippocampus of 9-month-old WT and 3 × Tg-AD mice by Nissler staining (n = 3 mice per group). (E–H) Analysis and quantification of Aβ deposition in hippocampal tissue of 9-month-old WT and 3 × Tg-AD mice by immunofluorescence (n = 4 mice per group). [file Data_Sheet_1.zip › FIG 2 pET-CT/PET-CT/M3/2.JPG]

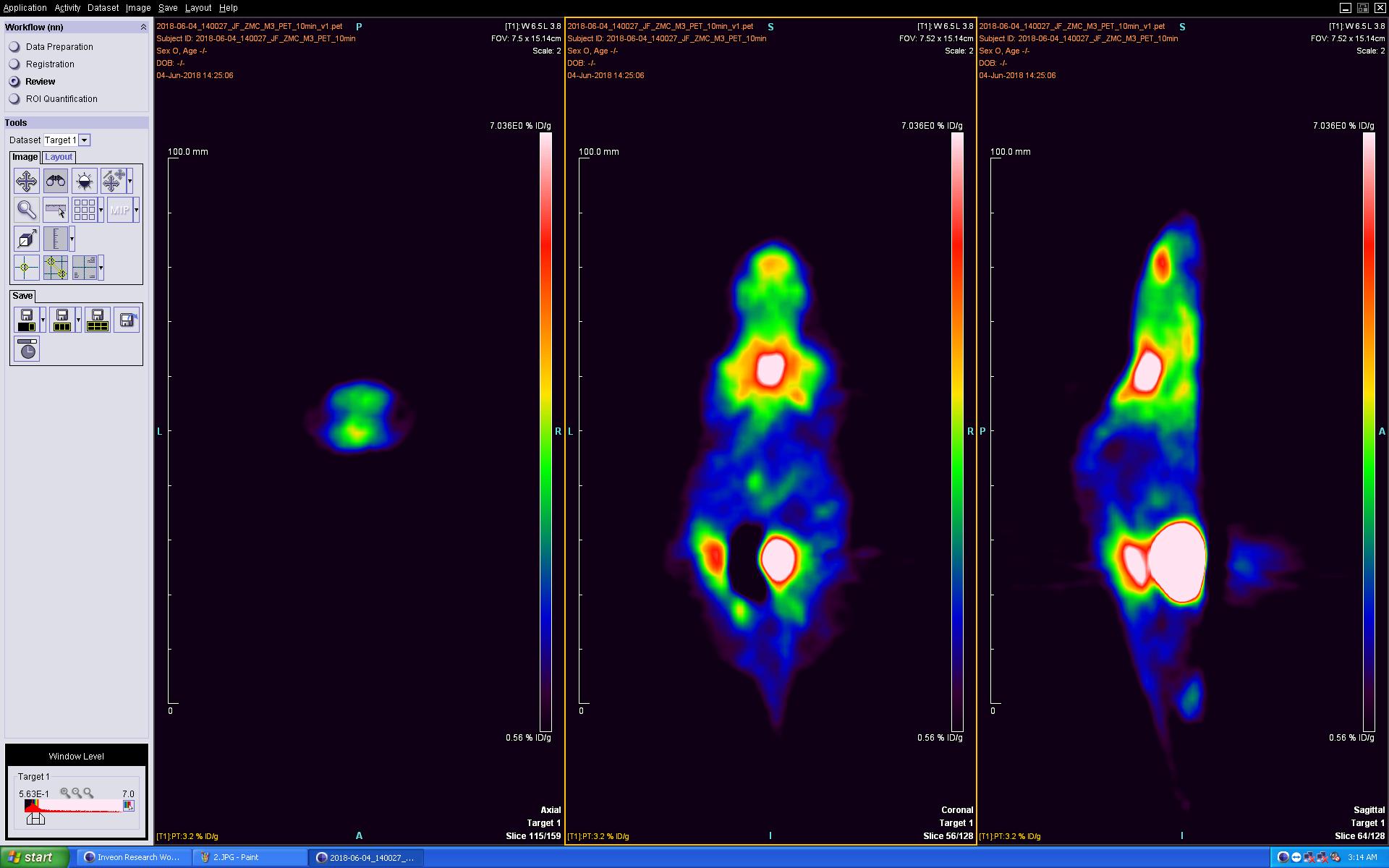

Supplement: Supplementary Figure 2 — Immunohistochemistry of hippocampal tissues from AD mice. (A) Histological changes in the hippocampus of 9-month-old WT and 3 × Tg-AD mice were analyzed by HE staining. (B–D) Analysis of neuronal status in the hippocampus of 9-month-old WT and 3 × Tg-AD mice by Nissler staining (n = 3 mice per group). (E–H) Analysis and quantification of Aβ deposition in hippocampal tissue of 9-month-old WT and 3 × Tg-AD mice by immunofluorescence (n = 4 mice per group). [file Data_Sheet_1.zip › FIG 2 pET-CT/PET-CT/M3/3.JPG]

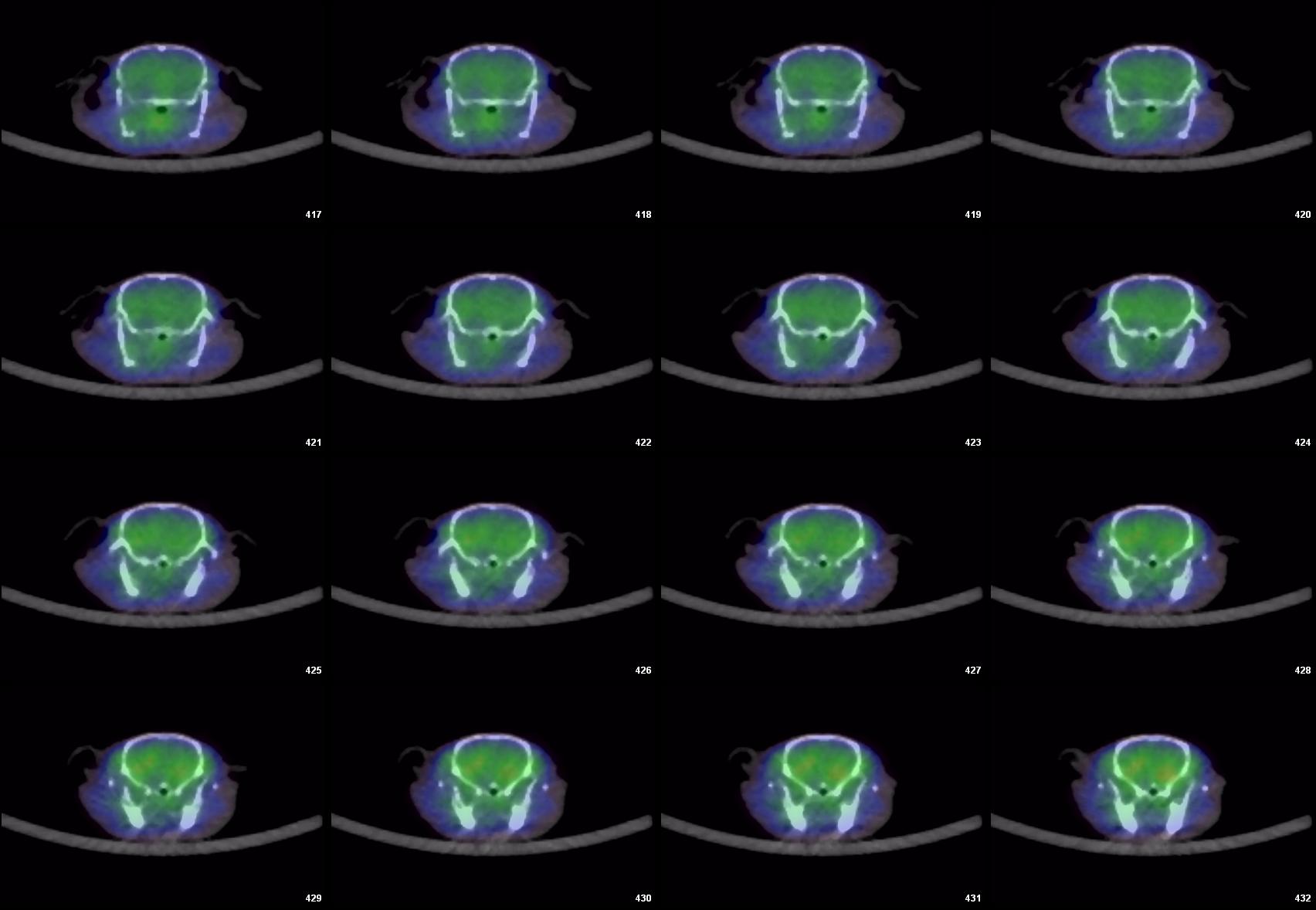

Supplement: Supplementary Figure 2 — Immunohistochemistry of hippocampal tissues from AD mice. (A) Histological changes in the hippocampus of 9-month-old WT and 3 × Tg-AD mice were analyzed by HE staining. (B–D) Analysis of neuronal status in the hippocampus of 9-month-old WT and 3 × Tg-AD mice by Nissler staining (n = 3 mice per group). (E–H) Analysis and quantification of Aβ deposition in hippocampal tissue of 9-month-old WT and 3 × Tg-AD mice by immunofluorescence (n = 4 mice per group). [file Data_Sheet_1.zip › FIG 2 pET-CT/PET-CT/M3/6.jpg]

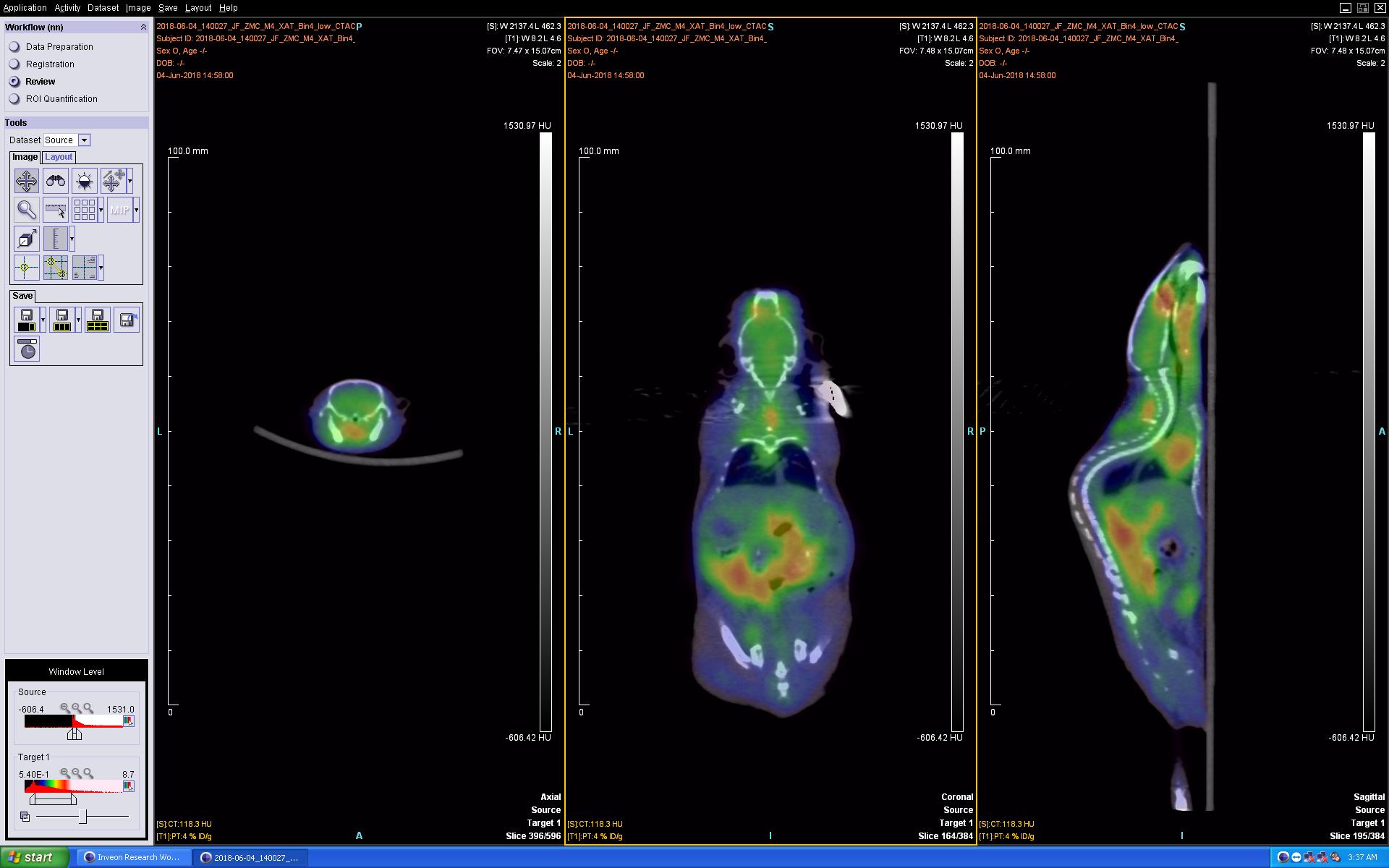

Supplement: Supplementary Figure 2 — Immunohistochemistry of hippocampal tissues from AD mice. (A) Histological changes in the hippocampus of 9-month-old WT and 3 × Tg-AD mice were analyzed by HE staining. (B–D) Analysis of neuronal status in the hippocampus of 9-month-old WT and 3 × Tg-AD mice by Nissler staining (n = 3 mice per group). (E–H) Analysis and quantification of Aβ deposition in hippocampal tissue of 9-month-old WT and 3 × Tg-AD mice by immunofluorescence (n = 4 mice per group). [file Data_Sheet_1.zip › FIG 2 pET-CT/PET-CT/M4/1.JPG]

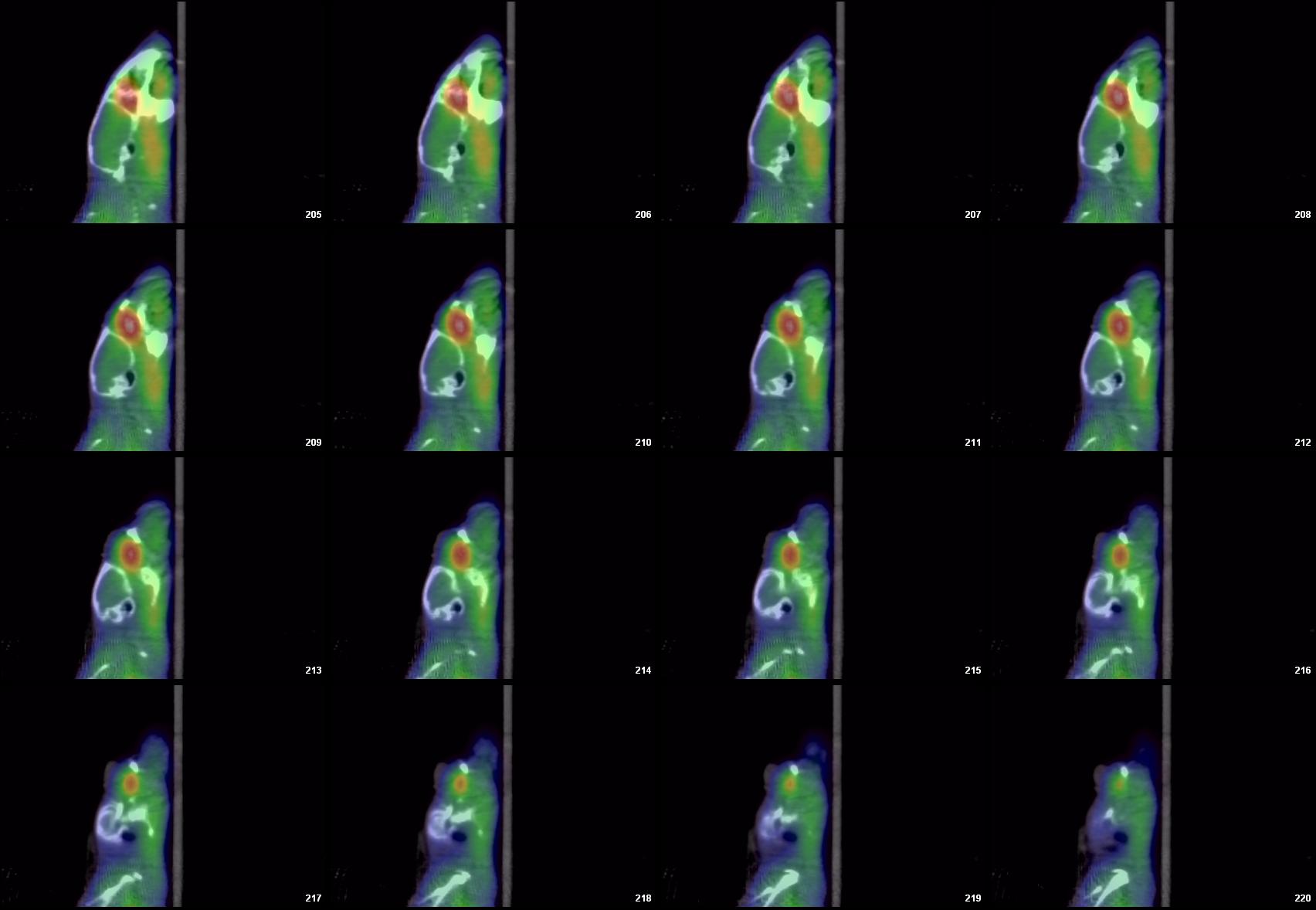

Supplement: Supplementary Figure 2 — Immunohistochemistry of hippocampal tissues from AD mice. (A) Histological changes in the hippocampus of 9-month-old WT and 3 × Tg-AD mice were analyzed by HE staining. (B–D) Analysis of neuronal status in the hippocampus of 9-month-old WT and 3 × Tg-AD mice by Nissler staining (n = 3 mice per group). (E–H) Analysis and quantification of Aβ deposition in hippocampal tissue of 9-month-old WT and 3 × Tg-AD mice by immunofluorescence (n = 4 mice per group). [file Data_Sheet_1.zip › FIG 2 pET-CT/PET-CT/M4/10.jpg]

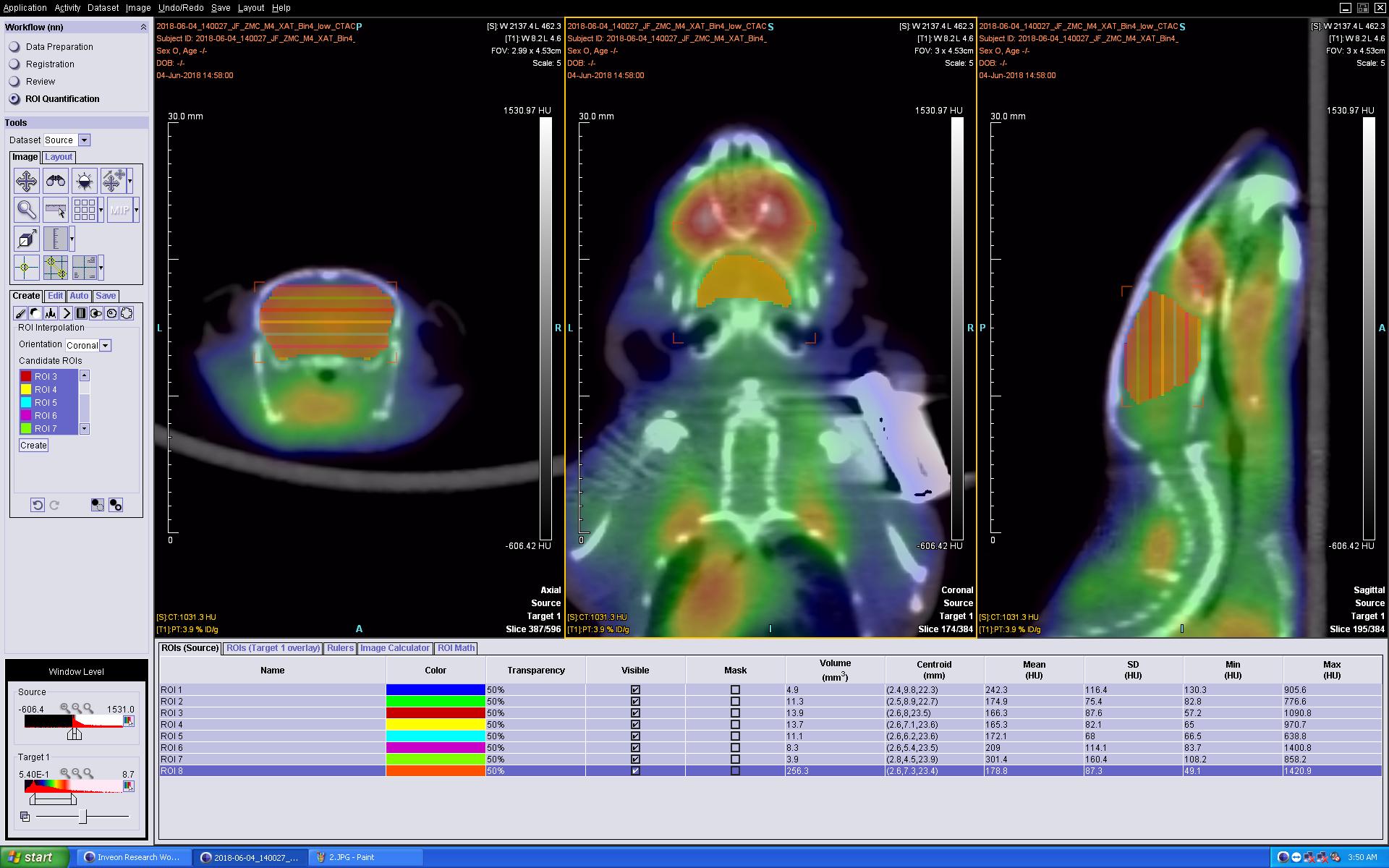

Supplement: Supplementary Figure 2 — Immunohistochemistry of hippocampal tissues from AD mice. (A) Histological changes in the hippocampus of 9-month-old WT and 3 × Tg-AD mice were analyzed by HE staining. (B–D) Analysis of neuronal status in the hippocampus of 9-month-old WT and 3 × Tg-AD mice by Nissler staining (n = 3 mice per group). (E–H) Analysis and quantification of Aβ deposition in hippocampal tissue of 9-month-old WT and 3 × Tg-AD mice by immunofluorescence (n = 4 mice per group). [file Data_Sheet_1.zip › FIG 2 pET-CT/PET-CT/M4/11.JPG]

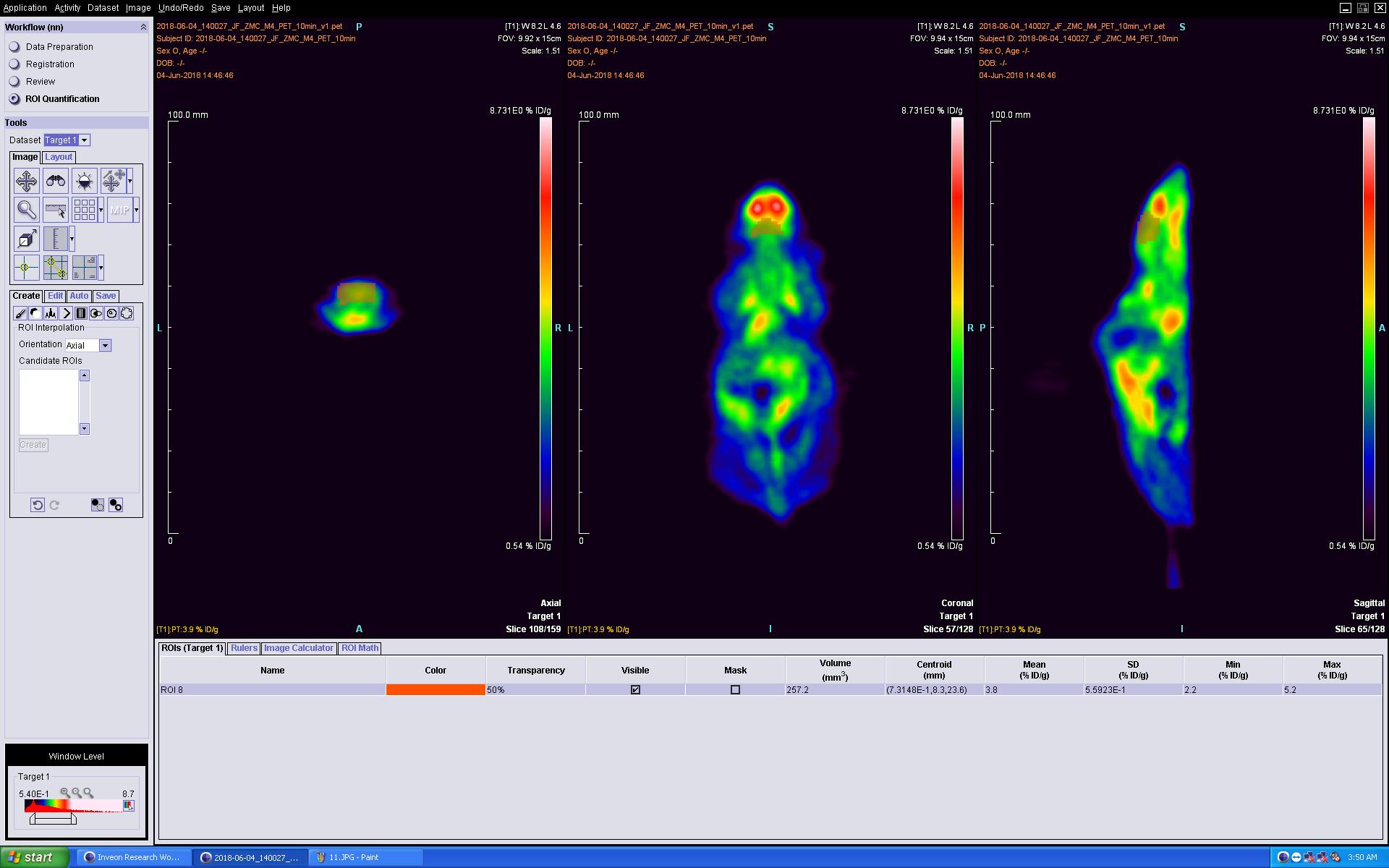

Supplement: Supplementary Figure 2 — Immunohistochemistry of hippocampal tissues from AD mice. (A) Histological changes in the hippocampus of 9-month-old WT and 3 × Tg-AD mice were analyzed by HE staining. (B–D) Analysis of neuronal status in the hippocampus of 9-month-old WT and 3 × Tg-AD mice by Nissler staining (n = 3 mice per group). (E–H) Analysis and quantification of Aβ deposition in hippocampal tissue of 9-month-old WT and 3 × Tg-AD mice by immunofluorescence (n = 4 mice per group). [file Data_Sheet_1.zip › FIG 2 pET-CT/PET-CT/M4/12.JPG]

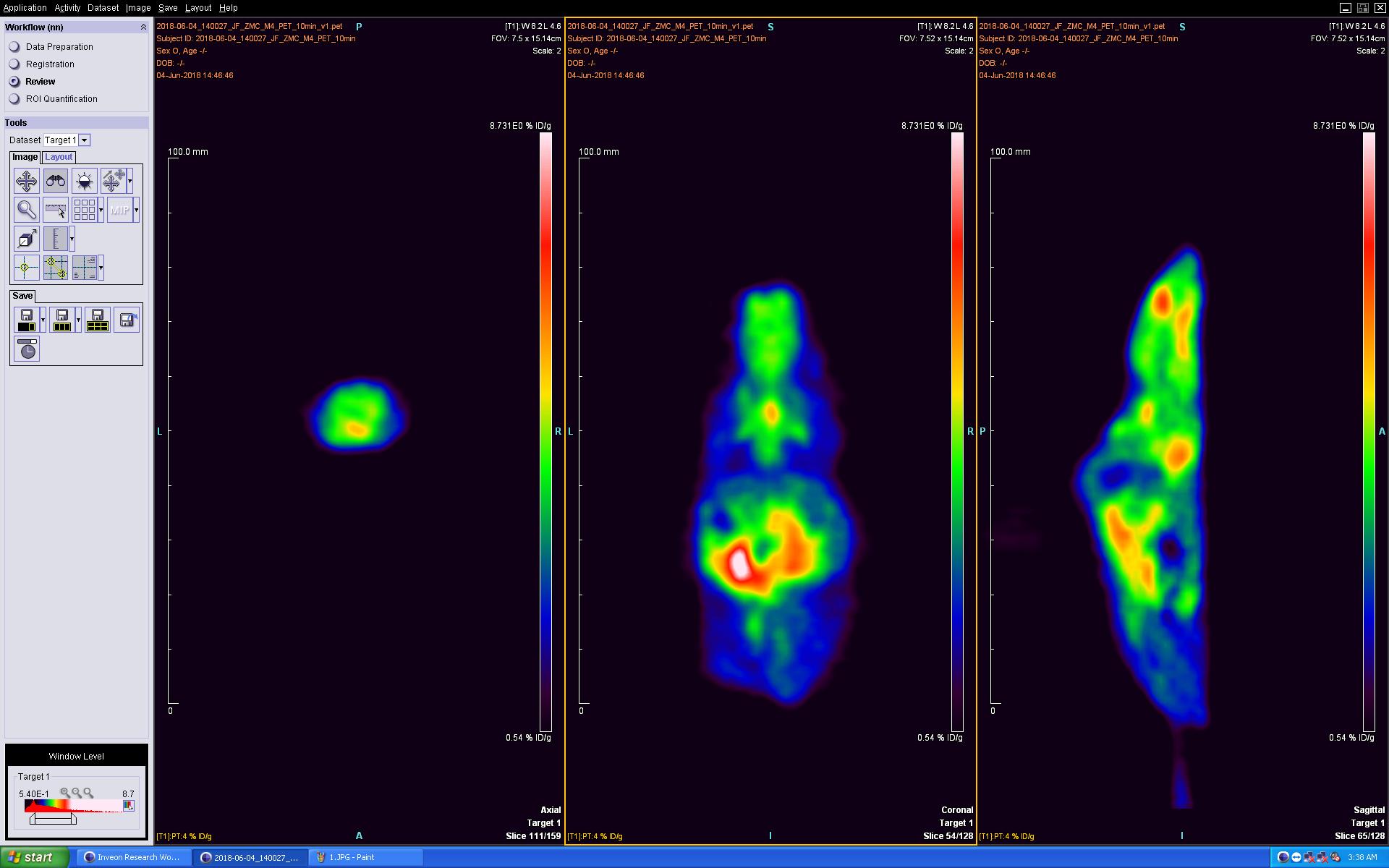

Supplement: Supplementary Figure 2 — Immunohistochemistry of hippocampal tissues from AD mice. (A) Histological changes in the hippocampus of 9-month-old WT and 3 × Tg-AD mice were analyzed by HE staining. (B–D) Analysis of neuronal status in the hippocampus of 9-month-old WT and 3 × Tg-AD mice by Nissler staining (n = 3 mice per group). (E–H) Analysis and quantification of Aβ deposition in hippocampal tissue of 9-month-old WT and 3 × Tg-AD mice by immunofluorescence (n = 4 mice per group). [file Data_Sheet_1.zip › FIG 2 pET-CT/PET-CT/M4/2.JPG]

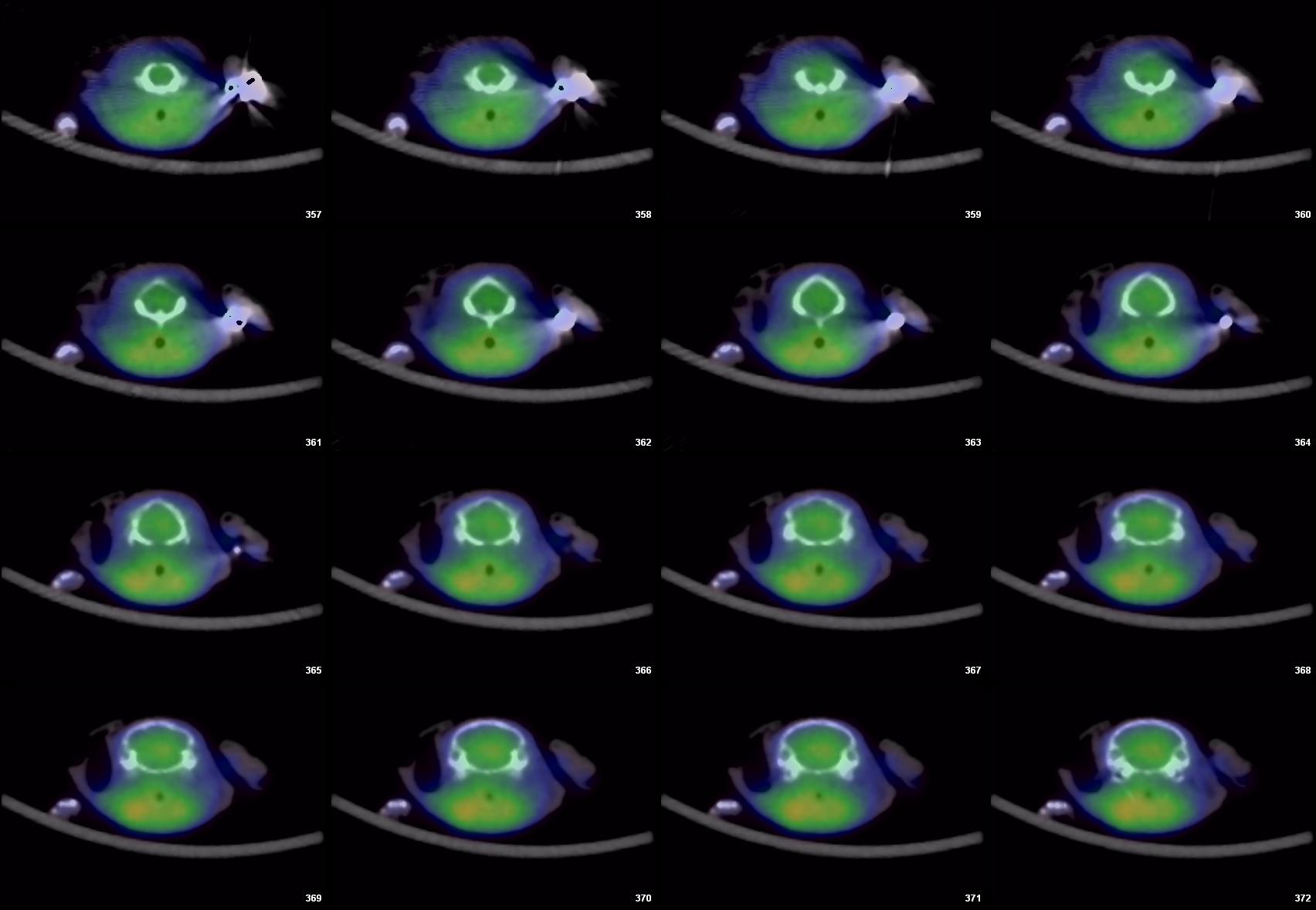

Supplement: Supplementary Figure 2 — Immunohistochemistry of hippocampal tissues from AD mice. (A) Histological changes in the hippocampus of 9-month-old WT and 3 × Tg-AD mice were analyzed by HE staining. (B–D) Analysis of neuronal status in the hippocampus of 9-month-old WT and 3 × Tg-AD mice by Nissler staining (n = 3 mice per group). (E–H) Analysis and quantification of Aβ deposition in hippocampal tissue of 9-month-old WT and 3 × Tg-AD mice by immunofluorescence (n = 4 mice per group). [file Data_Sheet_1.zip › FIG 2 pET-CT/PET-CT/M4/3.jpg]

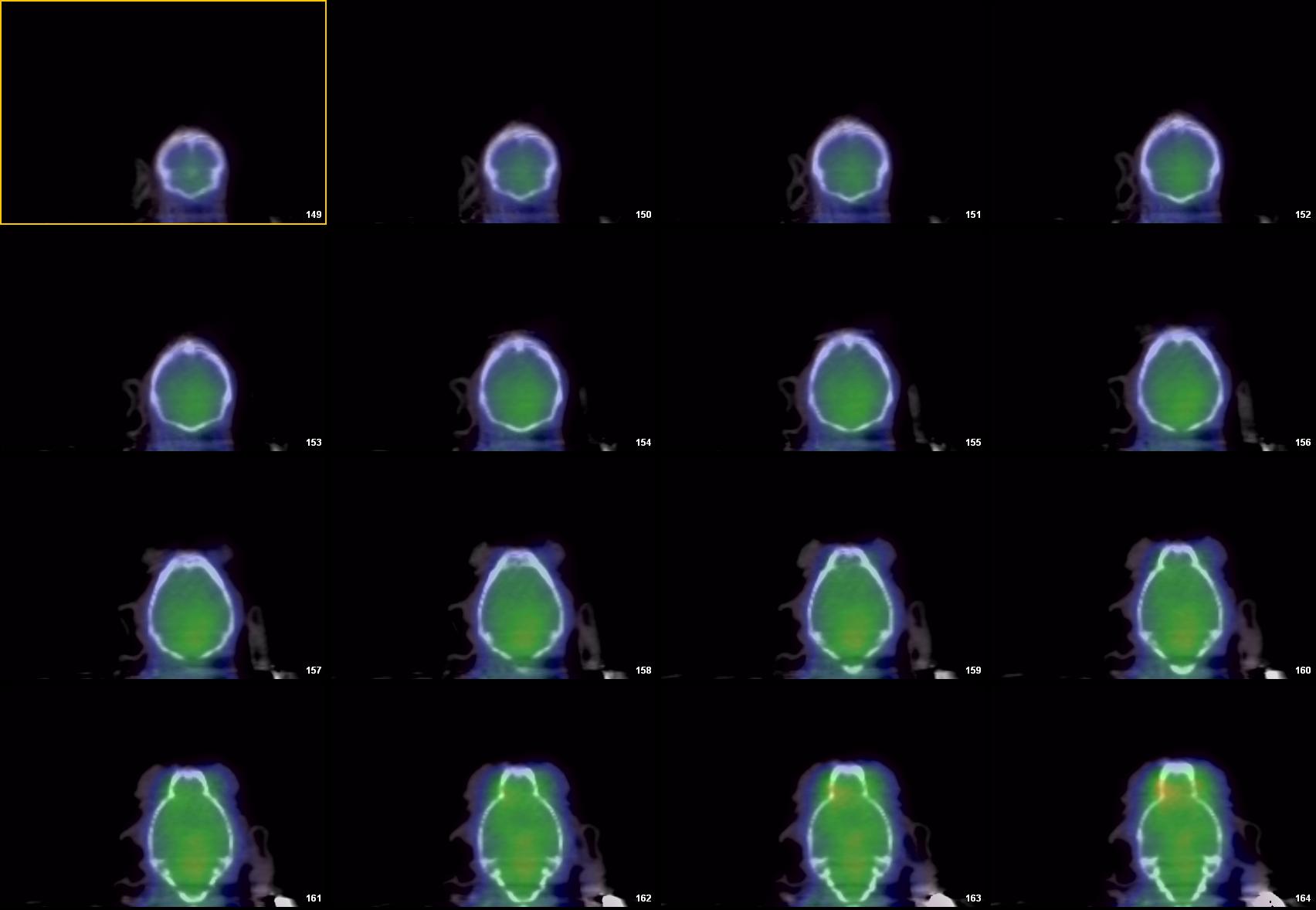

Supplement: Supplementary Figure 2 — Immunohistochemistry of hippocampal tissues from AD mice. (A) Histological changes in the hippocampus of 9-month-old WT and 3 × Tg-AD mice were analyzed by HE staining. (B–D) Analysis of neuronal status in the hippocampus of 9-month-old WT and 3 × Tg-AD mice by Nissler staining (n = 3 mice per group). (E–H) Analysis and quantification of Aβ deposition in hippocampal tissue of 9-month-old WT and 3 × Tg-AD mice by immunofluorescence (n = 4 mice per group). [file Data_Sheet_1.zip › FIG 2 pET-CT/PET-CT/M4/6.jpg]

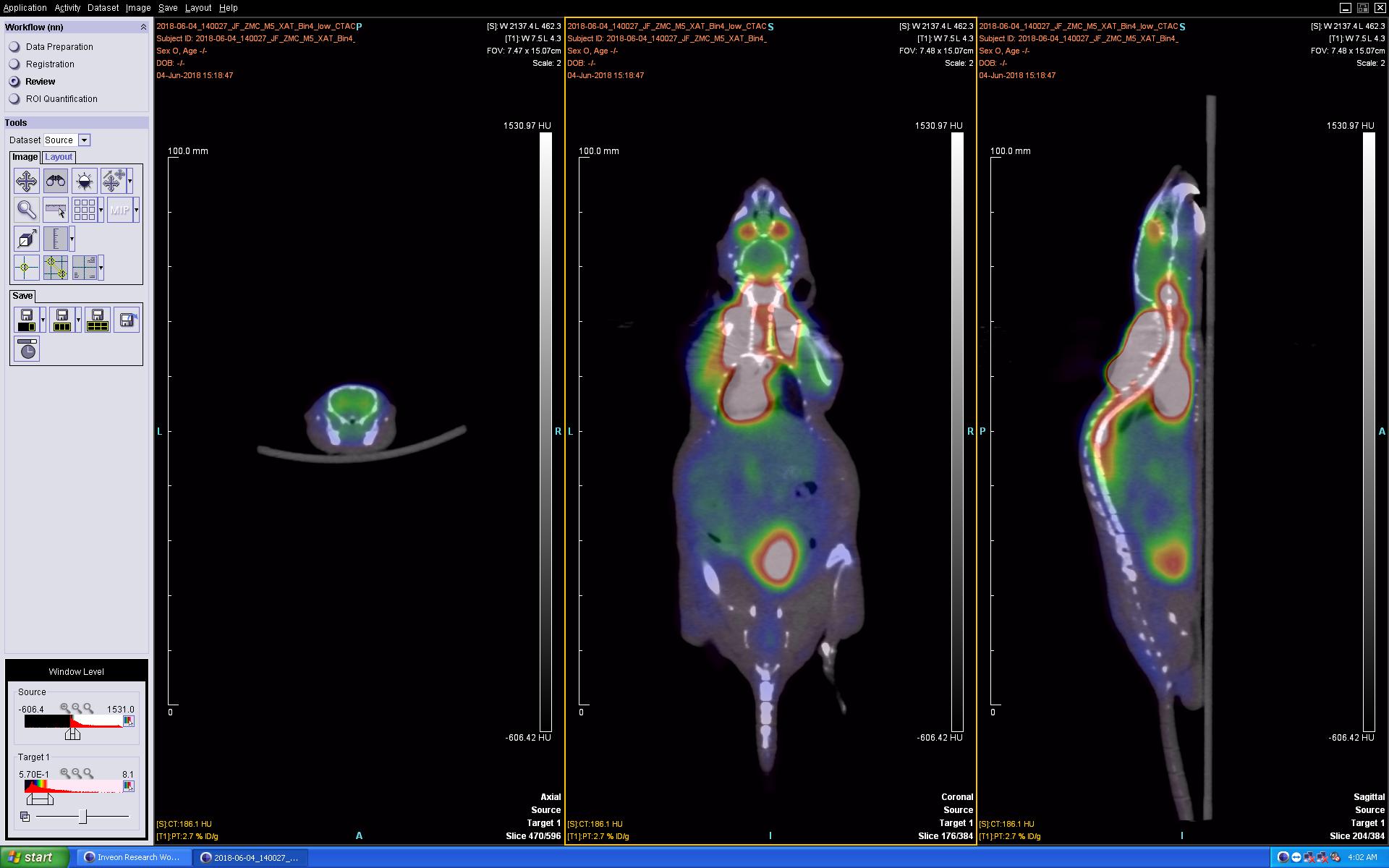

Supplement: Supplementary Figure 2 — Immunohistochemistry of hippocampal tissues from AD mice. (A) Histological changes in the hippocampus of 9-month-old WT and 3 × Tg-AD mice were analyzed by HE staining. (B–D) Analysis of neuronal status in the hippocampus of 9-month-old WT and 3 × Tg-AD mice by Nissler staining (n = 3 mice per group). (E–H) Analysis and quantification of Aβ deposition in hippocampal tissue of 9-month-old WT and 3 × Tg-AD mice by immunofluorescence (n = 4 mice per group). [file Data_Sheet_1.zip › FIG 2 pET-CT/PET-CT/M5/1.JPG]

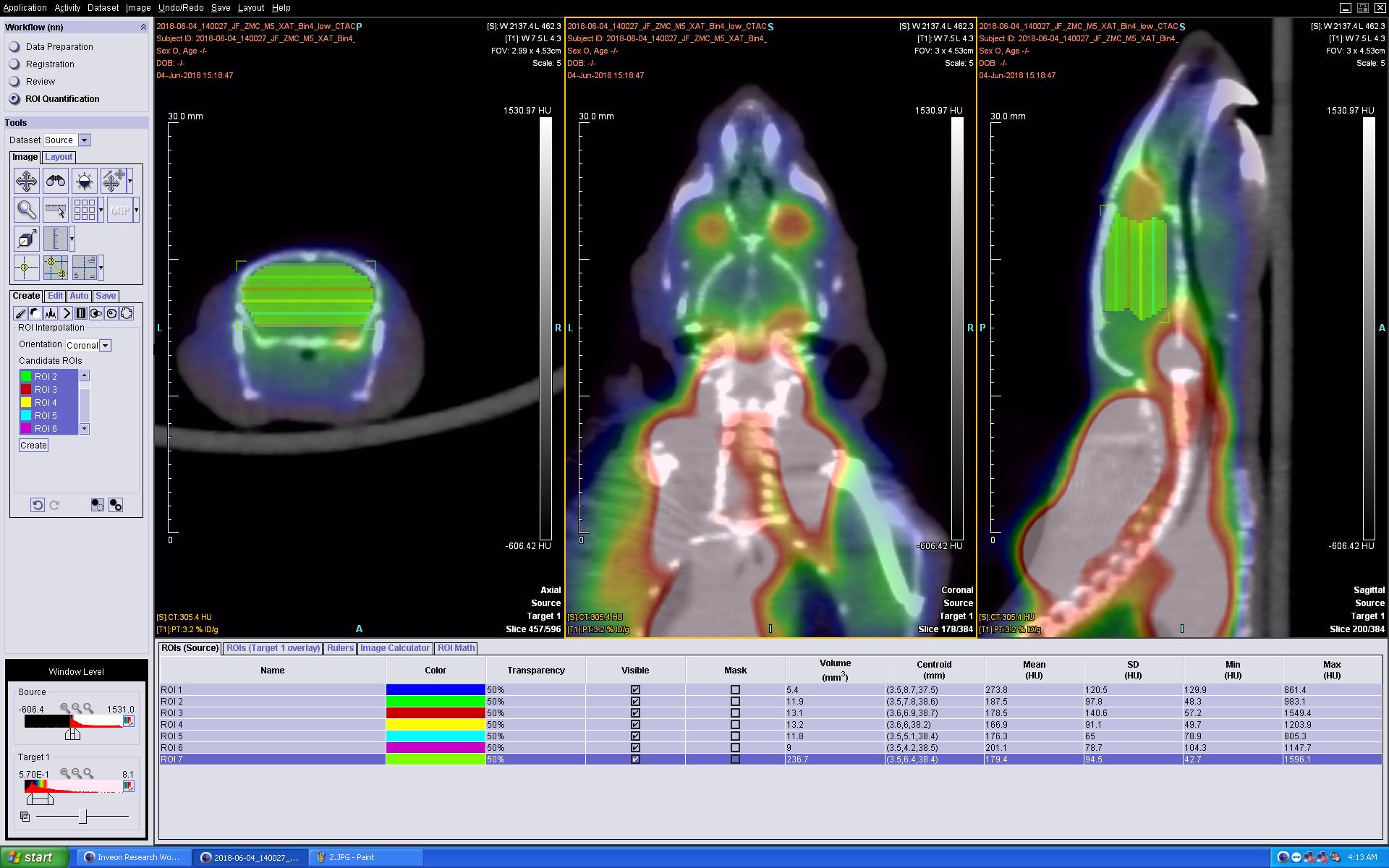

Supplement: Supplementary Figure 2 — Immunohistochemistry of hippocampal tissues from AD mice. (A) Histological changes in the hippocampus of 9-month-old WT and 3 × Tg-AD mice were analyzed by HE staining. (B–D) Analysis of neuronal status in the hippocampus of 9-month-old WT and 3 × Tg-AD mice by Nissler staining (n = 3 mice per group). (E–H) Analysis and quantification of Aβ deposition in hippocampal tissue of 9-month-old WT and 3 × Tg-AD mice by immunofluorescence (n = 4 mice per group). [file Data_Sheet_1.zip › FIG 2 pET-CT/PET-CT/M5/11.JPG]

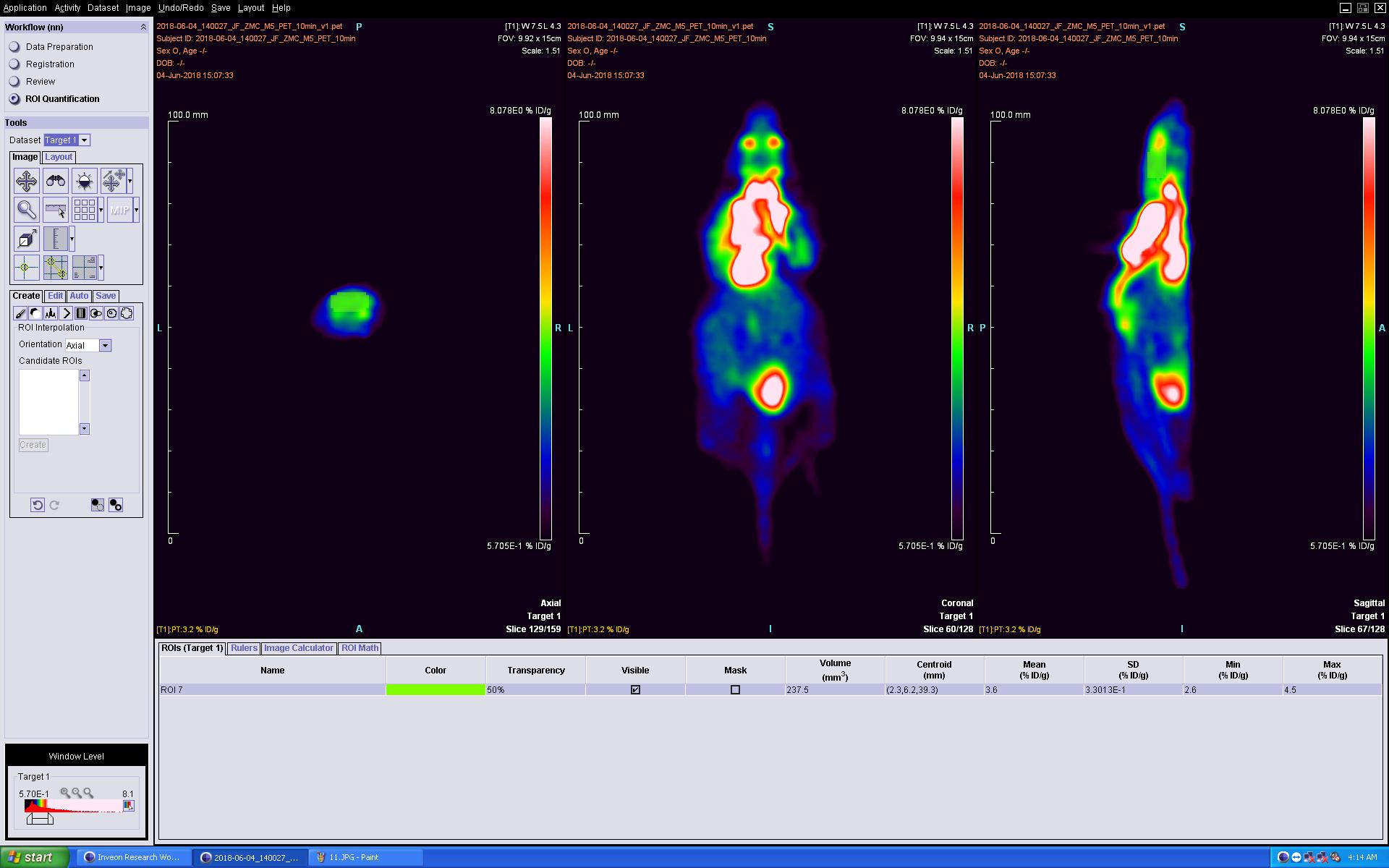

Supplement: Supplementary Figure 2 — Immunohistochemistry of hippocampal tissues from AD mice. (A) Histological changes in the hippocampus of 9-month-old WT and 3 × Tg-AD mice were analyzed by HE staining. (B–D) Analysis of neuronal status in the hippocampus of 9-month-old WT and 3 × Tg-AD mice by Nissler staining (n = 3 mice per group). (E–H) Analysis and quantification of Aβ deposition in hippocampal tissue of 9-month-old WT and 3 × Tg-AD mice by immunofluorescence (n = 4 mice per group). [file Data_Sheet_1.zip › FIG 2 pET-CT/PET-CT/M5/12.JPG]

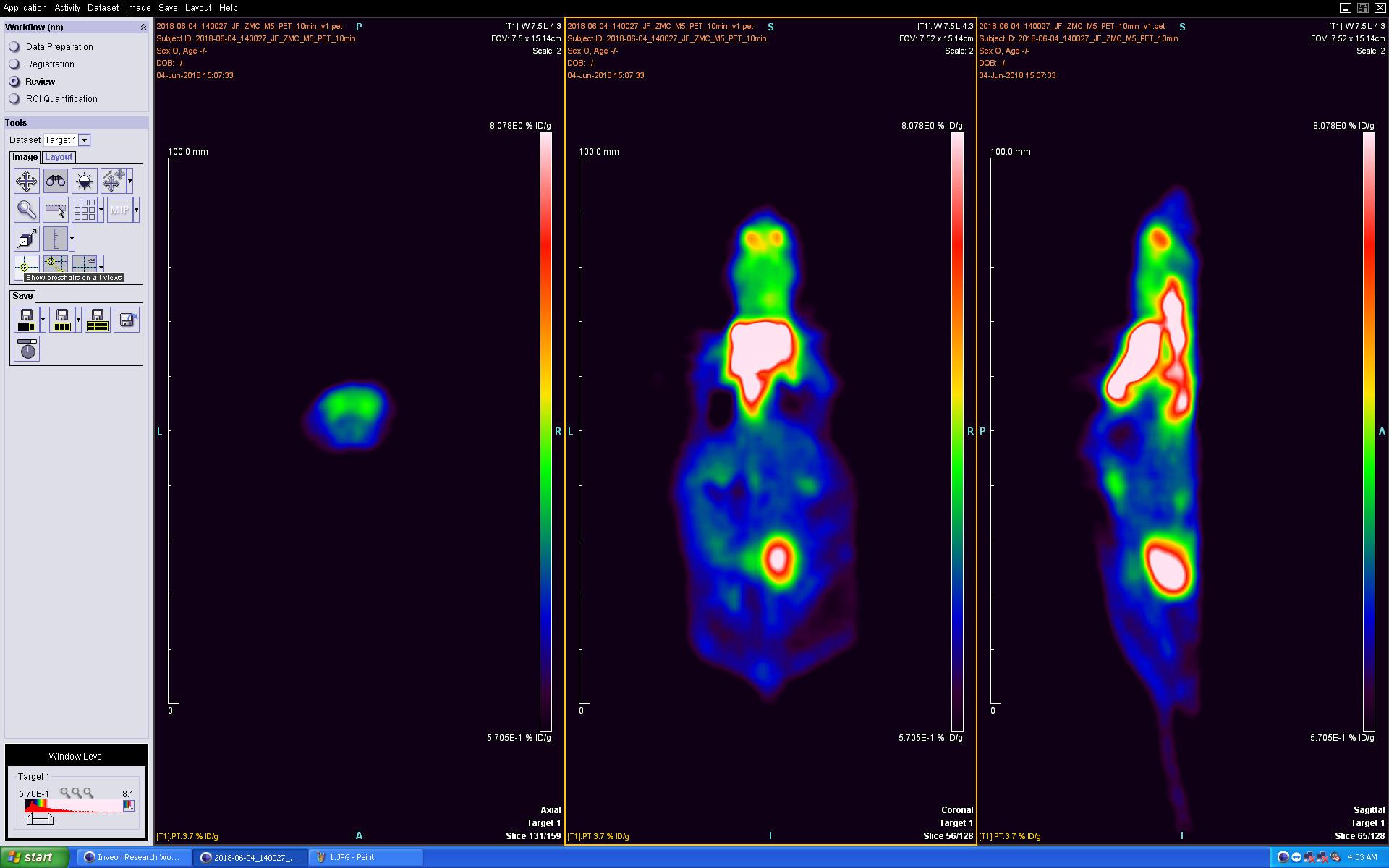

Supplement: Supplementary Figure 2 — Immunohistochemistry of hippocampal tissues from AD mice. (A) Histological changes in the hippocampus of 9-month-old WT and 3 × Tg-AD mice were analyzed by HE staining. (B–D) Analysis of neuronal status in the hippocampus of 9-month-old WT and 3 × Tg-AD mice by Nissler staining (n = 3 mice per group). (E–H) Analysis and quantification of Aβ deposition in hippocampal tissue of 9-month-old WT and 3 × Tg-AD mice by immunofluorescence (n = 4 mice per group). [file Data_Sheet_1.zip › FIG 2 pET-CT/PET-CT/M5/2.JPG]

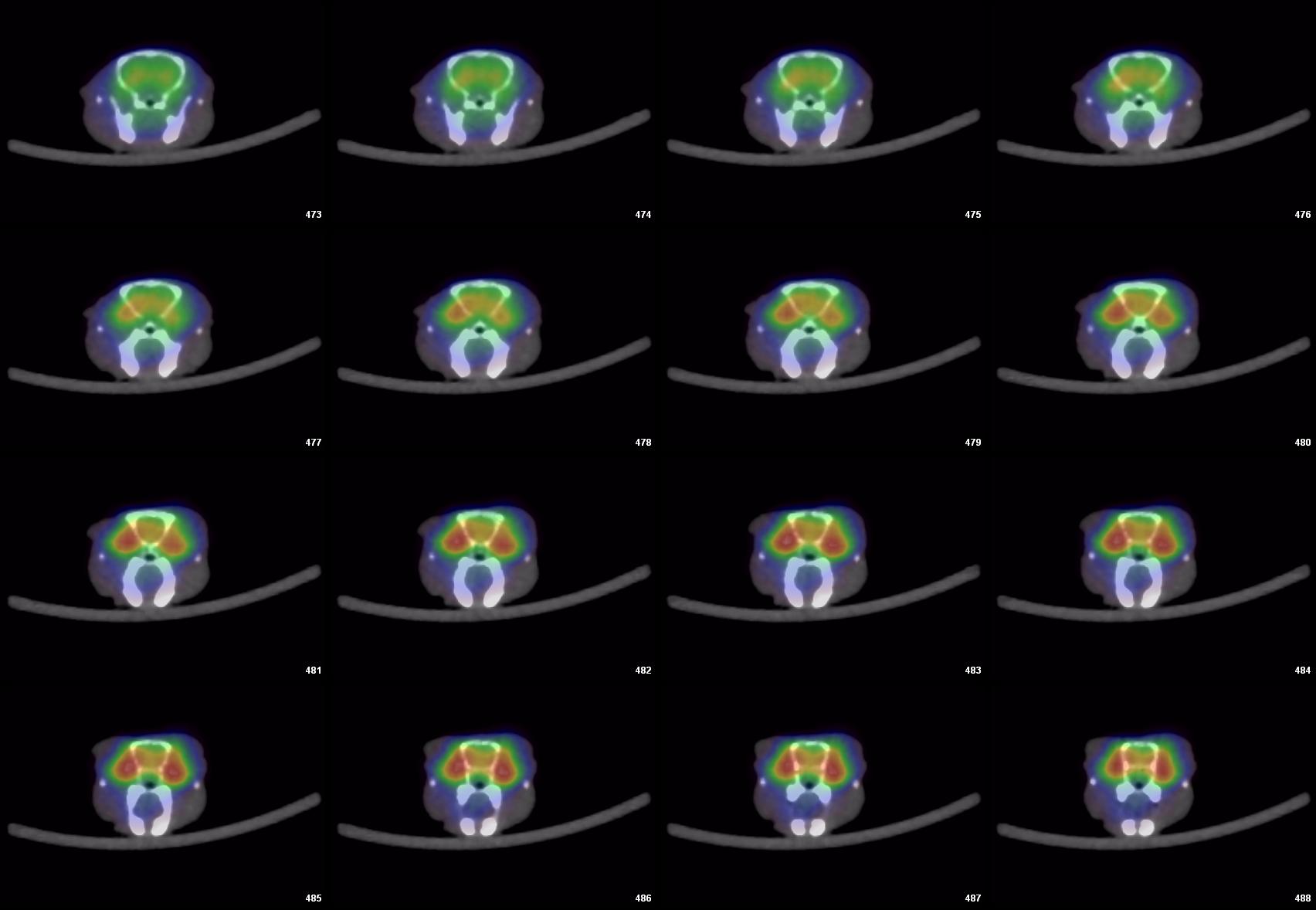

Supplement: Supplementary Figure 2 — Immunohistochemistry of hippocampal tissues from AD mice. (A) Histological changes in the hippocampus of 9-month-old WT and 3 × Tg-AD mice were analyzed by HE staining. (B–D) Analysis of neuronal status in the hippocampus of 9-month-old WT and 3 × Tg-AD mice by Nissler staining (n = 3 mice per group). (E–H) Analysis and quantification of Aβ deposition in hippocampal tissue of 9-month-old WT and 3 × Tg-AD mice by immunofluorescence (n = 4 mice per group). [file Data_Sheet_1.zip › FIG 2 pET-CT/PET-CT/M5/5.jpg]

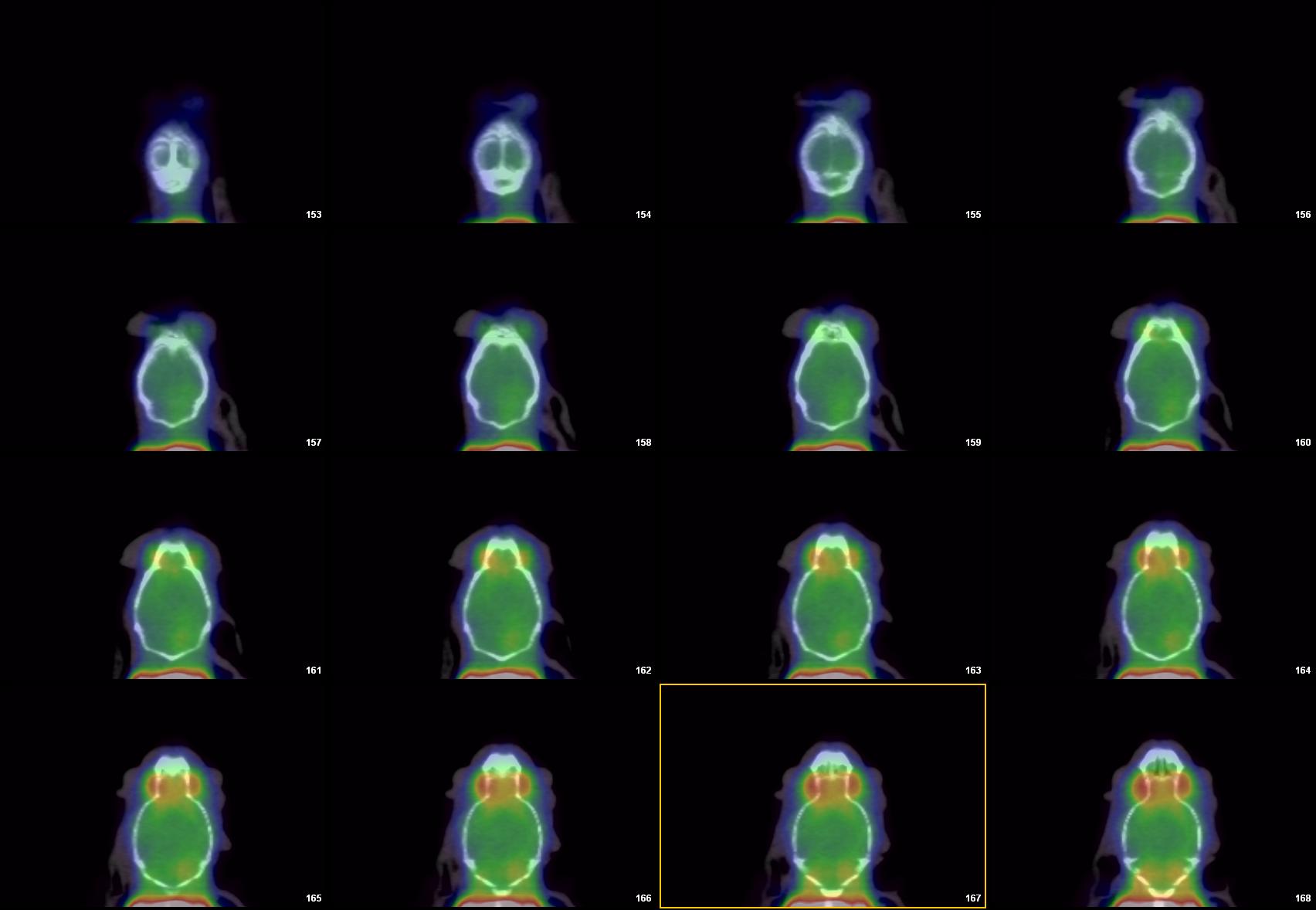

Supplement: Supplementary Figure 2 — Immunohistochemistry of hippocampal tissues from AD mice. (A) Histological changes in the hippocampus of 9-month-old WT and 3 × Tg-AD mice were analyzed by HE staining. (B–D) Analysis of neuronal status in the hippocampus of 9-month-old WT and 3 × Tg-AD mice by Nissler staining (n = 3 mice per group). (E–H) Analysis and quantification of Aβ deposition in hippocampal tissue of 9-month-old WT and 3 × Tg-AD mice by immunofluorescence (n = 4 mice per group). [file Data_Sheet_1.zip › FIG 2 pET-CT/PET-CT/M5/6.jpg]

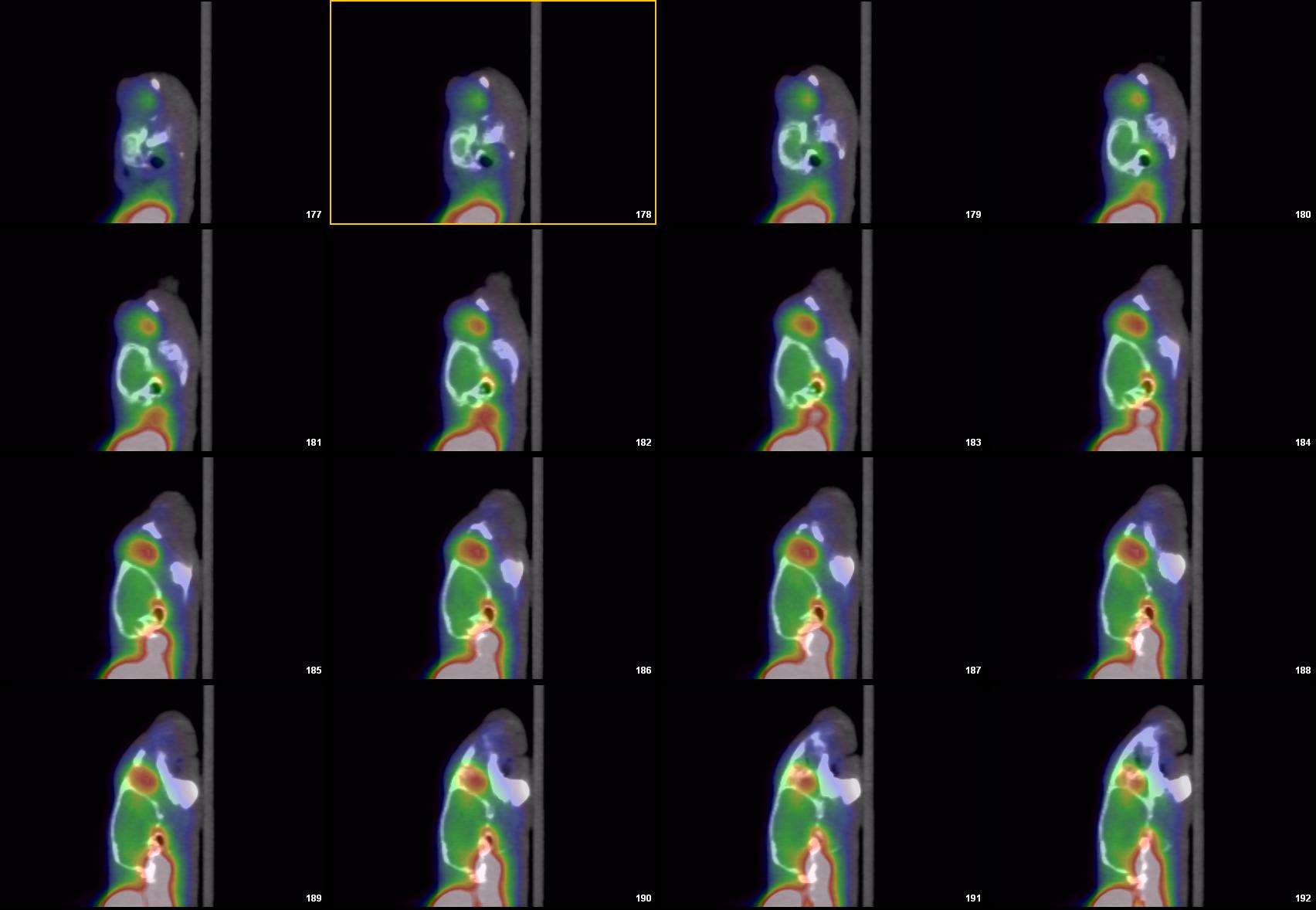

Supplement: Supplementary Figure 2 — Immunohistochemistry of hippocampal tissues from AD mice. (A) Histological changes in the hippocampus of 9-month-old WT and 3 × Tg-AD mice were analyzed by HE staining. (B–D) Analysis of neuronal status in the hippocampus of 9-month-old WT and 3 × Tg-AD mice by Nissler staining (n = 3 mice per group). (E–H) Analysis and quantification of Aβ deposition in hippocampal tissue of 9-month-old WT and 3 × Tg-AD mice by immunofluorescence (n = 4 mice per group). [file Data_Sheet_1.zip › FIG 2 pET-CT/PET-CT/M5/8.jpg]

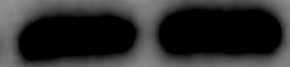

Supplement: Supplementary Figure 3 — Prospects: other regulatory roles of hnRNP A1. (A) Observation of the effect of different concentrations of VPC-80051 on viability in HT22 cells (n = 6 per group). (B) Enrichment of PKM1 and PKM2 mRNA by qPCR after pulling down RNA using hnRNP A1 antibody (n = 6 per group). (C) Observation of the effect of hnRNP A1 overexpression on lactate content of Aβ25–35-induced HT22 cells (n = 3 per group). (D) Observation of the effect of hnRNP A1 overexpression on GSH of Aβ25–35-induced HT22 cells (n = 3 per group). (E) Observation of the effect of hnRNP A1 overexpression on ROS of Aβ25–35-induced HT22 cells (n = 3 per group). (F) To observe the effect of hnRNP A1 overexpression on GSH after the use of glycolysis inhibitor 2-DG (n = 3 per group). (G) To observe the effect of hnRNP A1 overexpression on ROS after the use of glycolysis inhibitor 2-DG (n = 3 per group). [file Data_Sheet_5.zip › FIG3/HK pro(OE-A1)/HK1/12.13 hk 4.3.png]

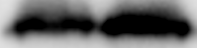

Supplement: Supplementary Figure 3 — Prospects: other regulatory roles of hnRNP A1. (A) Observation of the effect of different concentrations of VPC-80051 on viability in HT22 cells (n = 6 per group). (B) Enrichment of PKM1 and PKM2 mRNA by qPCR after pulling down RNA using hnRNP A1 antibody (n = 6 per group). (C) Observation of the effect of hnRNP A1 overexpression on lactate content of Aβ25–35-induced HT22 cells (n = 3 per group). (D) Observation of the effect of hnRNP A1 overexpression on GSH of Aβ25–35-induced HT22 cells (n = 3 per group). (E) Observation of the effect of hnRNP A1 overexpression on ROS of Aβ25–35-induced HT22 cells (n = 3 per group). (F) To observe the effect of hnRNP A1 overexpression on GSH after the use of glycolysis inhibitor 2-DG (n = 3 per group). (G) To observe the effect of hnRNP A1 overexpression on ROS after the use of glycolysis inhibitor 2-DG (n = 3 per group). [file Data_Sheet_5.zip › FIG3/HK pro(OE-A1)/HK1/12.13 hk4.2.png]

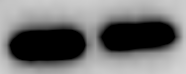

Supplement: Supplementary Figure 3 — Prospects: other regulatory roles of hnRNP A1. (A) Observation of the effect of different concentrations of VPC-80051 on viability in HT22 cells (n = 6 per group). (B) Enrichment of PKM1 and PKM2 mRNA by qPCR after pulling down RNA using hnRNP A1 antibody (n = 6 per group). (C) Observation of the effect of hnRNP A1 overexpression on lactate content of Aβ25–35-induced HT22 cells (n = 3 per group). (D) Observation of the effect of hnRNP A1 overexpression on GSH of Aβ25–35-induced HT22 cells (n = 3 per group). (E) Observation of the effect of hnRNP A1 overexpression on ROS of Aβ25–35-induced HT22 cells (n = 3 per group). (F) To observe the effect of hnRNP A1 overexpression on GSH after the use of glycolysis inhibitor 2-DG (n = 3 per group). (G) To observe the effect of hnRNP A1 overexpression on ROS after the use of glycolysis inhibitor 2-DG (n = 3 per group). [file Data_Sheet_5.zip › FIG3/HK pro(OE-A1)/HK1/12.13 tub 4.3.png]

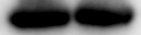

Supplement: Supplementary Figure 3 — Prospects: other regulatory roles of hnRNP A1. (A) Observation of the effect of different concentrations of VPC-80051 on viability in HT22 cells (n = 6 per group). (B) Enrichment of PKM1 and PKM2 mRNA by qPCR after pulling down RNA using hnRNP A1 antibody (n = 6 per group). (C) Observation of the effect of hnRNP A1 overexpression on lactate content of Aβ25–35-induced HT22 cells (n = 3 per group). (D) Observation of the effect of hnRNP A1 overexpression on GSH of Aβ25–35-induced HT22 cells (n = 3 per group). (E) Observation of the effect of hnRNP A1 overexpression on ROS of Aβ25–35-induced HT22 cells (n = 3 per group). (F) To observe the effect of hnRNP A1 overexpression on GSH after the use of glycolysis inhibitor 2-DG (n = 3 per group). (G) To observe the effect of hnRNP A1 overexpression on ROS after the use of glycolysis inhibitor 2-DG (n = 3 per group). [file Data_Sheet_5.zip › FIG3/HK pro(OE-A1)/HK1/12.13 tub4.2.png]

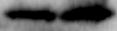

Supplement: Supplementary Figure 3 — Prospects: other regulatory roles of hnRNP A1. (A) Observation of the effect of different concentrations of VPC-80051 on viability in HT22 cells (n = 6 per group). (B) Enrichment of PKM1 and PKM2 mRNA by qPCR after pulling down RNA using hnRNP A1 antibody (n = 6 per group). (C) Observation of the effect of hnRNP A1 overexpression on lactate content of Aβ25–35-induced HT22 cells (n = 3 per group). (D) Observation of the effect of hnRNP A1 overexpression on GSH of Aβ25–35-induced HT22 cells (n = 3 per group). (E) Observation of the effect of hnRNP A1 overexpression on ROS of Aβ25–35-induced HT22 cells (n = 3 per group). (F) To observe the effect of hnRNP A1 overexpression on GSH after the use of glycolysis inhibitor 2-DG (n = 3 per group). (G) To observe the effect of hnRNP A1 overexpression on ROS after the use of glycolysis inhibitor 2-DG (n = 3 per group). [file Data_Sheet_5.zip › FIG3/HK pro(OE-A1)/HK1/2022-10-20 jxh 5'4hk.png]

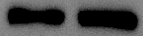

Supplement: Supplementary Figure 3 — Prospects: other regulatory roles of hnRNP A1. (A) Observation of the effect of different concentrations of VPC-80051 on viability in HT22 cells (n = 6 per group). (B) Enrichment of PKM1 and PKM2 mRNA by qPCR after pulling down RNA using hnRNP A1 antibody (n = 6 per group). (C) Observation of the effect of hnRNP A1 overexpression on lactate content of Aβ25–35-induced HT22 cells (n = 3 per group). (D) Observation of the effect of hnRNP A1 overexpression on GSH of Aβ25–35-induced HT22 cells (n = 3 per group). (E) Observation of the effect of hnRNP A1 overexpression on ROS of Aβ25–35-induced HT22 cells (n = 3 per group). (F) To observe the effect of hnRNP A1 overexpression on GSH after the use of glycolysis inhibitor 2-DG (n = 3 per group). (G) To observe the effect of hnRNP A1 overexpression on ROS after the use of glycolysis inhibitor 2-DG (n = 3 per group). [file Data_Sheet_5.zip › FIG3/HK pro(OE-A1)/HK1/2022.12.13 HK2.1.png]

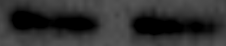

Supplement: Supplementary Figure 3 — Prospects: other regulatory roles of hnRNP A1. (A) Observation of the effect of different concentrations of VPC-80051 on viability in HT22 cells (n = 6 per group). (B) Enrichment of PKM1 and PKM2 mRNA by qPCR after pulling down RNA using hnRNP A1 antibody (n = 6 per group). (C) Observation of the effect of hnRNP A1 overexpression on lactate content of Aβ25–35-induced HT22 cells (n = 3 per group). (D) Observation of the effect of hnRNP A1 overexpression on GSH of Aβ25–35-induced HT22 cells (n = 3 per group). (E) Observation of the effect of hnRNP A1 overexpression on ROS of Aβ25–35-induced HT22 cells (n = 3 per group). (F) To observe the effect of hnRNP A1 overexpression on GSH after the use of glycolysis inhibitor 2-DG (n = 3 per group). (G) To observe the effect of hnRNP A1 overexpression on ROS after the use of glycolysis inhibitor 2-DG (n = 3 per group). [file Data_Sheet_5.zip › FIG3/HK pro(OE-A1)/HK1/2022.12.13 TUB2.1.png]

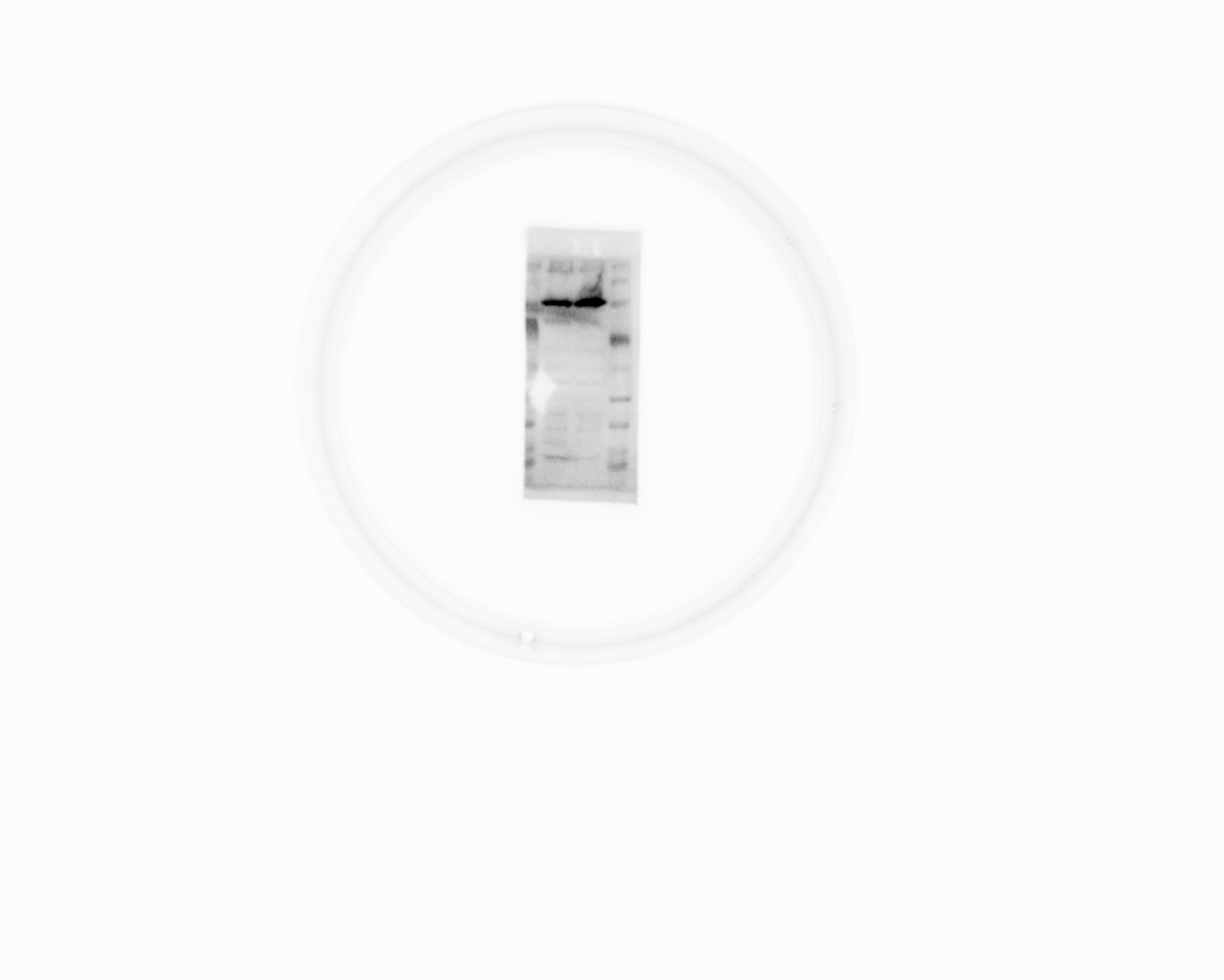

Supplement: Supplementary Figure 3 — Prospects: other regulatory roles of hnRNP A1. (A) Observation of the effect of different concentrations of VPC-80051 on viability in HT22 cells (n = 6 per group). (B) Enrichment of PKM1 and PKM2 mRNA by qPCR after pulling down RNA using hnRNP A1 antibody (n = 6 per group). (C) Observation of the effect of hnRNP A1 overexpression on lactate content of Aβ25–35-induced HT22 cells (n = 3 per group). (D) Observation of the effect of hnRNP A1 overexpression on GSH of Aβ25–35-induced HT22 cells (n = 3 per group). (E) Observation of the effect of hnRNP A1 overexpression on ROS of Aβ25–35-induced HT22 cells (n = 3 per group). (F) To observe the effect of hnRNP A1 overexpression on GSH after the use of glycolysis inhibitor 2-DG (n = 3 per group). (G) To observe the effect of hnRNP A1 overexpression on ROS after the use of glycolysis inhibitor 2-DG (n = 3 per group). [file Data_Sheet_5.zip › FIG3/HK pro(OE-A1)/HK1/original data/2022-10-20 5'4hk.tif]

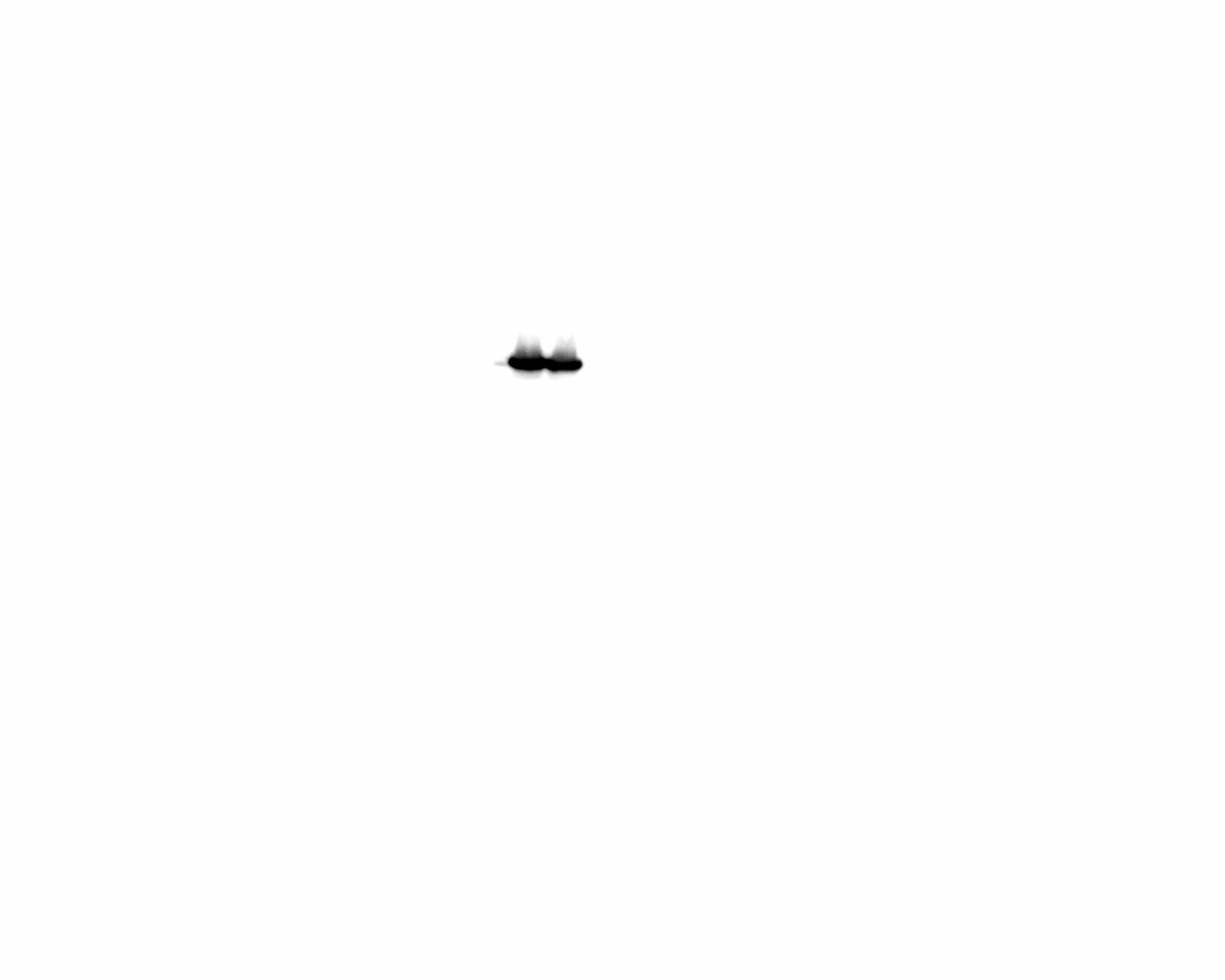

Supplement: Supplementary Figure 3 — Prospects: other regulatory roles of hnRNP A1. (A) Observation of the effect of different concentrations of VPC-80051 on viability in HT22 cells (n = 6 per group). (B) Enrichment of PKM1 and PKM2 mRNA by qPCR after pulling down RNA using hnRNP A1 antibody (n = 6 per group). (C) Observation of the effect of hnRNP A1 overexpression on lactate content of Aβ25–35-induced HT22 cells (n = 3 per group). (D) Observation of the effect of hnRNP A1 overexpression on GSH of Aβ25–35-induced HT22 cells (n = 3 per group). (E) Observation of the effect of hnRNP A1 overexpression on ROS of Aβ25–35-induced HT22 cells (n = 3 per group). (F) To observe the effect of hnRNP A1 overexpression on GSH after the use of glycolysis inhibitor 2-DG (n = 3 per group). (G) To observe the effect of hnRNP A1 overexpression on ROS after the use of glycolysis inhibitor 2-DG (n = 3 per group). [file Data_Sheet_5.zip › FIG3/HK pro(OE-A1)/HK1/original data/wb 2022-10-20 5'4tub.tif]

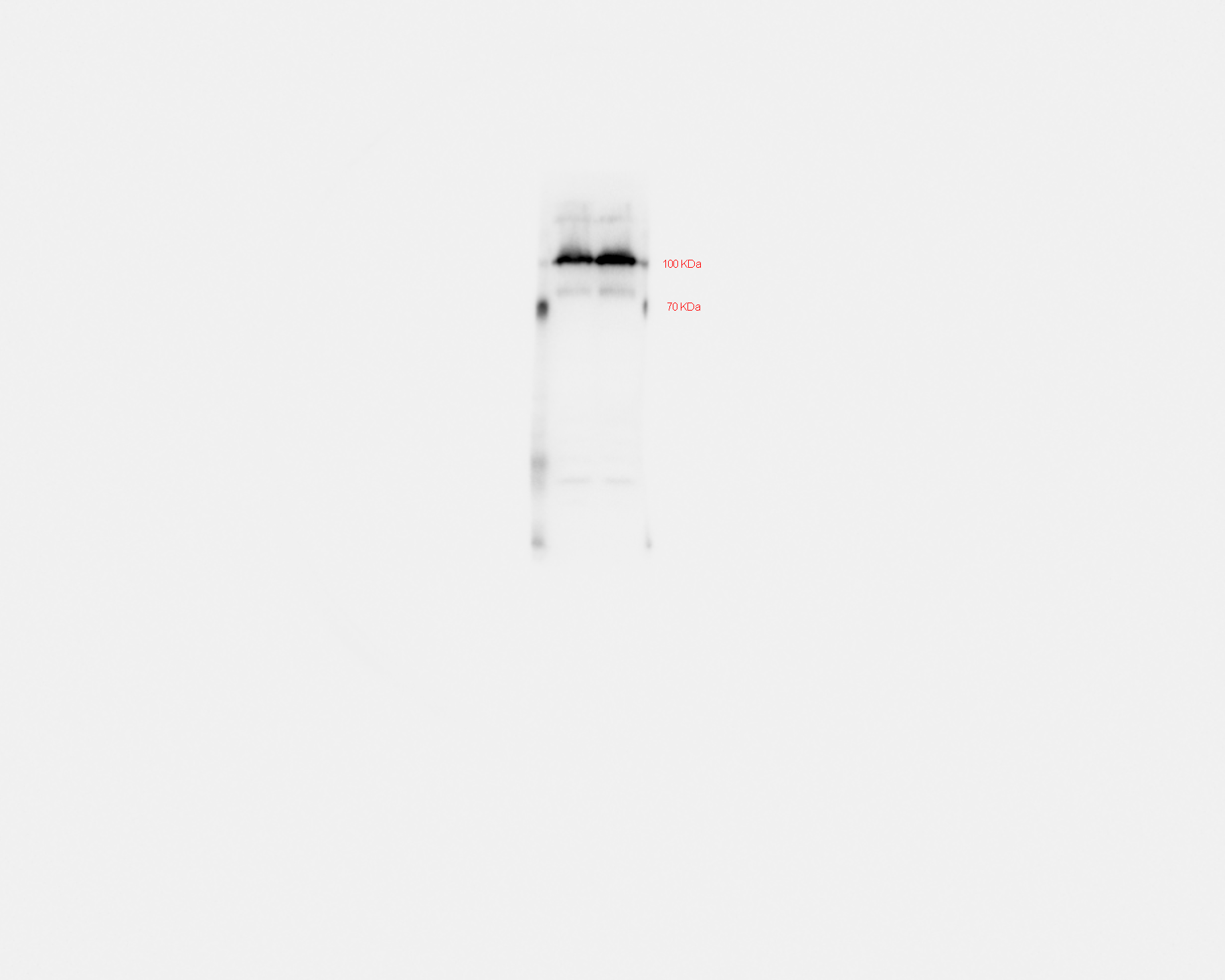

Supplement: Supplementary Figure 3 — Prospects: other regulatory roles of hnRNP A1. (A) Observation of the effect of different concentrations of VPC-80051 on viability in HT22 cells (n = 6 per group). (B) Enrichment of PKM1 and PKM2 mRNA by qPCR after pulling down RNA using hnRNP A1 antibody (n = 6 per group). (C) Observation of the effect of hnRNP A1 overexpression on lactate content of Aβ25–35-induced HT22 cells (n = 3 per group). (D) Observation of the effect of hnRNP A1 overexpression on GSH of Aβ25–35-induced HT22 cells (n = 3 per group). (E) Observation of the effect of hnRNP A1 overexpression on ROS of Aβ25–35-induced HT22 cells (n = 3 per group). (F) To observe the effect of hnRNP A1 overexpression on GSH after the use of glycolysis inhibitor 2-DG (n = 3 per group). (G) To observe the effect of hnRNP A1 overexpression on ROS after the use of glycolysis inhibitor 2-DG (n = 3 per group). [file Data_Sheet_5.zip › FIG3/HK pro(OE-A1)/HK1/original data/wb 2022-12-13 hk 4.2.tif]

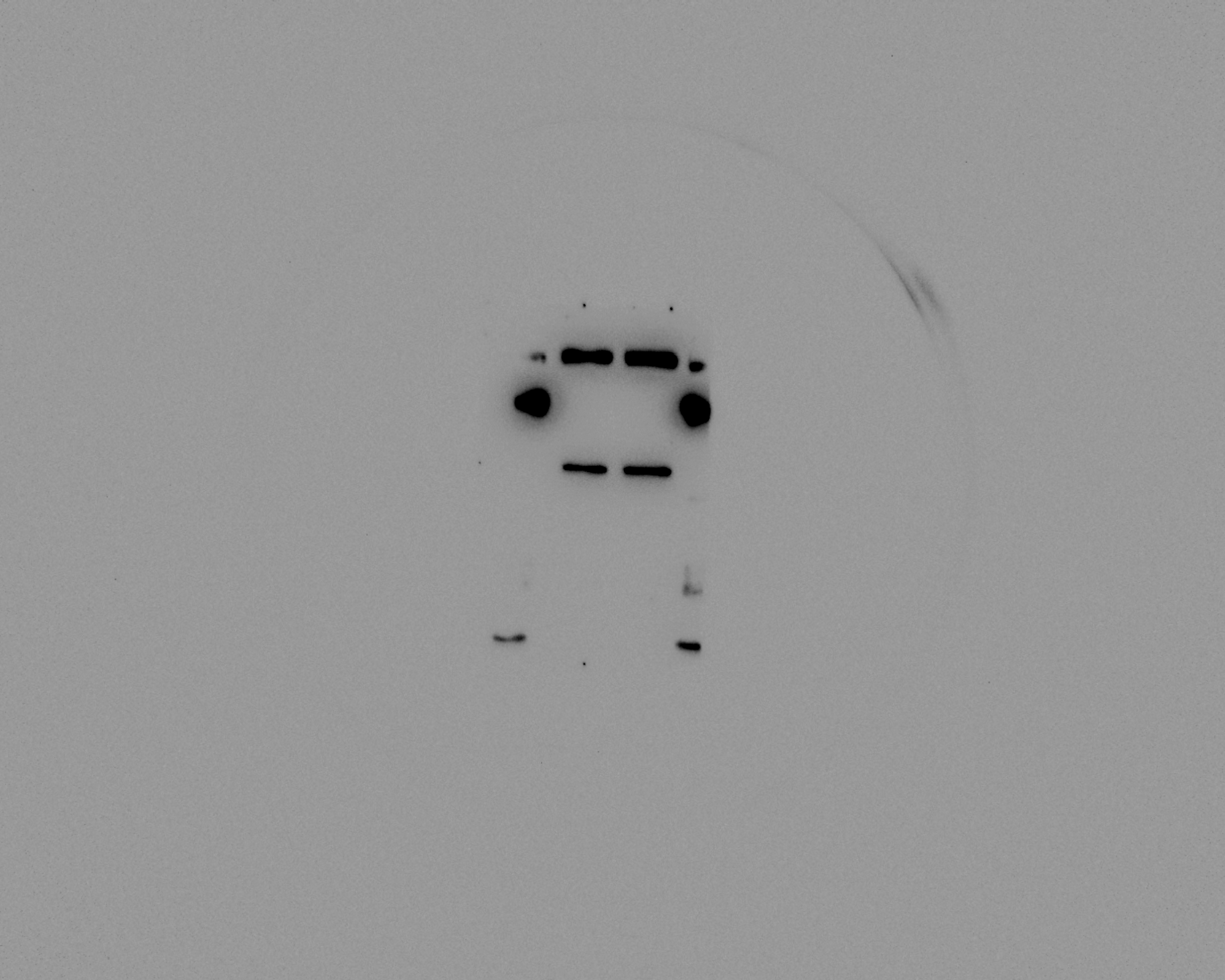

Supplement: Supplementary Figure 3 — Prospects: other regulatory roles of hnRNP A1. (A) Observation of the effect of different concentrations of VPC-80051 on viability in HT22 cells (n = 6 per group). (B) Enrichment of PKM1 and PKM2 mRNA by qPCR after pulling down RNA using hnRNP A1 antibody (n = 6 per group). (C) Observation of the effect of hnRNP A1 overexpression on lactate content of Aβ25–35-induced HT22 cells (n = 3 per group). (D) Observation of the effect of hnRNP A1 overexpression on GSH of Aβ25–35-induced HT22 cells (n = 3 per group). (E) Observation of the effect of hnRNP A1 overexpression on ROS of Aβ25–35-induced HT22 cells (n = 3 per group). (F) To observe the effect of hnRNP A1 overexpression on GSH after the use of glycolysis inhibitor 2-DG (n = 3 per group). (G) To observe the effect of hnRNP A1 overexpression on ROS after the use of glycolysis inhibitor 2-DG (n = 3 per group). [file Data_Sheet_5.zip › FIG3/HK pro(OE-A1)/HK1/original data/wb 2022-12-13 hk2.1.tif]

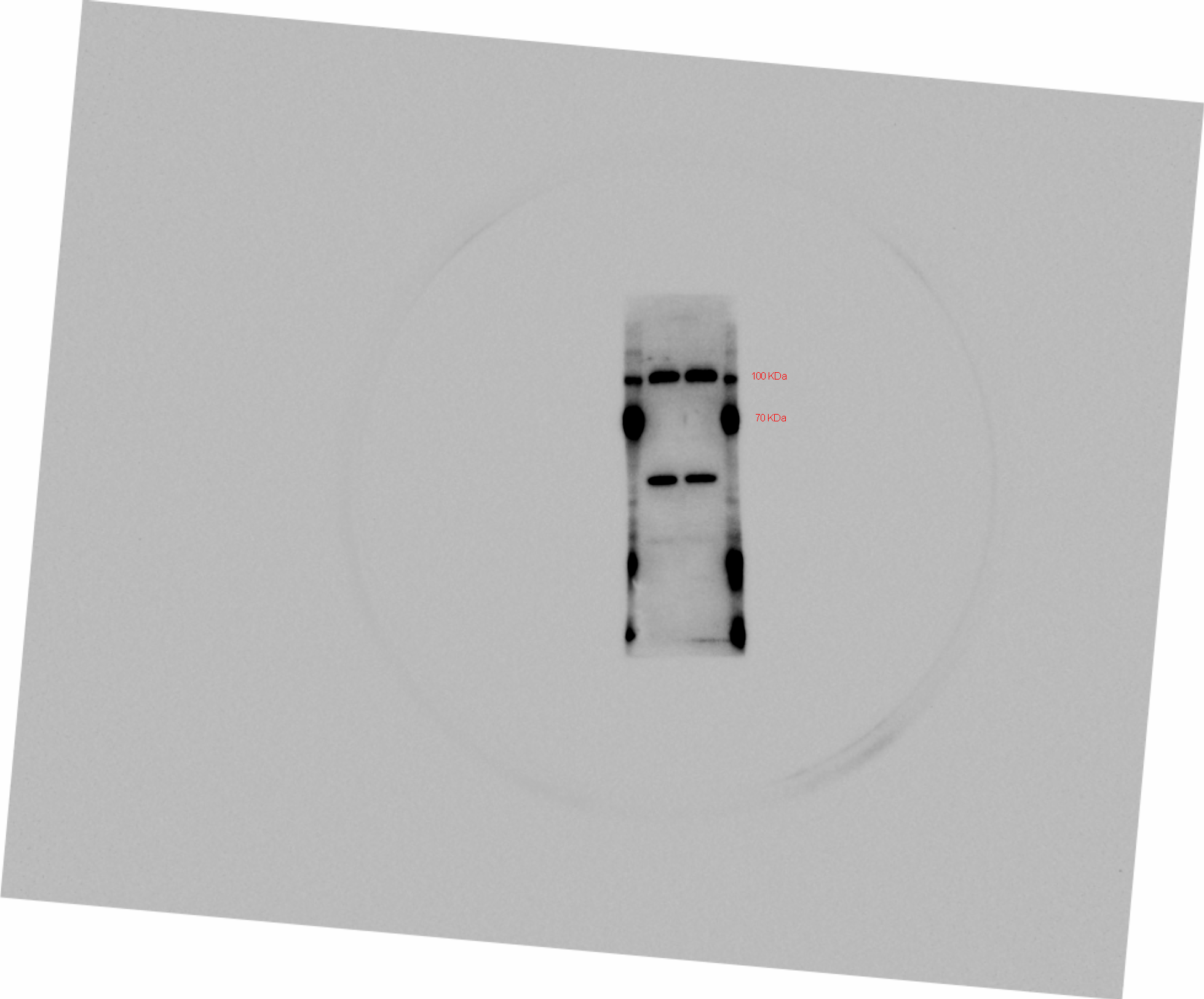

Supplement: Supplementary Figure 3 — Prospects: other regulatory roles of hnRNP A1. (A) Observation of the effect of different concentrations of VPC-80051 on viability in HT22 cells (n = 6 per group). (B) Enrichment of PKM1 and PKM2 mRNA by qPCR after pulling down RNA using hnRNP A1 antibody (n = 6 per group). (C) Observation of the effect of hnRNP A1 overexpression on lactate content of Aβ25–35-induced HT22 cells (n = 3 per group). (D) Observation of the effect of hnRNP A1 overexpression on GSH of Aβ25–35-induced HT22 cells (n = 3 per group). (E) Observation of the effect of hnRNP A1 overexpression on ROS of Aβ25–35-induced HT22 cells (n = 3 per group). (F) To observe the effect of hnRNP A1 overexpression on GSH after the use of glycolysis inhibitor 2-DG (n = 3 per group). (G) To observe the effect of hnRNP A1 overexpression on ROS after the use of glycolysis inhibitor 2-DG (n = 3 per group). [file Data_Sheet_5.zip › FIG3/HK pro(OE-A1)/HK1/original data/wb 2022-12-13 hk4.3.tif]

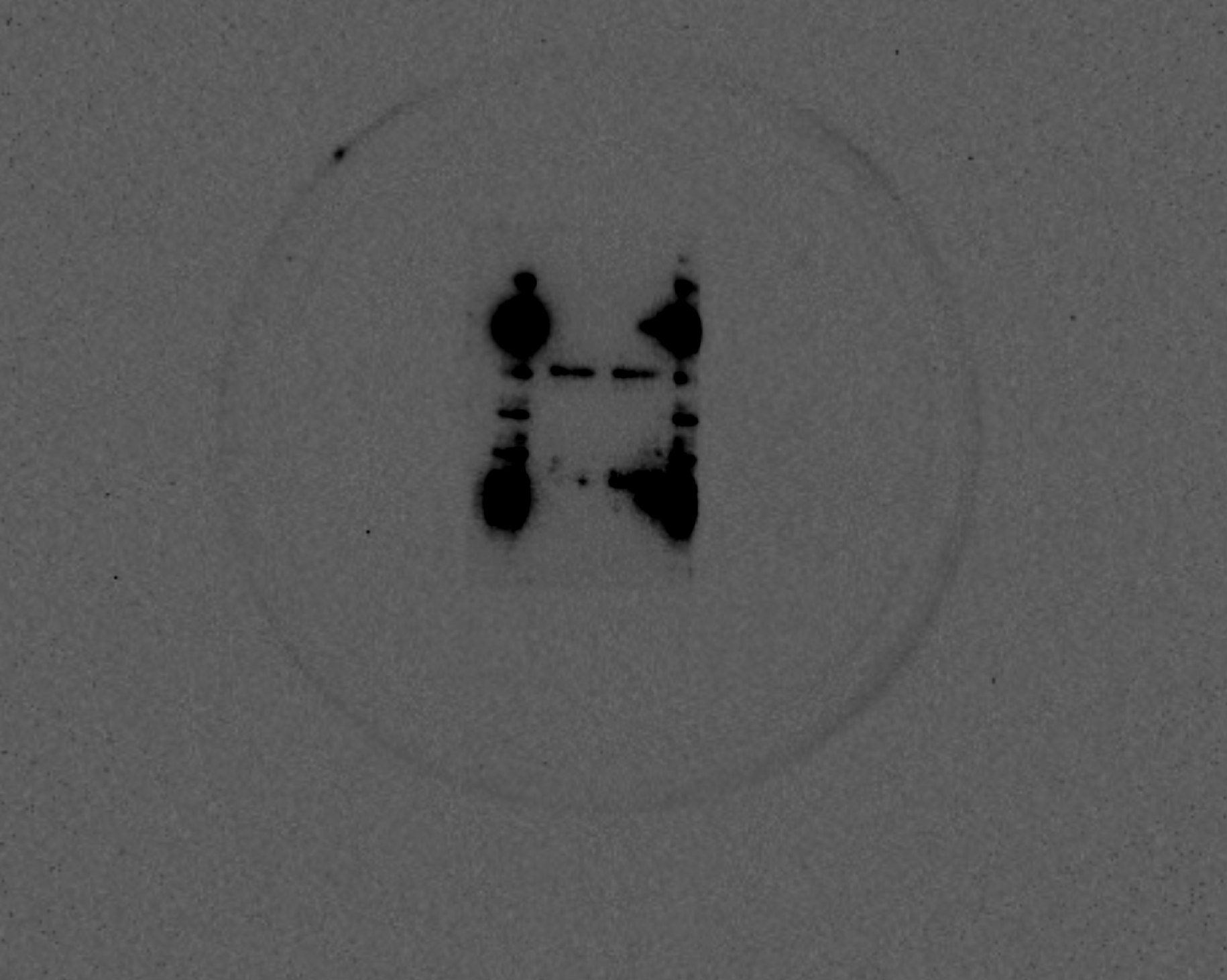

Supplement: Supplementary Figure 3 — Prospects: other regulatory roles of hnRNP A1. (A) Observation of the effect of different concentrations of VPC-80051 on viability in HT22 cells (n = 6 per group). (B) Enrichment of PKM1 and PKM2 mRNA by qPCR after pulling down RNA using hnRNP A1 antibody (n = 6 per group). (C) Observation of the effect of hnRNP A1 overexpression on lactate content of Aβ25–35-induced HT22 cells (n = 3 per group). (D) Observation of the effect of hnRNP A1 overexpression on GSH of Aβ25–35-induced HT22 cells (n = 3 per group). (E) Observation of the effect of hnRNP A1 overexpression on ROS of Aβ25–35-induced HT22 cells (n = 3 per group). (F) To observe the effect of hnRNP A1 overexpression on GSH after the use of glycolysis inhibitor 2-DG (n = 3 per group). (G) To observe the effect of hnRNP A1 overexpression on ROS after the use of glycolysis inhibitor 2-DG (n = 3 per group). [file Data_Sheet_5.zip › FIG3/HK pro(OE-A1)/HK1/original data/wb 2022-12-16 tub 2.1.tif]

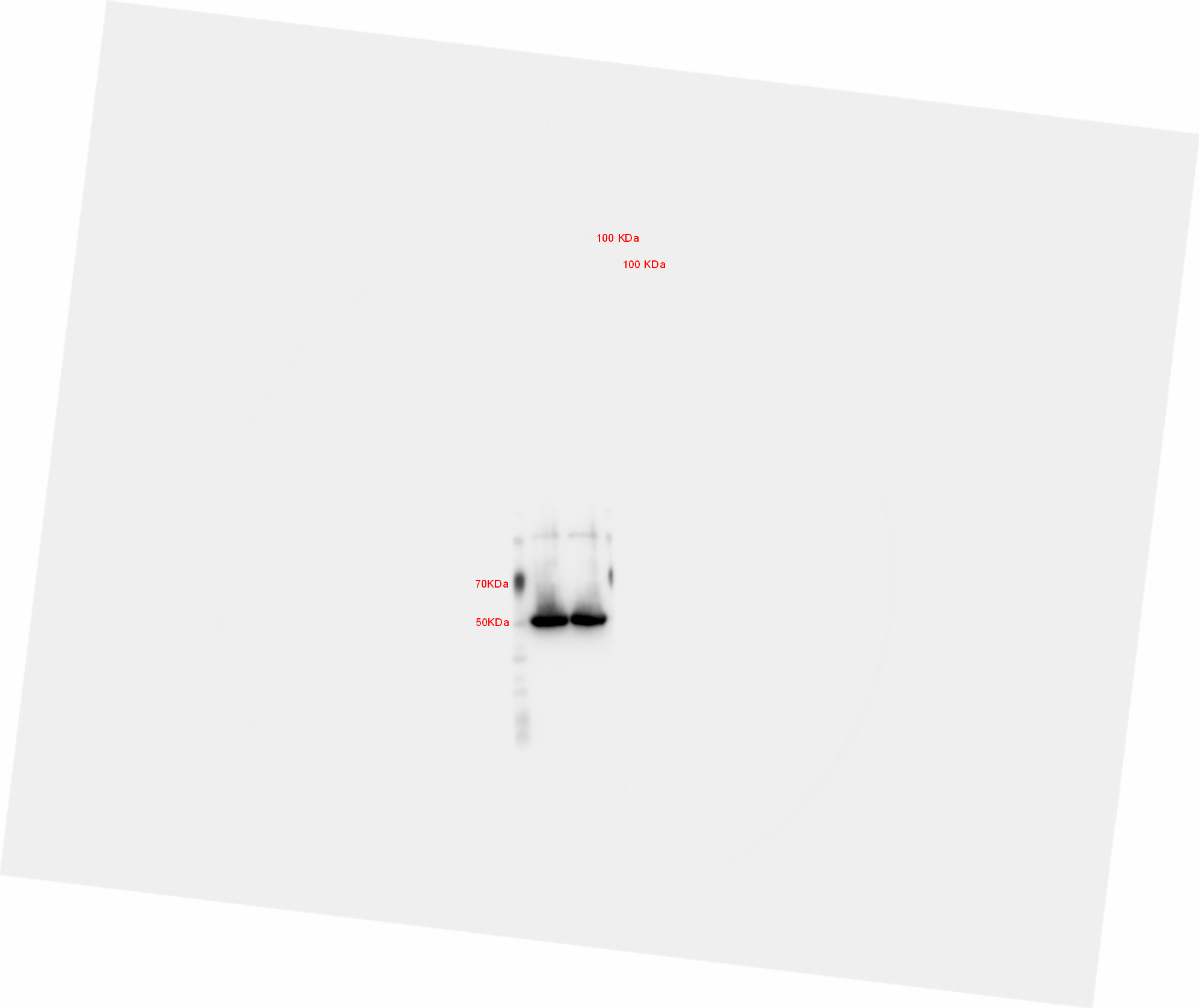

Supplement: Supplementary Figure 3 — Prospects: other regulatory roles of hnRNP A1. (A) Observation of the effect of different concentrations of VPC-80051 on viability in HT22 cells (n = 6 per group). (B) Enrichment of PKM1 and PKM2 mRNA by qPCR after pulling down RNA using hnRNP A1 antibody (n = 6 per group). (C) Observation of the effect of hnRNP A1 overexpression on lactate content of Aβ25–35-induced HT22 cells (n = 3 per group). (D) Observation of the effect of hnRNP A1 overexpression on GSH of Aβ25–35-induced HT22 cells (n = 3 per group). (E) Observation of the effect of hnRNP A1 overexpression on ROS of Aβ25–35-induced HT22 cells (n = 3 per group). (F) To observe the effect of hnRNP A1 overexpression on GSH after the use of glycolysis inhibitor 2-DG (n = 3 per group). (G) To observe the effect of hnRNP A1 overexpression on ROS after the use of glycolysis inhibitor 2-DG (n = 3 per group). [file Data_Sheet_5.zip › FIG3/HK pro(OE-A1)/HK1/original data/wb 2022-12-16 tub 4.2.tif]

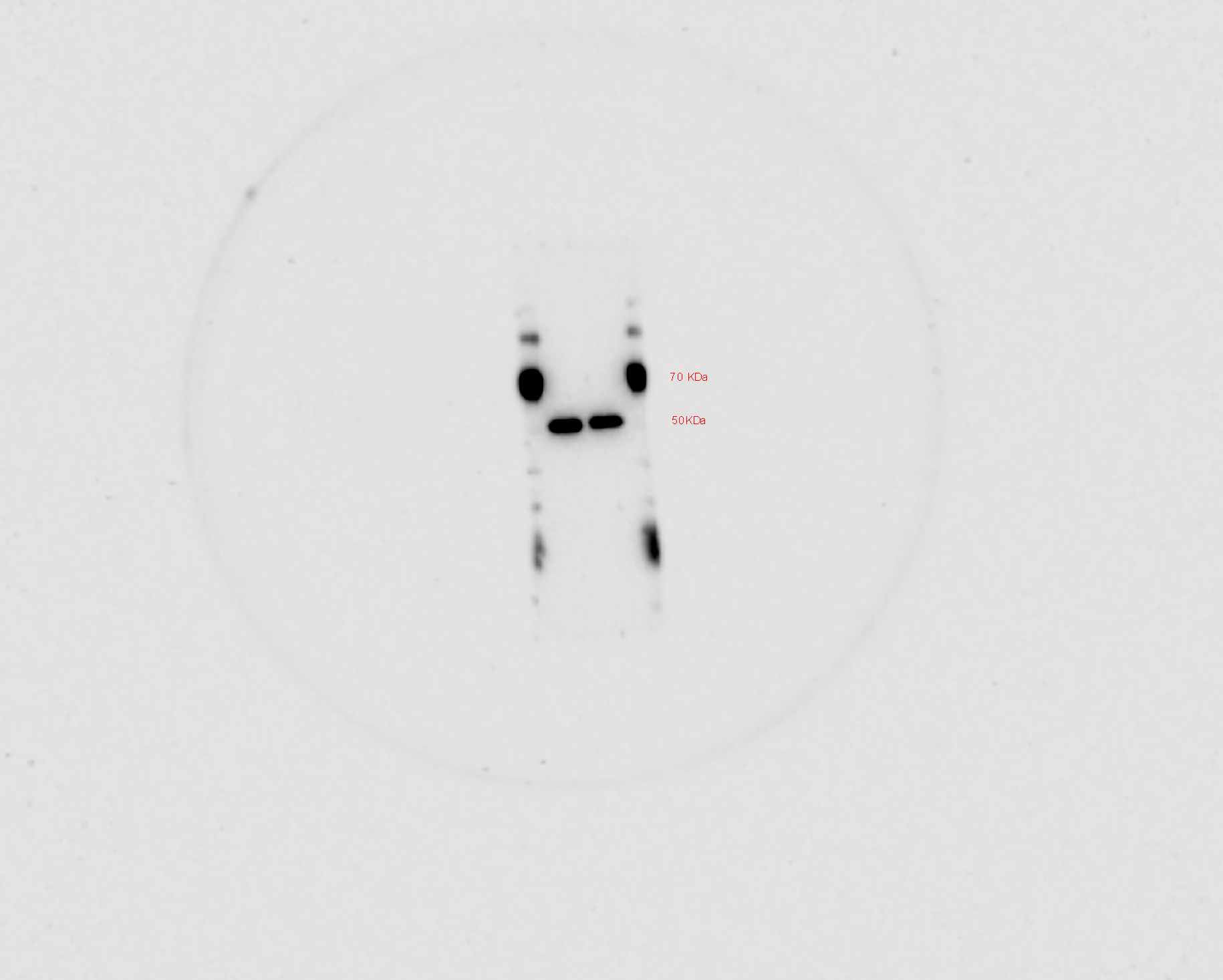

Supplement: Supplementary Figure 3 — Prospects: other regulatory roles of hnRNP A1. (A) Observation of the effect of different concentrations of VPC-80051 on viability in HT22 cells (n = 6 per group). (B) Enrichment of PKM1 and PKM2 mRNA by qPCR after pulling down RNA using hnRNP A1 antibody (n = 6 per group). (C) Observation of the effect of hnRNP A1 overexpression on lactate content of Aβ25–35-induced HT22 cells (n = 3 per group). (D) Observation of the effect of hnRNP A1 overexpression on GSH of Aβ25–35-induced HT22 cells (n = 3 per group). (E) Observation of the effect of hnRNP A1 overexpression on ROS of Aβ25–35-induced HT22 cells (n = 3 per group). (F) To observe the effect of hnRNP A1 overexpression on GSH after the use of glycolysis inhibitor 2-DG (n = 3 per group). (G) To observe the effect of hnRNP A1 overexpression on ROS after the use of glycolysis inhibitor 2-DG (n = 3 per group). [file Data_Sheet_5.zip › FIG3/HK pro(OE-A1)/HK1/original data/wb 2022-12-16 tub 4.3.tif]

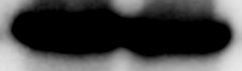

Supplement: Supplementary Figure 3 — Prospects: other regulatory roles of hnRNP A1. (A) Observation of the effect of different concentrations of VPC-80051 on viability in HT22 cells (n = 6 per group). (B) Enrichment of PKM1 and PKM2 mRNA by qPCR after pulling down RNA using hnRNP A1 antibody (n = 6 per group). (C) Observation of the effect of hnRNP A1 overexpression on lactate content of Aβ25–35-induced HT22 cells (n = 3 per group). (D) Observation of the effect of hnRNP A1 overexpression on GSH of Aβ25–35-induced HT22 cells (n = 3 per group). (E) Observation of the effect of hnRNP A1 overexpression on ROS of Aβ25–35-induced HT22 cells (n = 3 per group). (F) To observe the effect of hnRNP A1 overexpression on GSH after the use of glycolysis inhibitor 2-DG (n = 3 per group). (G) To observe the effect of hnRNP A1 overexpression on ROS after the use of glycolysis inhibitor 2-DG (n = 3 per group). [file Data_Sheet_5.zip › FIG3/HK pro(OE-A1)/HK1/wb 2022-10-20 jxh 5'4tub.png]

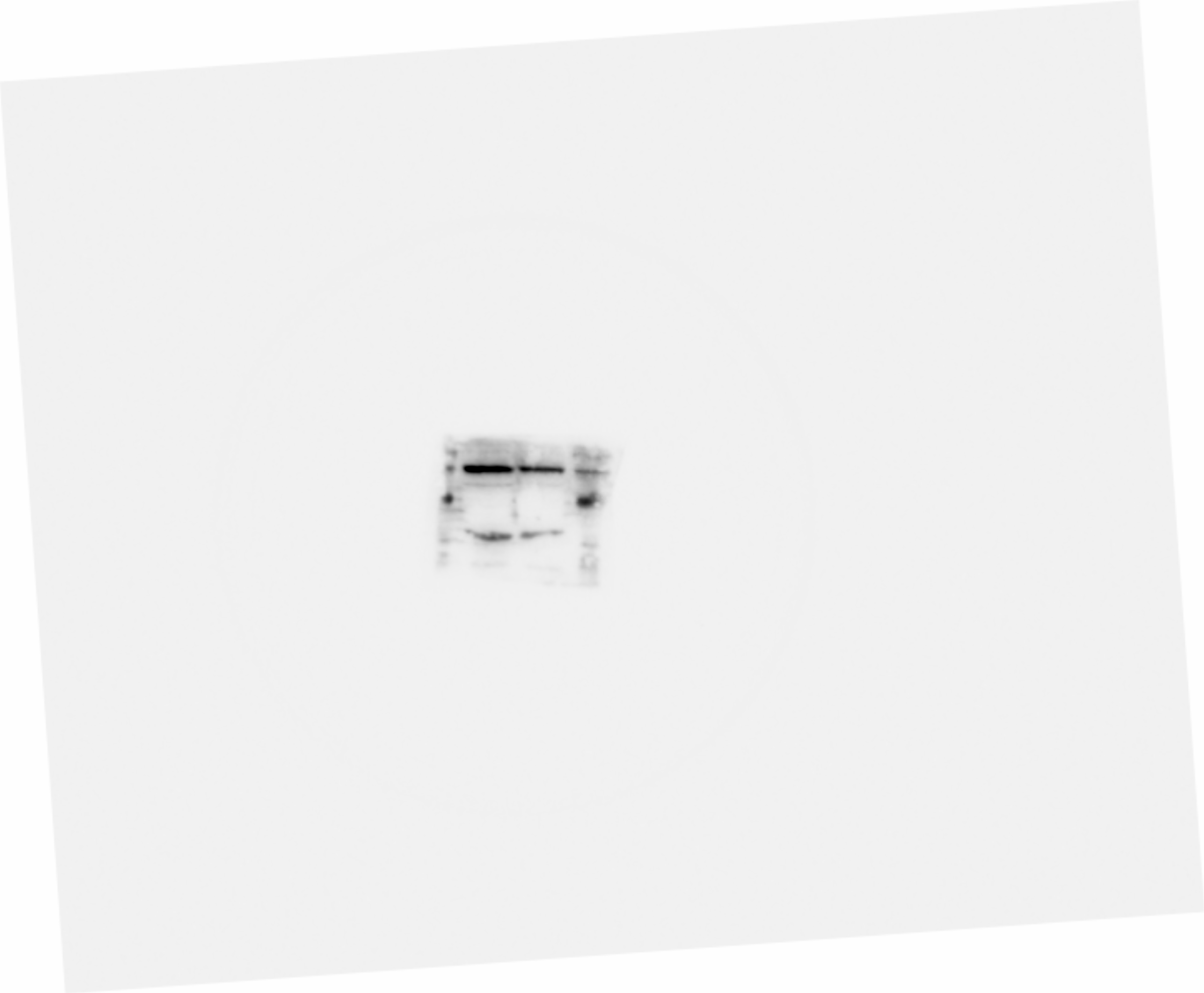

Supplement: Supplementary Figure 3 — Prospects: other regulatory roles of hnRNP A1. (A) Observation of the effect of different concentrations of VPC-80051 on viability in HT22 cells (n = 6 per group). (B) Enrichment of PKM1 and PKM2 mRNA by qPCR after pulling down RNA using hnRNP A1 antibody (n = 6 per group). (C) Observation of the effect of hnRNP A1 overexpression on lactate content of Aβ25–35-induced HT22 cells (n = 3 per group). (D) Observation of the effect of hnRNP A1 overexpression on GSH of Aβ25–35-induced HT22 cells (n = 3 per group). (E) Observation of the effect of hnRNP A1 overexpression on ROS of Aβ25–35-induced HT22 cells (n = 3 per group). (F) To observe the effect of hnRNP A1 overexpression on GSH after the use of glycolysis inhibitor 2-DG (n = 3 per group). (G) To observe the effect of hnRNP A1 overexpression on ROS after the use of glycolysis inhibitor 2-DG (n = 3 per group). [file Data_Sheet_5.zip › FIG3/hk(VPC)/original data/wb 2022-10-14 4'3hk.tif]

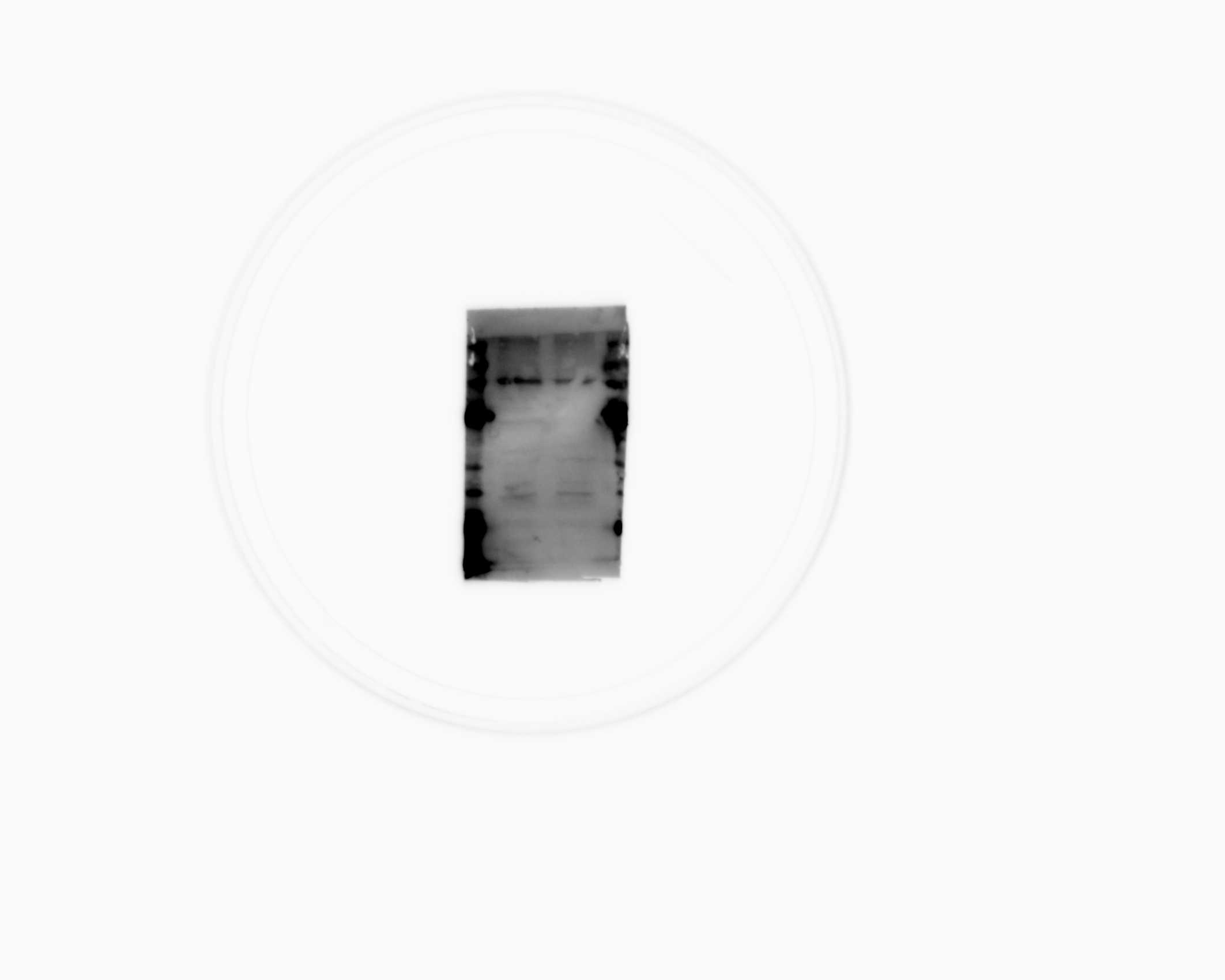

Supplement: Supplementary Figure 3 — Prospects: other regulatory roles of hnRNP A1. (A) Observation of the effect of different concentrations of VPC-80051 on viability in HT22 cells (n = 6 per group). (B) Enrichment of PKM1 and PKM2 mRNA by qPCR after pulling down RNA using hnRNP A1 antibody (n = 6 per group). (C) Observation of the effect of hnRNP A1 overexpression on lactate content of Aβ25–35-induced HT22 cells (n = 3 per group). (D) Observation of the effect of hnRNP A1 overexpression on GSH of Aβ25–35-induced HT22 cells (n = 3 per group). (E) Observation of the effect of hnRNP A1 overexpression on ROS of Aβ25–35-induced HT22 cells (n = 3 per group). (F) To observe the effect of hnRNP A1 overexpression on GSH after the use of glycolysis inhibitor 2-DG (n = 3 per group). (G) To observe the effect of hnRNP A1 overexpression on ROS after the use of glycolysis inhibitor 2-DG (n = 3 per group). [file Data_Sheet_5.zip › FIG3/hk(VPC)/original data/wb 2022-10-14 1'1hk.tif]

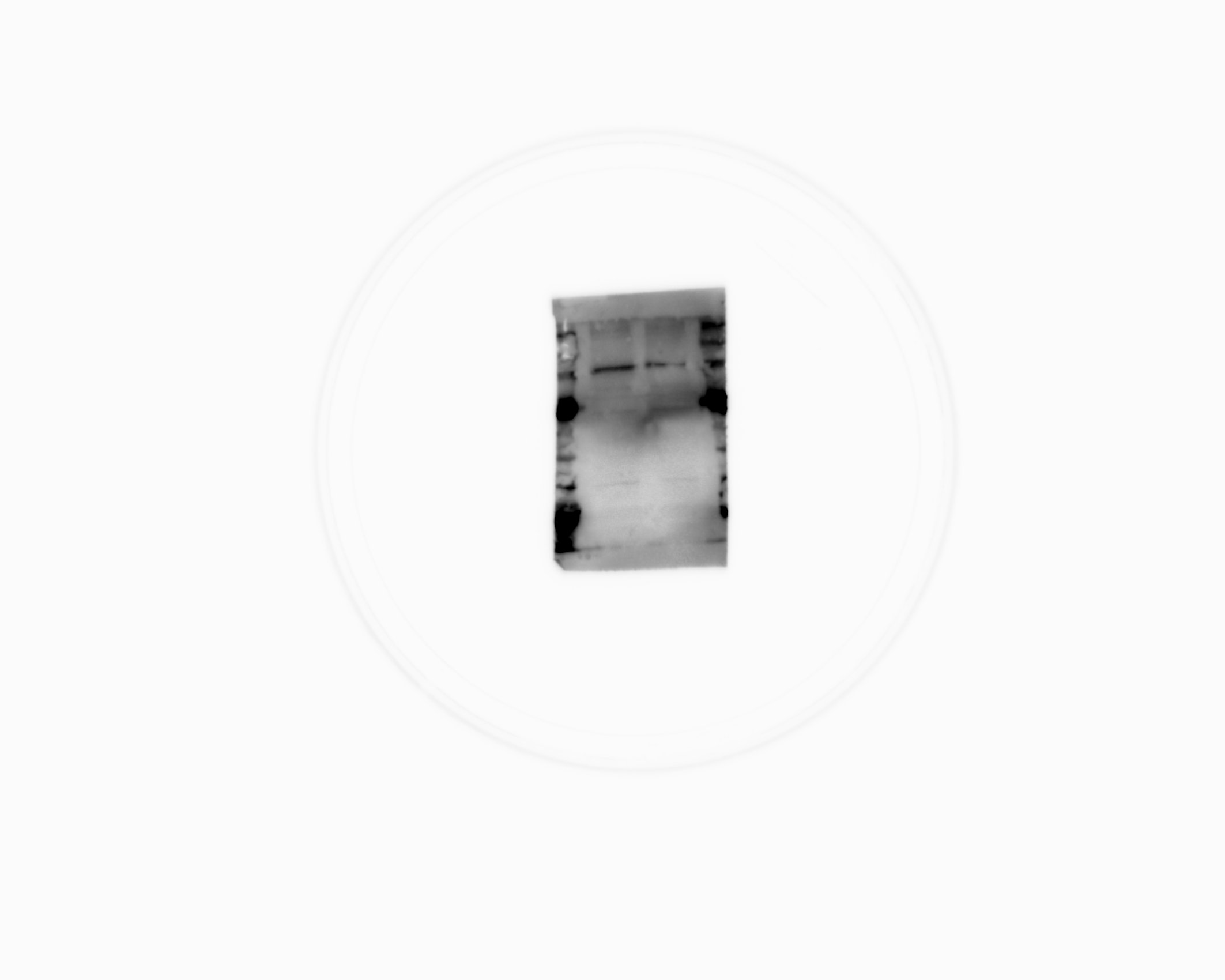

Supplement: Supplementary Figure 3 — Prospects: other regulatory roles of hnRNP A1. (A) Observation of the effect of different concentrations of VPC-80051 on viability in HT22 cells (n = 6 per group). (B) Enrichment of PKM1 and PKM2 mRNA by qPCR after pulling down RNA using hnRNP A1 antibody (n = 6 per group). (C) Observation of the effect of hnRNP A1 overexpression on lactate content of Aβ25–35-induced HT22 cells (n = 3 per group). (D) Observation of the effect of hnRNP A1 overexpression on GSH of Aβ25–35-induced HT22 cells (n = 3 per group). (E) Observation of the effect of hnRNP A1 overexpression on ROS of Aβ25–35-induced HT22 cells (n = 3 per group). (F) To observe the effect of hnRNP A1 overexpression on GSH after the use of glycolysis inhibitor 2-DG (n = 3 per group). (G) To observe the effect of hnRNP A1 overexpression on ROS after the use of glycolysis inhibitor 2-DG (n = 3 per group). [file Data_Sheet_5.zip › FIG3/hk(VPC)/original data/wb 2022-10-14 1'2 hk.tif]

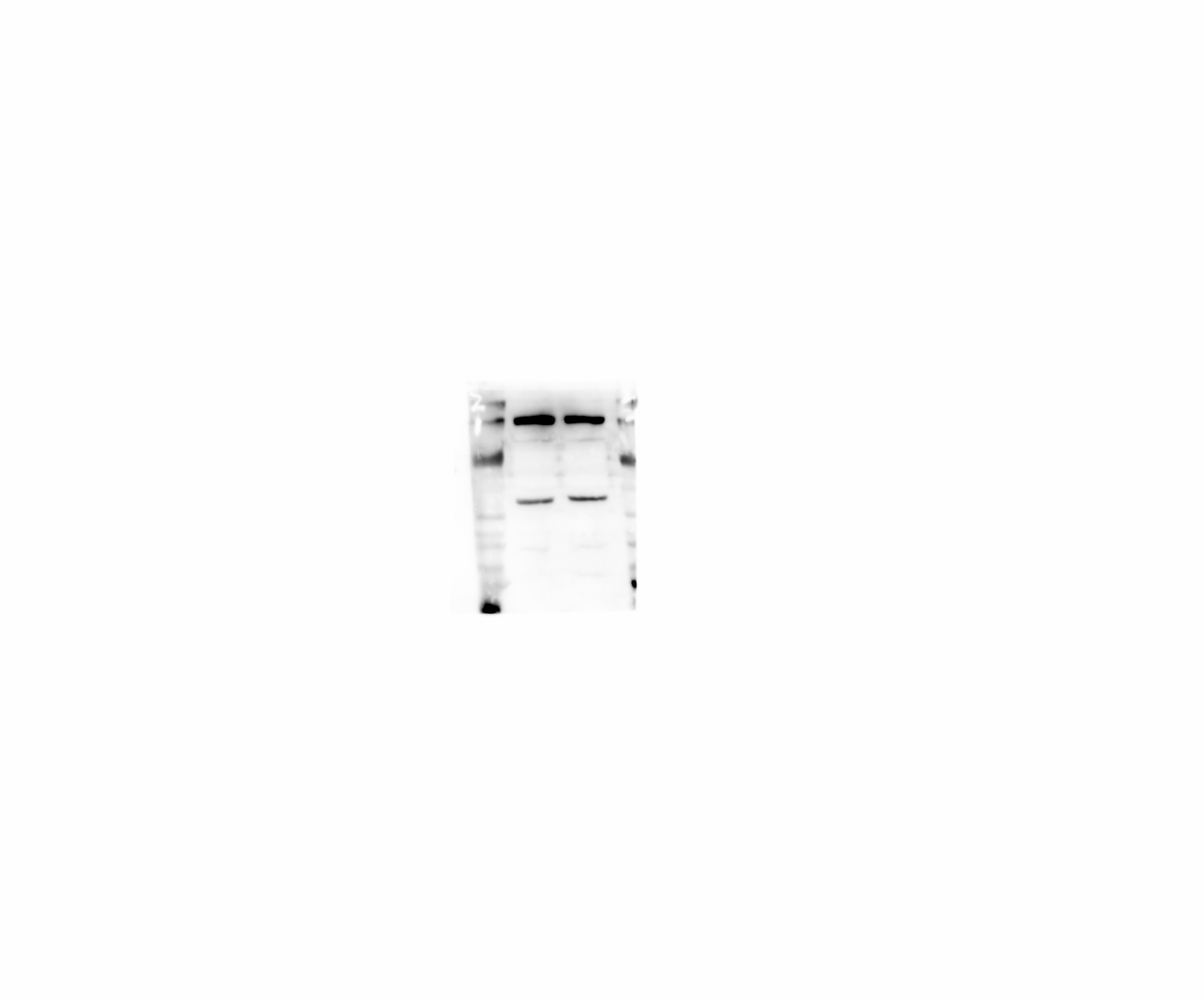

Supplement: Supplementary Figure 3 — Prospects: other regulatory roles of hnRNP A1. (A) Observation of the effect of different concentrations of VPC-80051 on viability in HT22 cells (n = 6 per group). (B) Enrichment of PKM1 and PKM2 mRNA by qPCR after pulling down RNA using hnRNP A1 antibody (n = 6 per group). (C) Observation of the effect of hnRNP A1 overexpression on lactate content of Aβ25–35-induced HT22 cells (n = 3 per group). (D) Observation of the effect of hnRNP A1 overexpression on GSH of Aβ25–35-induced HT22 cells (n = 3 per group). (E) Observation of the effect of hnRNP A1 overexpression on ROS of Aβ25–35-induced HT22 cells (n = 3 per group). (F) To observe the effect of hnRNP A1 overexpression on GSH after the use of glycolysis inhibitor 2-DG (n = 3 per group). (G) To observe the effect of hnRNP A1 overexpression on ROS after the use of glycolysis inhibitor 2-DG (n = 3 per group). [file Data_Sheet_5.zip › FIG3/hk(VPC)/original data/wb 2022-10-14 2'1hk.tif]

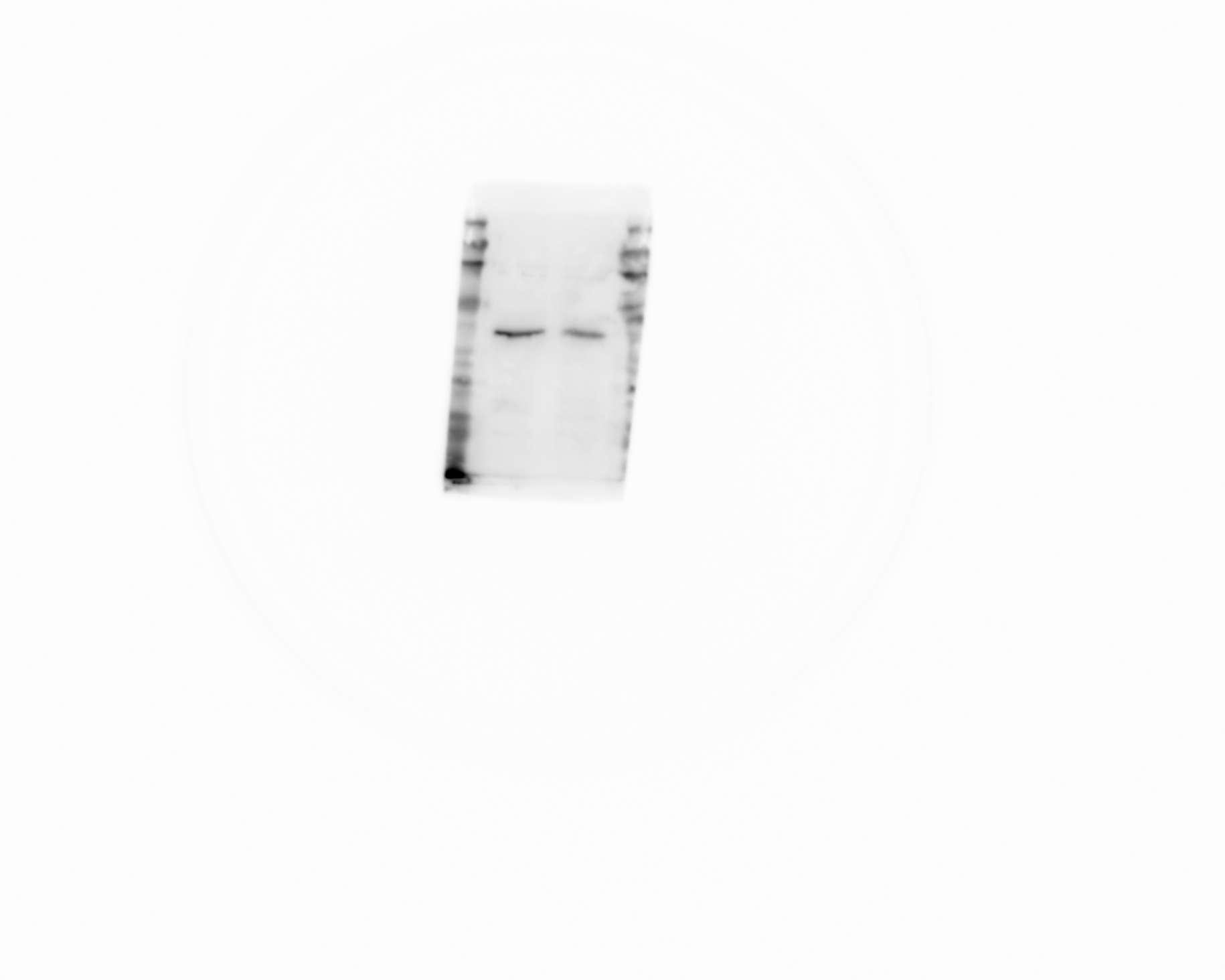

Supplement: Supplementary Figure 3 — Prospects: other regulatory roles of hnRNP A1. (A) Observation of the effect of different concentrations of VPC-80051 on viability in HT22 cells (n = 6 per group). (B) Enrichment of PKM1 and PKM2 mRNA by qPCR after pulling down RNA using hnRNP A1 antibody (n = 6 per group). (C) Observation of the effect of hnRNP A1 overexpression on lactate content of Aβ25–35-induced HT22 cells (n = 3 per group). (D) Observation of the effect of hnRNP A1 overexpression on GSH of Aβ25–35-induced HT22 cells (n = 3 per group). (E) Observation of the effect of hnRNP A1 overexpression on ROS of Aβ25–35-induced HT22 cells (n = 3 per group). (F) To observe the effect of hnRNP A1 overexpression on GSH after the use of glycolysis inhibitor 2-DG (n = 3 per group). (G) To observe the effect of hnRNP A1 overexpression on ROS after the use of glycolysis inhibitor 2-DG (n = 3 per group). [file Data_Sheet_5.zip › FIG3/hk(VPC)/original data/wb 2022-10-16 1'1tub.tif]

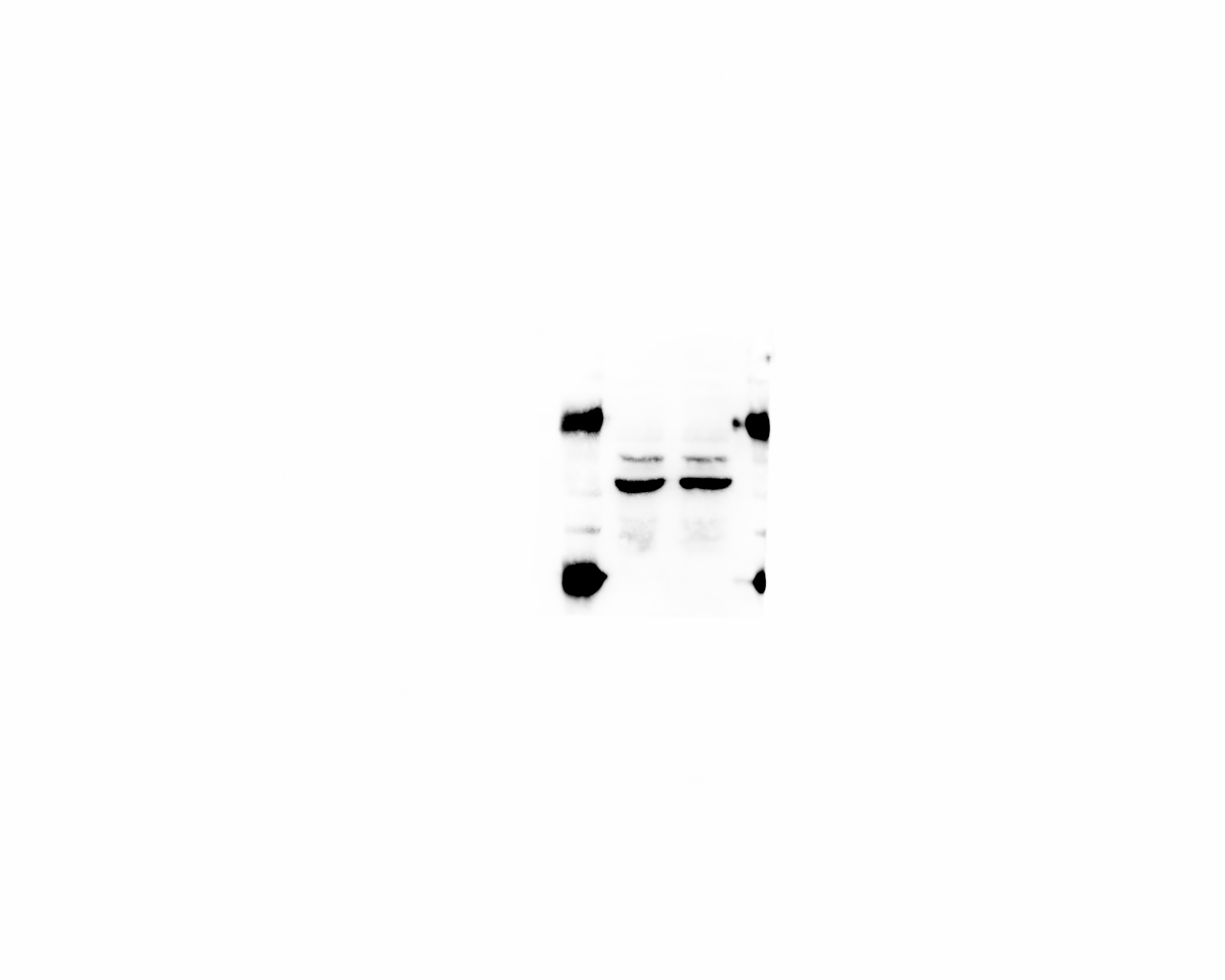

Supplement: Supplementary Figure 3 — Prospects: other regulatory roles of hnRNP A1. (A) Observation of the effect of different concentrations of VPC-80051 on viability in HT22 cells (n = 6 per group). (B) Enrichment of PKM1 and PKM2 mRNA by qPCR after pulling down RNA using hnRNP A1 antibody (n = 6 per group). (C) Observation of the effect of hnRNP A1 overexpression on lactate content of Aβ25–35-induced HT22 cells (n = 3 per group). (D) Observation of the effect of hnRNP A1 overexpression on GSH of Aβ25–35-induced HT22 cells (n = 3 per group). (E) Observation of the effect of hnRNP A1 overexpression on ROS of Aβ25–35-induced HT22 cells (n = 3 per group). (F) To observe the effect of hnRNP A1 overexpression on GSH after the use of glycolysis inhibitor 2-DG (n = 3 per group). (G) To observe the effect of hnRNP A1 overexpression on ROS after the use of glycolysis inhibitor 2-DG (n = 3 per group). [file Data_Sheet_5.zip › FIG3/hk(VPC)/original data/wb 2022-10-16 2'1tub.tif]

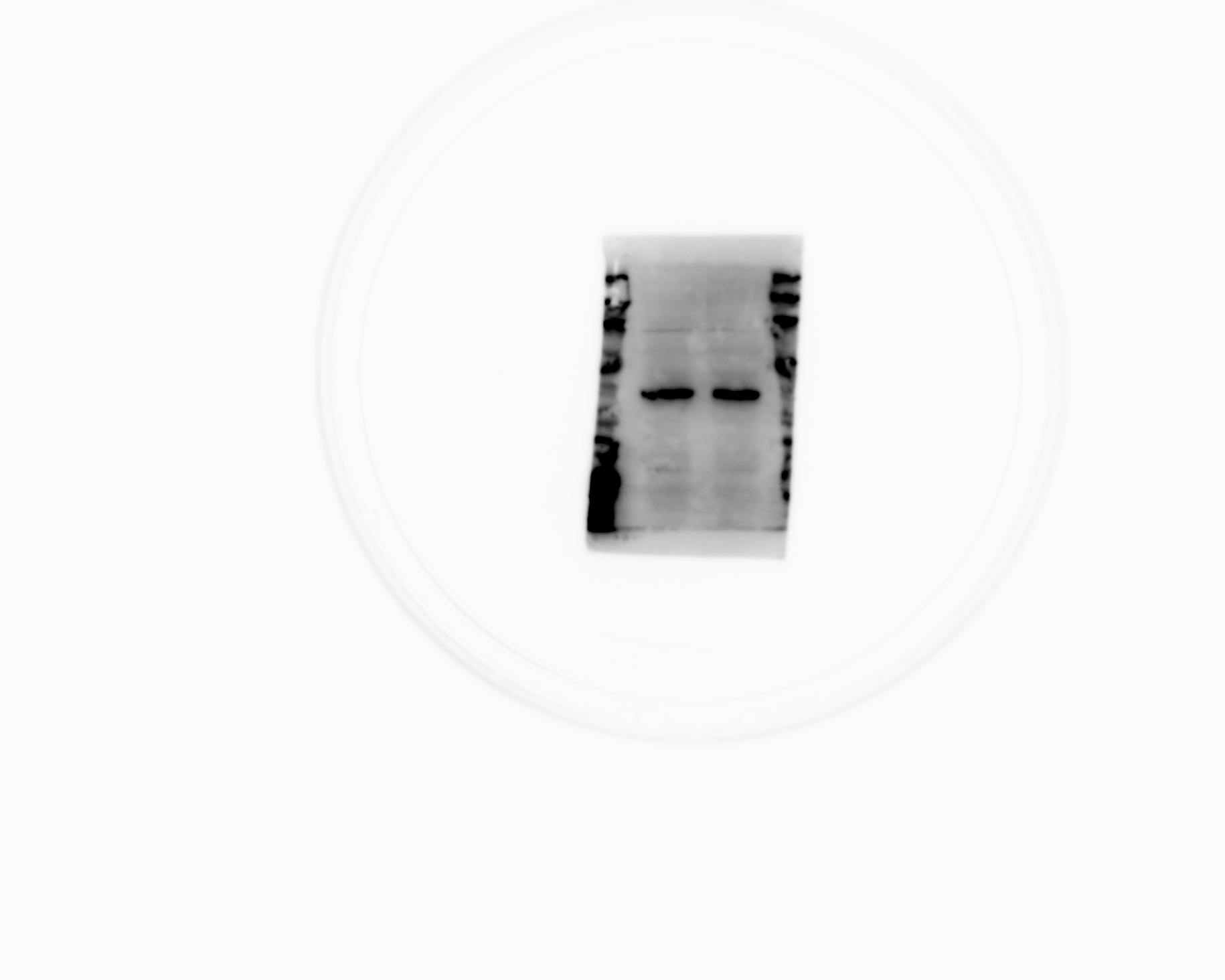

Supplement: Supplementary Figure 3 — Prospects: other regulatory roles of hnRNP A1. (A) Observation of the effect of different concentrations of VPC-80051 on viability in HT22 cells (n = 6 per group). (B) Enrichment of PKM1 and PKM2 mRNA by qPCR after pulling down RNA using hnRNP A1 antibody (n = 6 per group). (C) Observation of the effect of hnRNP A1 overexpression on lactate content of Aβ25–35-induced HT22 cells (n = 3 per group). (D) Observation of the effect of hnRNP A1 overexpression on GSH of Aβ25–35-induced HT22 cells (n = 3 per group). (E) Observation of the effect of hnRNP A1 overexpression on ROS of Aβ25–35-induced HT22 cells (n = 3 per group). (F) To observe the effect of hnRNP A1 overexpression on GSH after the use of glycolysis inhibitor 2-DG (n = 3 per group). (G) To observe the effect of hnRNP A1 overexpression on ROS after the use of glycolysis inhibitor 2-DG (n = 3 per group). [file Data_Sheet_5.zip › FIG3/hk(VPC)/original data/wb 2022-10-16 1'2tub.tif]

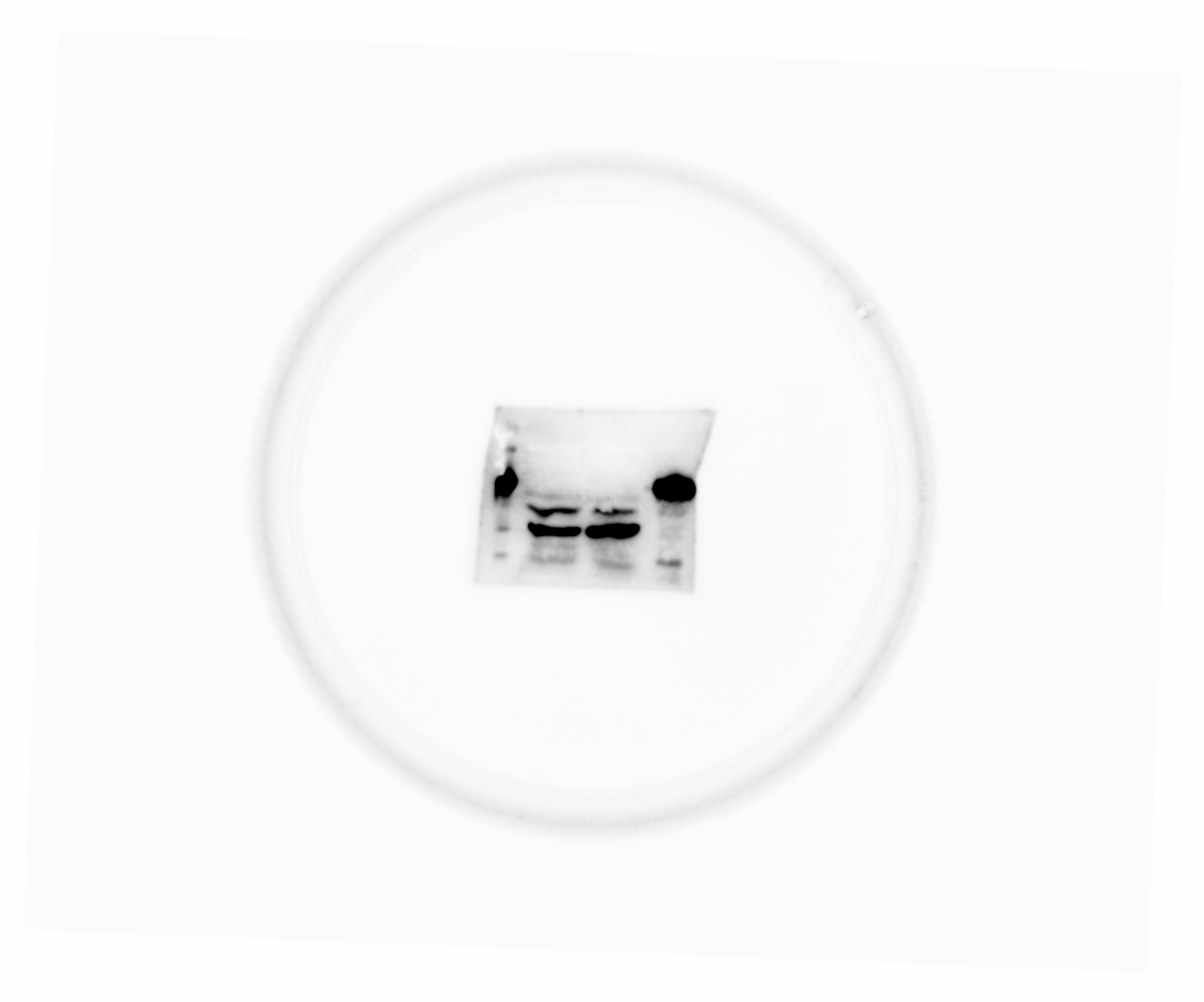

Supplement: Supplementary Figure 3 — Prospects: other regulatory roles of hnRNP A1. (A) Observation of the effect of different concentrations of VPC-80051 on viability in HT22 cells (n = 6 per group). (B) Enrichment of PKM1 and PKM2 mRNA by qPCR after pulling down RNA using hnRNP A1 antibody (n = 6 per group). (C) Observation of the effect of hnRNP A1 overexpression on lactate content of Aβ25–35-induced HT22 cells (n = 3 per group). (D) Observation of the effect of hnRNP A1 overexpression on GSH of Aβ25–35-induced HT22 cells (n = 3 per group). (E) Observation of the effect of hnRNP A1 overexpression on ROS of Aβ25–35-induced HT22 cells (n = 3 per group). (F) To observe the effect of hnRNP A1 overexpression on GSH after the use of glycolysis inhibitor 2-DG (n = 3 per group). (G) To observe the effect of hnRNP A1 overexpression on ROS after the use of glycolysis inhibitor 2-DG (n = 3 per group). [file Data_Sheet_5.zip › FIG3/hk(VPC)/original data/wb 2022-10-16 4'3 tub.tif]

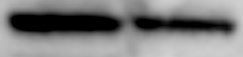

Supplement: Supplementary Figure 3 — Prospects: other regulatory roles of hnRNP A1. (A) Observation of the effect of different concentrations of VPC-80051 on viability in HT22 cells (n = 6 per group). (B) Enrichment of PKM1 and PKM2 mRNA by qPCR after pulling down RNA using hnRNP A1 antibody (n = 6 per group). (C) Observation of the effect of hnRNP A1 overexpression on lactate content of Aβ25–35-induced HT22 cells (n = 3 per group). (D) Observation of the effect of hnRNP A1 overexpression on GSH of Aβ25–35-induced HT22 cells (n = 3 per group). (E) Observation of the effect of hnRNP A1 overexpression on ROS of Aβ25–35-induced HT22 cells (n = 3 per group). (F) To observe the effect of hnRNP A1 overexpression on GSH after the use of glycolysis inhibitor 2-DG (n = 3 per group). (G) To observe the effect of hnRNP A1 overexpression on ROS after the use of glycolysis inhibitor 2-DG (n = 3 per group). [file Data_Sheet_5.zip › FIG3/hk(VPC)/wb 2022-10-14 jxh 4'3hk.png]

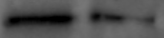

Supplement: Supplementary Figure 3 — Prospects: other regulatory roles of hnRNP A1. (A) Observation of the effect of different concentrations of VPC-80051 on viability in HT22 cells (n = 6 per group). (B) Enrichment of PKM1 and PKM2 mRNA by qPCR after pulling down RNA using hnRNP A1 antibody (n = 6 per group). (C) Observation of the effect of hnRNP A1 overexpression on lactate content of Aβ25–35-induced HT22 cells (n = 3 per group). (D) Observation of the effect of hnRNP A1 overexpression on GSH of Aβ25–35-induced HT22 cells (n = 3 per group). (E) Observation of the effect of hnRNP A1 overexpression on ROS of Aβ25–35-induced HT22 cells (n = 3 per group). (F) To observe the effect of hnRNP A1 overexpression on GSH after the use of glycolysis inhibitor 2-DG (n = 3 per group). (G) To observe the effect of hnRNP A1 overexpression on ROS after the use of glycolysis inhibitor 2-DG (n = 3 per group). [file Data_Sheet_5.zip › FIG3/hk(VPC)/wb 2022-10-14 jxh 1'1hk.png]

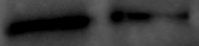

Supplement: Supplementary Figure 3 — Prospects: other regulatory roles of hnRNP A1. (A) Observation of the effect of different concentrations of VPC-80051 on viability in HT22 cells (n = 6 per group). (B) Enrichment of PKM1 and PKM2 mRNA by qPCR after pulling down RNA using hnRNP A1 antibody (n = 6 per group). (C) Observation of the effect of hnRNP A1 overexpression on lactate content of Aβ25–35-induced HT22 cells (n = 3 per group). (D) Observation of the effect of hnRNP A1 overexpression on GSH of Aβ25–35-induced HT22 cells (n = 3 per group). (E) Observation of the effect of hnRNP A1 overexpression on ROS of Aβ25–35-induced HT22 cells (n = 3 per group). (F) To observe the effect of hnRNP A1 overexpression on GSH after the use of glycolysis inhibitor 2-DG (n = 3 per group). (G) To observe the effect of hnRNP A1 overexpression on ROS after the use of glycolysis inhibitor 2-DG (n = 3 per group). [file Data_Sheet_5.zip › FIG3/hk(VPC)/wb 2022-10-14 jxh 1'2 hk.png]

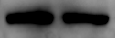

Supplement: Supplementary Figure 3 — Prospects: other regulatory roles of hnRNP A1. (A) Observation of the effect of different concentrations of VPC-80051 on viability in HT22 cells (n = 6 per group). (B) Enrichment of PKM1 and PKM2 mRNA by qPCR after pulling down RNA using hnRNP A1 antibody (n = 6 per group). (C) Observation of the effect of hnRNP A1 overexpression on lactate content of Aβ25–35-induced HT22 cells (n = 3 per group). (D) Observation of the effect of hnRNP A1 overexpression on GSH of Aβ25–35-induced HT22 cells (n = 3 per group). (E) Observation of the effect of hnRNP A1 overexpression on ROS of Aβ25–35-induced HT22 cells (n = 3 per group). (F) To observe the effect of hnRNP A1 overexpression on GSH after the use of glycolysis inhibitor 2-DG (n = 3 per group). (G) To observe the effect of hnRNP A1 overexpression on ROS after the use of glycolysis inhibitor 2-DG (n = 3 per group). [file Data_Sheet_5.zip › FIG3/hk(VPC)/wb 2022-10-14 jxh 2'1hk.png]

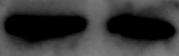

Supplement: Supplementary Figure 3 — Prospects: other regulatory roles of hnRNP A1. (A) Observation of the effect of different concentrations of VPC-80051 on viability in HT22 cells (n = 6 per group). (B) Enrichment of PKM1 and PKM2 mRNA by qPCR after pulling down RNA using hnRNP A1 antibody (n = 6 per group). (C) Observation of the effect of hnRNP A1 overexpression on lactate content of Aβ25–35-induced HT22 cells (n = 3 per group). (D) Observation of the effect of hnRNP A1 overexpression on GSH of Aβ25–35-induced HT22 cells (n = 3 per group). (E) Observation of the effect of hnRNP A1 overexpression on ROS of Aβ25–35-induced HT22 cells (n = 3 per group). (F) To observe the effect of hnRNP A1 overexpression on GSH after the use of glycolysis inhibitor 2-DG (n = 3 per group). (G) To observe the effect of hnRNP A1 overexpression on ROS after the use of glycolysis inhibitor 2-DG (n = 3 per group). [file Data_Sheet_5.zip › FIG3/hk(VPC)/wb 2022-10-16 jxh 1'1tub.png]

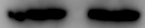

Supplement: Supplementary Figure 3 — Prospects: other regulatory roles of hnRNP A1. (A) Observation of the effect of different concentrations of VPC-80051 on viability in HT22 cells (n = 6 per group). (B) Enrichment of PKM1 and PKM2 mRNA by qPCR after pulling down RNA using hnRNP A1 antibody (n = 6 per group). (C) Observation of the effect of hnRNP A1 overexpression on lactate content of Aβ25–35-induced HT22 cells (n = 3 per group). (D) Observation of the effect of hnRNP A1 overexpression on GSH of Aβ25–35-induced HT22 cells (n = 3 per group). (E) Observation of the effect of hnRNP A1 overexpression on ROS of Aβ25–35-induced HT22 cells (n = 3 per group). (F) To observe the effect of hnRNP A1 overexpression on GSH after the use of glycolysis inhibitor 2-DG (n = 3 per group). (G) To observe the effect of hnRNP A1 overexpression on ROS after the use of glycolysis inhibitor 2-DG (n = 3 per group). [file Data_Sheet_5.zip › FIG3/hk(VPC)/wb 2022-10-16 jxh 1'2tub.png]

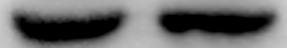

Supplement: Supplementary Figure 3 — Prospects: other regulatory roles of hnRNP A1. (A) Observation of the effect of different concentrations of VPC-80051 on viability in HT22 cells (n = 6 per group). (B) Enrichment of PKM1 and PKM2 mRNA by qPCR after pulling down RNA using hnRNP A1 antibody (n = 6 per group). (C) Observation of the effect of hnRNP A1 overexpression on lactate content of Aβ25–35-induced HT22 cells (n = 3 per group). (D) Observation of the effect of hnRNP A1 overexpression on GSH of Aβ25–35-induced HT22 cells (n = 3 per group). (E) Observation of the effect of hnRNP A1 overexpression on ROS of Aβ25–35-induced HT22 cells (n = 3 per group). (F) To observe the effect of hnRNP A1 overexpression on GSH after the use of glycolysis inhibitor 2-DG (n = 3 per group). (G) To observe the effect of hnRNP A1 overexpression on ROS after the use of glycolysis inhibitor 2-DG (n = 3 per group). [file Data_Sheet_5.zip › FIG3/hk(VPC)/wb 2022-10-16 jxh 2'1tub.png]

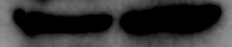

Supplement: Supplementary Figure 3 — Prospects: other regulatory roles of hnRNP A1. (A) Observation of the effect of different concentrations of VPC-80051 on viability in HT22 cells (n = 6 per group). (B) Enrichment of PKM1 and PKM2 mRNA by qPCR after pulling down RNA using hnRNP A1 antibody (n = 6 per group). (C) Observation of the effect of hnRNP A1 overexpression on lactate content of Aβ25–35-induced HT22 cells (n = 3 per group). (D) Observation of the effect of hnRNP A1 overexpression on GSH of Aβ25–35-induced HT22 cells (n = 3 per group). (E) Observation of the effect of hnRNP A1 overexpression on ROS of Aβ25–35-induced HT22 cells (n = 3 per group). (F) To observe the effect of hnRNP A1 overexpression on GSH after the use of glycolysis inhibitor 2-DG (n = 3 per group). (G) To observe the effect of hnRNP A1 overexpression on ROS after the use of glycolysis inhibitor 2-DG (n = 3 per group). [file Data_Sheet_5.zip › FIG3/hk(VPC)/wb 2022-10-16 jxh 4'3 tub.png]

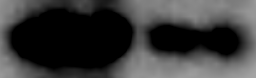

Supplement: Supplementary file 4 [file Data_Sheet_3.zip › Primary hippocampal neurons hnrnp A1/2022-10-20 jxh 7'4a.png]

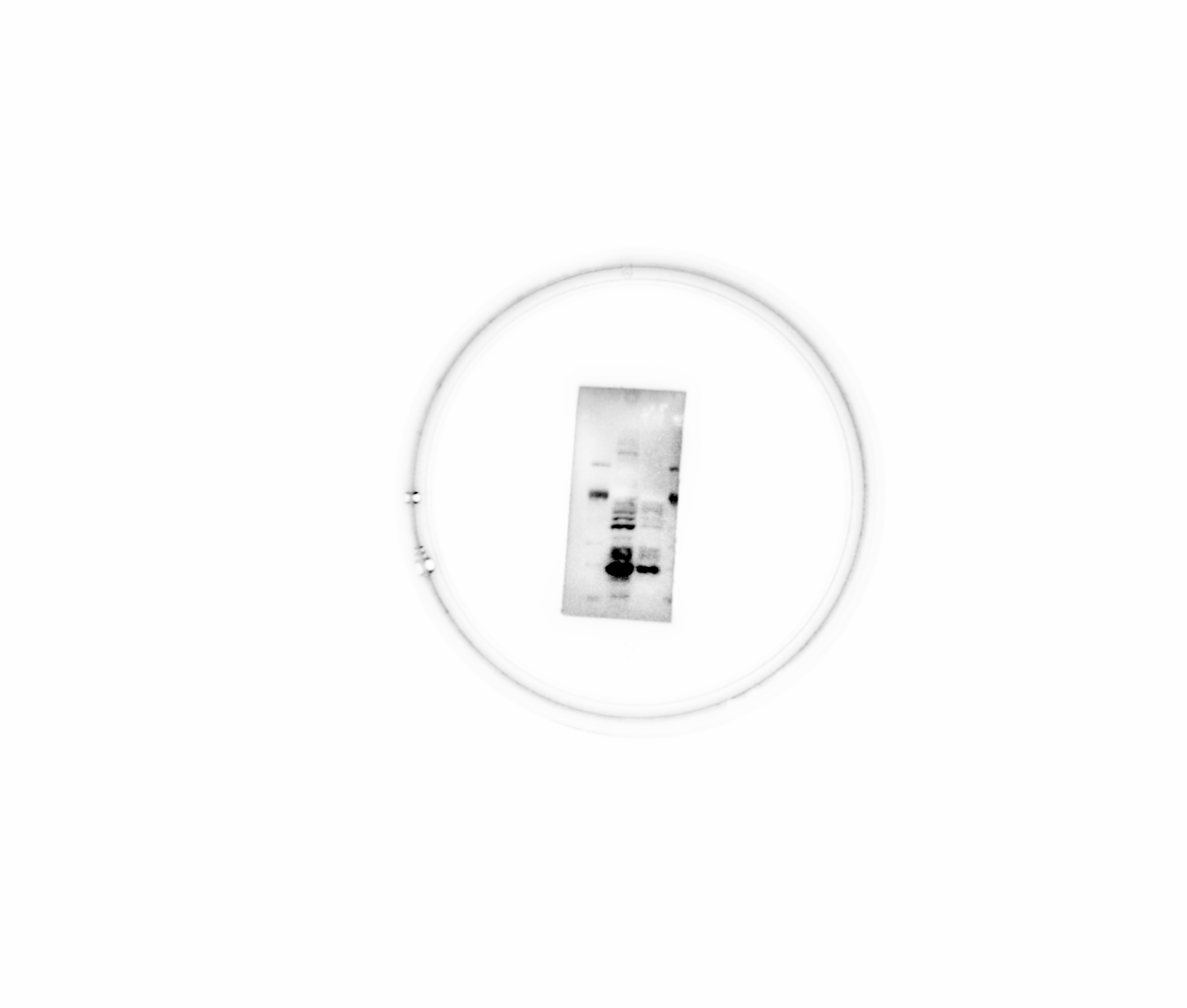

Supplement: Supplementary file 4 [file Data_Sheet_3.zip › Primary hippocampal neurons hnrnp A1/original data/2022-10-20 7'4a.tif]

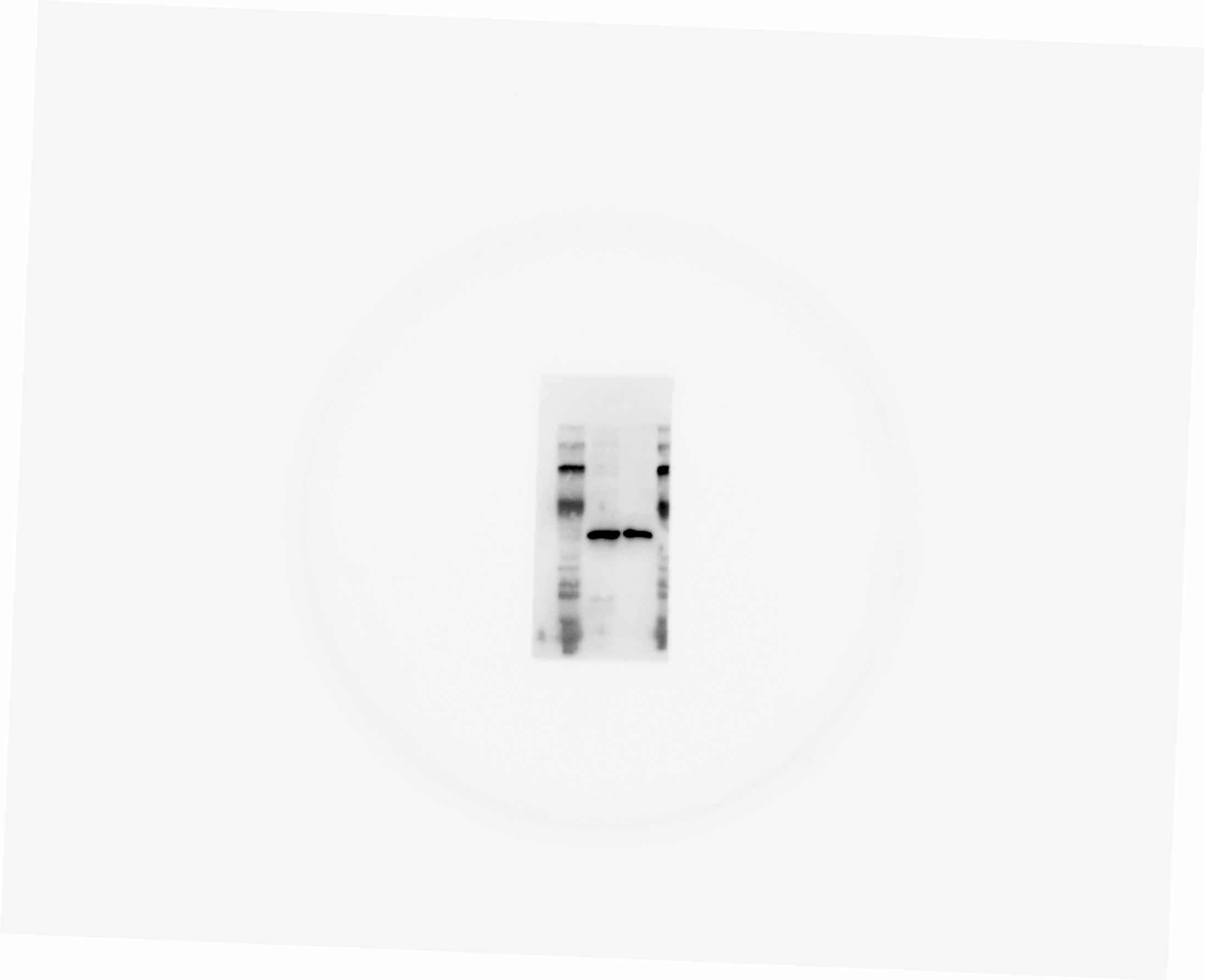

Supplement: Supplementary file 4 [file Data_Sheet_3.zip › Primary hippocampal neurons hnrnp A1/original data/wb 2022-10-20 7.4tub.tif]

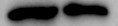

Supplement: Supplementary file 4 [file Data_Sheet_3.zip › Primary hippocampal neurons hnrnp A1/wb 2022-10-20 jxh 7.4tub.png]
