# Supplementary figures and images for: Suppression of hnRNP A1 binding to HK1 RNA leads to glycolytic dysfunction in Alzheimer’s disease models (part 2 of 4)
Source: Front Aging Neurosci. 2023 Aug 31;15:1218267. doi: 10.3389/fnagi.2023.1218267 (PMC10516183; doi:10.3389/fnagi.2023.1218267)

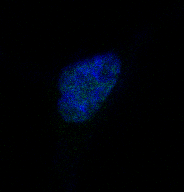

Supplement: Supplementary file 4 [file Data_Sheet_3.zip › hnRNP A1 immunofluorescence/a1.1/jxh2023.01.4_A1.1 A1_Crop001.tif]

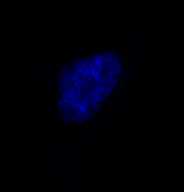

Supplement: Supplementary file 4 [file Data_Sheet_3.zip › hnRNP A1 immunofluorescence/a1.1/jxh2023.01.4_A1.1 A1_Crop001_ch00.tif]

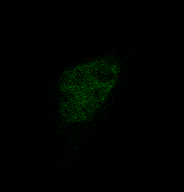

Supplement: Supplementary file 4 [file Data_Sheet_3.zip › hnRNP A1 immunofluorescence/a1.1/jxh2023.01.4_A1.1 A1_Crop001_ch01.tif]

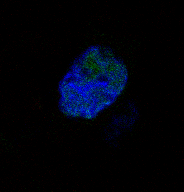

Supplement: Supplementary file 4 [file Data_Sheet_3.zip › hnRNP A1 immunofluorescence/a1.2/jxh2023.01.4_A1.2 A1_Crop001.tif]

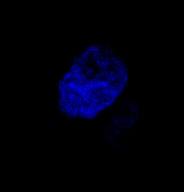

Supplement: Supplementary file 4 [file Data_Sheet_3.zip › hnRNP A1 immunofluorescence/a1.2/jxh2023.01.4_A1.2 A1_Crop001_ch00.tif]

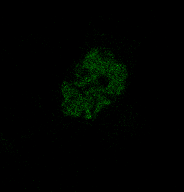

Supplement: Supplementary file 4 [file Data_Sheet_3.zip › hnRNP A1 immunofluorescence/a1.2/jxh2023.01.4_A1.2 A1_Crop001_ch01.tif]

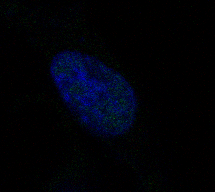

Supplement: Supplementary file 4 [file Data_Sheet_3.zip › hnRNP A1 immunofluorescence/a1.3/jxh2023.01.4_A1.3 A1_Crop001.tif]

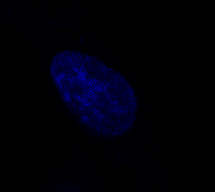

Supplement: Supplementary file 4 [file Data_Sheet_3.zip › hnRNP A1 immunofluorescence/a1.3/jxh2023.01.4_A1.3 A1_Crop001_ch00.tif]

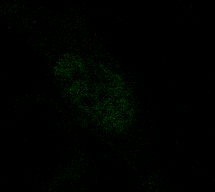

Supplement: Supplementary file 4 [file Data_Sheet_3.zip › hnRNP A1 immunofluorescence/a1.3/jxh2023.01.4_A1.3 A1_Crop001_ch01.tif]

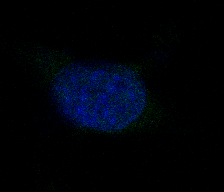

Supplement: Supplementary file 4 [file Data_Sheet_3.zip › hnRNP A1 immunofluorescence/a2.1/jxh2023.01.4_A2.1 A1_Crop001.tif]

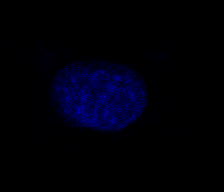

Supplement: Supplementary file 4 [file Data_Sheet_3.zip › hnRNP A1 immunofluorescence/a2.1/jxh2023.01.4_A2.1 A1_Crop001_ch00.tif]

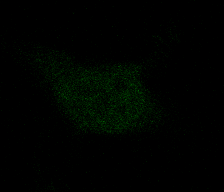

Supplement: Supplementary file 4 [file Data_Sheet_3.zip › hnRNP A1 immunofluorescence/a2.1/jxh2023.01.4_A2.1 A1_Crop001_ch01.tif]

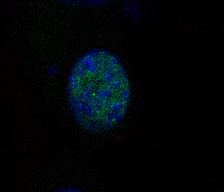

Supplement: Supplementary file 4 [file Data_Sheet_3.zip › hnRNP A1 immunofluorescence/a2.2/jxh2023.01.4_A2.2 A1_Crop001.tif]

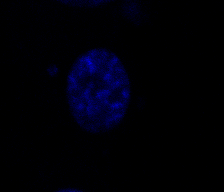

Supplement: Supplementary file 4 [file Data_Sheet_3.zip › hnRNP A1 immunofluorescence/a2.2/jxh2023.01.4_A2.2 A1_Crop001_ch00.tif]

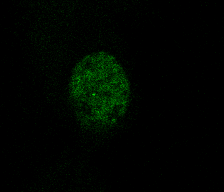

Supplement: Supplementary file 4 [file Data_Sheet_3.zip › hnRNP A1 immunofluorescence/a2.2/jxh2023.01.4_A2.2 A1_Crop001_ch01.tif]

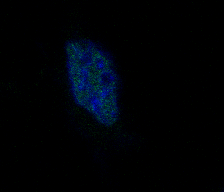

Supplement: Supplementary file 4 [file Data_Sheet_3.zip › hnRNP A1 immunofluorescence/a2.3/jxh2023.01.4_A2.3 A1_Crop001.tif]

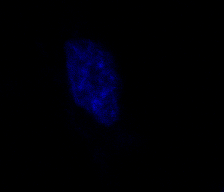

Supplement: Supplementary file 4 [file Data_Sheet_3.zip › hnRNP A1 immunofluorescence/a2.3/jxh2023.01.4_A2.3 A1_Crop001_ch00.tif]

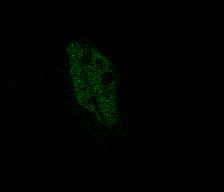

Supplement: Supplementary file 4 [file Data_Sheet_3.zip › hnRNP A1 immunofluorescence/a2.3/jxh2023.01.4_A2.3 A1_Crop001_ch01.tif]

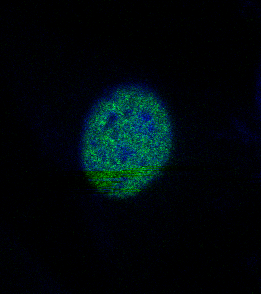

Supplement: Supplementary file 4 [file Data_Sheet_3.zip › hnRNP A1 immunofluorescence/o1.1/jxh2023.01.4_O1.1 A1_Crop001.tif]

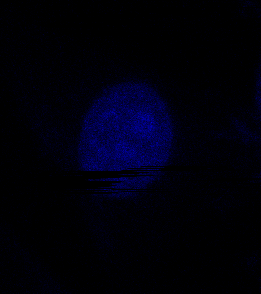

Supplement: Supplementary file 4 [file Data_Sheet_3.zip › hnRNP A1 immunofluorescence/o1.1/jxh2023.01.4_O1.1 A1_Crop001_ch00.tif]

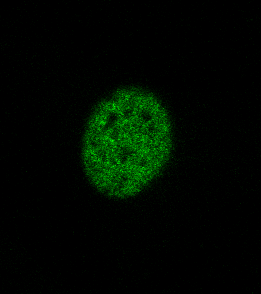

Supplement: Supplementary file 4 [file Data_Sheet_3.zip › hnRNP A1 immunofluorescence/o1.1/jxh2023.01.4_O1.1 A1_Crop001_ch01.tif]

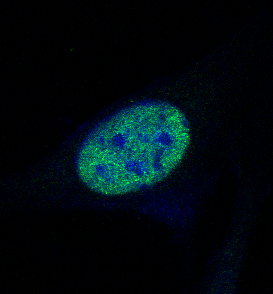

Supplement: Supplementary file 4 [file Data_Sheet_3.zip › hnRNP A1 immunofluorescence/o1.2/jxh2023.01.4_O1.2 A1_Crop001.tif]

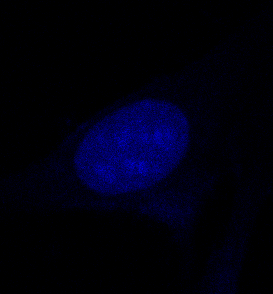

Supplement: Supplementary file 4 [file Data_Sheet_3.zip › hnRNP A1 immunofluorescence/o1.2/jxh2023.01.4_O1.2 A1_Crop001_ch00.tif]

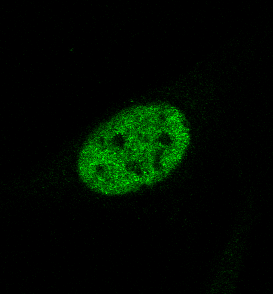

Supplement: Supplementary file 4 [file Data_Sheet_3.zip › hnRNP A1 immunofluorescence/o1.2/jxh2023.01.4_O1.2 A1_Crop001_ch01.tif]

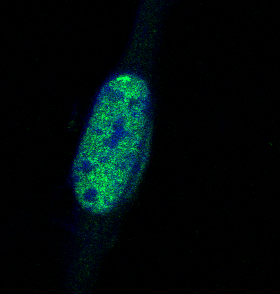

Supplement: Supplementary file 4 [file Data_Sheet_3.zip › hnRNP A1 immunofluorescence/o1.3/jxh2023.01.4_O1.3 A1_Crop001.tif]

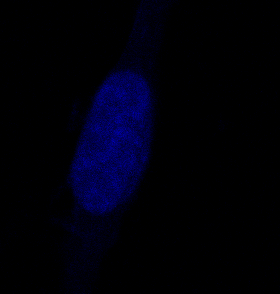

Supplement: Supplementary file 4 [file Data_Sheet_3.zip › hnRNP A1 immunofluorescence/o1.3/jxh2023.01.4_O1.3 A1_Crop001_ch00.tif]

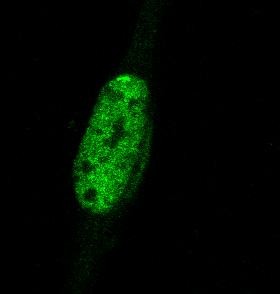

Supplement: Supplementary file 4 [file Data_Sheet_3.zip › hnRNP A1 immunofluorescence/o1.3/jxh2023.01.4_O1.3 A1_Crop001_ch01.tif]

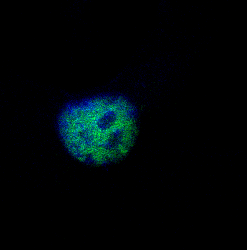

Supplement: Supplementary file 4 [file Data_Sheet_3.zip › hnRNP A1 immunofluorescence/o1.4/jxh2023.01.4_O1.4 A1_Crop001.tif]

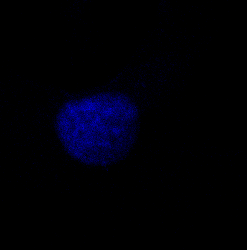

Supplement: Supplementary file 4 [file Data_Sheet_3.zip › hnRNP A1 immunofluorescence/o1.4/jxh2023.01.4_O1.4 A1_Crop001_ch00.tif]

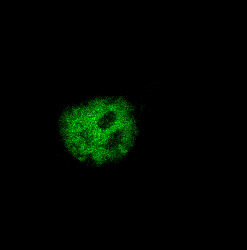

Supplement: Supplementary file 4 [file Data_Sheet_3.zip › hnRNP A1 immunofluorescence/o1.4/jxh2023.01.4_O1.4 A1_Crop001_ch01.tif]

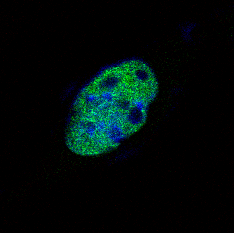

Supplement: Supplementary file 4 [file Data_Sheet_3.zip › hnRNP A1 immunofluorescence/o1.5/jxh2023.01.4_O1.5 A1_Crop001.tif]

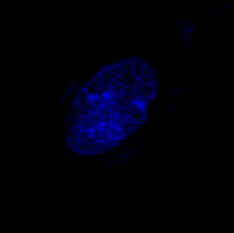

Supplement: Supplementary file 4 [file Data_Sheet_3.zip › hnRNP A1 immunofluorescence/o1.5/jxh2023.01.4_O1.5 A1_Crop001_ch00.tif]

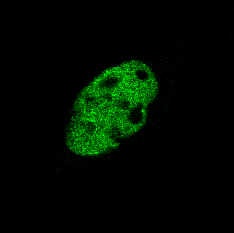

Supplement: Supplementary file 4 [file Data_Sheet_3.zip › hnRNP A1 immunofluorescence/o1.5/jxh2023.01.4_O1.5 A1_Crop001_ch01.tif]

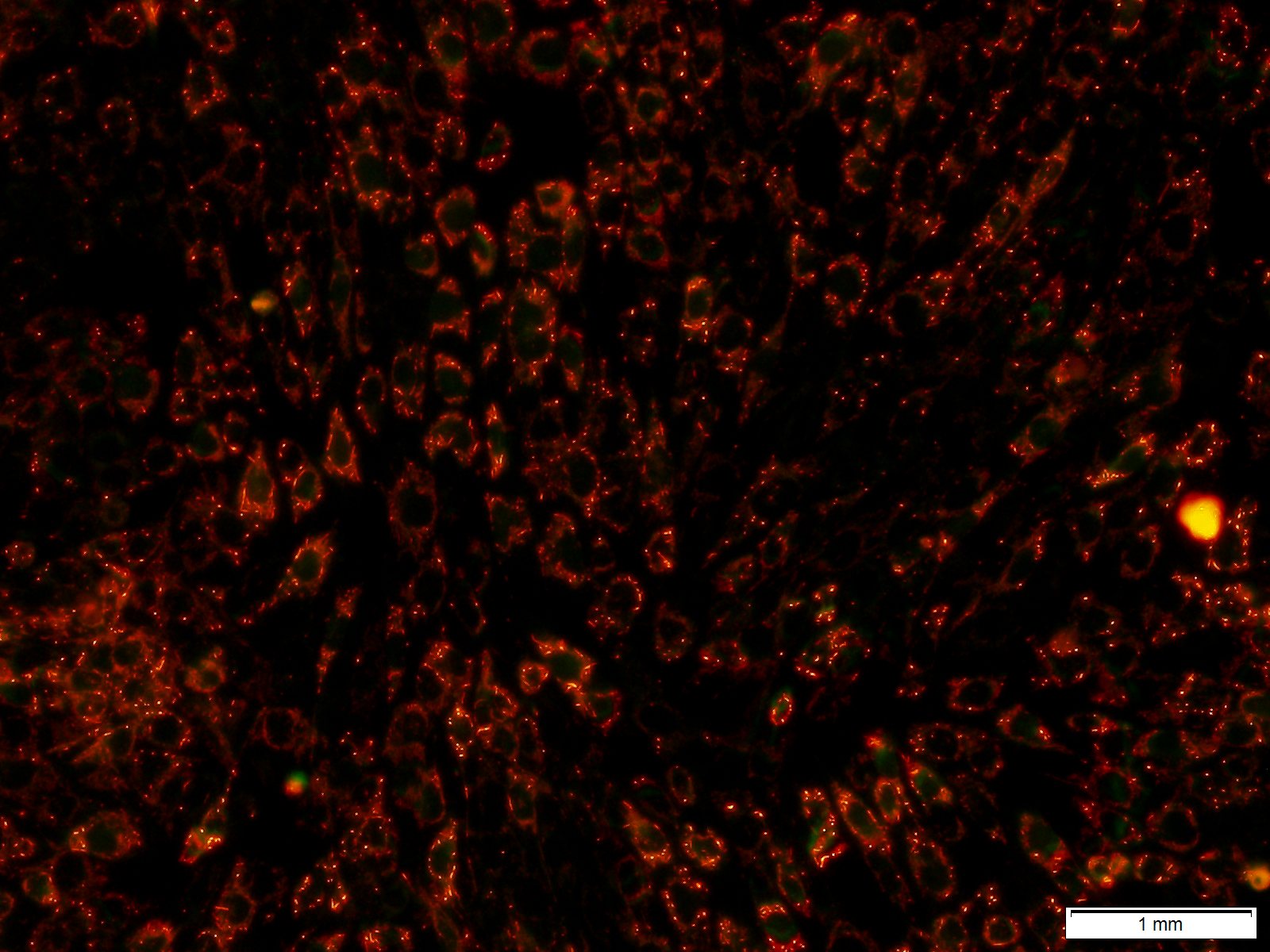

Supplement: Supplementary file 4 [file Data_Sheet_3.zip › mitochondrial membrane potential (VPC)/K1 mer.tif]

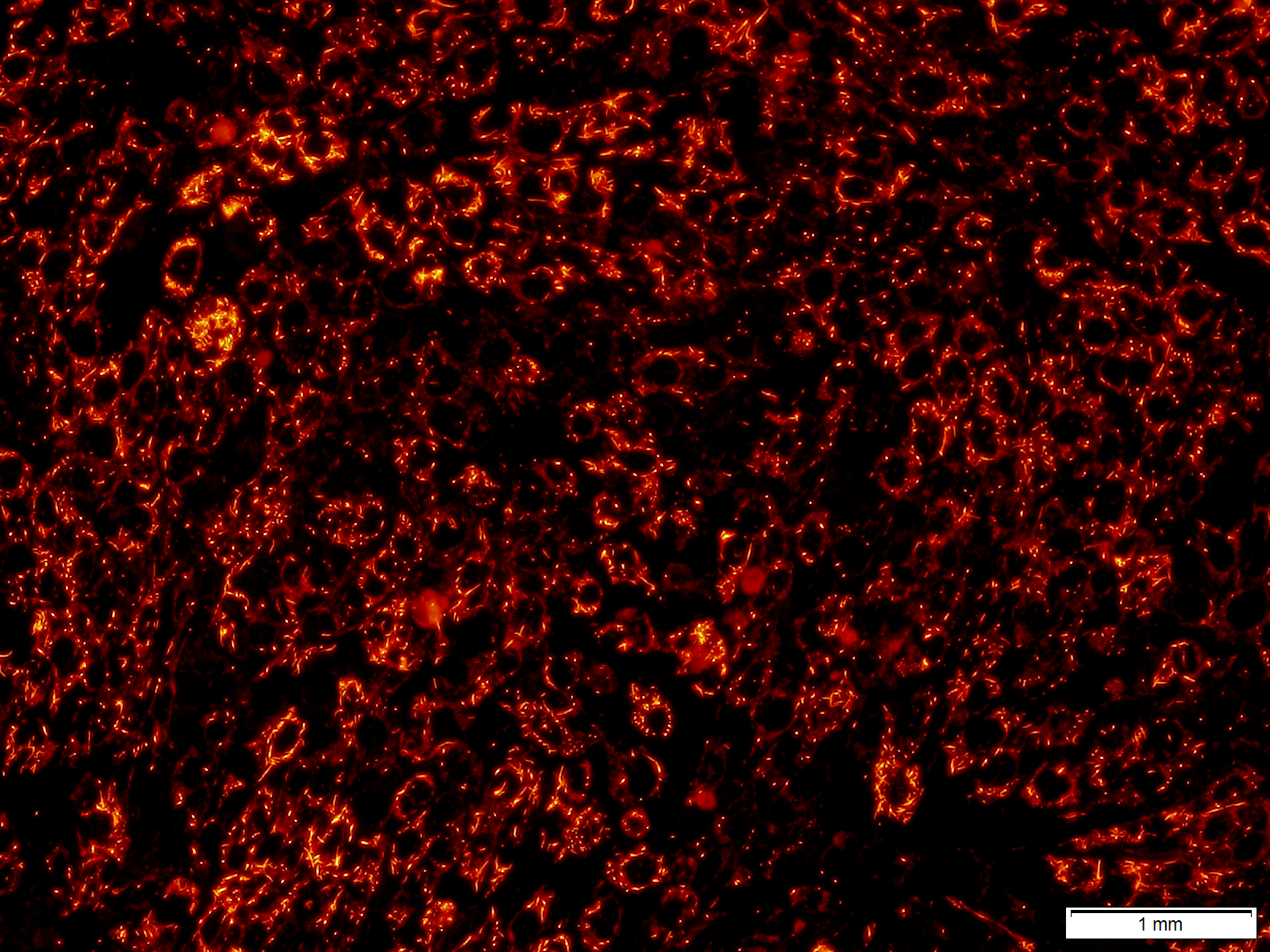

Supplement: Supplementary file 4 [file Data_Sheet_3.zip › mitochondrial membrane potential (VPC)/K2 红.tif]

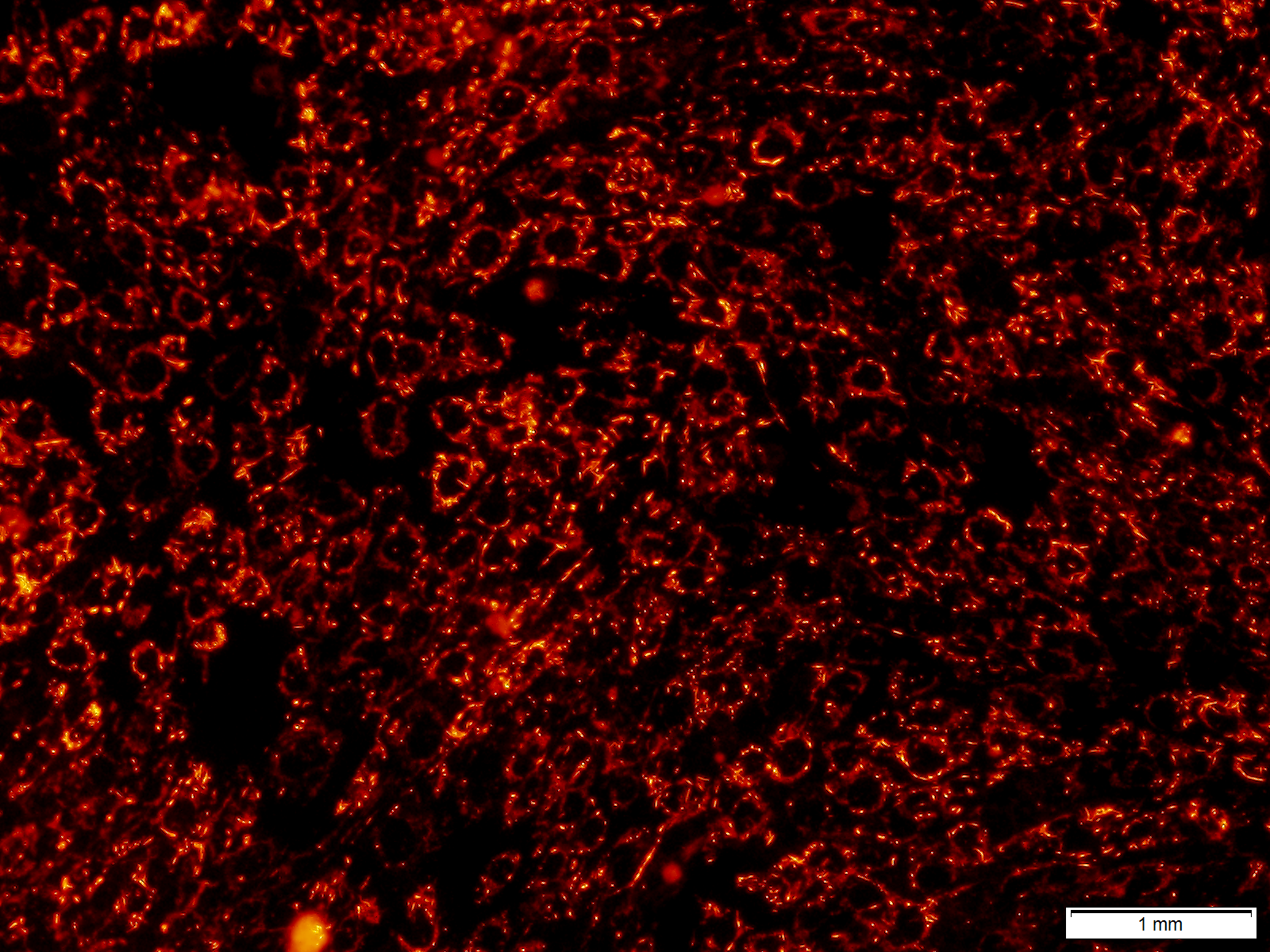

Supplement: Supplementary file 4 [file Data_Sheet_3.zip › mitochondrial membrane potential (VPC)/K3 红.tif]

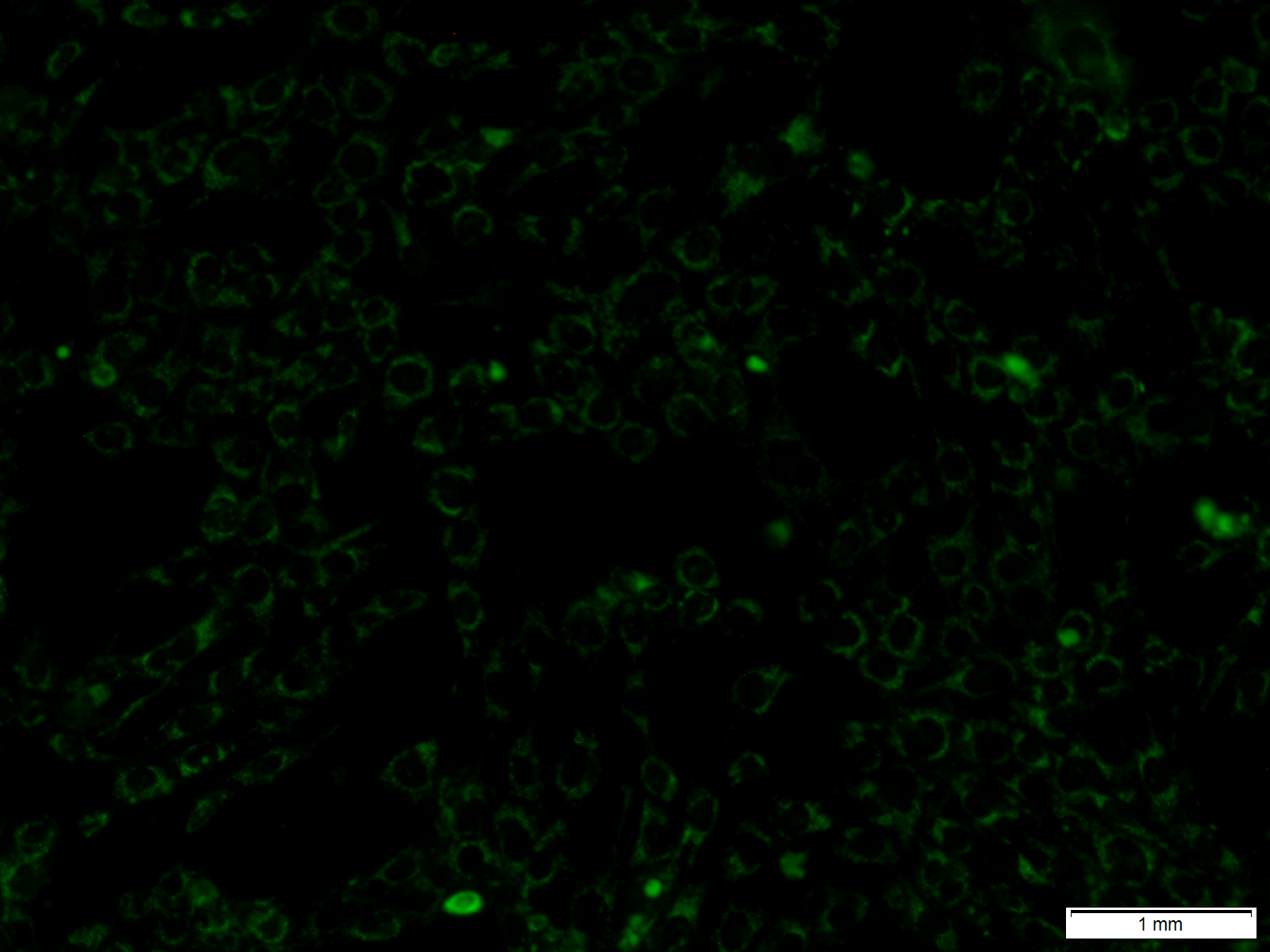

Supplement: Supplementary file 4 [file Data_Sheet_3.zip › mitochondrial membrane potential (VPC)/VPC2 mer.tif]

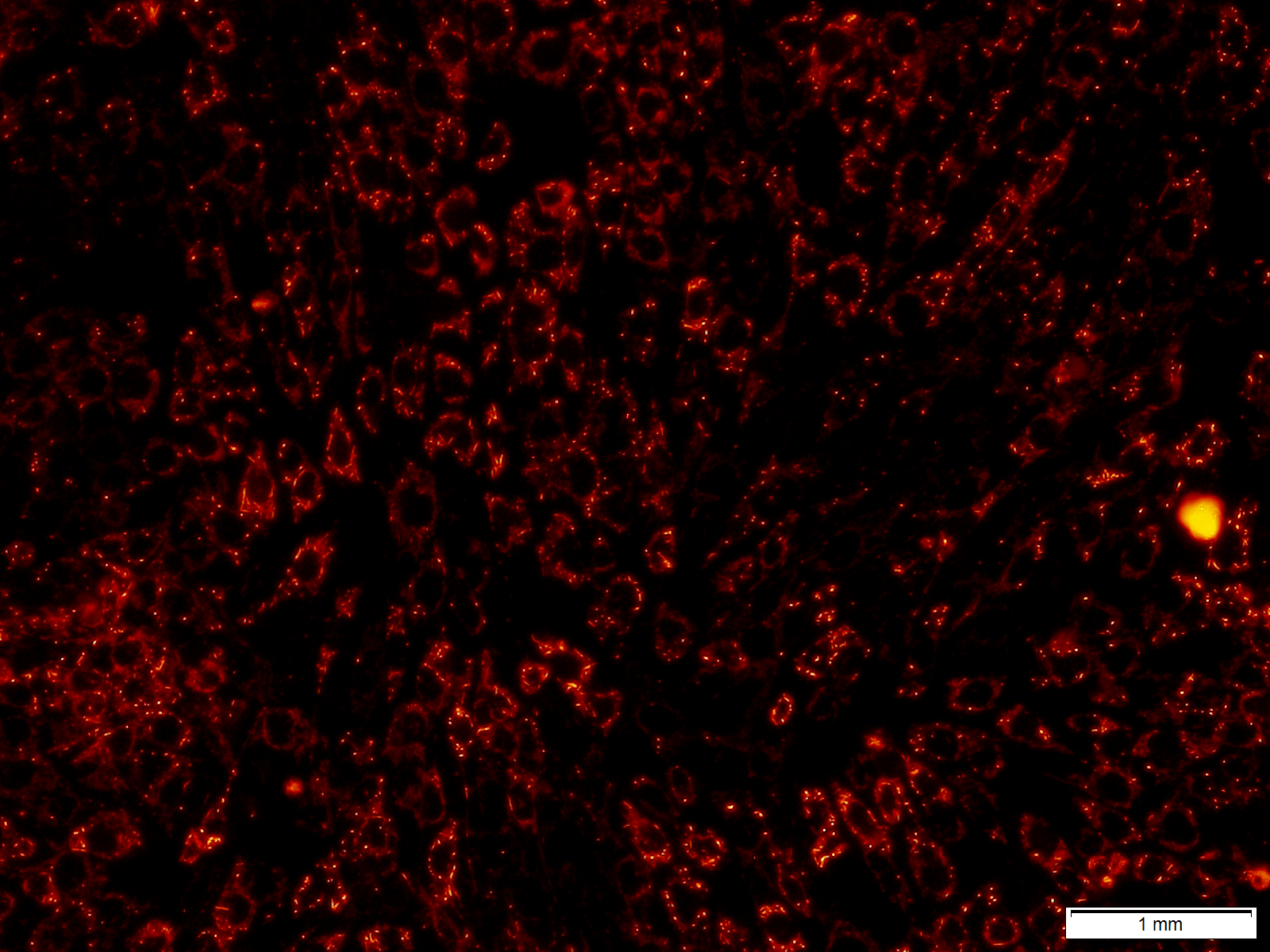

Supplement: Supplementary file 4 [file Data_Sheet_3.zip › mitochondrial membrane potential (VPC)/k1 红.tif]

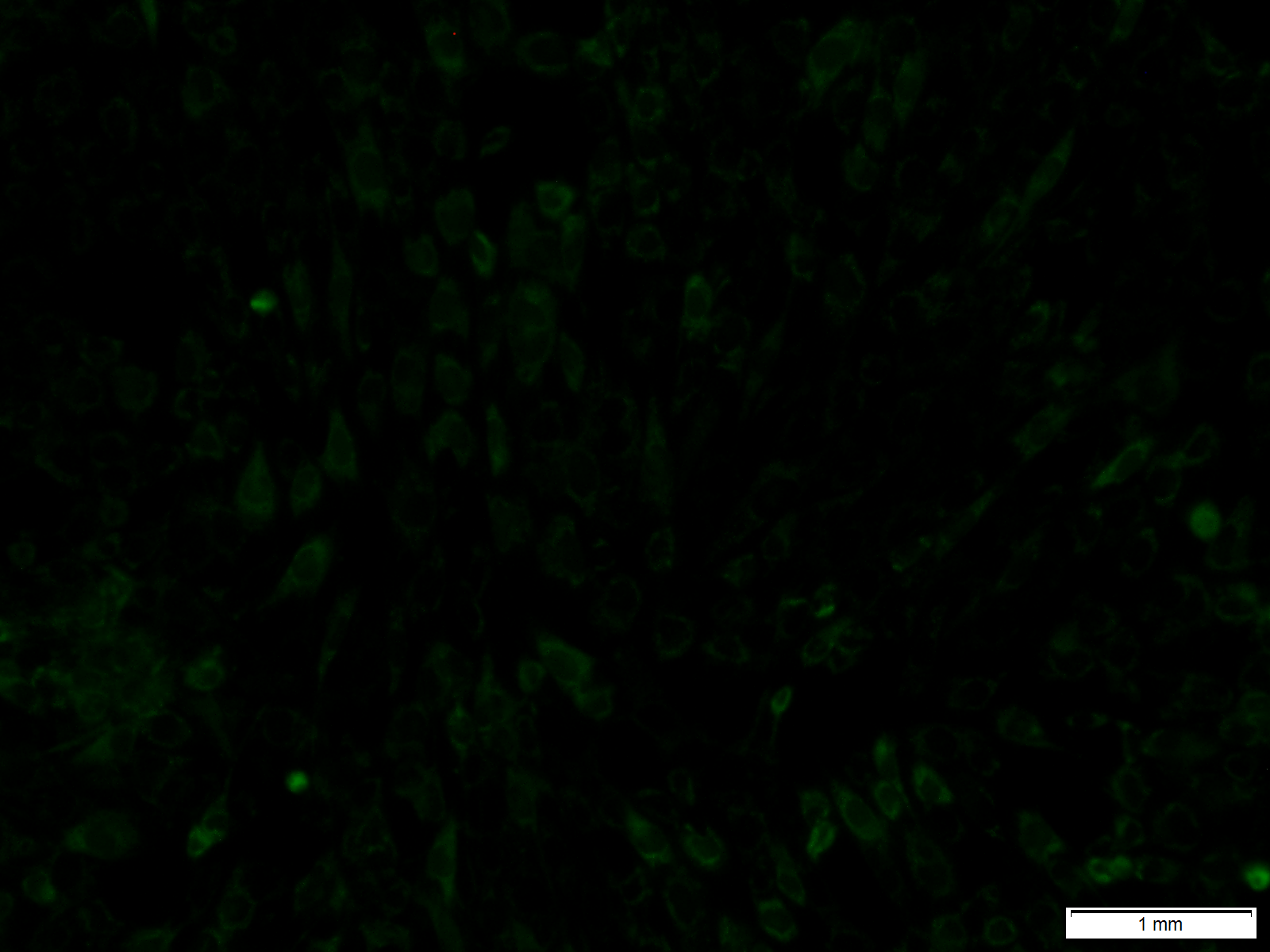

Supplement: Supplementary file 4 [file Data_Sheet_3.zip › mitochondrial membrane potential (VPC)/k1 绿.tif]

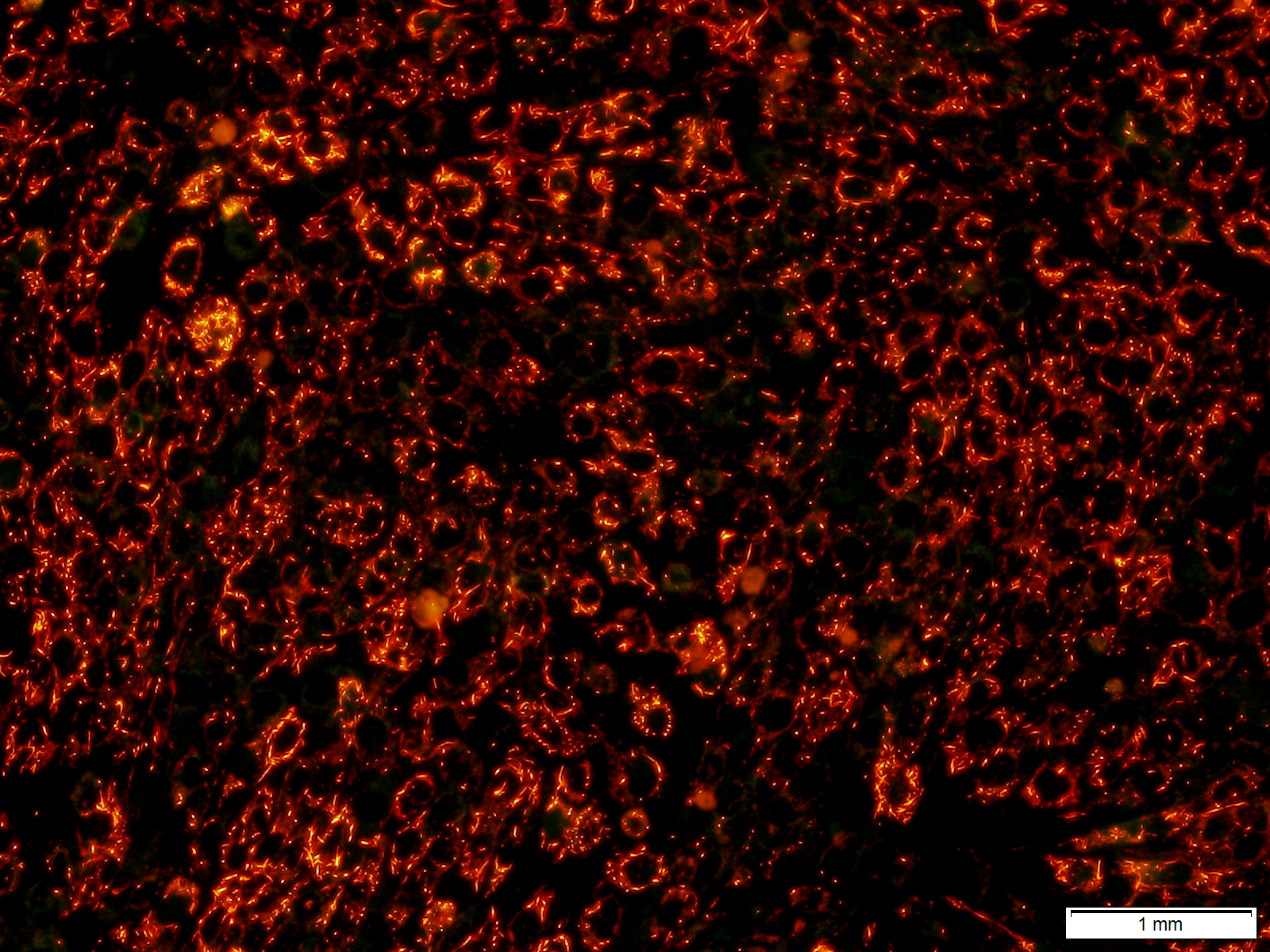

Supplement: Supplementary file 4 [file Data_Sheet_3.zip › mitochondrial membrane potential (VPC)/k2 mer.tif]

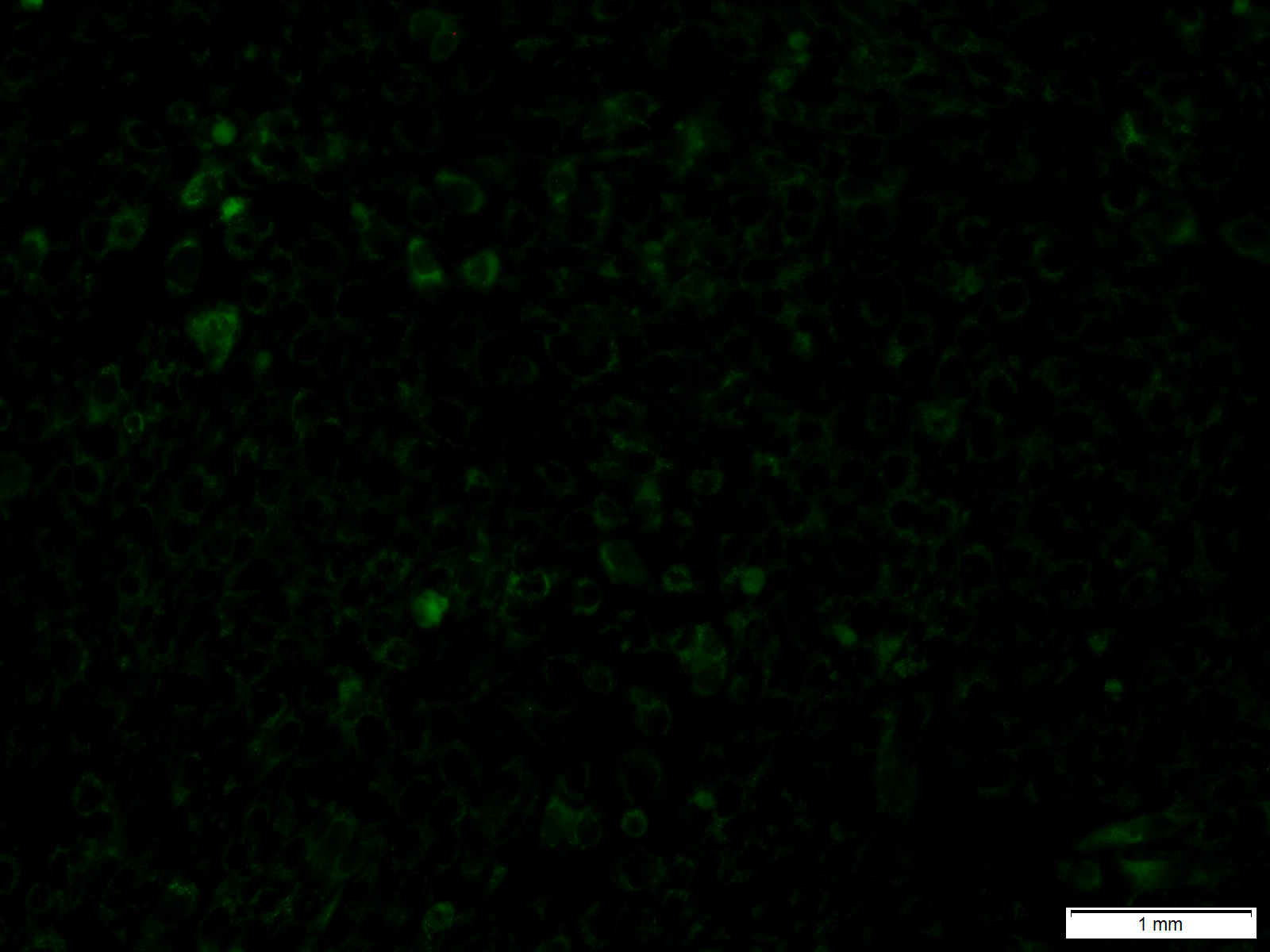

Supplement: Supplementary file 4 [file Data_Sheet_3.zip › mitochondrial membrane potential (VPC)/k2 绿.tif]

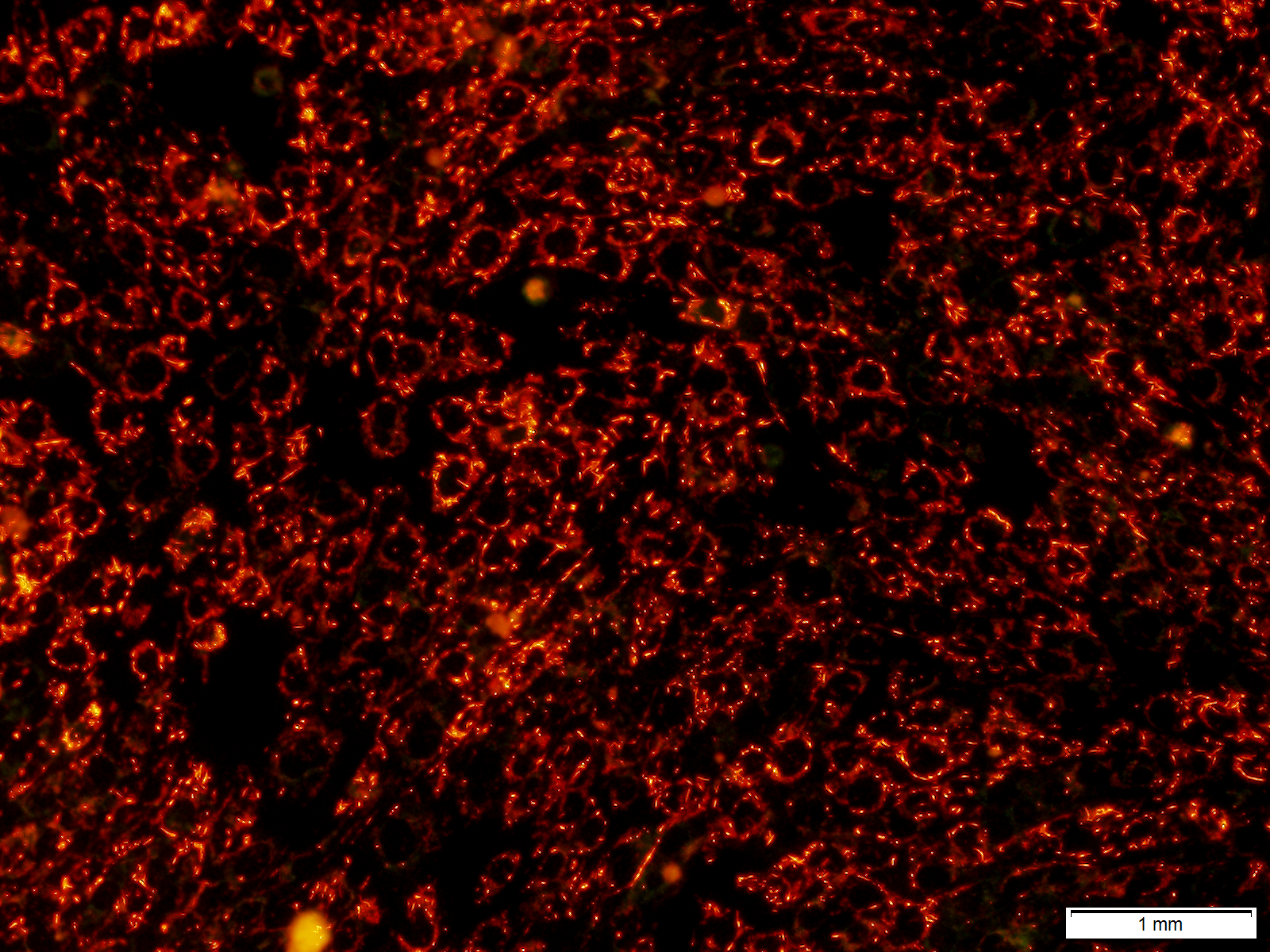

Supplement: Supplementary file 4 [file Data_Sheet_3.zip › mitochondrial membrane potential (VPC)/k3 mer.tif]

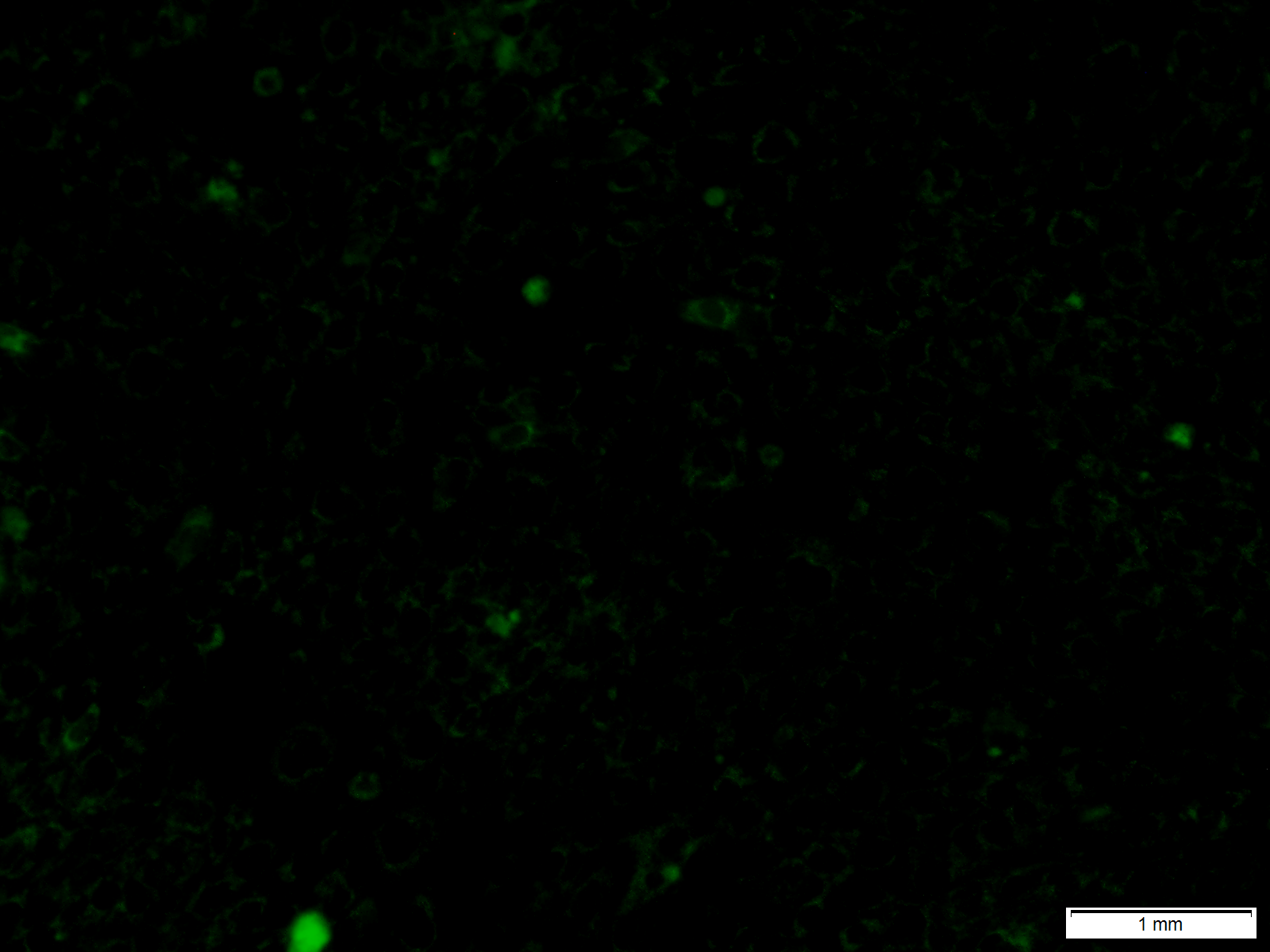

Supplement: Supplementary file 4 [file Data_Sheet_3.zip › mitochondrial membrane potential (VPC)/k3 绿.tif]

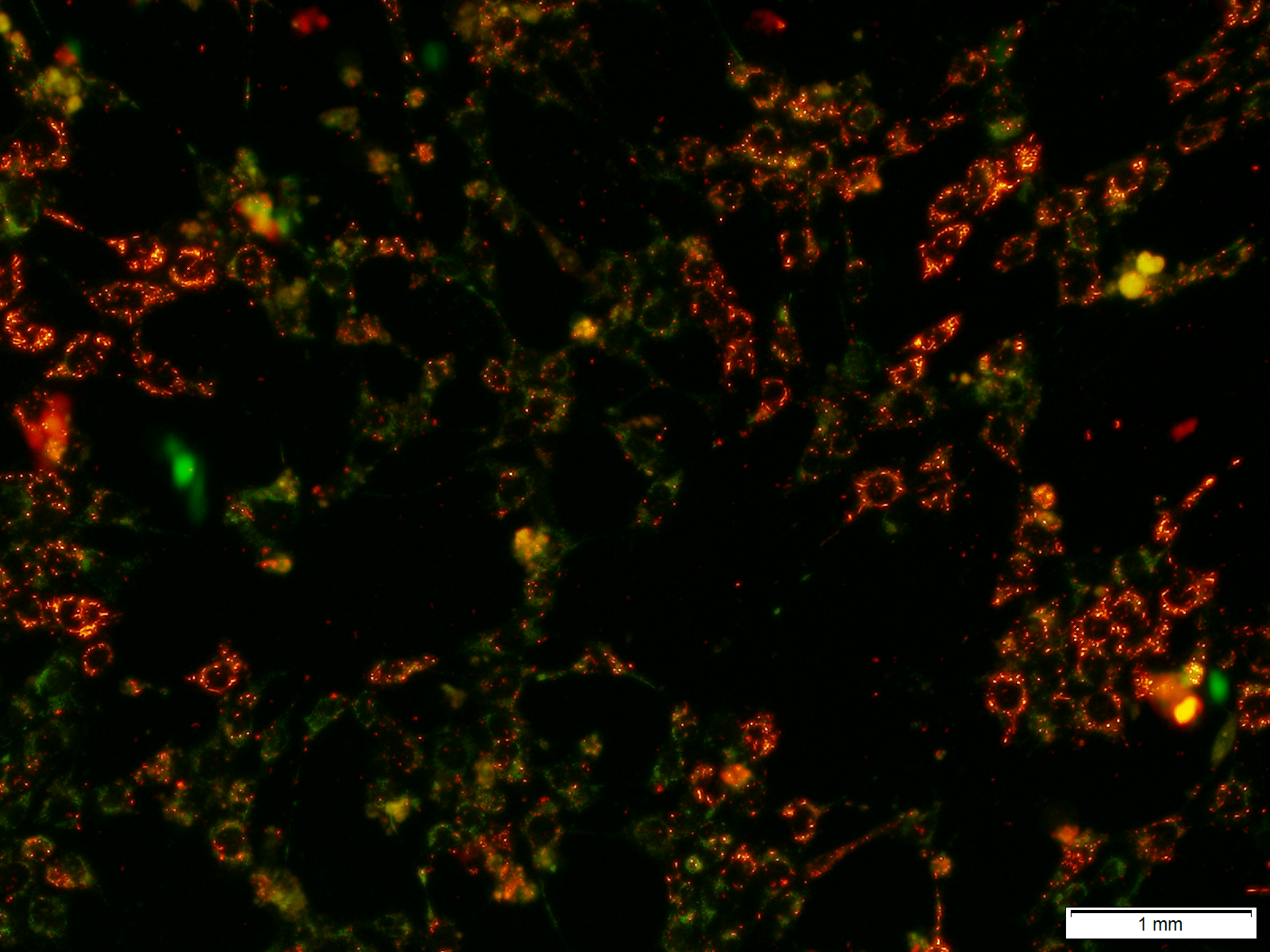

Supplement: Supplementary file 4 [file Data_Sheet_3.zip › mitochondrial membrane potential (VPC)/vpc1 MER.tif]

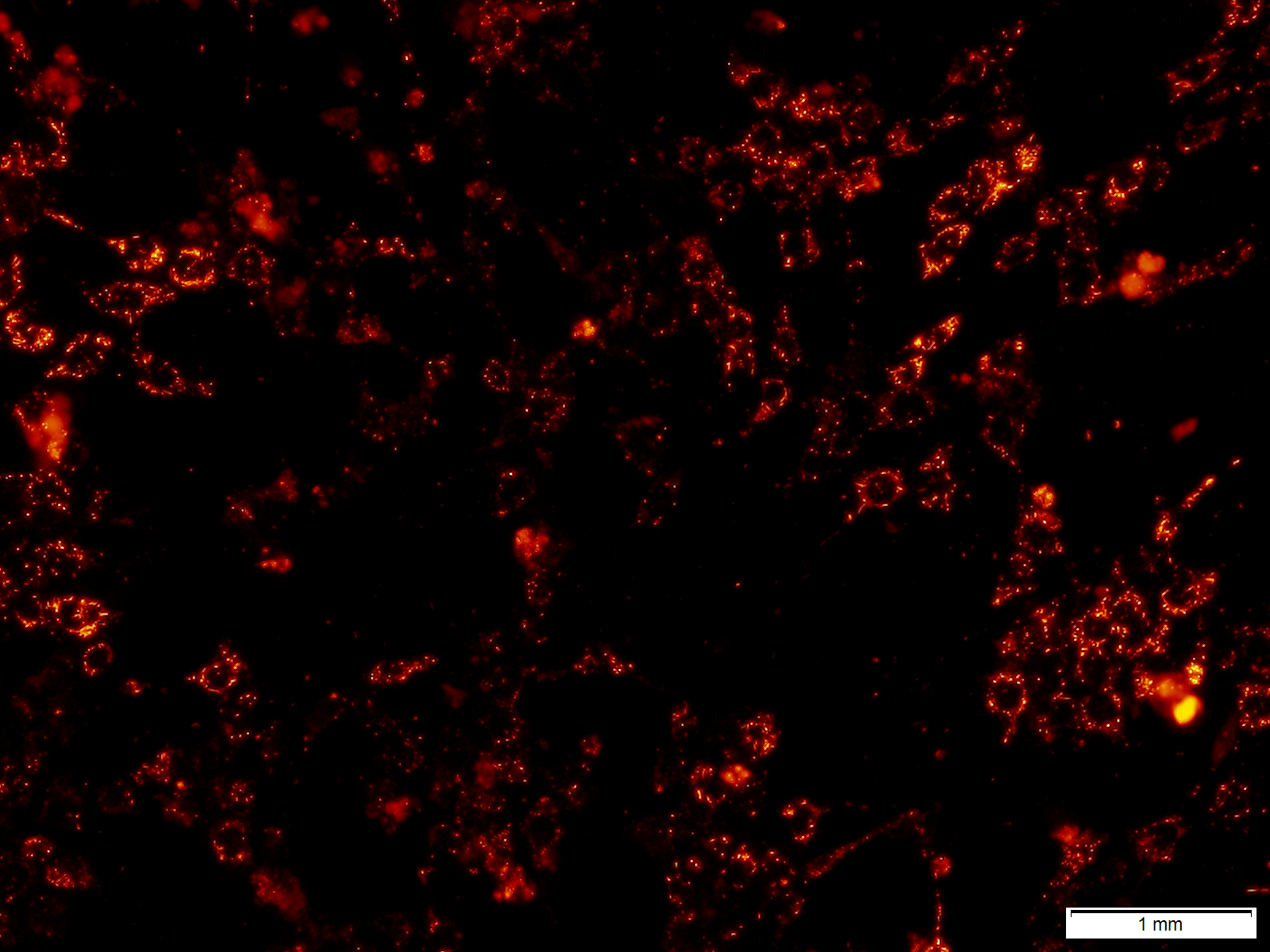

Supplement: Supplementary file 4 [file Data_Sheet_3.zip › mitochondrial membrane potential (VPC)/vpc1 红1.tif]

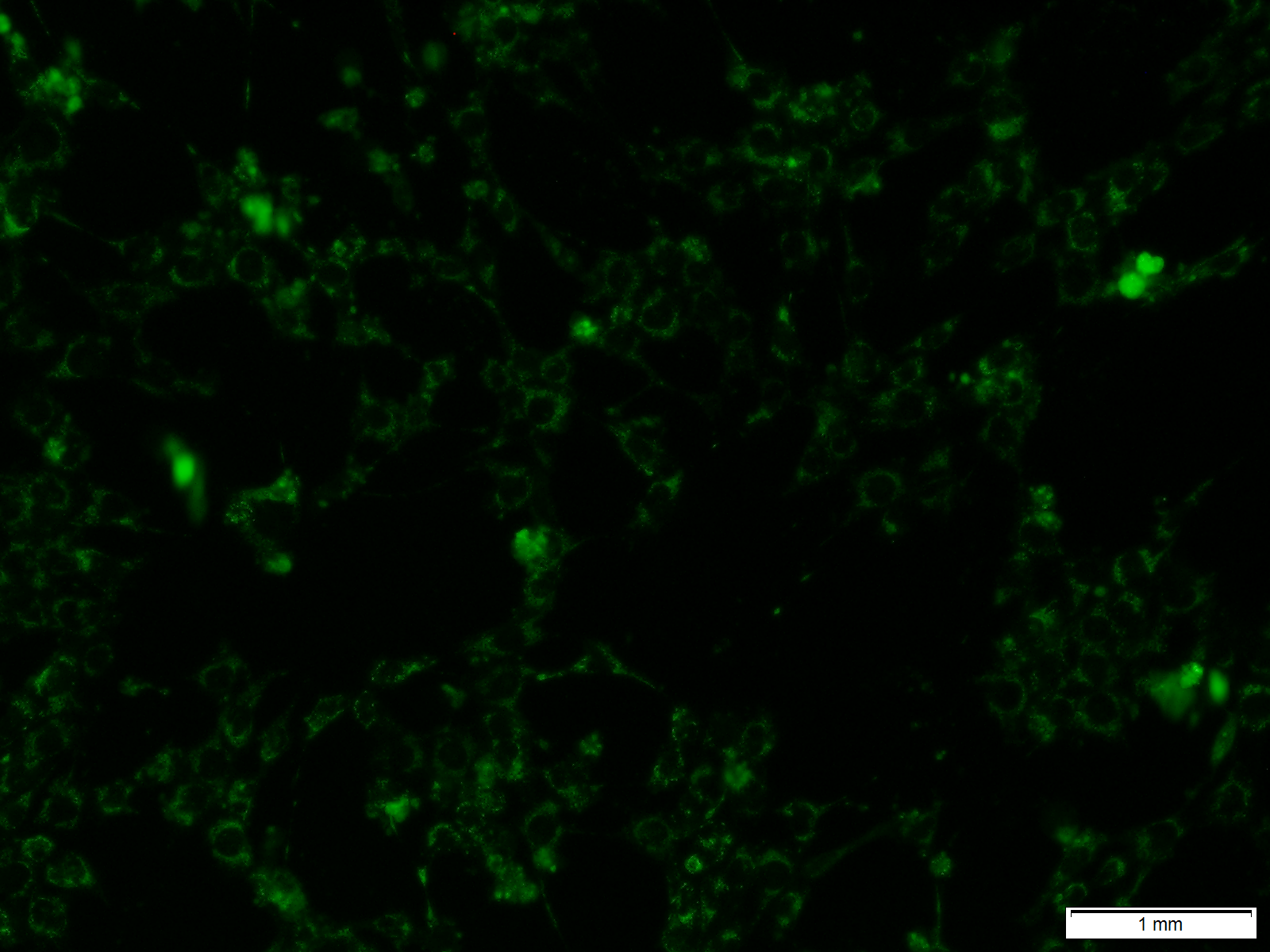

Supplement: Supplementary file 4 [file Data_Sheet_3.zip › mitochondrial membrane potential (VPC)/vpc1 绿1.tif]

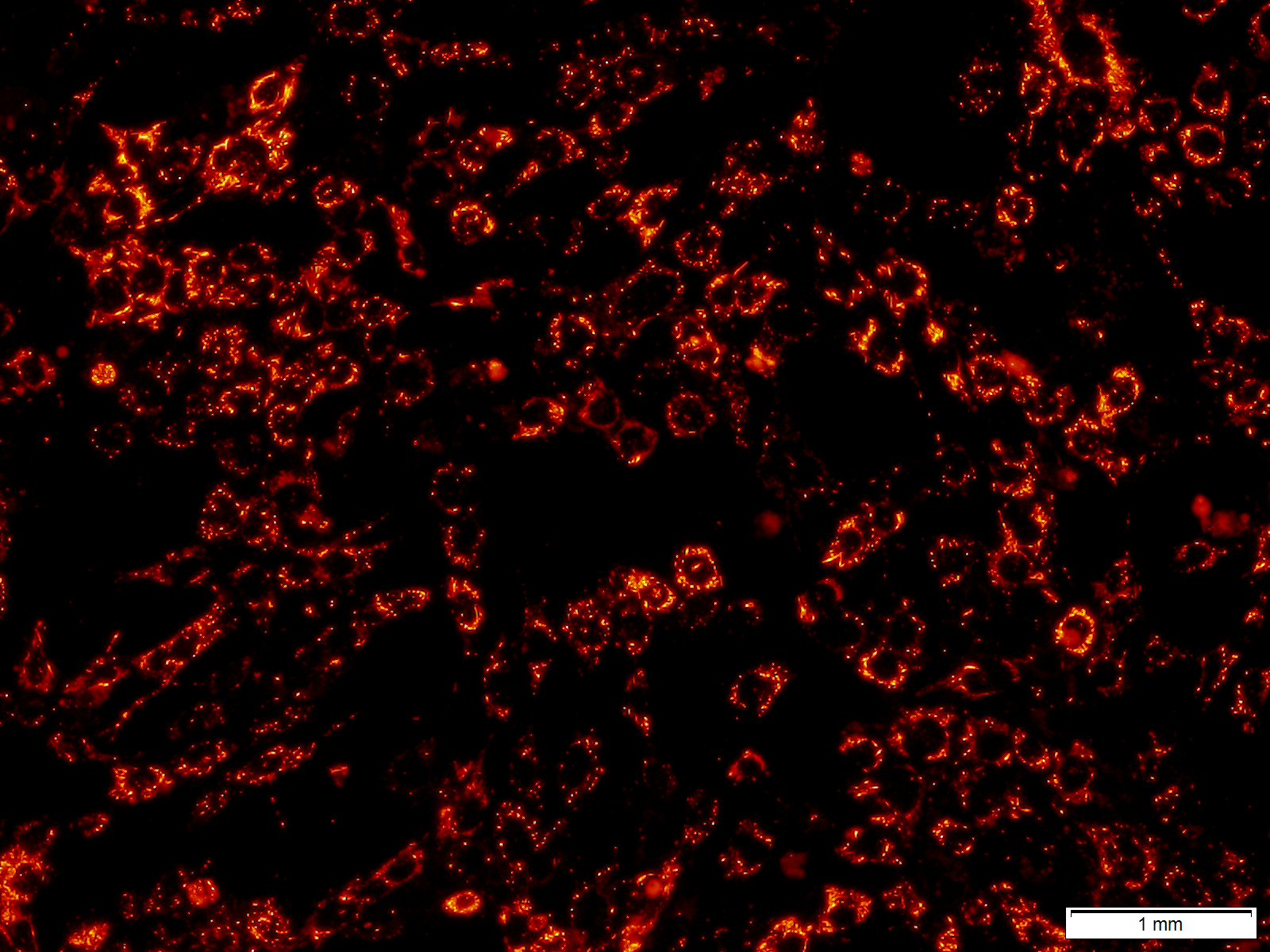

Supplement: Supplementary file 4 [file Data_Sheet_3.zip › mitochondrial membrane potential (VPC)/vpc2 红.tif]

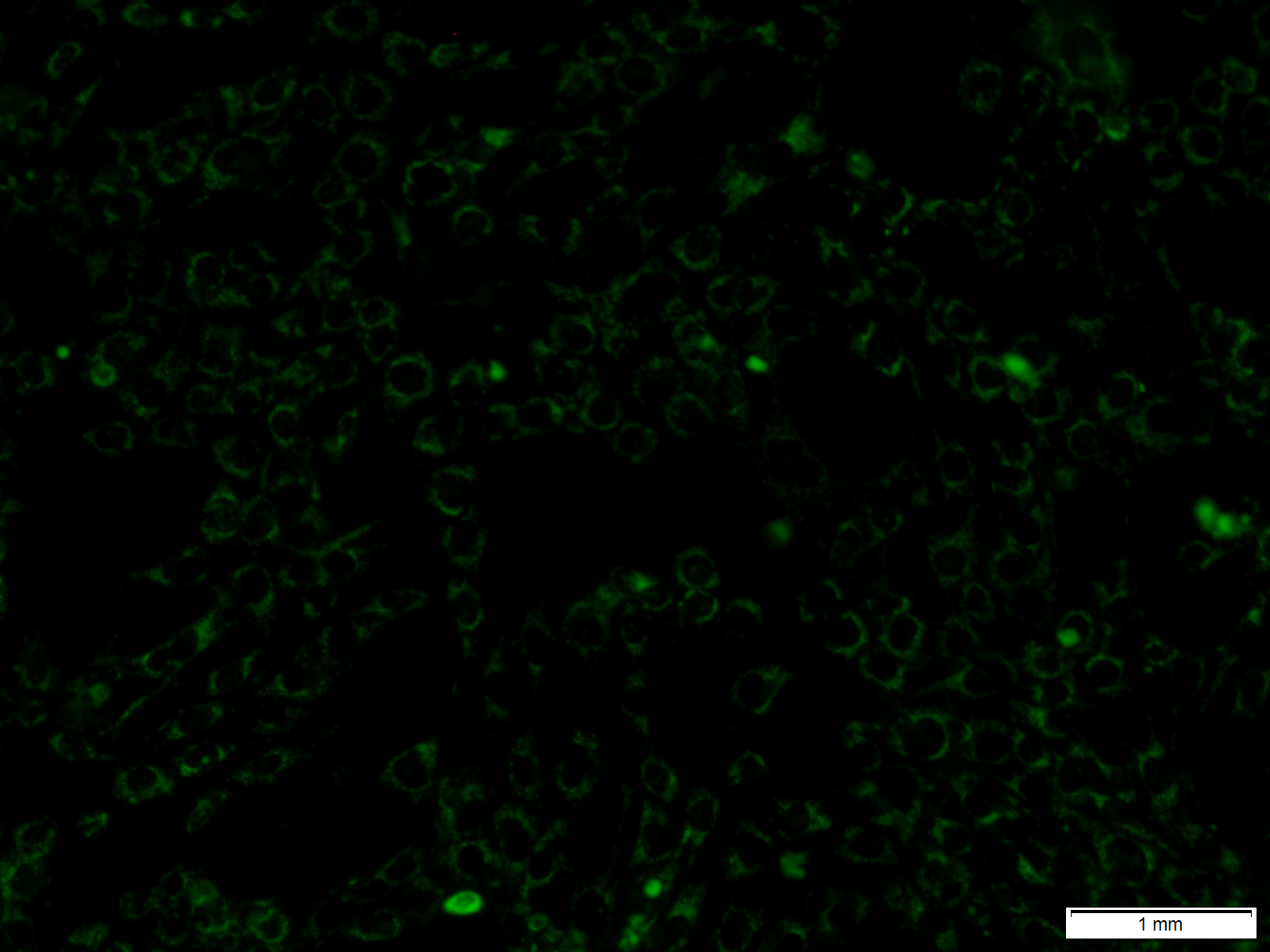

Supplement: Supplementary file 4 [file Data_Sheet_3.zip › mitochondrial membrane potential (VPC)/vpc2 绿.tif]

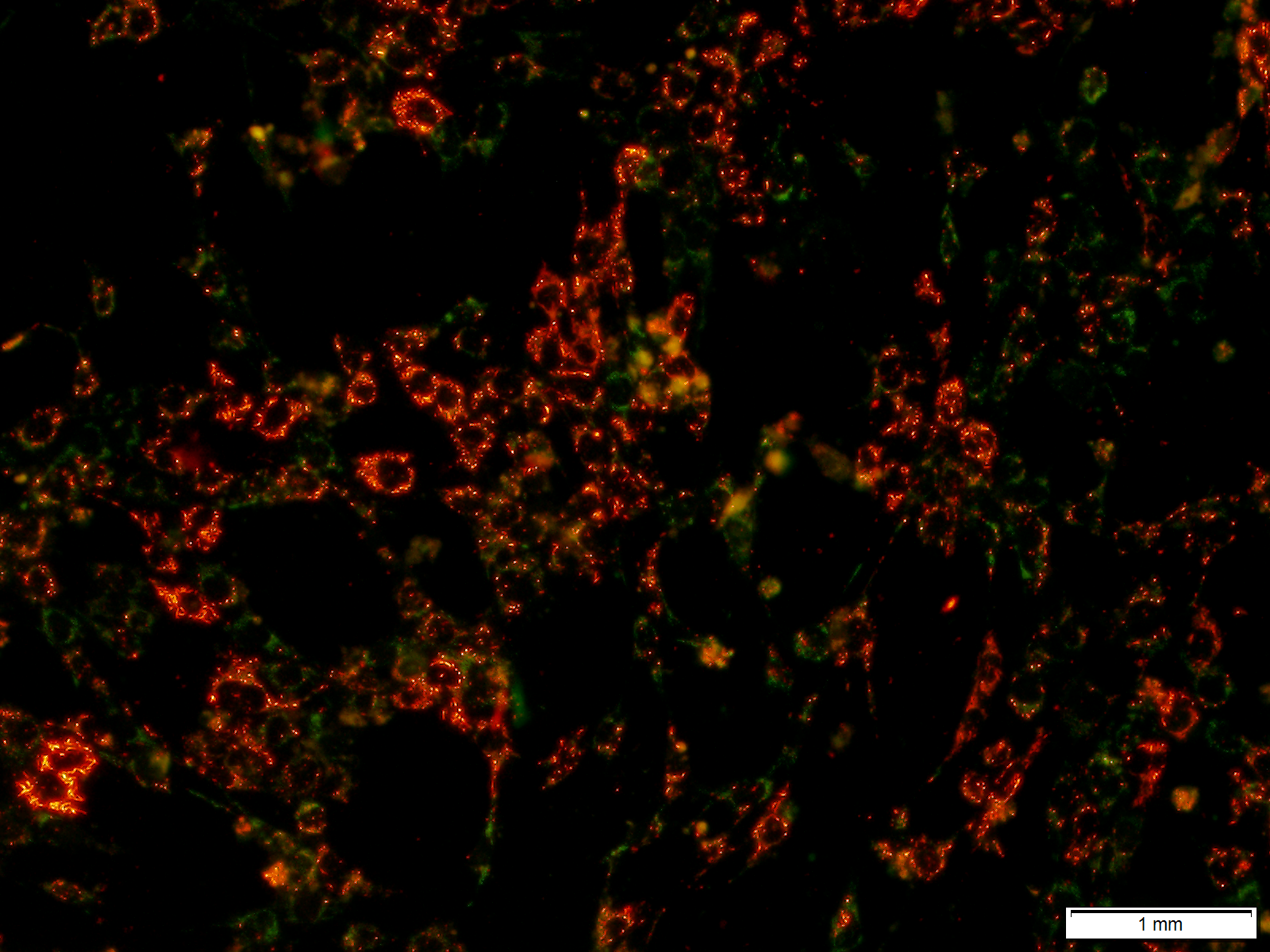

Supplement: Supplementary file 4 [file Data_Sheet_3.zip › mitochondrial membrane potential (VPC)/vpc3 mer.tif]

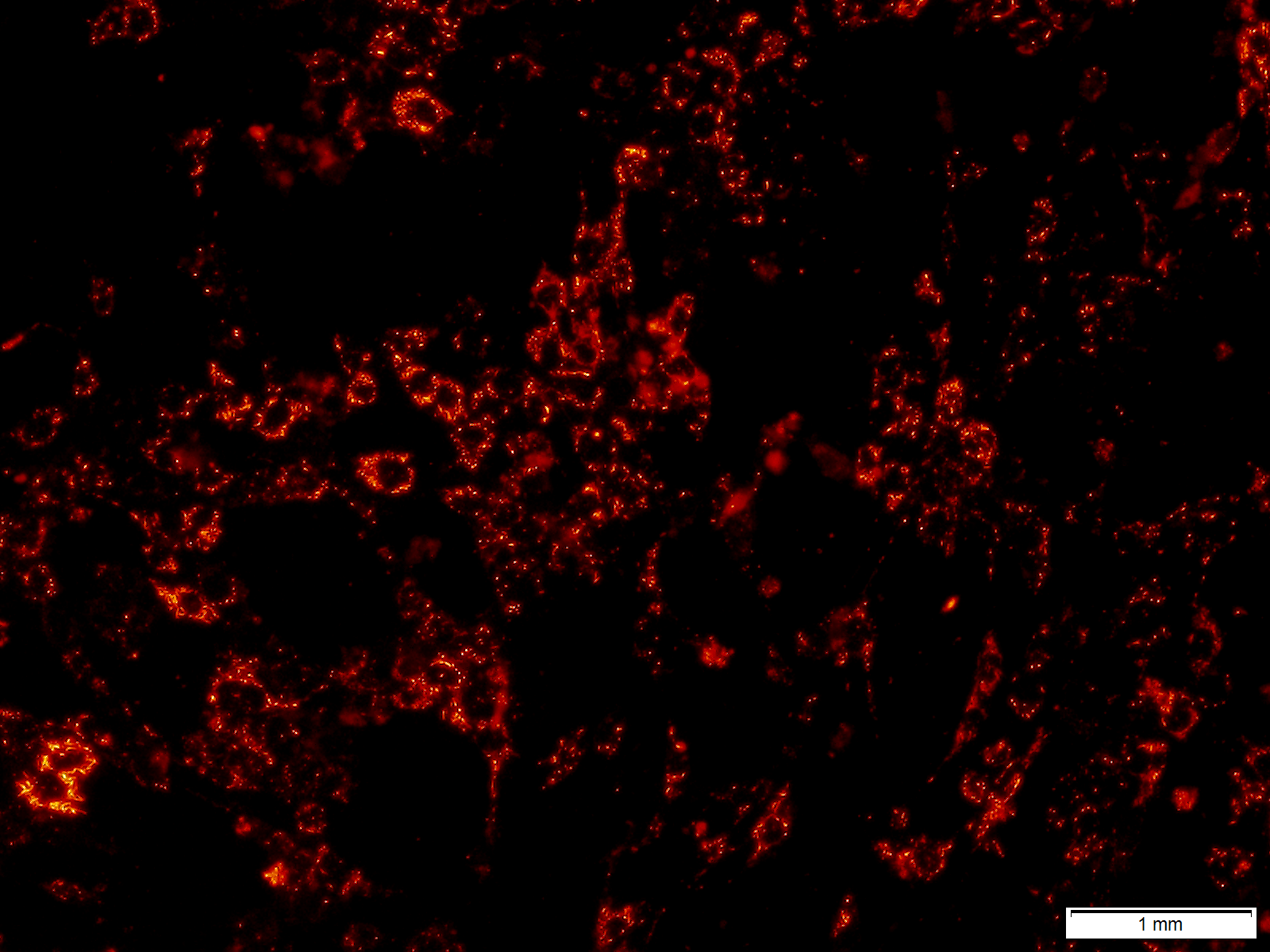

Supplement: Supplementary file 4 [file Data_Sheet_3.zip › mitochondrial membrane potential (VPC)/vpc3 红.tif]

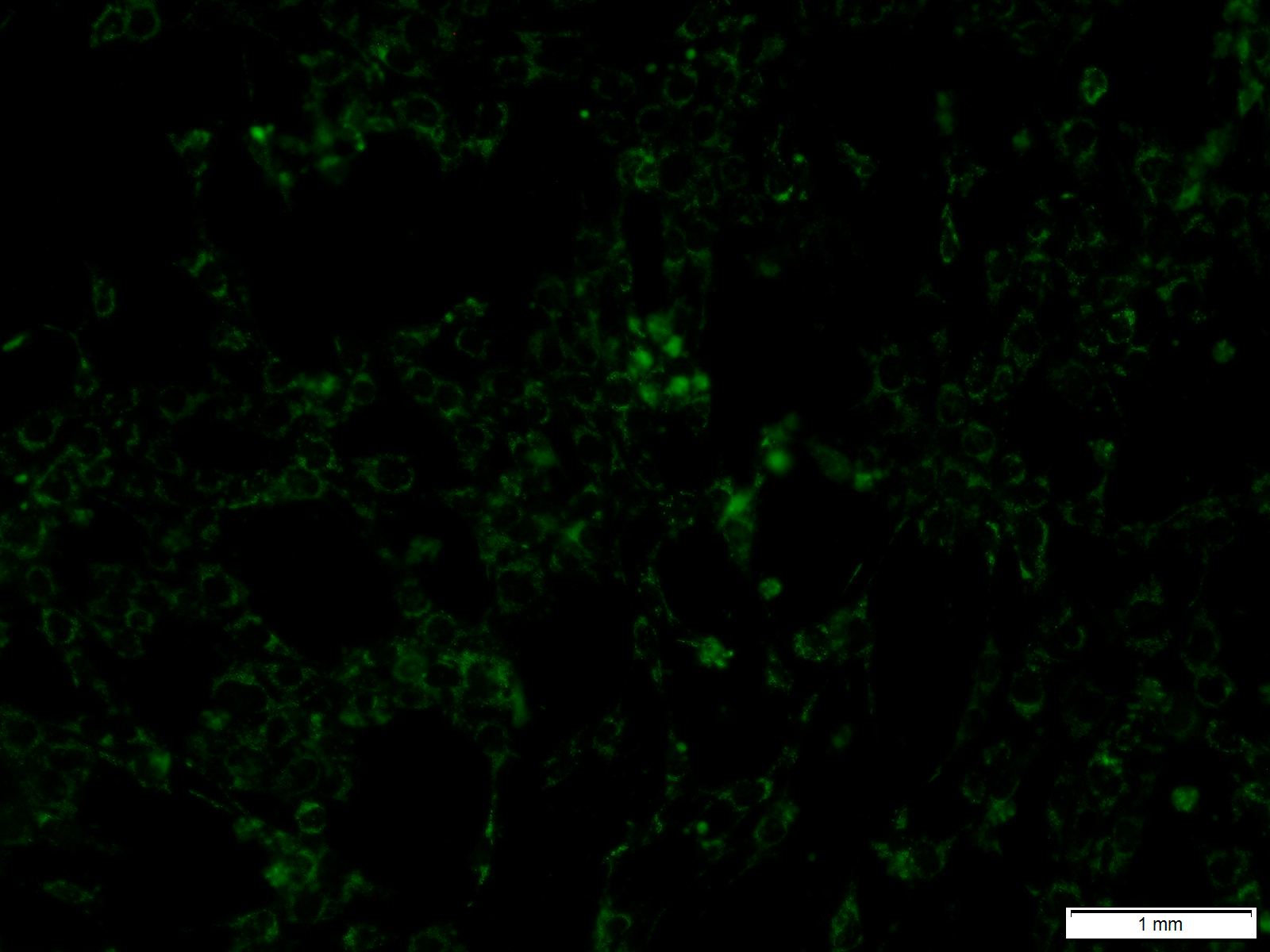

Supplement: Supplementary file 4 [file Data_Sheet_3.zip › mitochondrial membrane potential (VPC)/vpc3 绿.tif]

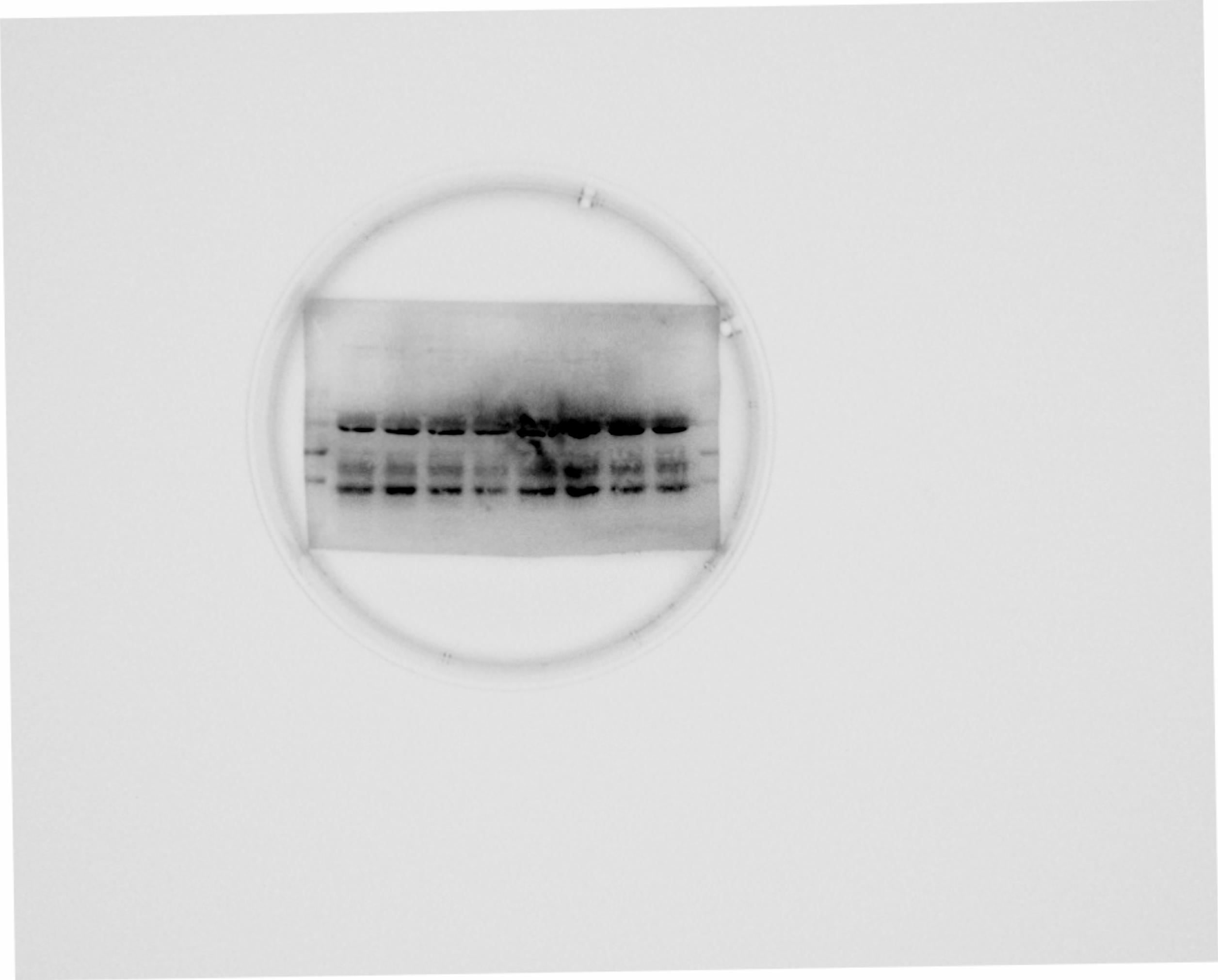

Supplement: Supplementary file 5 [file Data_Sheet_4.zip › in vivo(HNRNP A1)/TG WT/original data/2022-09-29 5a.tif]

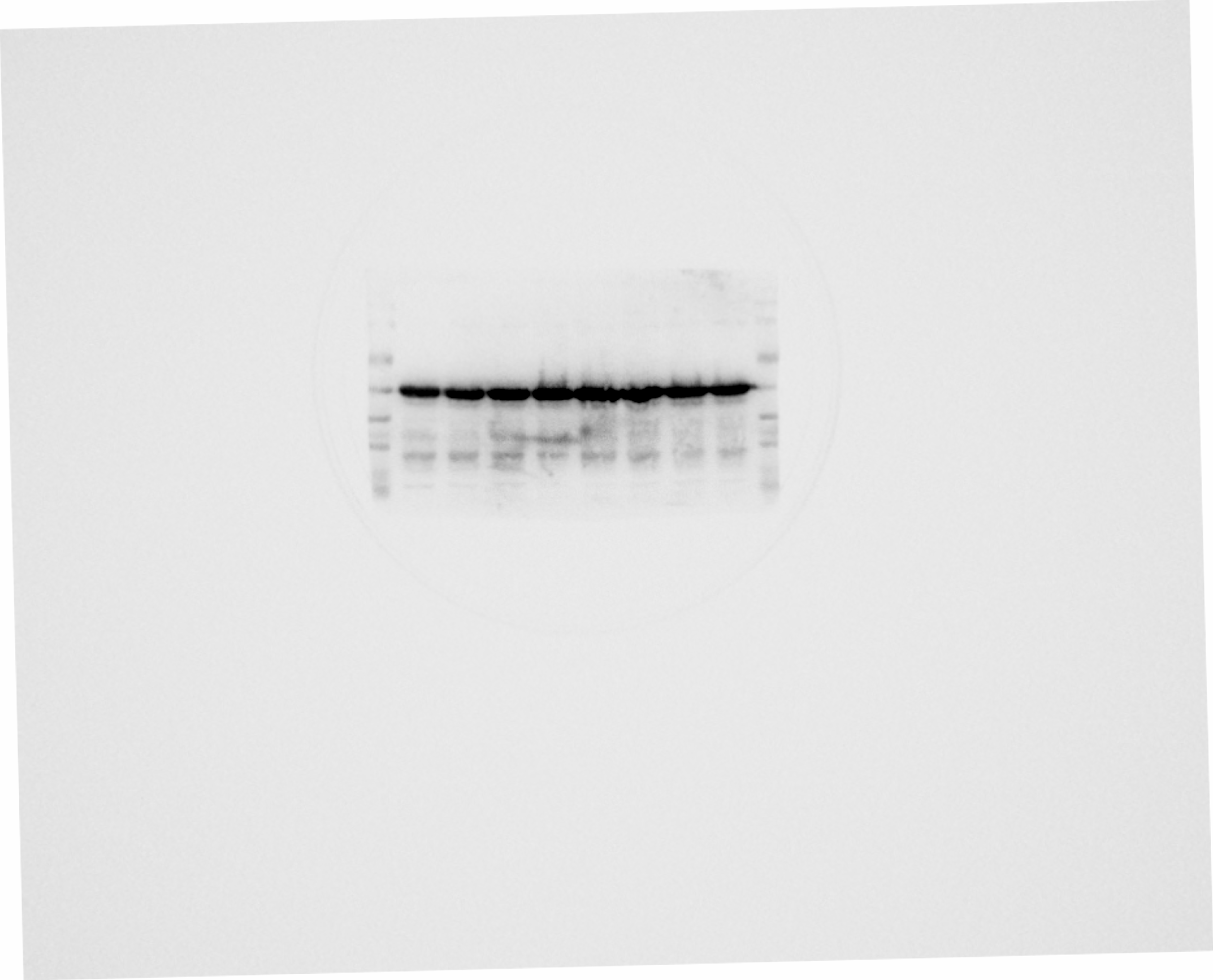

Supplement: Supplementary file 5 [file Data_Sheet_4.zip › in vivo(HNRNP A1)/TG WT/original data/2022-10-01 5tub.tif]

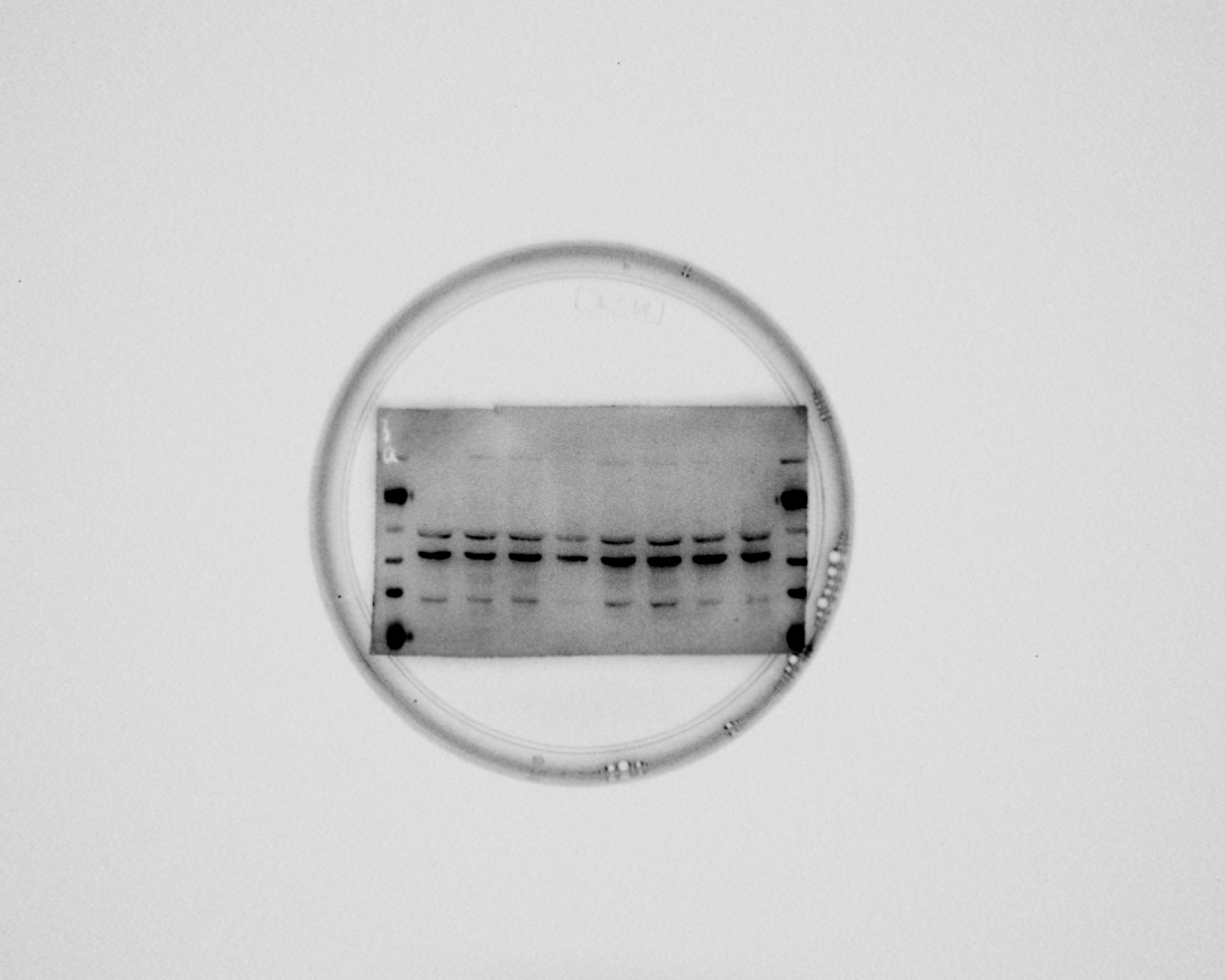

Supplement: Supplementary file 5 [file Data_Sheet_4.zip › in vivo(HNRNP A1)/TG WT/original data/2022-10-17 10'16 3a.tif]

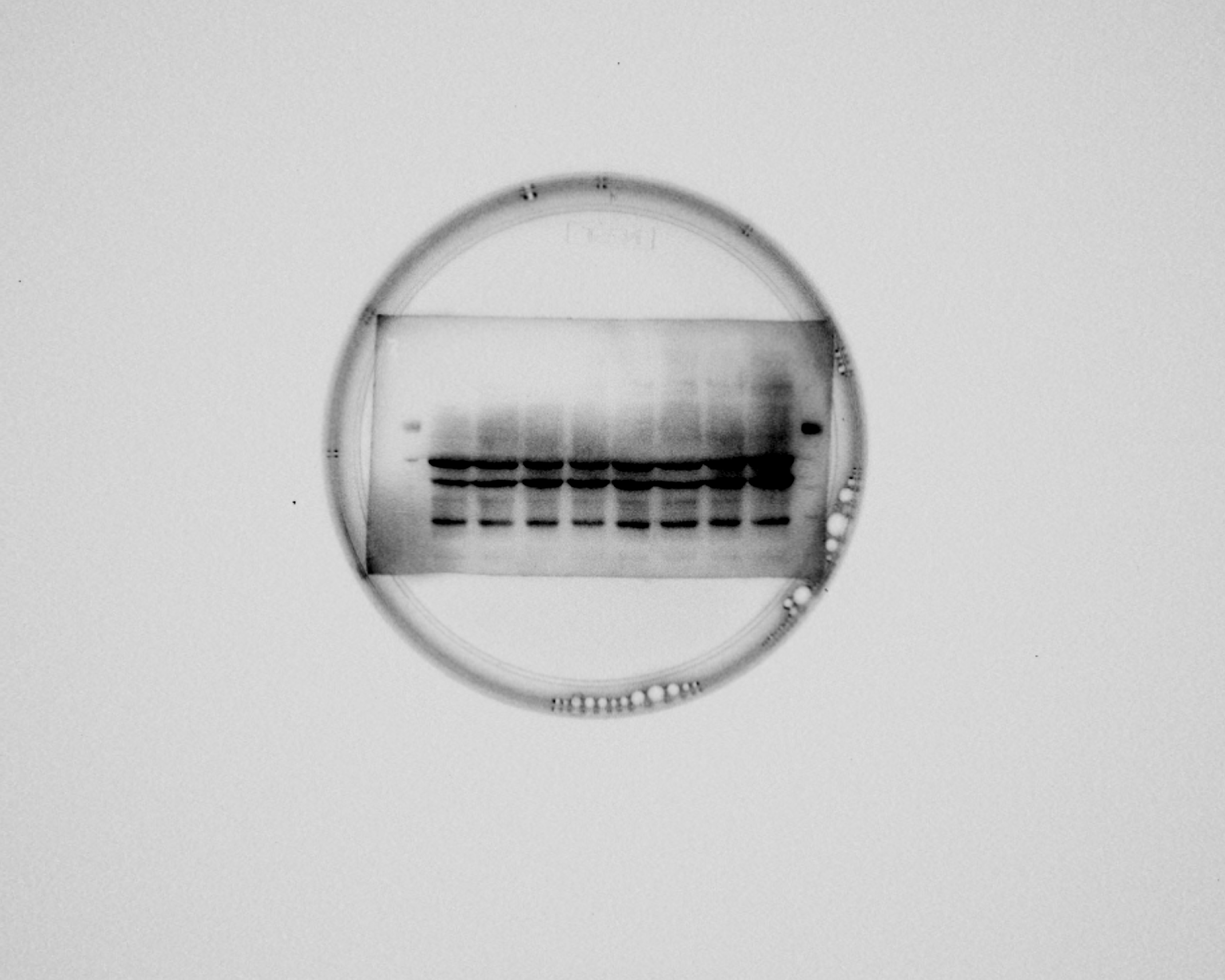

Supplement: Supplementary file 5 [file Data_Sheet_4.zip › in vivo(HNRNP A1)/TG WT/original data/2022-10-17 2a.tif]

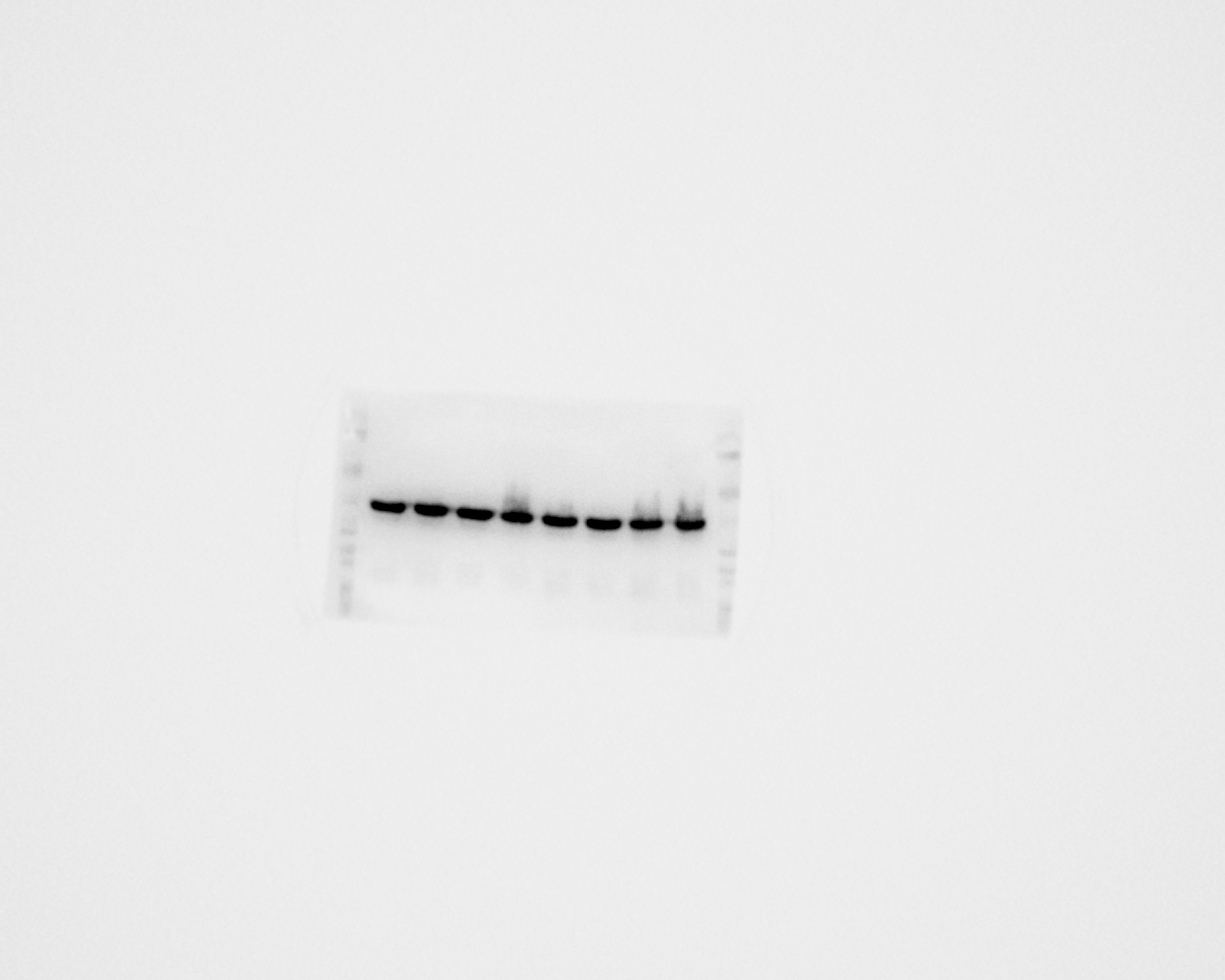

Supplement: Supplementary file 5 [file Data_Sheet_4.zip › in vivo(HNRNP A1)/TG WT/original data/2022-10-18 10'16 3tub.tif]

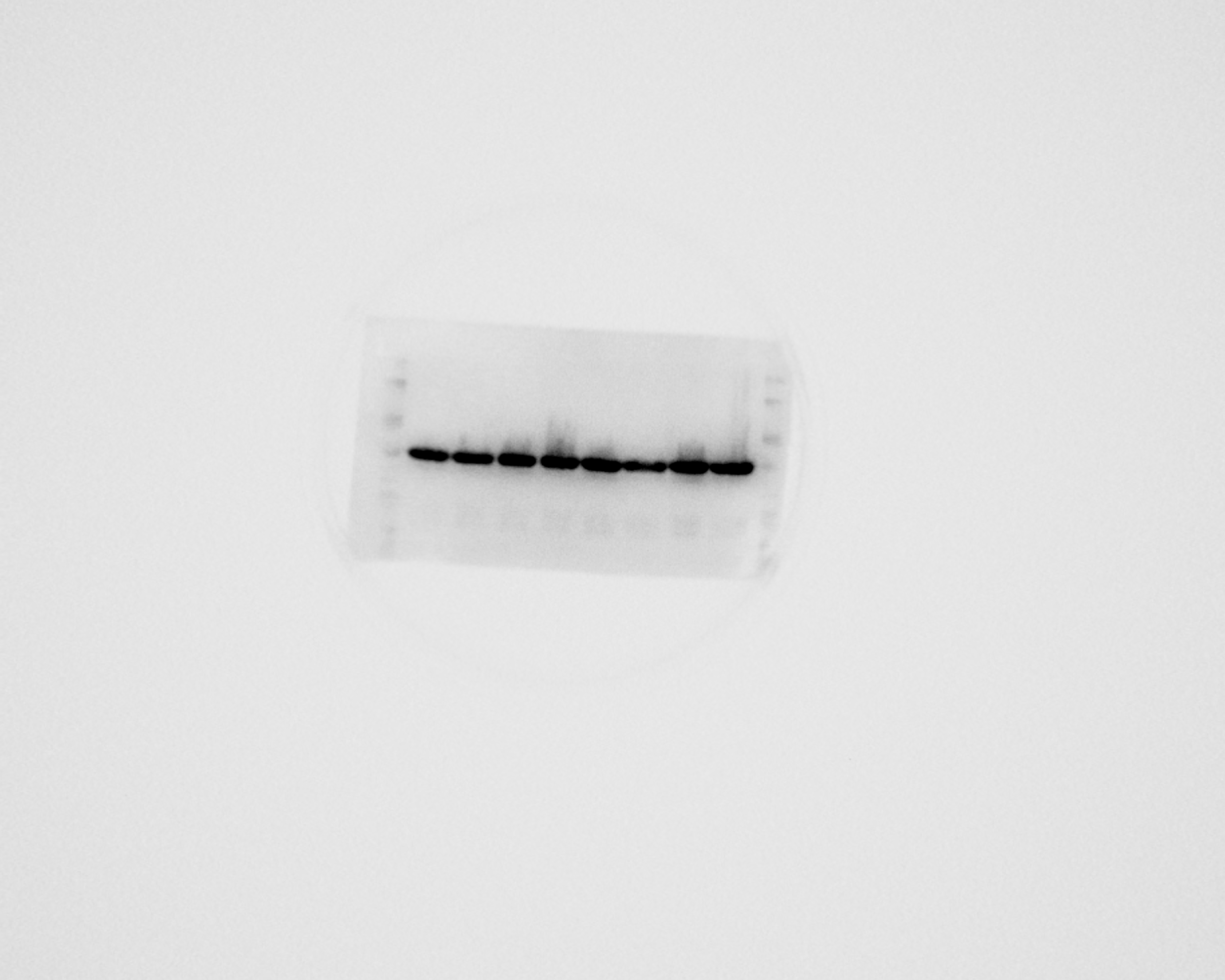

Supplement: Supplementary file 5 [file Data_Sheet_4.zip › in vivo(HNRNP A1)/TG WT/original data/2022-10-18 10'17 2tub.tif]

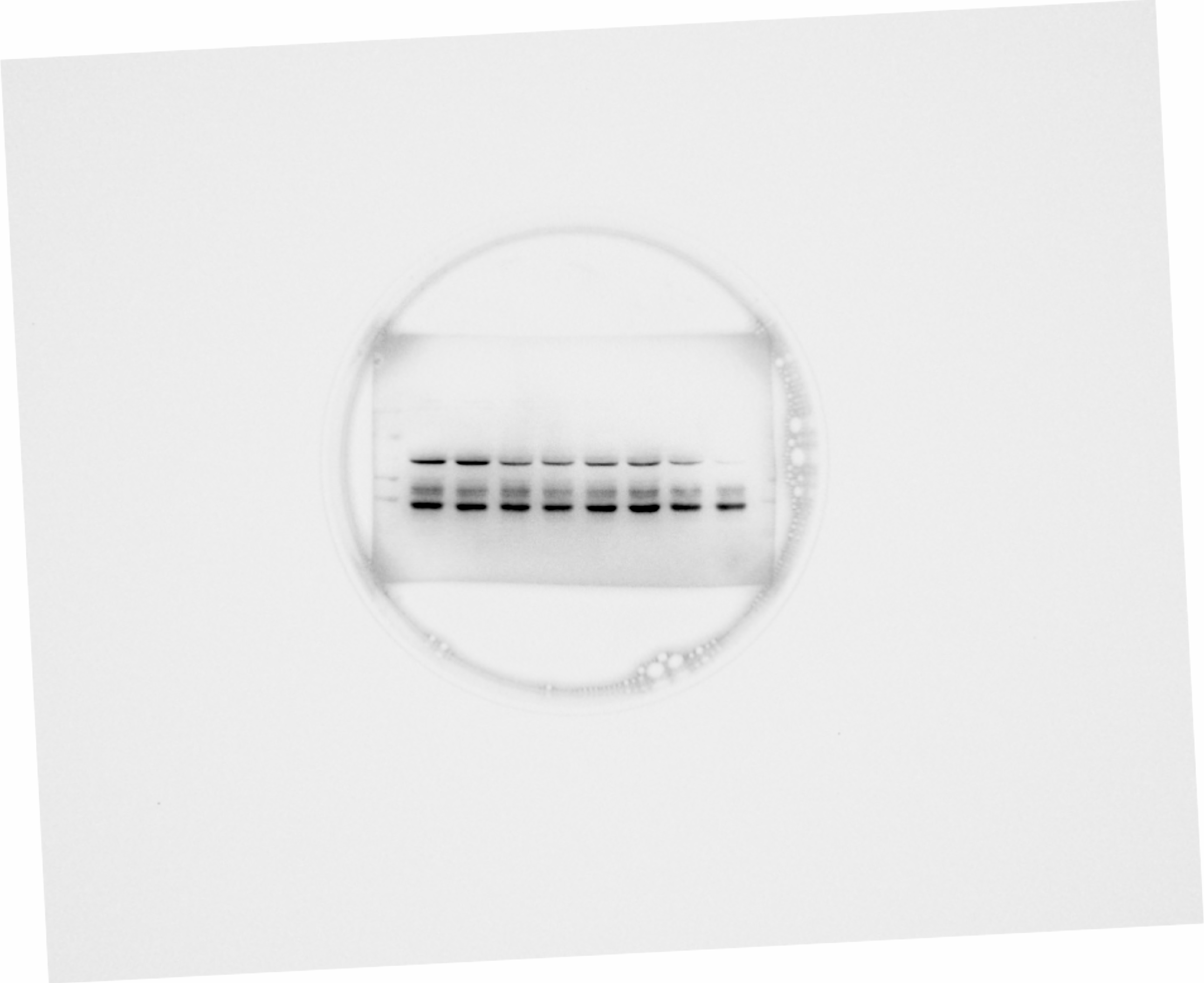

Supplement: Supplementary file 5 [file Data_Sheet_4.zip › in vivo(HNRNP A1)/WT TG/original data/2022-09-29 3a.tif]

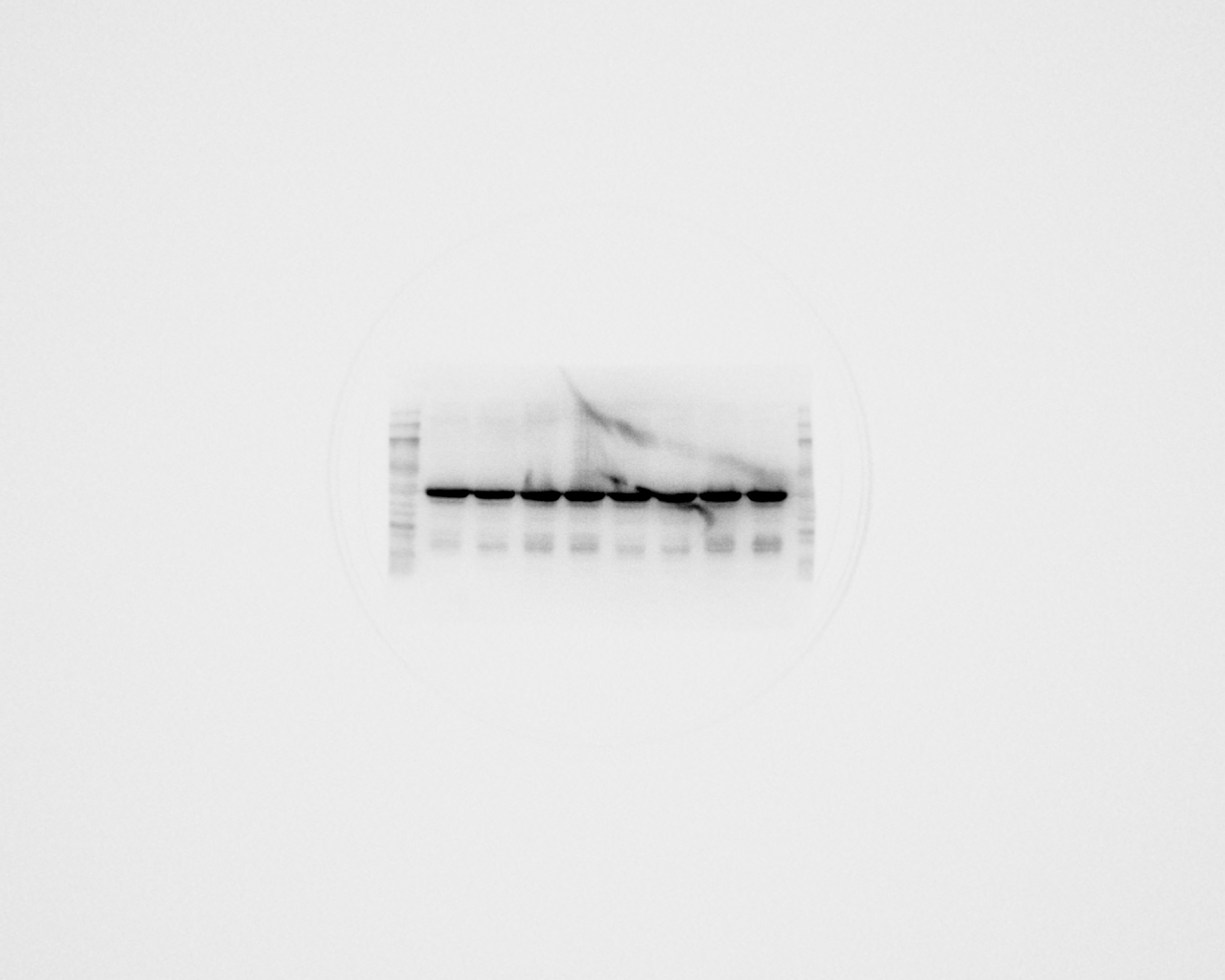

Supplement: Supplementary file 5 [file Data_Sheet_4.zip › in vivo(HNRNP A1)/WT TG/original data/2022-10-01 3tub.tif]

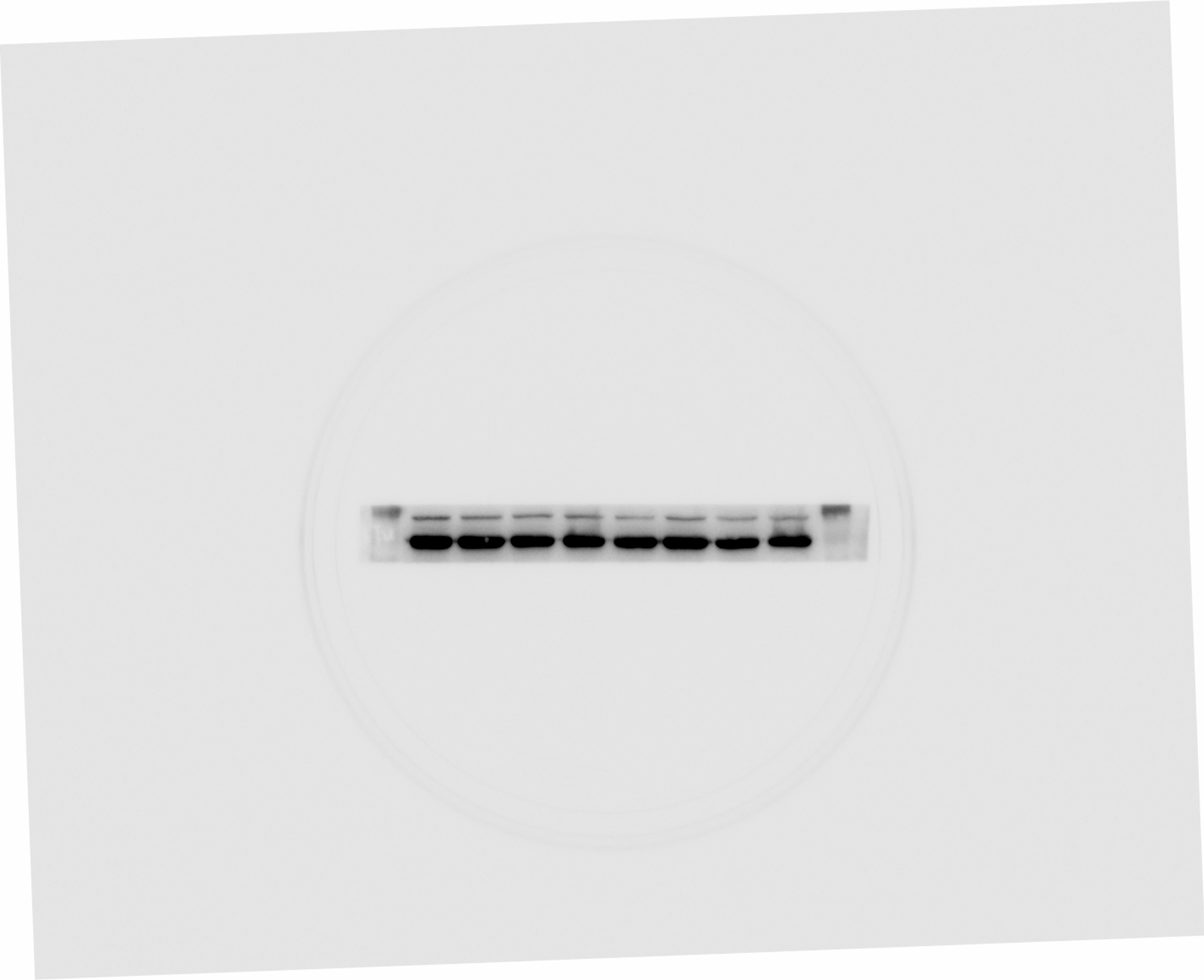

Supplement: Supplementary file 5 [file Data_Sheet_4.zip › in vivo(HNRNP A1)/WT TG/original data/wb 2022-09-11 1tub.tif]

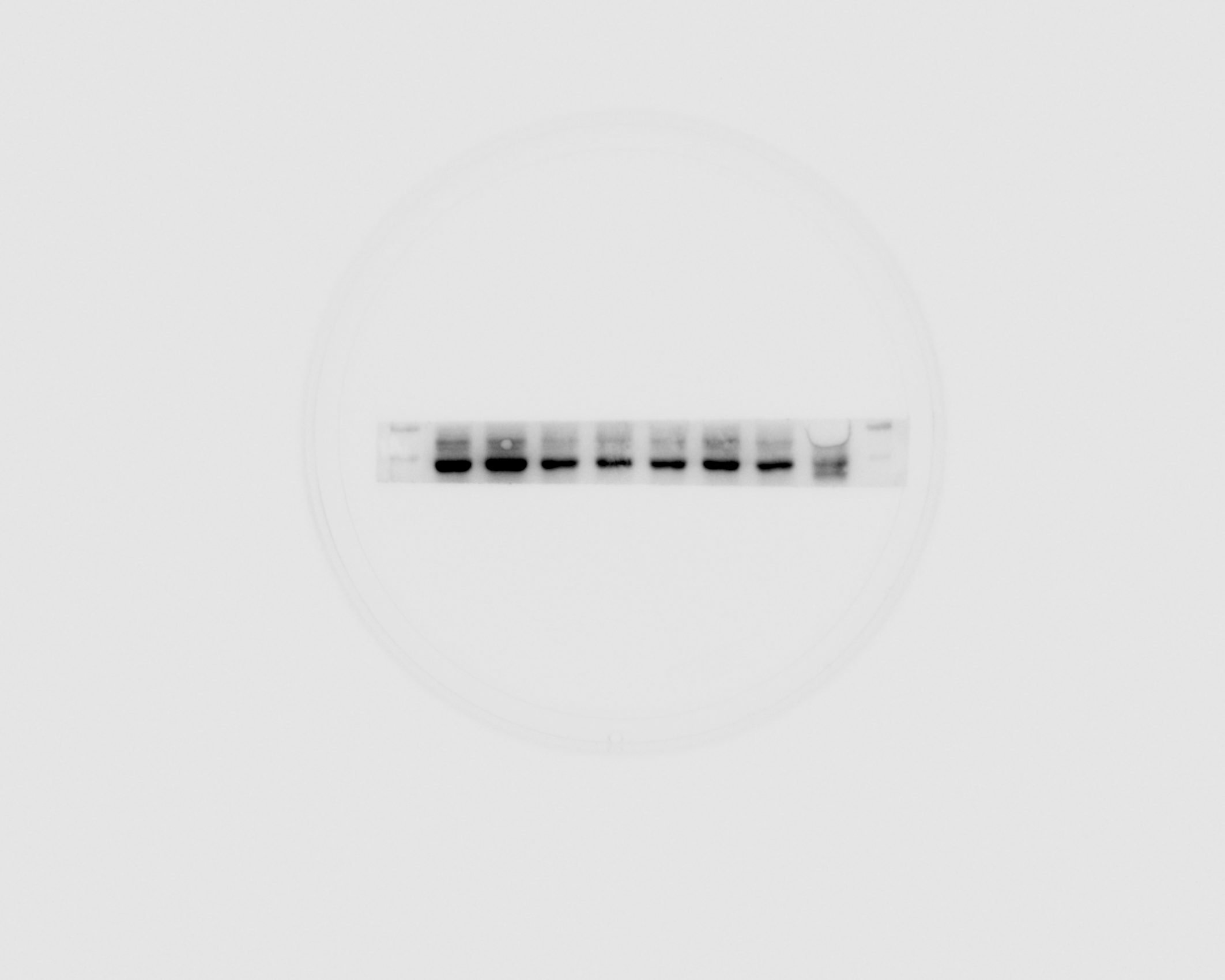

Supplement: Supplementary file 5 [file Data_Sheet_4.zip › in vivo(HNRNP A1)/WT TG/original data/wb 2022-09-11 1a.tif]

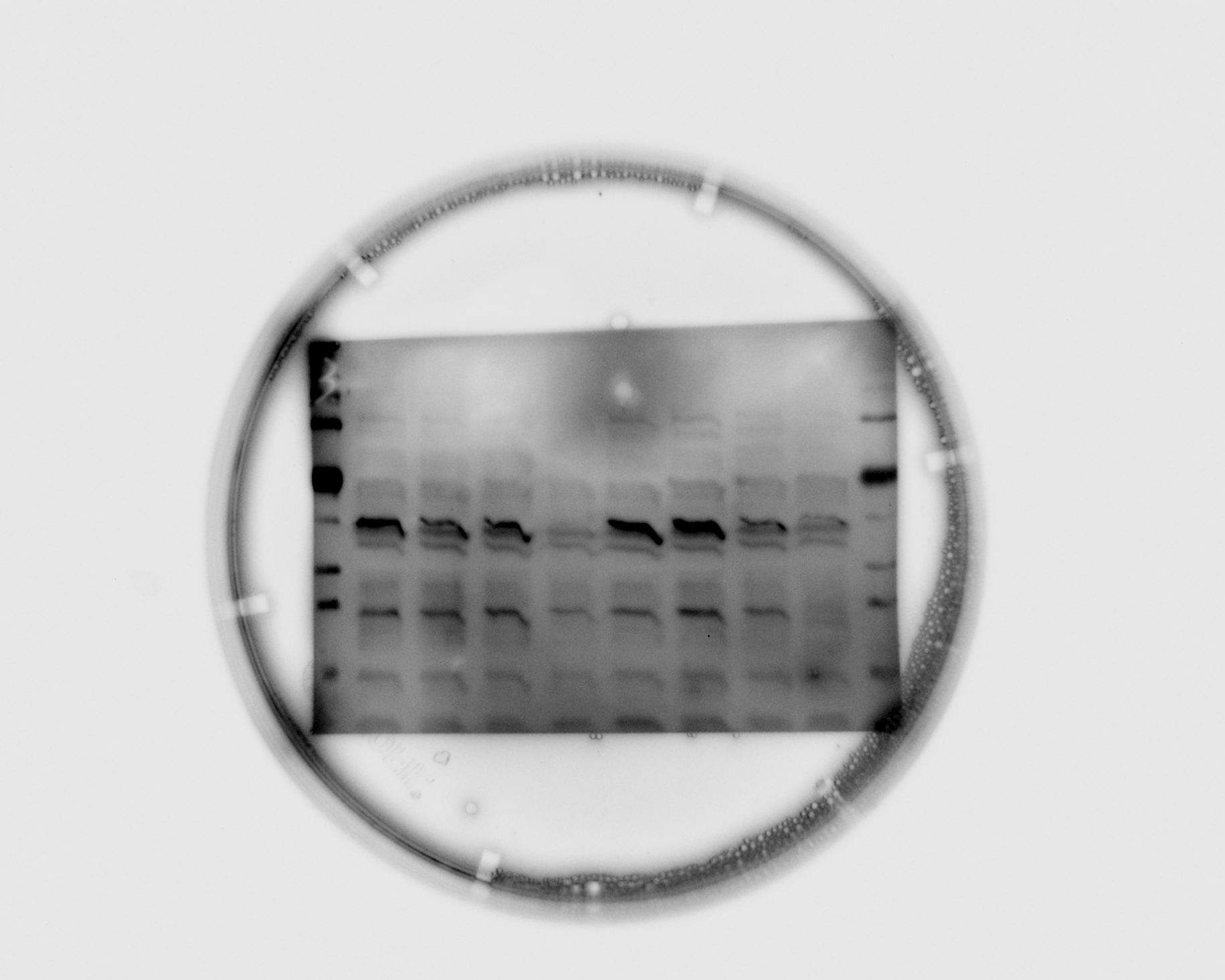

Supplement: Supplementary file 5 [file Data_Sheet_4.zip › in vivo(HNRNP A1)/WT TG/original data/wb 2022-11-01 3a.tif]

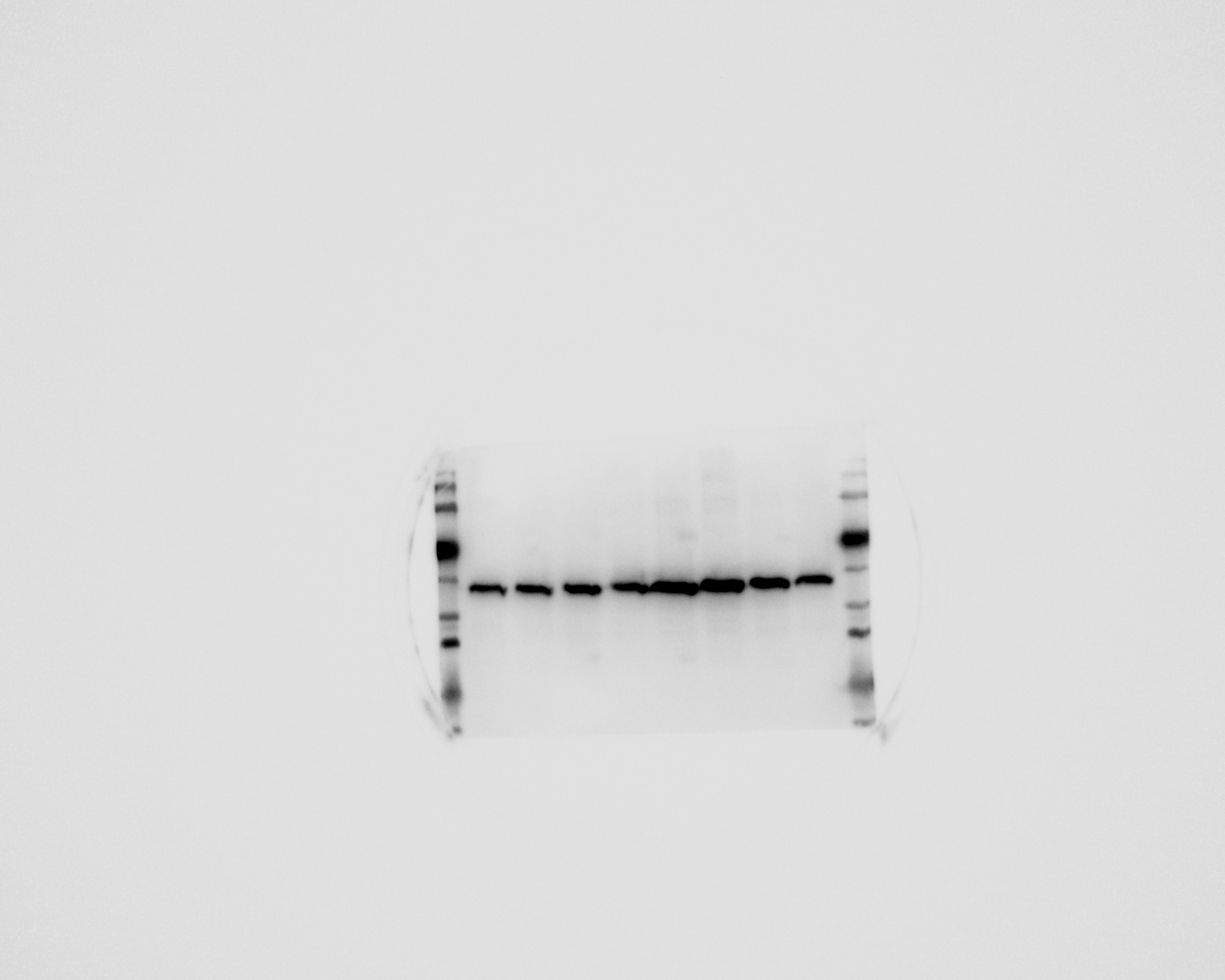

Supplement: Supplementary file 5 [file Data_Sheet_4.zip › in vivo(HNRNP A1)/WT TG/original data/wb 2022-11-05 3tub.tif]

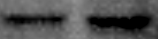

Supplement: Supplementary file 6 [file Data_Sheet_6.zip › FIG5/Aβ APP(VPC)/Aβ/2022-10-20 jxh 6'1ab.png]

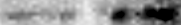

Supplement: Supplementary file 6 [file Data_Sheet_6.zip › FIG5/Aβ APP(VPC)/Aβ/2022-10-20 jxh 8'1ab.png]

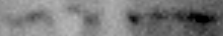

Supplement: Supplementary file 6 [file Data_Sheet_6.zip › FIG5/Aβ APP(VPC)/Aβ/2022-10-20 jxh 8'2ab.png]

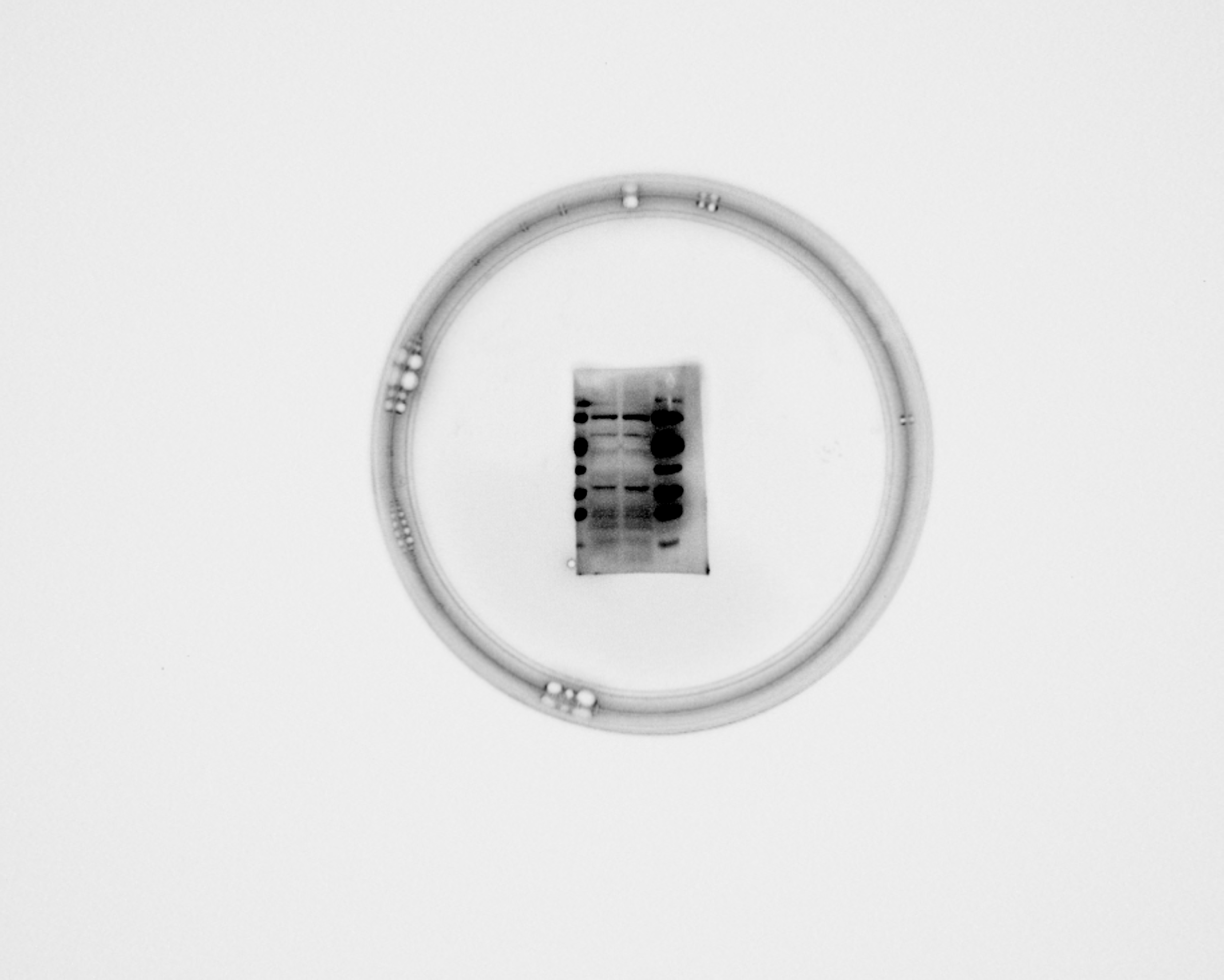

Supplement: Supplementary file 6 [file Data_Sheet_6.zip › FIG5/Aβ APP(VPC)/Aβ/original data/2022-10-20 6'1ab.tif]

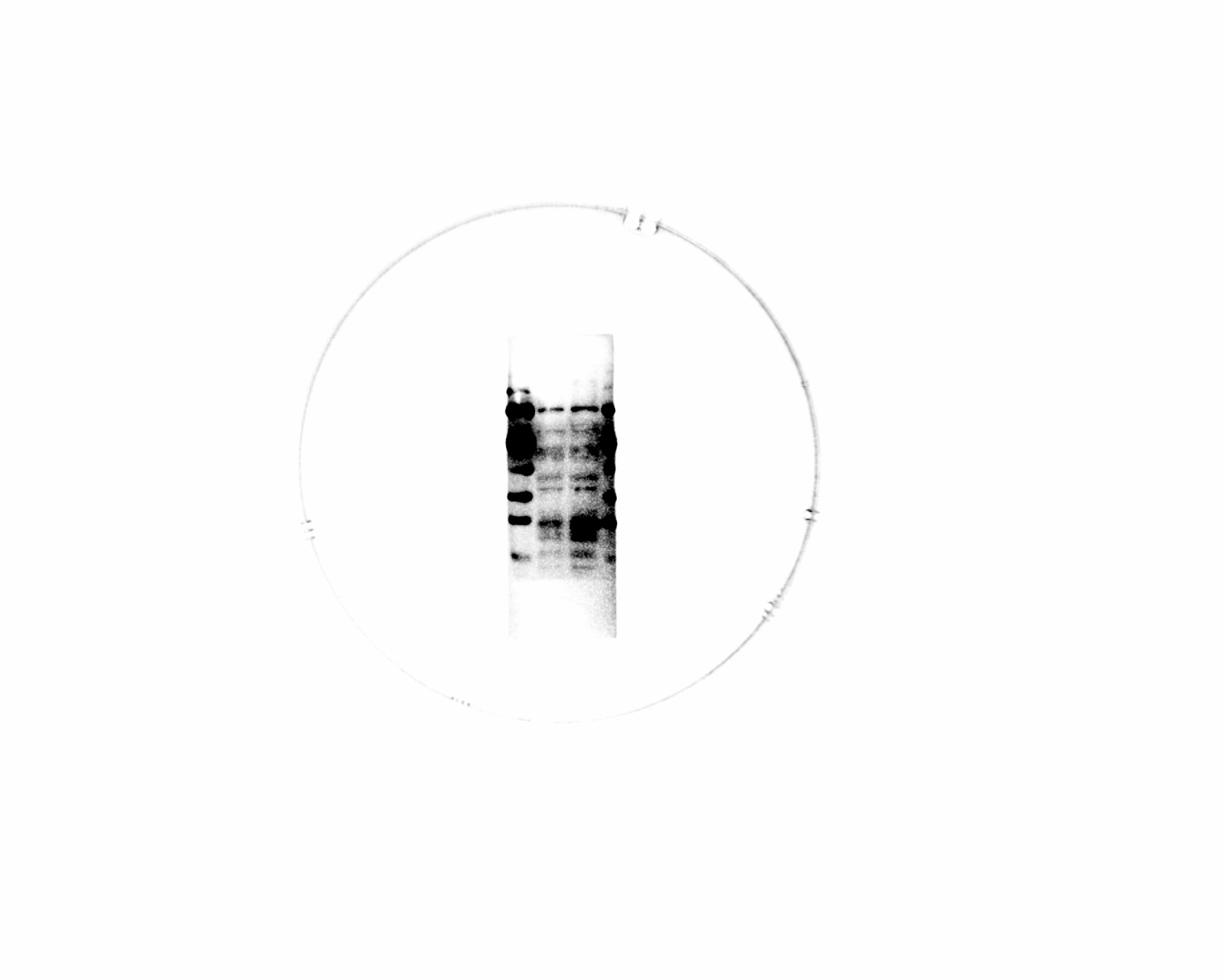

Supplement: Supplementary file 6 [file Data_Sheet_6.zip › FIG5/Aβ APP(VPC)/Aβ/original data/2022-10-20 8'1ab.tif]

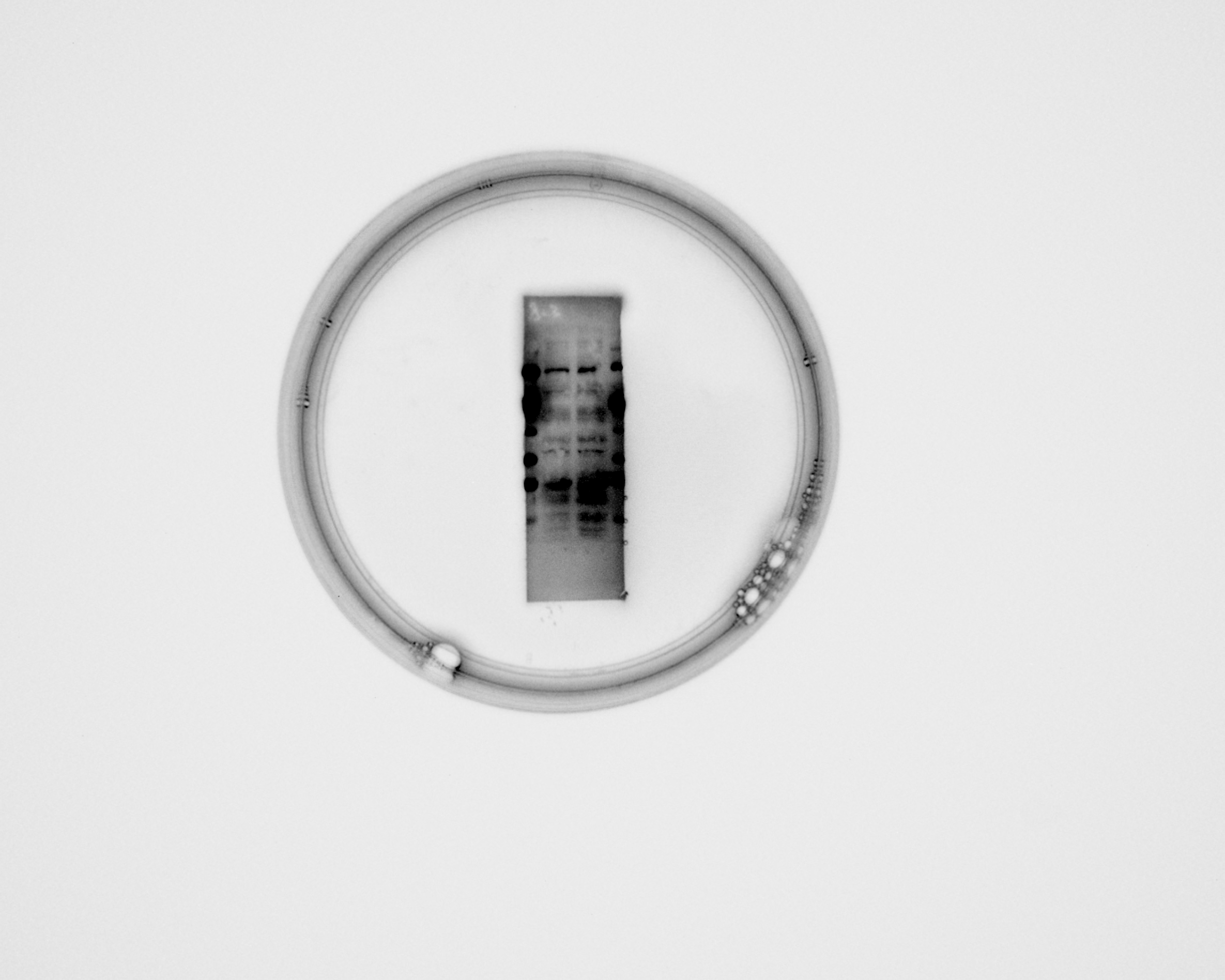

Supplement: Supplementary file 6 [file Data_Sheet_6.zip › FIG5/Aβ APP(VPC)/Aβ/original data/2022-10-20 8'2ab.tif]

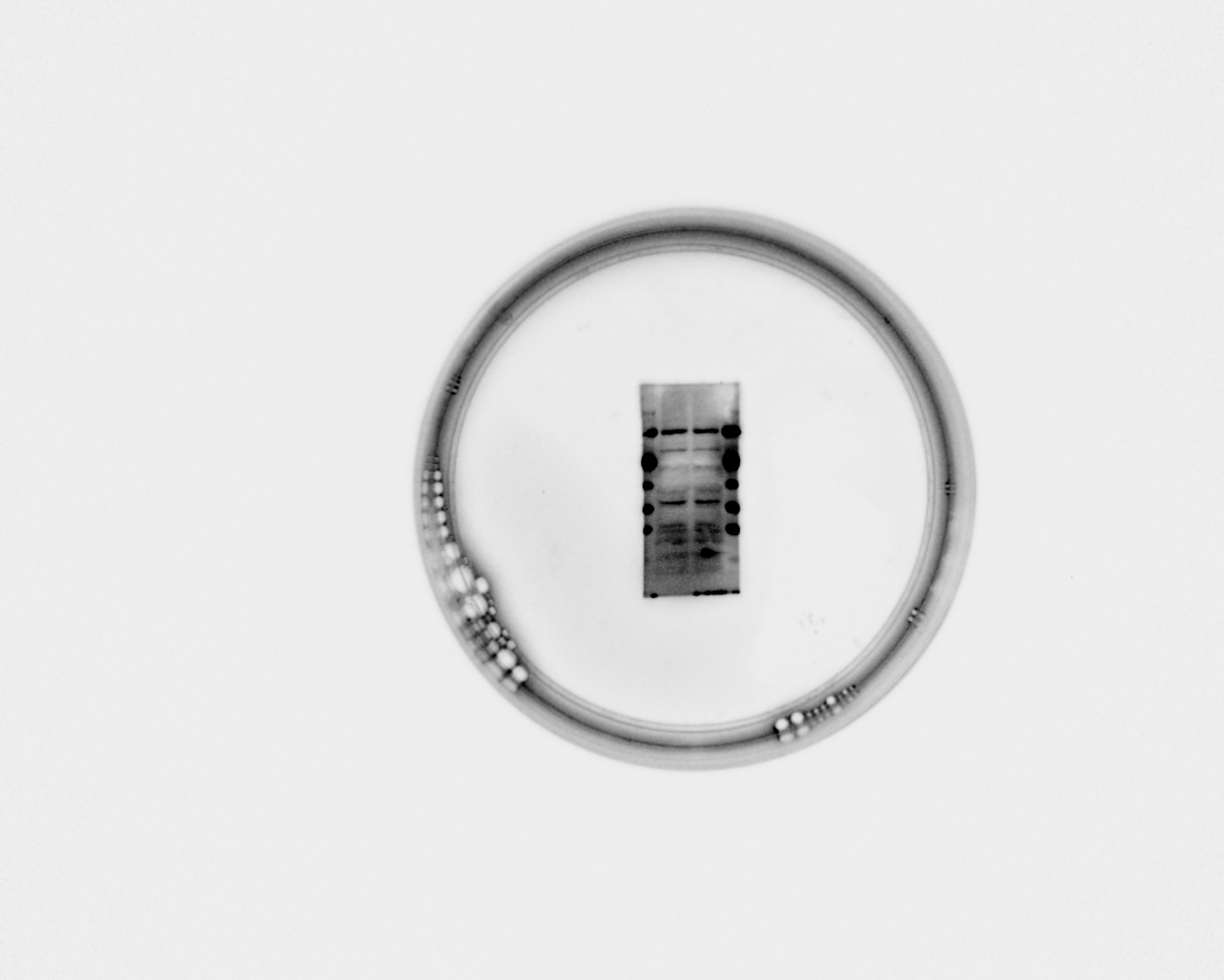

Supplement: Supplementary file 6 [file Data_Sheet_6.zip › FIG5/Aβ APP(VPC)/Aβ/original data/2022-10-20 6'3ab.tif]

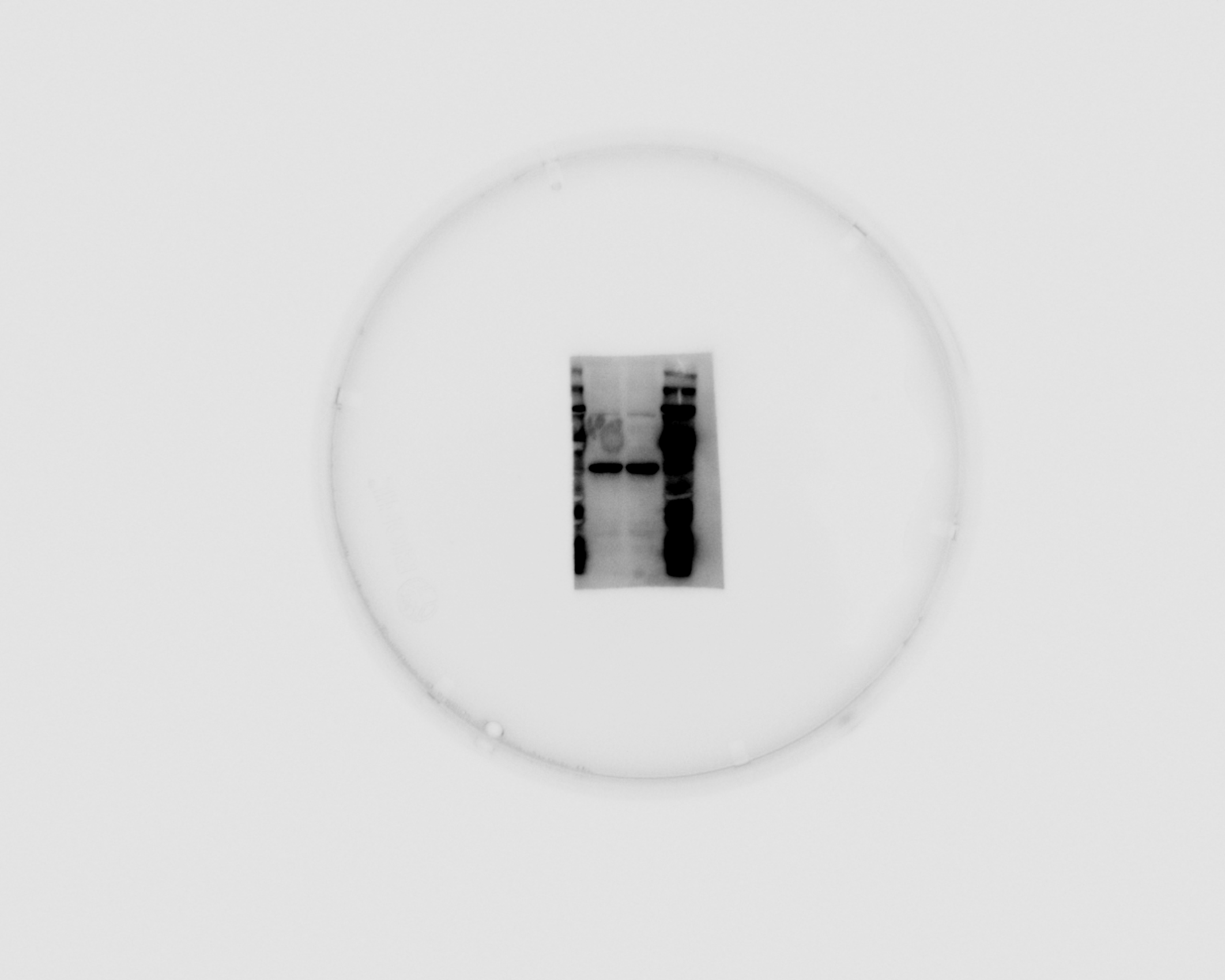

Supplement: Supplementary file 6 [file Data_Sheet_6.zip › FIG5/Aβ APP(VPC)/Aβ/original data/wb 2022-10-20 6'1tub.tif]

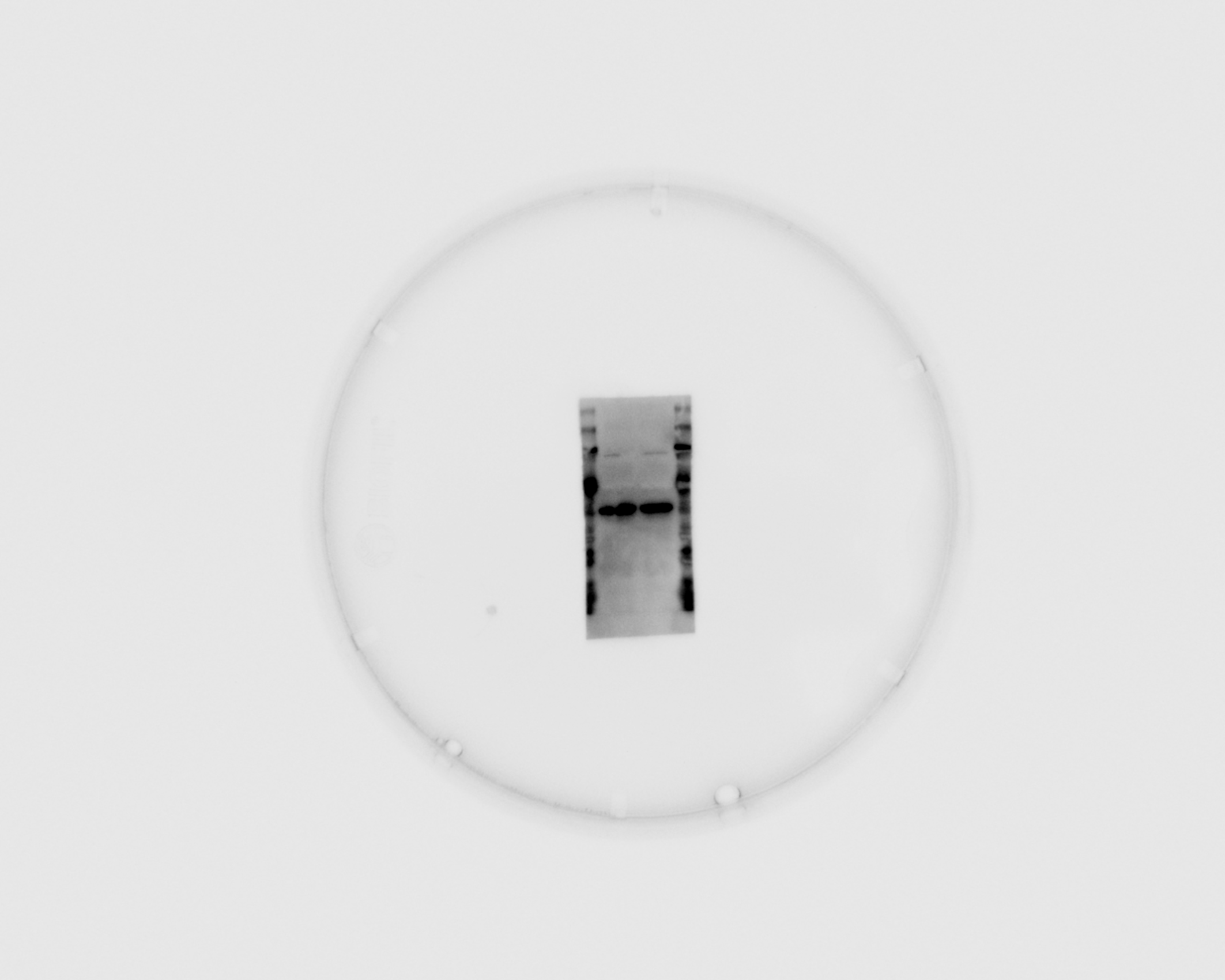

Supplement: Supplementary file 6 [file Data_Sheet_6.zip › FIG5/Aβ APP(VPC)/Aβ/original data/wb 2022-10-20 6'3tub.tif]

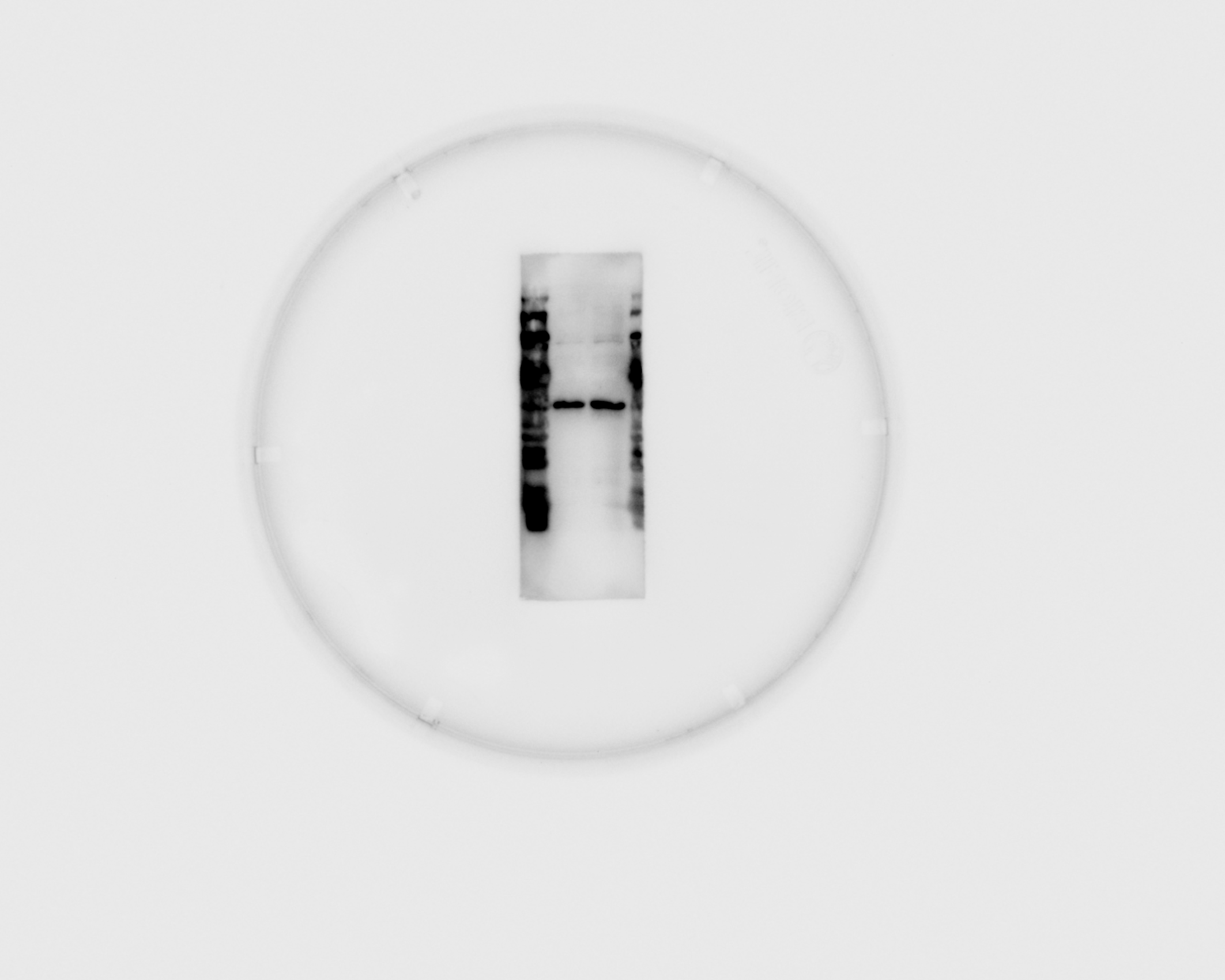

Supplement: Supplementary file 6 [file Data_Sheet_6.zip › FIG5/Aβ APP(VPC)/Aβ/original data/wb 2022-10-20 8'1tub.tif]

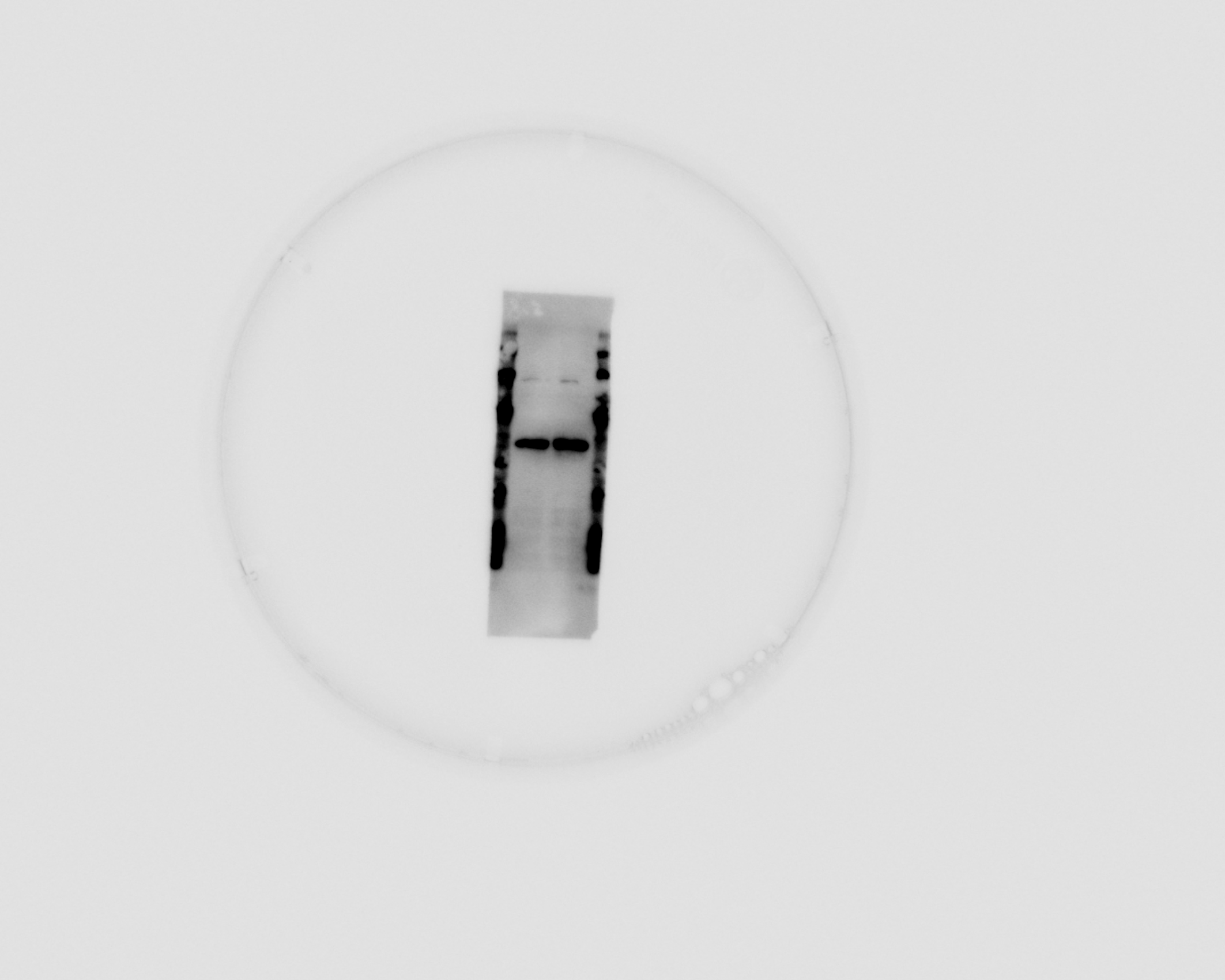

Supplement: Supplementary file 6 [file Data_Sheet_6.zip › FIG5/Aβ APP(VPC)/Aβ/original data/wb 2022-10-20 8'2tub.tif]

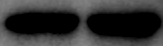

Supplement: Supplementary file 6 [file Data_Sheet_6.zip › FIG5/Aβ APP(VPC)/Aβ/wb 2022-10-20 jxh 6'1tub.png]

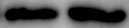

Supplement: Supplementary file 6 [file Data_Sheet_6.zip › FIG5/Aβ APP(VPC)/Aβ/wb 2022-10-20 jxh 8'1tub.png]

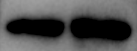

Supplement: Supplementary file 6 [file Data_Sheet_6.zip › FIG5/Aβ APP(VPC)/Aβ/wb 2022-10-20 jxh 8'2tub.png]

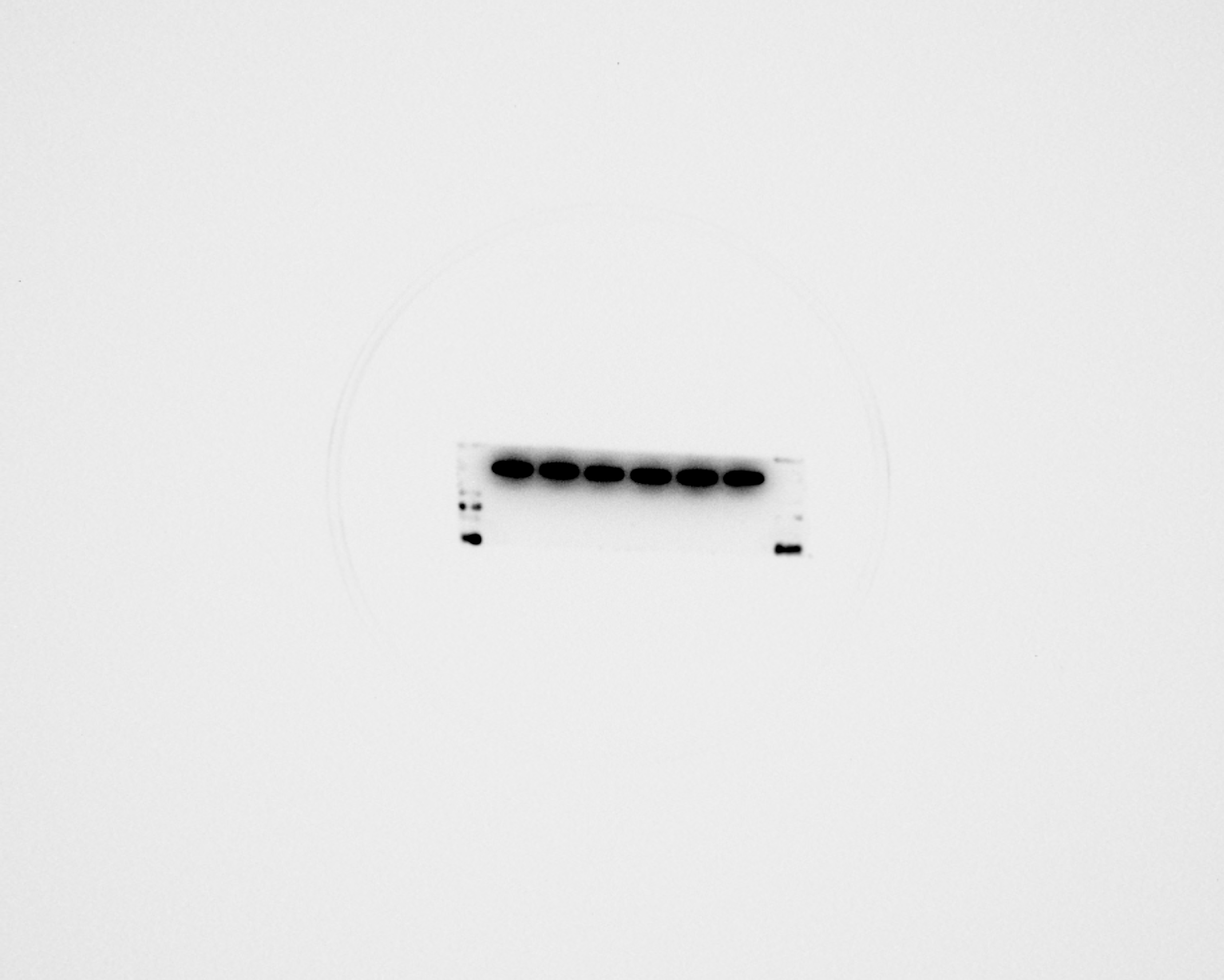

Supplement: Supplementary file 6 [file Data_Sheet_6.zip › FIG5/Aβ APP(VPC)/app/original data/2022-10-09 10'4 1tub.tif]

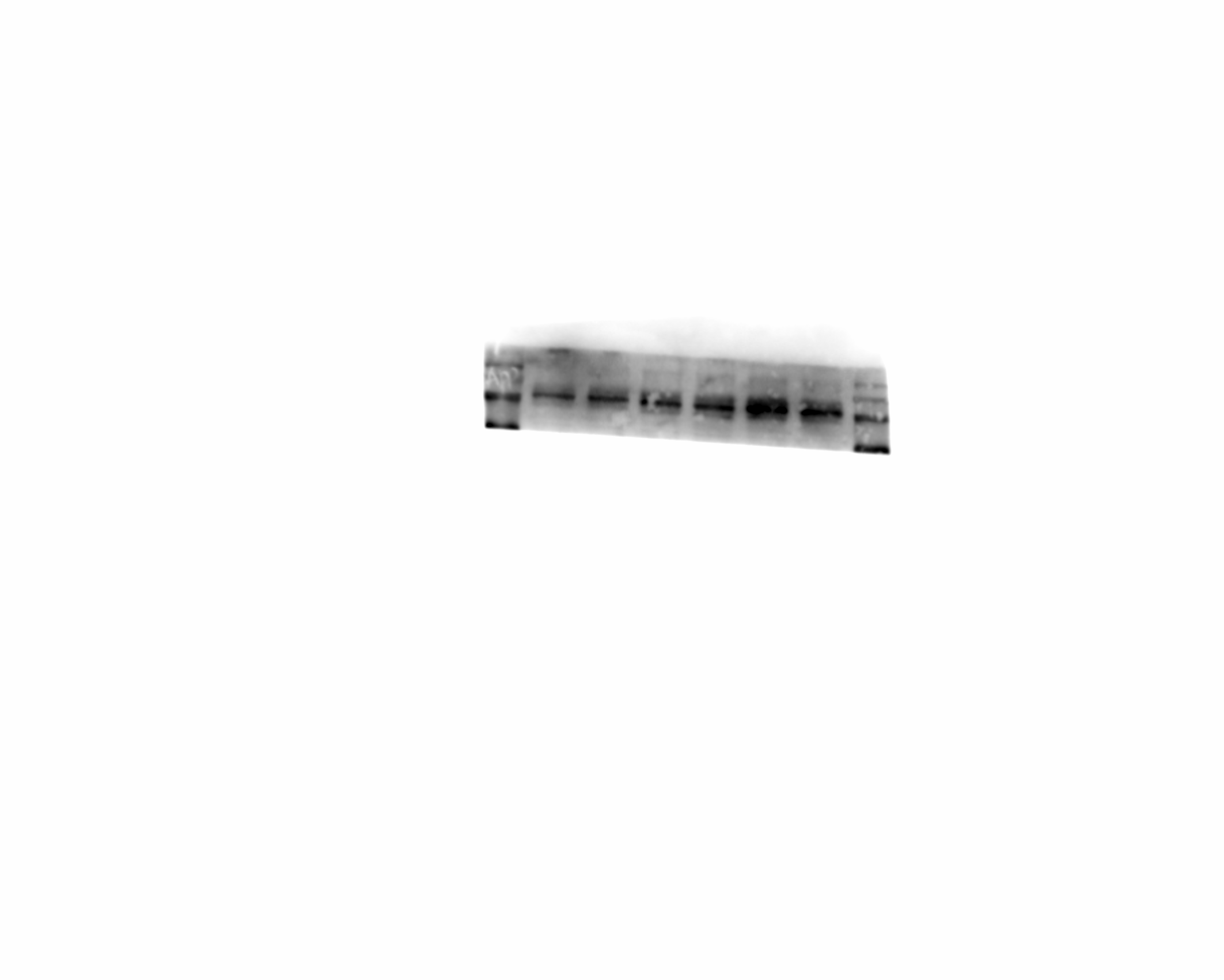

Supplement: Supplementary file 6 [file Data_Sheet_6.zip › FIG5/Aβ APP(VPC)/app/original data/wb 2022-10-03 1app.tif]

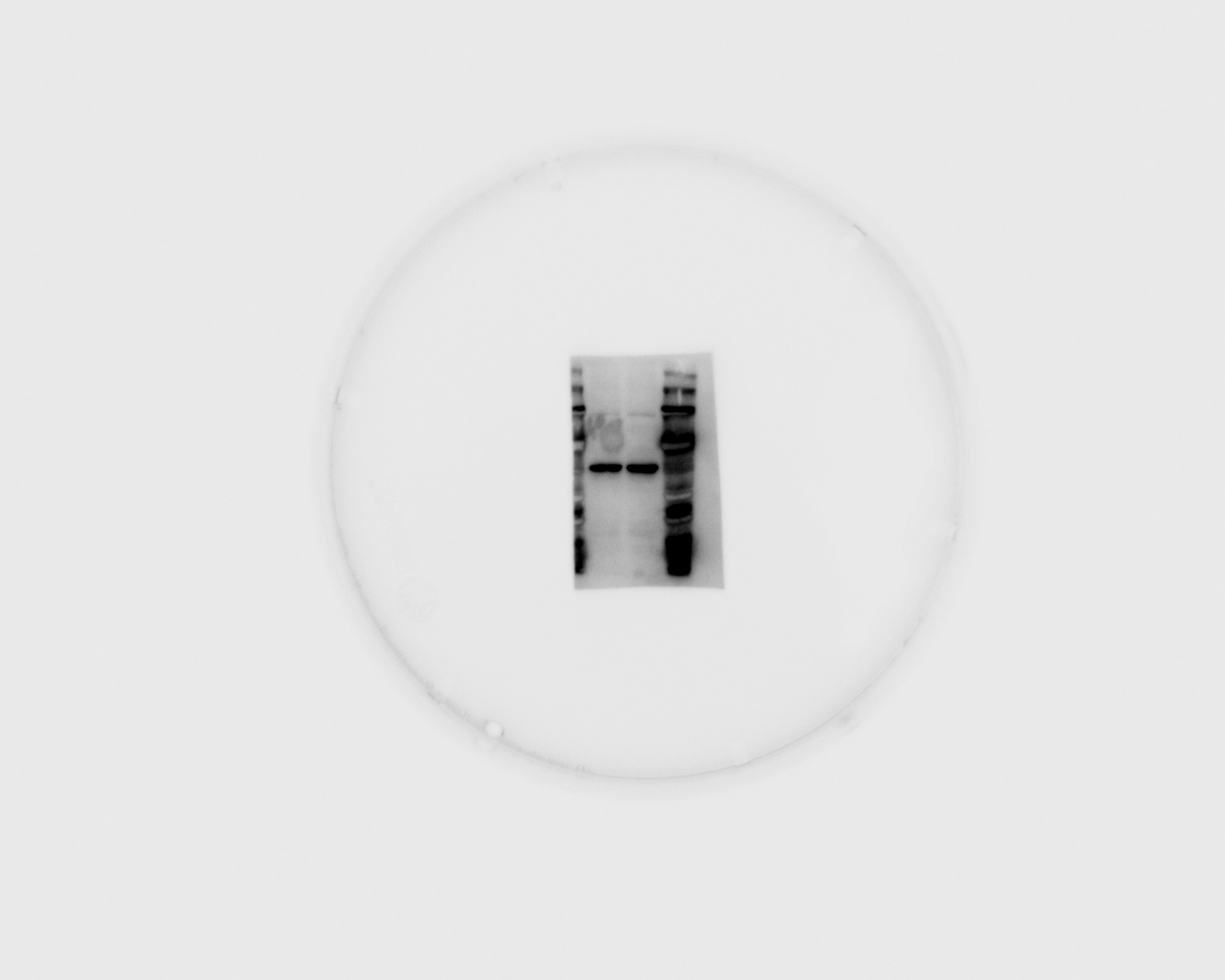

Supplement: Supplementary file 6 [file Data_Sheet_6.zip › FIG5/Aβ APP(VPC)/app/original data/wb 2022-10-20 6'1tub.tif]

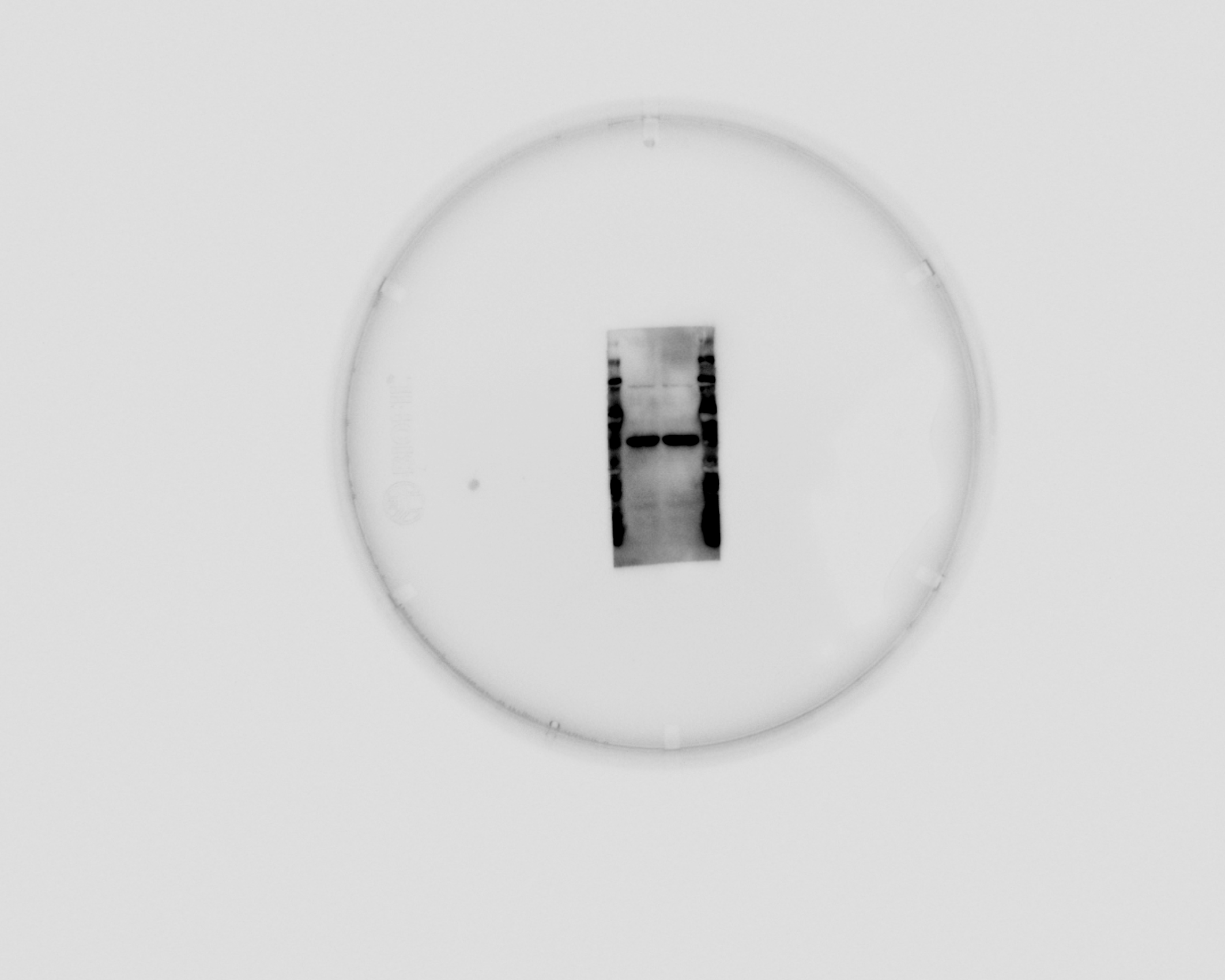

Supplement: Supplementary file 6 [file Data_Sheet_6.zip › FIG5/Aβ APP(VPC)/app/original data/wb 2022-10-20 6'2tub.tif]

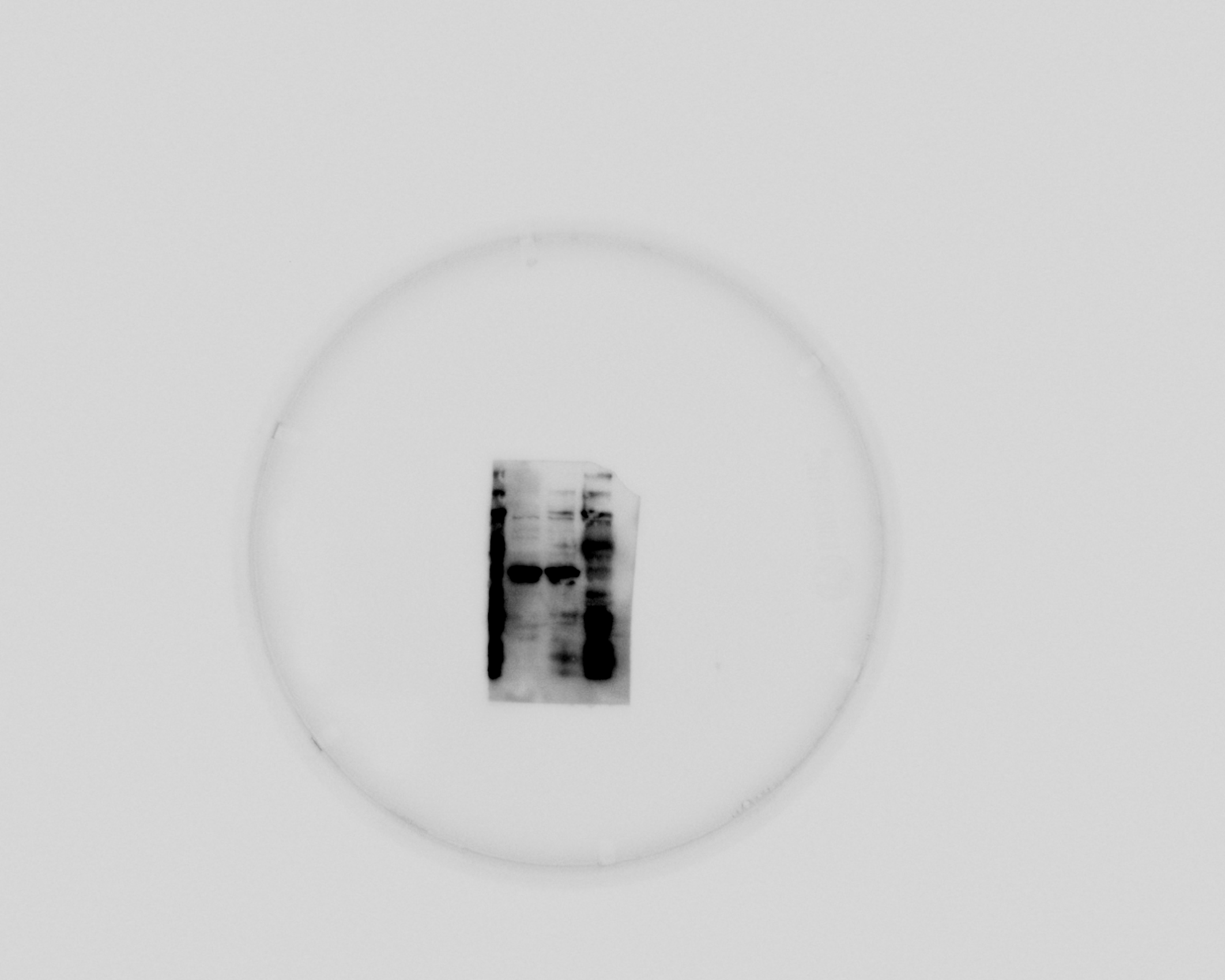

Supplement: Supplementary file 6 [file Data_Sheet_6.zip › FIG5/Aβ APP(VPC)/app/original data/wb 2022-10-20 6'4tub.tif]

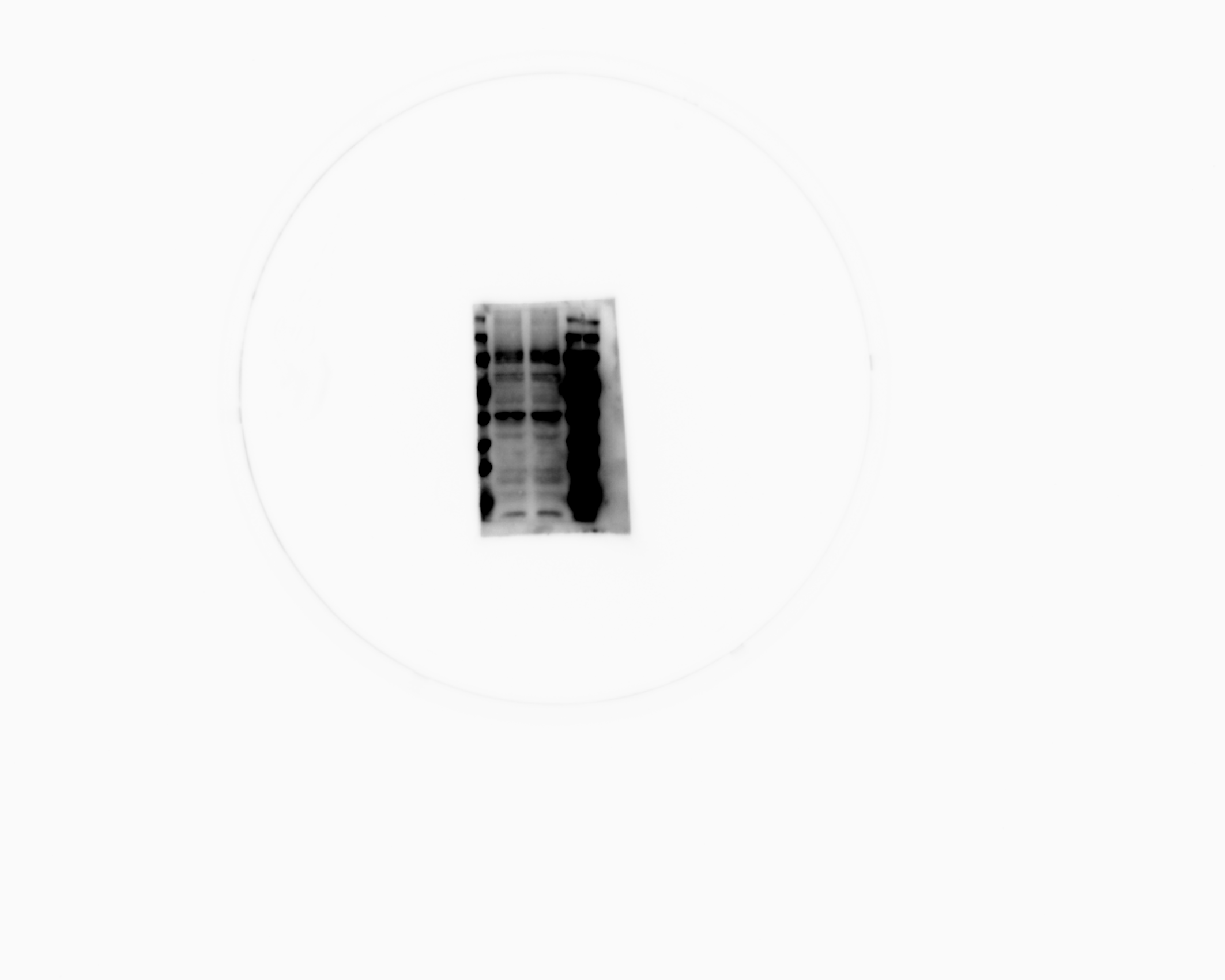

Supplement: Supplementary file 6 [file Data_Sheet_6.zip › FIG5/Aβ APP(VPC)/app/original data/wb 2022-10-22 6'1app.tif]

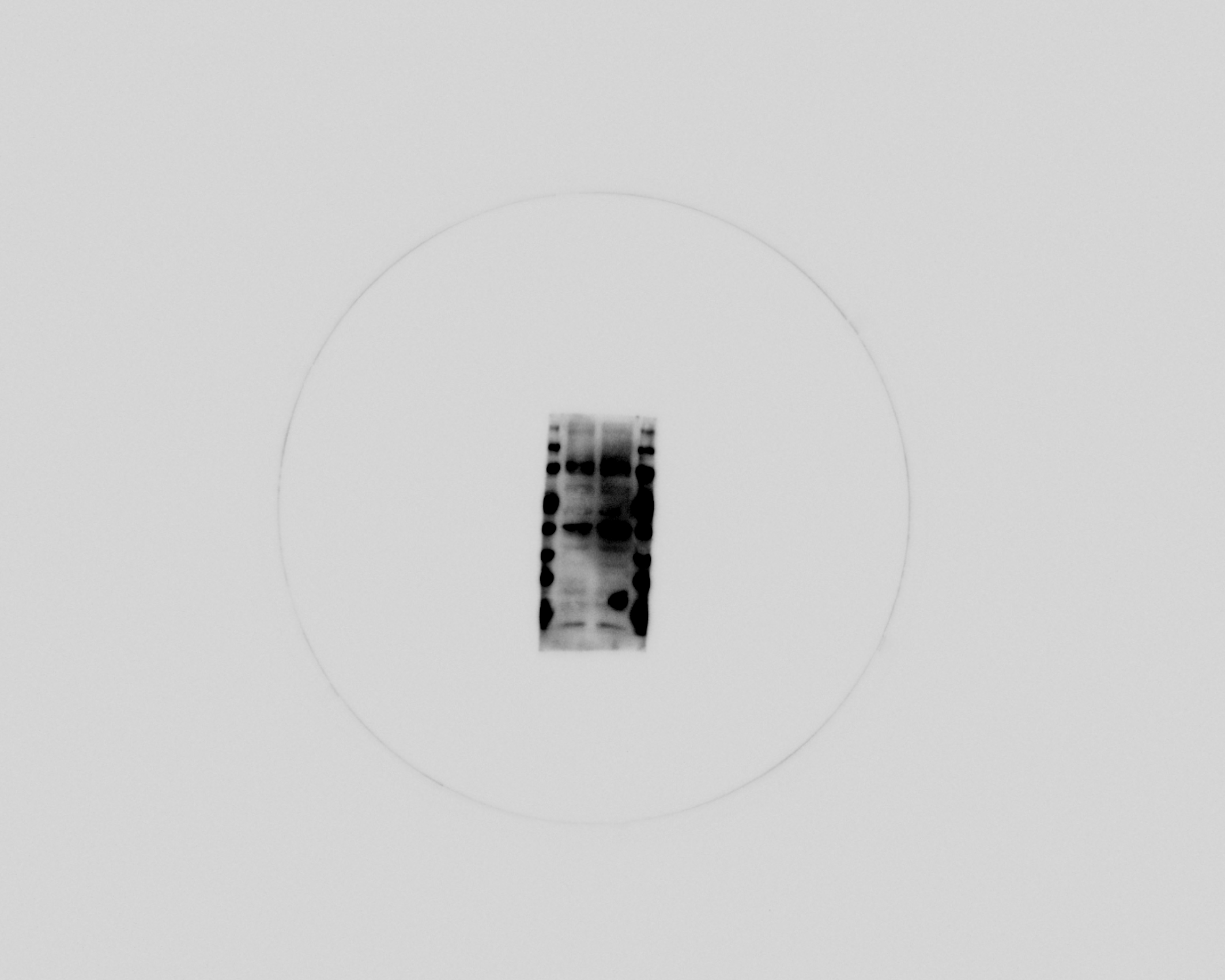

Supplement: Supplementary file 6 [file Data_Sheet_6.zip › FIG5/Aβ APP(VPC)/app/original data/wb 2022-10-22 6'2app.tif]

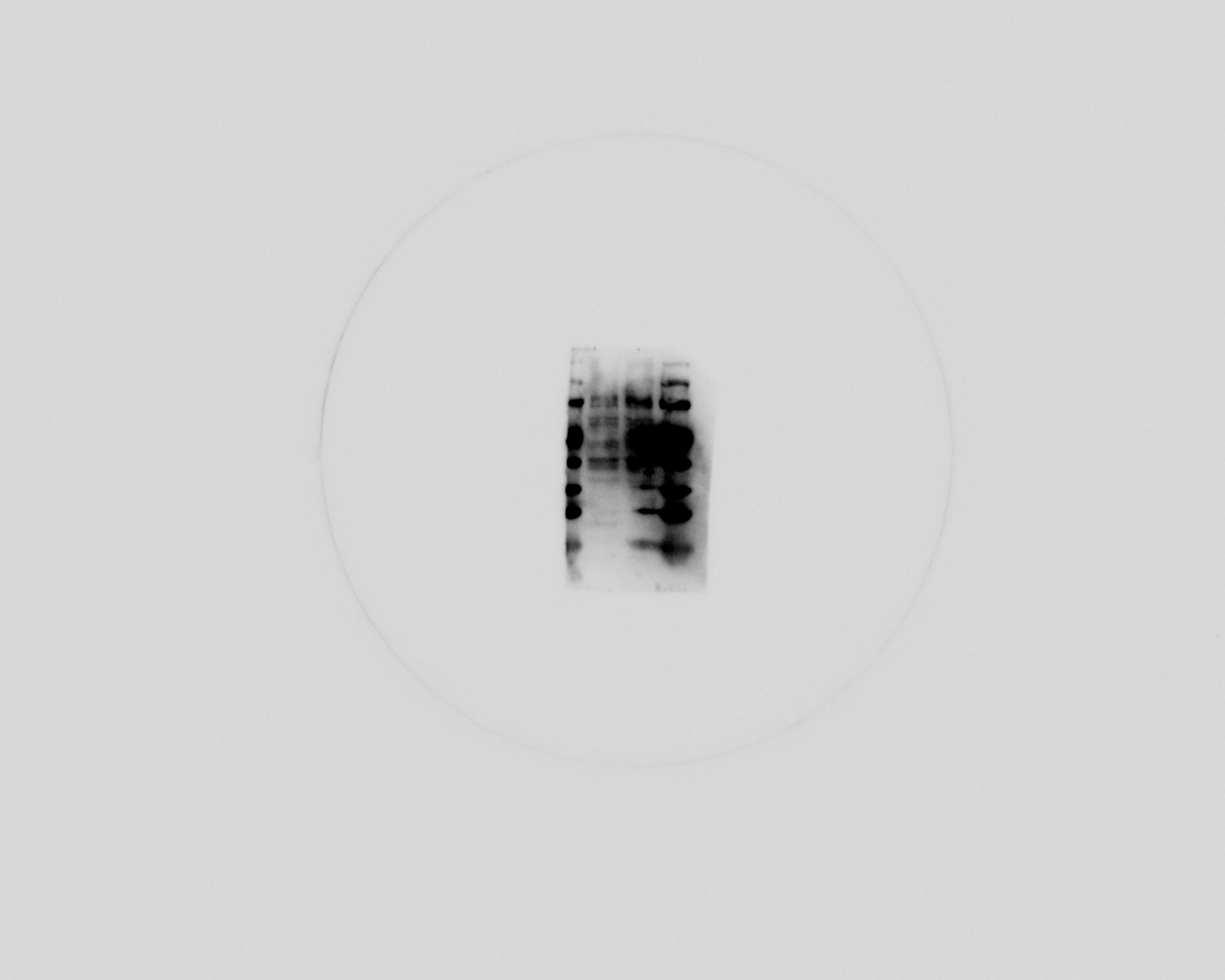

Supplement: Supplementary file 6 [file Data_Sheet_6.zip › FIG5/Aβ APP(VPC)/app/original data/wb 2022-10-22 6'4app.tif]

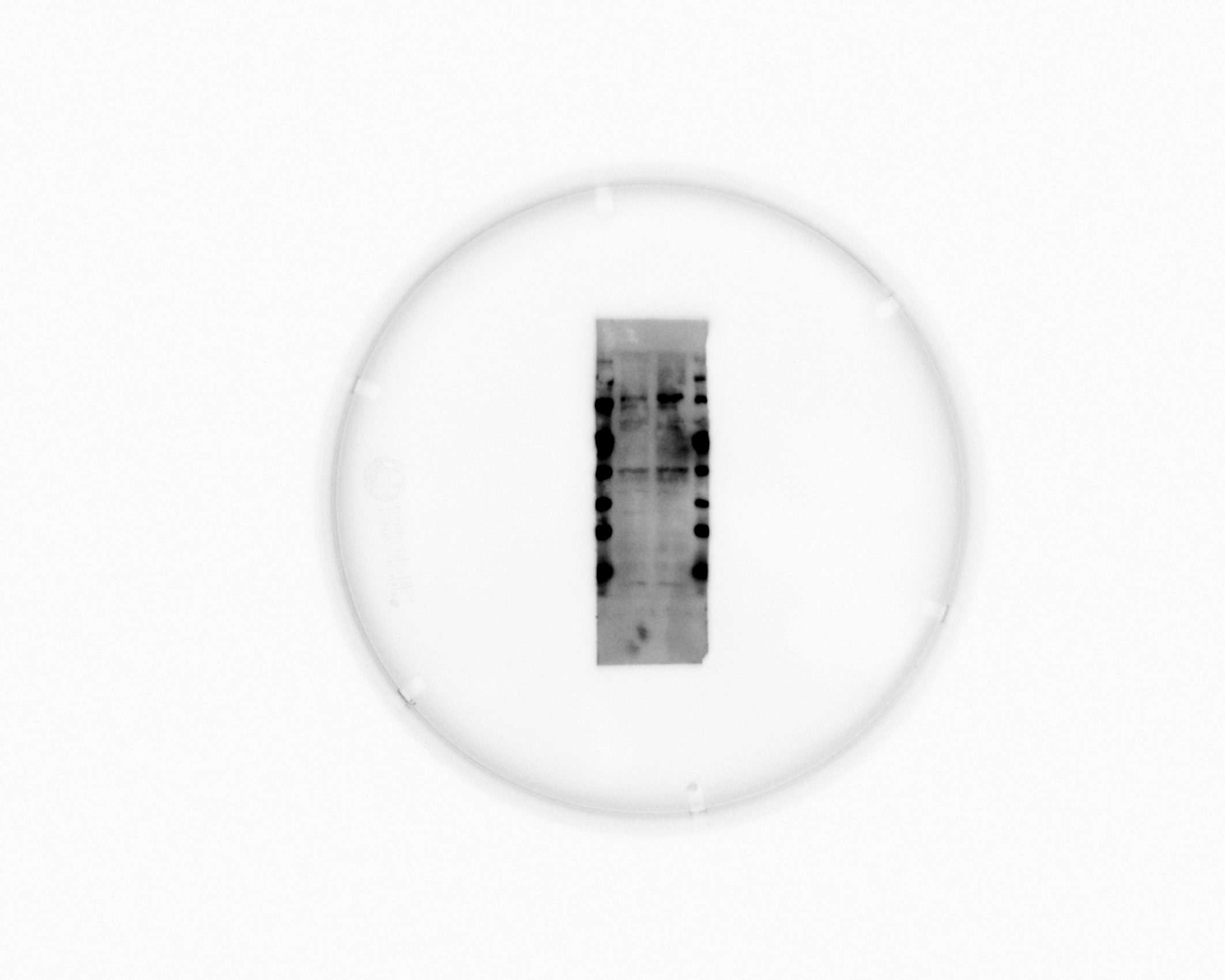

Supplement: Supplementary file 6 [file Data_Sheet_6.zip › FIG5/Aβ APP(VPC)/app/original data/wb 2022-10-22 8'2app.tif]

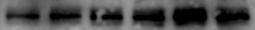

Supplement: Supplementary file 6 [file Data_Sheet_6.zip › FIG5/Aβ APP(VPC)/app/wb 2022-10-03 jxh 1app.png]

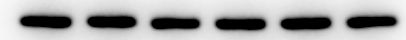

Supplement: Supplementary file 6 [file Data_Sheet_6.zip › FIG5/Aβ APP(VPC)/app/wb 2022-10-03 jxh 1tub.png]

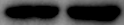

Supplement: Supplementary file 6 [file Data_Sheet_6.zip › FIG5/Aβ APP(VPC)/app/wb 2022-10-20 jxh 6'1tub.png]

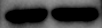

Supplement: Supplementary file 6 [file Data_Sheet_6.zip › FIG5/Aβ APP(VPC)/app/wb 2022-10-20 jxh 6'2tub.png]

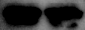

Supplement: Supplementary file 6 [file Data_Sheet_6.zip › FIG5/Aβ APP(VPC)/app/wb 2022-10-20 jxh 6'4tub.png]

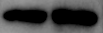

Supplement: Supplementary file 6 [file Data_Sheet_6.zip › FIG5/Aβ APP(VPC)/app/wb 2022-10-20 jxh 8'2tub.png]

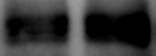

Supplement: Supplementary file 6 [file Data_Sheet_6.zip › FIG5/Aβ APP(VPC)/app/wb 2022-10-22 jxh 6'1app.png]

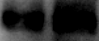

Supplement: Supplementary file 6 [file Data_Sheet_6.zip › FIG5/Aβ APP(VPC)/app/wb 2022-10-22 jxh 6'2app.png]

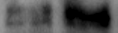

Supplement: Supplementary file 6 [file Data_Sheet_6.zip › FIG5/Aβ APP(VPC)/app/wb 2022-10-22 jxh 6'4app.png]

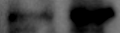

Supplement: Supplementary file 6 [file Data_Sheet_6.zip › FIG5/Aβ APP(VPC)/app/wb 2022-10-22 jxh 8'2app.png]

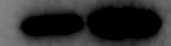

Supplement: Supplementary file 8 [file Data_Sheet_8.zip › p-p38/3.1 p-p38.png]

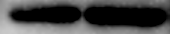

Supplement: Supplementary file 8 [file Data_Sheet_8.zip › p-p38/3.1 tub.png]

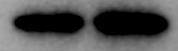

Supplement: Supplementary file 8 [file Data_Sheet_8.zip › p-p38/3.2 p-p38.png]

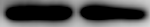

Supplement: Supplementary file 8 [file Data_Sheet_8.zip › p-p38/3.2 tub.png]
