# Supplementary figures and images for: Suppression of hnRNP A1 binding to HK1 RNA leads to glycolytic dysfunction in Alzheimer’s disease models (part 3 of 4)
Source: Front Aging Neurosci. 2023 Aug 31;15:1218267. doi: 10.3389/fnagi.2023.1218267 (PMC10516183; doi:10.3389/fnagi.2023.1218267)

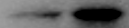

Supplement: Supplementary file 8 [file Data_Sheet_8.zip › p-p38/3.3 p-p38.png]

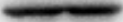

Supplement: Supplementary file 8 [file Data_Sheet_8.zip › p-p38/3.3 tub.png]

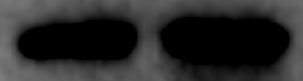

Supplement: Supplementary file 8 [file Data_Sheet_8.zip › p-p38/4.2 p-p38.png]

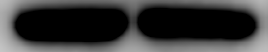

Supplement: Supplementary file 8 [file Data_Sheet_8.zip › p-p38/4.2 tub.png]

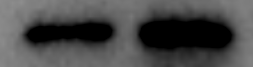

Supplement: Supplementary file 8 [file Data_Sheet_8.zip › p-p38/4.3 p-p38.png]

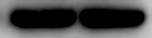

Supplement: Supplementary file 8 [file Data_Sheet_8.zip › p-p38/4.3tub.png]

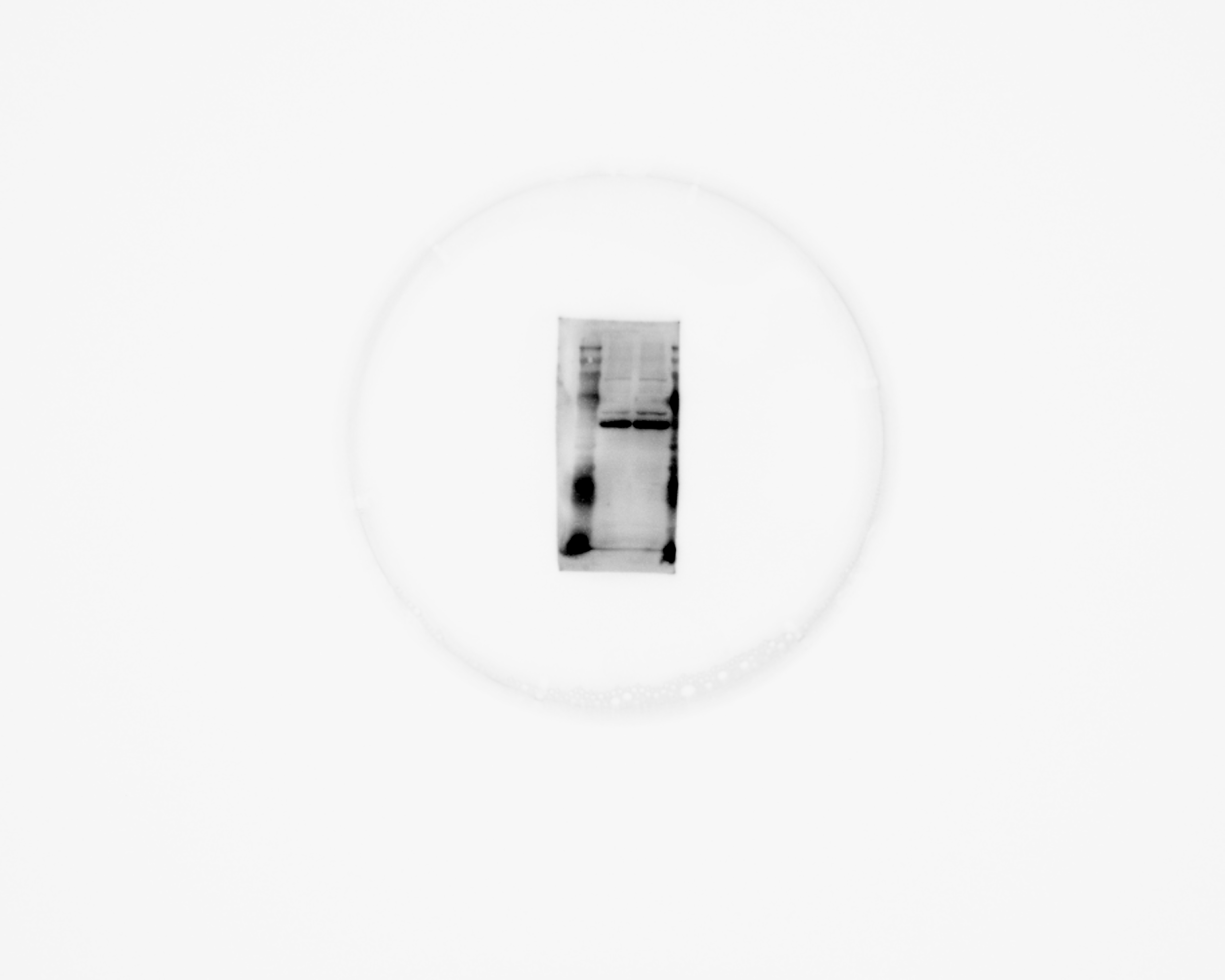

Supplement: Supplementary file 8 [file Data_Sheet_8.zip › p-p38/original data/2022-12-31 3'1tub.tif]

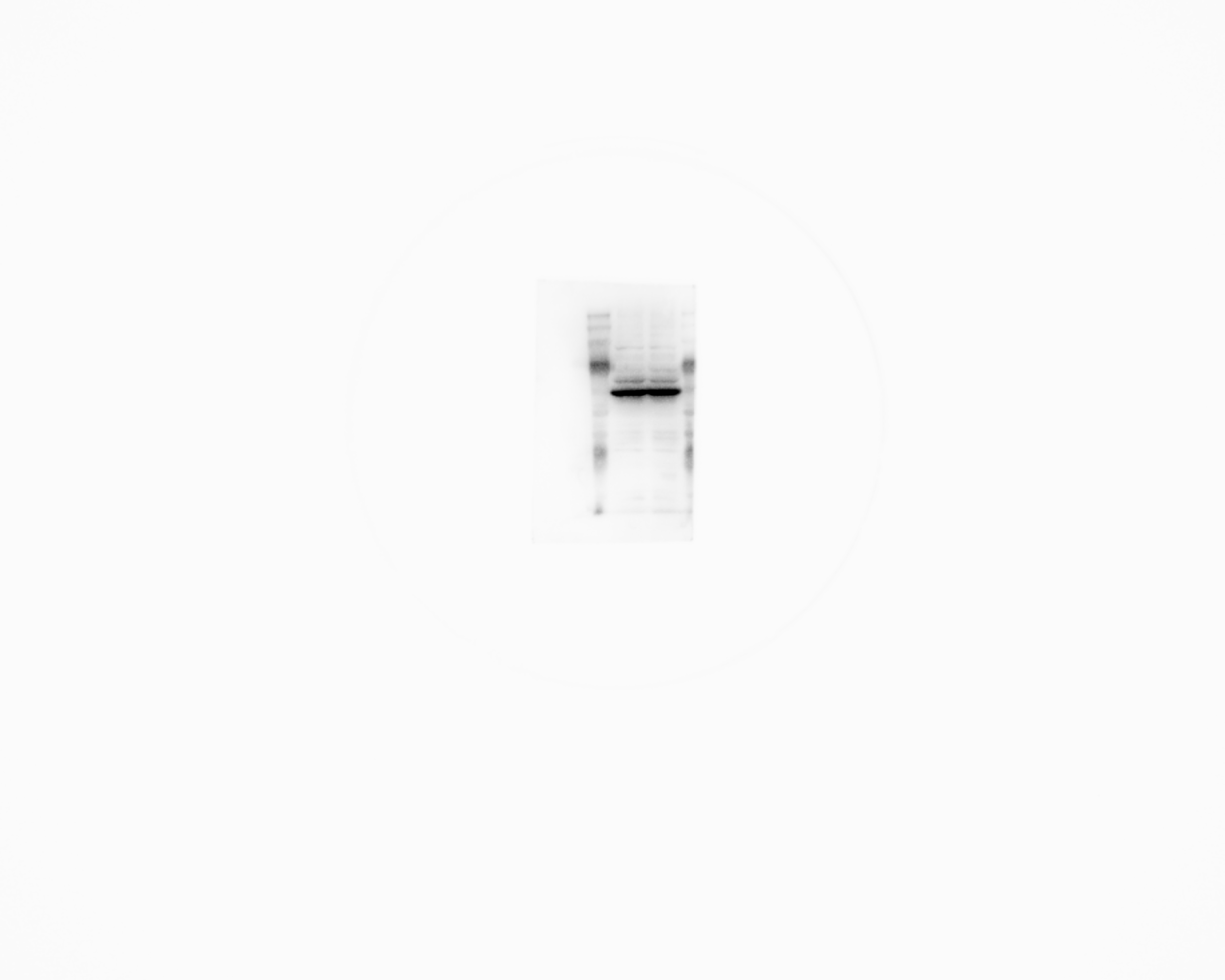

Supplement: Supplementary file 8 [file Data_Sheet_8.zip › p-p38/original data/2022-12-31 3'3tub.tif]

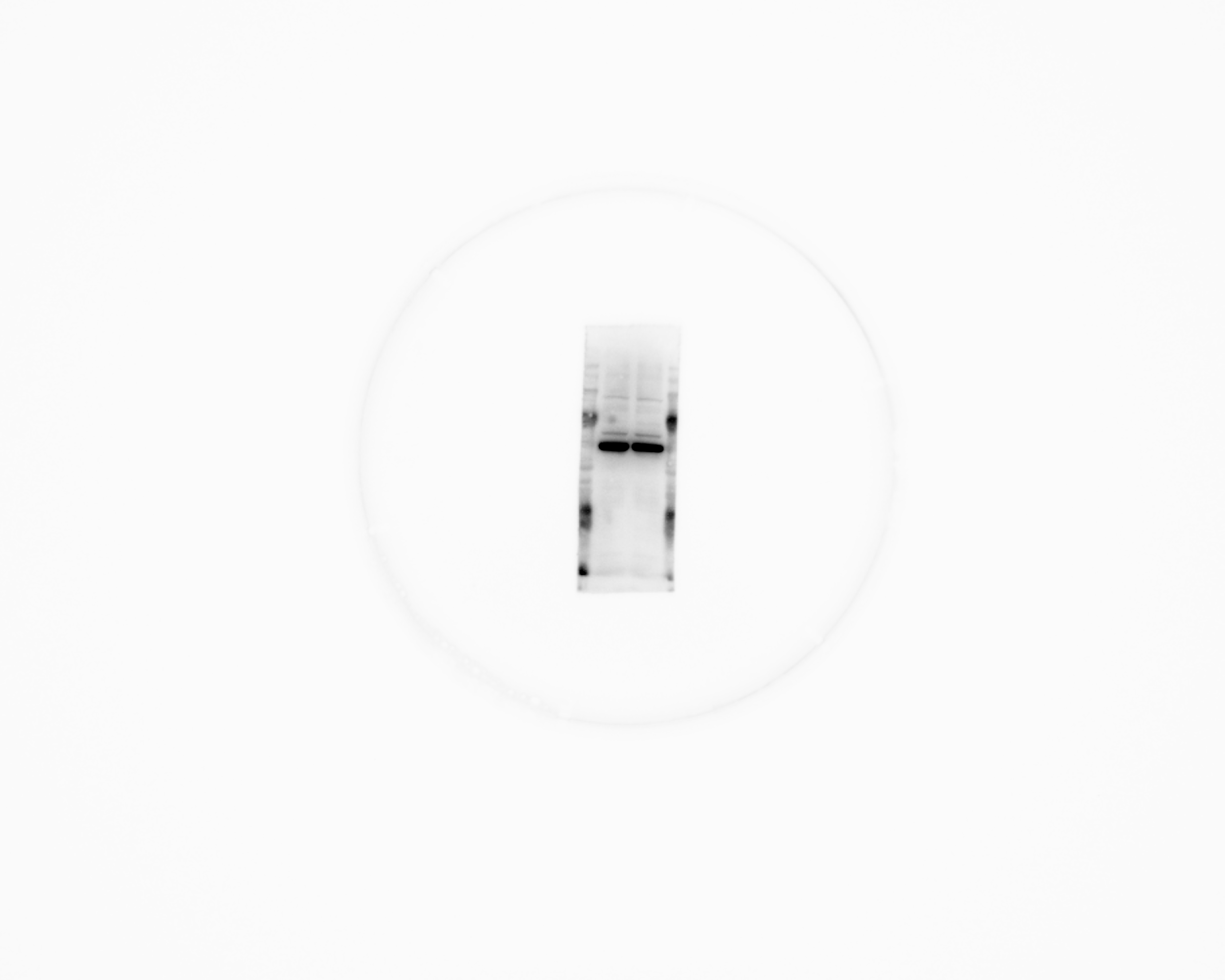

Supplement: Supplementary file 8 [file Data_Sheet_8.zip › p-p38/original data/2022-12-31 4'2 tub.tif]

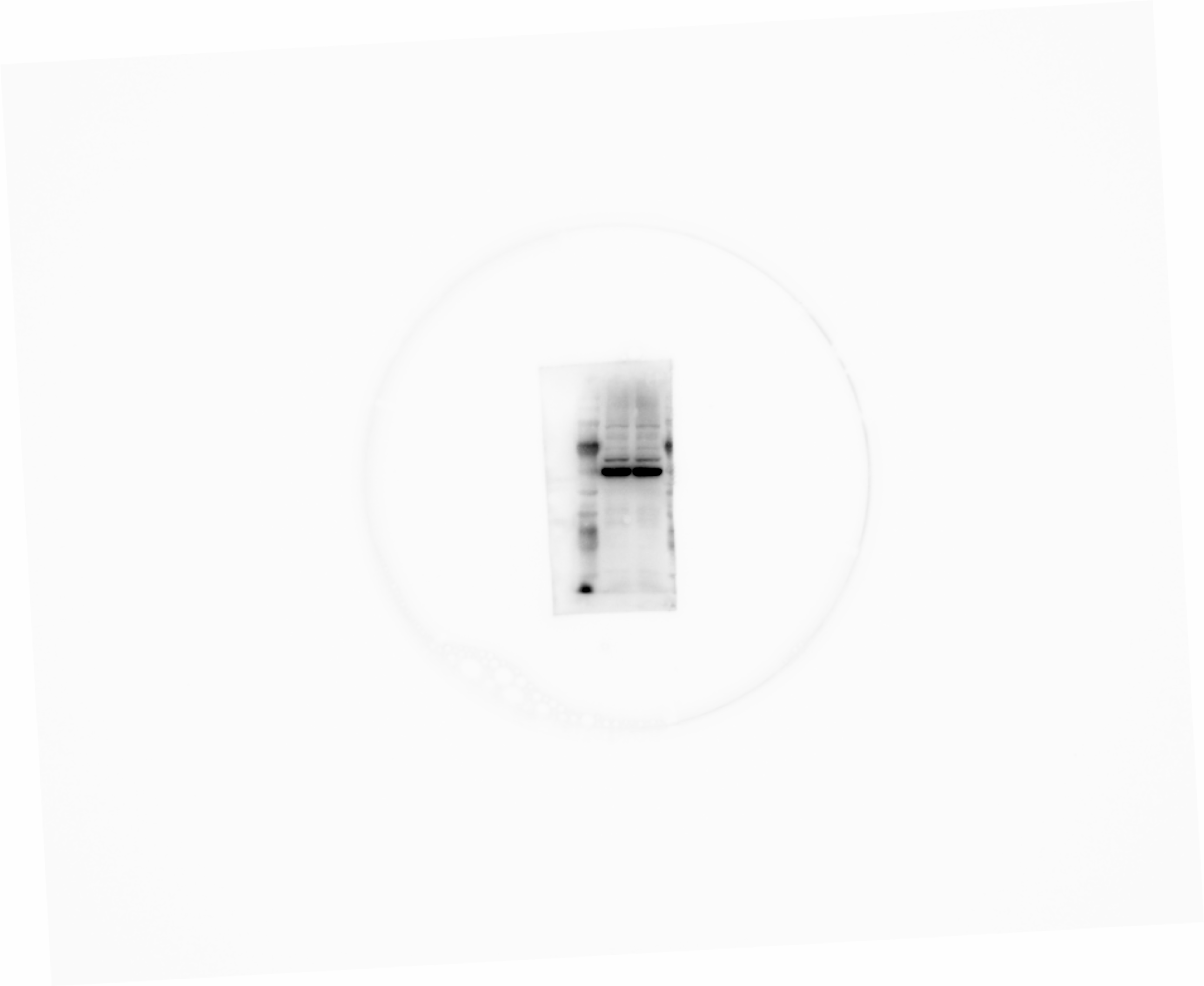

Supplement: Supplementary file 8 [file Data_Sheet_8.zip › p-p38/original data/2022-12-31 4'3tub.tif]

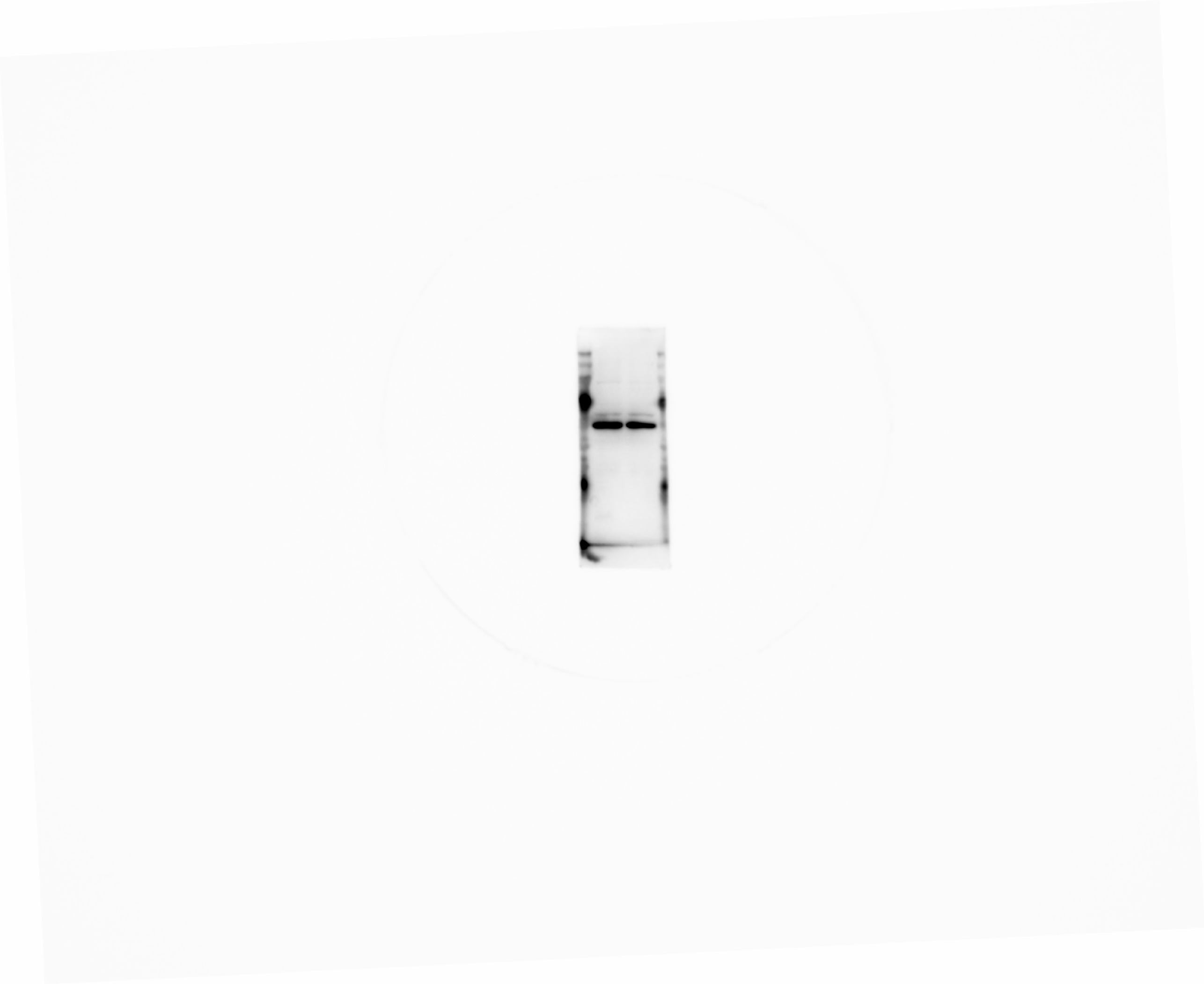

Supplement: Supplementary file 8 [file Data_Sheet_8.zip › p-p38/original data/2022-12-31 3'2tub.tif]

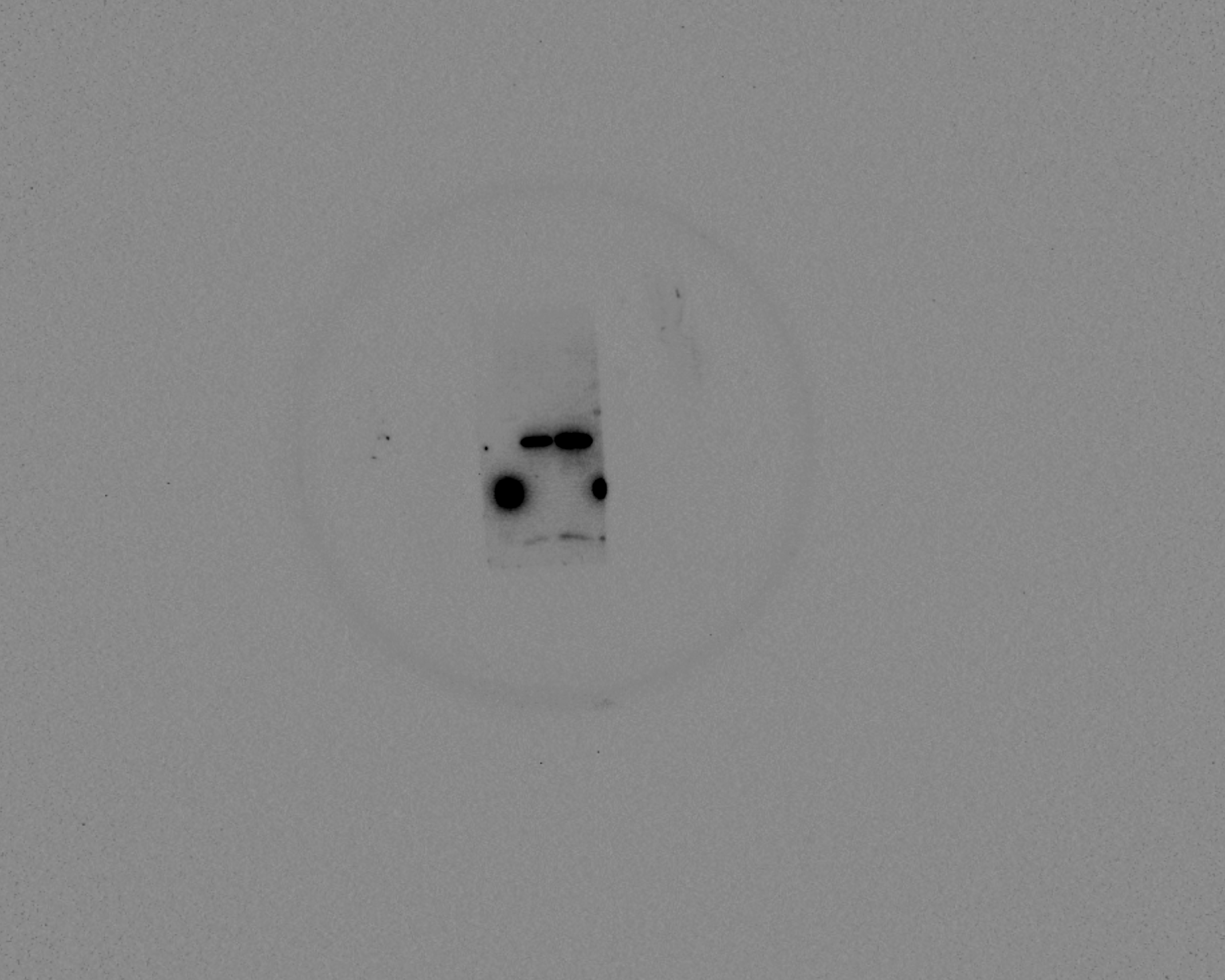

Supplement: Supplementary file 8 [file Data_Sheet_8.zip › p-p38/original data/wb 2022-12-29 3.1p38.tif]

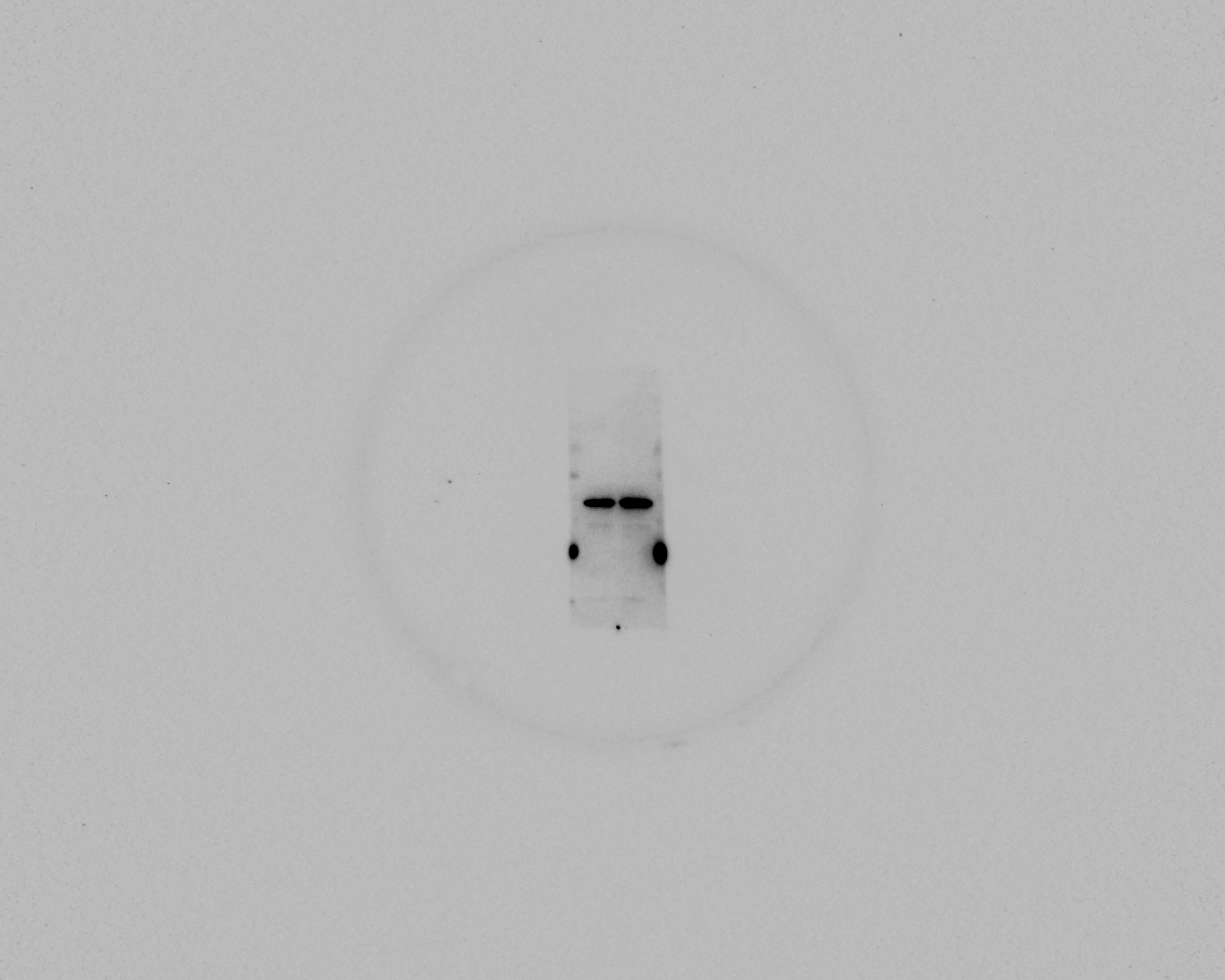

Supplement: Supplementary file 8 [file Data_Sheet_8.zip › p-p38/original data/wb 2022-12-29 3.2p38.tif]

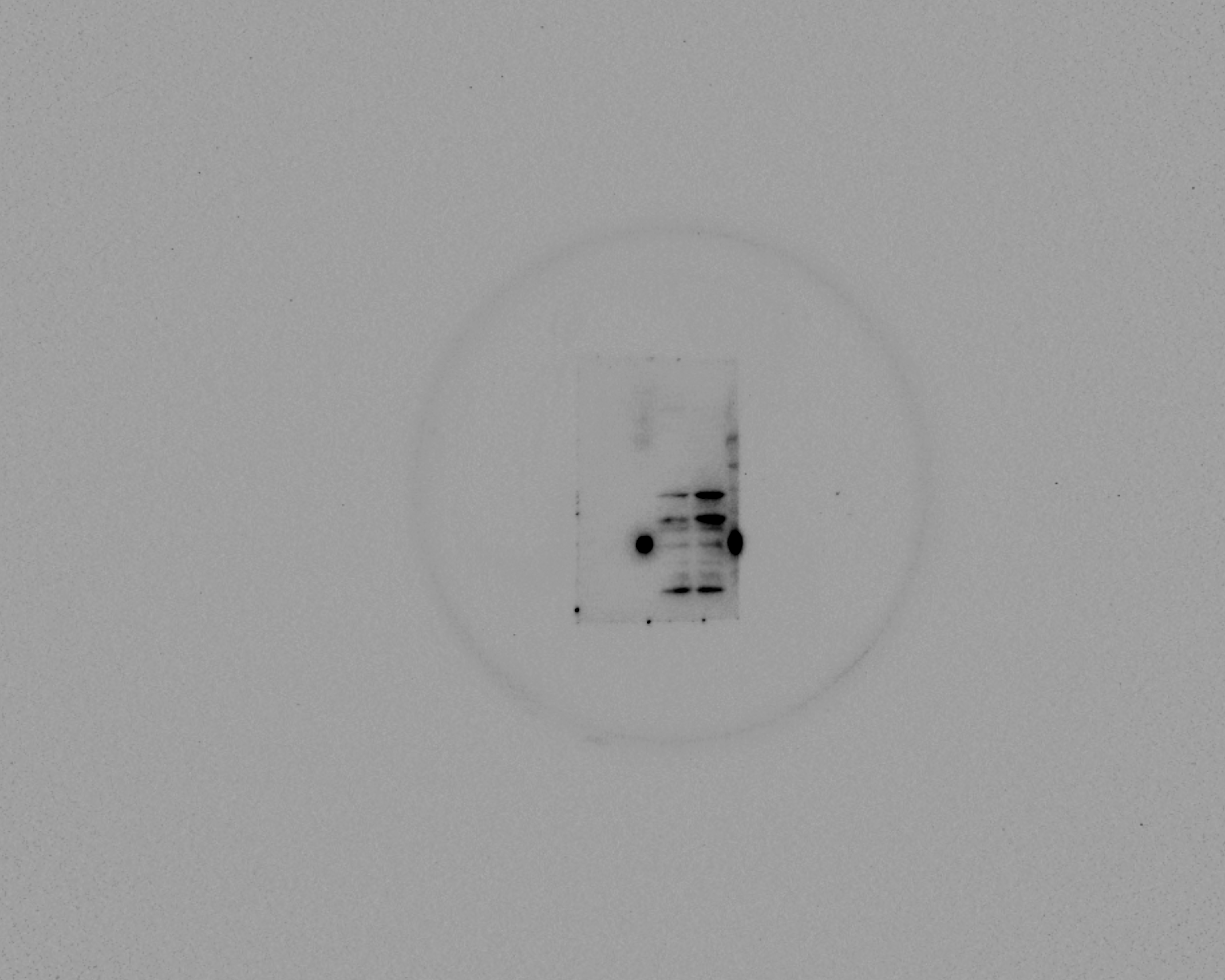

Supplement: Supplementary file 8 [file Data_Sheet_8.zip › p-p38/original data/wb 2022-12-29 3.3p38.tif]

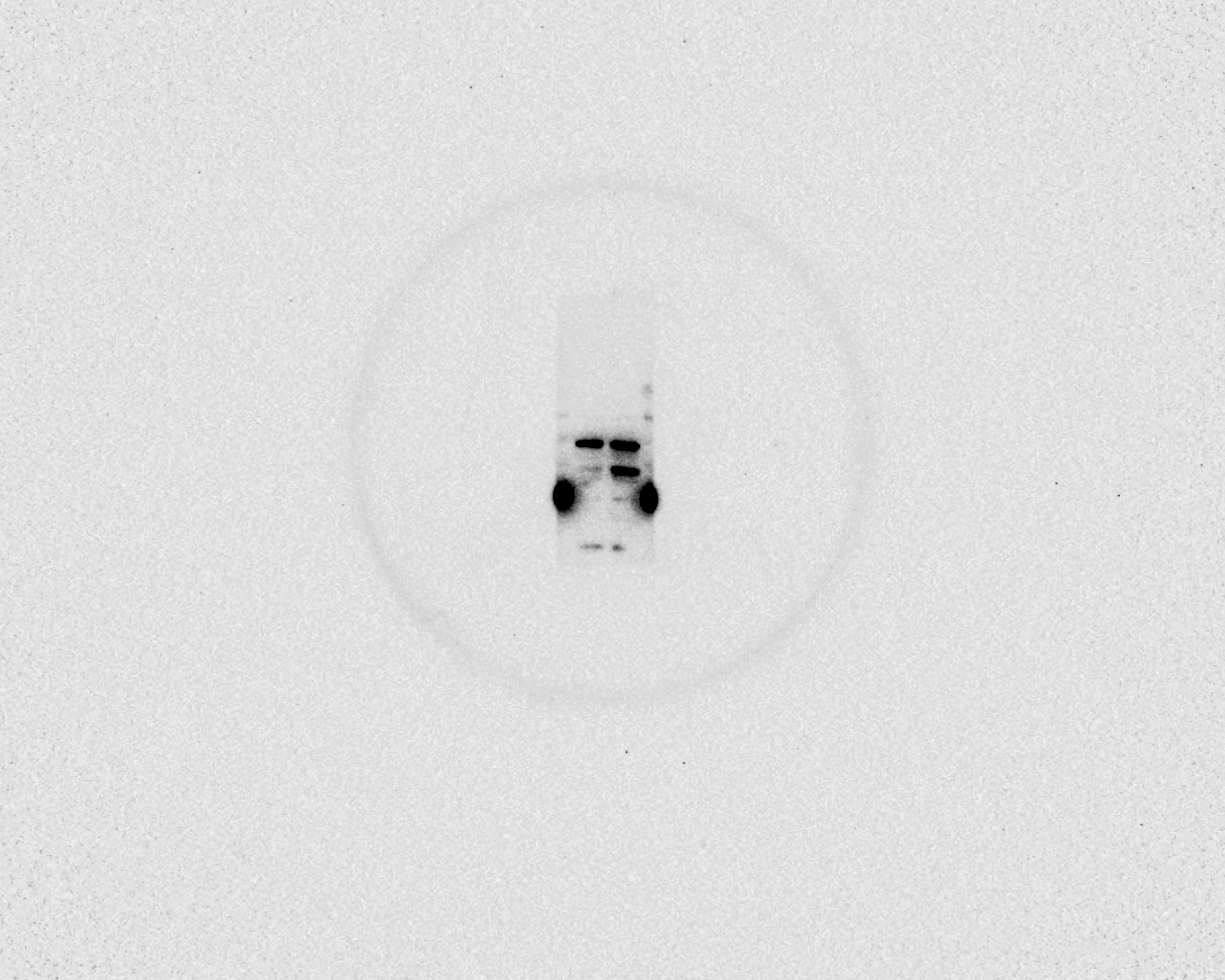

Supplement: Supplementary file 8 [file Data_Sheet_8.zip › p-p38/original data/wb 2022-12-29 4.2p38.tif]

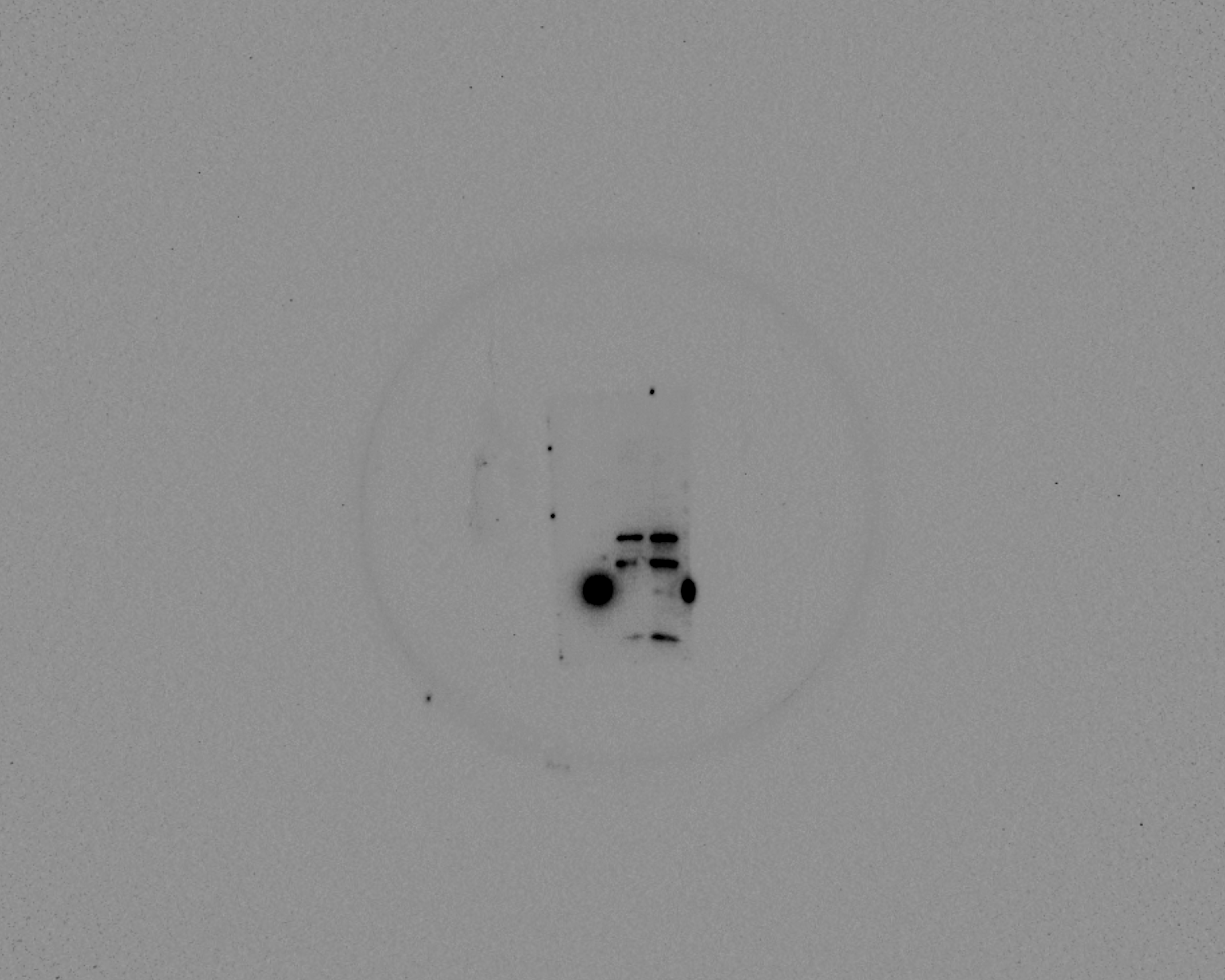

Supplement: Supplementary file 8 [file Data_Sheet_8.zip › p-p38/original data/wb 2022-12-29 4.3p38.tif]

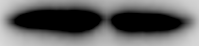

Supplement: Supplementary file 9 [file Data_Sheet_9.zip › P38/3.3 p38.png]

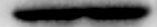

Supplement: Supplementary file 9 [file Data_Sheet_9.zip › P38/3.3 tub.png]

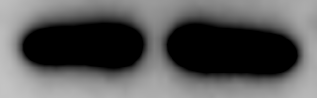

Supplement: Supplementary file 9 [file Data_Sheet_9.zip › P38/4.2 p38.png]

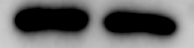

Supplement: Supplementary file 9 [file Data_Sheet_9.zip › P38/4.3 p38.png]

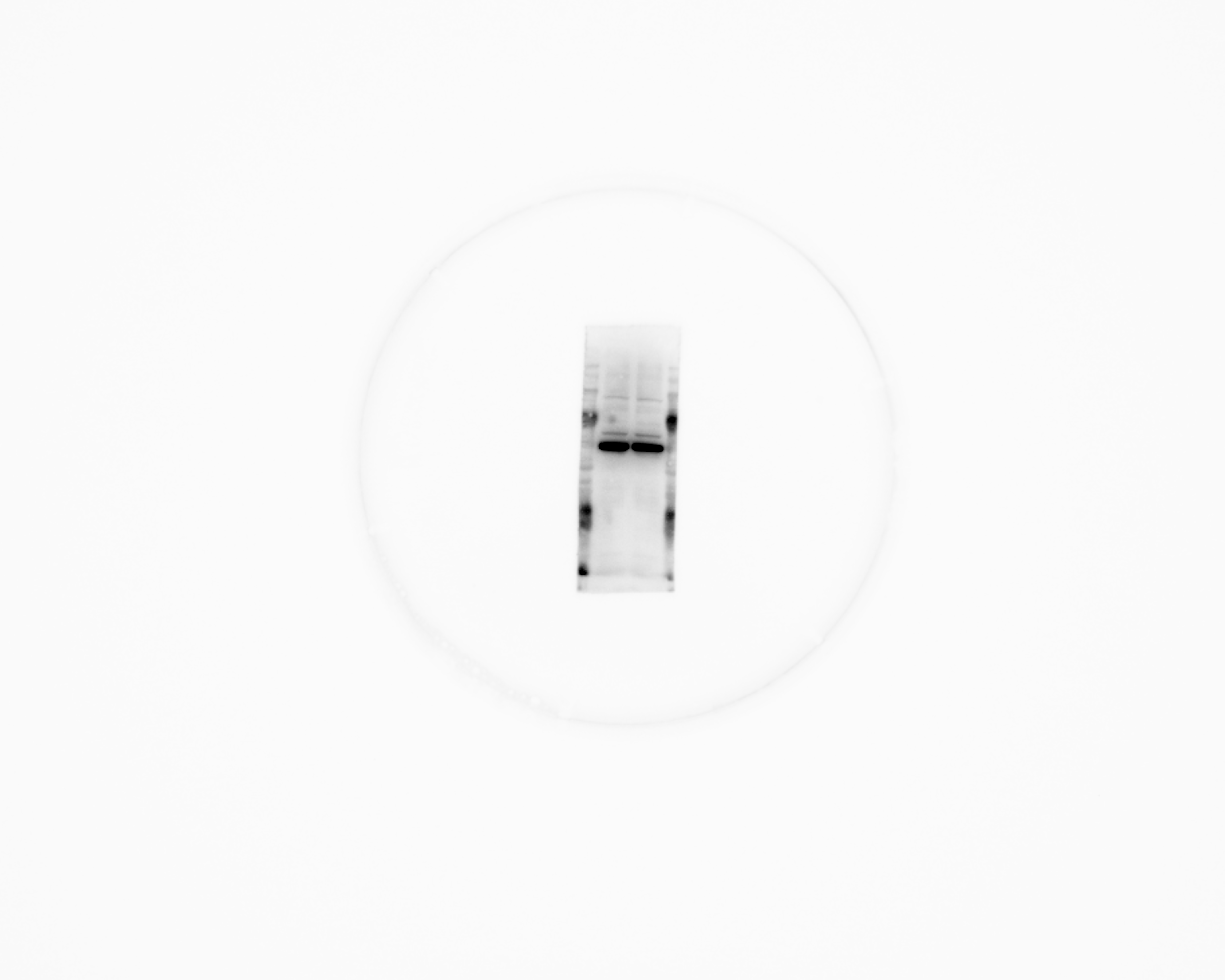

Supplement: Supplementary file 9 [file Data_Sheet_9.zip › P38/original data/2022-12-31 4'2 tub.tif]

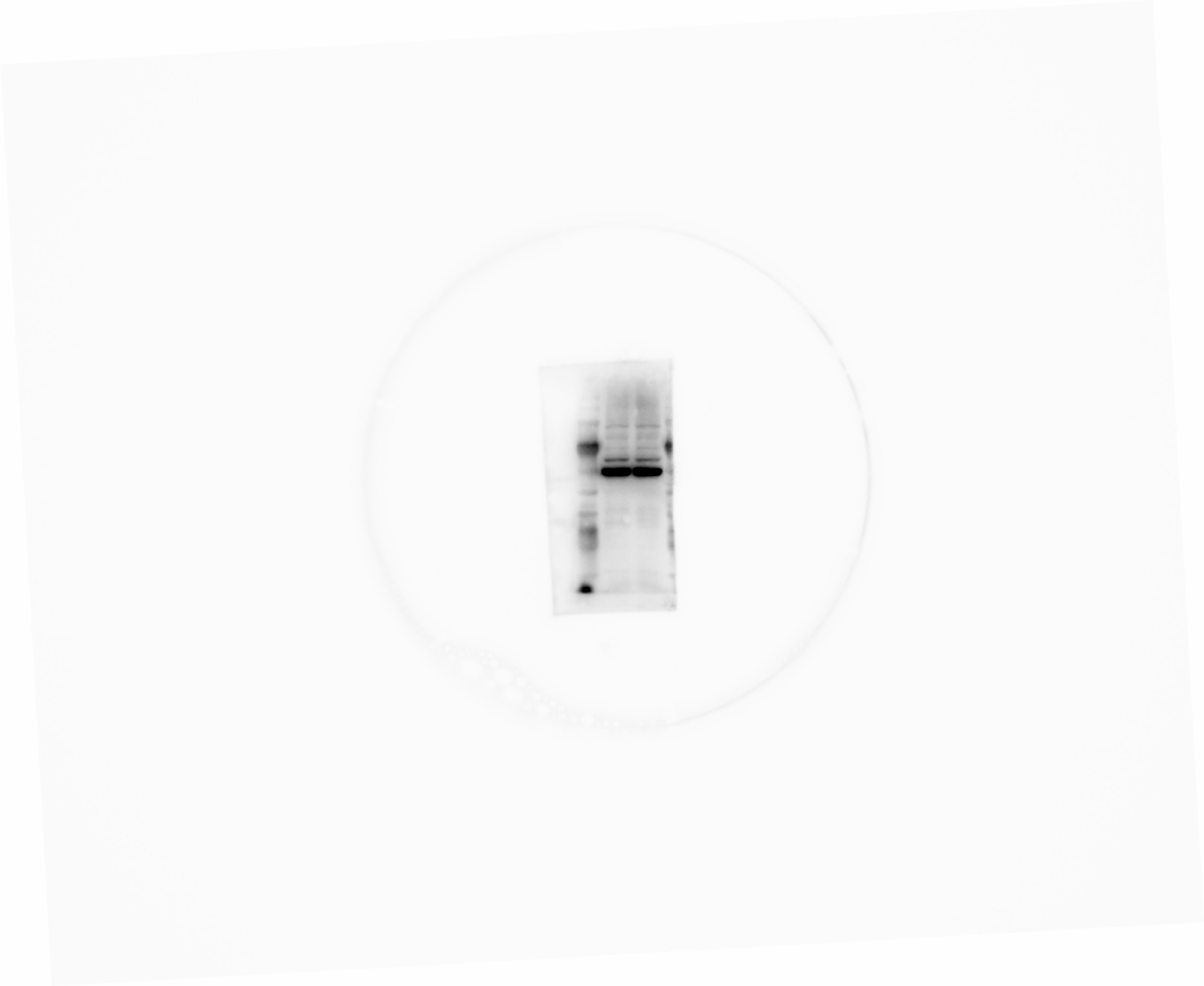

Supplement: Supplementary file 9 [file Data_Sheet_9.zip › P38/original data/2022-12-31 4'3tub.tif]

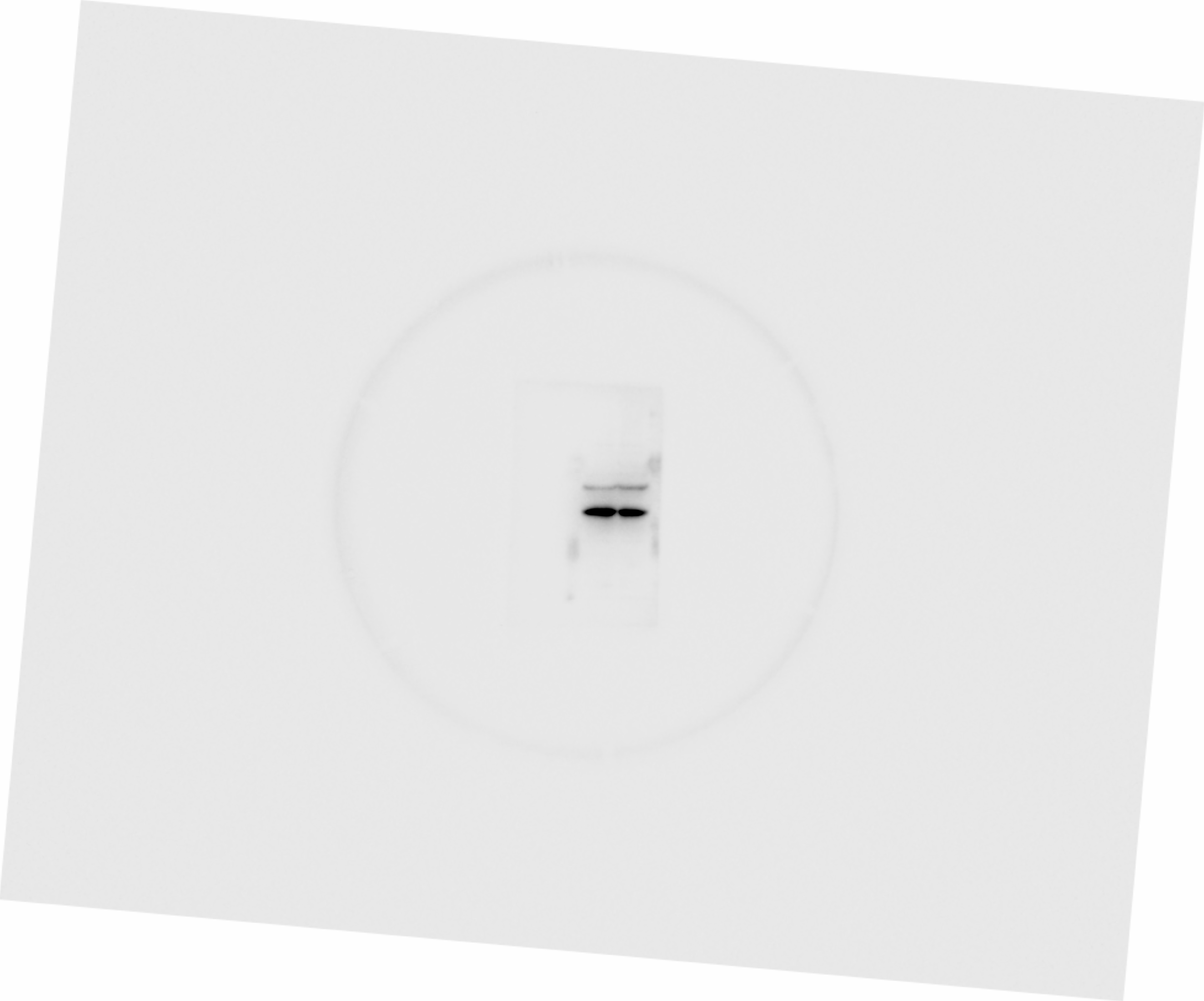

Supplement: Supplementary file 9 [file Data_Sheet_9.zip › P38/original data/wb 2023-01-01 12.29 3.3p38.tif]

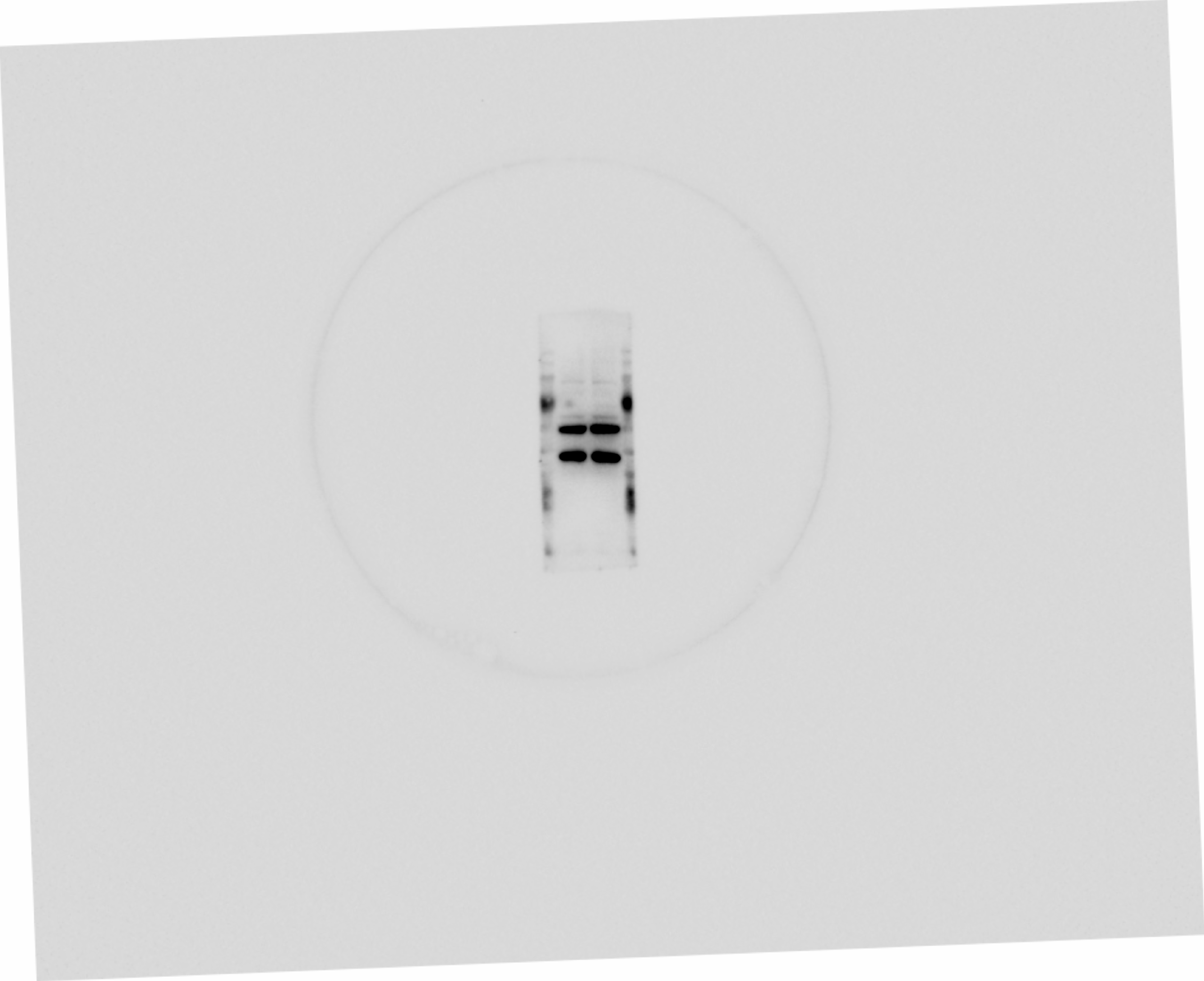

Supplement: Supplementary file 9 [file Data_Sheet_9.zip › P38/original data/wb 2023-01-01 12.29 4.2p38.tif]

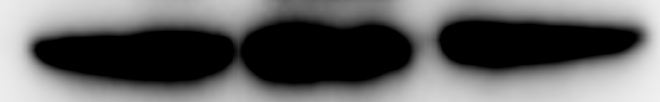

Supplement: Supplementary file 9 [file Data_Sheet_9.zip › P38 mapk-in-1 (hnrnp A1)/1.1TUB.png]

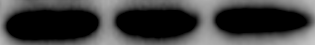

Supplement: Supplementary file 9 [file Data_Sheet_9.zip › P38 mapk-in-1 (hnrnp A1)/1.1a.png]

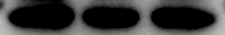

Supplement: Supplementary file 9 [file Data_Sheet_9.zip › P38 mapk-in-1 (hnrnp A1)/1.2a.png]

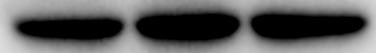

Supplement: Supplementary file 9 [file Data_Sheet_9.zip › P38 mapk-in-1 (hnrnp A1)/1.2tub.png]

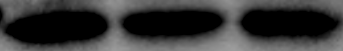

Supplement: Supplementary file 9 [file Data_Sheet_9.zip › P38 mapk-in-1 (hnrnp A1)/2.3a.png]

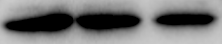

Supplement: Supplementary file 9 [file Data_Sheet_9.zip › P38 mapk-in-1 (hnrnp A1)/2.3tub.png]

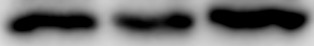

Supplement: Supplementary file 9 [file Data_Sheet_9.zip › P38 mapk-in-1 (hnrnp A1)/4.1a.png]

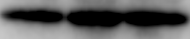

Supplement: Supplementary file 9 [file Data_Sheet_9.zip › P38 mapk-in-1 (hnrnp A1)/4.1tub.png]

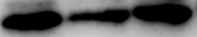

Supplement: Supplementary file 9 [file Data_Sheet_9.zip › P38 mapk-in-1 (hnrnp A1)/4.2a.png]

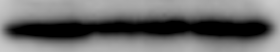

Supplement: Supplementary file 9 [file Data_Sheet_9.zip › P38 mapk-in-1 (hnrnp A1)/4.2tub.png]

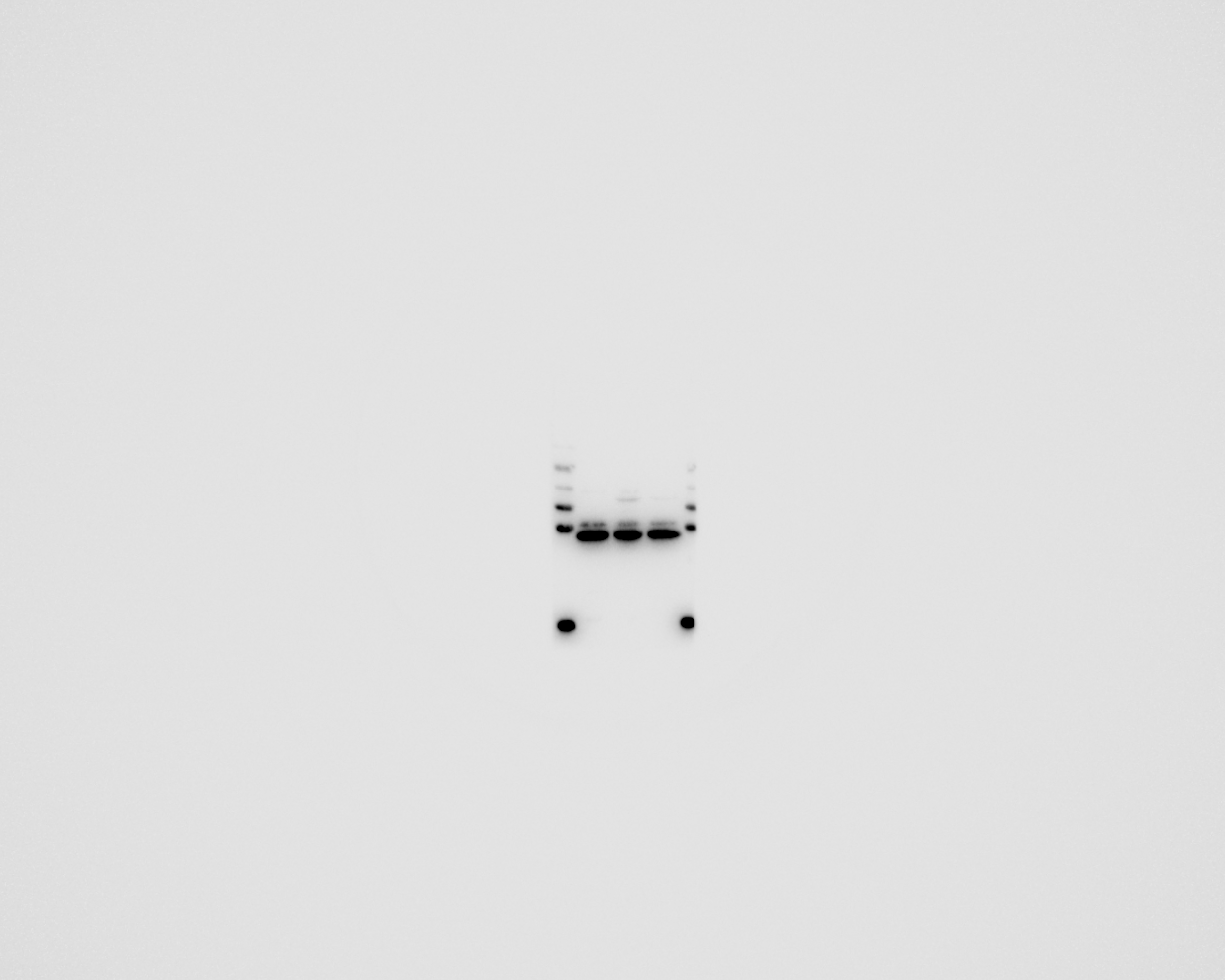

Supplement: Supplementary file 9 [file Data_Sheet_9.zip › P38 mapk-in-1 (hnrnp A1)/original data/wb 2022-12-31 1.1a.tif]

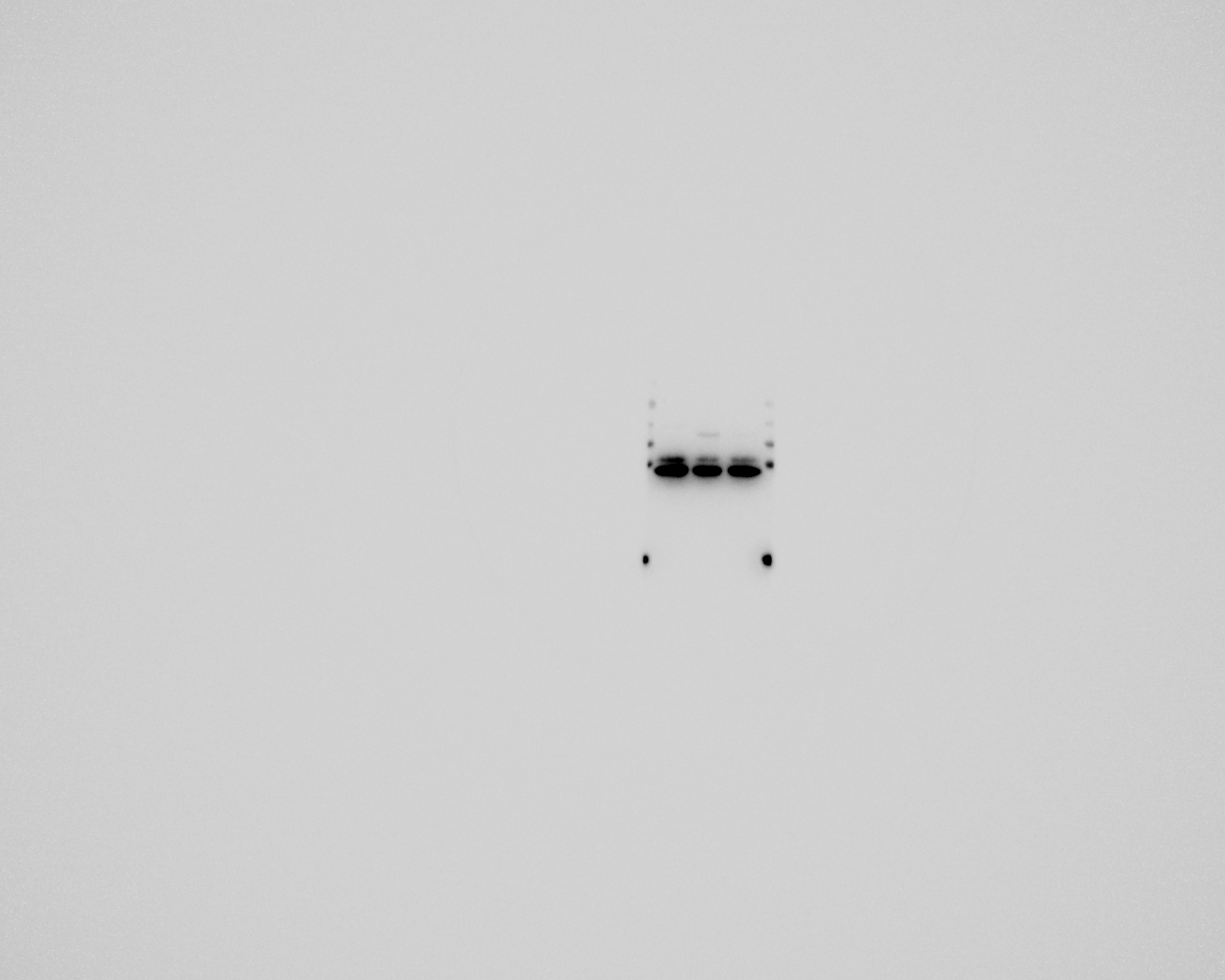

Supplement: Supplementary file 9 [file Data_Sheet_9.zip › P38 mapk-in-1 (hnrnp A1)/original data/wb 2022-12-31 1.2a.tif]

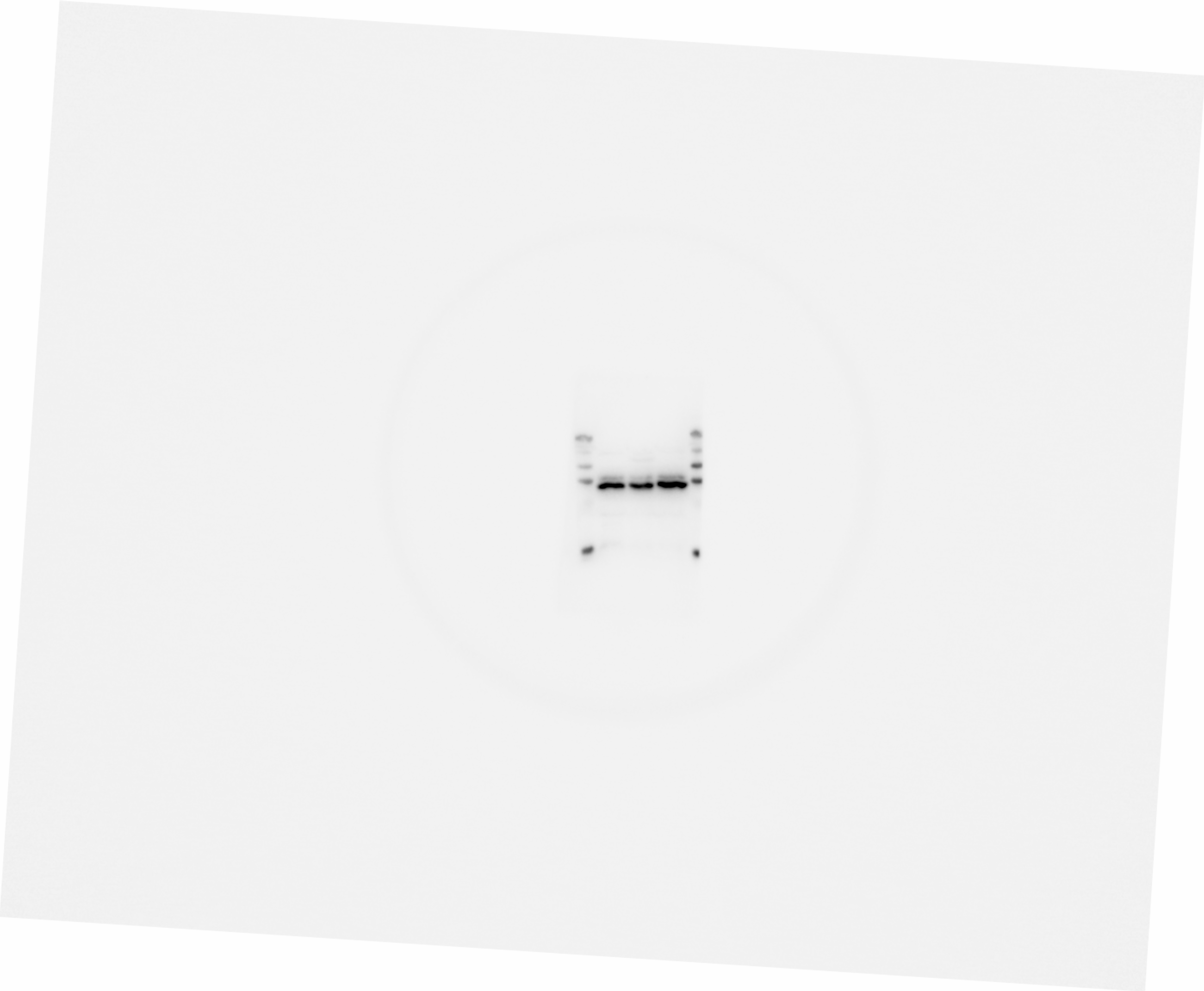

Supplement: Supplementary file 9 [file Data_Sheet_9.zip › P38 mapk-in-1 (hnrnp A1)/original data/wb 2022-12-31 4.1a.tif]

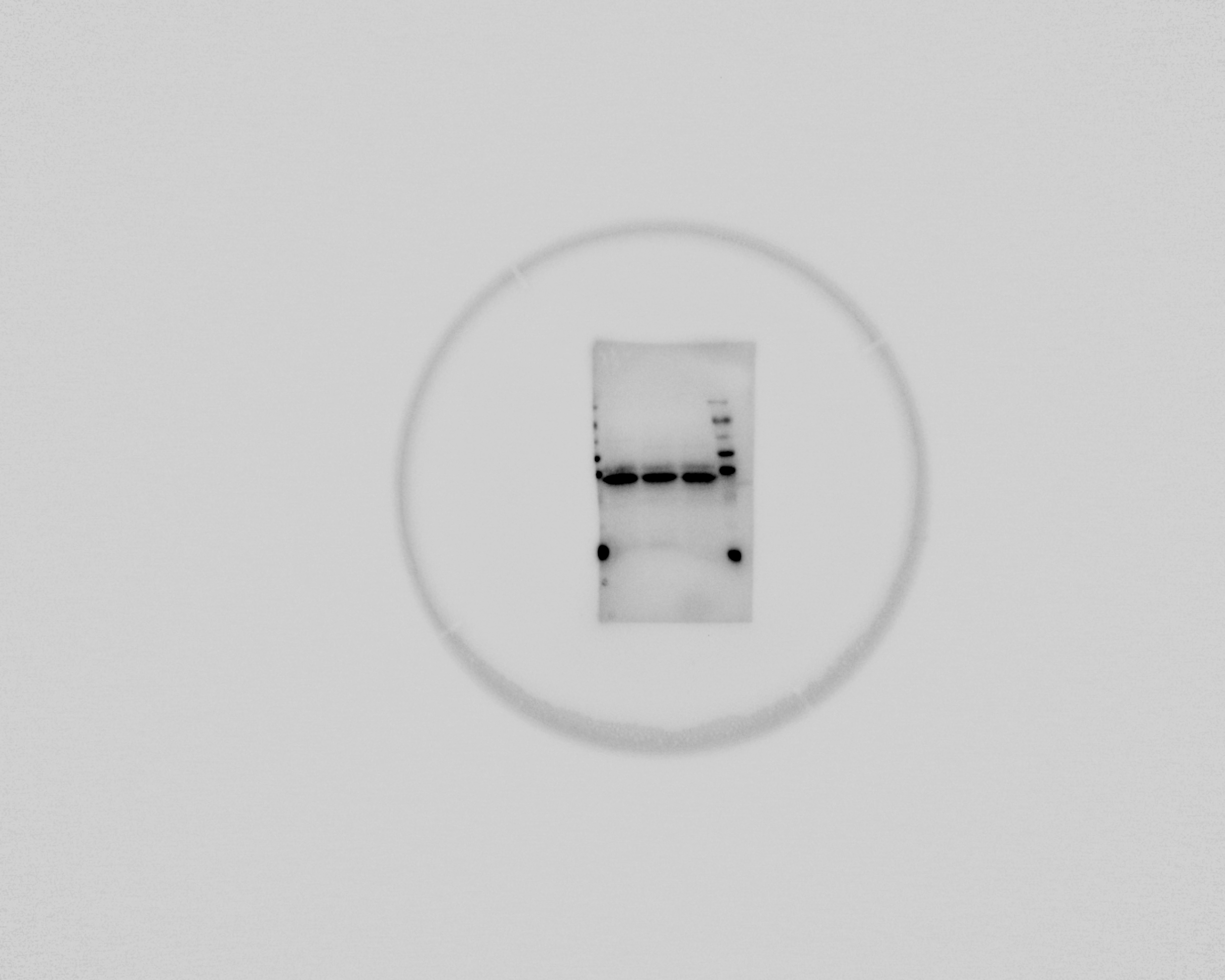

Supplement: Supplementary file 9 [file Data_Sheet_9.zip › P38 mapk-in-1 (hnrnp A1)/original data/wb 2022-12-31 2.3a.tif]

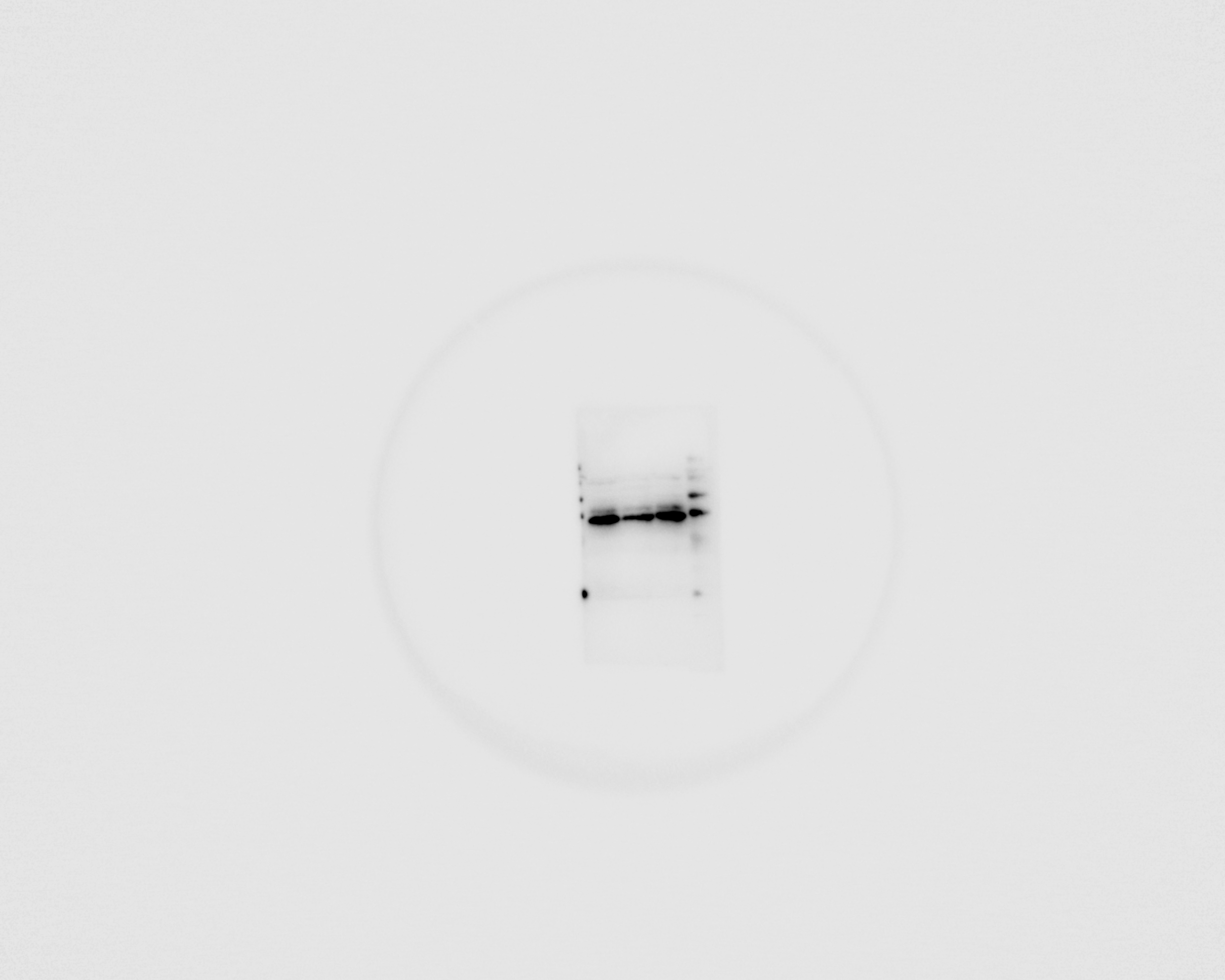

Supplement: Supplementary file 9 [file Data_Sheet_9.zip › P38 mapk-in-1 (hnrnp A1)/original data/wb 2022-12-31 4.2a.tif]

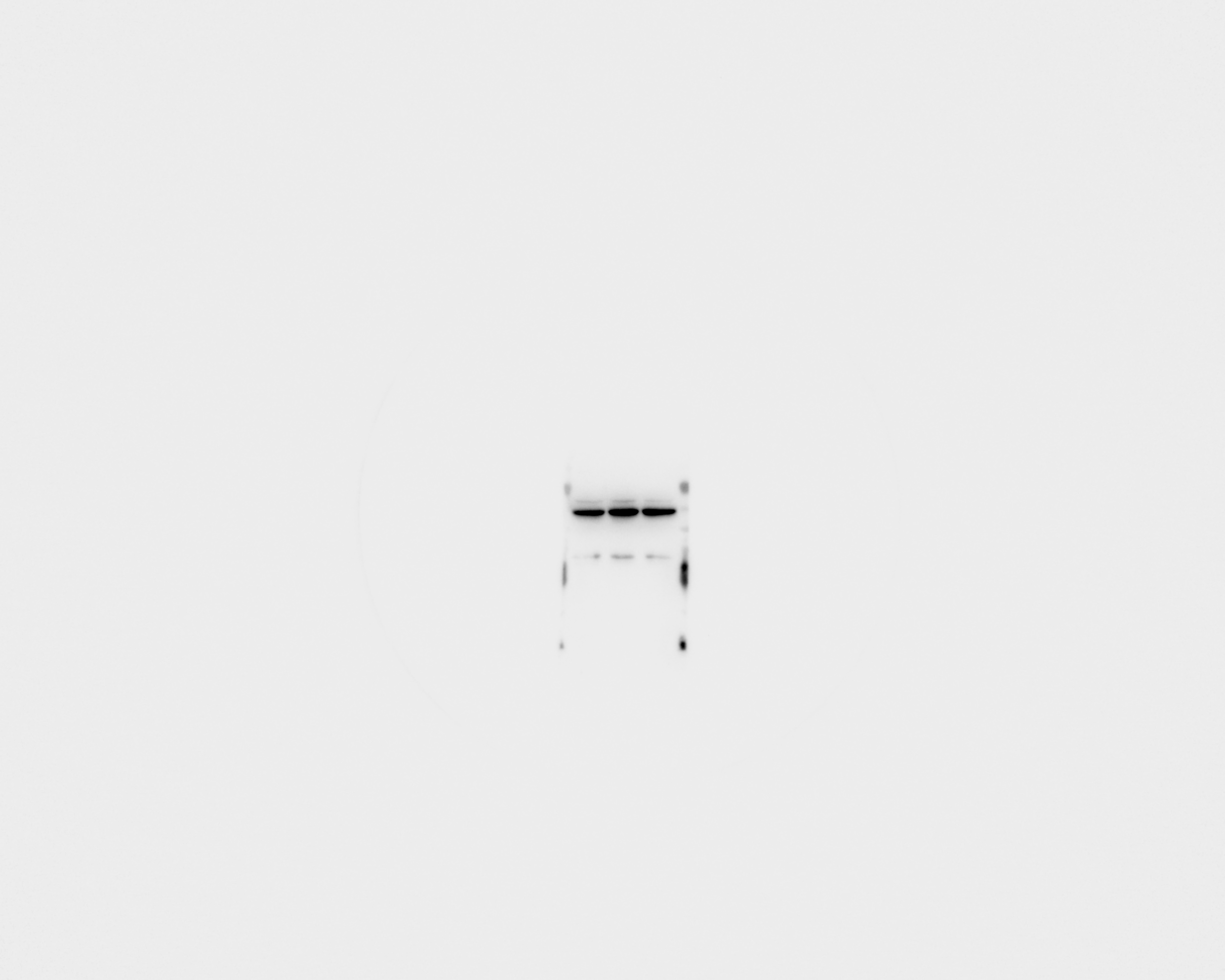

Supplement: Supplementary file 9 [file Data_Sheet_9.zip › P38 mapk-in-1 (hnrnp A1)/original data/wb 2023-01-01 1.2tub.tif]

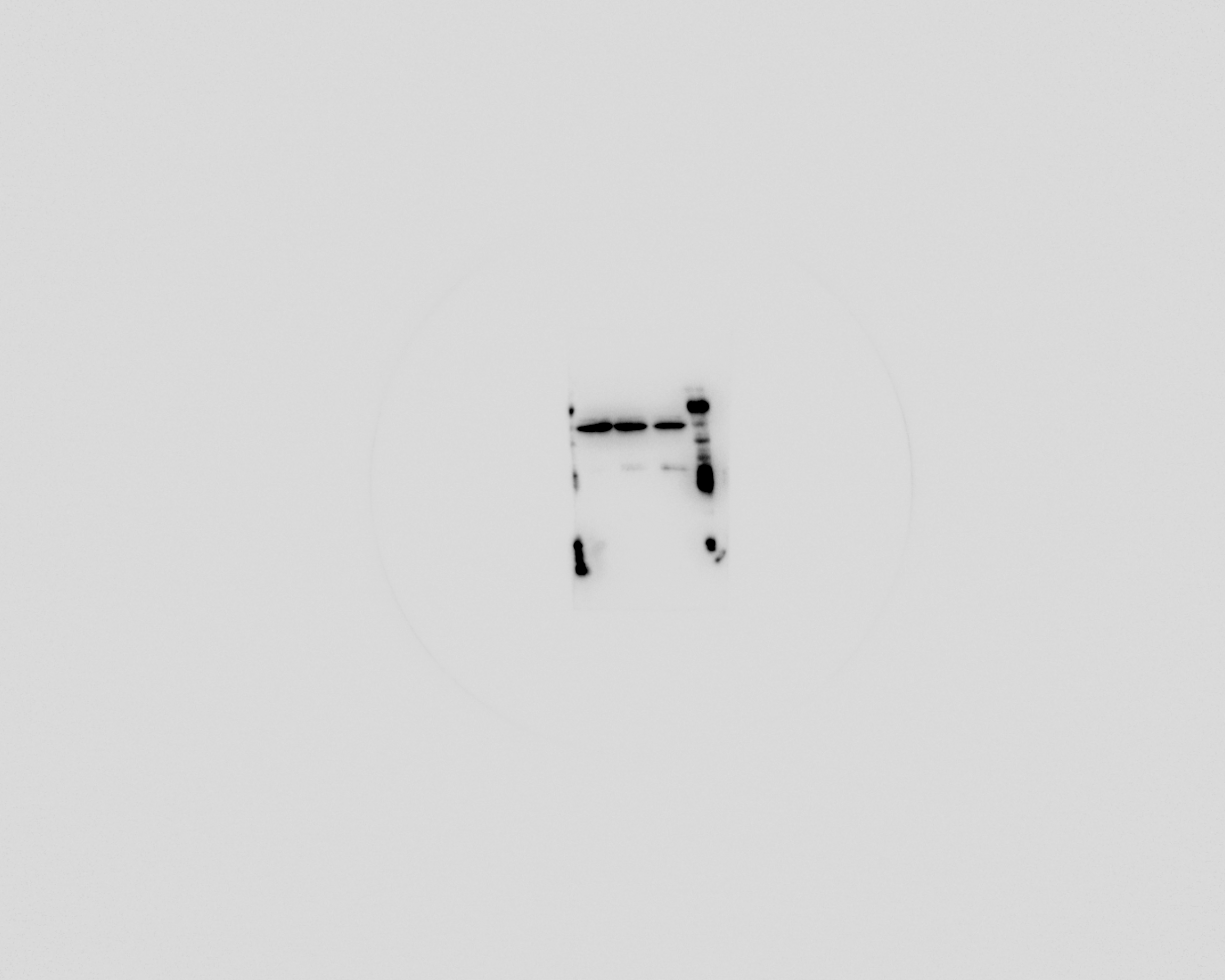

Supplement: Supplementary file 9 [file Data_Sheet_9.zip › P38 mapk-in-1 (hnrnp A1)/original data/wb 2023-01-01 2.3tub.tif]

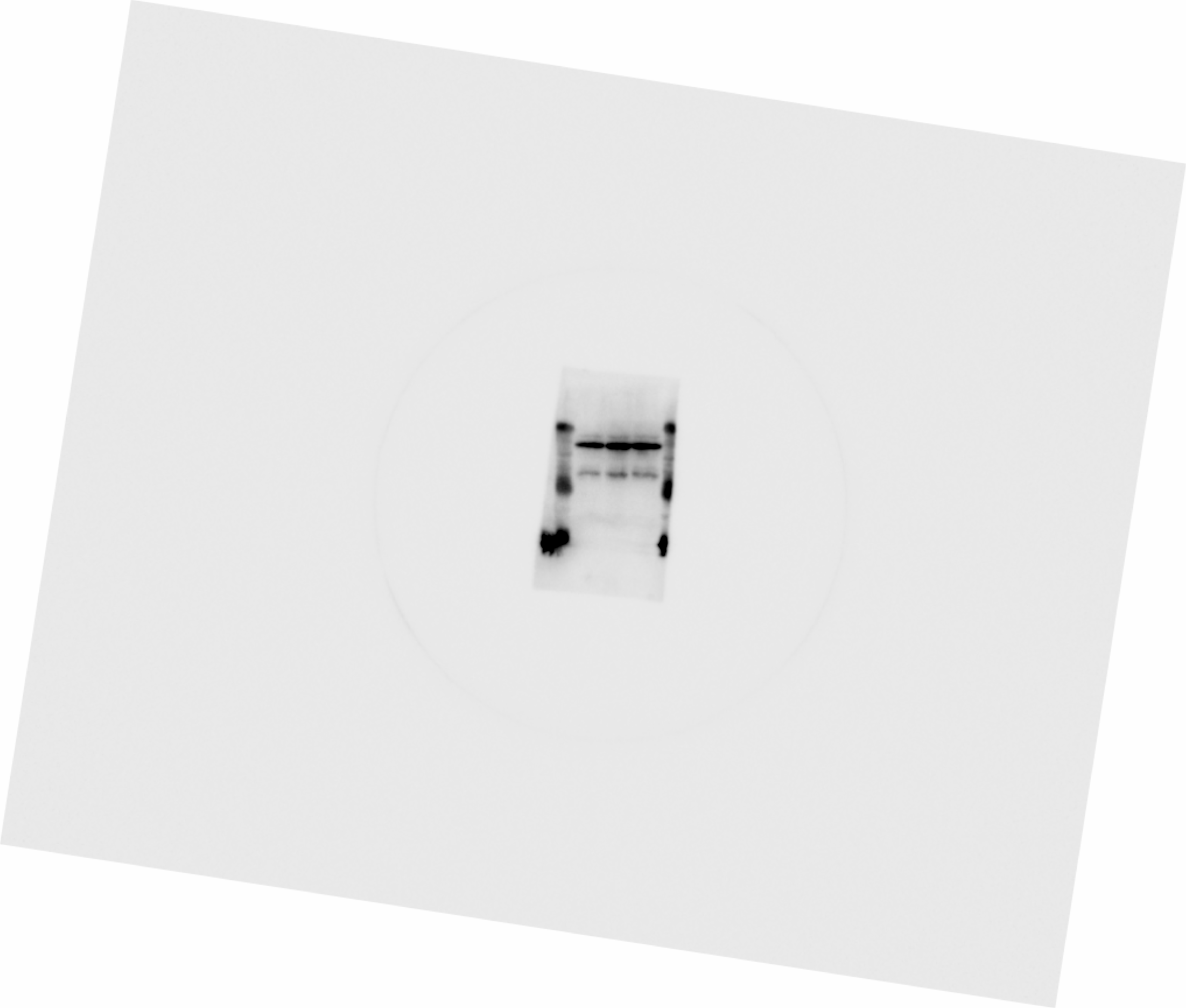

Supplement: Supplementary file 9 [file Data_Sheet_9.zip › P38 mapk-in-1 (hnrnp A1)/original data/wb 2023-01-01 4.1tub.tif]

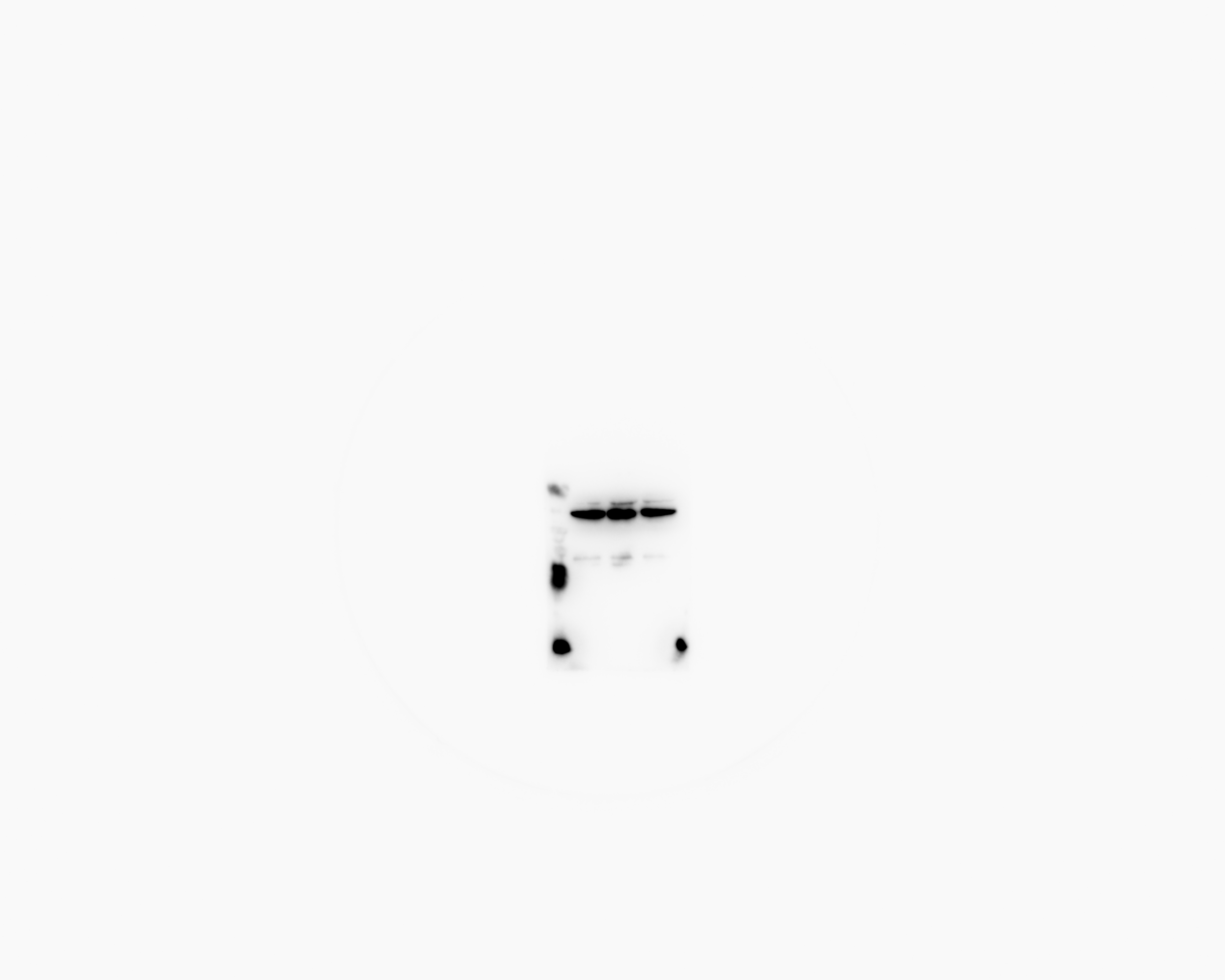

Supplement: Supplementary file 9 [file Data_Sheet_9.zip › P38 mapk-in-1 (hnrnp A1)/original data/wb 2023-01-01 tub1.1.tif]

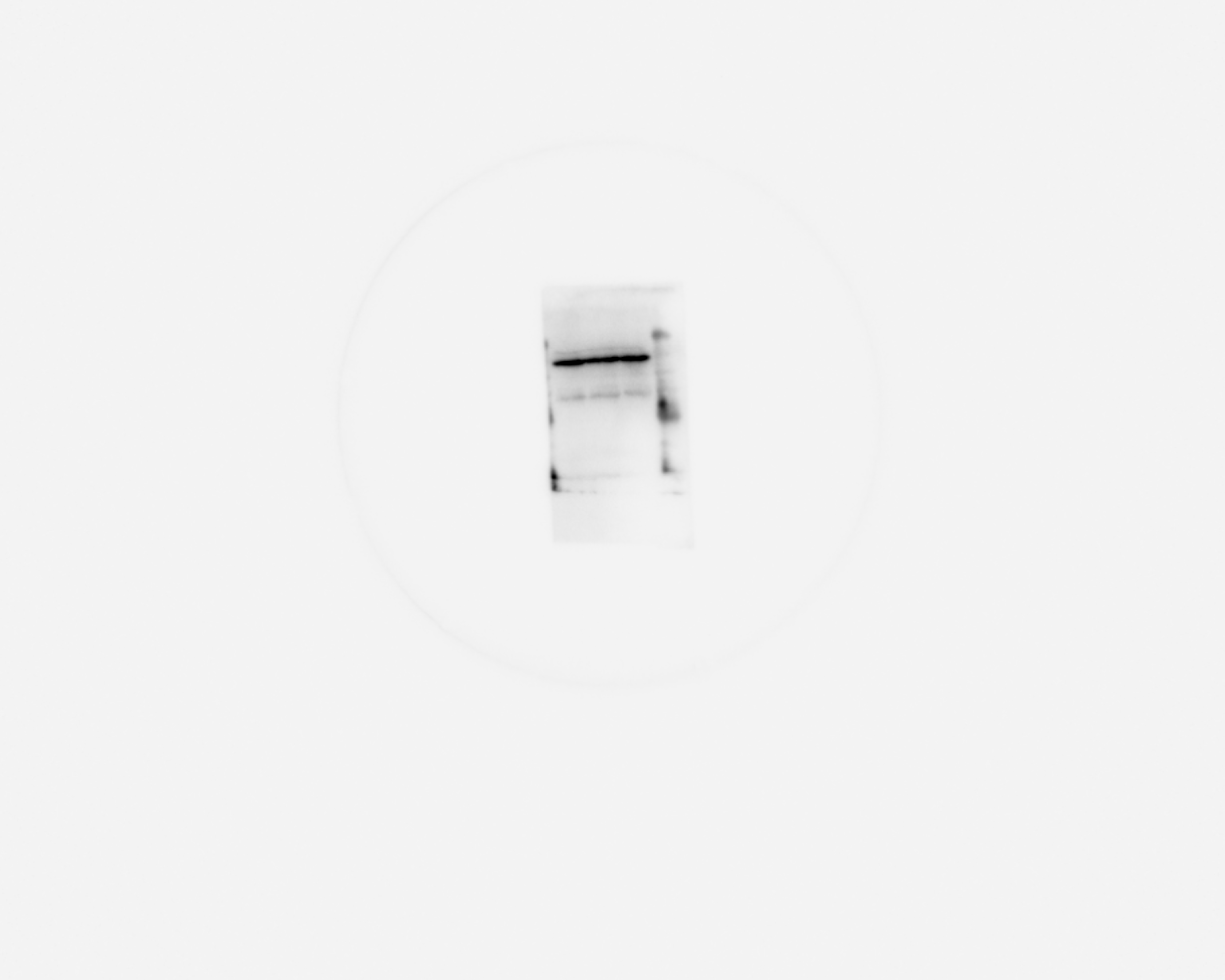

Supplement: Supplementary file 9 [file Data_Sheet_9.zip › P38 mapk-in-1 (hnrnp A1)/original data/wb 2023-01-01 4.2tub.tif]

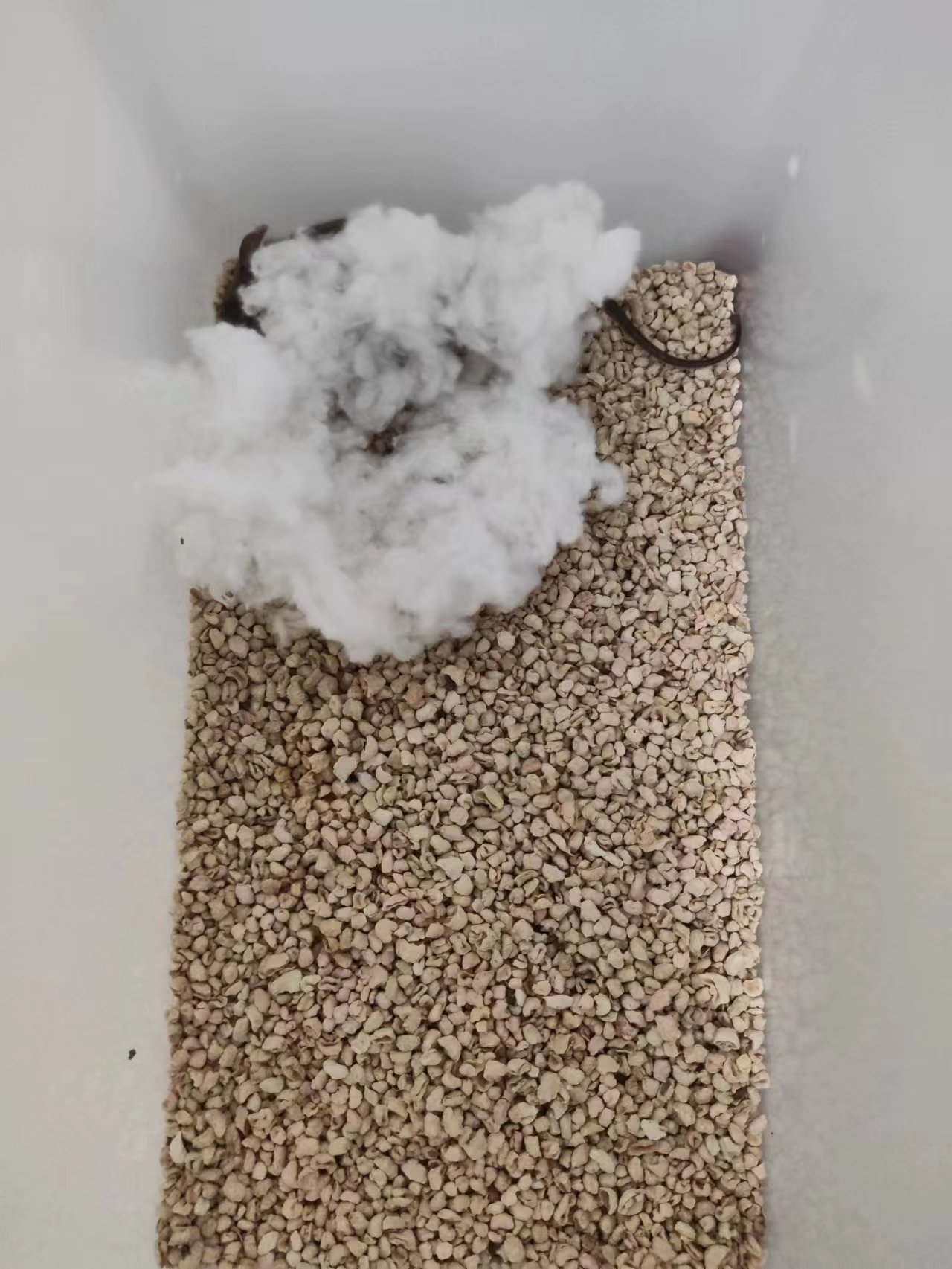

Supplement: Supplementary file 10 [file Data_Sheet_10.zip › FIGS3/nesting/3tg-ad/1.jpg]

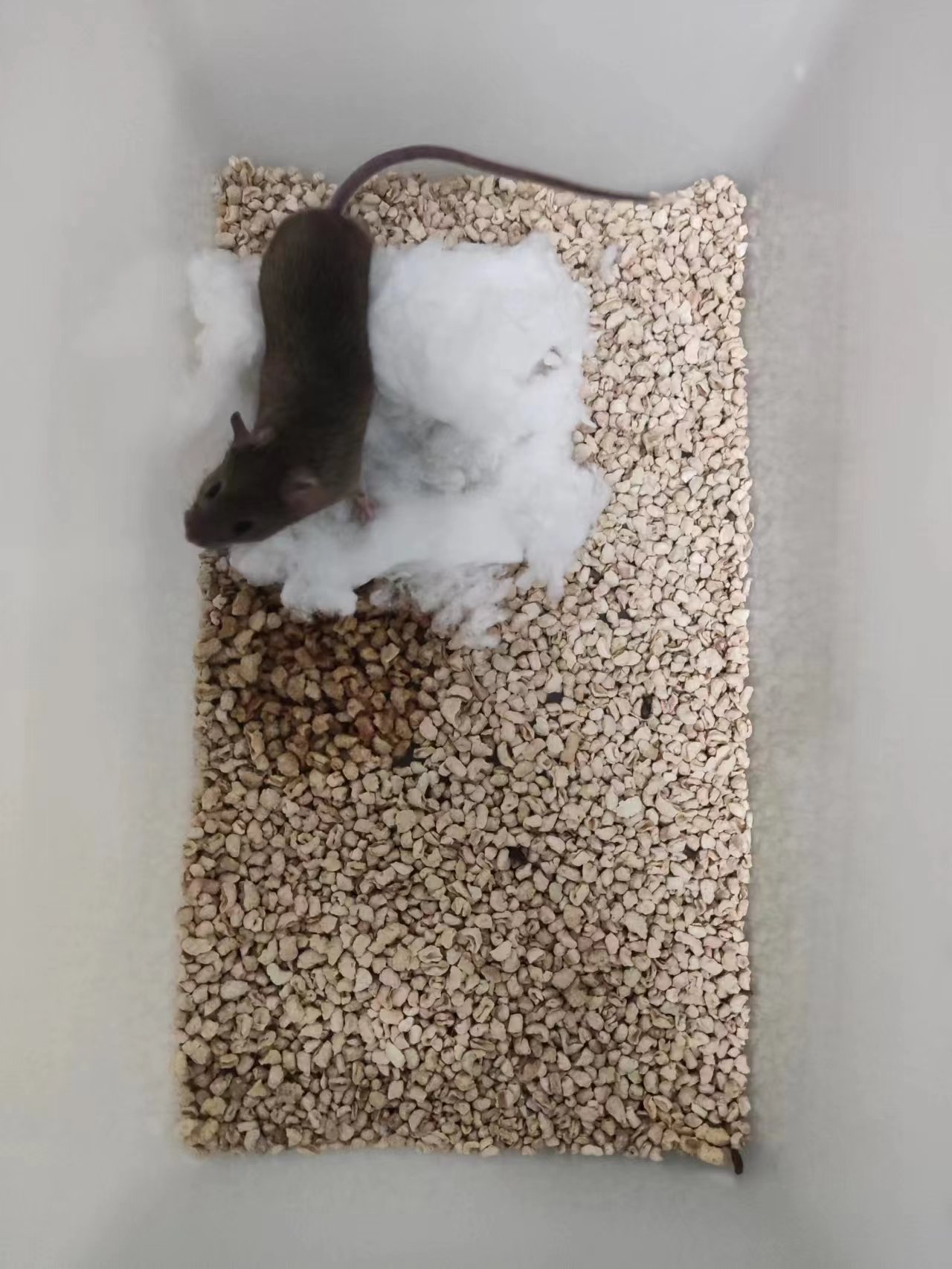

Supplement: Supplementary file 10 [file Data_Sheet_10.zip › FIGS3/nesting/3tg-ad/2.jpg]

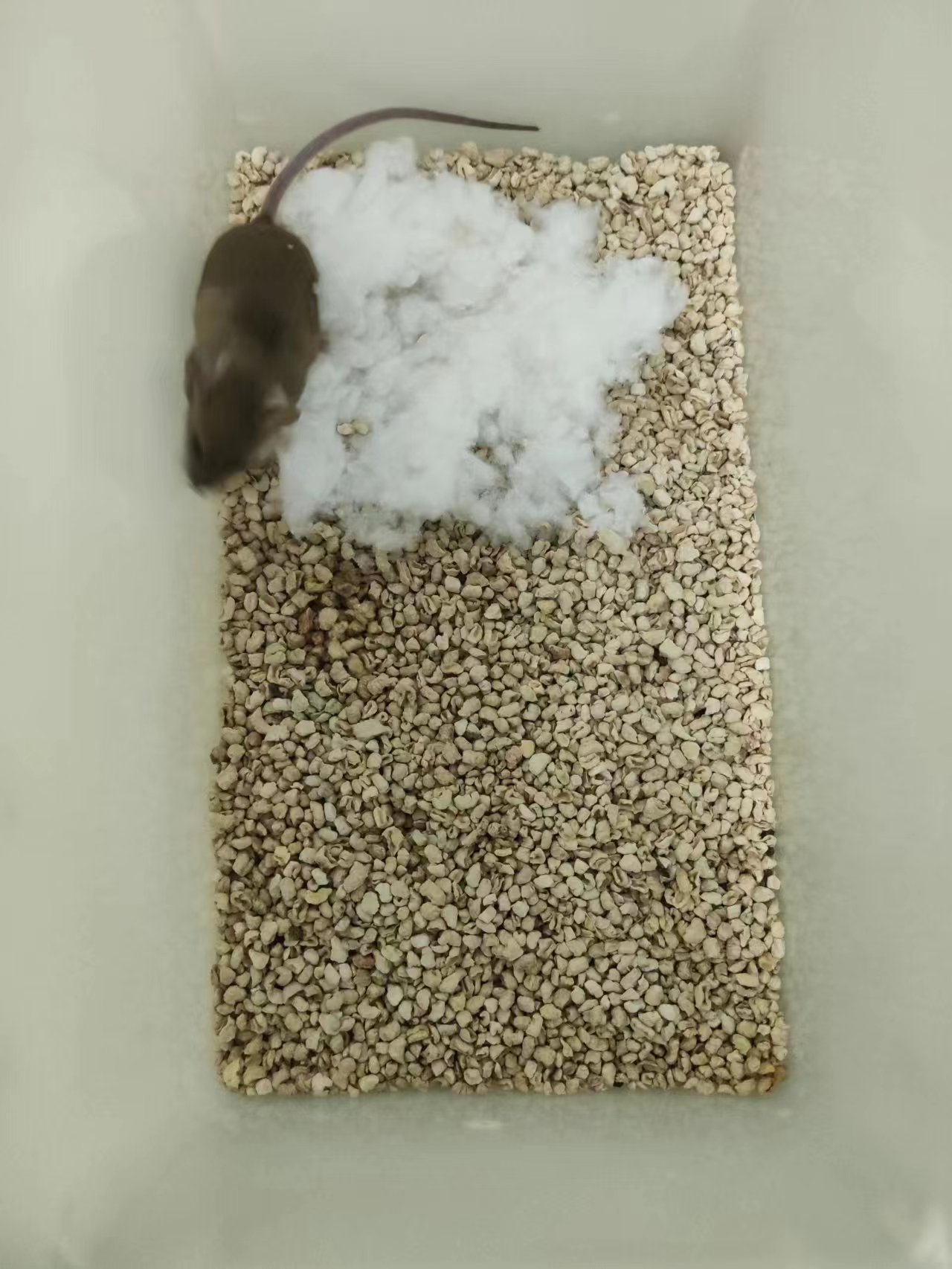

Supplement: Supplementary file 10 [file Data_Sheet_10.zip › FIGS3/nesting/3tg-ad/3.jpg]

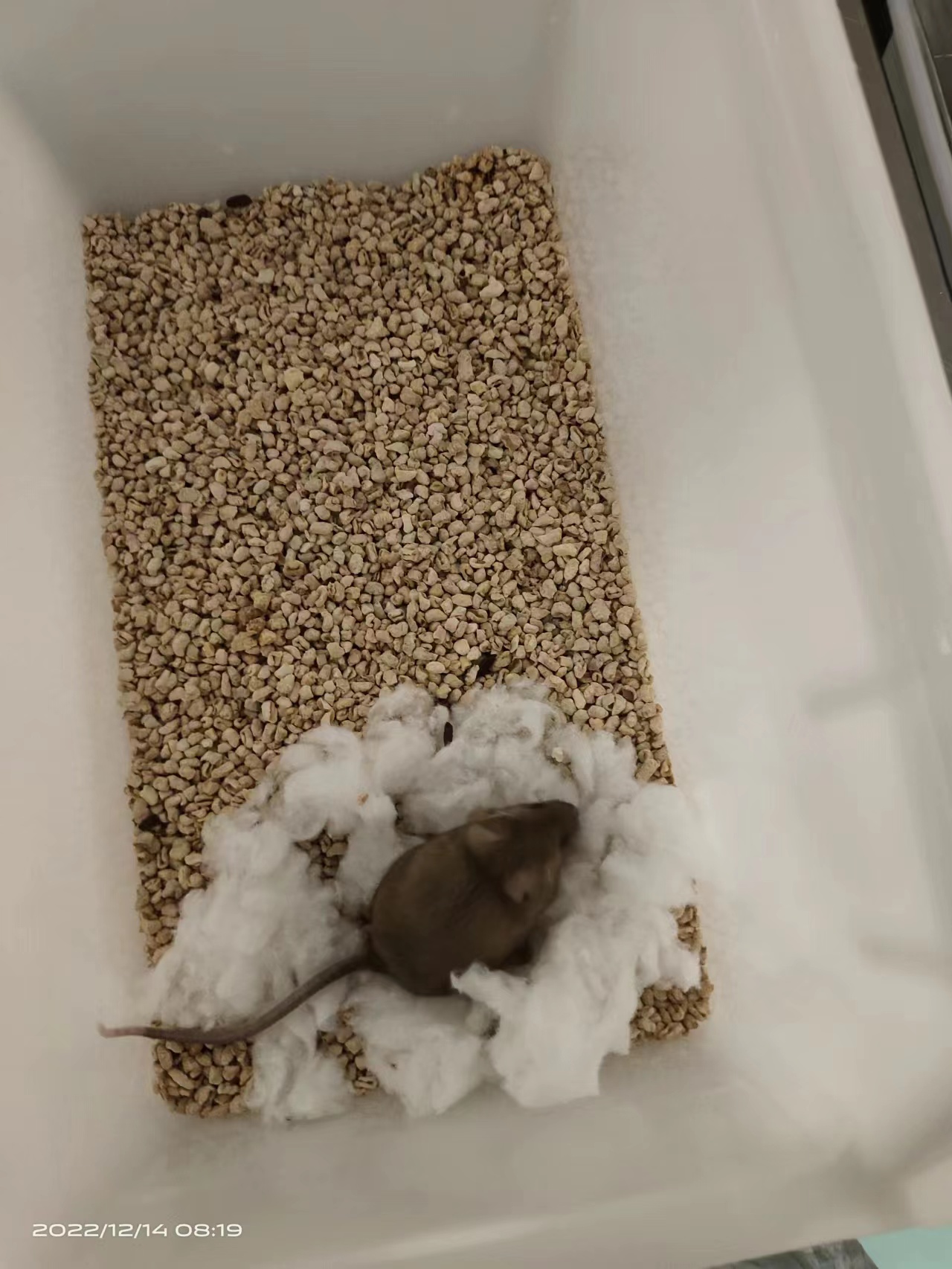

Supplement: Supplementary file 10 [file Data_Sheet_10.zip › FIGS3/nesting/3tg-ad/9abfb500dae95644fde49df9d25a77e.jpg]

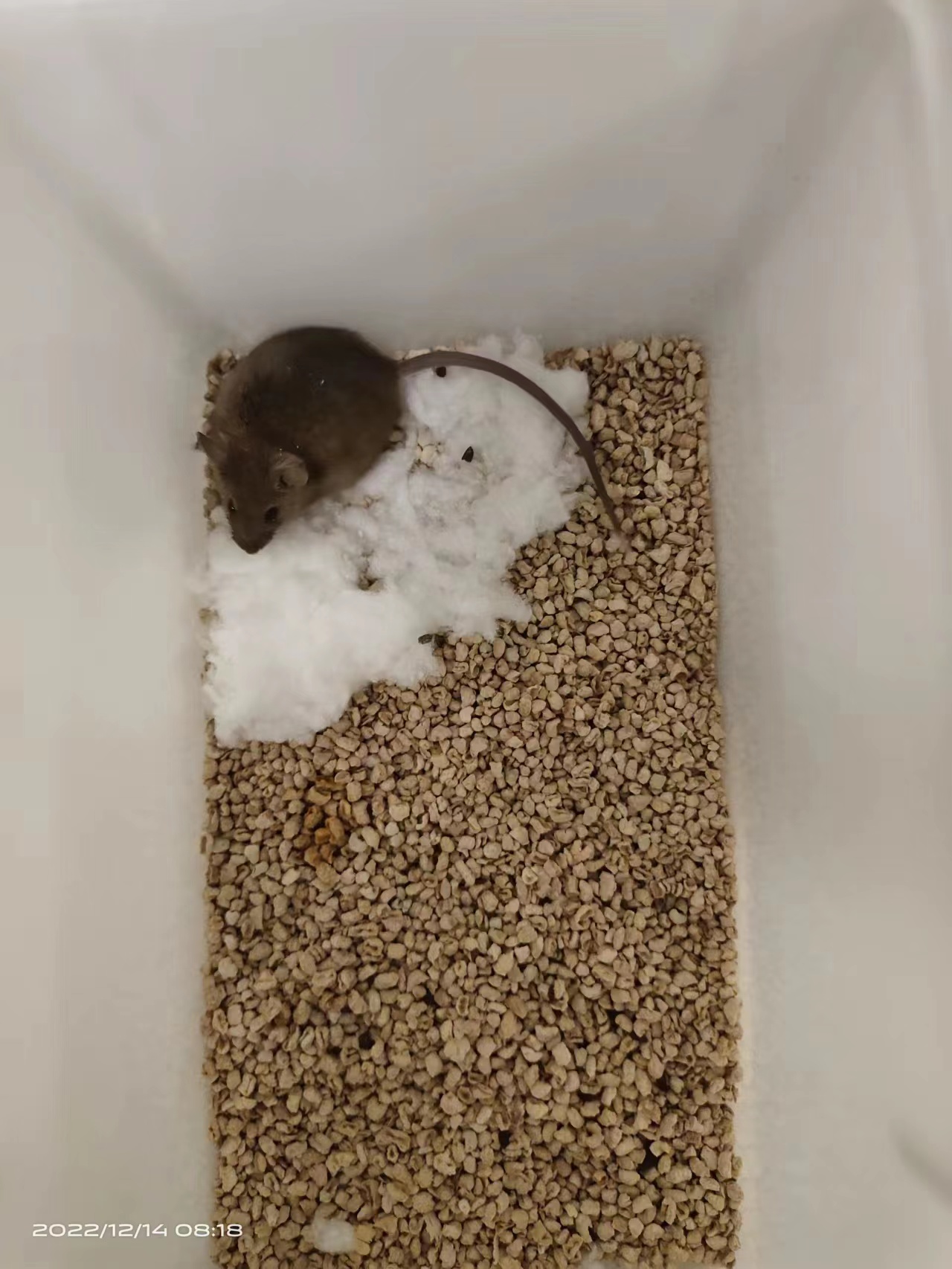

Supplement: Supplementary file 10 [file Data_Sheet_10.zip › FIGS3/nesting/3tg-ad/c15f5fcdfb76e5fb7bba9ad9eba6946.jpg]

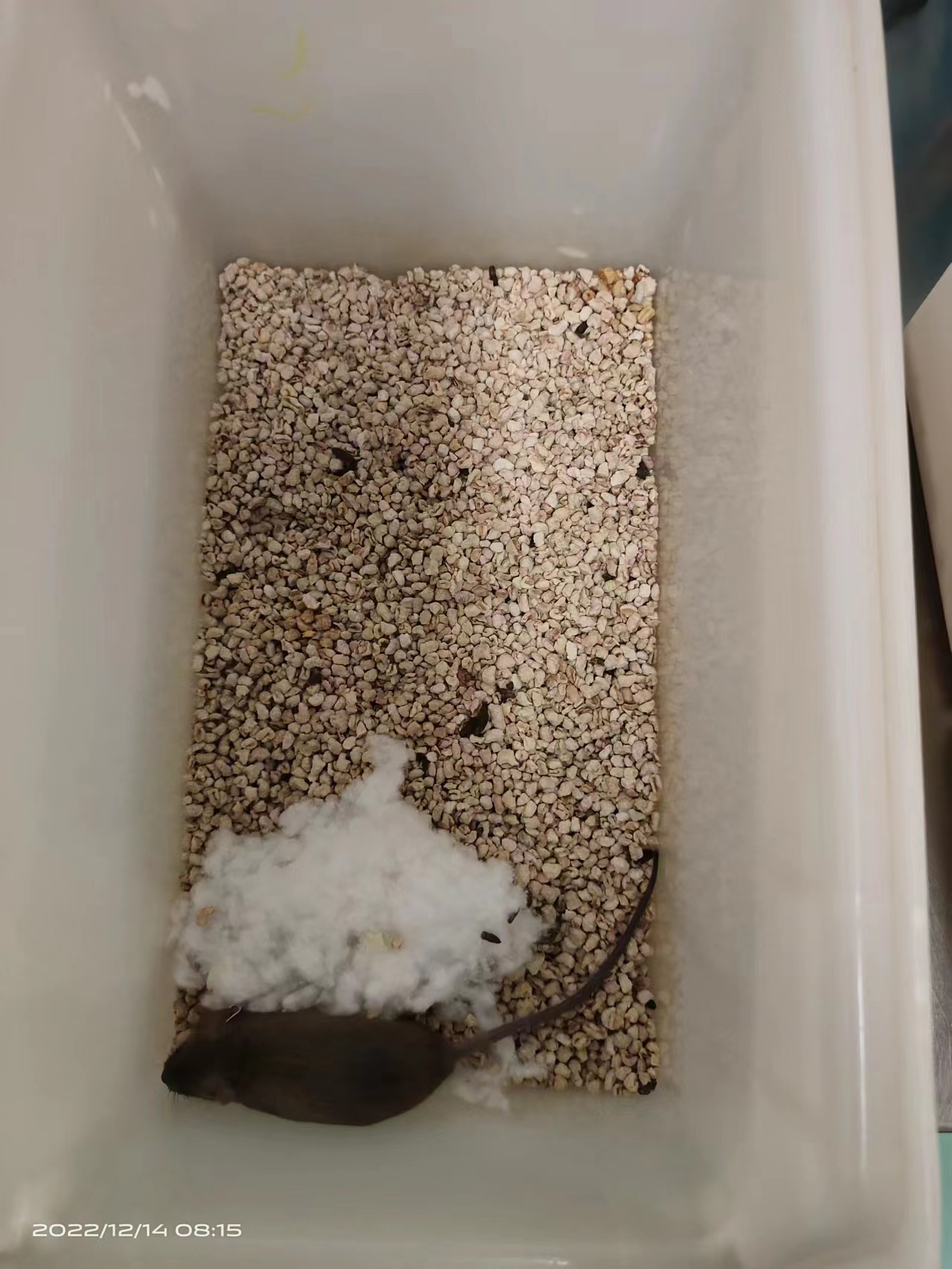

Supplement: Supplementary file 10 [file Data_Sheet_10.zip › FIGS3/nesting/3tg-ad/e6aee436b9fdd1853ad6c6fe26714a1.jpg]

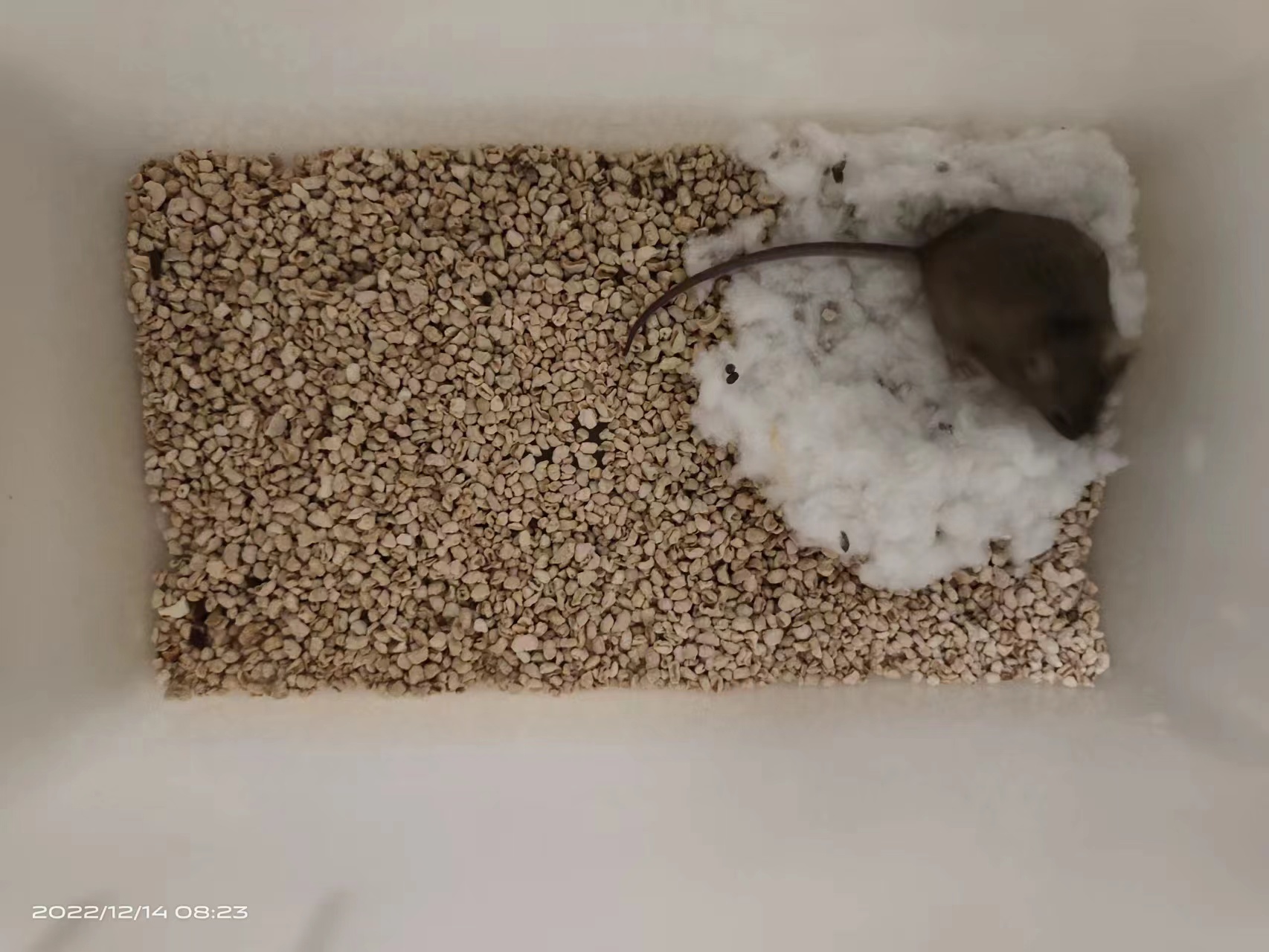

Supplement: Supplementary file 10 [file Data_Sheet_10.zip › FIGS3/nesting/wt/259dc2c15859b4a8afbc731425e340d.jpg]

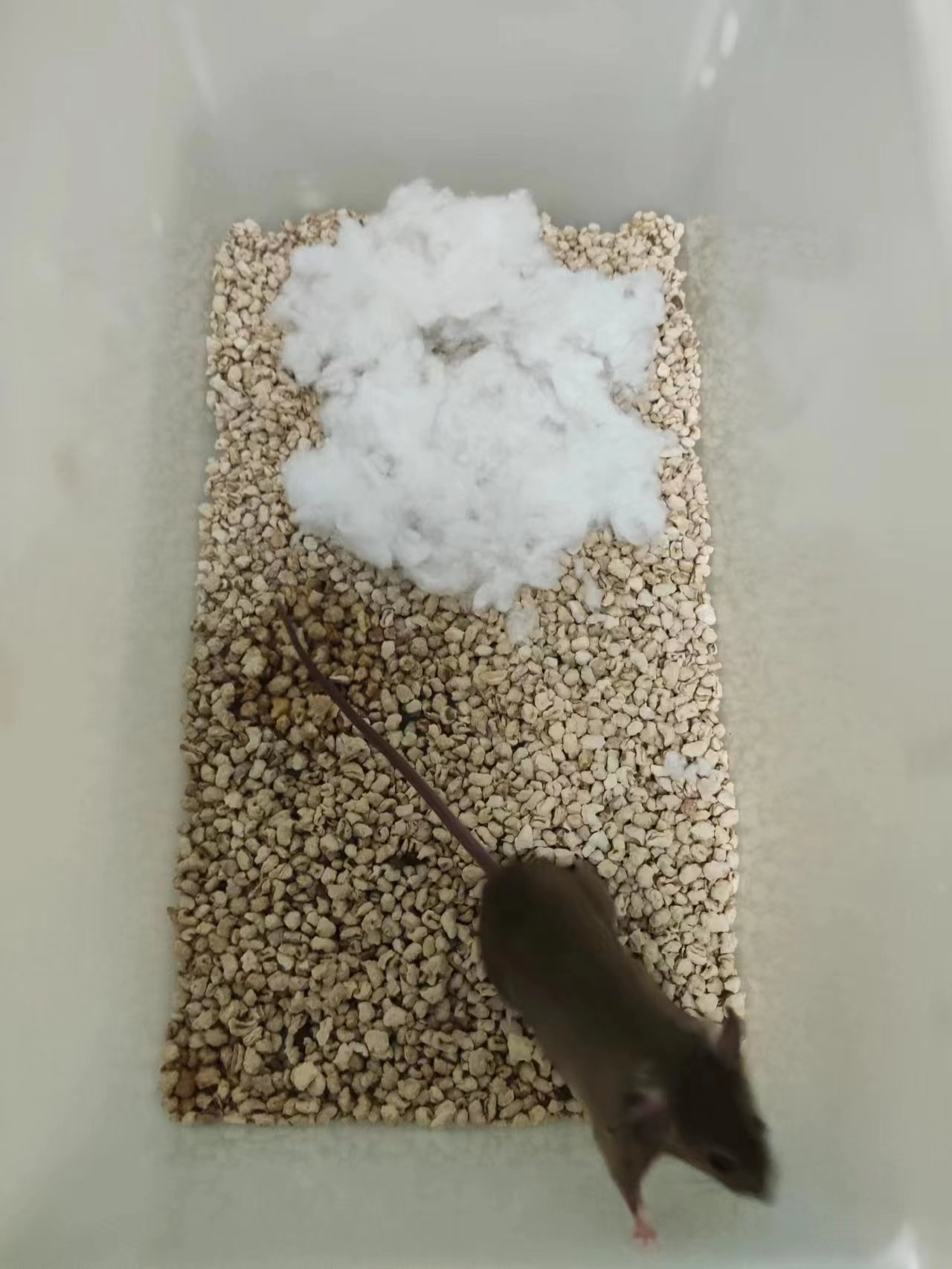

Supplement: Supplementary file 10 [file Data_Sheet_10.zip › FIGS3/nesting/wt/4.jpg]

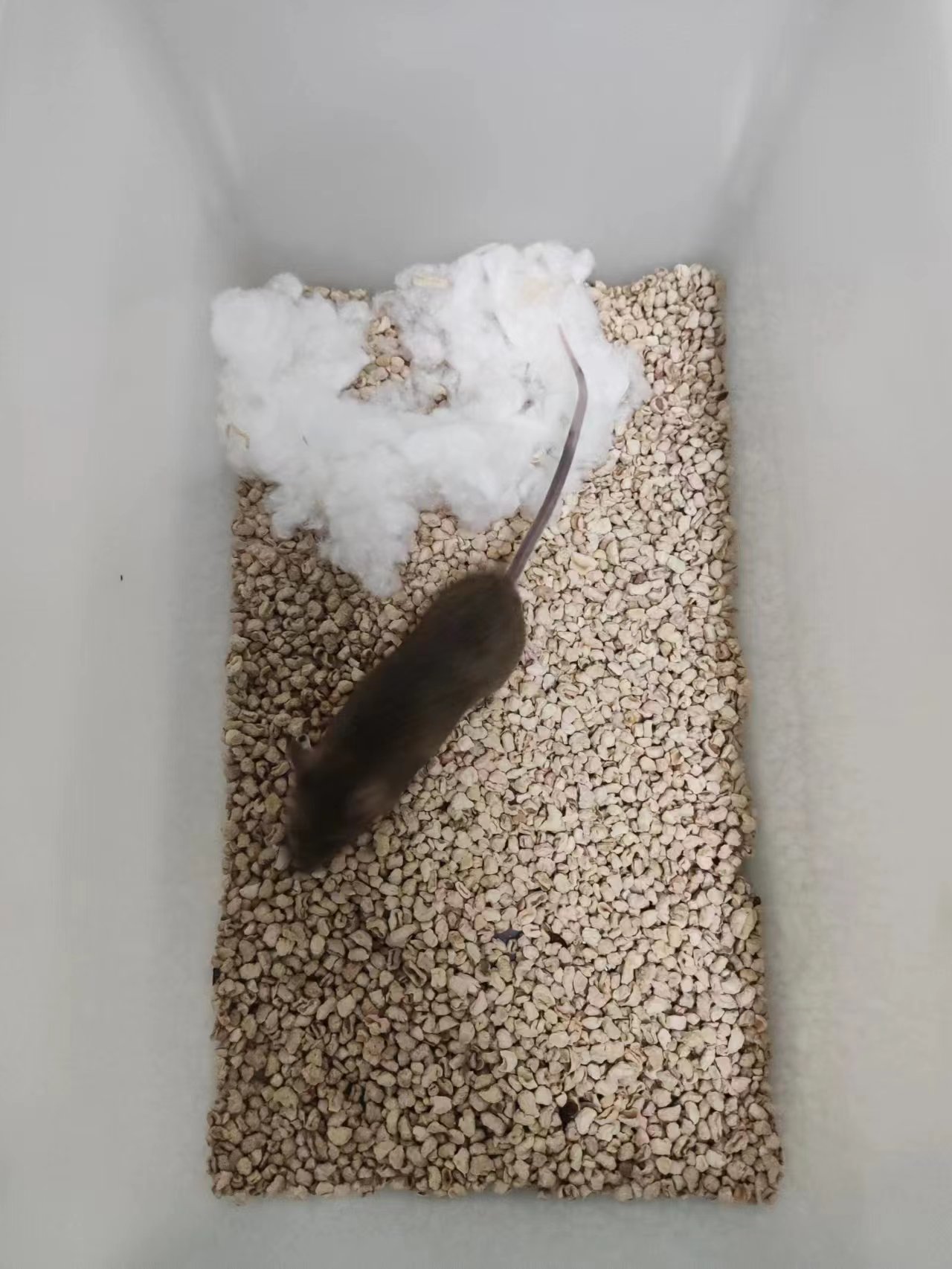

Supplement: Supplementary file 10 [file Data_Sheet_10.zip › FIGS3/nesting/wt/5.jpg]

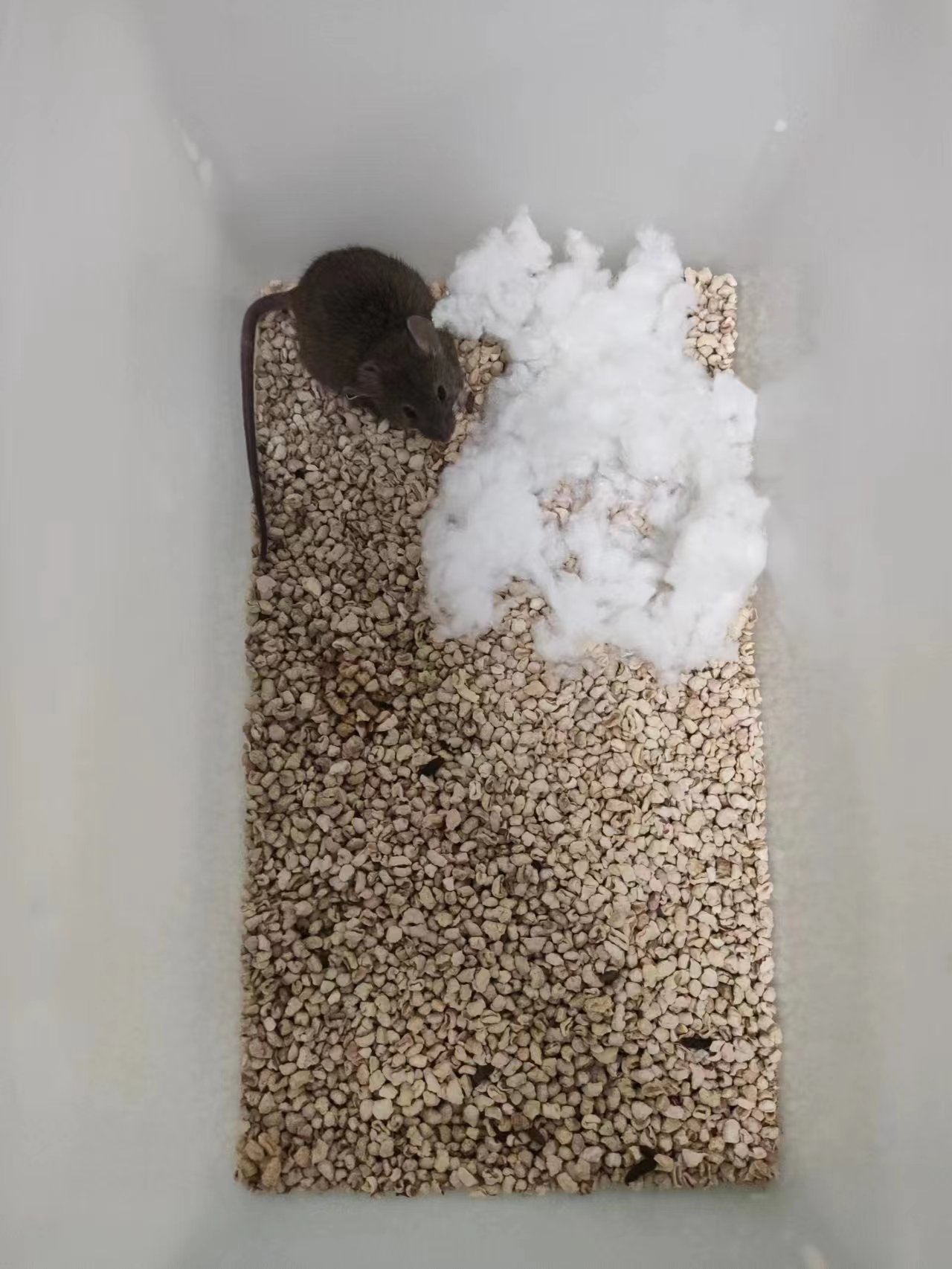

Supplement: Supplementary file 10 [file Data_Sheet_10.zip › FIGS3/nesting/wt/6.jpg]

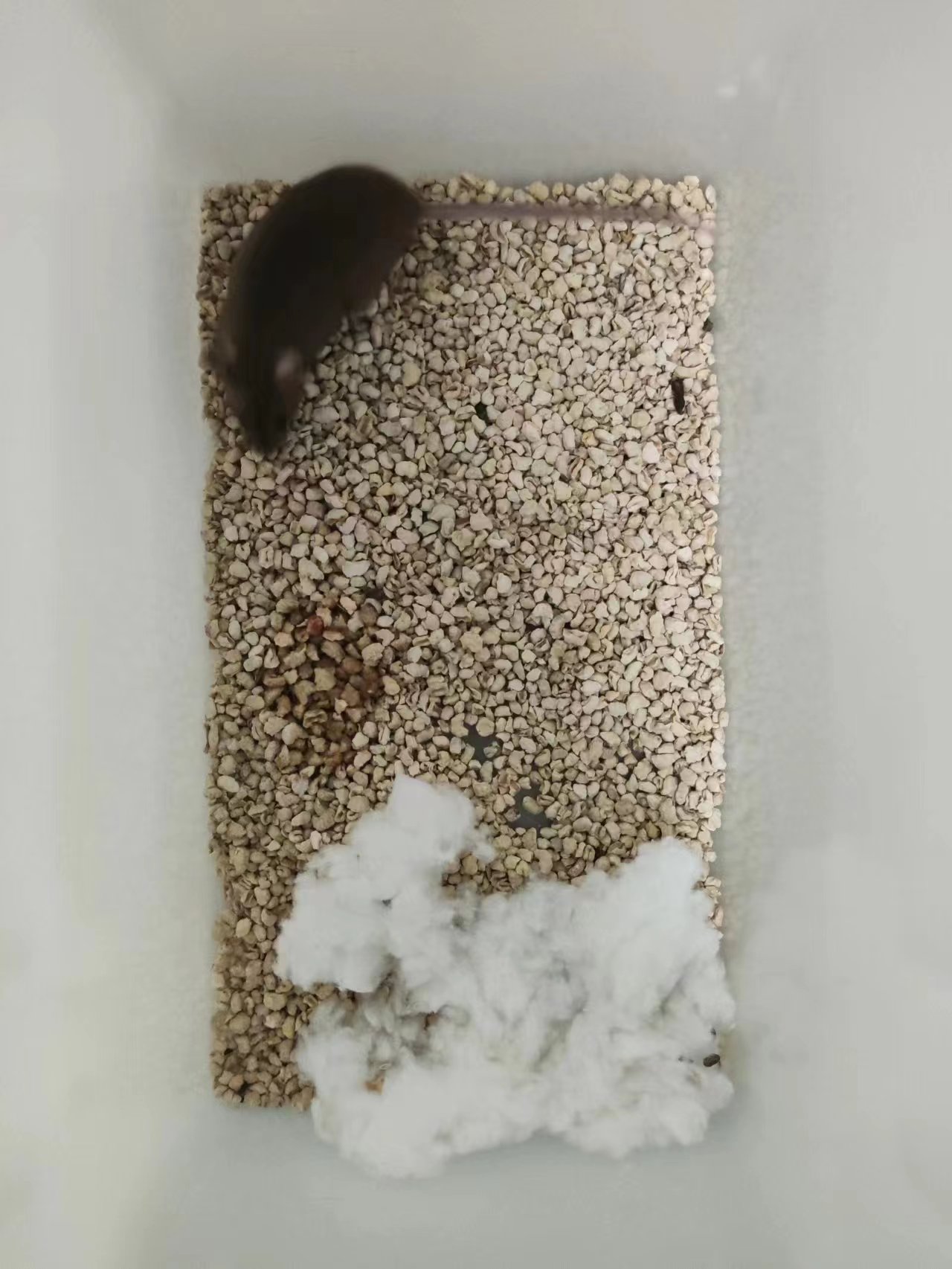

Supplement: Supplementary file 10 [file Data_Sheet_10.zip › FIGS3/nesting/wt/7.jpg]

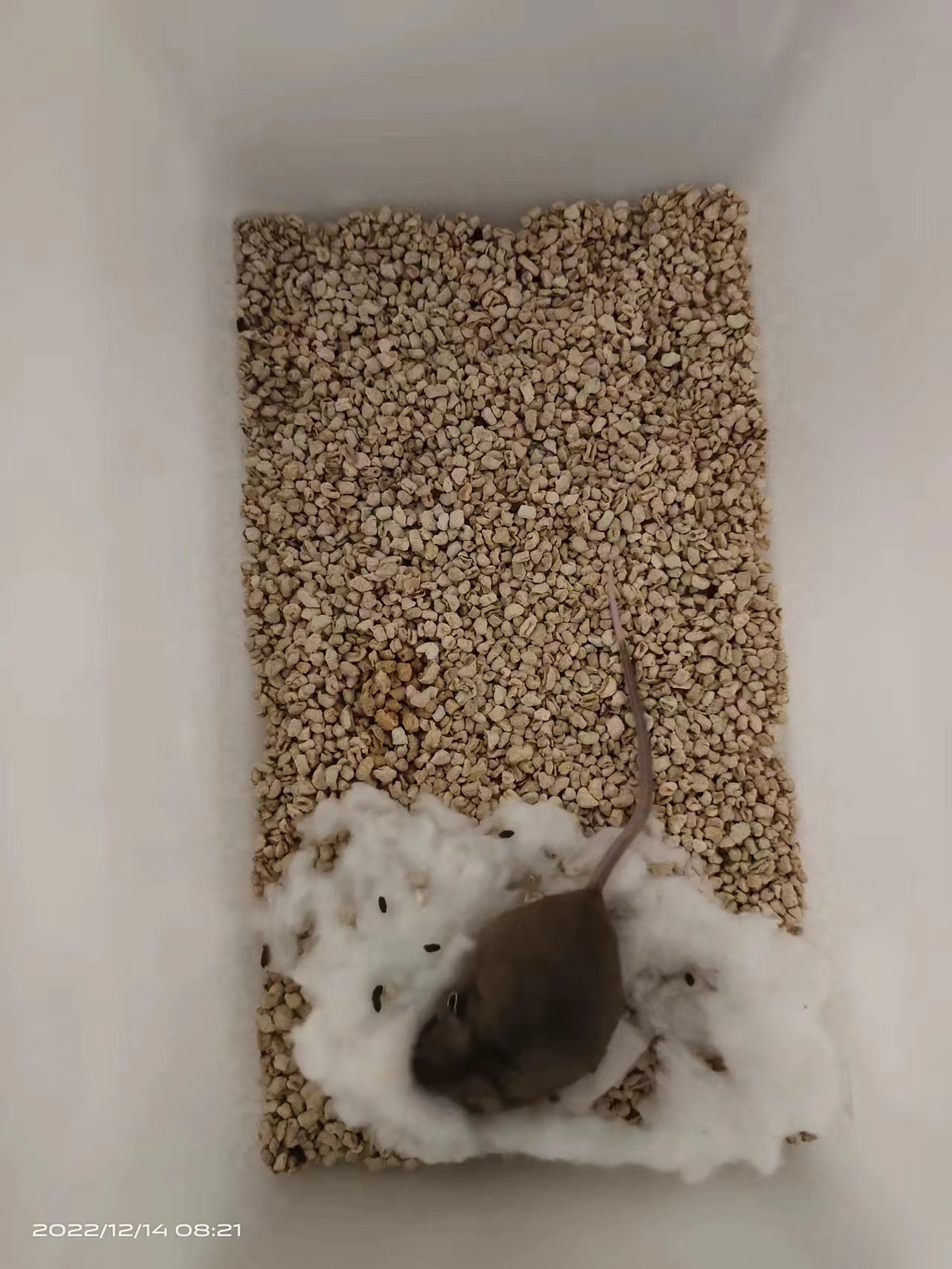

Supplement: Supplementary file 10 [file Data_Sheet_10.zip › FIGS3/nesting/wt/db5b4759b85fa6d951057ebe7187046.jpg]

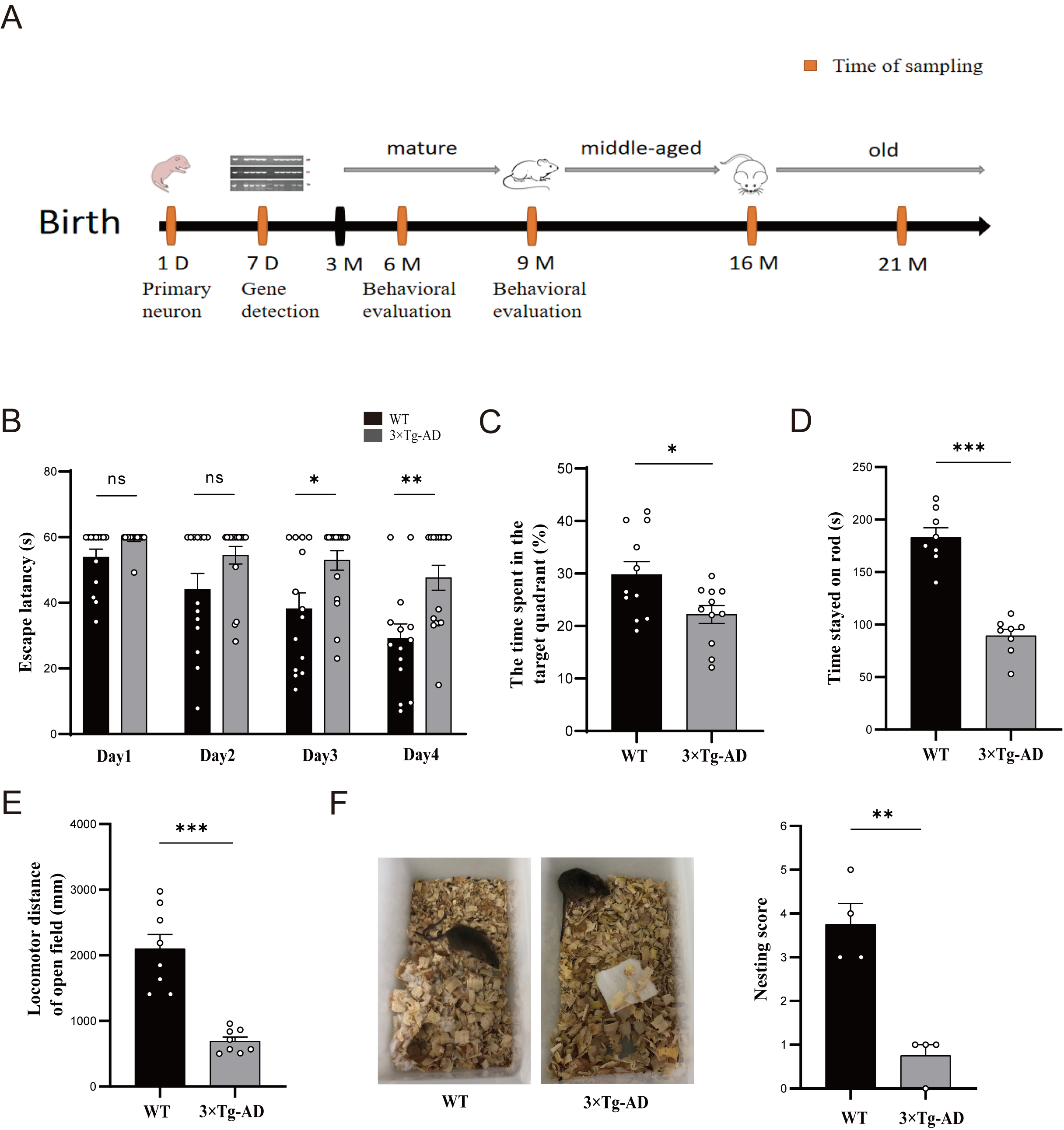

Supplement: Supplementary file 11 [file Data_Sheet_11.zip › FIGS1/FIG1.tif]

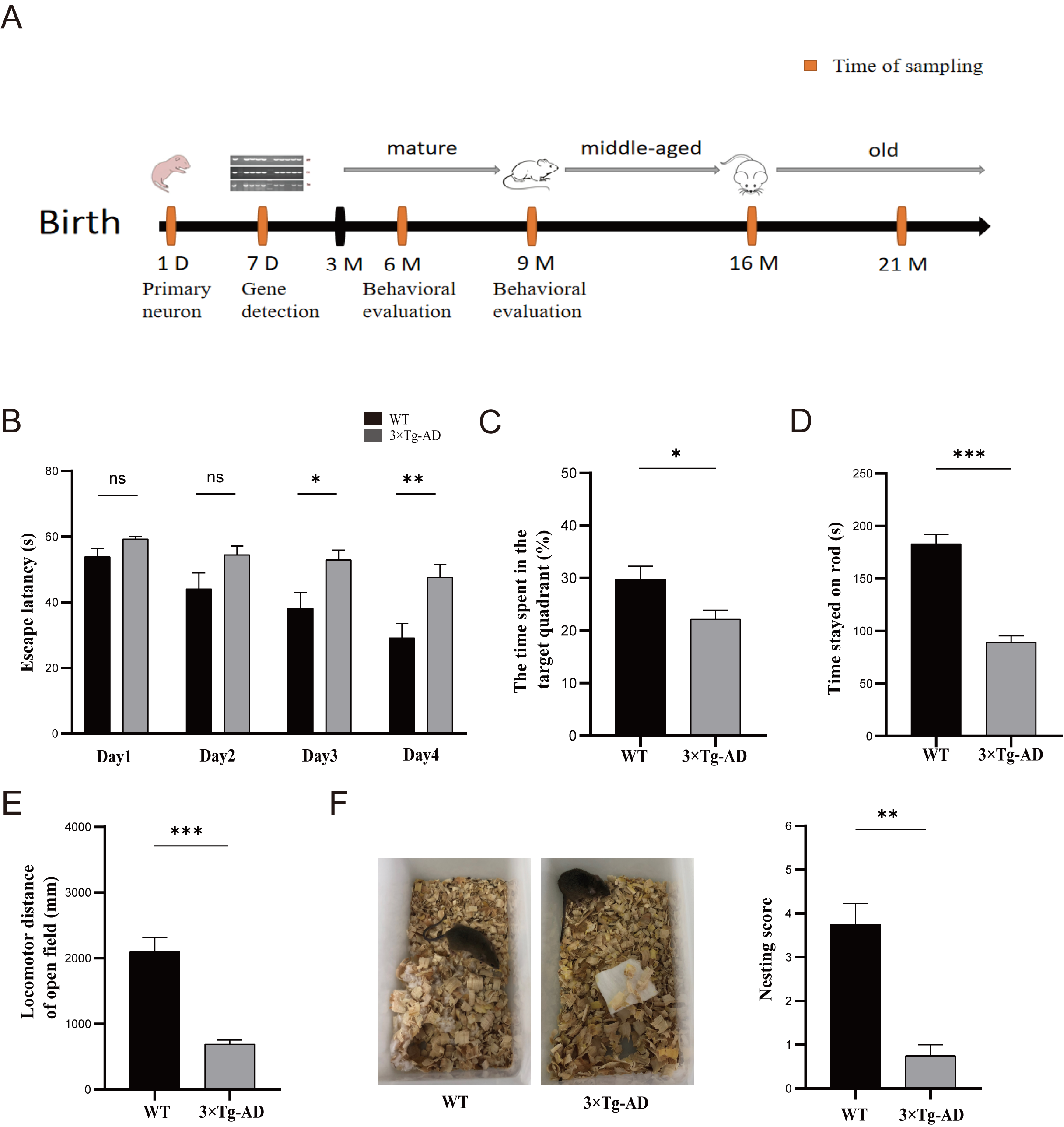

Supplement: Supplementary file 11 [file Data_Sheet_11.zip › FIGS1/FIGs1.tif]

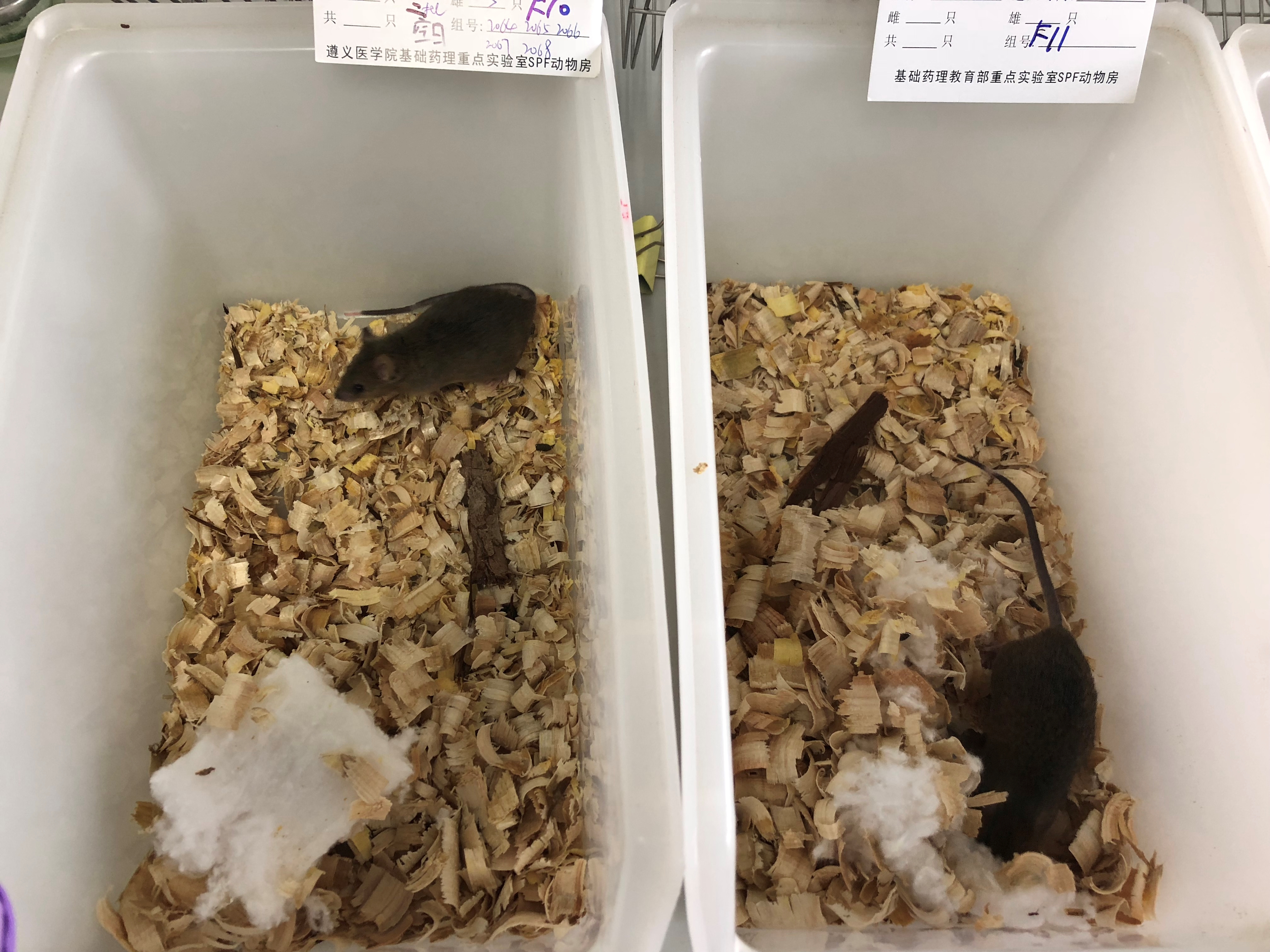

Supplement: Supplementary file 11 [file Data_Sheet_11.zip › FIGS1/Nest building experiment/K1 K2.JPG]

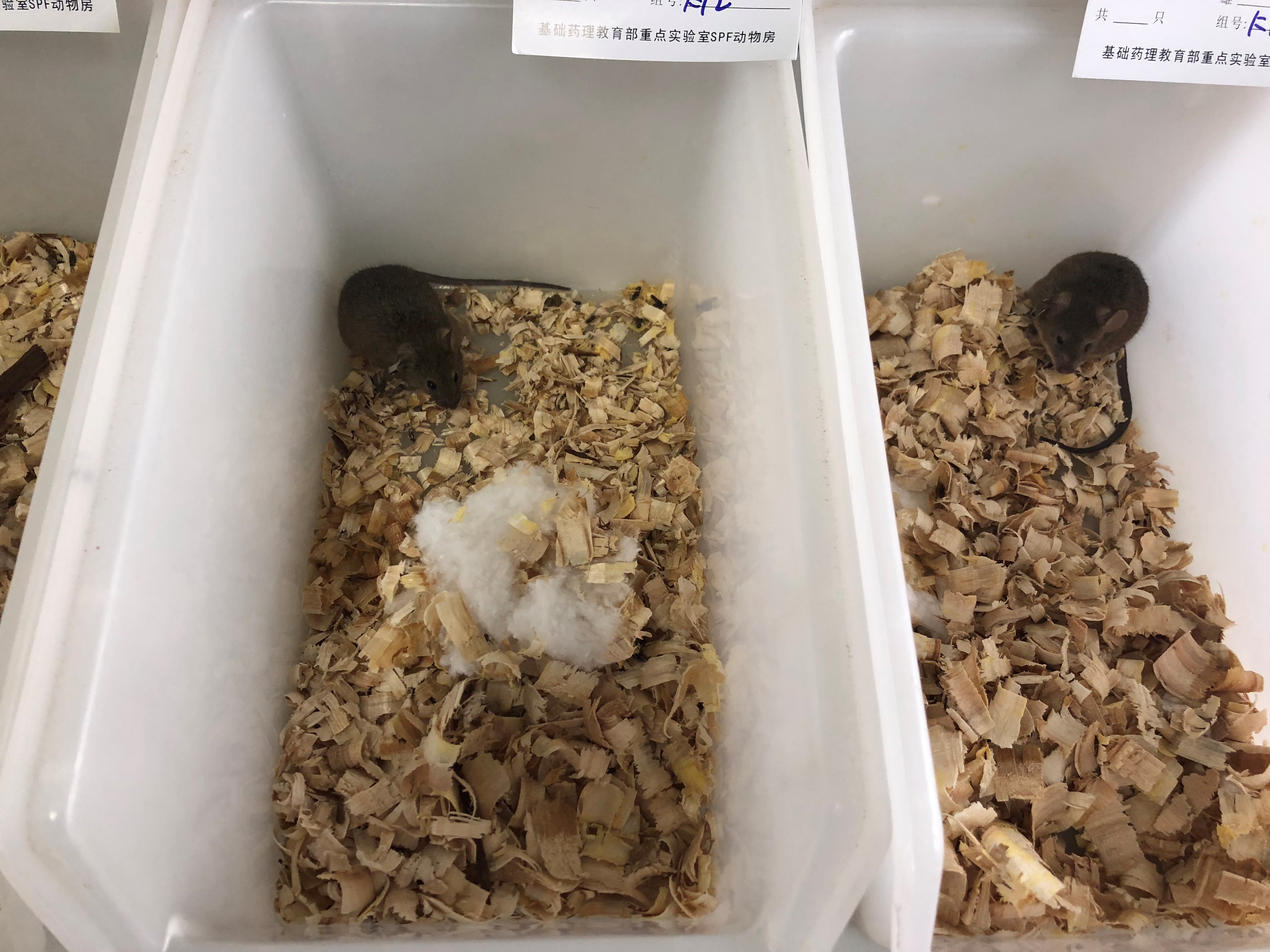

Supplement: Supplementary file 11 [file Data_Sheet_11.zip › FIGS1/Nest building experiment/K3 K4.JPG]

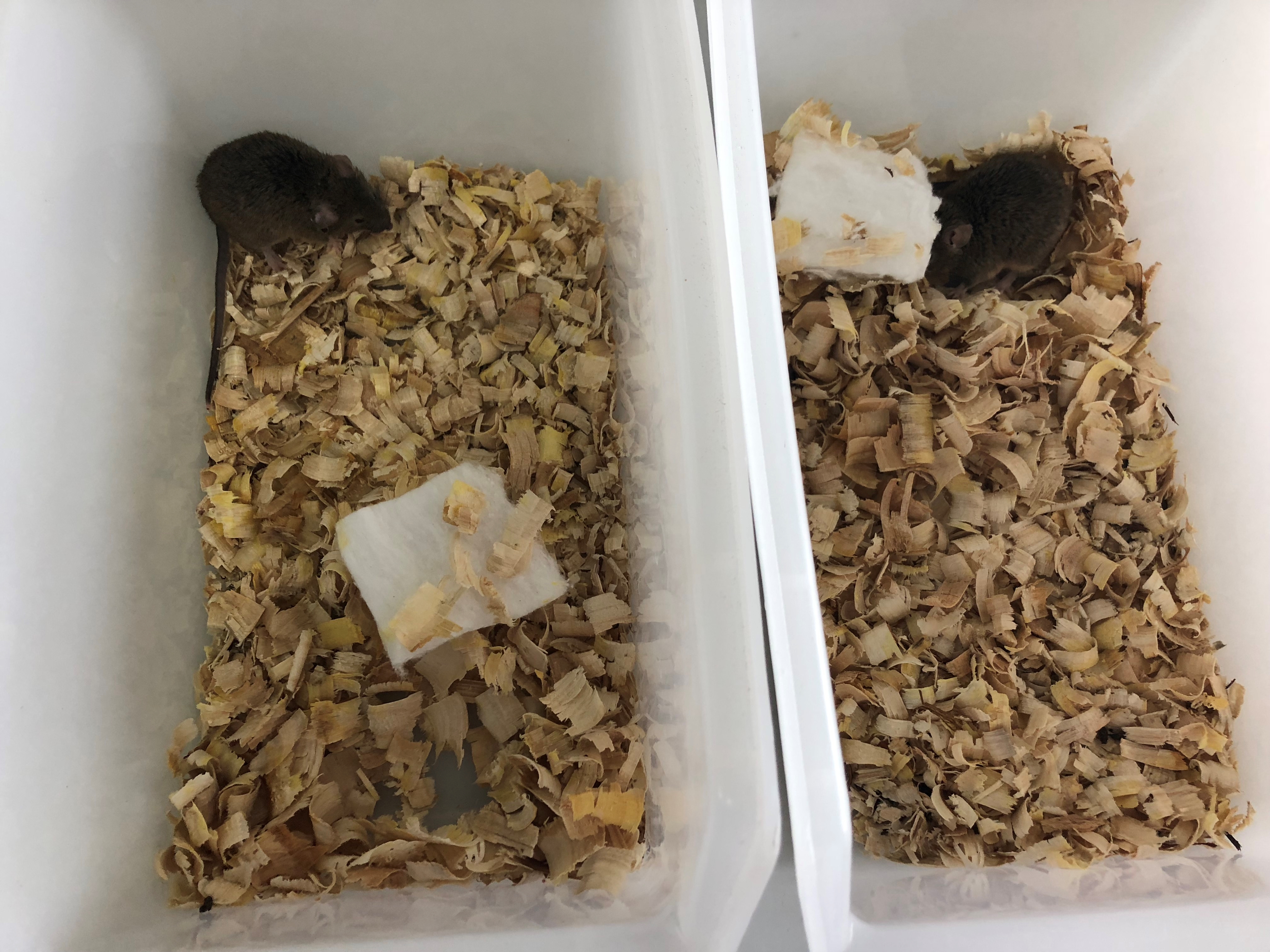

Supplement: Supplementary file 11 [file Data_Sheet_11.zip › FIGS1/Nest building experiment/M1 M2.JPG]

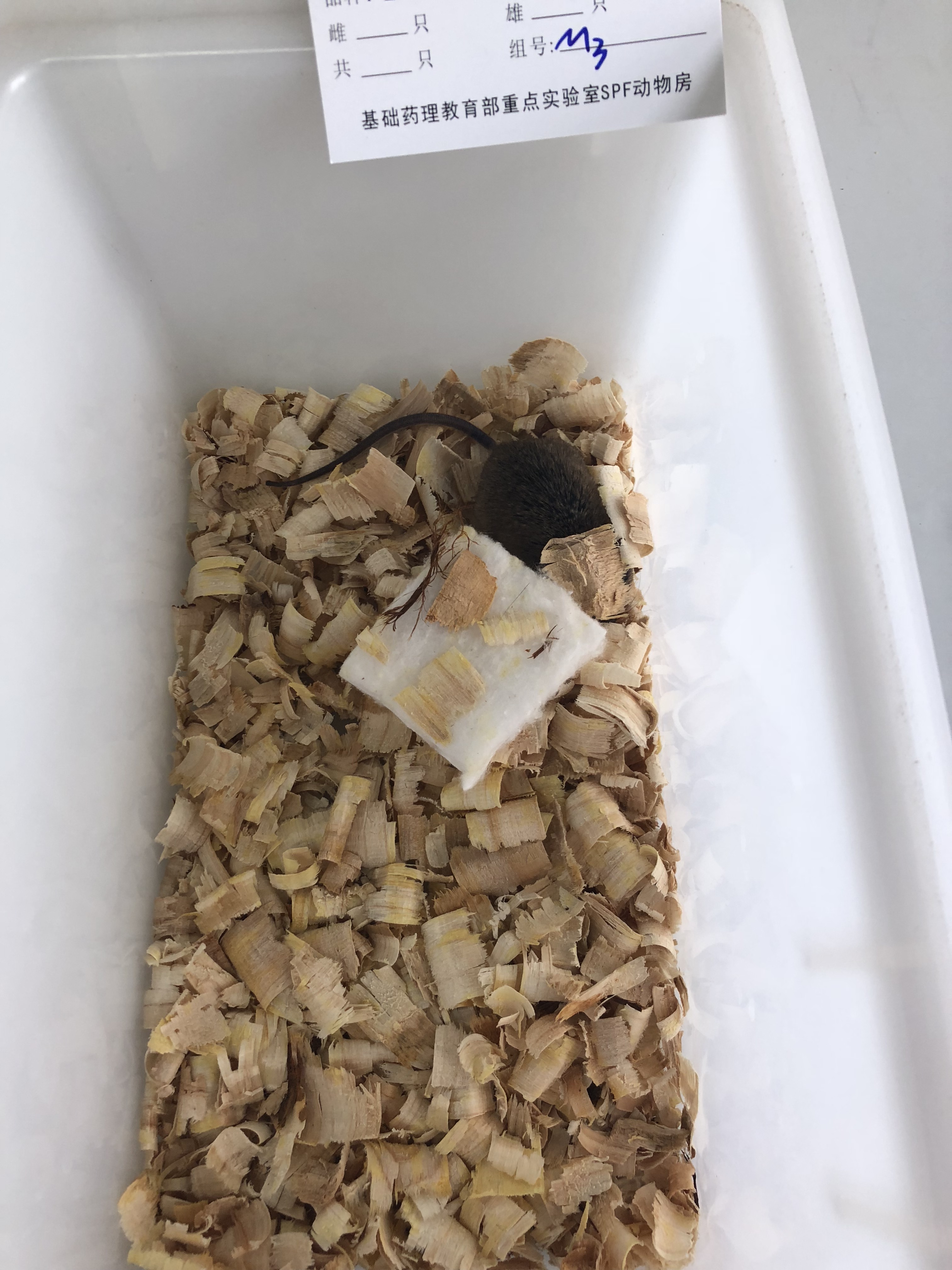

Supplement: Supplementary file 11 [file Data_Sheet_11.zip › FIGS1/Nest building experiment/M3.JPG]

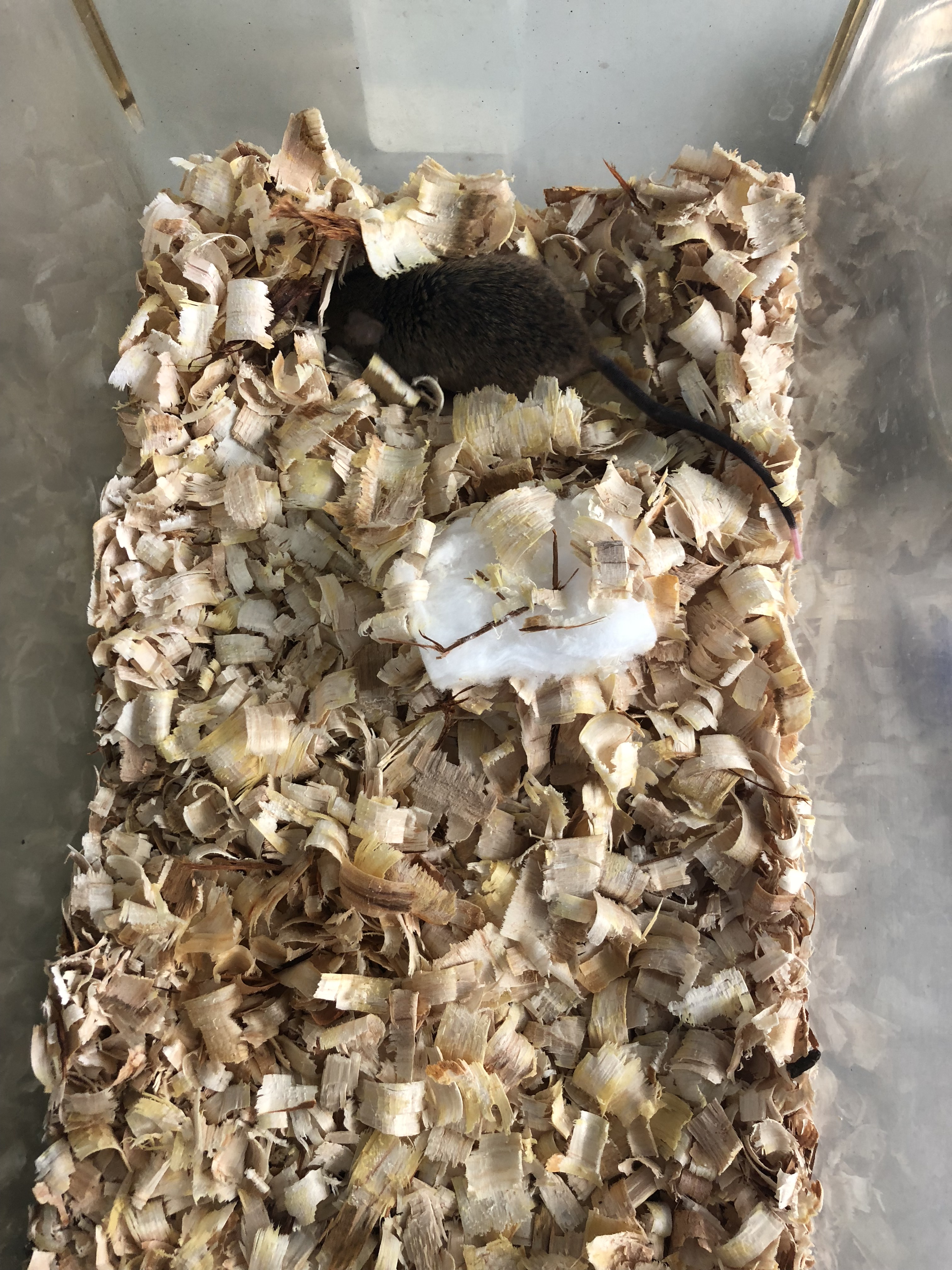

Supplement: Supplementary file 11 [file Data_Sheet_11.zip › FIGS1/Nest building experiment/M4.JPG]

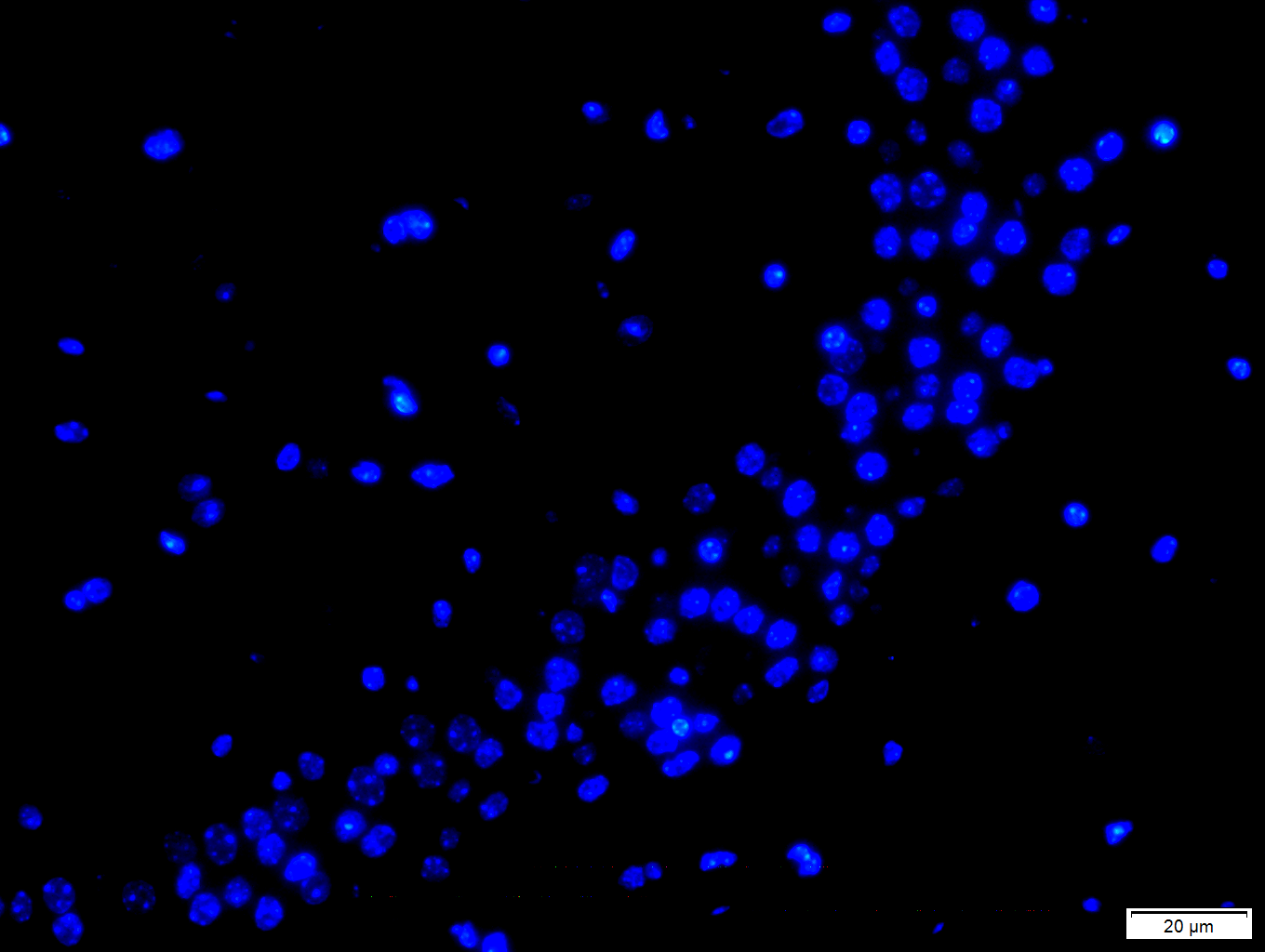

Supplement: Supplementary file 12 [file Data_Sheet_12.zip › Aβ immunofluorescence/CA3/3TG/M1/M01 CA3 DAPI.tif]

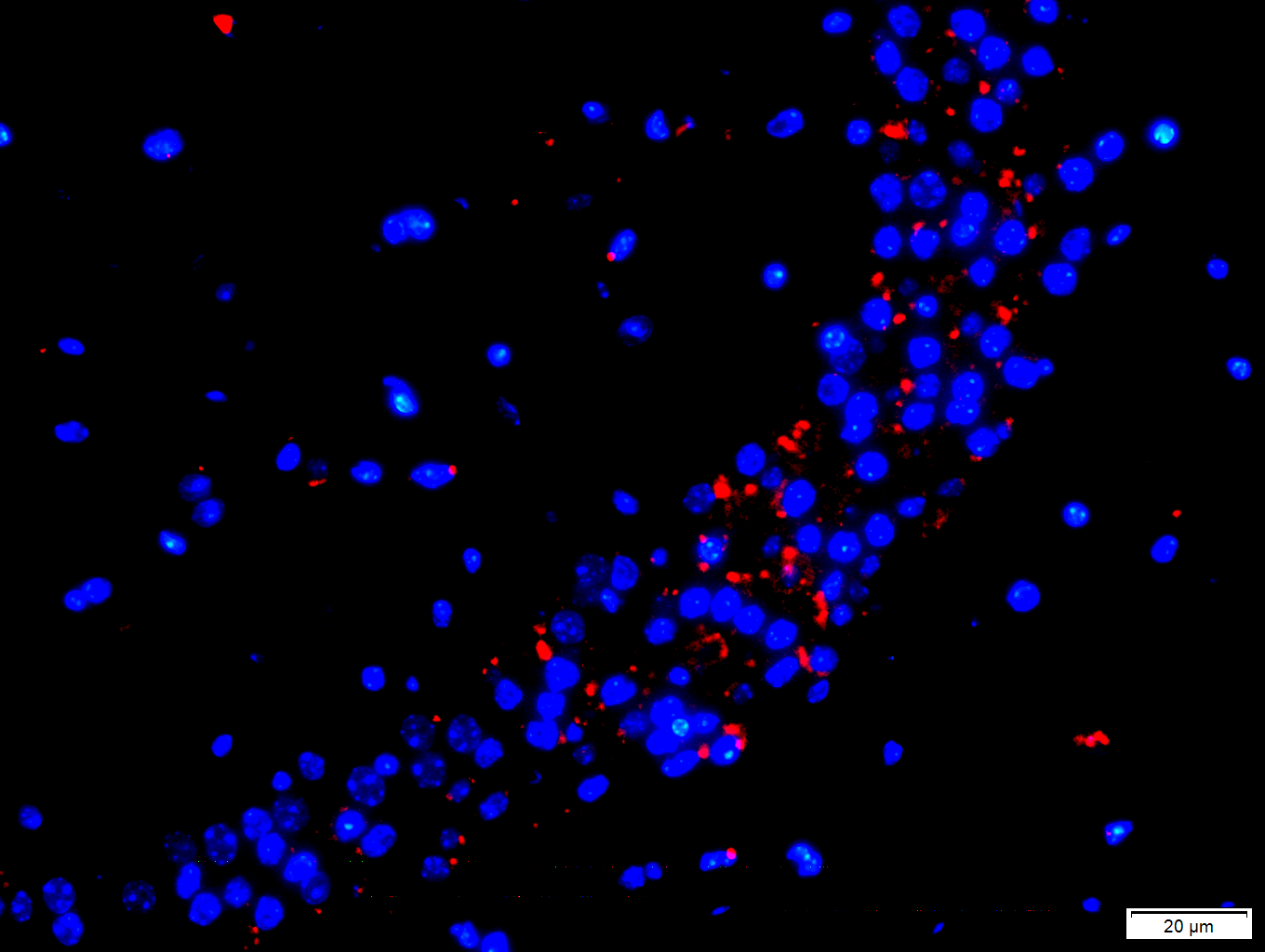

Supplement: Supplementary file 12 [file Data_Sheet_12.zip › Aβ immunofluorescence/CA3/3TG/M1/M01 CA3 merge.tif]

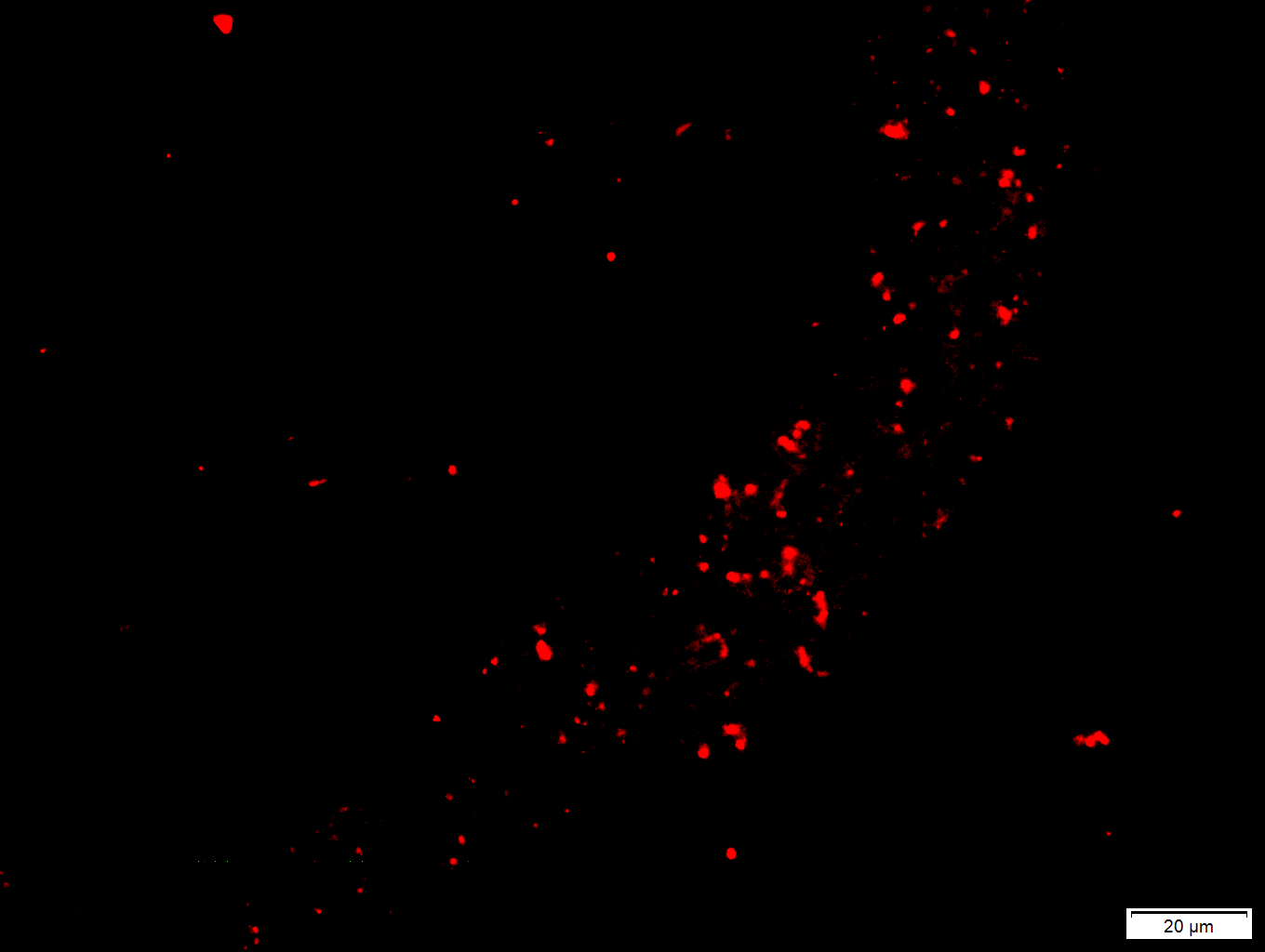

Supplement: Supplementary file 12 [file Data_Sheet_12.zip › Aβ immunofluorescence/CA3/3TG/M1/M01 CA3.tif]

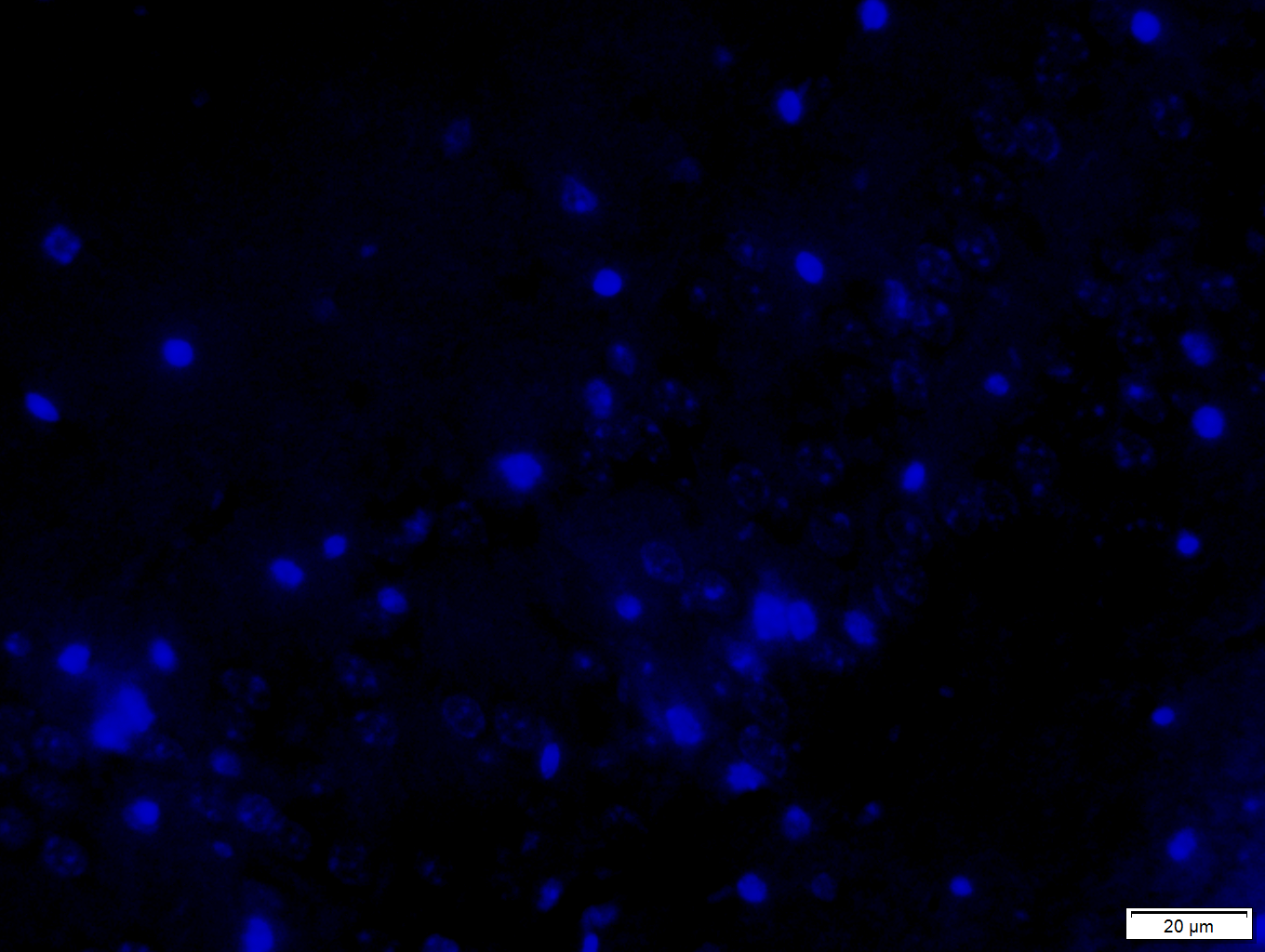

Supplement: Supplementary file 12 [file Data_Sheet_12.zip › Aβ immunofluorescence/CA3/3TG/M4/40X CA3 DAPI.tif]

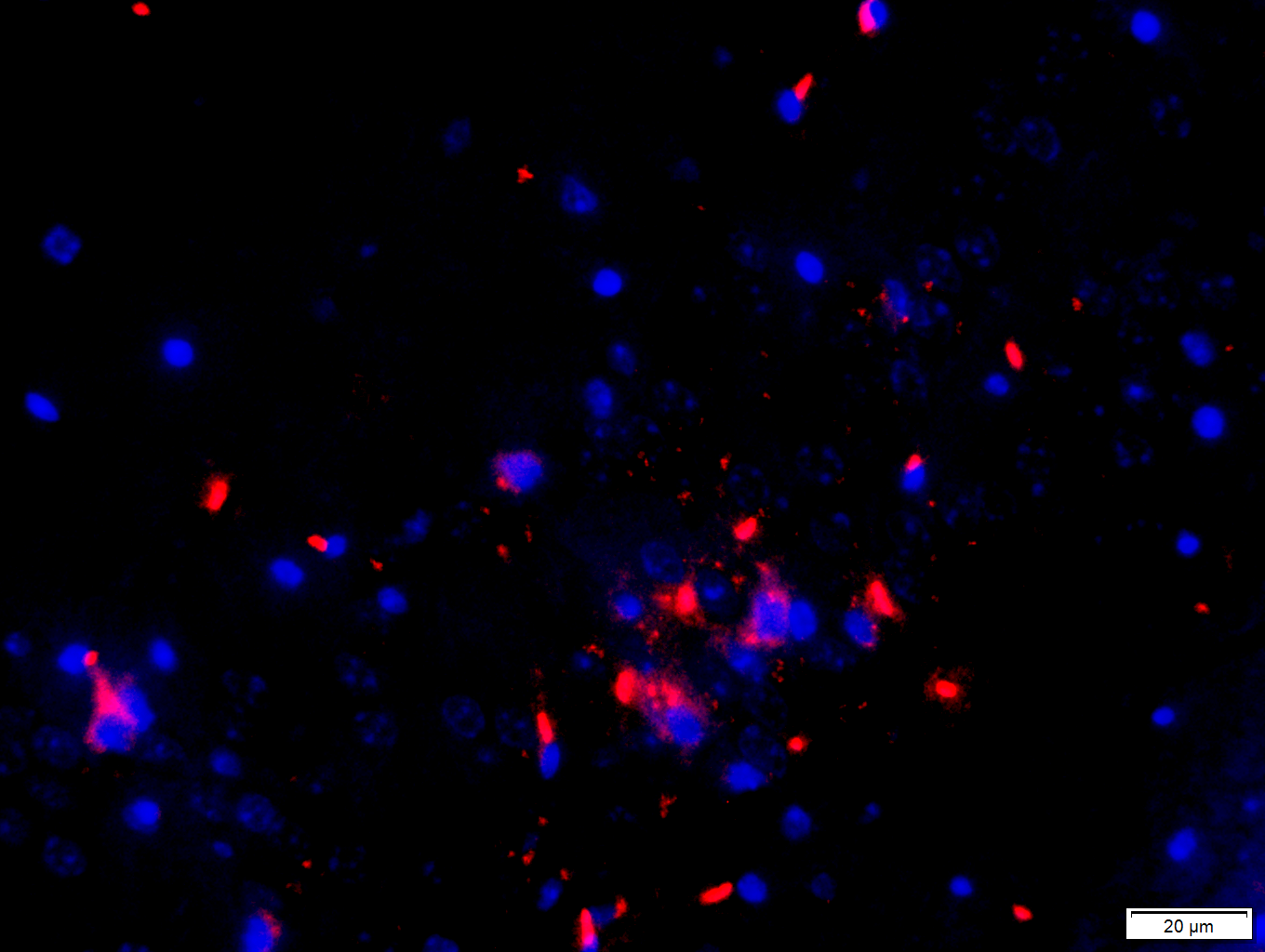

Supplement: Supplementary file 12 [file Data_Sheet_12.zip › Aβ immunofluorescence/CA3/3TG/M4/40X CA3 merge.tif]

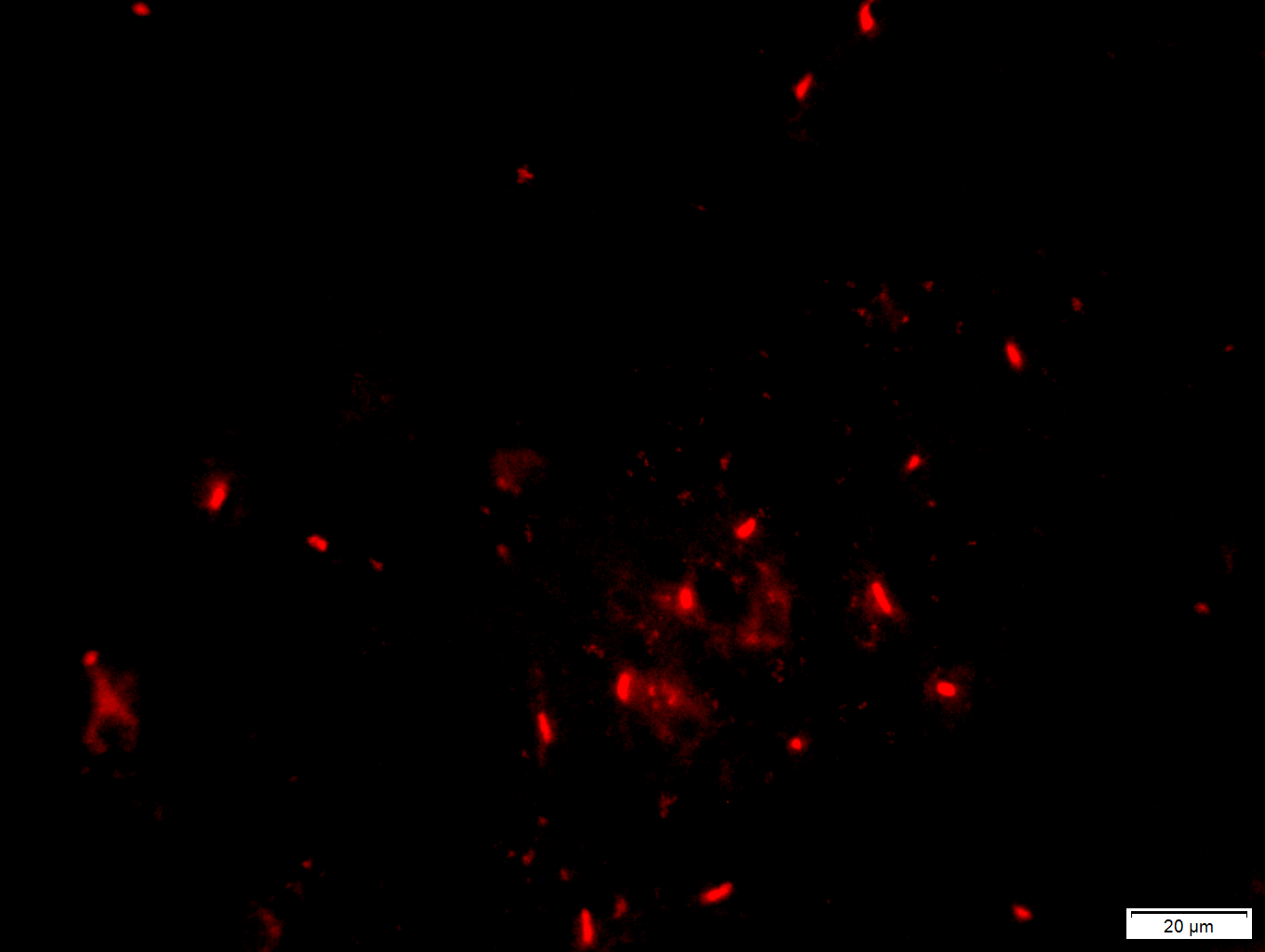

Supplement: Supplementary file 12 [file Data_Sheet_12.zip › Aβ immunofluorescence/CA3/3TG/M4/40X CA3.tif]

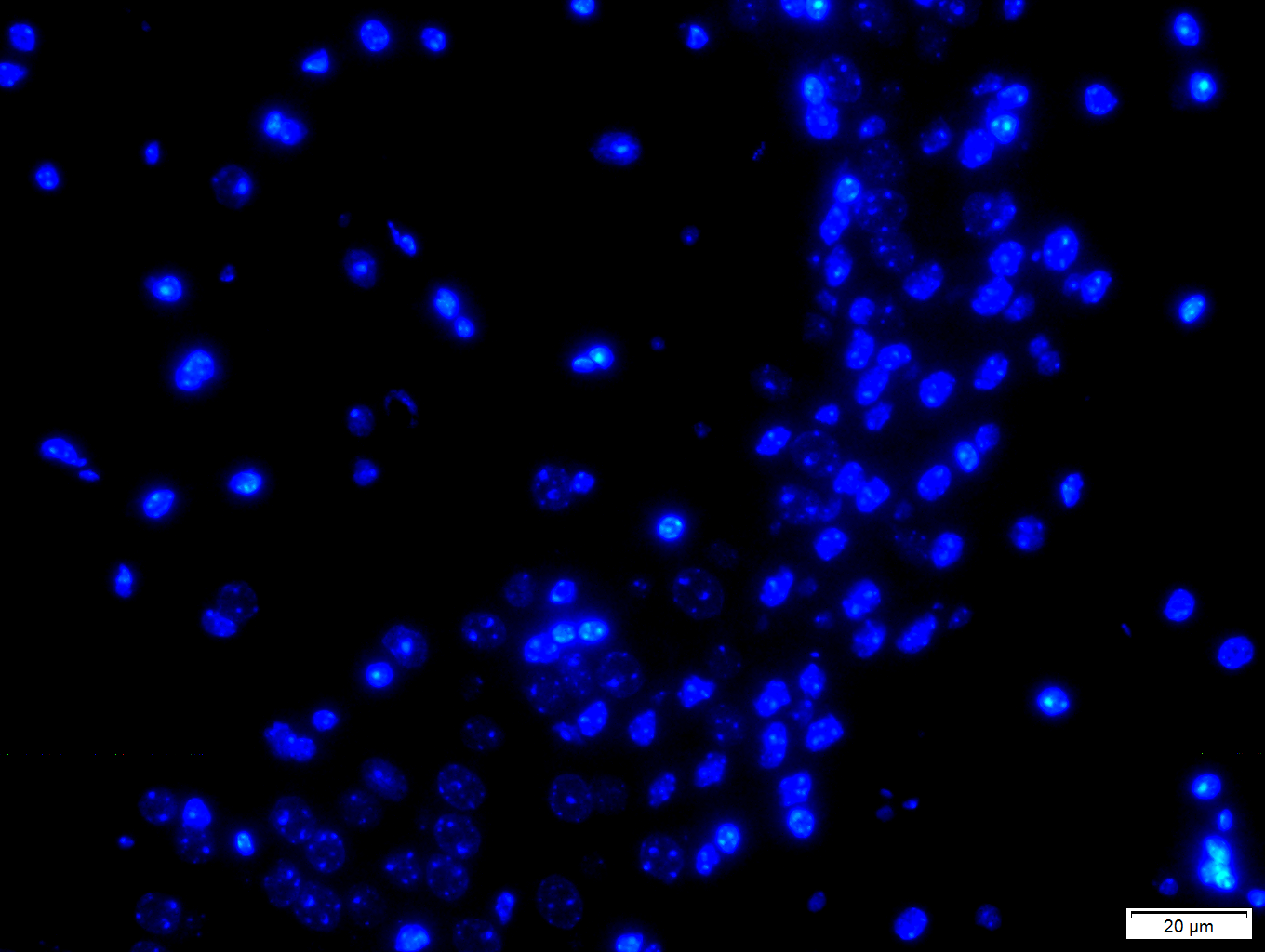

Supplement: Supplementary file 12 [file Data_Sheet_12.zip › Aβ immunofluorescence/CA3/3TG/M5/M5 CA3-1 DAPI.tif]

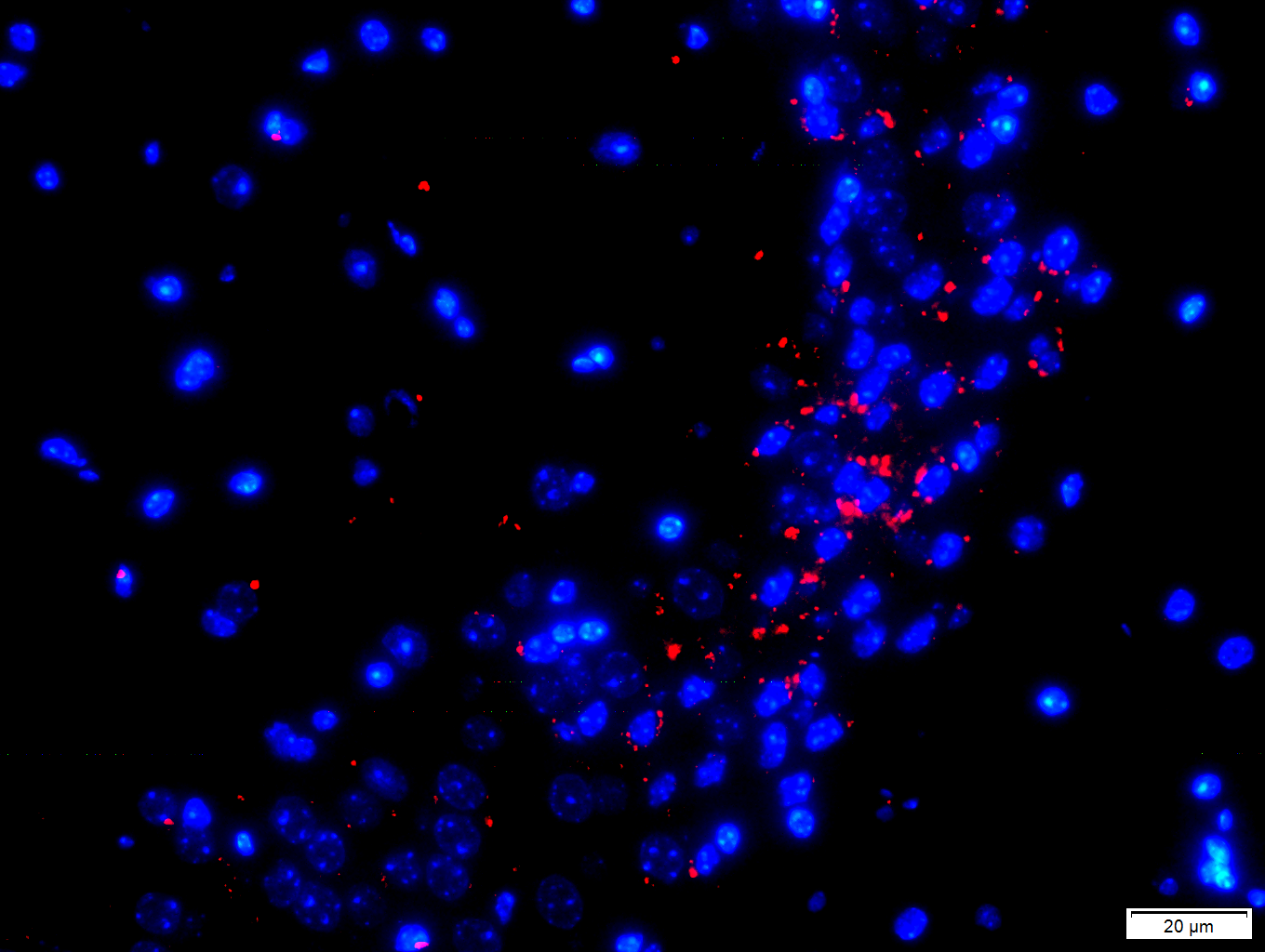

Supplement: Supplementary file 12 [file Data_Sheet_12.zip › Aβ immunofluorescence/CA3/3TG/M5/M5 CA3-1 merge.tif]

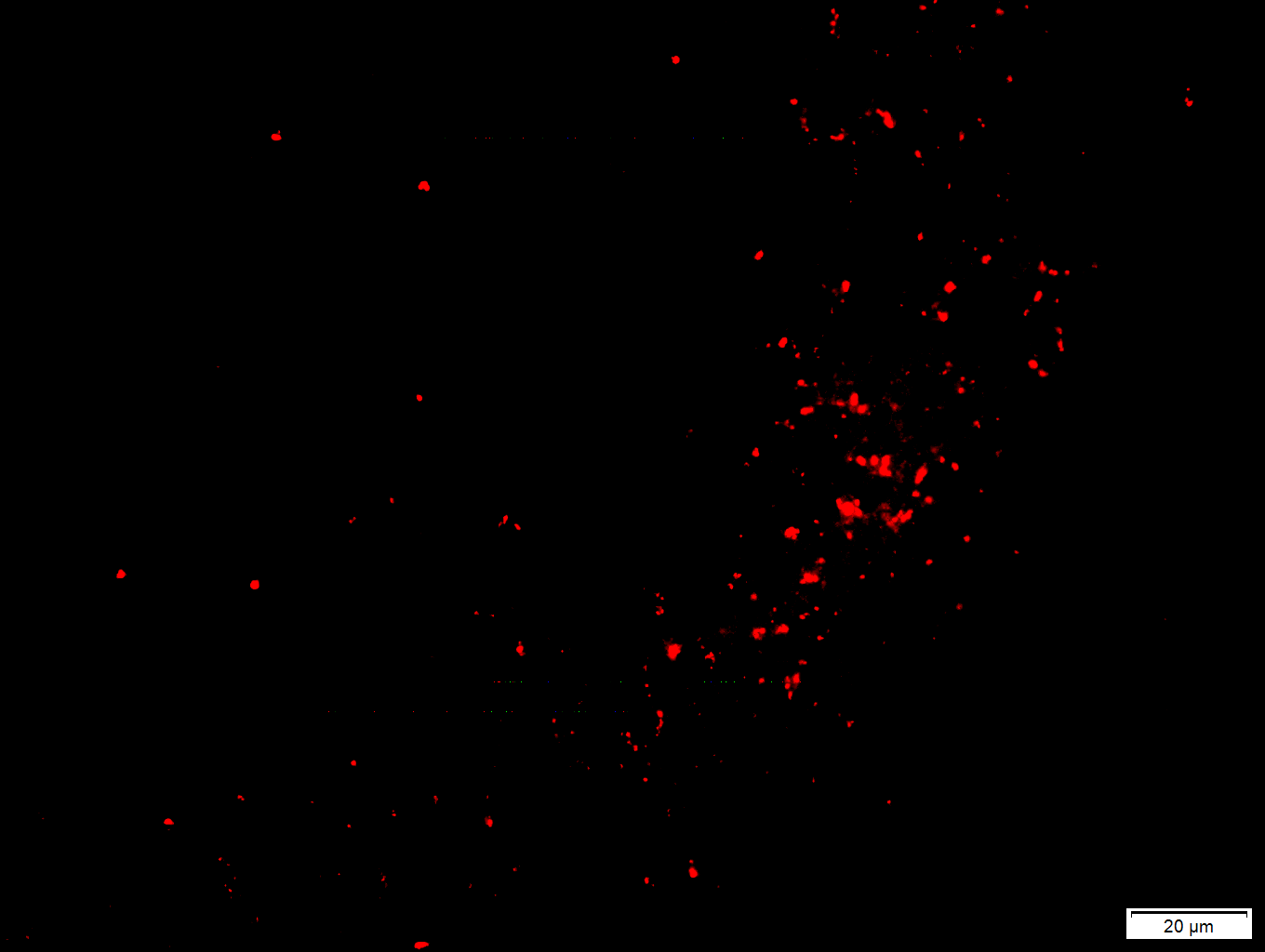

Supplement: Supplementary file 12 [file Data_Sheet_12.zip › Aβ immunofluorescence/CA3/3TG/M5/M5 CA3-1.tif]

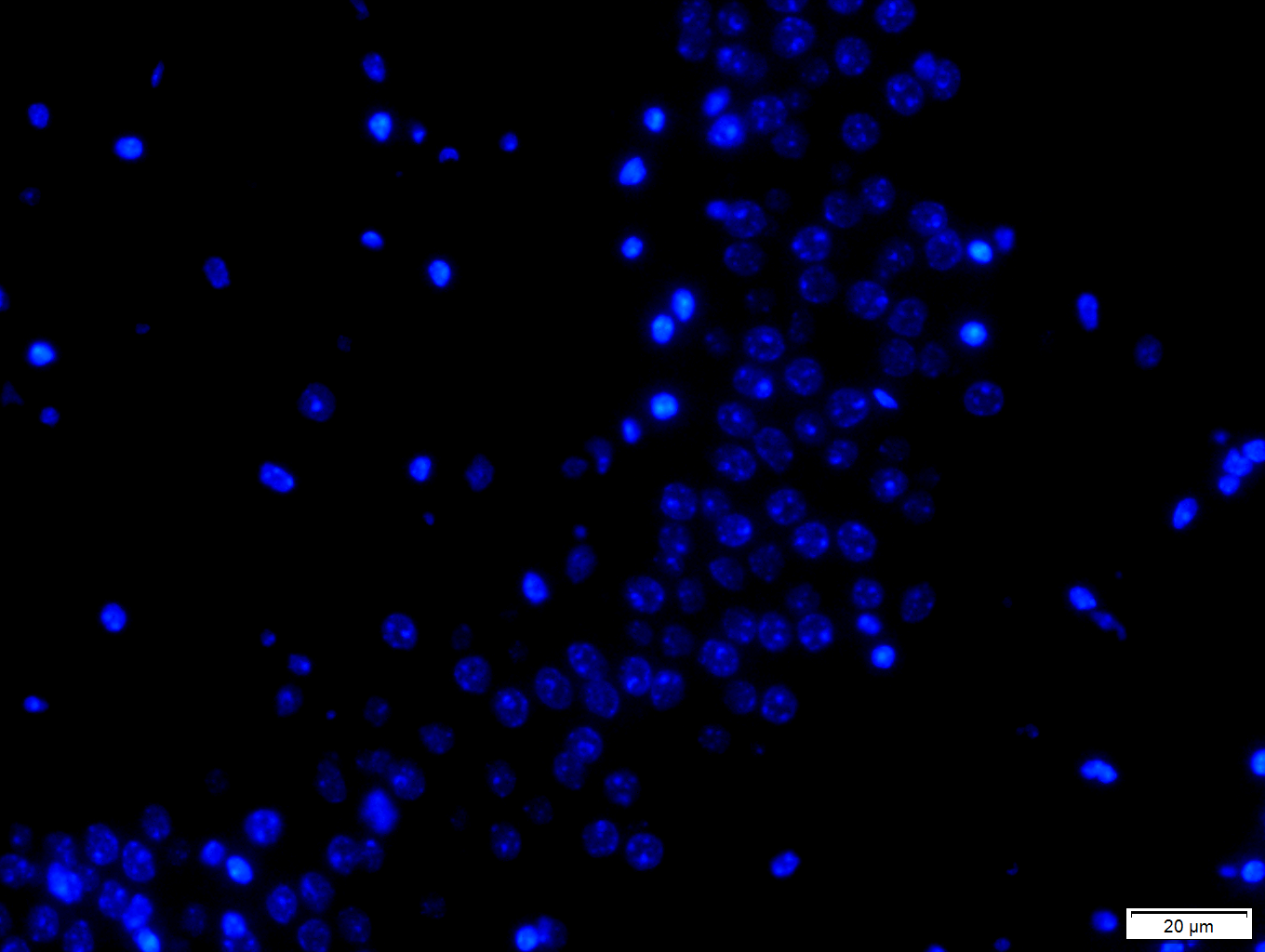

Supplement: Supplementary file 12 [file Data_Sheet_12.zip › Aβ immunofluorescence/CA3/WT/K11/k11 ca3 dapi.tif]

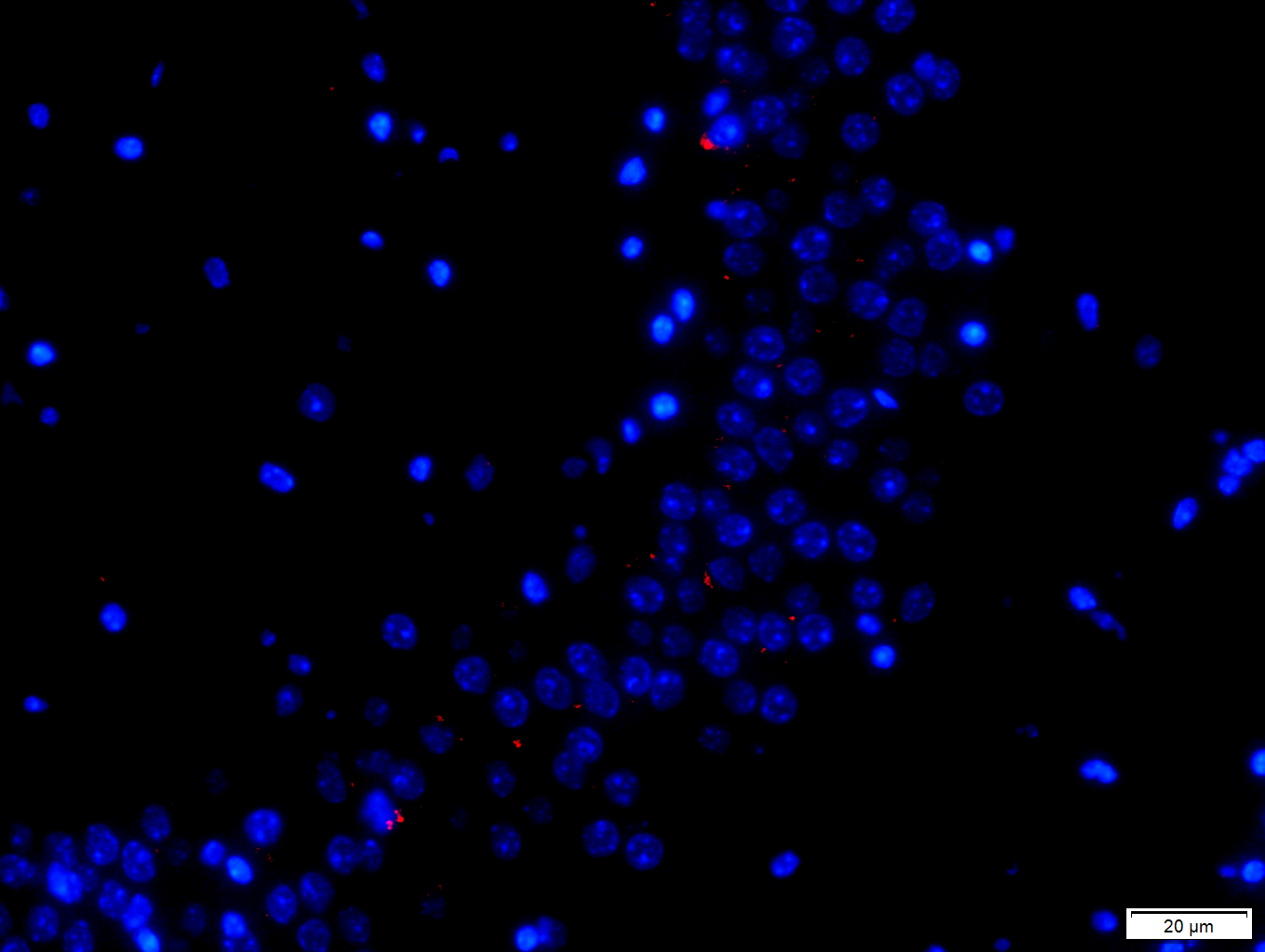

Supplement: Supplementary file 12 [file Data_Sheet_12.zip › Aβ immunofluorescence/CA3/WT/K11/k11 ca3 merge.tif]

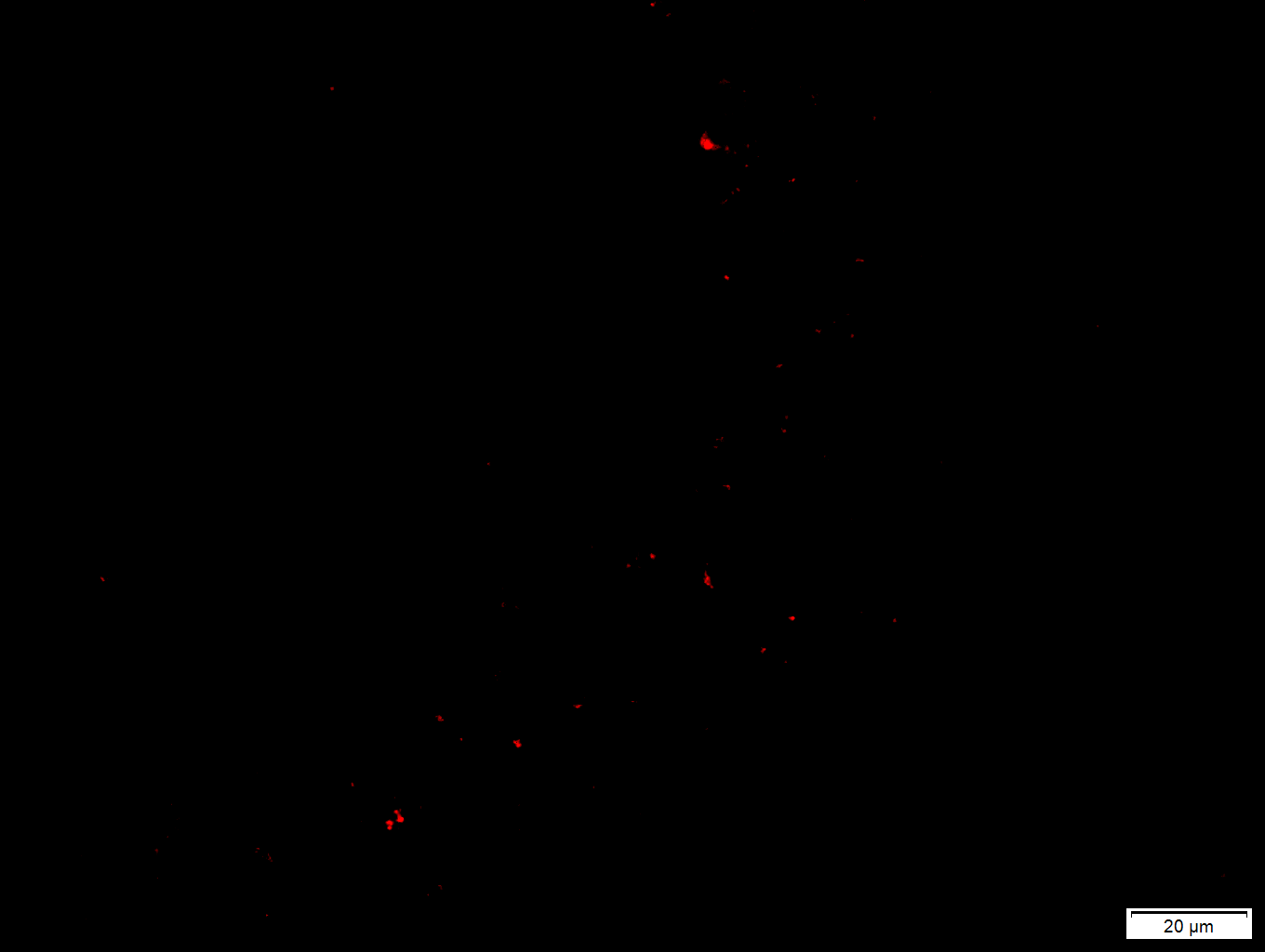

Supplement: Supplementary file 12 [file Data_Sheet_12.zip › Aβ immunofluorescence/CA3/WT/K11/k11 ca3.tif]

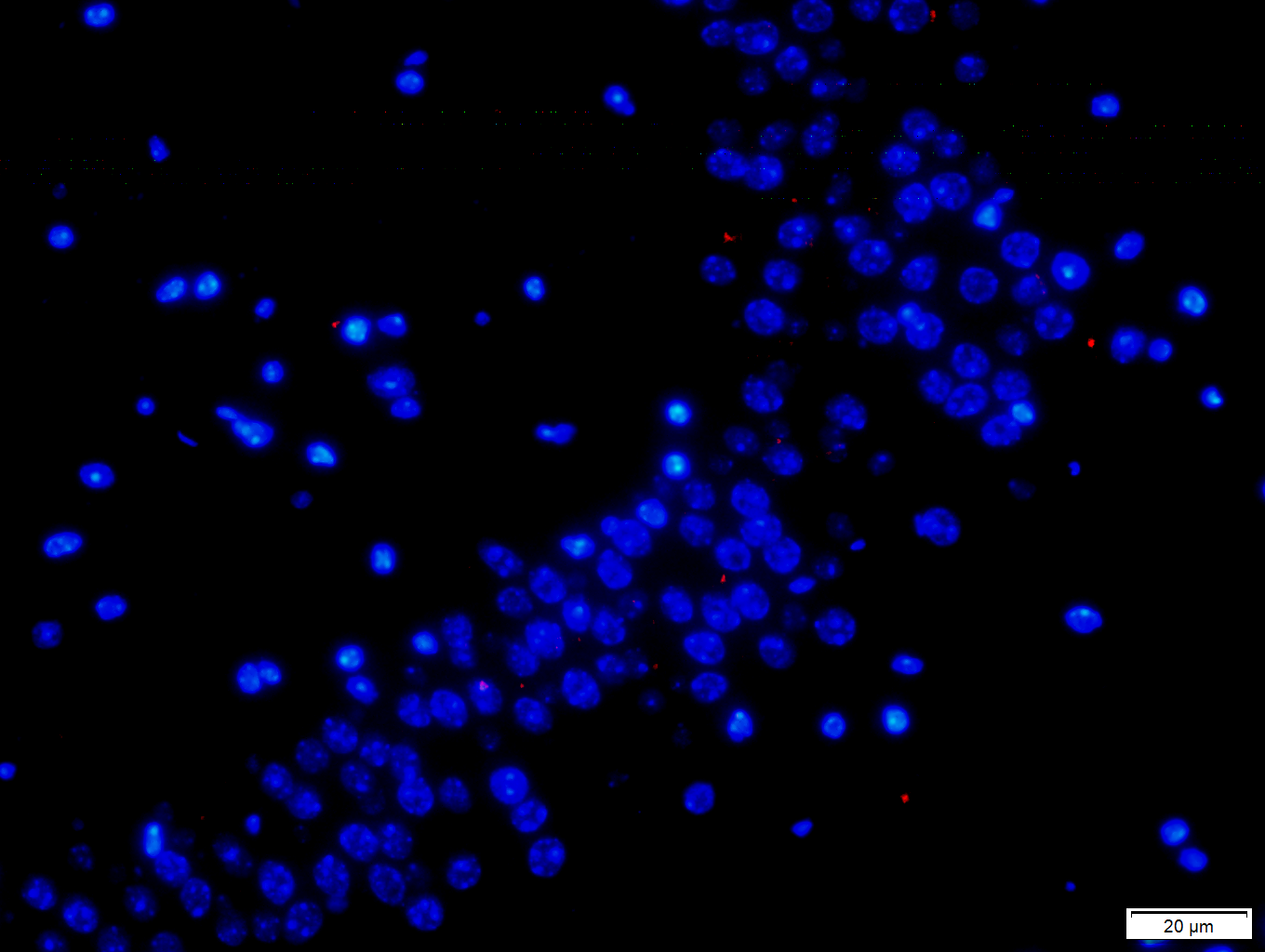

Supplement: Supplementary file 12 [file Data_Sheet_12.zip › Aβ immunofluorescence/CA3/WT/K12/K12 CA3 MERGE.tif]

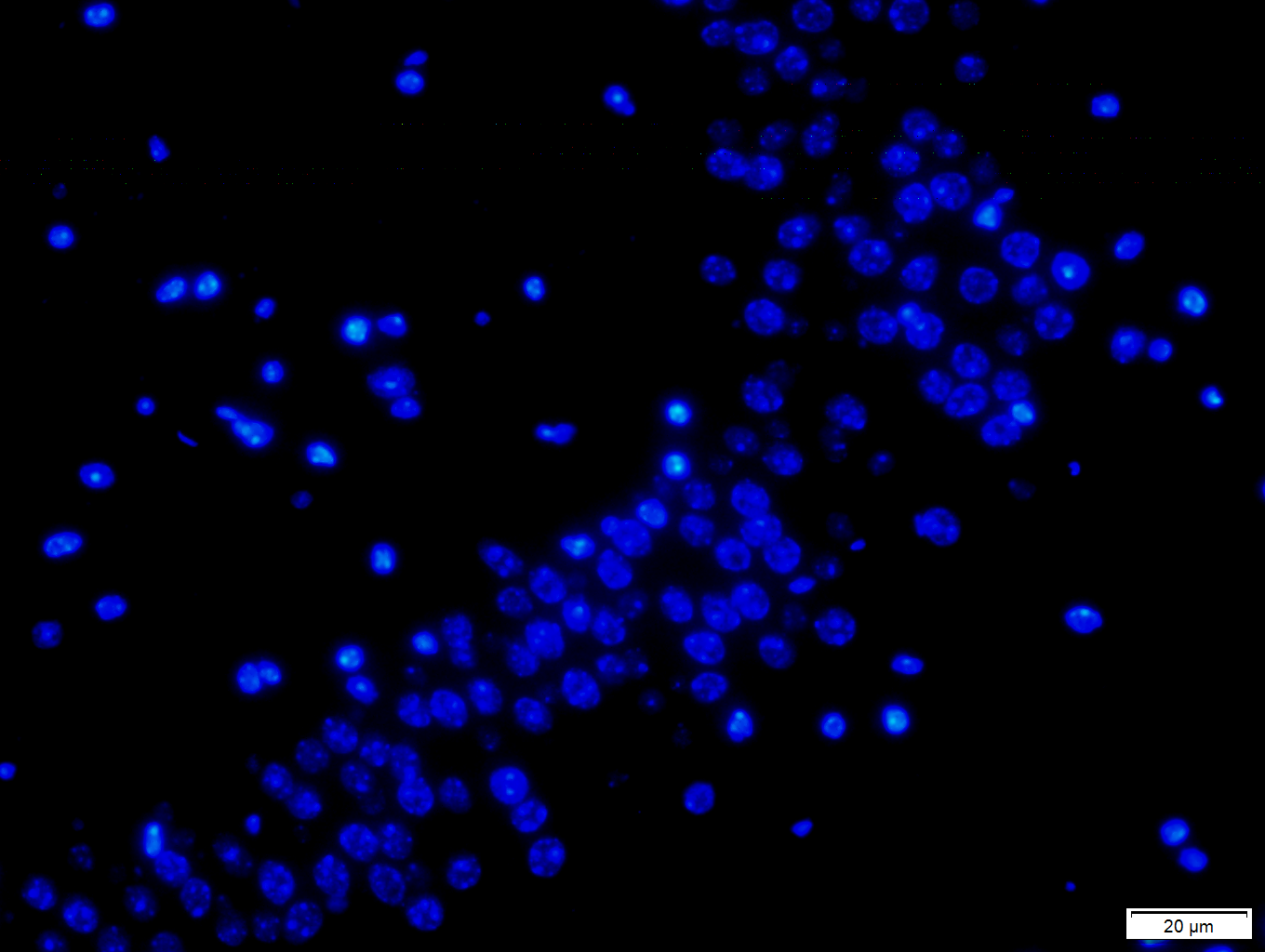

Supplement: Supplementary file 12 [file Data_Sheet_12.zip › Aβ immunofluorescence/CA3/WT/K12/K12 CA3 PAPI.tif]

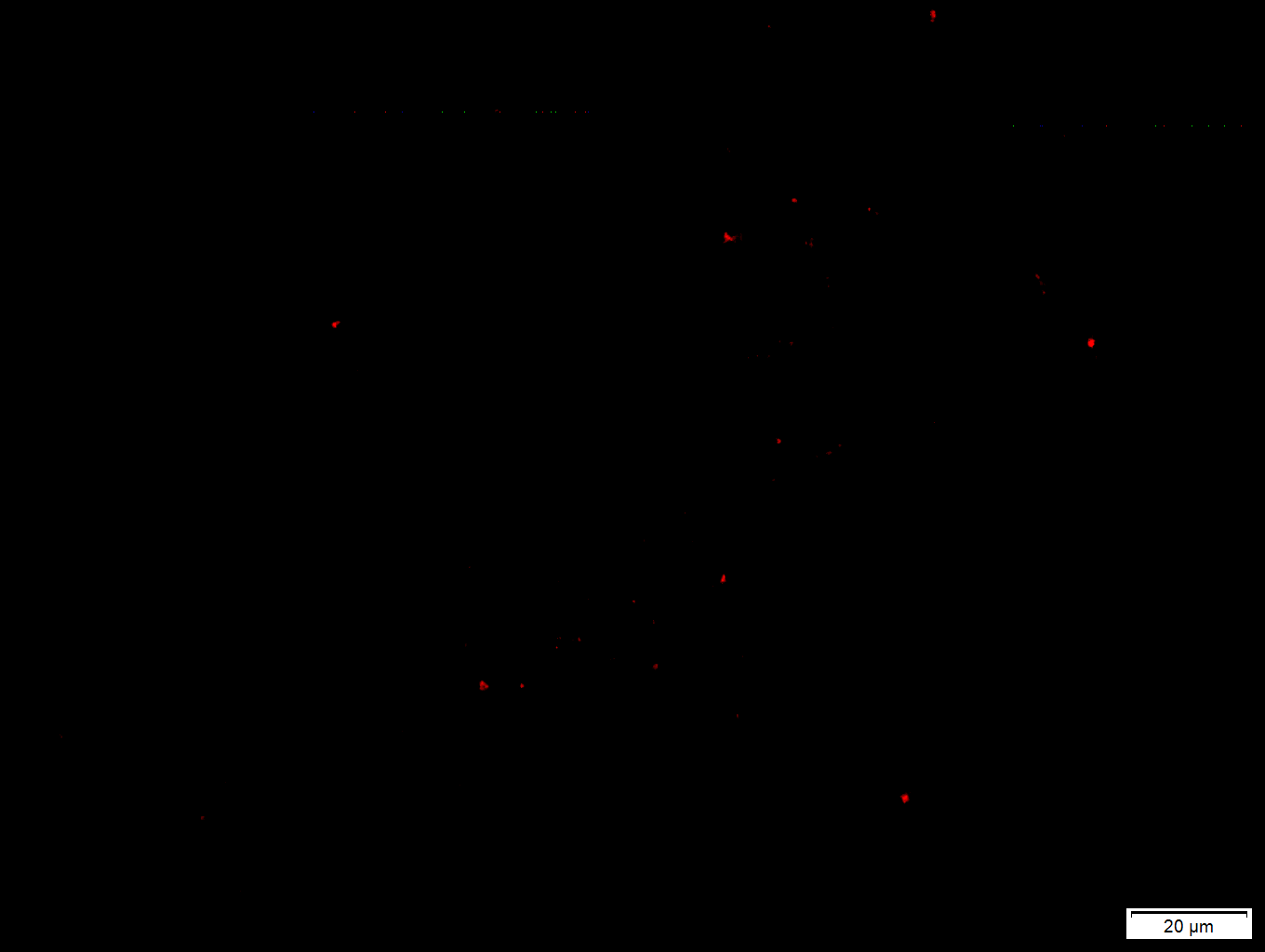

Supplement: Supplementary file 12 [file Data_Sheet_12.zip › Aβ immunofluorescence/CA3/WT/K12/K12 CA3.tif]

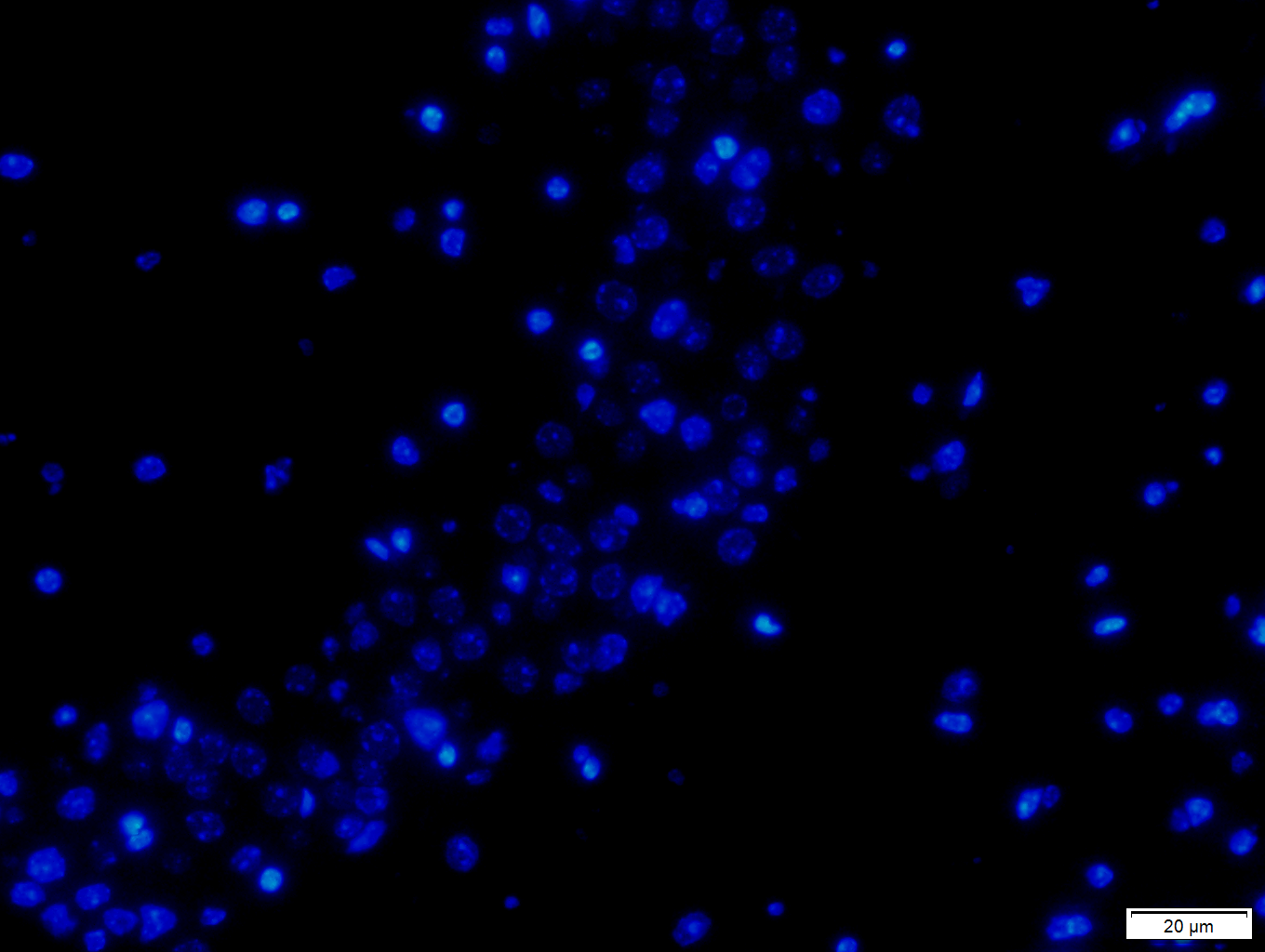

Supplement: Supplementary file 12 [file Data_Sheet_12.zip › Aβ immunofluorescence/CA3/WT/K13/k13 ca3 dapi.tif]

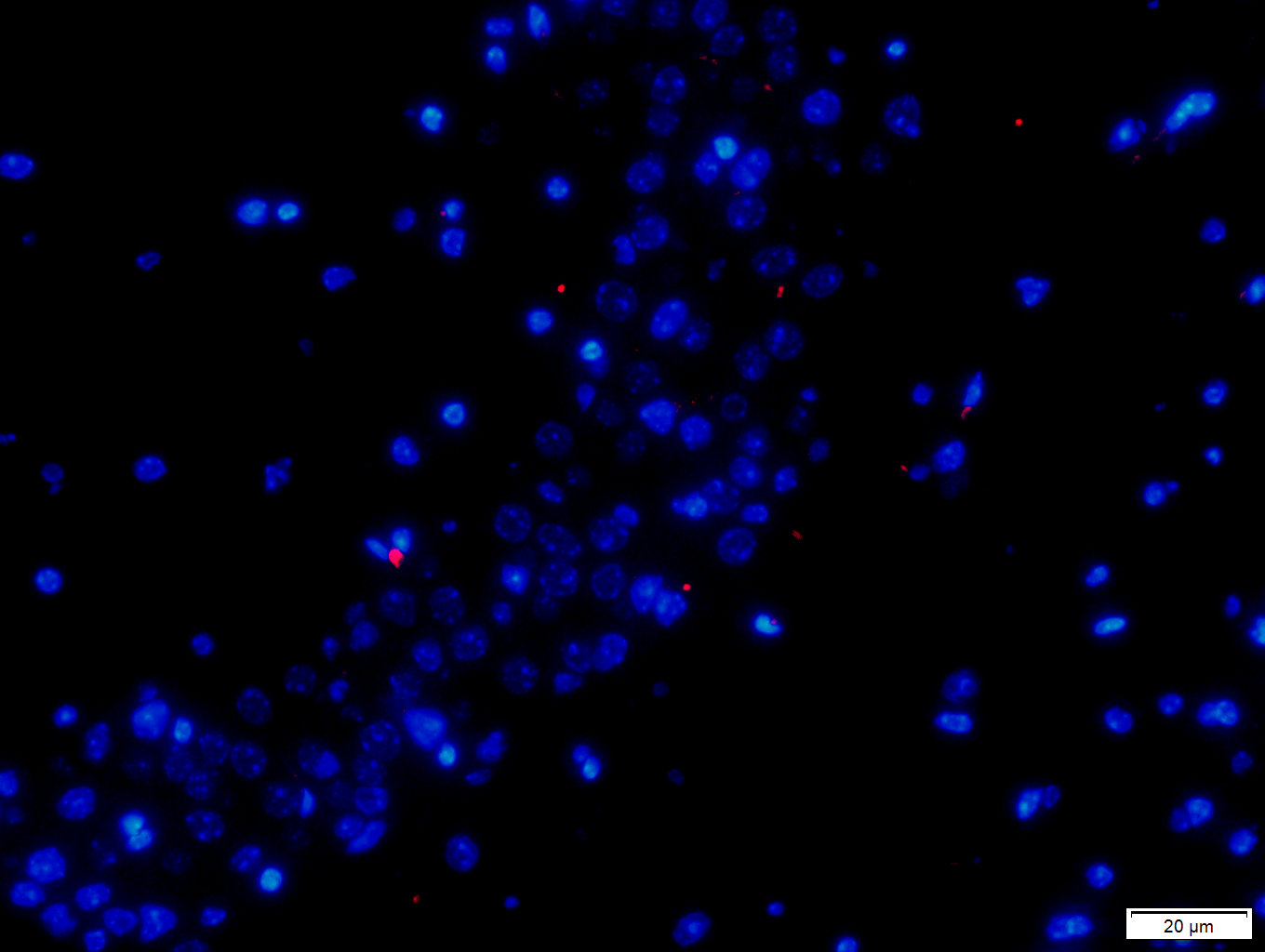

Supplement: Supplementary file 12 [file Data_Sheet_12.zip › Aβ immunofluorescence/CA3/WT/K13/k13 ca3 merge.tif]

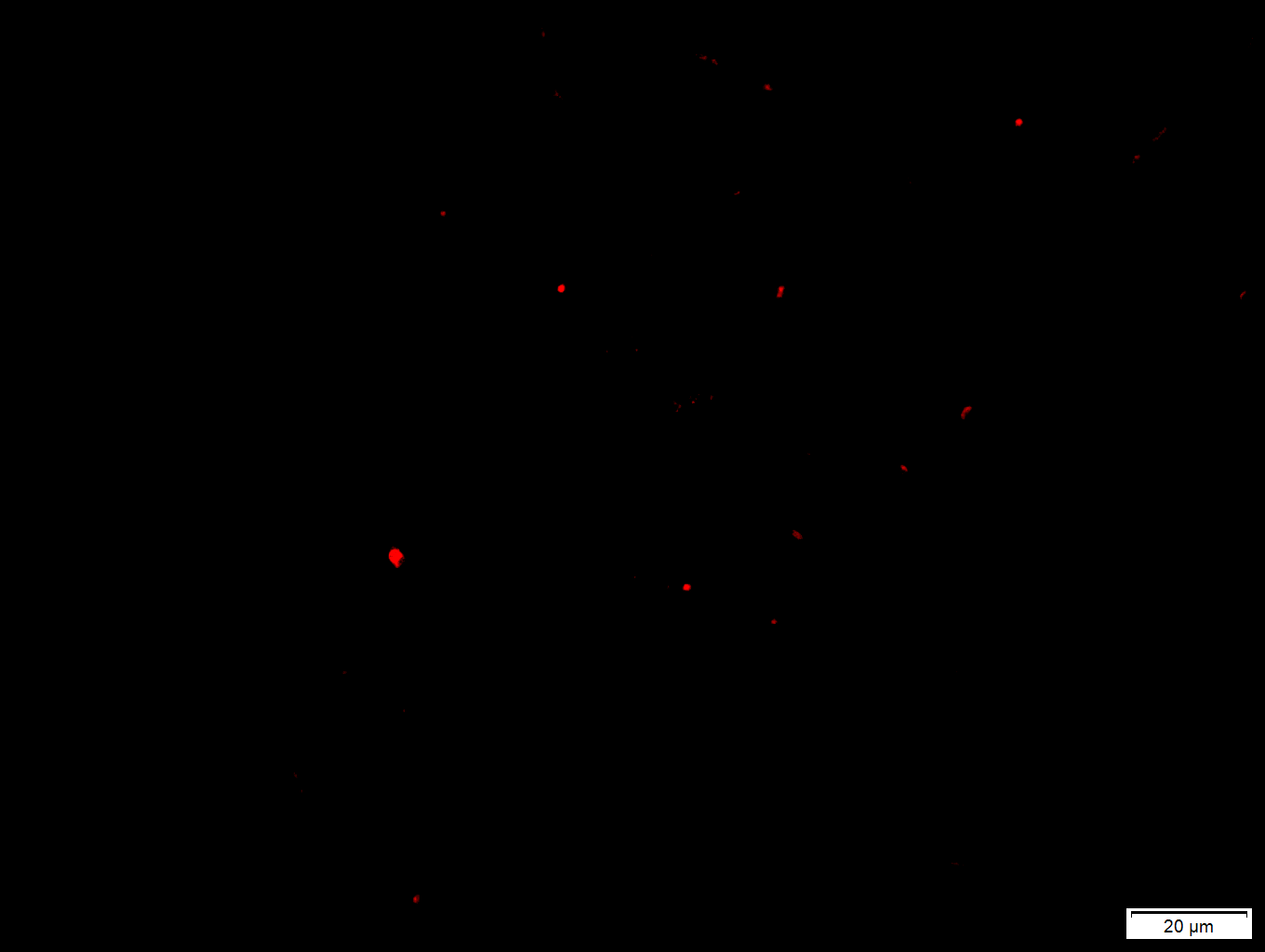

Supplement: Supplementary file 12 [file Data_Sheet_12.zip › Aβ immunofluorescence/CA3/WT/K13/k13 ca3.tif]

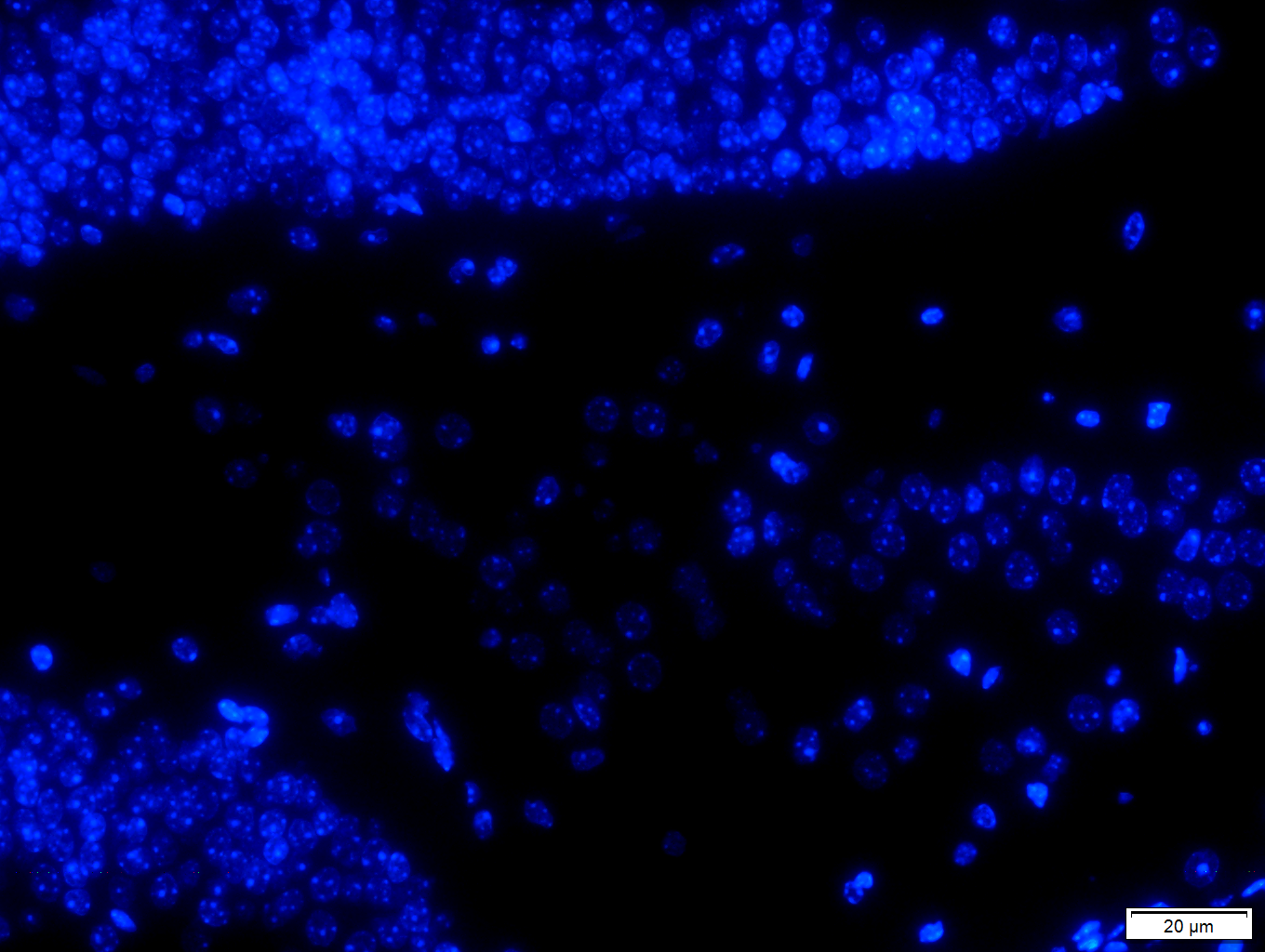

Supplement: Supplementary file 12 [file Data_Sheet_12.zip › Aβ immunofluorescence/DG/3TG/M1/M01 DG DAPI-2.tif]

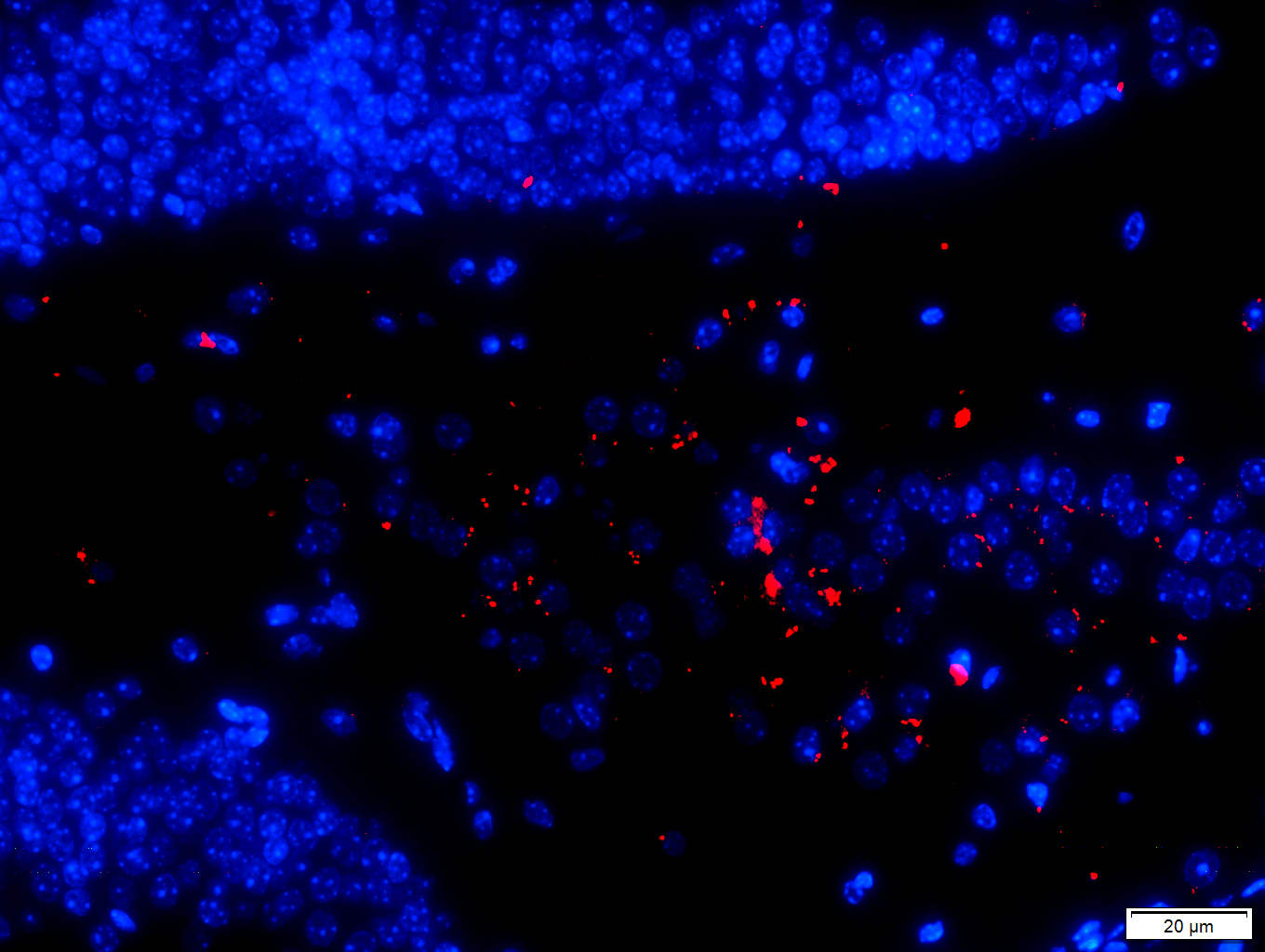

Supplement: Supplementary file 12 [file Data_Sheet_12.zip › Aβ immunofluorescence/DG/3TG/M1/M01 DG-2 merge.tif]

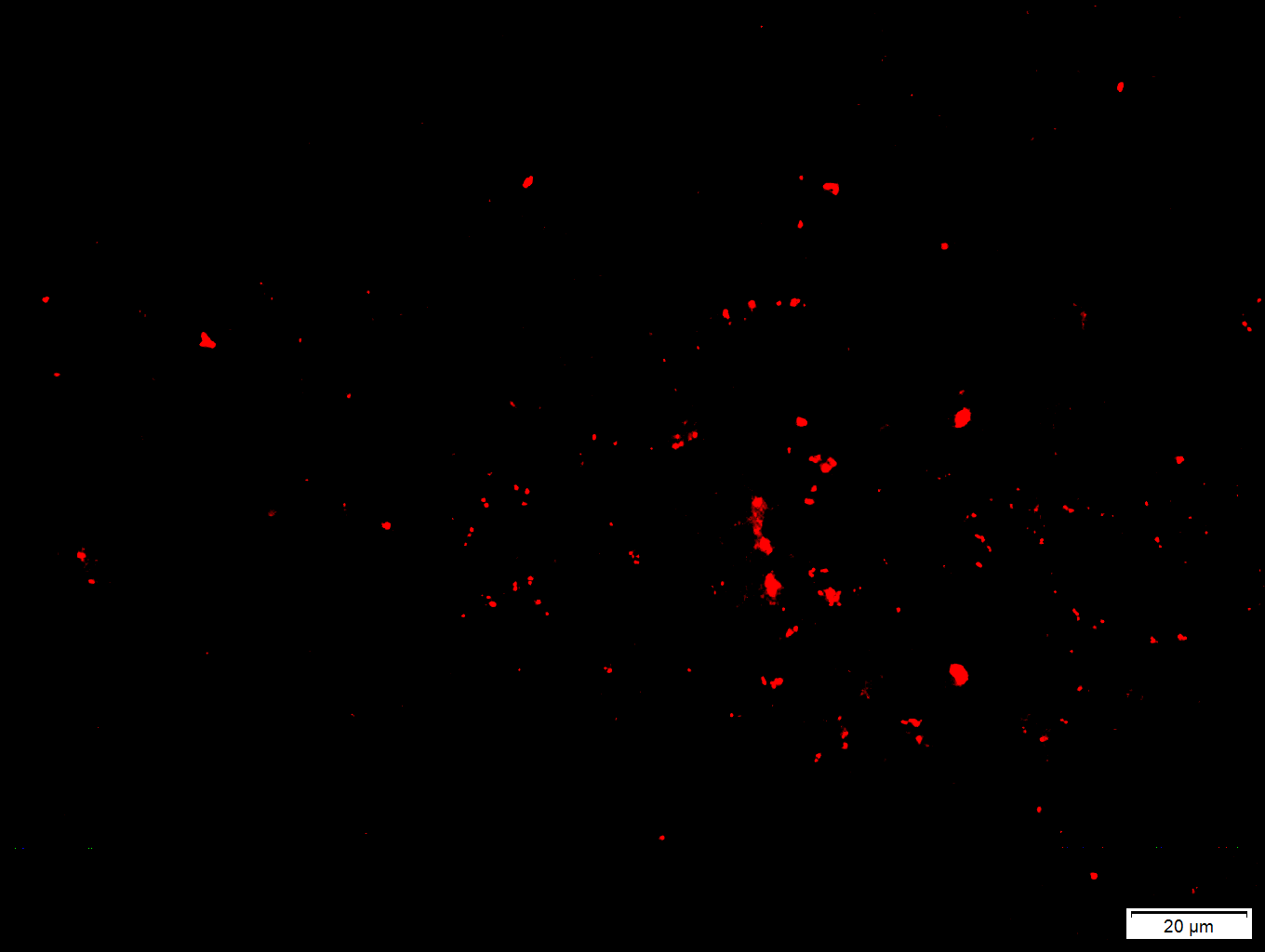

Supplement: Supplementary file 12 [file Data_Sheet_12.zip › Aβ immunofluorescence/DG/3TG/M1/M01 DG-2.tif]

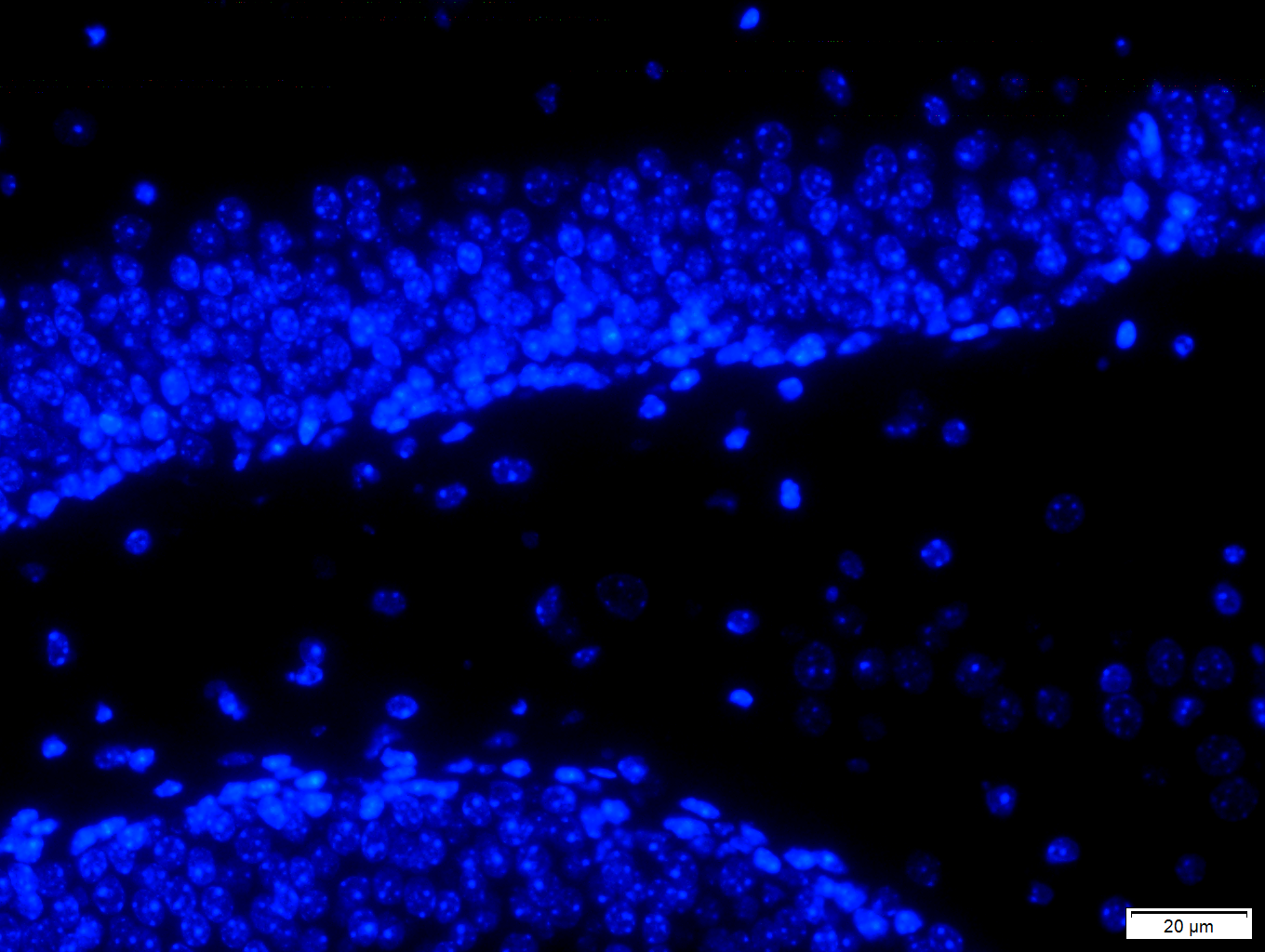

Supplement: Supplementary file 12 [file Data_Sheet_12.zip › Aβ immunofluorescence/DG/3TG/M5/M5 DG-1 DAPI.tif]

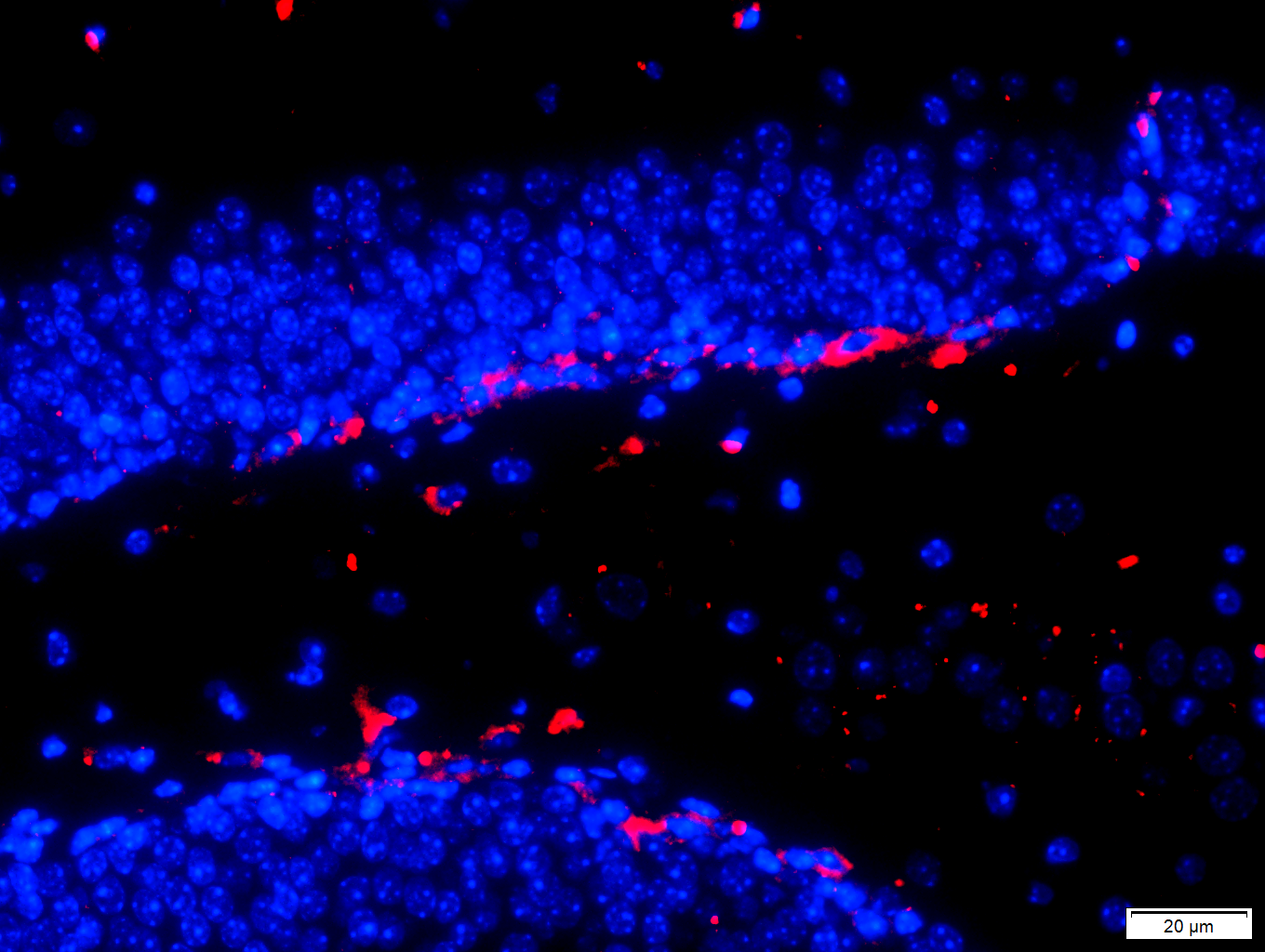

Supplement: Supplementary file 12 [file Data_Sheet_12.zip › Aβ immunofluorescence/DG/3TG/M5/M5 DG-1 merge.tif]

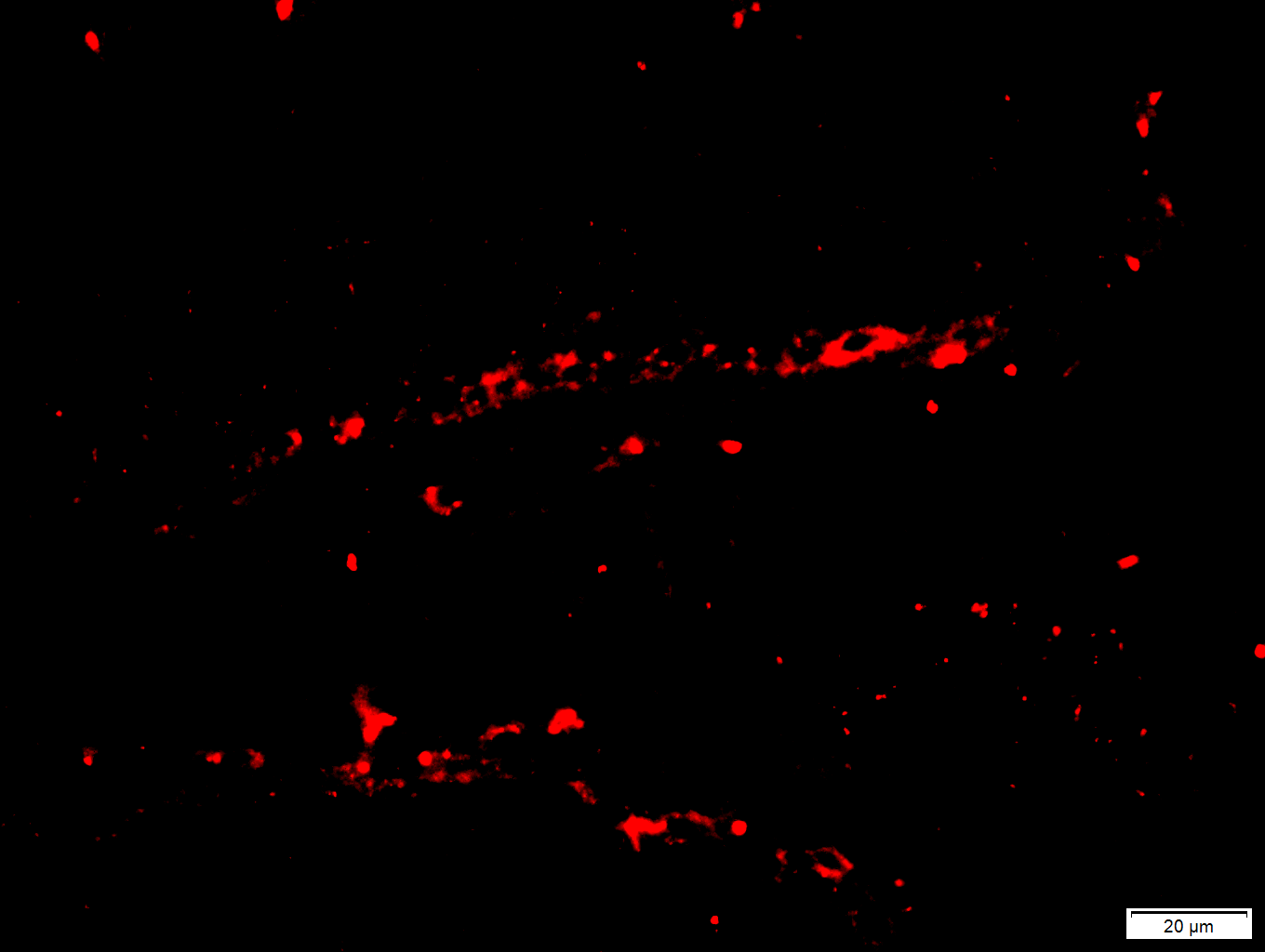

Supplement: Supplementary file 12 [file Data_Sheet_12.zip › Aβ immunofluorescence/DG/3TG/M5/M5 DG-1.tif]

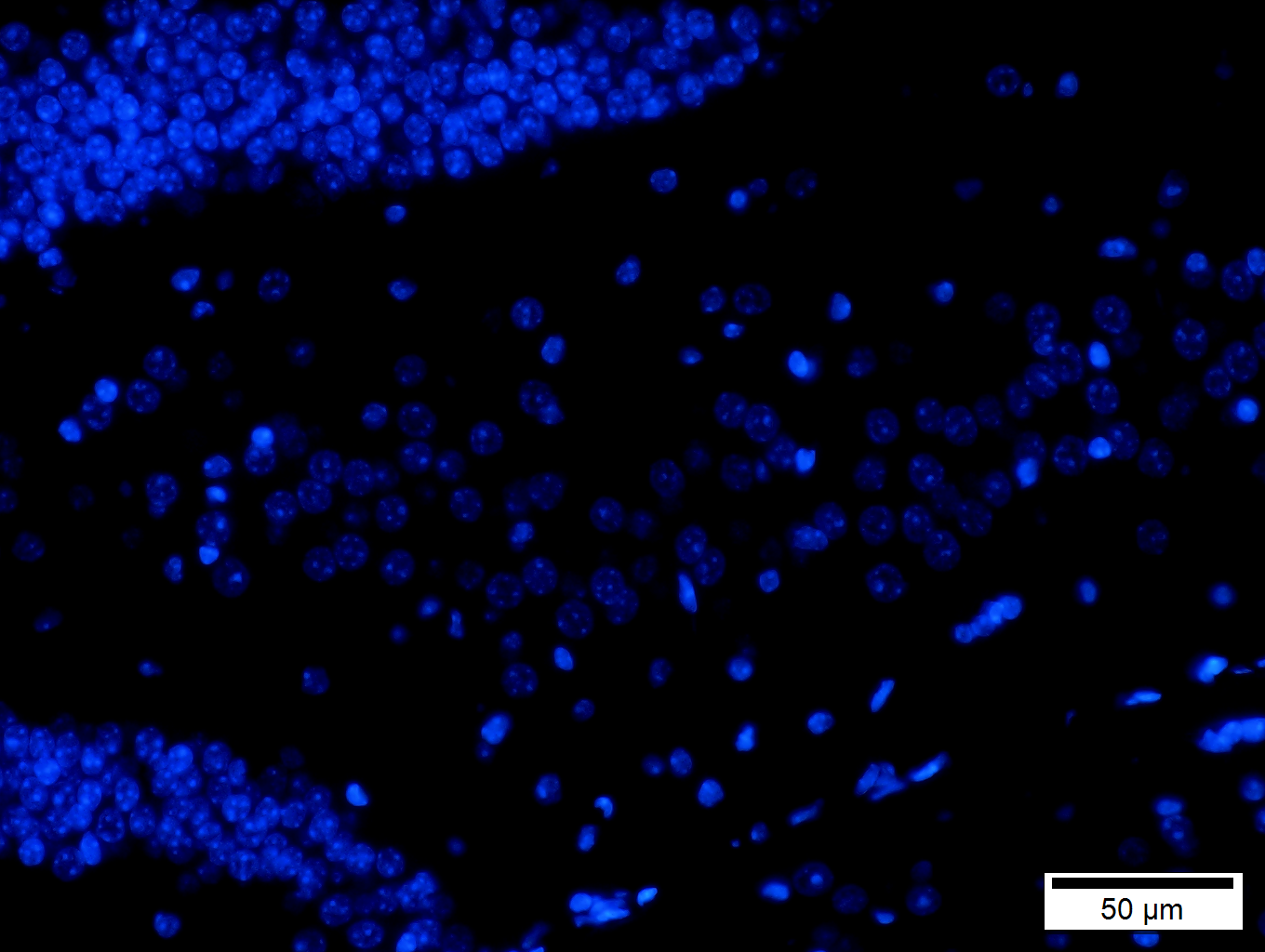

Supplement: Supplementary file 12 [file Data_Sheet_12.zip › Aβ immunofluorescence/DG/3TG/Y163/Y163 DG 40X-1.tif]

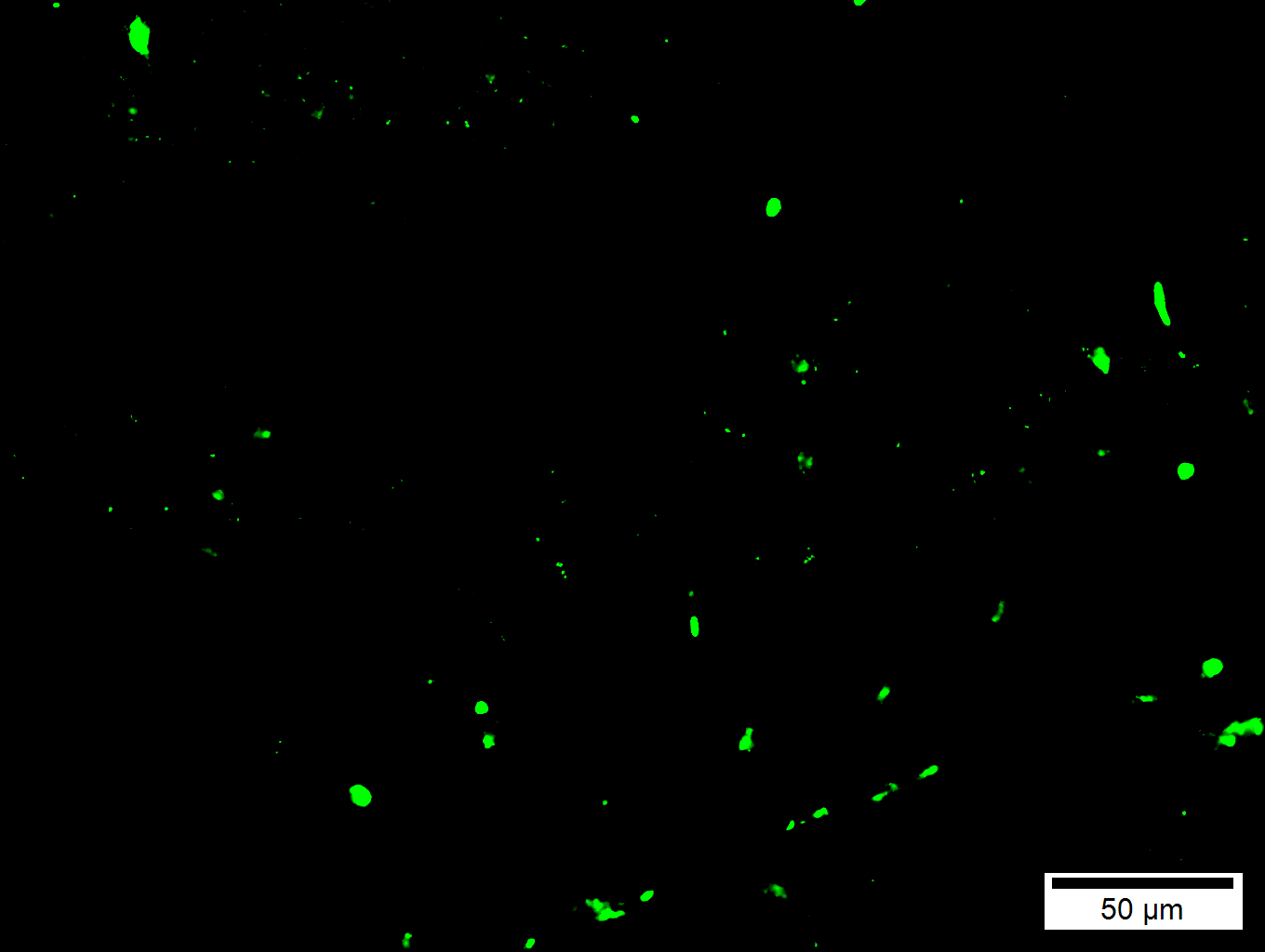

Supplement: Supplementary file 12 [file Data_Sheet_12.zip › Aβ immunofluorescence/DG/3TG/Y163/Y163 DG 40X-2.tif]

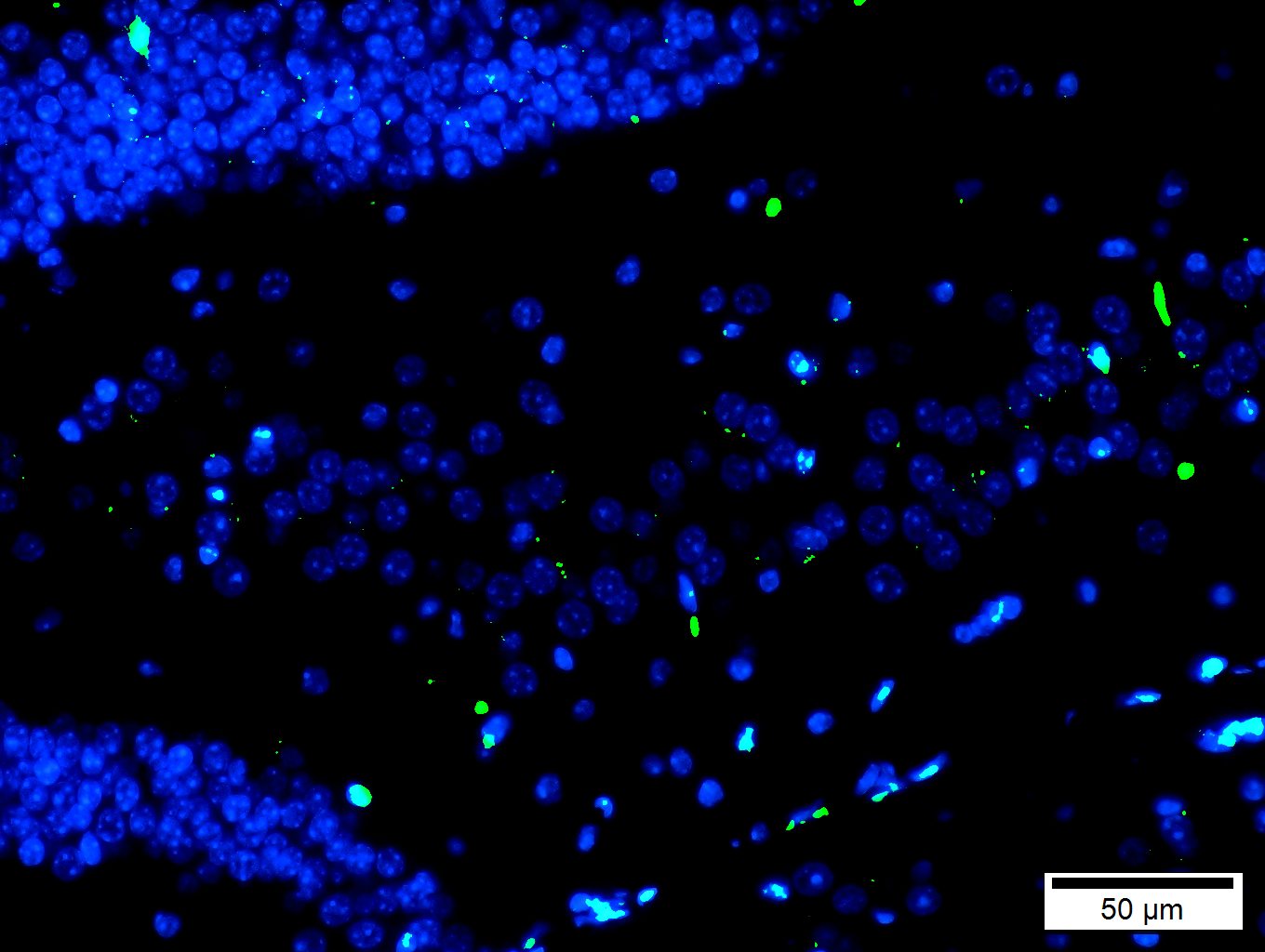

Supplement: Supplementary file 12 [file Data_Sheet_12.zip › Aβ immunofluorescence/DG/3TG/Y163/Y163 DG 40X-3.tif]

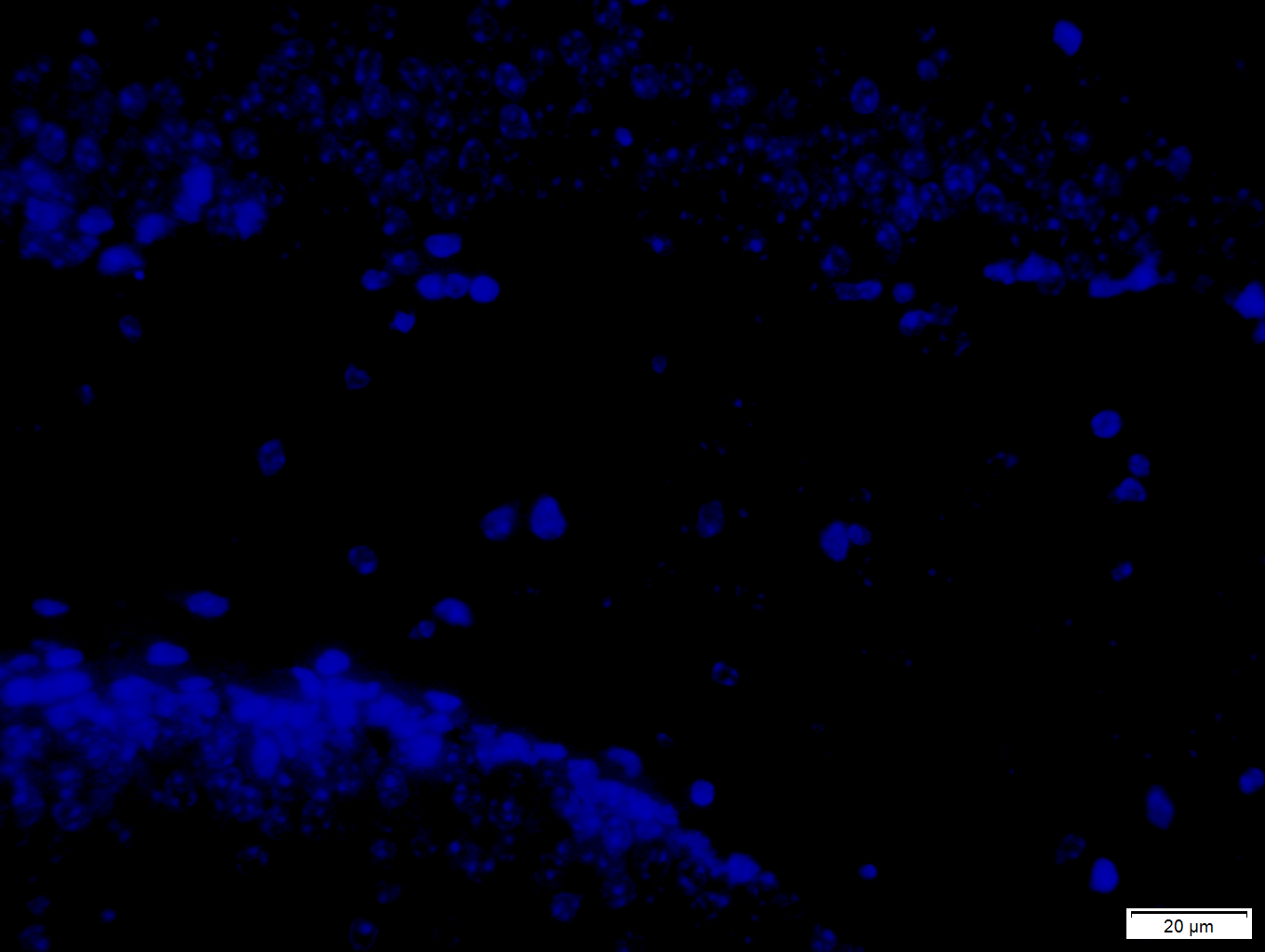

Supplement: Supplementary file 12 [file Data_Sheet_12.zip › Aβ immunofluorescence/DG/3TG/m4/40X DG DAPI.tif]

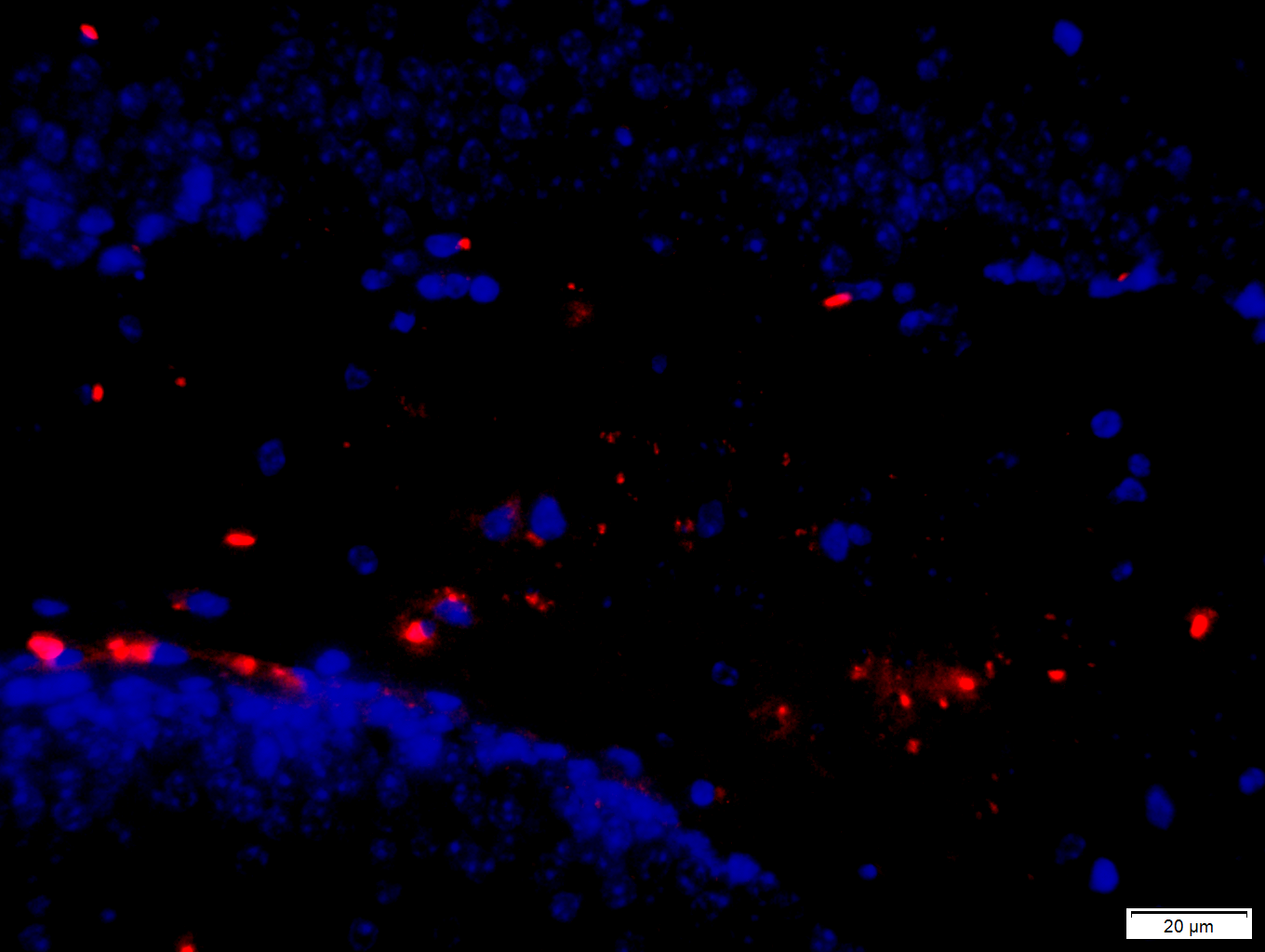

Supplement: Supplementary file 12 [file Data_Sheet_12.zip › Aβ immunofluorescence/DG/3TG/m4/40X DG merge.tif]

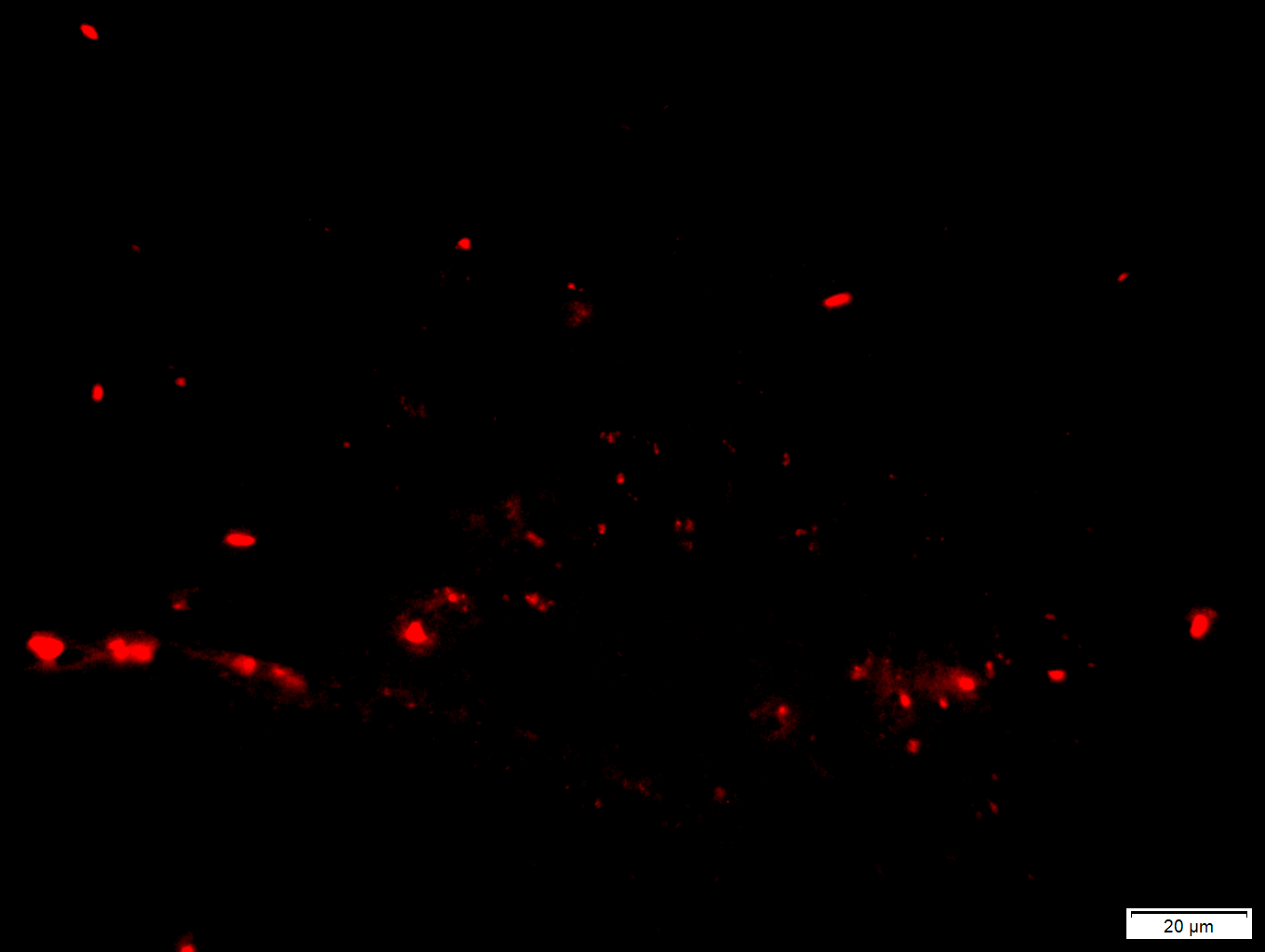

Supplement: Supplementary file 12 [file Data_Sheet_12.zip › Aβ immunofluorescence/DG/3TG/m4/40X DG.tif]

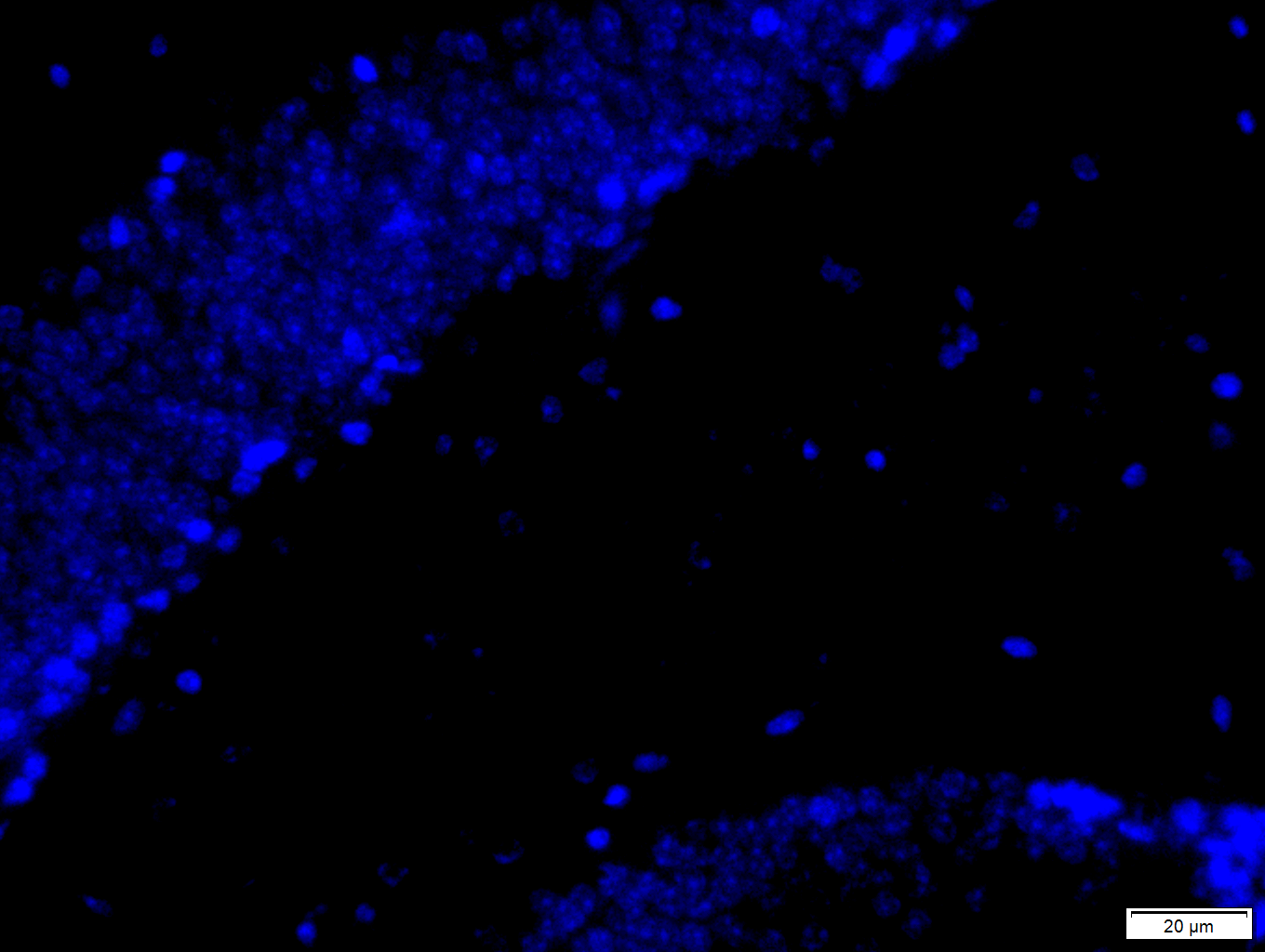

Supplement: Supplementary file 12 [file Data_Sheet_12.zip › Aβ immunofluorescence/DG/WT/K10/k10 dg dapi.tif]

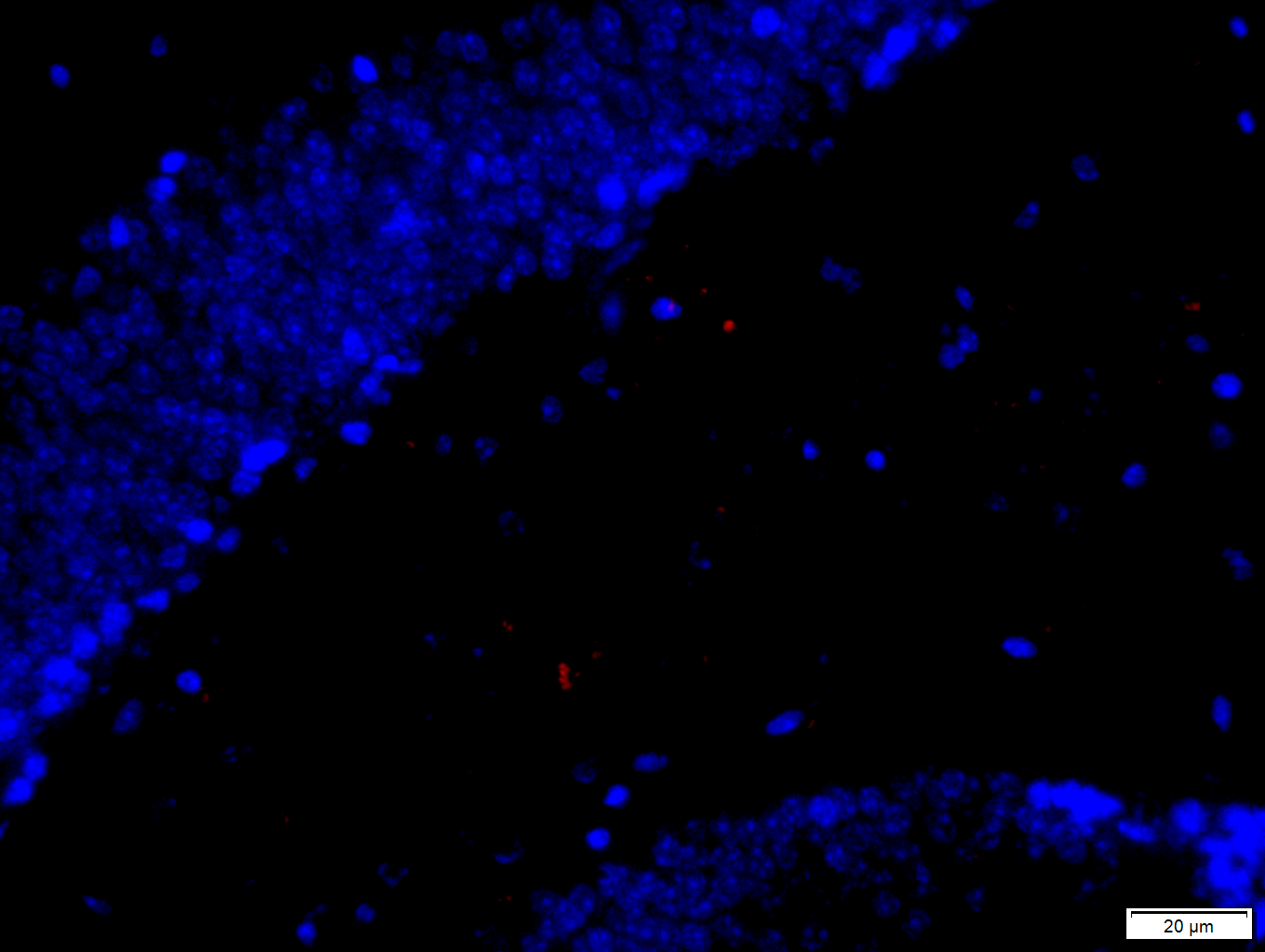

Supplement: Supplementary file 12 [file Data_Sheet_12.zip › Aβ immunofluorescence/DG/WT/K10/k10 dg merge.tif]

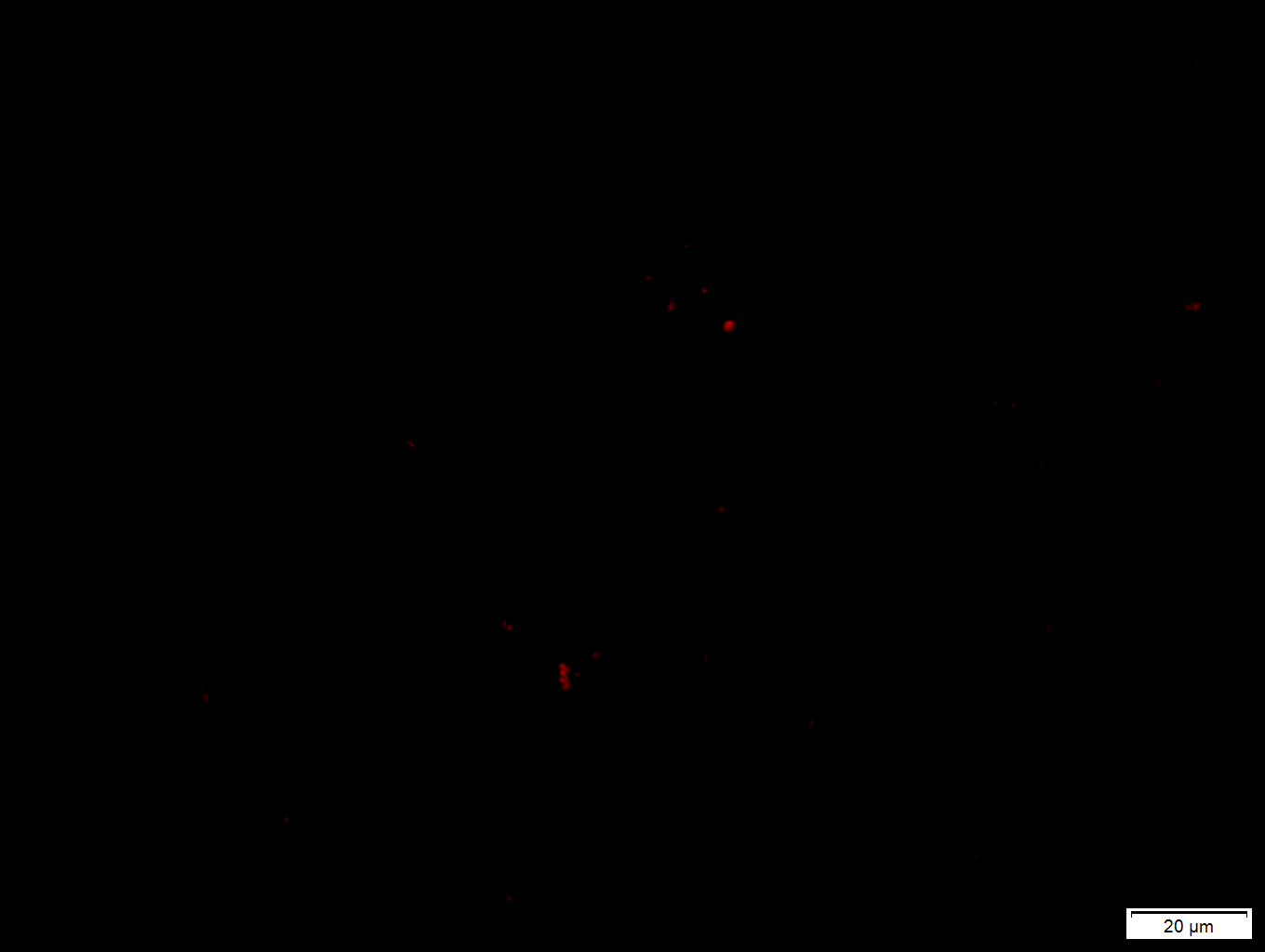

Supplement: Supplementary file 12 [file Data_Sheet_12.zip › Aβ immunofluorescence/DG/WT/K10/k10 dg.tif]

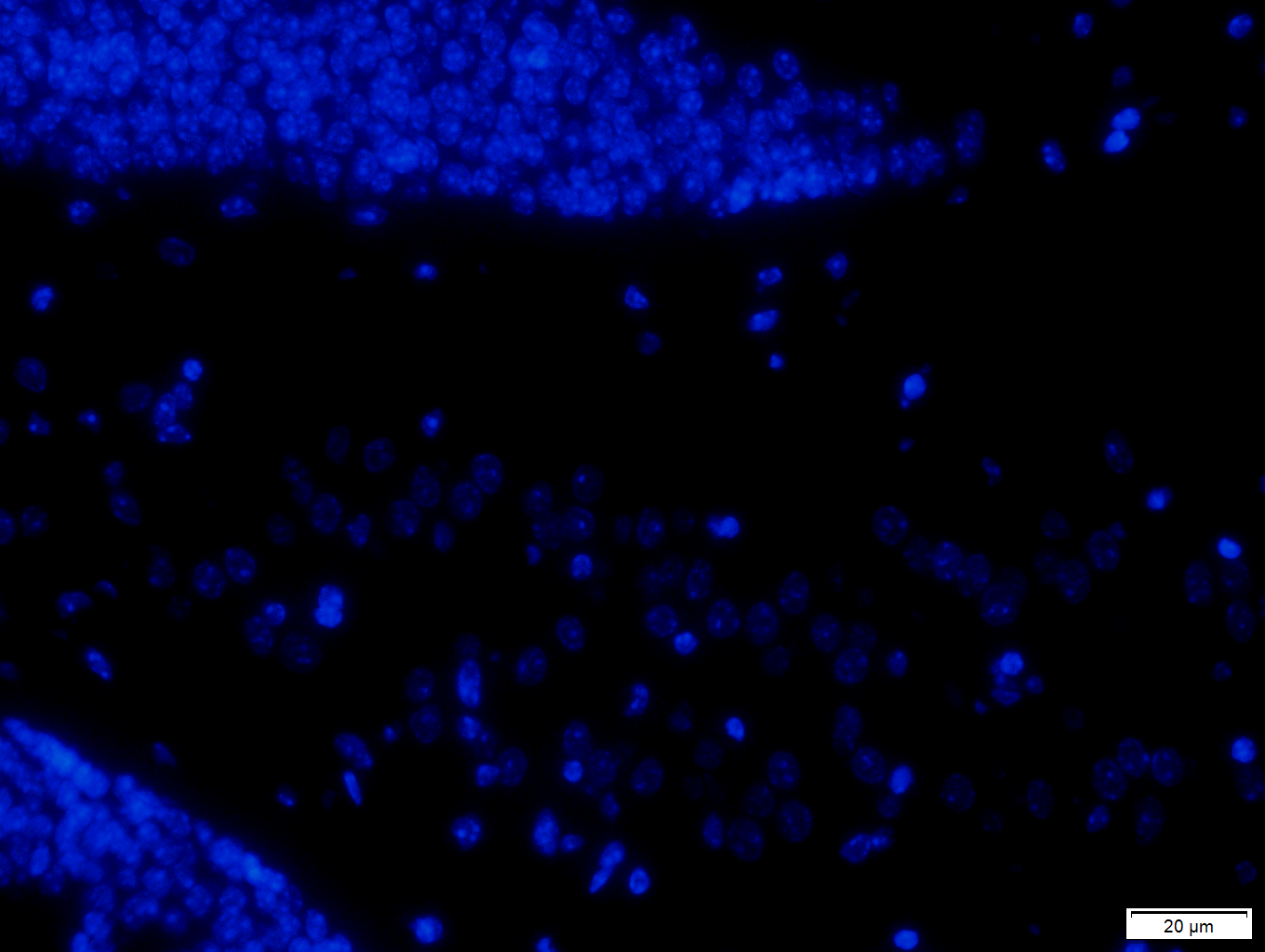

Supplement: Supplementary file 12 [file Data_Sheet_12.zip › Aβ immunofluorescence/DG/WT/K11/k11 dg dapi.tif]

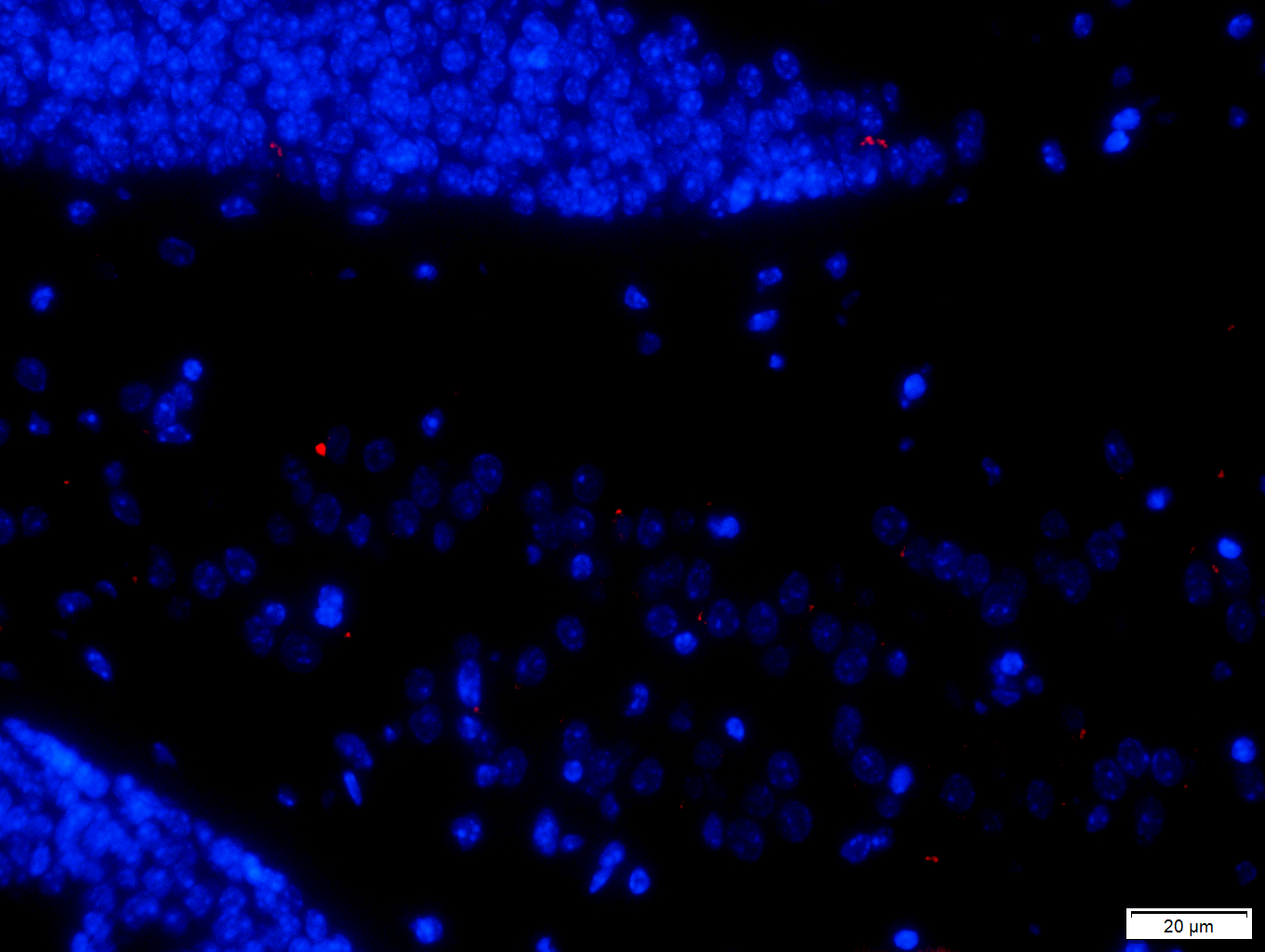

Supplement: Supplementary file 12 [file Data_Sheet_12.zip › Aβ immunofluorescence/DG/WT/K11/k11 dg merge.tif]

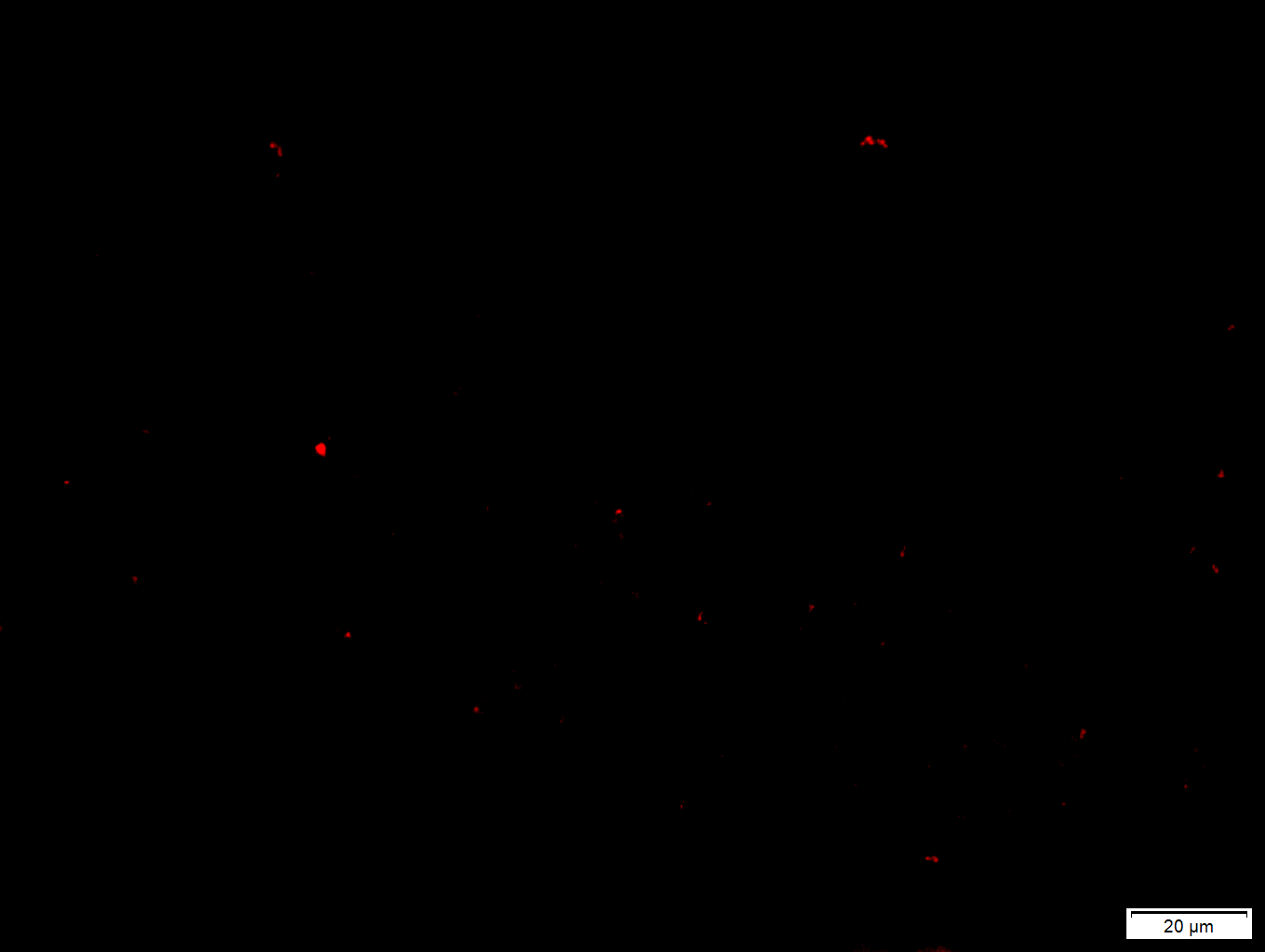

Supplement: Supplementary file 12 [file Data_Sheet_12.zip › Aβ immunofluorescence/DG/WT/K11/k11 dg.tif]

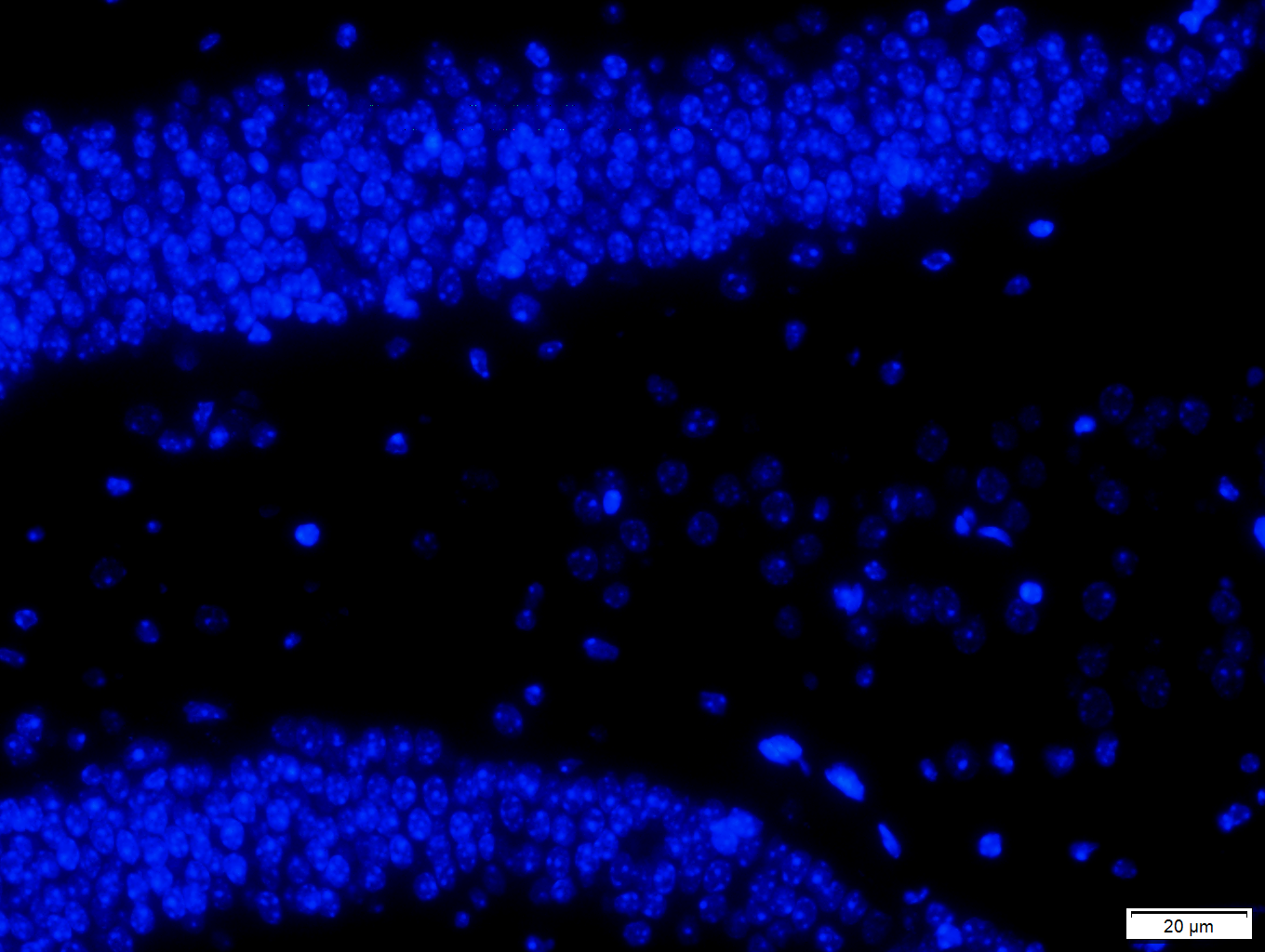

Supplement: Supplementary file 12 [file Data_Sheet_12.zip › Aβ immunofluorescence/DG/WT/K12/K12 DG DAPI.tif]
