# Supplementary figures and images for: Suppression of hnRNP A1 binding to HK1 RNA leads to glycolytic dysfunction in Alzheimer’s disease models (part 4 of 4)
Source: Front Aging Neurosci. 2023 Aug 31;15:1218267. doi: 10.3389/fnagi.2023.1218267 (PMC10516183; doi:10.3389/fnagi.2023.1218267)

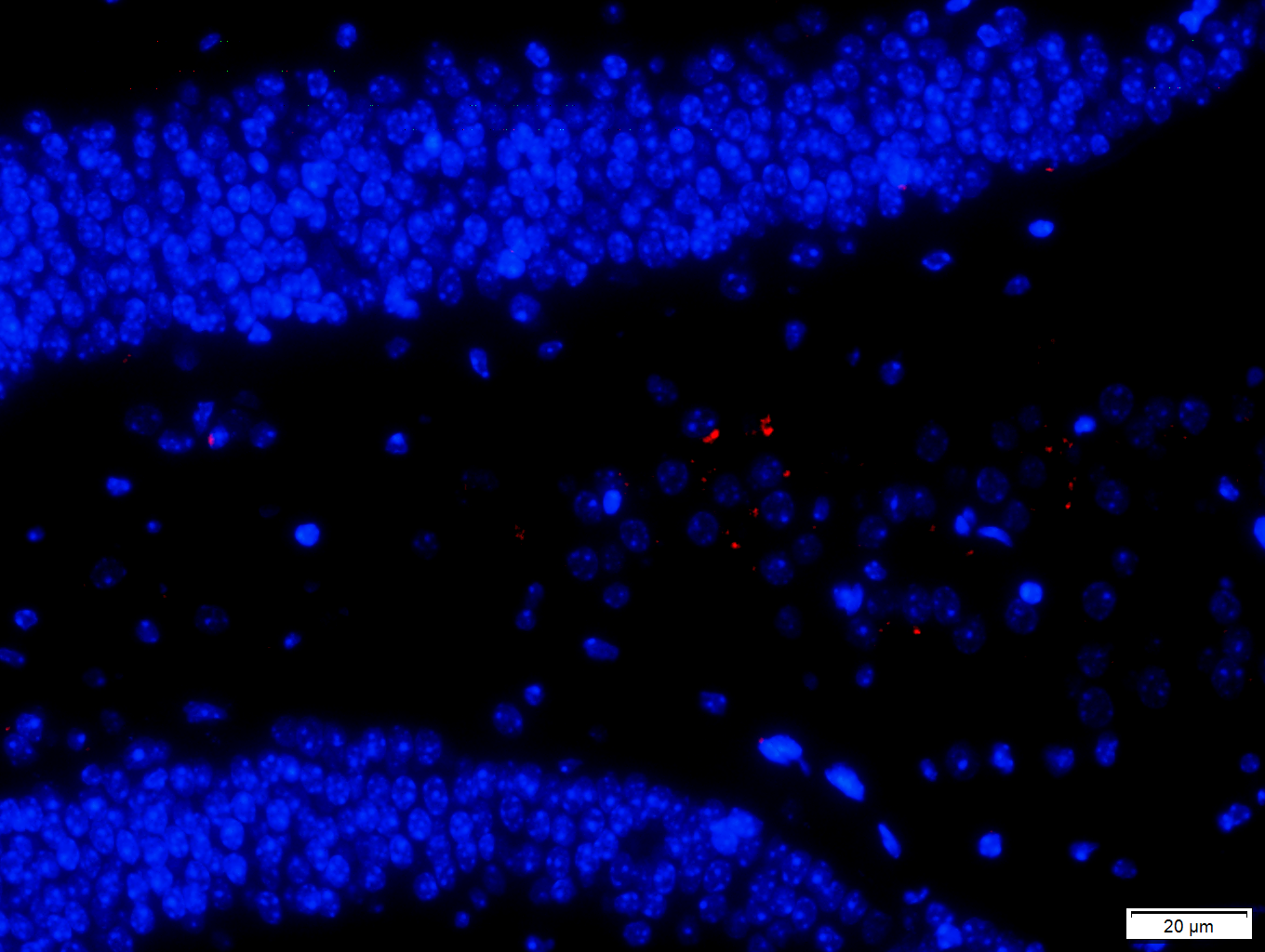

Supplement: Supplementary file 12 [file Data_Sheet_12.zip › Aβ immunofluorescence/DG/WT/K12/K12 DG merge.tif]

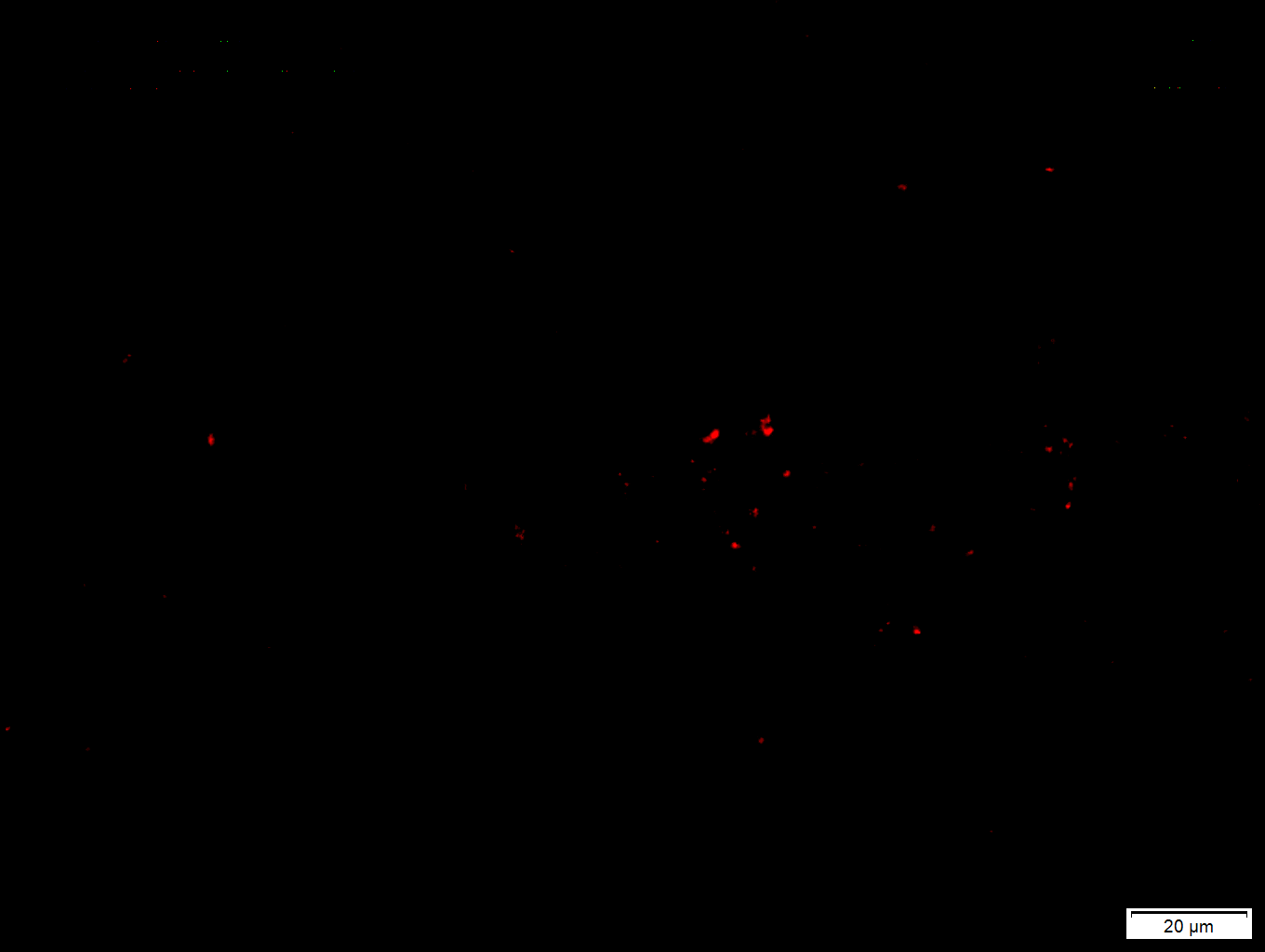

Supplement: Supplementary file 12 [file Data_Sheet_12.zip › Aβ immunofluorescence/DG/WT/K12/K12 DG.tif]

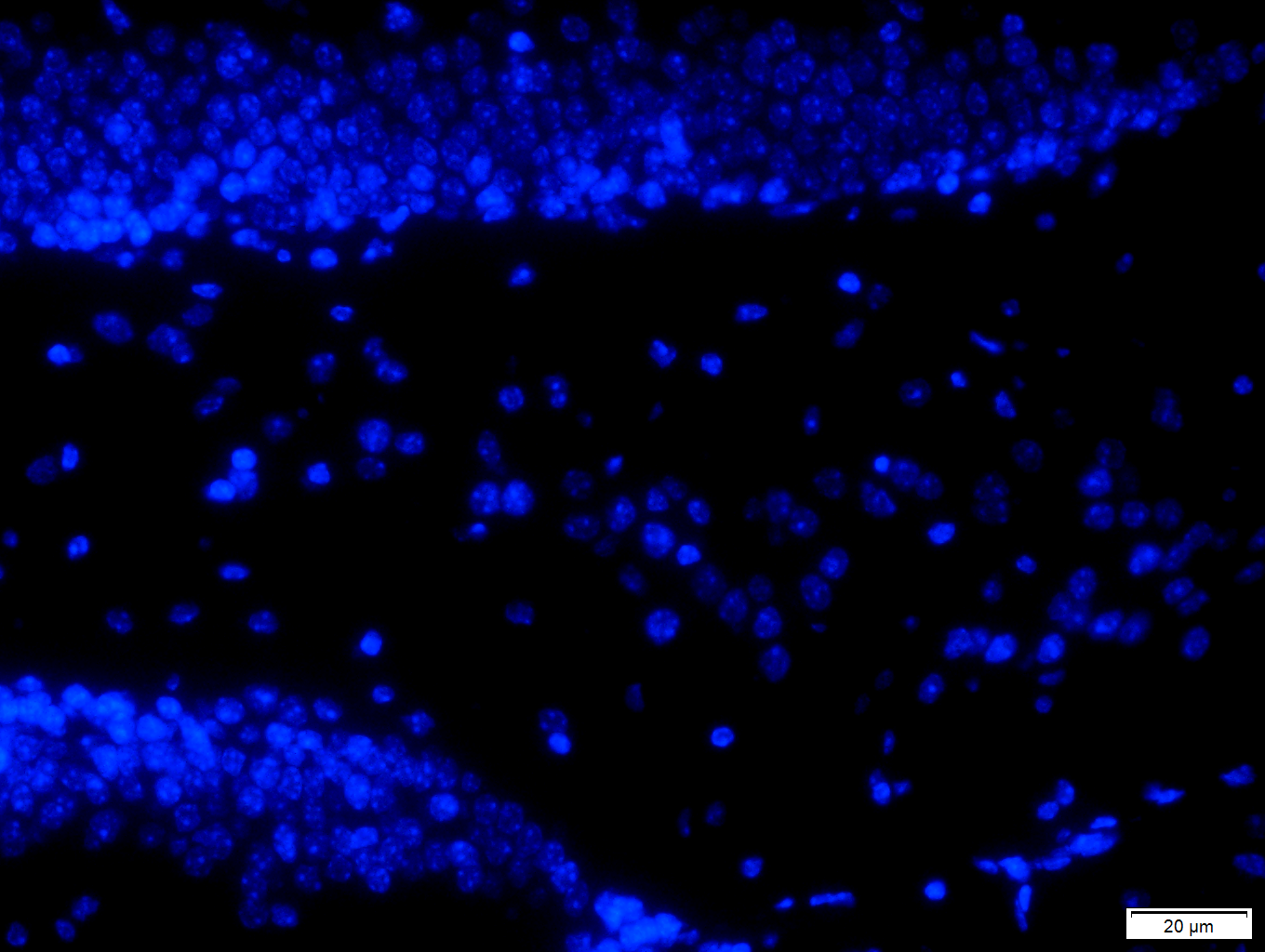

Supplement: Supplementary file 12 [file Data_Sheet_12.zip › Aβ immunofluorescence/DG/WT/K13/k13 dg dapi.tif]

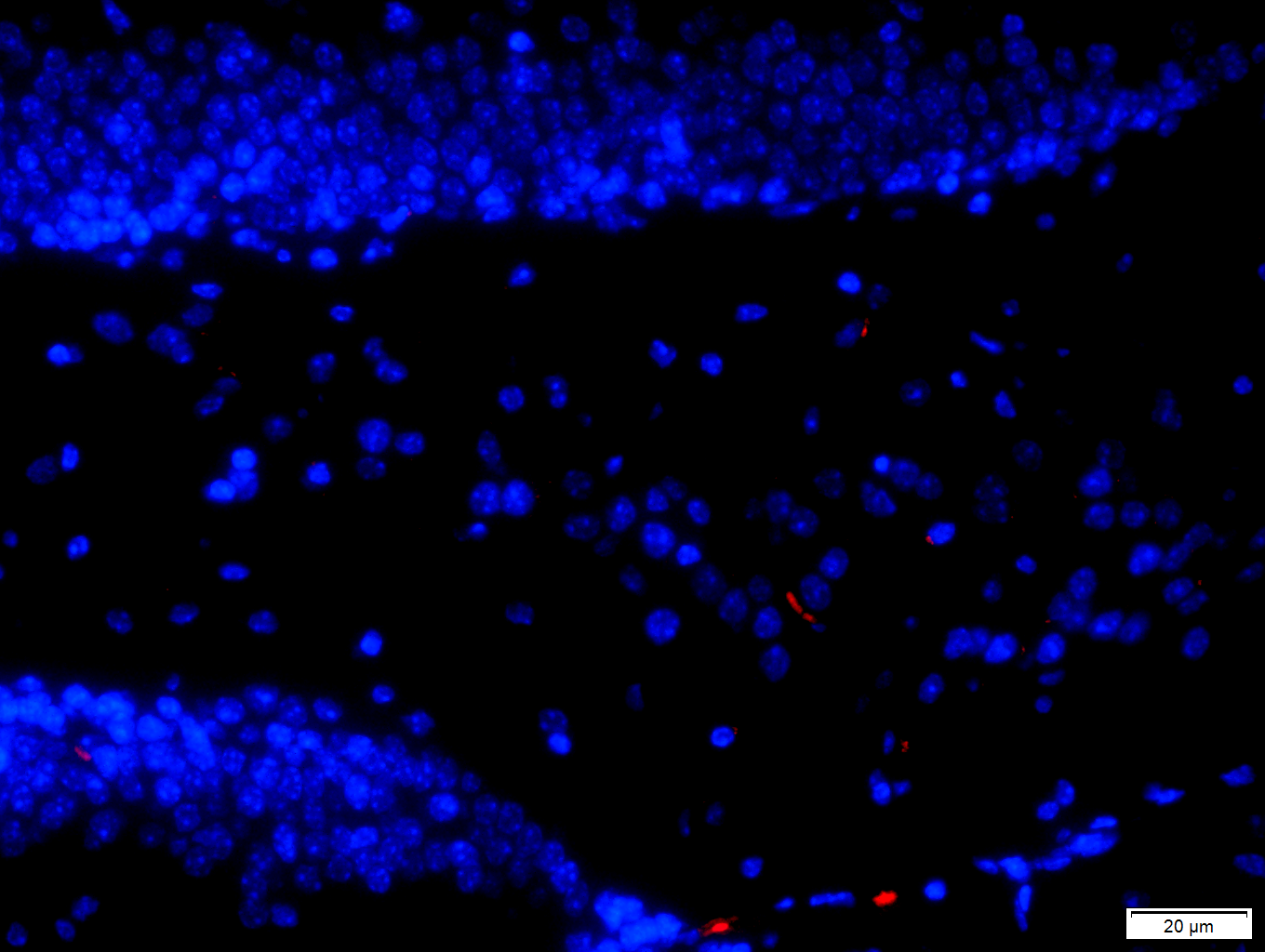

Supplement: Supplementary file 12 [file Data_Sheet_12.zip › Aβ immunofluorescence/DG/WT/K13/k13 dg merge.tif]

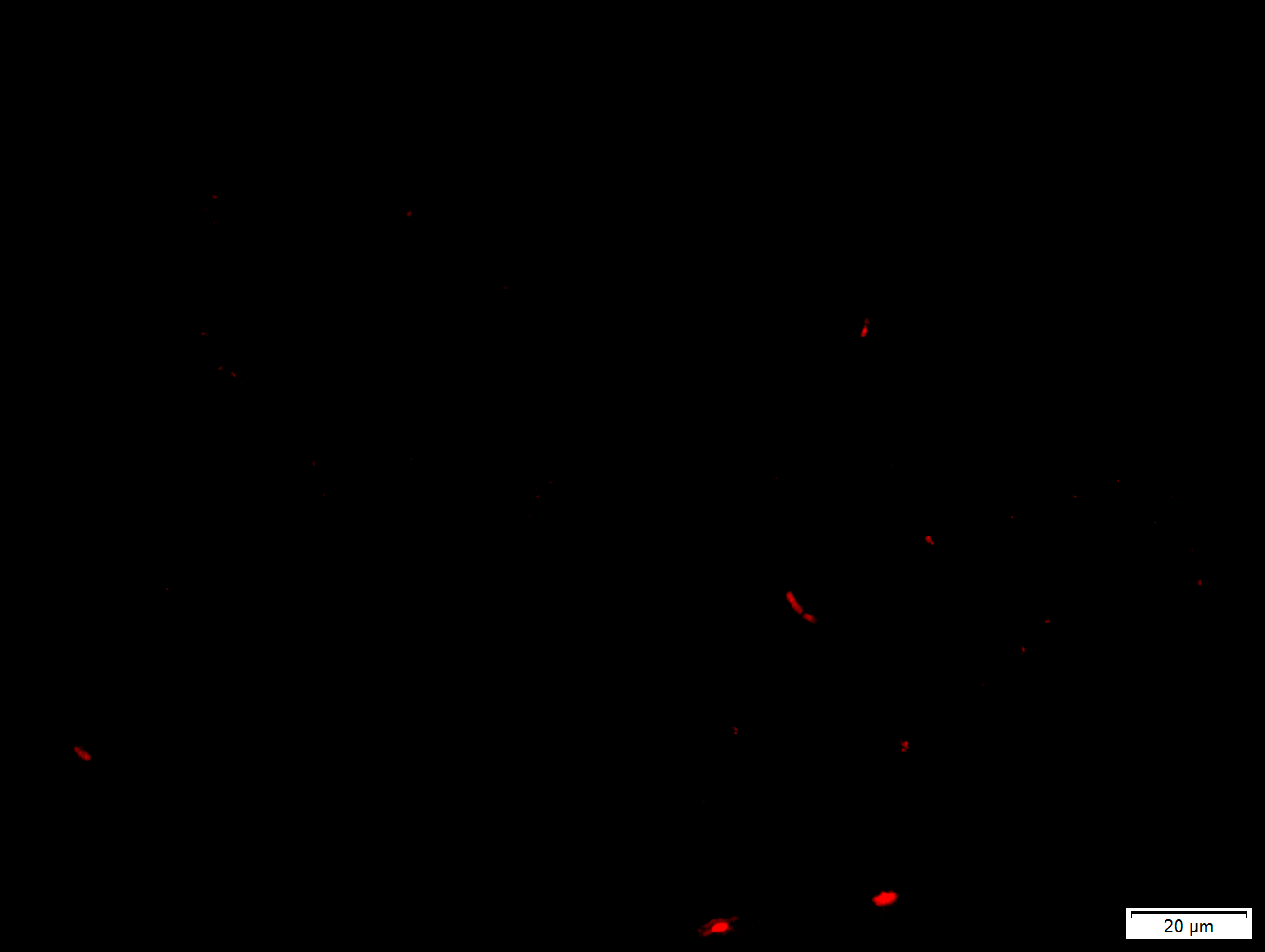

Supplement: Supplementary file 12 [file Data_Sheet_12.zip › Aβ immunofluorescence/DG/WT/K13/k13 dg.tif]
